# Access to chiral dihydrophenanthridines *via* a palladium(0)-catalyzed Suzuki coupling and C–H arylation cascade reaction using new chiral-bridged biphenyl bifunctional ligands†

**DOI:** 10.1039/d4sc00621f

**Published:** 2024-04-04

**Authors:** Bin Chen, Bendu Pan, Xiaobo He, Long Jiang, Albert S. C. Chan, Liqin Qiu

**Affiliations:** a School of Chemistry, IGCME, The Key Laboratory of Low-Carbon Chemistry & Energy Conservation of Guangdong Province, Guangdong Provincial Key Laboratory of Chiral Molecules and Drug Discovery, Sun Yat-sen University Guangzhou 510006 People's Republic of China qiuliqin@mail.sysu.edu.cn

## Abstract

A class of chiral-bridged biphenyl phosphine-carboxylate bifunctional ligands CB-Phos has been developed and successfully applied to Pd(0)-catalyzed single enantioselective C–H arylation and a one pot cascade reaction involving Suzuki cross-coupling and C–H arylation. The catalytic system provides a new and convenient way for the synthesis of versatile chiral dihydrophenanthridines with rich structures and broad functional group tolerance. Good to excellent yields with high enantioselectivities were generally achieved. The reaction mechanism of the cascade reaction was also preliminarily discussed.

## Abstract 2

New bifunctional ligands enable enantioselective C–H arylation and a one pot cascade reaction involving Suzuki cross-coupling and C–H arylation.

## Graphical abstract

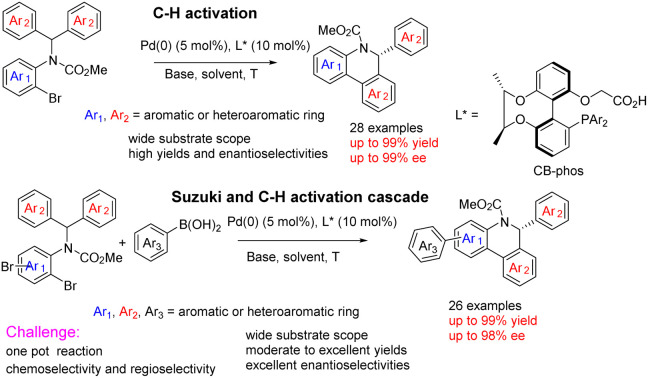

## Introduction

Selective catalytic activation and functionalization of C–H bonds using transition-metal complexes has broad synthetic potential because of its economic and ecological benefits.^1^ Recent impressive progress in this vibrant and fast advancing research area has opened up unimaginable opportunities for more effective strategic disconnection and streamlined synthesis,^2^ and catalytic enantioselective C–H activation has emerged as a simple and powerful method for constructing enantio-enriched molecules of high added value.^3^ Palladium(0)-catalyzed asymmetric intramolecular C–H functionalization generates cyclic products that allow access to four-,^4^ five-,^5^ six-,^6^ and seven-membered rings,^7^ which typically proceeds *via* a reversible carboxylate-assisted concerted metallation-deprotonation (CMD) mechanism.^8^ The enantio-determining step is usually C–H activation. Consistent with this mechanism, chiral auxiliary ligands and chiral bases have been successfully employed to induce enantioselectivity in palladium(0)-catalyzed C–H activation reactions.^4–7^ To our knowledge, a pioneering study by Baudoin and co-workers described the union of an ancillary ligand and the base in the same bifunctional molecule^9^ for directing palladium(0)-catalyzed enantioselective C–H arylation, generating bioactive dihydrophenanthridine derivatives.^10^ However, heterocyclic aromatic substrates were not studied in this catalytic system and their unsatisfactory enantioselective control in certain substrates still restricts their applications. On the other hand, single C–H arylation functionalization in the catalytic reaction limited the construction of versatile chiral dihydrophenanthridines.

**Scheme 1:**
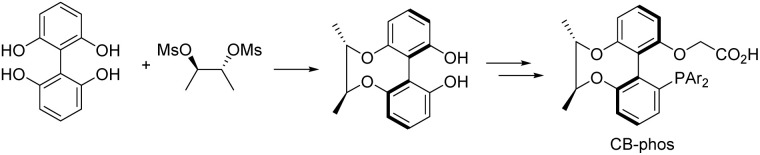
Axially chiral-bridged 2,2′-biphenyldiol-based phosphine-carboxylate bifunctional ligands CB-Phos.

**Scheme 2:**
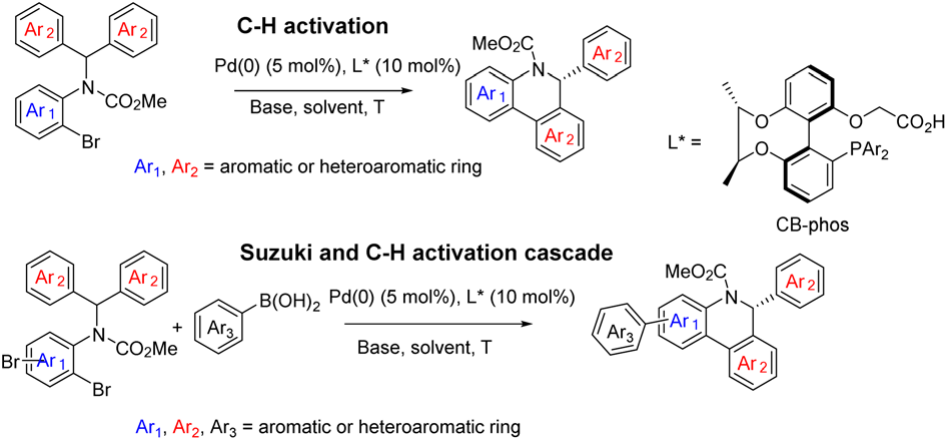
Asymmetric catalytic strategies for substituted chiral dihydrophenanthridine derivatives.

With the aid of asymmetric synthesis of axially chiral 2,2′-biphenyldiols *via* desymmetrization of prochiral tetrahydroxybiphenyl^11^ and other group's previous studies,^12^ our group has made effort toward the design and development of chiral ligand scaffolds using diastereoselective synthesis techniques.^13^ Due to the wide variety of substrates for asymmetric catalysis and the different demands for chiral ligands and corresponding catalysts, it is important and necessary to develop new classes of chiral ligands with novel features and explore their practical applications. Inspired by Baudoin's literature, we herein disclose the successful preparation of a new class of phosphine-carboxylate bifunctional ligands based on axially chiral-bridged 2,2′-biphenyldiol (named CB-Phos) (Scheme 1) and their application in the highly enantioselective synthesis of various bioactive chiral dihydrophenanthridine derivatives through C–H functionalization and cascade reaction strategies.^14^ The one-pot cascade reaction fully demonstrated good compatibility between palladium(0)-catalyzed C–H arylation and Suzuki cross-coupling reactions^15^ (Scheme 2).

## Results and discussion

Initial studies of C–H arylation were performed with model substrate 1a. A brief survey of some phosphines confirmed that ligands L1–L4 did not provide promising results for this transformation in the presence of a pivalic acid additive (Table 1, entries 1–4), while L5 showed excellent activity for the reaction with moderate enantioselectivity (Table 1, entry 5). We then introduced the chiral-bridged biphenyl bifunctional ligands L6–L11, which were prepared *via* complete asymmetric desymmetrization of 2,2′,6,6′-tetrahydroxybiphenyl without the need for a resolution process (Scheme 1 and ESI†). Pleasingly, ligand L6 furnished the desired product 2a in 93% yield and 86% ee in the absence of a carboxylic acid additive, and it is particularly noteworthy that no molecular sieves were required compared to the catalytic system in the literature^9*a*^ (Table 1, entry 6). Further refinement of reaction conditions was also performed using L6, including temperature, solvent and base (see the ESI†). At a temperature reduced to 80°C and a Cs_2_CO_3_ dosage of 1.5 equiv., the ee value of the product reached 92.2% with 92% yield (Table 1, entry 7). Ligand L7 with a 3,5-dimethylphenyl group attached to the phosphorus exhibited some improvement, affording 2a in 96% yield and 96.1% ee (Table 1, entry 8). The use of *p*-methoxyl-substituted ligand L8 resulted in an almost quantitative formation of 2a but with a slight decrease in enantioselectivity (Table 1, entry 9), which can be interpreted as that the *para*-electron-donating group methoxyl promoted the oxidative addition of Pd(0) with 1a and gave the anticipated palladium species, whereas employment of ligands L9 and L10 possessing bulky 3,5-di-*tert*-butylphenyl and 3,5-di-*tert*-butyl-4-methoxylphenyl groups had no improved income in yield and enantioselectivity (Table 1, entries 10–11). The performance of the *p*-methyl-substituted ligand L11 was worse than that of the parent ligand L7 (Table 1, entry 12).

**Table 1:** Selected optimization studies of C–H arylation^a^

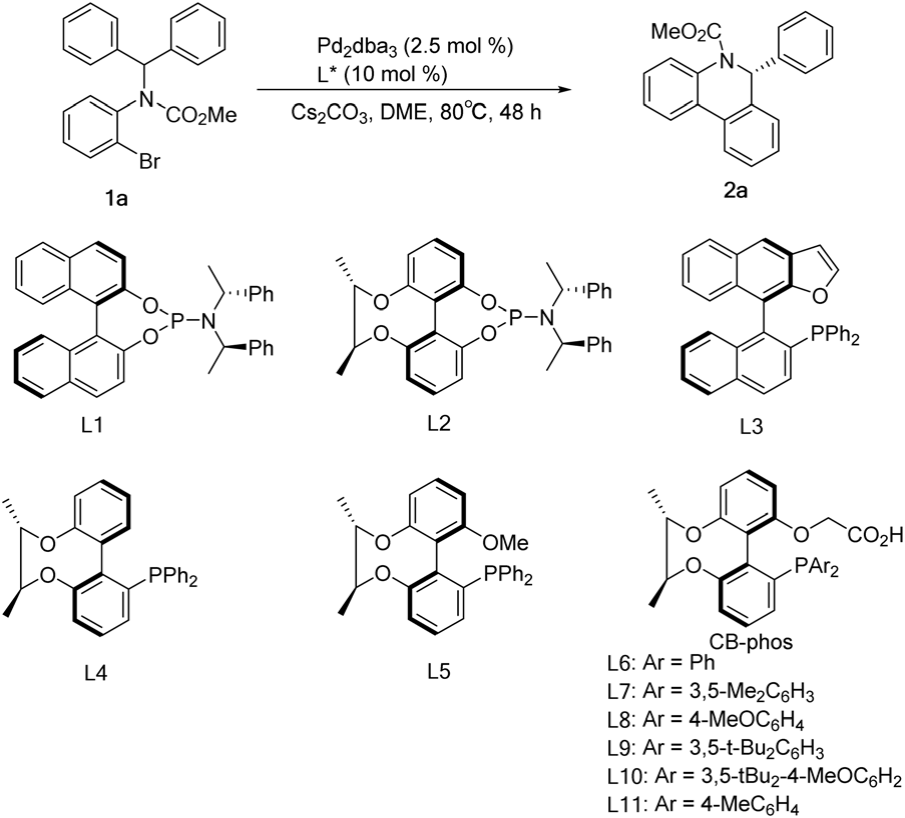			
Entry	L	Yield^c^ [%]	ee^d^ [%]
1^b^	L1	67	6.8
2^b^	L2	55	10.4
3^b^	L3	72	26.0
4^b^	L4	22	7.8
5^b^	L5	97	55.0
6^e^	L6	93	86.0
7	L6	92	92.2
8	L7	96	96.1
9	L8	99	93.6
10	L9	94	92.0
11	L10	71	90.6
12	L11	55	80.4

a Reaction conditions: 1a (0.1 mmol), Pd_2_dba_3_ (2.5 mol%), L* (10 mol%), Cs_2_CO_3_ (1.5 equiv.), 0.05 M in DME, 80 °C.

b Reaction conditions: 1a (0.1 mmol), Pd_2_dba_3_ (2.5 mol%), L* (10 mol%), Cs_2_CO_3_ (1.5 equiv.), pivalic acid (0.3 equiv.), 0.05 M in DME, 120 °C.

c Yield of the isolated product.

d The ee values were determined by HPLC analysis using a chiral stationary phase.

e Performed at 120 °C.

**Scheme 3:**
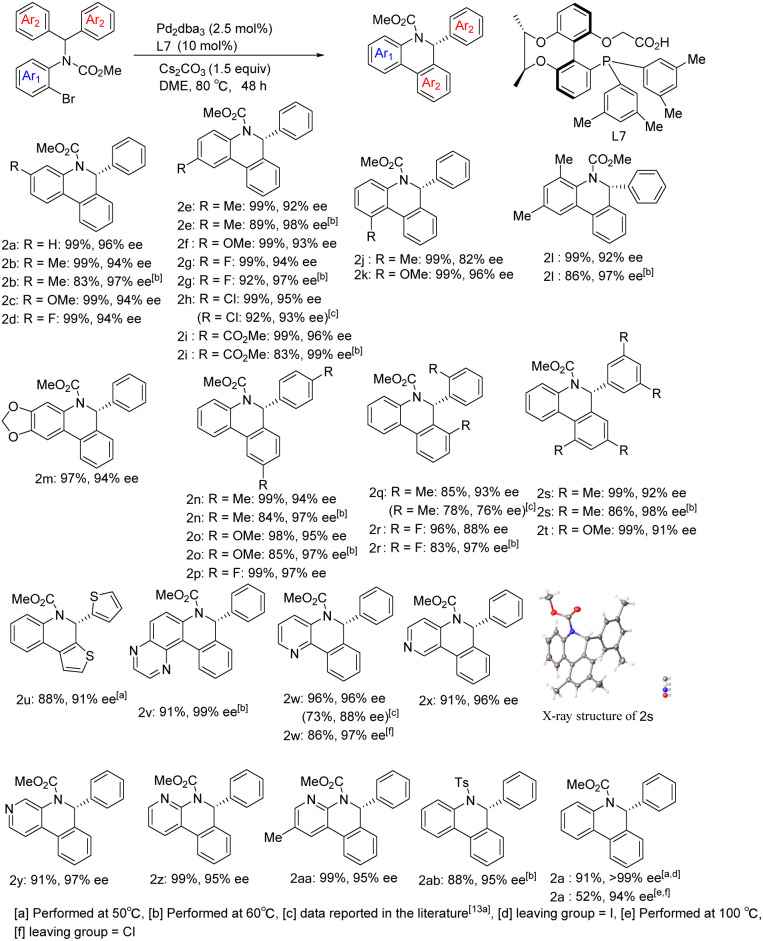
Scope of the enantioselective C–H arylation.

With optimized reaction conditions in hand, we began to examine the substrate scope of the reaction using Pd/L7 (Scheme 3). Various substituents at different positions of the aniline ring (Me, OMe, F, and Cl), regardless of their electronic properties, were well tolerated and resulted in good yields and excellent enantioselectivities (2b–2i). However, the sterically hindered *ortho*-methyl bromide substrate reduced the ee of product 2j. Substrates derived from polysubstituted anilines also performed well (2l and 2m). With respect to the substituent effect on the diaryl rings attached to the prochiral carbon atom, substrates with *para*- or *ortho*-substituent or 3,5-disubstituent moieties generated arylation products in excellent yields and enantiocontrol (2n–2t). Through single crystal X-ray diffraction analysis, the absolute configuration of 2s was determined to be *R*. Heteroaromatics were able to react at lower temperatures. Taking thiophene as an example, the corresponding product 2u was obtained with 88% yield and 91% ee. Notably, substrates containing potentially coordinating pyridine or pyrazine motifs still underwent C–H arylation smoothly, perfectly affording the corresponding products (2v–2aa) in 91–99% yield and 95–99% ee. In addition, using chlorine *in lieu* of bromine as the leaving group, the chloropyridine derivative also delivered product 2w in 86% yield and 97% ee under standard reaction conditions. It is worth noting that the reaction results of bromide substrates using our chiral-bridged biphenyl ligand L7 are superior to those reported in the literature with its binaphthyl-based counterpart^9*a*^ (2w: 96%, 96% ee *vs.* 73%, 88% ee; 2h: 99%, 95% ee *vs.* 92%, 93% ee; 2q: 85%, 93% ee *vs.* 78%, 76% ee), especially for product 2w with a nitrogen heterocyclic structure, which may be due to the smaller steric hindrance and better chiral regulation of our chiral-bridged ligand. The substrate derived from benzopyrazine provided new product 2v with almost perfect enantioselectivity and excellent yield even at lower temperature. All of these demonstrate the high effectiveness of our catalytic system in this class of heterocyclic substrate reactions. Apart from 2w, other products with heteroaromatic ring motifs (2u, 2v, and 2x–2z) are synthesized for the first time *via* this catalytic asymmetric approach, thus further extending the applicability of this catalytic system. Aside from the alkoxycarbonyl group on the nitrogen atom, the substrate bearing a tosyl group could also be transformed into the corresponding product (2ab) in 88% yield and 95% ee at 60 °C. With iodide instead of bromide as the leaving group, the reaction delivered product 2a in nearly quantitative yield and excellent enantioselectivity. Dropping the reaction temperature to 50 °C improved the enantioselectivity to 99% ee while maintaining an excellent yield. In contrast, by replacing bromide with chloride, the reaction became sluggish under standard reaction conditions. Further raising the reaction temperature to 100 °C, excellent enantioselectivity was obtained despite the moderate yield for the product, with a clear advantage of our ligand over its binaphthyl-based counterpart^9*a*^ (52% isolated yield and 94% ee at 100 °C *vs.* 16% NMR yield and 89% ee at 120 °C). In addition, to further confirm the potential of the enantioselectivity improvement, several substrates were chosen to react at a lower temperature. The results showed that the enantioselectivity further improved after reducing the temperature to 60 °C, despite a certain decrease in yield [2b (83%, 97% ee), 2e (89%, 98% ee), 2g (92%, 97% ee), 2i (83%, 99% ee), 2l (86%, 97% ee), 2n (84%, 97% ee), 2o (85%, 97% ee), 2r (83%, 97% ee), and 2s (86%, 98% ee)]. In general, the reactions achieved higher yields and enantioselectivities compared to those reported in the literature^9*a*^ employing our ligand L7 and the associated catalytic system.

After completing the aforementioned investigation on the C–H arylation reaction, we turned our attention to the cascade reaction. The feasibility of Suzuki coupling and the enantioselective C–H arylation cascade reaction was tested using the model substrates aryl dibromide 3a and phenylboronic acid 3b. Standard reaction conditions involve a combination of ligand (10 mol%) with Pd_2_dba_3_ (2.5 mol%), 3.0 equiv. of cesium carbonate and toluene as the solvent at 100 °C. Ligand L6, which had been successfully used for the above C–H arylation, enabled the cascade reaction in a moderate yield and at the expected level of enantioselectivity (Table 2, entry 1). Ligand L7 provided the reaction result similarly (Table 2, entry 2). *p*-Methoxyl-substituted ligand L8 showed better performance than L7 in the yield (Table 2, entry 3). The enantioselectivity of the cascade reaction using ligands L9 and L10 is still much lower, which is consistent with the trend in enantioselective C–H arylation reactions. However, the resulting yield is comparable to that with ligand L6 or L7, which is different from that in the C–H arylation (Table 2, entries 4–5). Usually, ligands with bulky aryl substituents linked to the phosphorus atom are more efficient in the Suzuki reaction, contrary to the requirement of C–H arylation transformation in this cascade reaction. These results indicate the need for careful screening and optimization of ligands to meet different demands in the cascade reaction. The reaction conditions, including temperature, base and palladium source, were further refined using L8 (see the ESI†). When the temperature dropped to 60 °C and the amount of Cs_2_CO_3_ was 3.0 eq., the ee value of the target product reached 97.0% in 77% yield. As the best solvent for the C–H arylation, 1,2-dimethoxyethane (DME) provided a slightly lower yield here (Table 2, entry 6). THF, a solvent commonly used in Suzuki reactions, proved to be inefficient enough when used here (Table 2, entry 7). DMSO was detrimental to this transformation, and no desired product was detected (Table 2, entry 8). Of note, replacement of toluene with DMF achieved a higher yield of the desired product, but the ee value dropped to 92.4% (Table 2, entry 9). Further mixing of DMF and toluene did not yield more positive results (Table 2, entries 10–11). Altogether, the results reveal a pronounced dependence of the yield and enantioselectivity of the desired cascade reaction on the ligand, palladium source, reaction temperature, solvent and base.

**Table 2:** Selected optimization of Suzuki coupling and C–H arylation cascade^a^

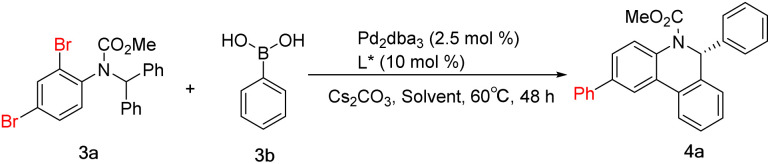				
Entry	L*	Solvent	Yield^b^ [%]	ee^c^ [%]
1	L6	Toluene	50.6	88.4
2	L7	Toluene	51.1	89.4
3	L8	Toluene	58	89.4
4	L9	Toluene	50	62.4
5	L10	Toluene	49.6	52.2
6	L8	DME	71.3	97.1
7	L8	THF	31	90.6
8	L8	DMSO	N.D.	—
9	L8	DMF	86	92.4
10	L8	DMF : toluene/1 : 3	77	96.6
11	L8	DMF : toluene/1 : 1	79	92.8

a Reaction conditions: 3a (0.1 mmol), 3b (1.1 equiv.), Pd_2_dba_3_ (2.5 mol%), L* (10 mol%), Cs_2_CO_3_ (3.0 equiv.), 0.05 M in the solvent, 60 °C.

b Yield of the isolated product.

c The ee values were determined by HPLC analysis using a chiral stationary phase.

**Scheme 4:**
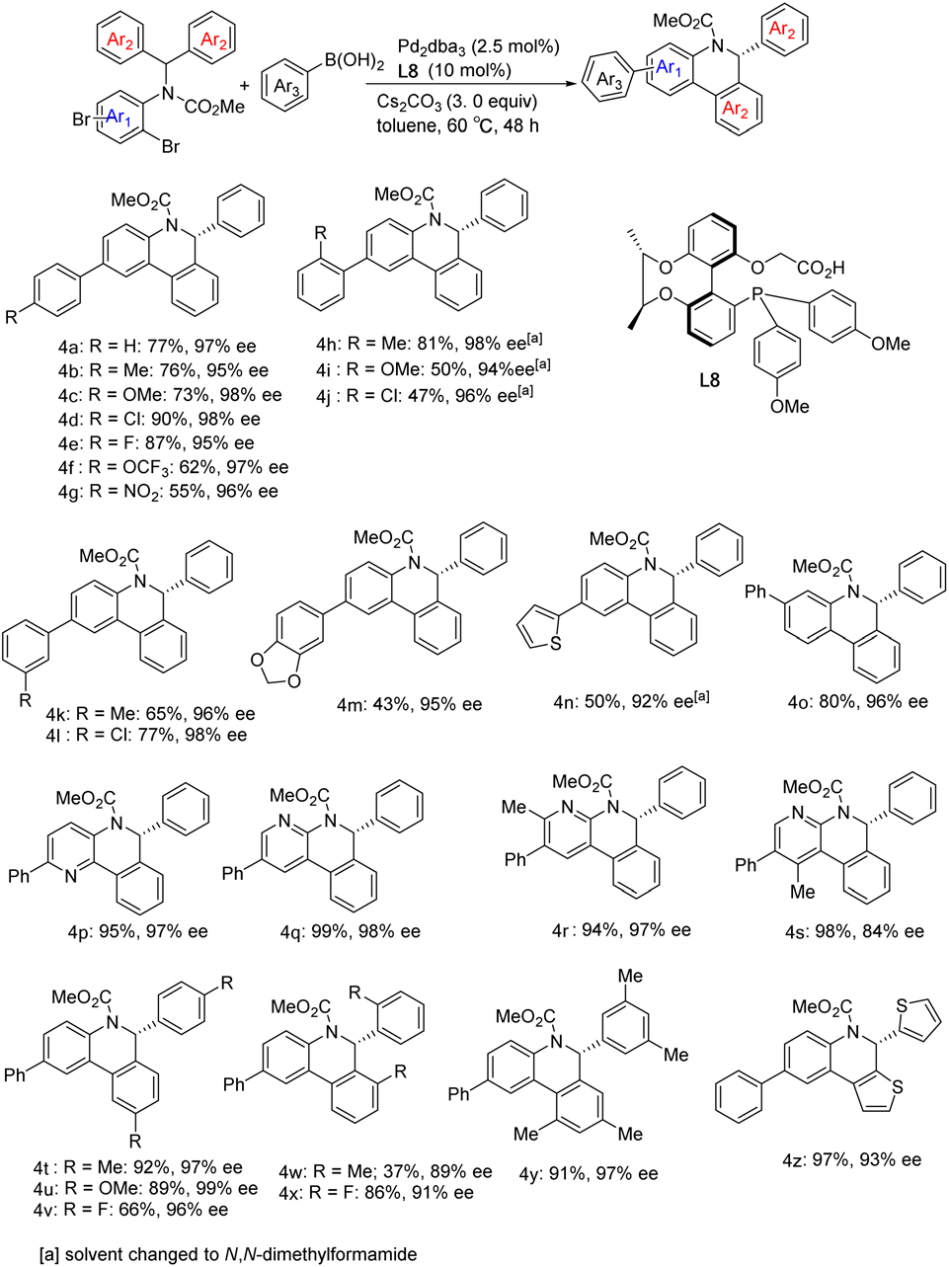
Scope of the Suzuki and C–H arylation cascade.

Considering the yield and enantioselectivity comprehensively, L8 was chosen as the optimal ligand to explore the substrate scope of the palladium-catalyzed Suzuki and C–H arylation cascade reaction. A series of aryl boronic acids bearing various substituents at different positions were used for the examination. For phenylboronic acids containing *para*- or *meta*-substituents, the reaction proceeded smoothly, affording the desired products 4b–4f in moderate to high yields and excellent enantioselectivities. A decrease in yield was observed with *para*-nitro-substituted phenylboronic acid, which may be caused by deboration during the reaction. However, employing *ortho*-substituted phenylboronic acid as the substrate also had an impact on the reaction rate. The solvent was switched to DMF to improve the yields of 4h–4j. The chlorine substituents in 4j and 4l can be conveniently introduced by chlorophenylboronic acid and remain untouched during the reaction, which can be further functionalized *via* general coupling reactions. Thiophene-2-boronic acid and 3,4-(methylenedioxy) phenylboronic acid furnished the corresponding products 4m and 4n in moderate yields. Changing the relative position of the dibromide substitution on the aniline moiety, the Suzuki reaction could also occur at the *para* position of the C–H arylation, giving product 4o in 80% yield and 96% ee. Substrates containing a pyridine motif were more reactive and delivered products 4p–4r in excellent yields and enantioselectivities. But for product 4s, despite the very high yield received, the ee value dropped to 84%. This demonstrates the effect of *ortho*-methyl on the enantioselectivity of the C–H arylation. As for the diaryl rings, substrates with *para*- or 3,5-disubstituent patterns were well tolerated, whereas, a large decrease in the yield of 4w containing *ortho*-methyl groups on the diphenyl rings was indeed observed. Even more gratifyingly, the thiophene substrate also reacted well, acquiring product 4z excellently (Scheme 4).

**Scheme 5:**
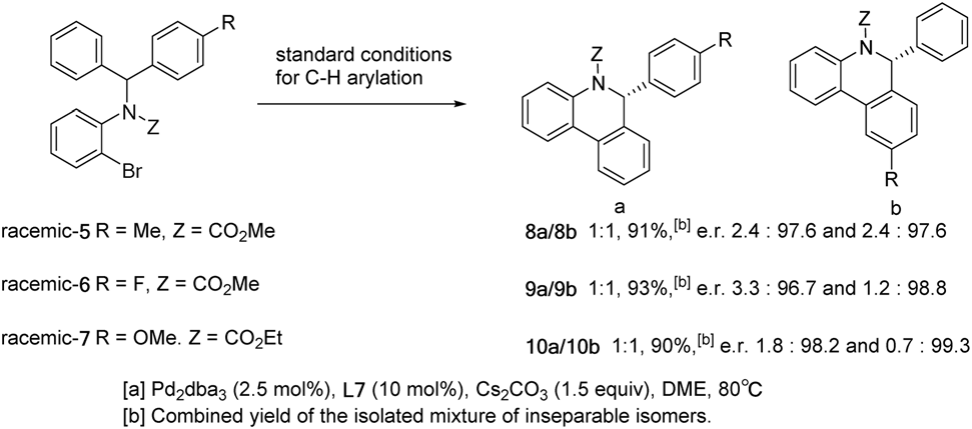
Parallel kinetic resolution of racemic substrates.

To explore the selectivity of the insertion reaction in C–H activation, we examined the parallel kinetic resolution of the racemic unsymmetrical diarylmethyl amine substrates 5, 6 and 7 (Scheme 5). Since the methoxycarbonyl group and the methoxy group attached to phenyl are difficult to distinguish in ^1^H NMR spectra, we replaced the methoxycarbonyl group on substrate 7 with ethoxycarbonyl. Under standard conditions for C–H arylation, substrates 5, 6 and 7 provided approximately 1 : 1 mixtures of highly enantioenriched isomers 8a/8b, 9a/9b and 10a/10b in high combined yields.

**Scheme 6:**
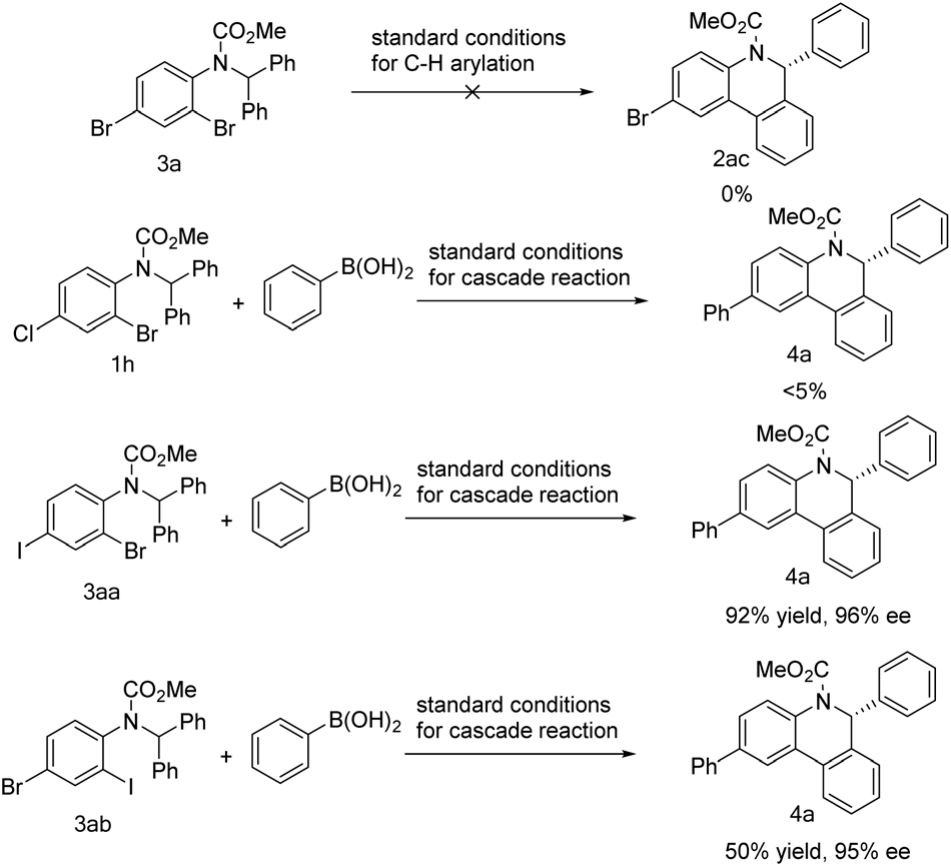
Behavior of different dihalogenated substrates in the cascade reaction.

Different dihalogenated substrates were further tested in the reaction (Scheme 6). Dibromide 3a failed to take part in the above C–H arylation. Substrate 1h that proceeded smoothly in the C–H arylation (Scheme 3, 2h) could only produce a small amount of the desired product 4a under standard cascade reaction conditions. In sharp contrast, iodide 3aa was shown to be competent in the cascade reaction and gave product 4a in high yield. However, after exchanging the relative positions of iodine and bromine, substrate 3ab brought about a significant decrease in yield.

**Scheme 7:**
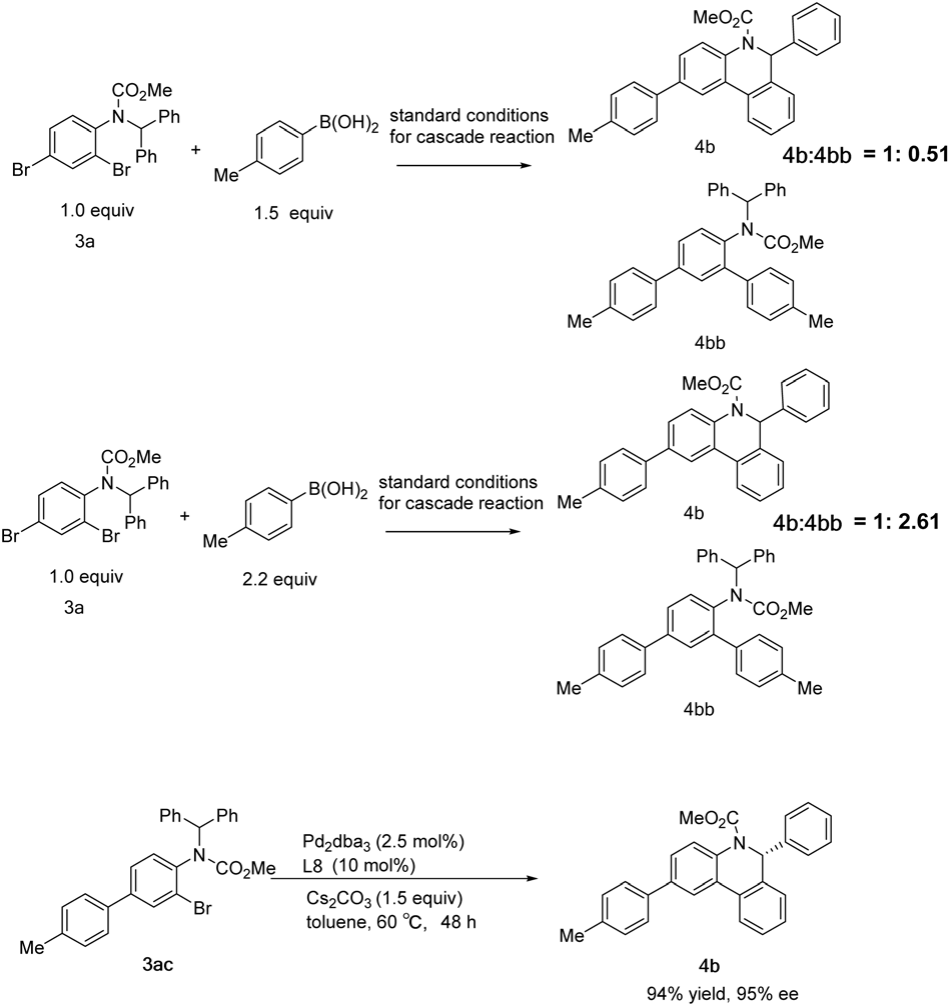
Rival side reaction in the cascade reaction.

Further exploration of the cascade reaction sequence was conducted. As the amount of *p*-tolylboronic acid was raised to 1.5 equiv., the by-product 4bb of the double Suzuki reaction increased, and the molar ratio of the desired product 4b to 4bb detected by NMR was 1 : 0.51, and the yield of 4b was 66%. On further increasing *p*-tolylboronic acid to 2.2 equiv., the molar ratio was reversed to 1 : 2.61 with 27% yield of 4b. Substrate 3ac was also readily converted to 4b with a yield of 94% and an ee of 95%. These results suggest that the cascade reaction is initiated by the Suzuki reaction of *para*-Br of aniline derivative 3a and the secondary Suzuki reaction competes with the intramolecular C–H arylation along with increasing arylboronic acid dosage (Scheme 7).

**Scheme 8:**
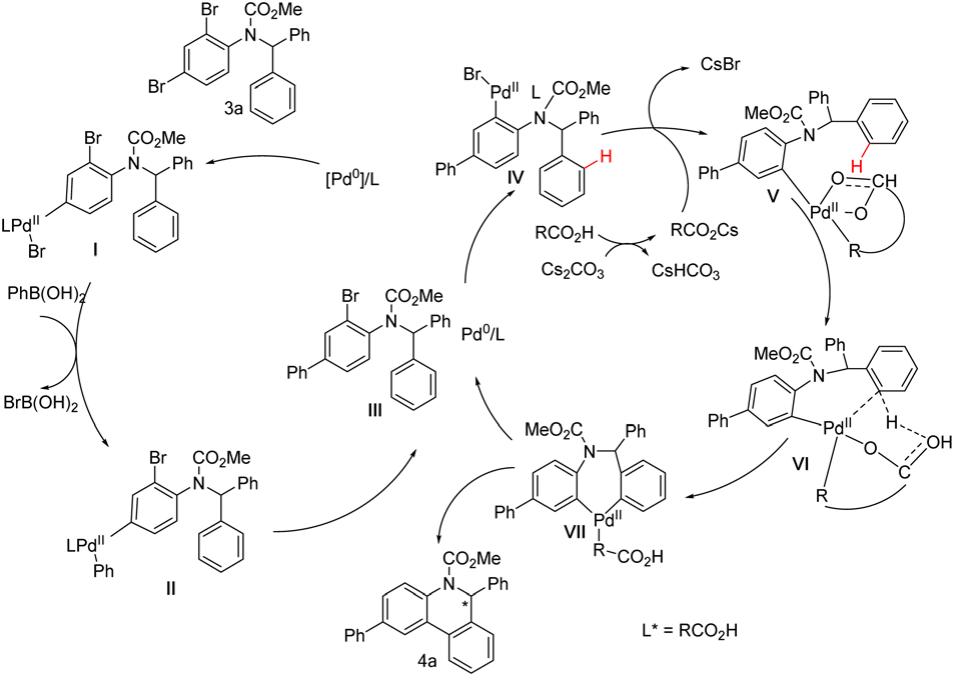
Plausible catalytic cycle for the cascade reaction.

Based on the above studies and general concepts, a brief catalytic cycle for the cascade reaction with 3a as the representative substrate is proposed in Scheme 8. First, an oxidative addition of Pd(0) to the *para*-C–Br bond of the amino group of 3a gives palladium species I, which will complete the whole Suzuki–Miyaura catalytic cycle and afford intermediate III. Then, a new oxidative addition adjacent to the amino group starts and generates palladium species IV, which undergoes C–H palladation to afford VI. The Pd coordinated with a chiral bifunctional ligand can discriminate between two aromatic rings and selectively form VII. Through reductive elimination, the target product 4a is ultimately obtained.

## Conclusion

In summary, we have developed a new class of chiral-bridged biphenyl phosphine-carboxylate bifunctional ligands and successfully applied them to the highly efficient synthesis of chiral dihydrophenanthridines through Pd(0)-catalyzed direct C–H enantioselective arylation. The catalytic system is compatible with various aryl substrates possessing electron-donating or electron-withdrawing groups, as well as heteroaryl substrates. On the basis of these results, the one-pot cascade reaction integrating Suzuki–Miyaura coupling and subsequent C–H arylation was further realized, and the target products with very high enantioselectivity and good to excellent yield were generally obtained. The method provides a new and convenient way for the synthesis of versatile chiral dihydrophenanthridines with abundant structures and broad functional group tolerance. The reaction mechanism of the cascade reaction was also preliminarily explored. More in-depth mechanistic research and more application development are currently ongoing in our laboratory.

## Data availability

The data underlying this study are available in the published article and its ESI.†

## Author contributions

Bin Chen conceived the idea and designed and performed most of the experiment, Bendu Pan and Xiaobo He synthesized some of the starting materials, Long Jiang helped to analyze single crystal X-ray diffraction, Albert S. C. Chan conceived the idea, and Liqin Qiu conceived the idea and supervised the research.

## Conflicts of interest

There are no conflicts to declare.

## Supplementary Material

SC-015-D4SC00621F-s001

SC-015-D4SC00621F-s002

## References

[cit1] Dyker G. (1999). Angew. Chem., Int. Ed..

[cit2] Johnson J. A., Li N., Sames D. (2002). J. Am. Chem. Soc..

[cit3] Newton C. G., Wang S.-G., Oliveira C. C., Cramer N. (2017). Chem. Rev..

[cit4] Pedroni J., Boghi M., Saget T., Cramer N. (2014). Angew. Chem., Int. Ed..

[cit5] Albicker M. R., Cramer N. (2009). Angew. Chem., Int. Ed..

[cit6] Ma X., Gu Z. (2014). RSC Adv..

[cit7] Saget T., Cramer N. (2013). Angew. Chem., Int. Ed..

[cit8] Ackermann L. (2011). Chem. Rev..

[cit9] Yang L., Neuburger M., Baudoin O. (2018). Angew. Chem., Int. Ed..

[cit10] Wulff H., Zhorov B. S. (2008). Chem. Rev..

[cit11] Tuyet T. M. T., Harada T., Hashimoto K., Hautsuda M., Oku A. (2000). J. Org. Chem..

[cit12] Uozumi Y., Hayashi T. (1991). J. Am. Chem. Soc..

[cit13] Qiu L., Qi J., Pai C.-C., Chan S., Zhou Z., Choi M. C. K., Chan A. S. C. (2002). Org. Lett..

[cit14] Fogg D. E., dosSantos E. N. (2004). Coord. Chem. Rev..

[cit15] Miyaura N., Suzuki A. (1995). Chem. Rev..

