# Supplementary material for: Access to chiral dihydrophenanthridines via a palladium(0)-catalyzed Suzuki coupling and C–H arylation cascade reaction using new chiral-bridged biphenyl bifunctional ligands
Source: Chem Sci. 2024 Apr 4;15(18):6884–90. doi: 10.1039/d4sc00621f (PMC11077526; doi:10.1039/d4sc00621f)
Supplement: SC-015-D4SC00621F-s001 [file SC-015-D4SC00621F-s001.pdf]

Electronic Supplementary Material (ESI) for Chemical Science.
This journal is © The Royal Society of Chemistry 2024

Supporting information

Access to Chiral Dihydrophenanthridines via a Palladium(0)-Catalyzed Suzuki Coupling and C-H Arylation Cascade Reaction Using New Chiral-Bridged Biphenyl Bifunctional Ligands

Bin Chen,[a] Bendu Pan,[a] Xiaobo He,[a] Long Jiang,[a] Albert S. C. Chan,[a] Liqin Qiu*[a]

a B. Chen, B. Pan, X. He, Prof. Dr. L. Jiang, Prof. Dr. A. S. C. Chan, Prof. Dr. L. Qiu
School of Chemistry, IGCME, The Key Laboratory of Low-Carbon Chemistry & Energy Conservation of Guangdong Province,
Guangdong Provincial Key Laboratory of Chiral Molecules and Drug Discovery
Sun Yat-sen University
Guangzhou 510006, People's Republic of China.
*E-mail: qiuliqin@mail.sysu.edu.cn

Contents

General Considerations 2

Experimental Section 2

Reference 39

NMR Spectra 39

HPLC Data 158

General Considerations

Unless otherwise noted, all reactions were carried out in a nitrogen-filled glove box or under nitrogen atmosphere using standard Schlenk techniques. Commercially available compounds were purchased from commercial suppliers and directly used without further purification. Solvents were dried and degassed according to standard procedures. The heat source for all reactions is oil bath. Column chromatography was carried out using silica gel (200-300 mesh). ^{1}H NMR, ^{19}F NMR, ^{31}P NMR and ^{13}C NMR spectra were recorded on a Bruker Avance III 400MHz spectrometer (400, 376, 162 and 100 MHz respectively). High-resolution mass spectra (HRMS) were obtained with Thermo Q Exactive mass spectrometer. Optical rotations were measured on a Perkin-Elmer 341 polarimeter. Enantiomeric excesses (ee values) of the products were determined by chiral HPLC analysis using an Aglient HP 1200 instrument (n-hexane/2-propanol as eluent) with a Chiral IA-3, IB-, IC-3, IE-3, OD-H or OJ-H column.

Experimental Section

(1) Preparation of ligands

General procedure A for the synthesis of **L6-L11**

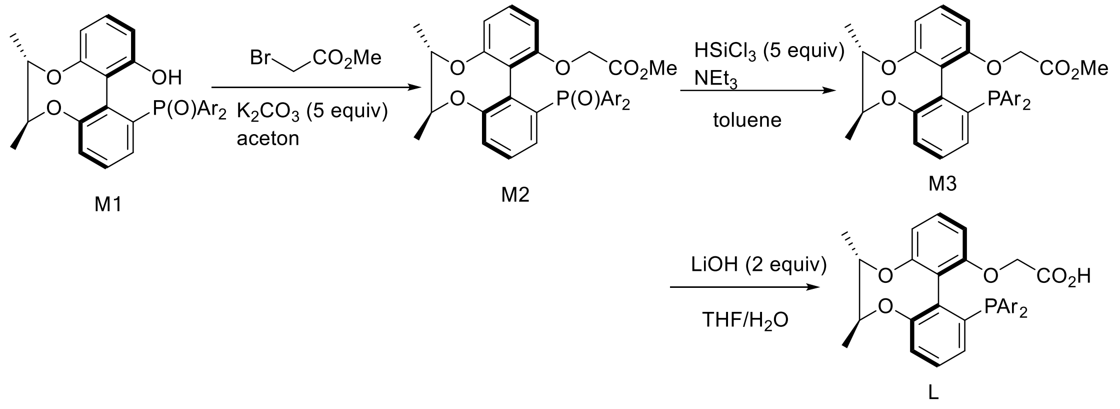

The preparation method of **M1** can referred to references [1, 2]

Under the protection of N_2, to a mixture of **M1** (1.5 mmol, 1 equiv) and K_2CO_3 (5 equiv) in anhydrous acetone (15 mL/mmol), methyl bromoacetate (5-8 equiv) was added. The mixture was refluxed for 12-24 h. After completion of the reaction (monitored by TLC), the system was cooled to room temperature, the mixture was filtered and concentrated to give the crude product **M2**.

The crude ester **M2** (1 equiv) was dissolved in dry toluene (15 mL/mmol), then triethylamine (11-15 equiv) was added to the solution under N_2. The system was cooled to 0 ℃, then trichlorosilane (4 equiv) was added via syringe. The reaction was heated and refluxed overnight until the reaction was complete (monitored by TLC). Upon cooling to room temperature, the reaction was diluted with toluene and a solution of saturated aqueous $NaHCO_3$ was then added, and the mixture was stirred for 20 min. The resulting suspension was filtered through a pad of celite and washed with toluene. The combined filtrate was dried over Na_2SO_4. The solvent was

then removed by rotary evaporation under vacuum to obtain the crude solid product of **M3** as a white foam, which was passed through a short pad of silica gel for purification using petroleum ether/ethyl acetate as an eluent if needed.

Under N_2, the above intermediate **M3** (1 equiv) was added to a tube with LiOH (2 equiv) and a mixture of THF/H_2O (1:1, 10 mL/mmol). The reaction was stirred at room temperature overnight. To the resulting solution aqueous HCl was added and the mixture was acidified (pH = 2), followed by quick extraction with ethyl acetate. The combined organic phase was dried over Na_2SO_4. The concentrated residue was then purified by flash column chromatography over silica gel to get the desired chiral ligand.

(*R*)-2-(6,6'-(2*S*,3*S*-butadioxyl)-2'-(diphenylphosphaneyl)-[1,1'-biphenyl]-2-yl) oxy) acetic acid (L6)

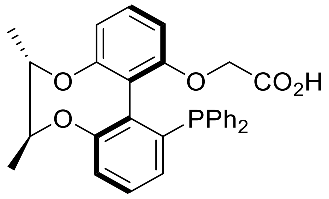

L6 was obtained according to the General procedure A, as a white solid (76% yield over three steps).

^{1}H NMR (400 MHz, $CDCl_3$) δ 11.38 (brs, 1H), 7.41-7.29 (m, 6H), 7.24-7.14 (m, 5H), 7.07-7.03 (m, 2H), 6.80-6.77 (m, 1H), 6.58-6.55 (m, 2H), 4.67 (d, *J* = 16.7 Hz, 1H), 4.61 (d, *J* = 16.7 Hz, 1H), 3.87-3.73 (m, 2H), 1.36 (d, *J* = 6.3 Hz, 3H), 1.31 (d, *J* = 6.2 Hz, 3H). ^{13}C NMR (101 MHz, $CDCl_3$) δ 170.50, 160.65, 159.41, 159.33, 153.27, 137.06, 134.77, 134.31, 134.12, 133.36, 133.17, 130.35, 129.53, 129.51, 129.33, 128.81, 128.77, 128.70, 128.09, 128.01, 122.97, 119.43, 116.06, 106.35, 99.98, 86.70, 85.90, 65.59, 18.99, 18.92 (observed complexity due to P-C splitting). $[\alpha]_D^{25}$ = -76.2 (*c* = 1.0 mg/mL in $CHCl_3$). Melting point: 118-119 °C. ^{31}P NMR (162 MHz, $CDCl_3$) δ -5.85. ESI-HRMS calcd. for $C_{30}H_{27}O_5P$ $[M+H]^+$ = 499.1669; found 499.1662. IR (neat): ν (cm^{-1}) 2230, 1705, 1448, 1322, 1248, 1018, 843, 738, 694, 615.

(*R*)-2-(6,6'-(2*S*,3*S*-butadioxyl)-2'-(bis(3,5-dimethylphenyl)phosphaneyl)-[1,1'-biphenyl]-2-yl) oxy)acetic acid (L7)

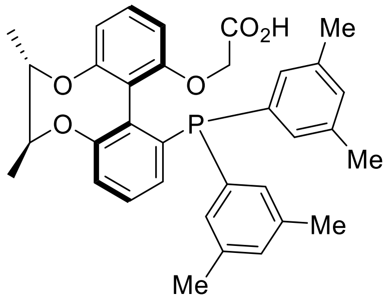

L7 was obtained according to the General procedure A, as a white solid (68% yield over three steps).

^{1}H NMR (400 MHz, $CDCl_3$) δ 11.89 (brs, 1H), 7.32-7.28 (m, 1H), 7.18-7.10 (m, 2H), 7.06-7.00 (m, 3H), 6.80 (s, 1H), 6.70 (t, *J* = 6.4 Hz, 1H), 6.63 (s, 1H), 6.61 (s, 1H), 6.53 (d, *J* = 8.4 Hz, 1H), 6.48 (d, *J* = 8.1 Hz, 1H), 4.72 (d, *J* = 16.8 Hz, 1H), 4.67 (d, *J* = 16.8 Hz, 1H), 3.87-3.71 (m, 2H), 2.31 (s, 6H), 2.15 (s, 6H), 1.37 (d, *J* = 6.3 Hz, 3H), 1.32 (d, *J* = 6.2 Hz, 3H). ^{13}C NMR (101 MHz,

$CDCl_3$) δ 170.84, 160.50, 159.28, 153.01, 138.28, 138.20, 137.62, 137.28, 137.19, 133.93, 133.21, 132.41, 132.22, 131.52, 131.46, 131.30, 130.96, 130.78, 130.52, 130.07, 129.44, 129.41, 128.31, 122.52, 119.48, 115.83, 106.11, 86.80, 85.59, 65.59, 21.30, 20.99, 19.09, 19.00 (observed complexity due to P-C splitting). ^{31}P NMR (162 MHz, $CDCl_3$) δ -4.36. $[\alpha]_D^{25}$ = -125.3 (*c* = 1.0 mg/mL in $CHCl_3$). Melting point: 125-127 °C. ESI-HRMS calcd. for $C_{34}H_{35}O_5P$ $[M+H]^+$ = 555.2295; found 555.2286. IR (neat): ν (cm^{-1}) 2976, 1685, 1449, 1325, 1271, 1049, 879, 737, 695, 618.

(*R*)-2-(6,6'-(2*S*,3*S*-butadioxyl)-2'-(bis(4-methoxyphenyl)phosphaneyl)-[1,1'-biphenyl]-2-yl)oxy)acetic acid (L8)

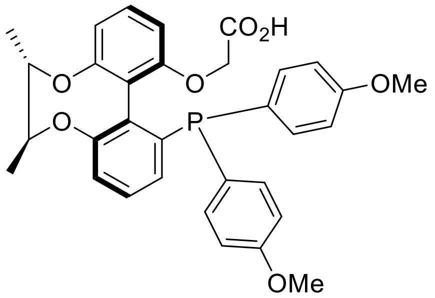

L8 was obtained according to the General procedure A, as a white solid (73% yield over three steps).

^{1}H NMR (400 MHz, $CDCl_3$) δ 11.79 (brs, 1H), 7.32-7.26 (m, 4H), 7.18-7.14 (m, 2H), 6.97-6.92 (m, 4H), 6.77-6.74 (m, 1H), 6.69-6.66 (m, 1H), 6.56-6.52 (m, 2H), 4.70 (d, *J* = 16.7 Hz, 1H), 4.66 (d, *J* = 16.7 Hz, 1H), 3.89-3.70 (m, 8H), 1.35 (d, *J* = 6.3 Hz, 3H), 1.30 (d, *J* = 6.3 Hz, 3H). ^{13}C NMR (101 MHz, $CDCl_3$) δ 170.72, 160.72, 160.56, 160.16, 159.36, 159.28, 153.16, 138.09, 135.88, 135.68, 134.79, 134.59, 131.49, 131.27, 130.24, 129.37, 129.34, 128.32, 125.60, 122.64, 119.50, 116.00, 114.50, 114.42, 113.81, 113.72, 106.17, 86.64, 85.83, 65.61, 55.22, 55.10, 18.99, 18.94 (observed complexity due to P-C splitting). ^{31}P NMR (162 MHz, $CDCl_3$) δ -8.68. $[\alpha]_D^{25}$ = -114.4 (*c* =1.0 mg/mL in $CHCl_3$). Melting point: 115-117 °C. ESI-HRMS calcd. for $C_{32}H_{31}O_7P$ $[M+H]^+$ = 559.1880; found 559.1871. IR (neat): ν (cm^{-1}) 2932, 1760, 1593, 1497, 1440, 1242, 1176, 1044, 1027, 940, 825, 793, 725, 616

(*R*)-2-(6,6'-(2*S*,3*S*-butadioxyl)-2'-(bis(3,5-di-tert-butylphenyl)phosphaneyl)-[1,1'-biphenyl]-2-yl)oxy)acetic acid (L9)

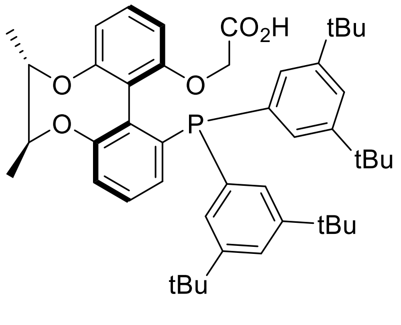

L9 was obtained according to the General procedure A, as a white solid (62% yield over three steps).

^{1}H NMR (400 MHz, $CDCl_3$) δ 11.93 (brs, 1H), 7.45 (s, 1H), 7.31-7.24 (m, 4H), 7.16-7.09 (m, 2H),

6.83 (d, *J* = 9.9 Hz, 2H), 6.77-6.73 (m, 1H), 6.57 (d, *J* = 8.4 Hz, 1H), 6.40 (d, *J* = 8.1 Hz, 1H), 4.78 (d, *J* = 16.8 Hz, 1H), 4.72 (d, *J* = 16.8 Hz, 1H), 3.83-3.64 (m, 2H), 1.34 (d, *J* = 6.2 Hz, 3H), 1.28 (s, 18 H), 1.24 (d, *J* = 6.3 Hz, 3H), 1.18 (s, 18 H). ^{13}C NMR (101 MHz, $CDCl_3$) δ 170.94, 160.55, 159.37, 159.29, 153.27, 150.89, 150.81, 149.92, 149.84, 138.97, 133.38, 132.68, 131.18, 130.97, 130.16, 129.19, 129.15, 128.64, 128.45, 128.09, 127.73, 127.53, 123.44, 123.30, 122.27, 119.70, 119.66, 115.85, 106.04, 86.81, 86.02, 65.76, 34.93, 34.65, 31.36, 31.23, 19.03, 18.96 (observed complexity due to P-C splitting). ^{31}P NMR (162 MHz, $CDCl_3$) δ -2.76. $[\alpha]_D^{25}$ = -78.1 (*c* = 1.0 mg/mL in $CHCl_3$). Melting point: 169-171 °C. ESI-HRMS calcd. for $C_{46}H_{59}O_5P$ $[M+H]^+$ = 723.4173; found 723.4163. IR (neat): ν (cm^{-1}) 2963, 1765, 1582, 1442, 1361, 1248, 1046, 873, 773, 709.

(*R*)-2-(6,6'-(2*S*,3*S*-butadioxyl)-2'-(bis(3,5-di-tert-butyl-4-methoxyphenyl)phosphaneyl)-[1,1'-biphenyl]-2-yl)oxy)acetic acid (L10)

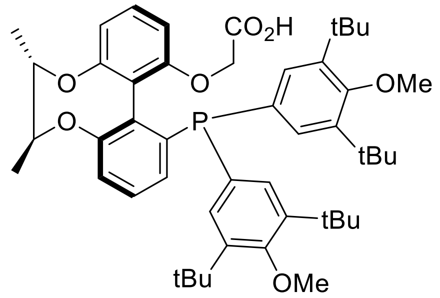

L10 was obtained according to the General procedure A, as a white solid (67% yield over three steps).

^{1}H NMR (400 MHz, $CDCl_3$) δ 12.03 (brs, 1H), 7.32-7.29 (m, 1H), 7.31 (t, *J* = 7.9 Hz, 1H), 7.25 (s, 1H), 7.23 (s, 1H), 7.15-7.11 (m, 2H), 6.86 (s, 1H), 6.83 (s, 1H), 6.75 (t, *J* = 6.3 Hz, 1H), 6.58 (d, *J* = 8.4 Hz, 1H), 6.41 (d, *J* = 8.1 Hz, 1H), 4.78 (d, *J* = 16.8 Hz, 1H), 4.72 (d, *J* = 16.8 Hz, 1H), 3.79-3.60 (m, 8H), 1.37 (s, 18H), 1.32 (d, *J* = 6.2 Hz, 3H), 1.26 (s, 18H), 1.23 (d, *J* = 6.4 Hz, 3H). ^{13}C NMR (101 MHz, $CDCl_3$) δ 170.96, 160.74, 160.59, 160.54, 159.43, 159.35, 153.33, 143.96, 143.89, 142.94, 142.85, 139.46, 132.90, 132.70, 132.43, 132.22, 130.83, 130.62, 130.18, 129.14, 129.10, 127.52, 127.39, 122.20, 119.68, 115.84, 106.10, 86.76, 86.07, 65.75, 64.28, 64.25, 35.89, 35.59, 32.01, 31.74, 19.09, 18.95 (observed complexity due to P-C splitting). ^{31}P NMR (162 MHz, $CDCl_3$) δ -5.09. $[\alpha]_D^{25}$ = -71.3 (*c* = 1.0 mg/mL in $CHCl_3$). Melting point: 220-223 °C. ESI-HRMS calcd. for $C_{48}H_{63}O_7P$ $[M+H]^+$ = 783.4384; found 783.4374. IR (neat): ν (cm^{-1}) 2957, 1766, 1445, 1314, 1221, 1112, 1007,939, 790, 784, 609.

(*R*)-2-(6,6'-(2*S*,3*S*-butadioxyl)-2'-(bis(4-methylphenyl)phosphaneyl)-[1,1'-biphenyl]-2-yl)oxy) acetic acid (L11)

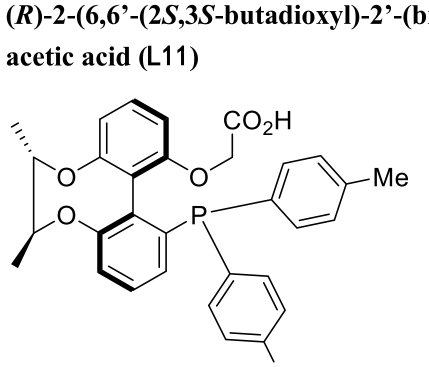

L11 was obtained according to the General procedure A, as a white solid (72% yield over three

steps).
^{1}H NMR (400 MHz, $CDCl_3$) δ 7.33-7.17 (m, 7H), 6.98-6.92 (m, 4H), 6.83-6.80 (m, 1H), 6.58-6.56 (m, 2H), 4.67 (d, J = 16.8 Hz, 1H), 4.64 (d, J = 16.8 Hz, 1H), 3.87-3.74 (m, 2H), 2.40 (s, 3H), 2.28 (s, 3H), 1.37 (d, J = 6.3Hz, 3H), 1.32 (d, J = 6.1 Hz, 3H). ^{13}C NMR (101 MHz, $CDCl_3$) δ 170.74, 160.65, 159.39, 159.30, 153.21, 134.26, 134.07, 133.21, 133.03, 130.40, 129.67, 129.59, 129.53, 129.49, 128.99, 128.90, 123.16, 115.98, 106.29, 86.70, 85.90, 65.59, 21.40, 21.31, 19.00, 18.94 (observed complexity due to P-C splitting). ^{31}P NMR (162 MHz, $CDCl_3$) δ -7.36. $[\alpha]_D^{25}$ = 102.5 (c = 1.0 mg/mL in $CHCl_3$). Melting point: 120-123 ºC. ESI-HRMS calcd. for $C_{32}H_{31}O_5P$ $[M+H]^+$ = 527.1982; found 527.1973. IR (neat): ν (cm^{-1}) 2948, 1691, 1454, 1254, 774, 713, 651.

(2) Synthesis of substrates

General procedure B: nucleophilic substitution reaction [2]

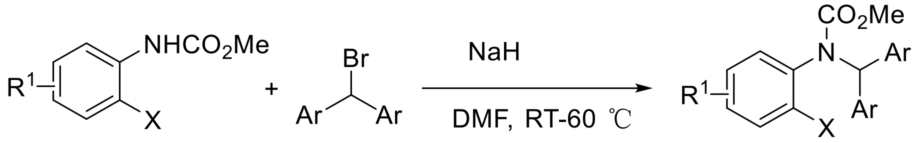

N-aryl-carbamate derivative (3 mmol, 1 equiv) was dissolved in dry DMF, then NaH (1.3-1.5 equiv) was added to the solution and the mixture was stirred at room temperature for 20 min. Bromodiarylmethane (1.5 equiv) was added to the mixture, then the system was heated to 60℃ and stirred for 4-8 h. The reaction mixture was poured into ice-cooled 1N HCl and extracted with ethyl acetate. The combined organic phase was washed with water, brine and dried over Na_2SO_4. The concentrated residue was then purified by flash column chromatography over silica gel to get the desired product.

General procedure C: Mitsunobu reaction [3]

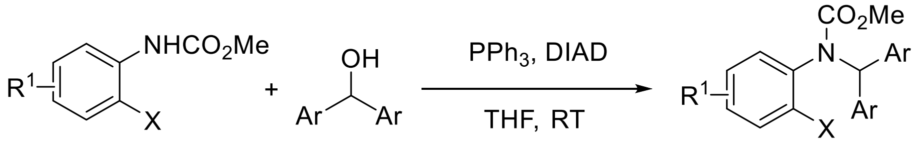

To a solution of benzhydryl alcohol (3 mmol, 1 equiv) in anhydrous THF (10 mL), *N*-aryl-carbamate derivative (3 mmol, 1 equiv) and triphenylphosphine (2 equiv) were added at 0℃ under N_2 atmosphere. Then diisopropylazodicarboxylate (DIAD) (2 equiv) was added within 5 min, the orange-red color of DIAD disappeared immediately with slight heat release. The mixture was stirred at room temperature overnight in N_2 atmosphere. The solvent was evaporated under reduced pressure and the residue was purified by flash column chromatography over silica gel to get the desired product.

Methyl benzhydryl(2-bromo-5-methoxyphenyl) carbamate (1c)

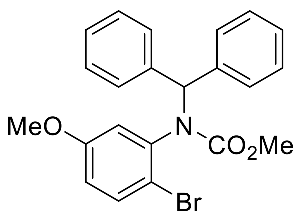

1c

1c was obtained according to the General procedure B, as a white solid (89% yield).
^{1}H NMR (400 MHz, $CDCl_3$) δ 7.43-7.29 (m, 6H), 7.20-7.10 (m, 5H), 6.75-6.73 (m, 2H), 6.61 (dd,

J = 8.8, 2.9 Hz, 1H), 3.76 (s, 3H), 3.61 (s, 3H). ^{13}C NMR (101 MHz, $CDCl_3$) δ 158.71, 155.97, 140.78, 139.73, 137.19, 133.02, 130.85, 128.50, 128.30, 127.78, 127.76, 127.12, 126.55, 116.71, 116.42, 114.47, 66.73, 55.37, 53.37. Melting point: 132.1-135.3 °C. ESI-HRMS calcd. for $C_{22}H_{20}BrNO_3$ $[M+Na]^+$ = 448.0519; found 448.0519. IR (neat): v (cm^{-1}) 2955, 1693, 1441,1309,1030,722,702.

methyl benzhydryl(2-bromo-5-fluorophenyl) carbamate (1d)

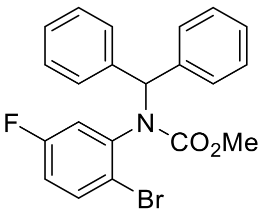

1d was obtained according to the General procedure B, as a white solid (84% yield).

^{1}H NMR (400 MHz, $CDCl_3$) δ 7.40-7.33 (m, 6H), 7.18-7.11 (m, 5H), 7.02-6.99 (m, 1H), 6.80-6.75 (m, 2H), 3.77 (s, 3H). ^{13}C NMR (101 MHz, $CDCl_3$) δ 161.25 (d, J = 246.7 Hz), 155.66, 140.45, 140.41 (d, J = 10.0 Hz), 137.03, 133.54 (d, J = 8.7 Hz), 130.95, 128.48, 127.99, 127.87, 127.49, 127.29, 120.87 (d, J = 3.8 Hz), 118.16 (d, J = 23.1 Hz), 115.88 (d, J = 22.0 Hz), 66.85, 53.47. ^{19}F NMR (376 MHz, $CDCl_3$) δ -113.48. Melting point: 112.3-114.3 °C. ESI-HRMS calcd. for $C_{21}H_{17}BrFNO_2$ $[M+Na]^+$ = 436.0319; found 436.0318. IR (neat): v (cm^{-1}) 2975, 1711, 1438, 1318, 1064, 817, 716, 700.

Methyl benzhydryl(2-bromo-4-methoxyphenyl) carbamate (1f)

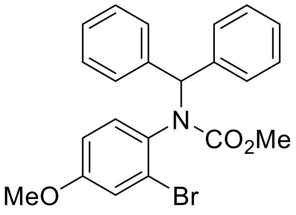

1f

1f was obtained according to the General procedure B, as a white solid (89% yield).

^{1}H NMR (400 MHz, $CDCl_3$) δ 7.42-7.36 (m, 4H), 7.32-7.29 (m, 1H), 7.22-7.07 (m, 6H), 6.94 (d, J = 2.8 Hz, 1H), 6.73 (brs, 1H), 6.67 (dd, J = 8.8 Hz, 2.9 Hz, 1H), 3.75 (s, 3H), 3.73 (s, 3H). ^{13}C NMR (101 MHz, $CDCl_3$) δ 158.79, 156.37, 141.00, 137.34, 131.88, 131.05, 130.72, 128.25, 127.72, 127.69, 127.00, 126.68, 118.08, 113.10, 66.83, 55.54, 53.33. Melting point: 107.5-109.3 °C. ESI-HRMS calcd. for $C_{22}H_{20}BrNO_3$ $[M+Na]^+$ = 448.0519; found 448.0518. IR (neat): v (cm^{-1}) 2960, 1704, 1436,1285, 1030, 743, 699.

Methyl benzhydryl(2-bromo-3-methylphenyl) carbamate (1j)

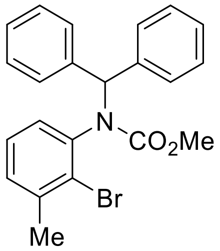

1j was obtained according to the General procedure B, as a white solid (82% yield).

^{1}H NMR (400 MHz, $CDCl_3$) δ 7.43-7.36 (m, 4H), 7.32-7.29 (m, 1H), 7.17-7.01 (m, 8H), 6.71 (s, 1H), 3.74 (s, 3H), 2.29 (s, 3H). ^{13}C NMR (101 MHz, $CDCl_3$) δ 156.13, 141.10, 139.39, 139.32, 137.30, 130.99, 129.57, 128.84, 128.23, 127.86, 127.68, 127.66, 127.51, 126.98, 126.63, 66.98, 53.29, 23.81. Melting point: 144.3-146.7 °C. ESI-HRMS calcd. for $C_{22}H_{20}BrNO_2$ $[M+Na]^+$ = 432.0570; found 432.0569. IR (neat): v (cm^{-1}) 2977, 1698, 1437, 1317, 775, 700.

Methyl benzhydryl(2-bromo-3-methoxyphenyl) carbamate (1k)

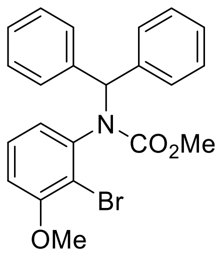

1k was obtained according to the General procedure B, as a white solid (90% yield).

^{1}H NMR (400 MHz, $CDCl_3$) δ 7.43-7.36 (m, 4H), 7.32-7.28 (m, 1H), 7.17-7.09 (m, 6H), 6.89 (dd, *J* = 8.0, 1.1 Hz, 1H), 6.74-6.71 (m, 2H), 3.81 (s, 3H), 3.73 (m, 3H). ^{13}C NMR (101 MHz, $CDCl_3$) δ 156.61, 155.98, 140.99, 140.65, 137.32, 130.85, 128.22, 127.72, 127.65, 127.60, 127.35, 126.98, 122.66, 116.10, 110.80, 66.97, 56.46, 53.30. Melting point: 125.8-127.3 °C. ESI-HRMS calcd. for $C_{22}H_{20}BrNO_3$ $[M+Na]^+$ = 448.0519; found 448.0517. IR (neat): v (cm^{-1}) 2978, 1695, 1438, 1315,1261, 1023, 770, 726, 701.

Methyl benzhydryl(6-bromobenzo[d][1,3]dioxol-5-yl)carbamate (1m)

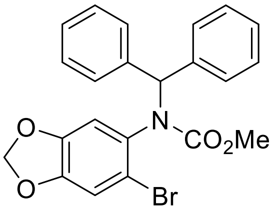

1m was obtained according to the General procedure B, as a white solid (77% yield).

^{1}H NMR (400 MHz, $CDCl_3$) δ 7,40-7.36 (m, 4H), 7.32-7.28 (m, 1H), 7.22-7.16 (m, 3H), 7.13-7.11 (m, 2H), 6.83 (s, 1H), 6.72 (s, 1H), 6.70 (s, 1H), 5.95-5.94 (m, 2H), 3.75 (s, 3H). ^{13}C NMR (101 MHz, $CDCl_3$) δ 156.20, 147.24, 146.91, 140.81, 137.14, 132.38, 131.04, 128.32, 127.82, 127.73, 127.55, 127.08, 117.54, 112.27, 110.65, 102.14, 66.76, 53.41. Melting point: 131.4-133.0 °C. ESI-HRMS calcd. for $C_{22}H_{18}BrNO_4$ $[M+Na]^+$ = 462.0311; found 462.0306. IR (neat): v (cm^{-1}) 2924, 1714, 1477, 1317, 1204, 1028, 772, 747, 701.

Methyl (2-bromophenyl)(di(thiophen-2-yl)methyl)carbamate (1u)

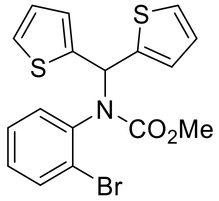

1u was obtained according to the General procedure C, as a pale yellow solid (53% yield).

^{1}H NMR (400 MHz, $CDCl_3$) δ 7.52-7.50 (m, 1H), 7.33-7.32 (m, 1H), 7.28-7.21 (m, 3H), 7.18-7.13 (m, 2H), 7.05-7.04 (m, 1H), 7.00-6.98 (m, 1H), 6.89-6.82 (m, 2H), 3.76 (s, 3H). ^{13}C NMR (101 MHz, $CDCl_3$) δ 155.28, 144.97, 138.76, 133.06, 130.42, 129.30, 128.73, 127.76, 127.15, 126.92,

126.64, 126.16, 125.61, 57.22, 53.48. Melting point: 115.2-116.9 °C. ESI-HRMS calcd. for $C_{17}H_{14}BrNO_2S_2$ $[M+Na]^+$ = 429.9542; found 429.9538. IR (neat): ν (cm^{-1}) 2977, 1697, 1439, 1220, 1050, 772, 749.

Methyl benzhydryl(5-bromoquinoxalin-6-yl)carbamate (1v)

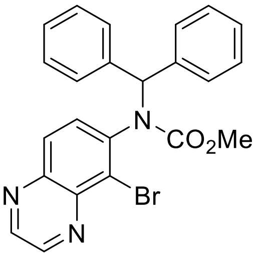

1v was obtained according to the General procedure B, as a white solid (88% yield).

^{1}H NMR (400 MHz, $CDCl_3$) δ 8.90 (d, J = 1.8 Hz, 1H), 8.83 (d, J = 1.8 Hz, 1H), 7.91 (d, J = 8.9 Hz, 1H), 7.67 (d, J = 8.9 Hz, 1H), 7,49-7.33 (m, 5H), 7.16-7.15 (m, 2H), 7.06-7.04 (m, 3H), 6.82 (s, 1H), 3.74 (s, 3H). 13C NMR (101 MHz, CDCl3) δ 155.56, 145.52, 145.50, 142.38, 141.63, 141.00, 140.42, 137.22, 131.92, 130.58, 128.46, 128.39, 127.98, 127.85, 127.53, 127.29, 99.96, 67.20, 53.49. Melting point: 178.2-180.3 °C. ESI-HRMS calcd. for $C_{23}H_{18}BrN_3O_2$ $[M+Na]^+$ = 470.0475; found 470.0471. IR (neat): ν (cm^{-1}) 2953, 1702, 1435, 1307, 1079, 959, 883, 717, 704.

Methyl benzhydryl(3-bromopyridin-4-yl)carbamate (1x)

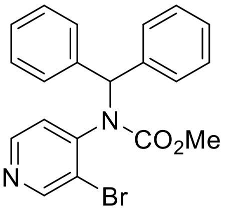

1x was obtained according to the General procedure B, as a white solid (88% yield).

^{1}H NMR (400 MHz, $CDCl_3$) δ 8.56 (s, 1H), 8.31 (d, J = 5.2 Hz, 1H), 7.28-7.27 (m, 10H), 7.12 (d, J = 5.2 Hz, 1H), 6.80 (s, 1H), 3.77 (s, 3H). ^{13}C NMR (101 MHz, $CDCl_3$) δ 154.92, 152.78, 148.70, 146.98, 128.28, 127.83, 124.94, 124.07, 66.64, 53.58. Melting point: 112.8-114.5 °C.ESI-HRMS calcd. for $C_{20}H_{17}BrN_2O_2$ $[M+H]^+$ = 397.0546; found 397.0543. IR (neat): ν (cm^{-1}) 2976, 1727, 1574, 1435, 1320, 1112, 771, 627.

Methyl benzhydryl(4-bromopyridin-3-yl)carbamate (1y)

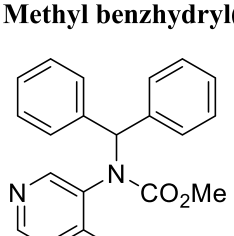

1y was obtained according to the General procedure B, as a white solid (84% yield).

^{1}H NMR (400 MHz, $CDCl_3$) δ 8.43 (s, 1H), 8.15 (d, J = 5.1 Hz, 1H), 7.41-7.32 (m, 6H), 7.19-7.07 (m, 5H), 6.78 (s, 1H), 3.75 (s, 3H). ^{13}C NMR (101 MHz, $CDCl_3$) δ 155.61, 150.94, 148.62, 140.19, 136.94, 136.59, 136.41, 130.90, 128.48, 128.06, 128.02, 127.90, 127.42, 127.35, 66.95, 53.53. Melting point:122.4-124.1 °C. ESI-HRMS calcd. for r $C_{20}H_{17}BrN_2O_2$ $[M+H]^+$ = 397.0546; found 397.0544. IR (neat): ν (cm^{-1}) 2955, 1701, 1439, 1309, 1006, 732, 700 ,659.

Methyl benzhydryl(3-bromopyridin-2-yl)carbamate (1z)

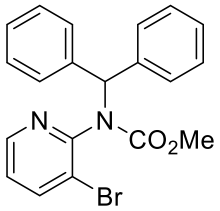

1z was obtained according to the General procedure B, as a white solid (85% yield).
^{1}H NMR (400 MHz, $CDCl_3$) δ 8.38 (dd, J = 4.6, 1.6 Hz, 1H), 7.77-7.68 (m, 3H), 7.42-7.19 (m, 8H), 6.93 (dd, J = 7.9, 4.6 Hz, 1H), 6.70 (s, 1H), 3.71 (s, 3H). ^{13}C NMR (101 MHz, $CDCl_3$) δ 154.88, 151.78, 146.93, 141.50, 128.50, 127.81, 127.19, 126.54, 123.43, 122.56, 67.11, 53.24. Melting point: 149.8-152.1 °C. ESI-HRMS calcd. for $C_{20}H_{17}BrN_2O_2$ $[M+H]^+$ = 397.0546; found 397.0544. IR (neat): ν (cm^{-1}) 2955, 1712, 1434, 1329, 1086, 1020, 699, 626.

Methyl benzhydryl(3-bromo-5-methylpyridin-2-yl)carbamate (1aa)

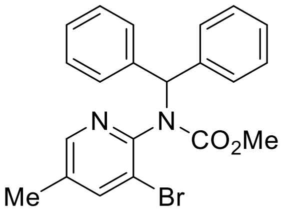

1aa was obtained according to the General procedure B, as a white solid (76% yield).
^{1}H NMR (400 MHz, $CDCl_3$) δ 8.20 (s, 1H), 7.51 (s, 1H), 7.42-7.10 (m, 10H), 6.66 (s, 1H), 3.70 (s, 3H), 2.23 (s, 3H). ^{13}C NMR (101 MHz, $CDCl_3$) δ 155.07, 149.26, 147.24, 143.84, 141.84, 133.72, 130.68, 128.49, 127.95, 127.56, 126.54, 121.86, 67.11, 53.19, 17.48. Melting point: 165.7-167.4 °C. ESI-HRMS calcd. for $C_{21}H_{19}BrN_2O_2$ $[M+H]^+$ = 411.0703; found 411.0696. IR (neat): ν (cm^{-1}) 2952, 1719, 1437 1315, 1084, 758, 704.

Methyl benzhydryl(2,4-dibromophenyl)carbamate (3a)

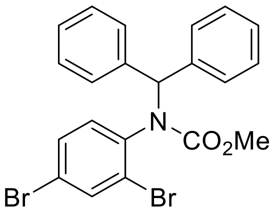

3a was obtained according to the General procedure B, as a white solid (80% yield).
^{1}H NMR (400 MHz, $CDCl_3$) δ 7.56 (s, 1H), 7.39-7.32 (m, 5H), 7.17-7.15 (m, 4H), 7.08-7.06 (m, 3H), 6.74 (s, 1H), 3.74 (s, 1H). ^{13}C NMR (101 MHz, $CDCl_3$) δ 155.71, 140.48, 138.43, 136.94, 135.47, 131.49, 130.86, 130.62, 128.36, 127.99, 127.90, 127.60, 127.20, 127.15, 121.36, 99.98, 66.72, 53.42. Melting point: 119.6-121.3 °C. ESI-HRMS calcd. for $C_{21}H_{17}Br_2NO_2$ $[M+Na]^+$ = 495.9518; found 495.9508. IR (neat): ν (cm^{-1}) 2977, 1706, 1470, 1302, 1219, 772, 740.

Methyl benzhydryl(2,5-dibromophenyl)carbamate (3o)

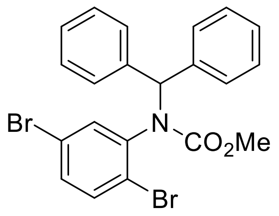

3o was obtained according to the General procedure B, as a white solid (78% yield).
^{1}H NMR (400 MHz, $CDCl_3$) δ 7.41-7.34 (m, 6H), 7.27-7.31 (m, 7H), 6.73 (brs, 1H), 3.76 (s, 3H). ^{13}C NMR (101 MHz, $CDCl_3$) δ 155.59, 140.52, 137.09, 133.94, 133.80, 131.66, 130.91, 128.48, 127.99, 127.87, 127.46, 127.29, 125.17, 120.33, 67.00, 53.48. Melting point: 122.9-124.3 °C. ESI-HRMS calcd. for $C_{21}H_{17}Br_2NO_2$ $[M+Na]^+$ = 495.9518; found 495.9512. IR (neat): ν (cm^{-1}) 2976, 1715, 1438, 1320, 1219, 913, 772, 743, 699.

Methyl benzhydryl(2,6-dibromopyridin-3-yl)carbamate (3p)

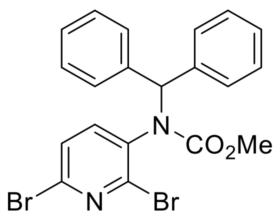

3p was obtained according to the General procedure B, as a white solid (82% yield).
^{1}H NMR (400 MHz, $CDCl_3$) δ 7.42-7.24 (m, 7H), 7.22-7.18 (m, 3H), 7.09-7.07 (m, 2H), 6.77 (s, 1H), 3.76 (s, 3H). ^{13}C NMR (101 MHz, $CDCl_3$) δ 155.21, 144.92, 139.96, 137.89, 136.63, 136.53, 130.69, 128.58, 128.41, 128.28, 127.47, 127.41, 126.75, 66.62, 53.63. Melting point: 183.1-184.9 °C. ESI-HRMS calcd. for $C_{20}H_{16}Br_2N_2O_2$ $[M+Na]^+$ = 496.9471; found 496.9544. IR (neat): ν (cm^{-1}) 2976, 1708, 1427, 1325, 1089, 771, 743, 700.

Methyl benzhydryl(3,5-dibromopyridin-2-yl)carbamate (3q)

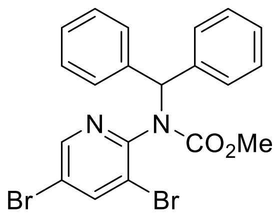

3q was obtained according to the General procedure B, as a white solid (83% yield).
^{1}H NMR (400 MHz, $CDCl_3$) δ 8.38 (s, 1H), 7.81 (s, 1H), 7.58-7.19 (m, 10H), 6.66 (s, 1H), 3.69 (s, 3H). ^{13}C NMR (101 MHz, $CDCl_3$) δ 154.60, 150.70, 147.83, 143.36, 127.90, 127.37, 122.79, 118.47, 67.12, 53.30. Melting point: 162.3-165.4 °C. ESI-HRMS calcd. for $C_{20}H_{16}Br_2N_2O_2$ $[M+Na]^+$ = 496.9471; found 496.9461. IR (neat): ν (cm^{-1}) 2976, 1708, 1504, 1219, 1029, 772.

Methyl benzhydryl(3,5-dibromo-6-methylpyridin-2-yl)carbamate (3r)

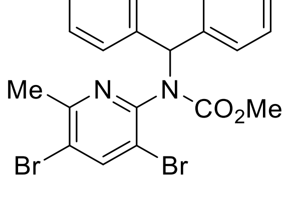

3r was obtained according to the General procedure B, as a white solid (79% yield).
^{1}H NMR (400 MHz, $CDCl_3$) δ 7.77 (s, 1H), 7.65-7.18 (m, 10H), 6.65 (s, 1H), 3.68 (s, 3H), 2.51 (s, 3H). ^{13}C NMR (101 MHz, $CDCl_3$) δ 154.97, 154.65, 149.67, 143.90, 127.79, 127.27, 118.94, 118.85, 67.12, 53.23, 24.01. Melting point: 134.7-136.1 °C. ESI-HRMS calcd. for $C_{21}H_{18}Br_2N_2O_2$ $[M+Na]^+$ = 510.9627; found 510.9621. IR (neat): v (cm^{-1}) 2956, 1721, 1424, 1311, 1043, 770, 751, 699.

Methyl benzhydryl(3,5-dibromo-4-methylpyridin-2-yl)carbamate (3s)

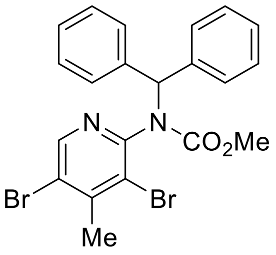

3s was obtained according to the General procedure B, as a white solid (75% yield).
^{1}H NMR (400 MHz, $CDCl_3$) δ 7.82(s, 1H), 7.82-7.58 (m, 2H), 7.46-7.35 (m, 2H), 7.34-7.07 (m, 5H), 6.61 (s, 1H), 3.67 (s, 3H), 2.42 (s, 3H). Melting point: 169.3-172.0 °C. ESI-HRMS calcd. for $C_{21}H_{18}Br_2N_2O_2$ $[M+Na]^+$ = 510.9627; found 510.9617. IR (neat): v (cm^{-1}) 2977, 1717, 1433, 1324, 1094, 769, 750, 701.

Methyl (di-p-tolylmethyl)(2,4-dibromophenyl)carbamate (3t)

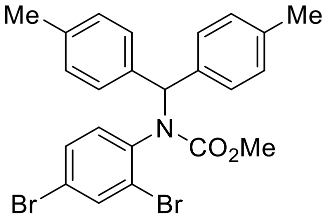

3t was obtained according to the General procedure C, as a white solid (54% yield).
^{1}H NMR (400 MHz, $CDCl_3$) δ 7.54 (s, 1H), 7.23-7.14 (m, 5H), 7.03 (d, J = 8.4 Hz, 1H), 6.95-6.91 (m, 4H), 6.62 (s, 1H), 3.71 (s, 3H), 2.35 (s, 3H), 2.26 (s, 3H). ^{13}C NMR (101 MHz, $CDCl_3$) δ 155.68, 138.64, 137.74, 137.57, 136.71, 135.45, 134.18, 131.49, 130.66, 130.57, 129.02, 128.58, 127.54, 127.16, 121.24, 66.34, 53.33, 21.11, 21.05. Melting point: 117.8-119.3 °C. ESI-HRMS calcd. for $C_{23}H_{21}Br_2NO_2$ $[M+Na]^+$ = 523.9831; found 523.9824. IR (neat): v (cm^{-1}) 2976, 1718, 1439, 1315, 1219, 1011, 771, 745.

Methyl (bis(4-methoxyphenyl)methyl)(2,4-dibromophenyl)carbamate (3u)

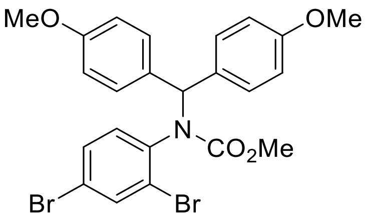

3u was obtained according to the General procedure C, as a white solid (56% yield).
^{1}H NMR (400 MHz, $CDCl_3$) δ 7.58 (d, J = 2.2 Hz, 1H), 7.29-7.25 (m, 3H), 7.03-6.90 (m, 5H), 6.71-6.66 (m, 3H), 3.84 (s, 3H), 3.77 (s, 3H), 3.74 (s, 3H). ^{13}C NMR (101 MHz, $CDCl_3$) δ 159.10, 158.63, 155.70, 138.44, 135.50, 132.80, 131.92, 131.39, 130.62, 129.28, 128.78, 127.25, 121.34,

113.70, 113.22, 65.57, 55.28, 55.16, 53.36. Melting point: 131.0-133.3 °C. ESI-HRMS calcd. for $C_{23}H_{21}Br_2NO_4$ $[M+Na]^+$ = 555.9730; found 555.9725. IR (neat): ν (cm^{-1}) 2951, 1711, 1509, 1469, 1296, 1246, 775, 752.

Methyl (bis(4-fluorophenyl)methyl)(2,4-dibromophenyl)carbamate (3v)

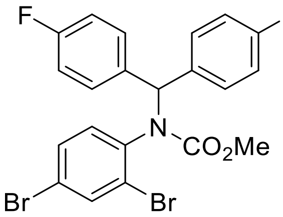

3v was obtained according to the General procedure C, as a white solid (52% yield).
^{1}H NMR (400 MHz, $CDCl_3$) δ 7.60 (d, J = 2.2 Hz, 1H), 7.34-7.28 (m, 3H), 7.10-6.99 (m, 5H), 6.90-6.86 (m, 2H), 6.67 (s, 1H), 3.74 (s, 3H). ^{13}C NMR (101 MHz, $CDCl_3$) δ 162.36 (d, J = 246.4 Hz), 161.99 (d, J = 245.0 Hz), 155.63, 138.13, 136.03 (d, J = 2.3 Hz), 135.71, 132.70 (d, J = 3.2 Hz), 132.37 (d, J = 8.2 Hz), 131.32, 130.83, 129.21 (d, J = 7.9 Hz), 127.06, 121.74, 115.34 (d, J = 21.3 Hz), 115.01 (d, J = 21.4 Hz), 65.40, 53.52. ^{19}F NMR (376 MHz, $CDCl_3$) δ -113.28, -115.24. Melting point: 127.6-129.3 °C. ESI-HRMS calcd. for $C_{21}H_{15}Br_2F_2NO_2$ $[M+Na]^+$ = 531.9330; found 531.9323. IR (neat): ν (cm^{-1})2976, 2951, 1717, 1506, 1470, 1439, 1316, 1219, 7723, 746.

Methyl (di-o-tolylmethyl)(2,4-dibromophenyl)carbamate (3w)

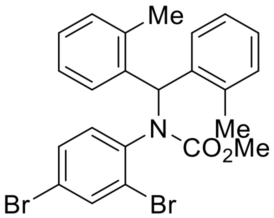

3w was obtained according to the General procedure C, as a white solid (45% yield).
^{1}H NMR (400 MHz, $CDCl_3$) δ 7.60 (s, 1H), 7.53-7.46 (m, 1H), 7.28-7.03 (m, 8H), 6.88 (brs, 1H), 6.73 (brs, 1H), 3.75 (s, 3H), 2.58 (s, 3H), 2.06 (s, 3H). ^{13}C NMR (101 MHz, $CDCl_3$) δ 155.57, 138.27, 135.67, 131.41, 130.91, 130.47, 130.06, 127.70, 126.57, 126.43, 125.92, 125.29, 120.52, 61.52, 53.45, 20.18, 19.83. Melting point: 135.7-137.8 °C. ESI-HRMS calcd. for $C_{23}H_{21}Br_2NO_2$ $[M+Na]^+$ = 523.9831; found 523.9822. IR (neat): ν (cm^{-1}) 2947, 1714, 1436, 1307, 1058, 775, 743.

Methyl (bis(2-fluorophenyl)methyl)(2,4-dibromophenyl)carbamate (3x)

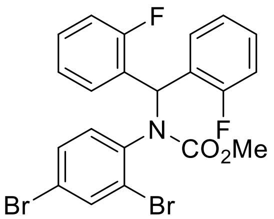

3x was obtained according to the General procedure C, as a white solid (46% yield).
^{1}H NMR (400 MHz, $CDCl_3$) δ 7.63-7.60 (m, 3H), 7.34-7.12 (m, 6H), 7.05-6.91 (m, 4H), 3.70 (s, 3H). ^{13}C NMR (101 MHz, $CDCl_3$) δ 160.46 (d, J = 247.9 Hz), 160.19 (d, J = 245.4 Hz), 155.34, 138.85, 135.56, 131.68, 131.53, 130.71, 129.93 (d, J = 8.4 Hz), 129.31 (d, J = 8.0 Hz), 128.91, 127.17 (d, J = 11.7 Hz), 126.64, 124.44 (d, J = 12.4 Hz), 123.84 (d, J = 2.8 Hz), 123.47 (d, J = 2.8

Hz), 121.52, 115.74 (d, J = 20.7 Hz), 115.53 (d, J = 21.6 Hz), 55.92, 53.44. ^{19}F NMR (376 MHz, $CDCl_3$) δ -112.54, -115.08. Melting point: 115.0-117.8 °C. ESI-HRMS calcd. for $C_{21}H_{15}Br_2F_2NO_2$ $[M+Na]^+$ = 531.9330; found 531.9329. IR (neat): ν (cm^{-1}) 2954, 1701, 1472, 1310, 1222, 1062, 771, 757.

Methyl (bis(3,5-dimethylphenyl)methyl)(2,4-dibromophenyl)carbamate (3y)

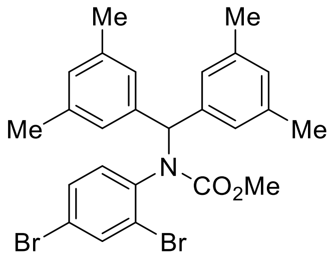

3y was obtained according to the General procedure C, as a white solid (53% yield).

^{1}H NMR (400 MHz, $CDCl_3$) δ 7.55 (s, 1H), 7.28-7.25 (m, 1H), 7.10-7.08 (m, 1H), 6.98-6.95 (m, 3H), 6.82 (s, 1H), 6.65-6.63 (m, 3H), 3.76 (s, 3H), 2.34 (s, 6H), 2.17 (s, 6H). ^{13}C NMR (101 MHz, $CDCl_3$) δ 155.72, 140.53, 138.62, 137.75, 137.08, 136.75, 135.25, 131.54, 130.32, 129.36, 128.83, 128.76, 127.18, 125.37, 121.07, 66.69, 53.36, 21.50, 21.12. Melting point: 123.1-125.5 °C. ESI-HRMS calcd. for $C_{25}H_{25}Br_2NO_2$ $[M+Na]^+$ = 552.0150; found 552.0143. IR (neat): ν (cm^{-1}) 2924, 1702, 1437, 1219, 1051, 771, 743.

Methyl (di(thiophen-2-yl)methyl)(2,4-dibromophenyl)carbamate (3z)

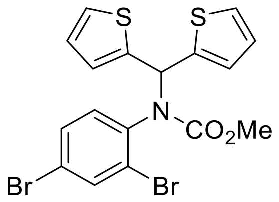

3z was obtained according to the General procedure C, as a white solid (59% yield).

^{1}H NMR (400 MHz, $CDCl_3$) δ 7.66 (d, J = 2.2 Hz, 1H), 7.36-7.24 (m, 4H), 7.05 -7.04(m, 1H), 7.00-6.95 (m, 2H), 6.91 (dd, J = 5.1, 3.6 Hz, 1H), 6.85-6.84 (m, 1H), 3.76 (s, 3H). ^{13}C NMR (101 MHz, $CDCl_3$) δ 155.14, 144.55, 138.34, 137.17, 135.52, 131.41, 130.95, 128.82, 127.92, 127.35, 126.77, 126.34, 125.80, 122.17, 56.98, 53.58. Melting point: 117.0-119.6 °C. ESI-HRMS calcd. for $C_{17}H_{13}Br_2NO_2S_2$ $[M+Na]^+$ = 507.8647; found 507.8640. IR (neat): ν (cm^{-1}) 2976, 1706, 1471, 1306, 1005, 771, 715.

Methyl benzhydryl(2-bromo-4-iodophenyl)carbamate (3aa)

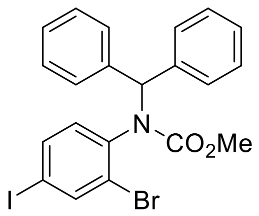

3aa was obtained according to the General procedure B, as a white solid (71% yield).

^{1}H NMR (400 MHz, $CDCl_3$) δ 7.75 (s, 1H), 7.46-7.32 (m, 6H), 7.22-7.06 (m, 5H), 6.91 (d, J = 8.3 Hz, 1H), 6.73 (s, 1H), 3.74 (s, 3H). ^{13}C NMR (101 MHz, $CDCl_3$) δ 155.68, 141.14, 140.50, 139.20, 136.99, 136.67, 131.86, 130.85, 128.37, 128.00, 127.93, 127.66, 127.33, 127.22, 92.66, 66.77, 53.43. Melting point: 120.1-123.3 °C. ESI-HRMS calcd. for $C_{21}H_{17}BrINO_2$ $[M+Na]^+$ = 543.9380;

found 543.9373. IR (neat): ν (cm^{-1}) 2977, 1709, 1435, 1300, 1055, 1005, 772, 738, 690.

Methyl benzhydryl(4-bromo-2-iodophenyl)carbamate (3ab)

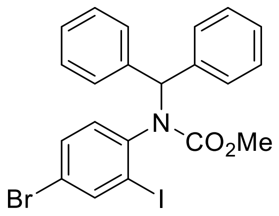

3ab was obtained according to the General procedure B, as a white solid (78% yield).
^{1}H NMR (400 MHz, $CDCl_3$) δ 7.82 (s, 1H), 7.39-7.28 (m, 6H), 7.23-7.16 (m, 3H), 7.09-7.02 (m, 3H), 6.79 (s, 1H), 3.76 (s, 3H). ^{13}C NMR (101 MHz, $CDCl_3$) δ 155.46, 141.60, 141.56, 140.52, 136.61, 131.46, 131.25, 130.71, 128.37, 128.05, 127.91, 127.67, 127.21, 121.34, 104.63, 66.60, 53.45. Melting point: 129.9-132.1 °C. ESI-HRMS calcd. for $C_{21}H_{17}BrINO_2$ $[M+Na]^+$ = 543.9380; found 543.9377. IR (neat): ν (cm^{-1}) 2949, 1716, 1437, 1321, 1051, 773, 732, 703.

Methyl (2-bromophenyl)(phenyl(p-tolyl)methyl)carbamate (5)

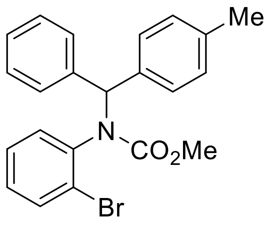

5 was obtained according to the General procedure C, as a viscous oil (45% yield). Two isomers (1:1) were observed by NMR.
^{1}H NMR (400 MHz, $CDCl_3$) δ 7.42-7.36 (m, 3H), 7.31-7.07 (m, 7H), 7.05-7.00 (m, 1H), 6.98-6.92 (m, 2H), 6.71 (s, 1H), 3.75 (s, 3H), 2.32 (d, 3H). ^{13}C NMR (101 MHz, $CDCl_3$) δ 156.01, 141.17, 139.25, 139.20, 137.84, 137.44, 137.39, 136.66, 134.28, 133.03, 133.02, 130.92, 130.84, 130.56, 129.00, 128.65, 128.38, 128.25, 127.69, 127.66, 127.60, 127.58, 127.44, 126.97, 126.25, 66.70, 66.62, 53.34, 21.13, 21.10. ESI-HRMS calcd. for $C_{22}H_{20}BrNO_2$ $[M+Na]^+$ = 432.0570; found 432.0561. IR (neat): ν (cm^{-1}) 2952, 1699, 1439, 1317, 1012, 740, 621.

Methyl (2-bromophenyl)((4-fluorophenyl)(phenyl)methyl)carbamate (6)

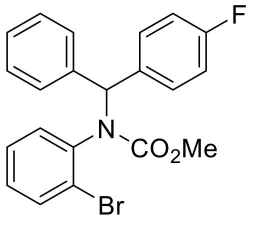

6 was obtained according to the General procedure C, as a viscous oil (43% yield). Two isomers (5:4) were observed by NMR.
^{1}H NMR (400 MHz, $CDCl_3$) δ 7.45-7.32 (m, 5H), 7.26-7.03 (m, 7H), 6.85-6.66 (m, 2H), 3.76 (d, 3H). ^{13}C NMR (101 MHz, $CDCl_3$) δ 163.45, 163.14, 161.00, 160.70, 156.06, 155.99, 140.74, 139.23, 138.85, 137.20, 136.64, 133.21, 133.18, 133.11, 132.81, 132.73, 130.72, 130.58, 130.46, 129.47, 129.39, 128.93, 128.88, 128.63, 128.45, 127.94, 127.88, 127.75, 127.60, 127.49, 127.24, 126.33, 126.14, 115.28, 115.06, 114.75, 114.53, 66.56, 65.92, 53.48, 53.45. The assignment of all peaks in ^{13}C NMR is difficult due to complexity of the spectrum (the rotamers and C-F coupling)

and they are listed as singlets. ^{19}F NMR (376 MHz, $CDCl_3$) δ -114.02, -115.82. ESI-HRMS calcd. for $C_{21}H_{17}BrFNO_2$ [M+Na] $^+$ = 436.0319; found 436.0323. IR (neat): ν (cm^{-1}) 2951, 1702, 1440, 1316, 1013, 773, 727, 698.

Ethyl (2-bromophenyl) ((4-methoxyphenyl) (phenyl) methyl) carbamate (7)

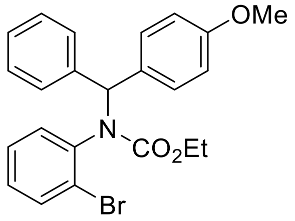

7 was obtained according to the General procedure C, as a viscous oil (48% yield). Two isomers (7:5) were observed by NMR.

^{1}H NMR (400 MHz, $CDCl_3$) δ 7.43-7.31 (m, 5H), 7.26-7.10 (m, 4H), 7.05-6.91 (m, 3H), 6.73-6.64 (m, 2H), 4.32-4.13 (m, 2H), 3.80 (d, 3H), 1.20 (t, *J* = 6 Hz. 3H). ^{13}C NMR (101 MHz, $CDCl_3$) δ 158.95, 158.59, 155.57, 155.52, 133.03, 132.26, 130.75, 130.56, 129.58, 129.04, 128.63, 128.27, 127.72, 127.66, 127.50, 127.45, 127.41, 126.91, 126.35, 126.25, 113.62, 112.99, 66.39, 66.06, 62.17, 55.32, 55.15, 14.63. ESI-HRMS calcd. for $C_{23}H_{22}BrNO_3$ [M+Na] $^+$ = 462.0675; found 462.0679. IR (neat): ν (cm^{-1}) 2836, 1711, 1511, 1322, 1247, 1029, 774, 732.

(3) Optimization Studies

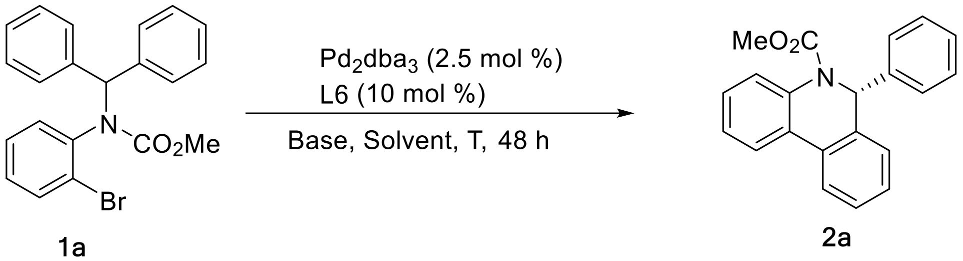

Entry	Temp. (°C)	Solvent	Base	Yield (%) [b]	ee (%) [c]
1	120	DME	Cs_2CO_3	93	86.0
2	100	DME	Cs_2CO_3	93	86.8
3	80	DME	Cs_2CO_3	92	92.2
4	60	DME	Cs_2CO_3	79	93.8
5	80	toluene	Cs_2CO_3	93	92.0
6	80	DMF	Cs_2CO_3	90	87.0
7	80	DME	Na_2CO_3	42	92.2
8	80	DME	K_2CO_3	80	66.0
9	80	DME	K_3PO_4	29	69.0

[a] Reaction conditions: **1a** (0.1 mmol), Pd_2dba_3 (2.5 mol%), **L6** (10 mol%), base (1.5 equiv), 0.05 M in solvent, 48 h. [b] Yield of isolated product. [c] ee values were determined by HPLC analysis using a chiral stationary phase.

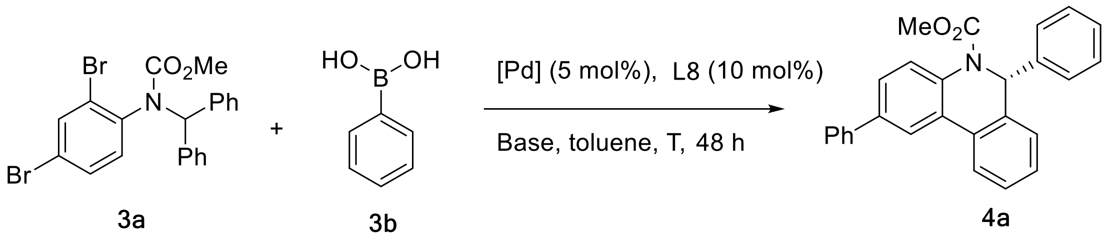

Entry	Temp. (℃)	Base	Pd	Yield (%)[b]	ee (%) [c]
1	120	Cs_2CO_3	Pd_2dba_3	39	65.4
2	100	Cs_2CO_3	Pd_2dba_3	58	89.4
3	80	Cs_2CO_3	Pd_2dba_3	63	94.2
4	60	Cs_2CO_3	Pd_2dba_3	77	97.0
5	50	Cs_2CO_3	Pd_2dba_3	52	97.5
6	40	Cs_2CO_3	Pd_2dba_3	trace	~
7	60	K_3PO_4	Pd_2dba_3	N.D.	~
8	60	K_2CO_3	Pd_2dba_3	N.D.	~
9	60	CSF	Pd_2dba_3	23	94.0
10	60	NaOMe	Pd_2dba_3	14	96.0
11	60	Cs_2CO_3	Pd $(OAc)_2$	62	91.6
12	60	Cs_2CO_3	Pd $(PPh_3)_4$	59	70.8

[a] Reaction conditions: **3a** (0.1 mmol), **3b** (1.1 equiv), Pd (5 mol%), **L8**(10 mol%), base (1.5 equiv), 0.05 M in toluene, 48 h. [b] Yield of isolated product. [c] ee values were determined by HPLC analysis using a chiral stationary phase.

(4) Catalytic asymmetric reaction

General procedure D: the asymmetric C-H arylation

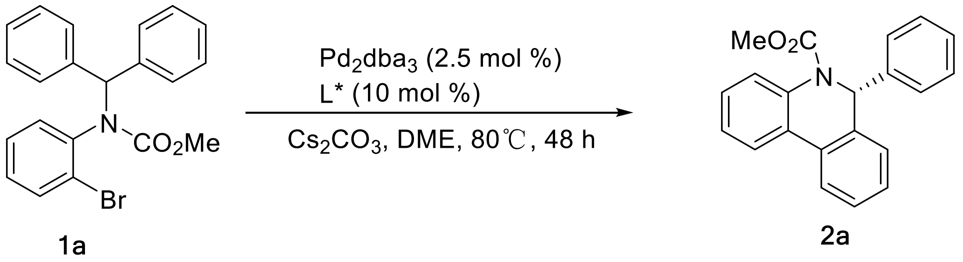

An oven-dried sealing tube was charged with **1a** (0.1 mmol, 1 equiv), pd_2dba_3 (0.025 equiv), chiral ligand (0.1 equiv) and cesium carbonate (1.5 equiv) in glovebox, then dry and degassed DME (2 mL) was added into the tube. The reaction was performed at 80 ℃ for 48 h. After the required time, the reaction was cooled to room temperature and diluted with ethyl acetate. Followed by filtration through a pad of celite and washed with ethyl acetate, the combined filtrate was evaporated under reduced pressure. The concentrated residue was then purified by flash column chromatography over silica gel to get the desired enantio-enriched dihydrophenanthridine **2a**.

General procedure E: the preparation of racemic phenanthridine products

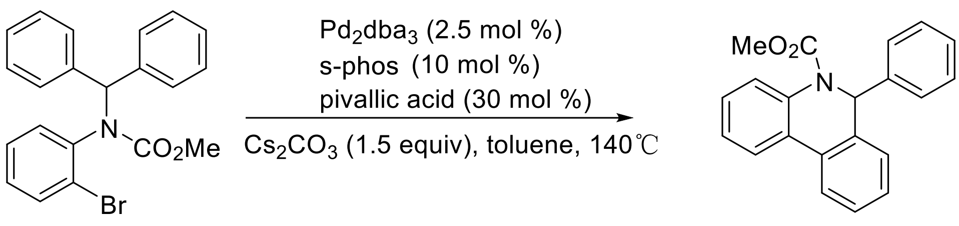

1 2

An oven-dried sealed tube was charged with **1a** (0.2 mmol, 1 equiv), pd_2dba_3 (0.025 equiv), s-phos (0.1 equiv), pivallic acid (0.3 equiv) and cesium carbonate (1.5 equiv) in glove box, then dry and degassed toluene (3 mL) was added into the tube. The reaction was performed at 140 ℃ for 24 h. After the required time, the reaction was cooled to room temperature and diluted with ethyl acetate. Followed by filtration through a pad of celite and washed with ethyl acetate, the filtrate was evaporated under reduced pressure. The concentrated residue was then purified by flash column chromatography over silica gel to get the desired racemic dihydrophenanthridine **2a**.

General procedure F: the Suzuki coupling and asymmetric C-H arylation cascade

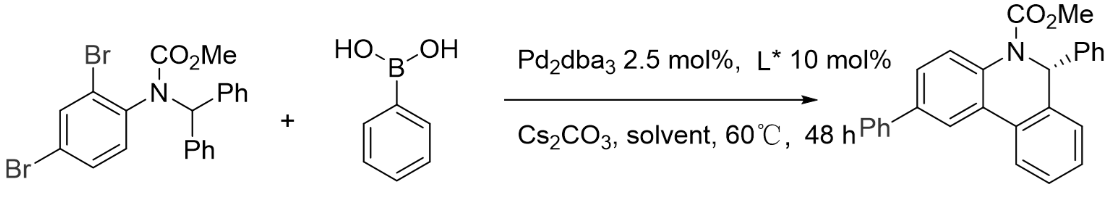

3a 3b 4a

An oven-dried sealed tube was charged with **3a** (0.1 mmol, 1.0 equiv), **3b** (1.1 equiv), pd_2dba_3 (0.025 equiv), chiral ligand (0.1 equiv) and cesium carbonate (3 equiv) in glove box, then dry and degassed toluene (2 mL) or DMF (2 mL) was added into the tube. The reaction was performed at 60℃ for 48 h. After the required time, the reaction was cooled to room temperature and diluted with ethyl acetate. Followed by filtration through a pad of celite and washed with ethyl acetate, the filtrate was evaporated under reduced pressure (when solvent was DMF, the filtrate was washed with water and dried over Na_2SO_4). The concentrated residue was then purified by flash column chromatography over silica gel to get the desired enantioenriched dihydrophenanthridine **4a**.

General procedure G: the preparation of racemic cascade products

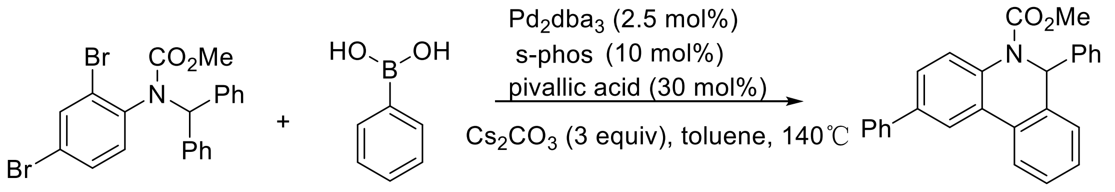

3a 3b 4a

An oven-dried sealed tube was charged with **3a** (0.2 mmol, 1.0 equiv), **3b** (1.1 equiv), pd_2dba_3 (0.025 equiv), s-phos (0.1 equiv), pivallic acid (0.3 equiv) and cesium carbonate (3.0 equiv) in glove box, then dry and degassed toluene (4 mL) was added into the tube. The reaction was performed at 140℃ for 24 h. After the required time, the reaction was cooled to room temperature and diluted with ethyl acetate. Followed by filtration through a pad of celite and washed with ethyl acetate, the filtrate was evaporated under reduced pressure. The concentrated residue was then purified by flash column chromatography over silica gel to get the desired

dihydrophenanthridine **4a**.

General procedure H: the preparation of substrate **3ac**

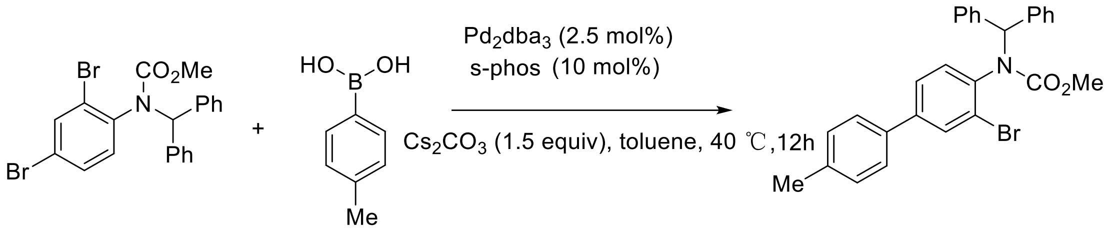

3a 3c 3ac

An oven-dried sealed tube was charged with **3a** (0.2 mmol, 1.0 equiv), **3c** (1.1 equiv), pd_2dba_3 (0.025 equiv), s-phos (0.1 equiv) and cesium carbonate (1.5 equiv) in glove box, then dry and degassed toluene (4 mL) was added into the tube. The reaction was performed at 40 for 12 h. After the required time, the reaction was cooled to room temperature and diluted with ethyl acetate. Followed by filtration through a pad of celite and washed with ethyl acetate, the filtrate was evaporated under reduced pressure. The concentrated residue was then purified by flash column chromatography over silica gel to get the desired substrate **3ac**.

Methyl (*R*)-6-phenylphenanthridine-5(6H)-carboxylate (2a) [2]

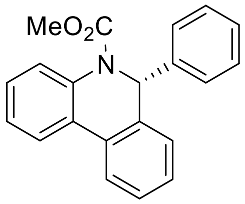

2a was obtained according to the General procedure D, as a viscous oil (99% yield, 96% ee).

^{1}H NMR (400 MHz, $CDCl_3$) δ 7.81 (d, J = 7.8 Hz, 1H), 7.70 (d, J = 7.9 Hz, 1H), 7.43-7.39 (m, 1H), 7.35-7.32 (m, 2H), 7.22-7.01 (m, 8H), 6.72 (brs, 1H), 3.81 (s, 3H).

Methyl (*R*)-3-methyl-6-phenylphenanthridine-5(6H)-carboxylate (2b) [2]

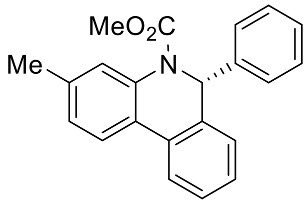

2b was obtained according to the General procedure D, as a viscous oil (99% yield, 94% ee；83% yield, 97% ee).

^{1}H NMR (400 MHz, $CDCl_3$) δ 7.83 (d, J = 7.8 Hz, 1H), 7.64 (d, J = 8.0 Hz, 1H), 7.47-7.43 (m, 1H), 7.38-7/35 (m, 2H), 7.09-7.07 (m, 5H), 6.98 (d, J = 8.0 Hz, 1H), 6.76 (brs, 1H), 3.87 (s, 3H), 2.33 (m, 3H).

Methyl (*R*)-3-methoxy-6-phenylphenanthridine-5(6H)-carboxylate (2c)

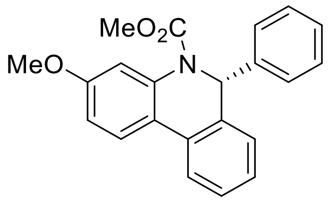

2c was obtained according to the General procedure D, as a white solid (99% yield, 94% ee).

^{1}H NMR (400 MHz, $CDCl_3$) δ 7.79 (d, J = 8 Hz, 1H), 7.68 (d, J = 8 Hz, 1H), 7.45 (t, J = 8 Hz, 1H), 7.37-7.32 (m, 2H), 7.20-7.09 (m, 6H), 6.78-6.75 (m, 2H), 3.89 (s, 3H), 3.81 (s, 3H). ^{13}C NMR (101 MHz, $CDCl_3$) δ 159.36, 154.82, 139.92, 136.02, 134.40, 131.39, 128.37, 128.20, 127.59, 127.32, 127.22, 126.87, 124.49, 123.10, 121.35, 111.37, 58.71, 55.36, 53.32. $[\alpha]_D^{25}$ = -197.2 (c = 1.0 mg/mL in $CHCl_3$). Melting point: 140.1-143.0 °C. ESI-HRMS calcd. for $C_{22}H_{19}NO_3$ $[M+Na]^+$ = 368.1257; found 368.1252. IR (neat): ν (cm^{-1}) 2925, 2852, 1709, 1437, 1221, 1048, 769, 747.

Methyl (*R*)-3-fluoro-6-phenylphenanthridine-5(6H)-carboxylate (2d)

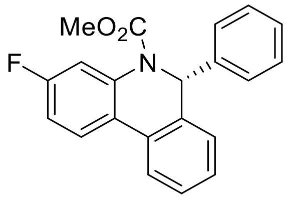

2d was obtained according to the General procedure D, as a white solid (99% yield, 94% ee).

^{1}H NMR (400 MHz, $CDCl_3$) δ 7.81 (d, J = 7.8 Hz, 1H), 7.72 (dd, J = 8.3, 6.1 Hz, 1H), 7.50-7.38 (m, 4H), 7.21-7.17 (m, 3H), 7.08-7.06 (m, 2H), 6.93-6.88 (m, 1H), 6.79 (s, 1H), 3,90 (s, 3H). ^{13}C NMR (101 MHz, $CDCl_3$) δ 162.12 (d, J = 246.7 Hz) 154.69, 139.48, 134.73, 130.71, 128.54, 128.27, 127.70, 127.50, 127.17, 124.91, 124.82, 123.54, 113.00 (d, J = 26.2Hz), 112.42(d, J = 22.0 Hz), 58.56, 53.52. ^{19}F NMR (376 MHz, $CDCl_3$) δ -112.87. $[\alpha]_D^{25}$ = -200.0 (c = 1.0 mg/mL in $CHCl_3$). Melting point: 123.4-124.9 °C. ESI-HRMS calcd. for $C_{21}H_{16}FNO_2$ $[M+Na]^+$ = 356.1057; found 356.1053. IR (neat): ν (cm^{-1}) 2924, 1713, 1437, 1319, 1075, 770, 750.

Methyl (*R*)-2-methyl-6-phenylphenanthridine-5(6H)-carboxylate (2e) [2]

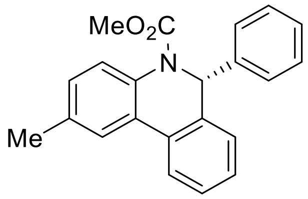

2e was obtained according to the General procedure D, as a white solid (99% yield, 92% ee; 89% yield, 98% ee).

^{1}H NMR (400 MHz, $CDCl_3$) δ 7.86 (d, J = 7.8 Hz, 1H), 7.55 (s, 1H), 7.47-7.37 (m, 4H), 7.16-7.03 (m, 6H), 6.78 (brs, 1H), 3.85 (s, 3H), 2.35 (s, 3H).

Methyl (*R*)-2-methoxy-6-phenylphenanthridine-5(6H)-carboxylate (2f)

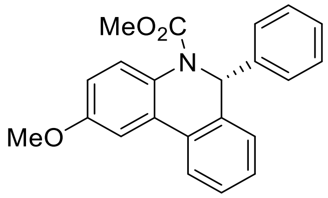

2f was obtained according to the General procedure D, as a white solid (99% yield, 93% ee).

^{1}H NMR (400 MHz, $CDCl_3$) δ 7.85 (d, *J* = 8 Hz, 1H), 7.50-7.27 (m, 5H), 7.20-7.08(m, 5H), 6.83-6.81 (m, 2H), 3.85 (s, 6H). ^{13}C NMR (101 MHz, CDCl3) δ 156.88, 139.72, 135.79, 131.32, 129.26, 128.37, 128.16, 127.93, 127.77, 127.30, 123.88, 113.77, 108.48, 58.50, 55.44, 53.26. $[\alpha]_D^{25}$ = -177.7 (*c* = 1.0 mg/mL in $CHCl_3$). Melting point: 124.2-125.5 ºC. ESI-HRMS calcd. for $C_{22}H_{19}NO_3$ $[M+Na]^+$ = 368.1257; found 368.1250.

Methyl (*R*)-2-fluoro-6-phenylphenanthridine-5(6H)-carboxylate (2g) [2]

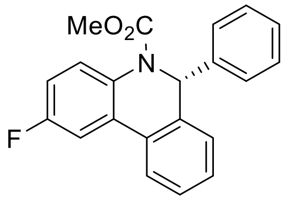

2g was obtained according to the General procedure D, as a white solid (99% yield, 94% ee; 92% yield, 97% ee).

^{1}H NMR (400 MHz, $CDCl_3$) δ 7.80-7.78 (m, 1H), 7.50-7.38 (m, 5H), 7.17-7.15 (m, 3H), 7.06-7.04 (m, 2H), 6.95-6.79 (m, 2H), 3.86 (s, 3H).

Methyl (*R*)-2-chloro-6-phenylphenanthridine-5(6H)-carboxylate (2h) [2]

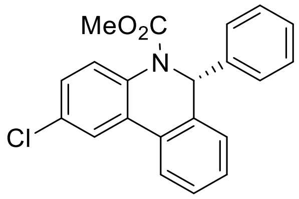

2h was obtained according to the General procedure D, as a white solid (99% yield, 95% ee).

^{1}H NMR (400 MHz, $CDCl_3$) δ 7.82 (d, *J* = 7.6 Hz, 1H), 7.72 (d, *J* = 2.4 Hz, 1H), 7.50-7.38 (m, 4H), 7.20-7.13 (m, 4H), 7.09-7.04 (m, 2H), 6.78 (brs, 1H),3.87 (s, 3H).

Dimethyl (*R*)-6-phenylphenanthridine-2,5(6H)-dicarboxylate (2i) [2]

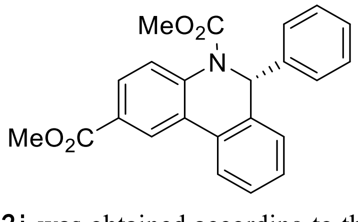

2i was obtained according to the General procedure D, as a white solid (99% yield, 96% ee; 83% yield, 99% ee).

^{1}H NMR (400 MHz, $CDCl_3$) δ 8.47 (d, *J* = 2.0 Hz, 1H), 7.97-7.88 (m, 2H), 7.62 (s, 1H), 7.49 (td, *J* = 7.4 Hz, 1.1 Hz, 1H), 7.43-7.37 (m, 2H), 7.16-7.12 (m, 3H), 7.05-7.03 (m, 2H), 6.79 (s, 1H), 3.92 (s, 3H)， 3.89 (s, 3H).

Methyl (*R*)-1-methyl-6-phenylphenanthridine-5(6H)-carboxylate (2j)

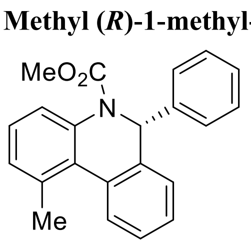

2j was obtained according to the General procedure D, as a white solid (99% yield, 82% ee).

^{1}H NMR (400 MHz, $CDCl_3$) δ 7.89 (d, *J* = 8 Hz, 1H), 7.49-7.39 (m, 4H), 7.18-7.10 (m, 6H), 7.02

(d, J = 8 Hz, 1H), 6.74 (brs, 1H), 3.86 (s, 3H), 2.64 (s, 3H). ^{13}C NMR (101 MHz, $CDCl_3$) δ 155.15, 138.99, 137.75, 136.04, 134.55, 131.69, 128.70, 128.36, 128.18, 128.02, 127.54, 127.28, 127.16, 127.03, 124.10, 58.85, 53.26, 22.98. $[\alpha]_D^{25}$ = -78.5 (c = 1.0 mg/mL in $CHCl_3$). Melting point: 155.9-157.6 °C. ESI-HRMS calcd. for $C_{22}H_{19}NO_2$ $[M+Na]^+$ = 352.1308; found 352.1302. IR (neat): ν (cm^{-1}) 2921, 1699, 1440, 1321, 1079, 769, 746, 734, 687.

Methyl (*R*)-1-methoxy-6-phenylphenanthridine-5(6H)-carboxylate (2k)

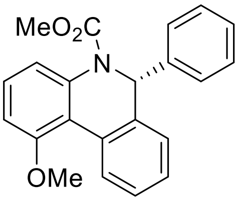

2k was obtained according to the General procedure D, as a white solid (99% yield, 96% ee).

^{1}H NMR (400 MHz, $CDCl_3$) δ 8.49 (d, J = 8 Hz, 1H), 7.47-7.35 (m, 3H), 7.19-7.11 (m, 7H), 6.75-6.73 (m, 2H), 3.89 (s, 3H), 3.86 (s, 3H). ^{13}C NMR (101 MHz, CDCl3) δ 156.73, 139.25, 136.63, 136.16, 129.69, 128.57, 128.03, 127.82, 127.56, 127.26, 127.22, 127.14, 127.07, 118.90, 117.97, 108.01, 58.69, 55.62, 53.27. $[\alpha]_D^{25}$ = -129.6 (c = 1.0 mg/mL in $CHCl_3$). Melting point: 145.0-146.8 °C. ESI-HRMS calcd. for $C_{22}H_{19}NO_3$ $[M+Na]^+$ = 368.1257; found 368.1252. IR (neat): ν (cm^{-1}) 2925, 1692, 1435, 1252, 1132, 773, 745.

Methyl (*R*)-2,4-dimethyl-6-phenylphenanthridine-5(6H)-carboxylate (2l) [2]

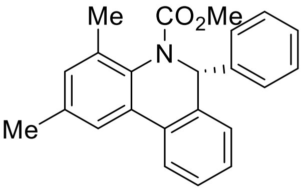

2l was obtained according to the General procedure D, as a white solid (99% yield, 92% ee; 86% yield, 97% ee).

^{1}H NMR (400 MHz, $CDCl_3$) δ 7.88 (d, J = 7.6 Hz, 1H), 7.49-7.42 (m, 1H), 7.40-7.36 (m, 3H), 7.16-7.11 (m, 3H), 7.04-7.00 (m, 2H), 6.87 (s, 1H), 6.78 (brs, 1H), 3.74 (s, 3H), 2.31 (s, 3H), 2.13 (s, 3H).

Methyl (*R*)-5-phenyl-[1,3]dioxolo[4,5-b]phenanthridine-6(5H)-carboxylate (2m)

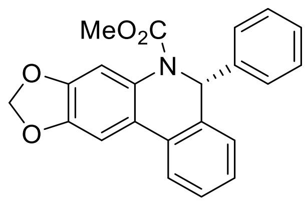

2m was obtained according to the General procedure D, as a viscous oil (97% yield, 94% ee).

^{1}H NMR (400 MHz, $CDCl_3$) δ 7.70 (d, J = 7.8 Hz, 1H), 7.47-7.43 (m, 1H), 7.37-7.33 (m, 2H), 7.21-7.16 (m, 4H), 7.09-7.07 (m, 2H), 6.89-6.79 (m, 1H), 5.97-5.93 (m, 2H), 3.86 (s, 3H). ^{13}C NMR (101 MHz, $CDCl_3$) δ 155.12, 147.18, 145.43, 139.58, 131.56, 128.42, 128.16, 127.58, 127.34, 127.25, 127.15, 123.34, 122.36, 107.22, 103.04, 101.38, 58.45, 53.37. $[\alpha]_D^{25}$ = -151.8 (c = 1.0 mg/mL in $CHCl_3$). ESI-HRMS calcd. for $C_{22}H_{17}NO_4$ $[M+Na]^+$ = 382.1050; found 382.1044. IR (neat): ν (cm^{-1}) 2926, 1702, 1494, 1329, 1218, 935, 771, 747.

Methyl (*R*)-9-methyl-6-(p-tolyl)phenanthridine-5(6H)-carboxylate (2n) [2]

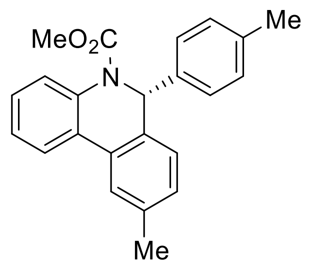

2n was obtained according to the General procedure D, as a viscous oil (99% yield, 94% ee; 84% yield, 97% ee).
^{1}H NMR (400 MHz, $CDCl_3$) δ 7.71 (d, J = 7.8 Hz, 1H), 7.64 (s, 1H), 7.23-7.09 (m, 5H), 6.94-6.89 (m, 4H), 6.68 (brs, 1H), 3.81 (s, 3H), 2.43 (s, 3H), 2.17 (s, 3H).

Methyl (*R*)-9-methoxy-6-(4-methoxyphenyl)phenanthridine-5(6H)-carboxylate (2o) [2]

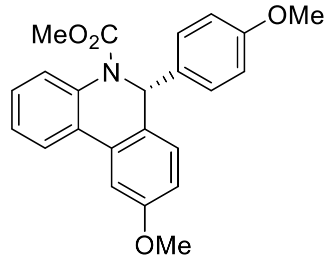

2o was obtained according to the General procedure D, as a white solid (98% yield, 95% ee; 85% yield, 97% ee).
^{1}H NMR (400 MHz, $CDCl_3$) δ 7.69 (d, J = 7.7 Hz, 1H), 7.54-7.41 (m, 1H), 7.34 (d, J = 2.5 Hz, 1H), 7.21-7.13 (m, 3H), 6.94-6.91 (m, 2H), 6.90-6.88 (m, 1H), 6.64-6.61 (m, 3H), 3.88 (s, 3H), 3.82 (s, 3H), 3.65 (s, 3H).

Methyl (*R*)-9-fluoro-6-(4-fluorophenyl)phenanthridine-5(6H)-carboxylate (2p) [2]

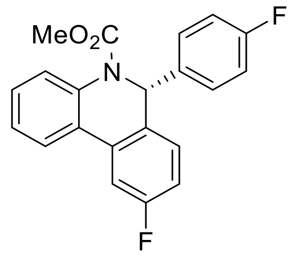

2p was obtained according to the General procedure D, as a white solid (99% yield, 97% ee).
^{1}H NMR (400 MHz, $CDCl_3$) δ 7.66 (d, J = 7.8 Hz, 1H), 7.54-7.45 (m, 2H), 7.32-7.23 (m 2H), 7.17 (t, J = 7.6 Hz, 1H), 7.06 (td, J = 8.3, 1.7 Hz, 1H), 7.00-6.97 (m, 2H), 6.80 (t, J = 8.5 Hz, 1H), 6.73 (brs, 1H), 3.85 (s, 3H).

Methyl (*R*)-7-methyl-6-(o-tolyl)phenanthridine-5(6H)-carboxylate (2q) [2]

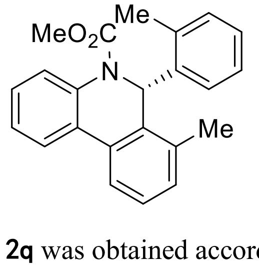

2q was obtained according to the General procedure D, as a white solid (85% yield, 93% ee).

^{1}H NMR (400 MHz, $CDCl_3$) δ 7.79-7.74 (m, 2H), 7.36 (t, J = 7.7 Hz, 1H), 7.23-7.14 (m, 5H), 7.08 (s, 1H), 7.02 (t, J = 7.4 Hz, 1H), 6.72 (t, J = 7.5 Hz, 1H), 6.30 (d, J = 7.8 Hz, 1H), 3.76 (s, 3H), 2.67 (s, 3H), 2.25 (s, 3H).

Methyl (*R*)-7-fluoro-6-(2-fluorophenyl)phenanthridine-5(6H)-carboxylate (2r) [2]

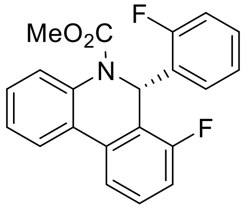

2r was obtained according to the General procedure D, as a white solid (96% yield, 88% ee; 83% yield, 97% ee).

^{1}H NMR (400 MHz, $CDCl_3$) δ 7.79 (d, J = 7.6 Hz, 1H), 7.68 (d, J = 7.8 Hz, 1H), 7.49-7.41 (m, 3H), 7.28-7.20 (m, 2H), 7.17-6.99 (m, 3H), 6.75 (t, J = 7.5 Hz, 1H), 6.50 (t, J = 7.6 Hz, 1H), 3.85 (s, 3H).

Methyl (*R*)-6-(3,5-dimethylphenyl)-8,10-dimethylphenanthridine-5(6H)-carboxylate (2s) [2]

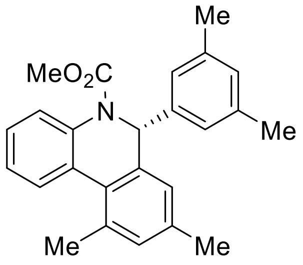

2s was obtained according to the General procedure D, as a white solid (99% yield, 92% ee; 86% yield, 98% ee).

^{1}H NMR (400 MHz, $CDCl_3$) δ 7.69 (d, J = 7.8 Hz, 1H), 7.35 (s, 1H), 7.18-7.04 (m, 4H), 6.74-6.47 (m, 4H), 3.84 (s, 3H), 2.67 (s, 3H), 2.39 (s, 3H), 2.15 (s, 3H) .^{13}C NMR (101 MHz, $CDCl_3$) δ 154.95, 139.32, 138.64, 137.52, 137.00, 135.87, 134.89, 132.63, 129.49, 129.05, 128.37, 127.59, 127.02, 126.41, 125.34, 124.39, 59.45, 53.25, 23.00, 21.34, 21.11.

Methyl (*R*)-6-(3,5-dimethoxyphenyl)-8,10-dimethoxyphenanthridine-5(6H)-carboxylate (2t)

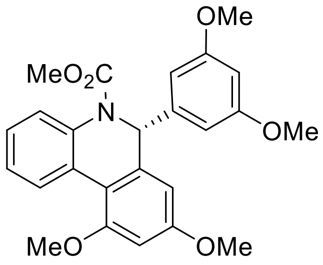

2t was obtained according to the General procedure D, as a viscous oil (99% yield, 91% ee).

^{1}H NMR (400 MHz, $CDCl_3$) δ 8.27 (d, J = 8.8 Hz, 1H), 7.34 (s, 1H), 7.15-7.10 (m, 2H), 6.58-6.55 (m, 3H), 6.27 (s, 2H), 6.23 (s, 1H), 3.93 (s, 3H), 3.87 (s, 3H), 3.83 (s, 3H), 3.65 (s, 6H). 13C NMR (101 MHz, CDCl3) δ 160.46, 159.91, 158.40, 141.60, 139.39, 134.26, 127.56, 127.10, 126.29, 124.61, 113.63, 105.87, 104.17, 99.09, 98.93, 59.20, 55.63, 55.43, 55.17, 53.18. $[\alpha]_D^{25}$ = -137.1 (c = 1.0 mg/mL in $CHCl_3$). ESI-HRMS calcd. for $C_{22}H_{17}NO_4$ $[M+Na]^+$ = 458.1574; found 458.1582. IR (neat): ν (cm^{-1}) 2924, 1689, 1596, 1460, 1322, 1149, 1025, 770, 747.

Methyl (*R*)-4-(thiophen-2-yl)thieno[2,3-c]quinoline-5(4H)-carboxylate (2u)

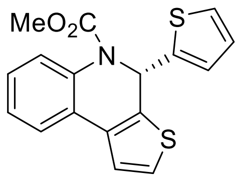

2u was obtained according to the General procedure D (50 ℃), as a viscous oil (88% yield, 92% ee).

^{1}H NMR (400 MHz, $CDCl_3$) δ 7.60-7.58 (m, 2H), 7.42-7.35 (m ,2H), 7.26-7.12 (m, 4H), 6.82-6.79 (dd, *J* = 4.8, 3.6 Hz, 1H), 6.73-6.72 (m, 1H), 3.90 (s, 3H). ^{13}C NMR (101 MHz, $CDCl_3$) δ 154.65, 143.22, 133.57, 132.48, 127.02, 126.50, 125.88, 125.64, 125.54, 125.20, 125.06, 123.28, 122.54, 53.52, 51.77. $[\alpha]_D^{25}$ = -197.2 (*c* = 1.0 mg/mL in $CHCl_3$). ESI-HRMS calcd. for $C_{17}H_{13}NO_2S_2$ $[M+Na]^+$ = 350.0280; found 350.0273. IR (neat): ν (cm^{-1}) 2924, 1701, 1376, 1220, 1031, 913, 772, 719.

Methyl (*R*)-8-phenylpyrazino[2,3-a]phenanthridine-7(8H)-carboxylate (2v)

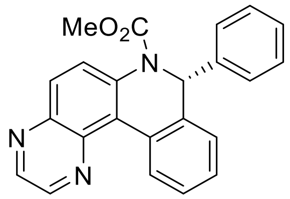

2v was obtained according to the General procedure D (60 ℃), as a pale yellow solid (91% yield, 99% ee).

^{1}H NMR (400 MHz, $CDCl_3$) δ 8.96 (d, *J* = 7.9 Hz, 1H), 8.90 (d, *J* = 1.6 Hz, 1H), 8.81 (d, *J* = 1.6 Hz, 1H), 8.02-7.94 (m, 2H), 7,62-7.56 (m, 1H), 7.53-7.49 (m, 2H), 7.15-7.06 (m, 5H), 6.83 (s, 1H), 3.95 (s, 3H). ^{13}C NMR (101 MHz, $CDCl_3$) δ 154.66, 143.95, 143.66, 141.17, 140.51, 138.70, 136.85, 131.00, 129.51, 129.22, 128.61, 128.21, 128.13, 127.90, 127.41, 127.27, 127.18, 124.84, 58.77, 53.69. $[\alpha]_D^{25}$ = -175.7 (*c* = 1.0 mg/mL in $CHCl_3$). Melting point: 189.0-190.3 ^{o}C. ESI-HRMS calcd. for $C_{23}H_{17}N_3O_2$ $[M+H]^+$ = 368.1394; found 368.1387. IR (neat): ν (cm^{-1}) 2957, 2926, 1693, 1318, 1243, 1028, 772, 742, 700.

Methyl (*R*)-6-phenylbenzo[c][1,5]naphthyridine-5(6H)-carboxylate (2w) [2]

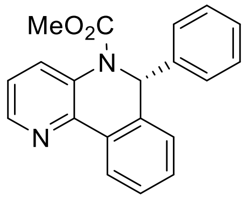

2w was obtained according to the General procedure D, as a white solid (96% yield, 96% ee).

^{1}H NMR (400 MHz, $CDCl_3$) δ 8.42-8.39 (m, 2H), 7.84 (s, 1H), 7.54-7.43 (m, 2H), 7.33 (d, *J* = 7.1 Hz, 1H), 7.17-7.14 (m, 4H), 7.06-7.03 (m, 2H), 6.79 (brs, 1H), 3.89 (s, 3H). [2]

Methyl (*R*)-6-phenylbenzo[c][1,6]naphthyridine-5(6H)-carboxylate (2x)

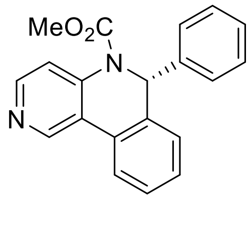

2x was obtained according to the General procedure D as a pale yellow solid (91% yield, 97% ee). ^{1}H NMR (400 MHz, $CDCl_3$) δ 9.04 (s, 1H), 8.43 (d, *J* = 4.9 Hz, 1H), 7.95 (d, *J* = 7.7 Hz, 1H), 7.59-7.58 (m, 1H), 7.54-7.38 (m, 3H), 7.19-7.17 (m, 3H), 7.06-7.04 (m, 2H), 6.78 (s, 1H), 3.95 (s, 3H). ^{13}C NMR (101 MHz, $CDCl_3$) δ 154.23, 148.79, 145.22, 142.29, 139.32, 135.09, 128.80, 128.74, 128.45, 127.84, 127.80, 127.06, 123.26, 118.96, 58.65, 53.77. $[\alpha]_D^{25}$ = -167.9 (*c* = 1.0 mg/mL in $CHCl_3$). Melting point: 139.0-141.3 °C. ESI-HRMS calcd. for $C_{20}H_{16}N_2O_2$ $[M+H]^+$ = 317.1285; found 317.1281. IR (neat): ν (cm^{-1}) 2952, 2925, 1705, 1446, 1269, 1220, 773, 752.

Methyl (*R*)-6-phenylbenzo[c][1,7]naphthyridine-5(6H)-carboxylate (2y)

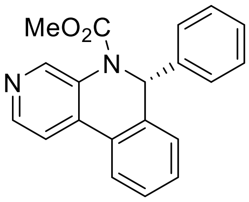

2y was obtained according to the General procedure D as a pale yellow solid (91% yield, 97% ee). ^{1}H NMR (400 MHz, $CDCl_3$) δ 8.76 (s, 1H), 8.39 (d, *J* = 5.2 Hz, 1H), 7.94-7.92 (m, 1H), 7.64 (d, *J* = 5.2 Hz, 1H), 7.54-7.49 (m, 2H), 7.44-7.41 (m, 1H), 7.20-7.16 (m, 3H), 7.05-7.03 (m, 2H), 6.84 (s, 1H), 3.91 (s, 3H). ^{13}C NMR (101 MHz, $CDCl_3$) δ 154.61, 147.53, 145.50, 139.06, 136.47, 134.47, 131.10, 129.87, 128.78, 128.68, 128.37, 128.04, 127.71, 127.17, 124.31, 117.00, 58.19, 53.64. $[\alpha]_D^{25}$ = -135.6 (*c* = 1.0 mg/mL in $CHCl_3$). Melting point: 145.9-147.3 °C. ESI-HRMS calcd. for $C_{20}H_{16}N_2O_2$ $[M+H]^+$ = 317.1285; found 317.1279. IR (neat): ν (cm^{-1}) 2922, 1700, 1380, 1259, 1076, 770, 744, 695.

Methyl (*R*)-6-phenylbenzo[c][1,8]naphthyridine-5(6H)-carboxylate (2z)

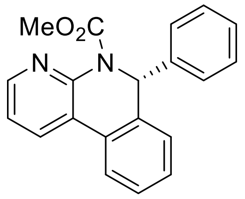

2z was obtained according to the General procedure D as a pale yellow solid (99% yield, 95% ee). ^{1}H NMR (400 MHz, $CDCl_3$) δ 8.40 (dd, *J* = 4.8, 1.8 Hz, 1H), 8.04 (dd, *J* = 7.8, 1.7 Hz, 1H), 7.83 (d, *J* = 7.2 Hz, 1H), 7.52-7.46 (m, 3H), 7.18-7.11 (m, 6H), 6.83 (s, 1H), 3.91 (s, 3H). ^{13}C NMR (101 MHz, $CDCl_3$) δ 154.96, 148.43, 147.98, 139.36, 135.68, 131.83, 129.96, 128.58, 128.52, 128.22, 127.87, 127.37, 127.10, 123.95, 123.32, 120.84, 59.26, 53.67. $[\alpha]_D^{25}$ = -169.9 (*c* = 1.0 mg/mL in $CHCl_3$). Melting point: 167.7-169.2 °C. ESI-HRMS calcd. for $C_{20}H_{16}N_2O_2$ $[M+H]^+$ = 317.1285; found 317.1282. IR (neat): ν (cm^{-1}) 2925, 1694, 1421, 1219, 913, 770, 756, 708.

Methyl (*R*)-2-methyl-6-phenylbenzo[c][1,8]naphthyridine-5(6H)-carboxylate (2aa)

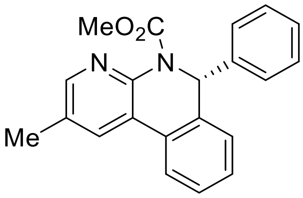

2aa was obtained according to the General procedure D as a pale yellow solid (99% yield, 95% ee).

^{1}H NMR (400 MHz, $CDCl_3$) δ 8.22 (d, *J* = 1.6 Hz, 1H), 7.84-7.82 (m, 2H), 7.51-7.43 (m, 3H), 7.19-7.10 (m, 5H), 6.82 (s, 1H), 3.90 (s, 3H), 2.34 (s, 3H). ^{13}C NMR (101 MHz, $CDCl_3$) δ 155.06,

148.32, 146.11, 139.45, 135.72, 132.36, 130.36, 130.05, 128.44, 128.19, 127.90, 127.31, 127.11, 123.91, 122.73, 59.24, 53.61, 18.08. $[\alpha]_D^{25}$ = -167.6 (*c* = 1.0 mg/mL in $CHCl_3$). Melting point: 179.2-180.9 °C. ESI-HRMS calcd. for $C_{21}H_{18}N_2O_2$ $[M+H]^+$ = 331.1441; found 331.1439. IR (neat): ν (cm^{-1}) 2922, 1696, 1433, 1243, 1045, 773, 718.

(*R*)-6-phenyl-5-tosyl-5,6-dihydrophenanthridine (2ab) [2]

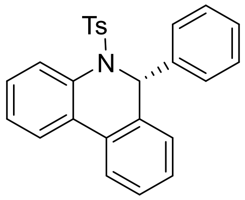

2ab was obtained according to the General procedure D, as a white solid (88% yield, 95% ee).
^{1}H NMR (400 MHz, $CDCl_3$) δ 7.63 (d, *J* = 7.6 Hz, 1H), 7.45 (d, *J* = 7.5 Hz, 1H), 7,26 (d, *J* = 7.4 Hz, 1H), 7.20-7.10 (m, 4H), 7.08-7.02 (m, 6H), 6.92 (d, *J* = 8.1 Hz,2H), 6.65 (d, *J* = 8.0 Hz, 2H), 6.41 (s, 1H), 2.09 (s, 3H).

Methyl (*R*)-2,6-diphenylphenanthridine-5(6H)-carboxylate (4a)

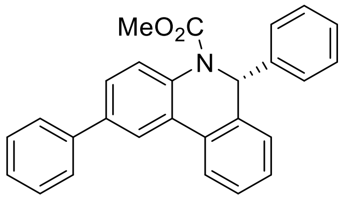

4a was obtained according to the General procedure F as a white solid (77% yield, 97% ee)
^{1}H NMR (400 MHz, $CDCl_3$) δ 8.00-7.96 (m, 2H), 7.65-7.38 (m, 10H), 7.20-7.13 (m, 5H), 6.83 (brs, 1H), 3.92 (s, 3H). ^{13}C NMR (101 MHz, $CDCl_3$) δ 155.13, 140.61, 139.77, 137.94, 135.53, 134.11, 131.28, 128.78, 128.45, 128.35, 128.24, 127.90, 127.77, 127.39, 127.29, 126.98, 126.80, 123.84, 122.22, 58.63, 53.39. $[\alpha]_D^{25}$ = -171.9 (*c* = 1.0 mg/mL in $CHCl_3$). Melting point: 120.0-124.0 °C. ESI-HRMS calcd. for $C_{27}H_{21}NO_2$ $[M+Na]^+$ = 414.1465; found 414.1455. IR (neat): ν (cm^{-1}) 2921, 1700, 1447, 1321, 1254, 769, 743, 698.

Methyl (*R*)-6-phenyl-2-(p-tolyl)phenanthridine-5(6H)-carboxylate (4b)

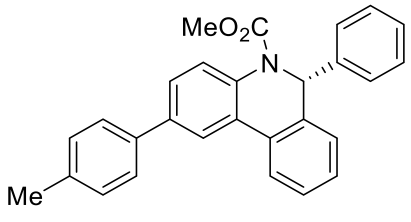

4b was obtained according to the General procedure F as a pale yellow solid (76% yield, 95% ee).
^{1}H NMR (400 MHz, $CDCl_3$) δ 7.97-7.95 (m, 2H), 7.55-7.41 (m, 7H), 7.29-7.27 (m, 2H), 7.22-7.12 (m, 5H), 6.82 (brs, 1H), 3.91 (s, 3H), 2.43 (s, 3H). ^{13}C NMR (101 MHz, $CDCl_3$) δ 153.89, 139.79, 137.88, 137.72, 135.51, 131.34, 129.50, 128.43, 128.29, 128.23, 127.85, 127.77, 127.37, 127.26, 126.81, 126.62, 126.15, 123.83, 121.99, 58.61, 53.39, 21.12. $[\alpha]_D^{25}$ = -128.8 (*c* = 1.0 mg/mL in $CHCl_3$). Melting point: 124.6-126.6 °C. ESI-HRMS calcd. for $C_{28}H_{23}NO_2$ $[M+Na]^+$ = 428.1621; found 428.1610. IR (neat): ν (cm^{-1}) 2912, 1709, 1491, 1219, 913, 772, 742.

Methyl (*R*)-2-(4-methoxyphenyl)-6-phenylphenanthridine-5(6H)-carboxylate (4c)

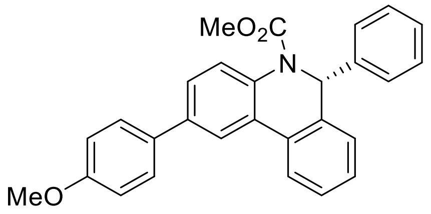

4c was obtained according to the General procedure F as a pale yellow solid (73% yield, 98% ee). ^{1}H NMR (400 MHz, $CDCl_3$) δ 7.97-7.94 (m, 2H), 7.58-7.56 (m, 2H), 7.51-7.41 (m, 5H), 7.20-7.13 (m, 5H), 7.02-7.00 (m, 2H), 6.83 (brs, 1H), 3.91 (s, 3H), 3.88 (s, 3H). ^{13}C NMR (101 MHz, $CDCl_3$) δ 159.17, 139.80, 137.59, 135.56, 133.17, 131.35, 130.95, 130.45, 128.43, 128.29, 128.23, 128.06, 128.01, 127.85, 127.77, 127.37, 127.26, 127.09, 126.42, 125.47, 123.83, 121.74, 114.30, 114.23, 58.60, 55.37, 53.38. $[\alpha]_D^{25}$ = -170.4 (*c* = 1.0 mg/mL in $CHCl_3$). Melting point: 134.1-135.8 °C. ESI-HRMS calcd. for $C_{28}H_{23}NO_3$ $[M+Na]^+$ = 444.1570; found 444.1560. IR (neat): ν (cm^{-1}) 2954, 1693, 1490, 1244, 1244, 913, 820, 772, 747.

Methyl (*R*)-2-(4-chlorophenyl)-6-phenylphenanthridine-5(6H)-carboxylate (4d)

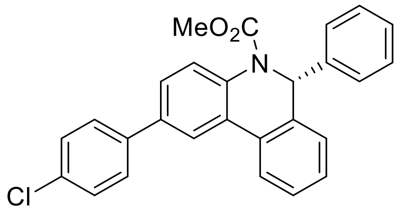

4d was obtained according to the General procedure F as a pale yellow solid (90% yield, 98% ee). ^{1}H NMR (400 MHz, $CDCl_3$) δ 7.96-7.94 (m, 2H), 7.66-7.49 (m, 4H), 7.44-7.42 (m, 5H), 7.19-7.12 (m, 5H), 6.82 (brs, 1H), 3.91 (s, 3H). ^{13}C NMR (101 MHz, $CDCl_3$) δ 155.02, 139.71, 139.07, 136.66, 135.53, 134.43, 133.38, 131.09, 128.92, 128.48, 128.25, 128.21, 128.02, 127.81, 127.41, 127.22, 126.60, 126.30, 123.81, 122.02, 58.62, 53.42. $[\alpha]_D^{25}$ = -113.5 (*c* = 1.0 mg/mL in $CHCl_3$). Melting point: 128.6-129.7 °C. ESI-HRMS calcd. for $C_{27}H_{20}ClNO_2$ $[M+Na]^+$ = 448.1075; found 448.1064. IR (neat): ν (cm^{-1}) 2925, 1710, 1484, 1219, 1065, 913, 772, 746.

Methyl (*R*)-2-(4-fluorophenyl)-6-phenylphenanthridine-5(6H)-carboxylate (4e)

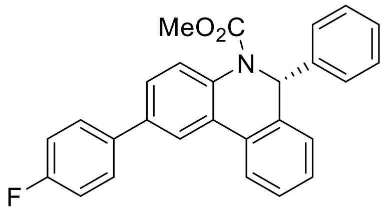

4e was obtained according to the General procedure F as a viscous oil (87% yield, 95% ee). ^{1}H NMR (400 MHz, $CDCl_3$) δ 7.96-7.93 (m, 2H), 7.60-7.42 (m, 7H), 7.22-7.13 (m, 7H), 6.83 (brs, 1H), 3.92 (s, 3H). ^{13}C NMR (101 MHz, $CDCl_3$) δ 162.45 (d, *J* = 244.9 Hz), 155.05, 139.73, 136.97, 136.75 (d, *J* = 3.1 Hz), 134.10, 131.15, 128.55 (d, *J* = 8.1 Hz), 128.35 (d, *J* = 11.8 Hz), 128.41, 128.25, 127.91 (d, *J* = 17.6 Hz), 127.41, 127.24 126.67, 126.24, 123.82, 122.06, 115.65 (d, *J* = 21.3 Hz), 58.60, 53.43. ^{19}F NMR (376 MHz, $CDCl_3$) δ -115.62. $[\alpha]_D^{25}$ = -120.6 (*c* = 1.0 mg/mL in $CHCl_3$). ESI-HRMS calcd. for $C_{28}H_{23}NO_3$ $[M+Na]^+$ = 444.1570; found 444.1560. ESI-HRMS calcd. for $C_{27}H_{20}FNO_2$ $[M+Na]^+$ = 432.1370; found 432.1362. IR (neat): ν (cm^{-1}) 2919, 1703, 1491, 1321, 1188, 964, 821, 771, 743, 696.

Methyl (*R*)-6-phenyl-2-(4-(trifluoromethoxy)phenyl)phenanthridine-5(6H)-carboxylate (4f)

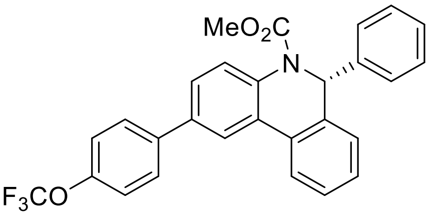

4f was obtained according to the General procedure F as a pale yellow solid (62% yield, 97% ee). ^{1}H NMR (400 MHz, $CDCl_3$) δ 7.96-7.94 (m, 2H), 7.64-7.62 (m, 3H), 7.53-7.43 (m, 4H), 7.32-7.29 (m, 2H), 7.22-7.12 (m, 5H), 6.83 (brs, 1H), 3.92(s, 3H). ^{13}C NMR (101 MHz, $CDCl_3$) δ 154.94, 148.65, 139.69, 139.39, 136.52, 135.54, 134.49, 131.06, 128.51, 128.32, 128.26, 128.07, 127.83, 127.44, 127.23, 126.73, 126.32, 123.82, 122.20, 121.25, 120.54 (q, *J* = 255.6 Hz) , 58.60, 53.45. ^{19}F NMR (376MHz, $CDCl_3$) δ -57.80. $[\alpha]_D^{25}$ = -133.4 (*c* = 1.0 mg/mL in $CHCl_3$). Melting point: 132.0-135.0 °C. ESI-HRMS calcd. for $C_{28}H_{20}F_3NO_3$ $[M+Na]^+$ = 498.1288; found 498.1276. IR (neat): ν (cm^{-1}) 2924, 1711, 1491, 1219, 913, 771, 742.

Methyl (*R*)-2-(4-nitrophenyl)-6-phenylphenanthridine-5(6H)-carboxylate (4g)

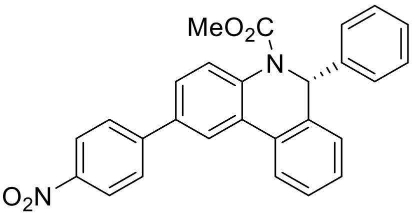

4g was obtained according to the General procedure F as a white solid (55% yield, 96% ee). ^{1}H NMR (400 MHz, $CDCl_3$) δ 8.34-8.31 (m, 2H), 8.01 (d, *J* = 2.0 Hz, 1H), 7.96 (d, *J* = 7.7 Hz, 1H), 7.79-7.77 (m, 2H), 7.55-7.42 (m, 5H), 7.20-7.10 (m, 5H), 6.83 (brs, 1H), 3.93 (s, 3H). ^{13}C NMR (101 MHz, $CDCl_3$) δ 157.38, 147.03, 139.57, 135.49, 135.30, 130.72, 128.75, 128.61, 128.31, 127.89, 127.59, 127.52, 127.20, 126.94, 126.53, 124.16, 123.82, 122.51, 58.60, 53.54. $[\alpha]_D^{25}$ = -139.0 (*c* = 1.0 mg/mL in $CHCl_3$). Melting point: 124.0-126.7 °C. ESI-HRMS calcd. for $C_{27}H_{20}N_2O_4$ $[M+Na]^+$ = 459.1315; found 459.1301. IR (neat): ν (cm^{-1}) 2920, 1715, 1324, 1244, 1063, 818, 769, 742.

Methyl (*R*)-6-phenyl-2-(o-tolyl)phenanthridine-5(6H)-carboxylate (4h)

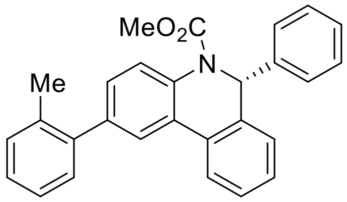

4h was obtained according to the General procedure F as a viscous oil (81% yield, 98% ee). ^{1}H NMR (400 MHz, $CDCl_3$) δ 7.88 (d, *J* = 7.6 Hz, 1H), 7.75 (d, J = 1.7 Hz, 1H), 7.49-7.42 (m, 4H), 7.31-7.29 (m, 3H), 7.25-7.15 (m, 7H), 6.85 (brs, 1H), 3.93 (s, 3H), 2.33 (s, 3H). 13C NMR (101 MHz, CDCl3) δ 155.04, 143.34, 141.29, 139.83, 138.59, 135.43, 134.83, 131.31, 130.50, 130.38, 129.77, 128.98, 128.88, 128.41, 128.21, 127.82, 127.72, 127.36, 127.29, 125.79, 125.45, 124.34, 123.77, 58.61, 53.37, 20.54. $[\alpha]_D^{25}$ = -124.3 (*c* = 1.0 mg/mL in $CHCl_3$). ESI-HRMS calcd. for $C_{28}H_{23}NO_2$ $[M+Na]^+$ = 428.1621; found 428.1610. IR (neat): ν (cm^{-1}) 2924, 1708, 1450, 1219, 913, 771, 743.

Methyl (*R*)-2-(2-methoxyphenyl)-6-phenylphenanthridine-5(6H)-carboxylate (4i)

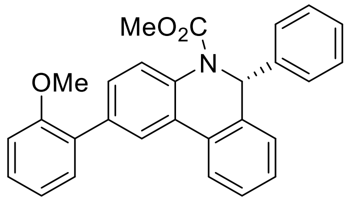

4i was obtained according to the General procedure F as a white solid (50% yield, 94% ee). ^{1}H NMR (400 MHz, $CDCl_3$) δ 7.96 (s, 1H), 7.92 (d, J = 7.7 Hz, 1H), 7.55-7.32 (m, 7H), 7,21-7,14 (m, 5H), 7.07-7.01 (m, 2H), 6.82 (brs, 1H), 3.90 (s, 3H), 3.84 (s, 3H). ^{13}C NMR (101 MHz, $CDCl_3$) δ 156.55, 139.95, 135.19, 131.46, 130.77, 130.03, 129.26, 128.66, 128.33, 128.21, 127.74, 127.63, 127.30, 125.33, 124.62, 123.82, 120.90, 99.99, 58.61, 55.62, 53.30. $[\alpha]_D^{25}$ = -123.6 (*c* = 1.0 mg/mL in $CHCl_3$). Melting point: 126.0-128.7 ºC.ESI-HRMS calcd. for $C_{28}H_{23}NO_3$ $[M+Na]^+$ = 444.1570; found 444.1558. IR (neat): v (cm^{-1}) 2954, 1693, 1490, 1320, 1245, 820, 771, 747.

Methyl (*R*)-2-(2-chlorophenyl)-6-phenylphenanthridine-5(6H)-carboxylate (4j)

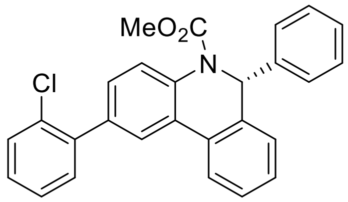

4j was obtained according to the General procedure F as a viscous oil (47% yield, 96% ee). ^{1}H NMR (400 MHz, $CDCl_3$) δ 7.91-7.88 (m, 2H), 7.52-7.45 (m, 3H), 7.41-7.30 (m, 6H), 7.20-7.13 (m, 5H), 6.83 (brs, 1H), 3.92 (s, 3H). 13C NMR (101 MHz, CDCl3) δ 155.03, 139.88, 139.80, 135.92, 135.43, 134.22, 132.53, 131.35, 131.18, 130.05, 129.10, 128.77, 128.57, 128.43, 128.24, 127.89, 127.71, 127.39, 127.29, 126.97, 126.85, 124.77, 123.85, 58.63, 53.38. $[\alpha]_D^{25}$ = -122.0 (*c* = 1.0 mg/mL in $CHCl_3$). ESI-HRMS calcd. for $C_{27}H_{20}ClNO_2$ $[M+Na]^+$ = 448.1075; found 448.1063. IR (neat): v (cm^{-1}) 2923, 1701, 1320, 1219, 913, 771, 745.

Methyl (*R*)-6-phenyl-2-(m-tolyl)phenanthridine-5(6H)-carboxylate (4k)

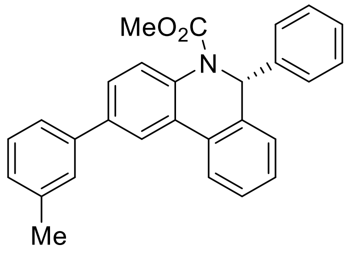

4k was obtained according to the General procedure F as a white solid (65% yield, 96% ee). ^{1}H NMR (400 MHz, $CDCl_3$) δ 7.99-7.97 (m, 2H), 7.54-7.35 (m, 8H), 7.22-7.14 (m, 6H), 6.84 (brs, 1H), 3.92 (s, 3H), 2.46 (s, 3H). ^{13}C NMR (101 MHz, $CDCl_3$) δ 155.09, 143.33, 140.62, 139.81, 138.36, 138.09, 135.56, 134.04, 131.35, 130.50, 128.98, 128.70, 128.45, 128.31, 128.24, 128.06, 127.88, 127.78, 127.39, 127.27, 126.84, 126.15, 124.11, 123.86, 122.23, 58.66, 53.37, 21.55. $[\alpha]_D^{25}$ = -145.9 (*c* = 1.0 mg/mL in $CHCl_3$). Melting point: 123.0-125.0 ºC. ESI-HRMS Calcd. For $C_{27}H_{20}N_2O_4$ $[M+Na]^+$ = 459.1315; found 459.1301. ESI-HRMS calcd. for $C_{28}H_{23}NO_2$ $[M+Na]^+$ = 428.1621; found 428.1610. IR (neat): v (cm^{-1}) 2921, 1702, 1219, 913, 772, 743.

Methyl (*R*)-2-(3-chlorophenyl)-6-phenylphenanthridine-5(6H)-carboxylate (4l)

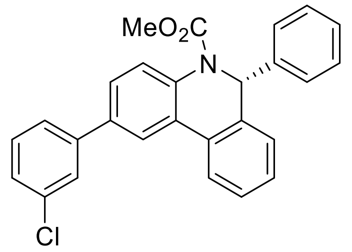

4l was obtained according to the General procedure F as a white solid (77% yield, 98% ee).
^{1}H NMR (400 MHz, $CDCl_3$) δ 7.97-7.95 (m, 2H), 7.61 (t, J = 1.8 Hz, 1H), 7.56-7.33 (m, 8H), 7.21-7.11 (m, 5H), 6.83 (brs, 1H), 3.92 (s, 3H). ^{13}C NMR (101 MHz, $CDCl_3$) δ 155.00, 142.46, 139.67, 136.47, 135.52, 134.69, 131.05, 130.02, 128.52, 128.27, 128.07, 127.80, 127.45, 127.28, 127.24, 127.12, 126.73, 126.29, 125.14, 123.87, 122.20, 58.60, 53.46. $[\alpha]_D^{25}$ = -154.8 (c = 1.0 mg/mL in $CHCl_3$). Melting point: 124.5-127.8 °C. ESI-HRMS calcd. for $C_{27}H_{20}ClNO_2$ $[M+Na]^+$ = 448.1075; found 448.1063. IR (neat): v (cm^{-1}) 2953, 1701, 1448, 1321, 1269, 768, 699.

Methyl (*R*)-2-(benzo[d][1,3]dioxol-5-yl)-6-phenylphenanthridine-5(6H)-carboxylate (4m)

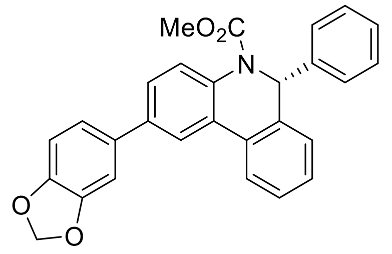

4m was obtained according to the General procedure F as a white solid (43% yield, 95% ee).
^{1}H NMR (400 MHz, $CDCl_3$) δ 7.94 (d, J = 7.8 Hz, 1H), 7.90 (d, J = 2.0 Hz, 1H), 7.52-7.39 (m, 5H), 7.21-7.08 (m, 7H), 6.91-6.89 (m, 1H), 6.81 (brs, 1H), 6.03 (s, 2H), 3.90 (s, 3H). ^{13}C NMR (101 MHz, $CDCl_3$) δ 148.13, 147.08, 139.75, 137.66, 135.49, 135.00, 133.77, 131.23, 128.44, 128.29, 128.22, 127.89, 127.78, 127.37, 127.24, 126.53, 123.81, 121.90, 120.48, 108.58, 107.52, 101.17, 58.58, 53.39. $[\alpha]_D^{25}$ = -127.8 (c = 1.0 mg/mL in $CHCl_3$). Melting point: 126.0-128.4 °C. ESI-HRMS calcd. for $C_{28}H_{21}NO_4$ $[M+Na]^+$ = 458.1363; found 458.1350. IR (neat): v (cm^{-1}) 2924, 1709, 1219, 913, 772, 743.

Methyl (*R*)-6-phenyl-2-(thiophen-2-yl)phenanthridine-5(6H)-carboxylate (4n)

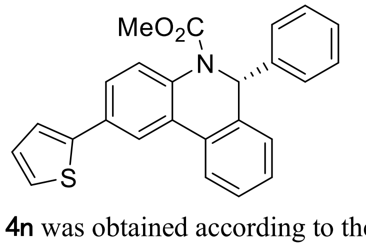

4n was obtained according to the General procedure F as a viscous oil (50% yield, 92% ee).
^{1}H NMR (400 MHz, $CDCl_3$) δ 7.99-7.94 (m, 2H), 7.51-7.41 (m, 5H), 7.33-7.30 (m, 2H), 7.18-7.10 (m, 6H), 6.80 (brs, 1H), 3.90 (s, 3H). ^{13}C NMR (101 MHz, $CDCl_3$) δ 154.98, 143.89, 143.32, 139.67, 135.55, 131.28, 130.99, 130.48, 128.96, 128.47, 128.39, 128.24, 128.04, 128.01, 127.74, 127.42, 127.22, 126.28, 125.64, 125.46, 124.70, 123.83, 123.07, 121.01, 58.62, 53.38. $[\alpha]_D^{25}$ = -120.9 (c = 1.0 mg/mL in $CHCl_3$). ESI-HRMS calcd. for $C_{25}H_{19}NO_2S$ $[M+Na]^+$ = 420.1029; found 420.1021. IR (neat): v (cm^{-1}) 2919, 1710, 1472, 1318, 1219, 964, 773.

Methyl (*R*)-3,6-diphenylphenanthridine-5(6H)-carboxylate (4o)

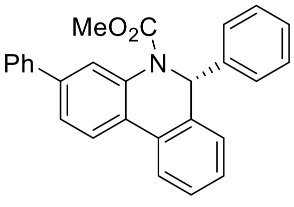

4o was obtained according to the General procedure F as a white solid (80% yield, 96% ee).
^{1}H NMR (400 MHz, $CDCl_3$) δ 7.92 (d, *J* = 7.7 Hz, 1H), 7.84 (d, *J* = 8.2 Hz, 1H), 7.64-7.36 (m, 10H), 7.19-7.13 (m, 5H), 6.85 (brs, 1H), 3.93 (s, 3H). ^{13}C NMR (101 MHz, $CDCl_3$) δ 154.99, 140.78, 140.36, 139.78, 135.34, 135.18, 131.13, 128.77, 128.47, 128.25, 127.78, 127.53, 127.38, 127.25, 127.13, 127.07, 124.60, 123.95, 123.87, 123.78, 58.63, 53.44. $[\alpha]_D^{25}$ = -171.0 (*c* = 1.0 mg/mL in $CHCl_3$). Melting point: 120.0-122.7 °C. ESI-HRMS calcd. for $C_{27}H_{21}NO_2$ $[M+Na]^+$ = 414.1465; found 414.1453. IR (neat): ν (cm^{-1}) 2923, 1709, 1439, 1219, 913, 771, 744, 696.

Methyl (*R*)-2,6-diphenylbenzo[c][1,5]naphthyridine-5(6H)-carboxylate (4p)

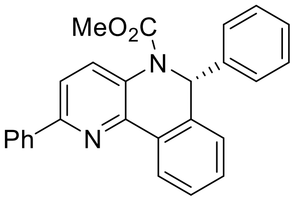

4p was obtained according to the General procedure F as a white solid (95% yield, 97% ee).
^{1}H NMR (400 MHz, $CDCl_3$) δ 8.66 (d, *J* = 7.7 Hz, 1H), 8.17-8.15 (m, 2H), 7.95-7.37 (m, 8H), 7.20-7.14 (m, 5H), 6.85 (brs, 1H), 3.94 (s, 3H). ^{13}C NMR (101 MHz, $CDCl_3$) δ 154.82, 152.71, 144.99, 139.91, 138.89, 135.99, 133.21, 131.65, 129.55, 128.82, 128.70, 128.53, 128.41, 127.68, 127.36, 127.07, 126.73, 125.33, 119.20, 58.46, 53.56. $[\alpha]_D^{25}$ = -126.9 (*c* = 1.0 mg/mL in $CHCl_3$). Melting point: 145.0-148.0 °C. ESI-HRMS calcd. for $C_{26}H_{20}N_2O_2$ $[M+H]^+$ = 393.1598; found 393.1587. IR (neat): ν (cm^{-1}) 2922, 1715, 1442, 1219, 913, 772, 744, 699.

Methyl (*R*)-2,6-diphenylbenzo[c][1,8]naphthyridine-5(6H)-carboxylate (4q)

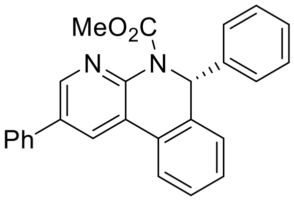

4q was obtained according to the General procedure F as a white solid (99% yield, 98% ee).
^{1}H NMR (400 MHz, $CDCl_3$) δ 8.64 (d, *J* = 1.9 Hz, 1H), 8.24 (d, *J* = 2.3 Hz, 1H), 7.93 (d, *J* = 7.1 Hz, 1H), 7.63-7.61 (m, 2H), 7.54-7.48 (m, 5H), 7.44-7.40 (m, 1H), 7.22-7.15 (m, 5H), 6.87 (s, 1H), 3.95 (s, 3H). 13C NMR (101 MHz, $CDCl_3$) δ 154.94, 147.45, 146.27, 139.41, 137.31, 135.72, 133.96, 130.25, 129.87, 129.09, 128.74, 128.59, 128.31, 128.08, 127.98, 127.45, 127.13, 127.02, 124.00, 123.07, 59.41, 53.78. $[\alpha]_D^{25}$ = -116.9 (*c* = 1.0 mg/mL in $CHCl_3$). Melting point: 183.8-185.4 °C. ESI-HRMS calcd. for $C_{26}H_{20}N_2O_2$ $[M+H]^+$ = 393.1598; found 393.1588. IR (neat): ν (cm^{-1}) 2923, 1701, 1432, 1220, 913, 771, 749, 696.

Methyl (*R*)-3-methyl-2,6-diphenylbenzo[c][1,8]naphthyridine-5(6H)-carboxylate (4r)

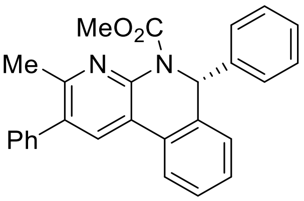

4r was obtained according to the General procedure F as a white solid (94% yield, 97% ee).

^{1}H NMR (400 MHz, CDCl$_3$) δ 7.90 (s, 1H), 7.83-7.81 (m, 1H), 7.49-7.37 (m, 8H), 7.21-7.15 (m, 5H), 6.87 (s, 1H), 3.93 (s, 3H), 2.50 (s, 3H). ^{13}C NMR (101 MHz, CDCl$_3$) δ 155.05, 154.56, 146.47, 139.63, 139.42, 135.22, 134.47, 133.10, 129.17, 128.44, 128.26, 127.95, 127.57, 127.37, 127.35, 123.66, 59.40, 53.63, 23.03. $[\alpha]_D^{25}$ = -152.0 (*c* = 1.0 mg/mL in CHCl$_3$). Melting point: 180.0-182.7 ºC. ESI-HRMS calcd. for $C_{27}H_{22}N_2O_2$ $[M+H]^+$ = 407.1754; found 407.1746. IR (neat): ν (cm^{-1}) 2920, 1703, 1422, 1274, 1072, 772, 752, 698.

Methyl (*R*)-1-methyl-2,6-diphenylbenzo[c][1,8]naphthyridine-5(6H)-carboxylate (4s)

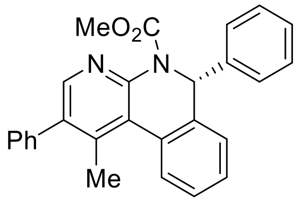

4s was obtained according to the General procedure F as a white solid (98% yield, 84% ee).

^{1}H NMR (400 MHz, CDCl$_3$) δ 8.24 (s, 1H), 7.89-7.87 (m, 1H), 7.56-7.38 (m, 6H), 7.34-7.32 (m, 2H), 7.21-7.14 (m, 5H), 6,78 (s, 1H), 3.92 (s, 3H), 2.47 (s, 3H). ^{13}C NMR (101 MHz, CDCl$_3$) δ 154.98, 148.37, 147.29, 142.32, 138.86, 138.25, 138.16, 136.62, 130.48, 129.66, 128.82, 128.43, 128.18, 127.90, 127.85, 127.65, 127.39, 127.23, 127.14, 123.71, 59.45, 53.70, 20.81. $[\alpha]_D^{25}$ = -100.3 (*c* = 1.0 mg/mL in CHCl$_3$). Melting point: 182.3-185.0 ºC. ESI-HRMS calcd. for $C_{27}H_{22}N_2O_2$ $[M+H]^+$ = 407.1754; found 407.1744. IR (neat): ν (cm^{-1})2920, 1730, 1430, 1254, 1082, 772, 747, 704.

Methyl (*R*)-9-methyl-2-phenyl-6-(p-tolyl)phenanthridine-5(6H)-carboxylate (4t)

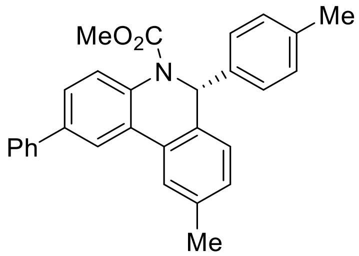

4t was obtained according to the General procedure F as a white solid (92% yield, 97% ee).

^{1}H NMR (400 MHz, CDCl$_3$) δ 7.99 (d, *J* = 2.0 Hz, 1H), 7.78 (s, 1H), 7.67-7.65 (m, 2H), 7.50-7.46 (m, 4H), 7.40-7.36 (m, 1H), 7.31-7.29 (m, 1H), 7.25-7.23 (m, 1H), 7.04-6.98 (m, 4H), 6.77 (s, 1H), 3.91 (s, 3H), 2.51 (s, 3H), 2.25 (s, 3H). ^{13}C NMR (101 MHz, CDCl$_3$) δ 154.99, 140.71, 138.01, 137.82, 137.06, 136.98, 132.97, 131.03, 128.93, 128.77, 128.71, 128.49, 127.59, 127.25, 127.19, 127.01, 126.63, 126.17, 124.32, 122.15, 58.23, 53.33, 21.55, 21.00. $[\alpha]_D^{25}$ = -146.9 (*c* = 1.0 mg/mL in CHCl$_3$). Melting point: 181.0-183.7 ºC. ESI-HRMS calcd. for $C_{29}H_{25}NO_2$ $[M+Na]^+$ = 442.1778; found 442.1765. IR (neat): ν (cm^{-1}) 2918, 1693, 1486, 1317, 1256, 1028, 755, 694.

Methyl (*R*)-9-methoxy-6-(4-methoxyphenyl)-2-phenylphenanthridine-5(6H)-carboxylate (4u)

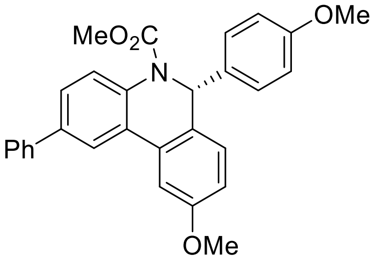

4u was obtained according to the General procedure F as a white solid (89% yield, 98% ee).
^{1}H NMR (400 MHz, $CDCl_3$) δ 7.96 (s, 1H), 7.66-7.29 (m, 9H), 7.05-7.96 (m, 3H), 6.73-6.71 (m, 3H), 3.94-3.91 (m, 6H), 3.71 (s, 1H). ^{13}C NMR (101 MHz, $CDCl_3$) δ 159.73, 158.76, 140.63, 137.88, 134.20, 132.36, 128.80, 128.62, 128.49, 128.36, 127.32, 127.02, 126.91, 126.25, 122.21, 113.60, 113.56, 109.07, 57.66, 55.56, 55.12, 53.35. $[\alpha]_D^{25}$ = -156.8 (*c* = 1.0 mg/mL in $CHCl_3$). Melting point: 182.0-185.0 °C. ESI-HRMS calcd. for $C_{29}H_{25}NO_4$ $[M+Na]^+$ = 474.1676; found 474.1660. IR (neat): ν (cm^{-1}) 2955, 2920, 1692, 1488, 1246, 1134, 1023, 771, 757, 692.

Methyl (*R*)-9-fluoro-6-(4-fluorophenyl)-2-phenylphenanthridine-5(6H)-carboxylate (4v)

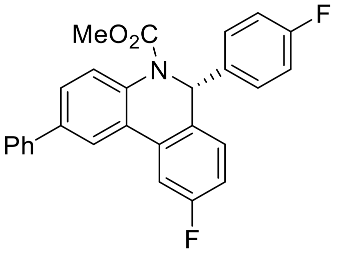

4v was obtained according to the General procedure F as a white solid (66% yield, 96% ee).
^{1}H NMR (400 MHz, $CDCl_3$) δ 7.91 (s, 1H), 7.67-7.62 (m, 4H), 7.53-7.46 (m, 3H), 7.41-7.35 (m, 2H), 7.15-7.05 (m, 3H), 6.90-6.85 (m, 2H), 6.80 (brs, 1H), 3.92 (s, 3H). ^{13}C NMR (101 MHz, $CDCl_3$) δ 162.99 (d, *J* = 244.3 Hz), 162.09 (d, *J* = 245.0 Hz), 154.91, 140.23, 138.25, 135.36 (d, *J* = 3.1 Hz), 133.76, 133.32 (d, *J* = 8.1 Hz), 131.08, 129.20 (d, *J* = 8.2 Hz), 128.94 (d, *J* = 7.8 Hz), 128.86, 127.51, 127.41 (d, *J* = 1.5 Hz), 126.97, 126.24, 122.33, 115.19 (d, *J* = 21.3 Hz), 114.93 (d, *J* = 22.0 Hz), 110.73 (d, *J* = 22.9 Hz), 57.41, 53.54. ^{19}F NMR (376 MHz, $CDCl_3$) δ -112.92, -114.79. $[\alpha]_D^{25}$ = -165.8 (*c* = 1.0 mg/mL in $CHCl_3$). Melting point: 131.0-133.4 °C. ESI-HRMS calcd. for $C_{27}H_{19}F_2NO_2$ $[M+Na]^+$ = 450.1276; found 450.1263. IR (neat): ν (cm^{-1}) 2957, 2919, 1684, 1489, 1443, 1325, 1313, 1258, 1225, 1181, 959, 764, 757, 695.

Methyl (*R*)-7-methyl-2-phenyl-6-(o-tolyl)phenanthridine-5(6H)-carboxylate (4w)

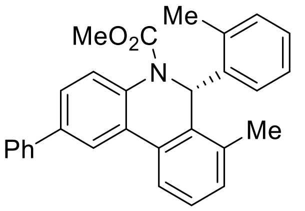

4w was obtained according to the General procedure F as a viscous oil (37% yield, 89% ee).
^{1}H NMR (400 MHz, $CDCl_3$) δ 8.04 (d, J = 1.9 Hz, 1H), 7.87 (d, J = 7.8 Hz, 1H), 7.68-7.65 (m, 2H), 7.50-7.36 (m, 7H), 7.25-7.18 (m, 2H), 7.07 (t, J = 7.4 Hz, 1H), 6.77 (d, J = 7.5 Hz, 1H), 6.40 (s, 1H), 3.82 (s, 3H), 2.72 (s, 3H), 2.29 (s, 3H). ^{13}C NMR (101 MHz, $CDCl_3$) δ 154.95, 140.73, 138.70, 137.43, 136.31, 135.41, 134.78, 133.92, 132.11, 130.79, 130.20, 129.98, 128.77, 128.57,

127.85, 127.75, 127.30, 127.04, 126.39, 125.50, 122.35, 121.50, 53.37, 53.31, 20.02, 18.81. $[\alpha]_D^{25}$ = -105.5 (*c* = 1.0 mg/mL in $CHCl_3$). ESI-HRMS calcd. for $C_{29}H_{25}NO_2$ $[M+Na]^+$ = 442.1778; found 442.1766. IR (neat): ν (cm^{-1})2921, 1706, 1492, 1316, 1188, 1081, 964, 771, 745, 697.

Methyl (*R*)-7-fluoro-6-(2-fluorophenyl)-2-phenylphenanthridine-5(6H)-carboxylate (4x)

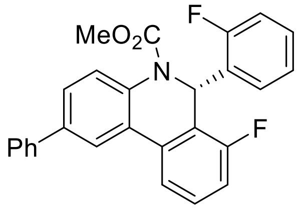

4x was obtained according to the General procedure F as a white solid (86% yield, 91% ee).

^{1}H NMR (400 MHz, $CDCl_3$) δ 8.03 (d, *J* = 2.0 Hz, 1H), 7.79 (d, *J* = 7.8 Hz, 1H), 7.67-7.61 (m, 3H), 7.54-7.38 (m, 6H), 7.21-7.04 (m, 3H), 6.81 (td, *J* = 7.6, 0.9 Hz, 1H), 6.59 (td, *J* = 7.6, 1.2Hz, 1H), 3.90 (s, 3H). ^{13}C NMR (101 MHz, $CDCl_3$) δ 160.82 (d, *J* = 192.7 Hz), 158.35 (d, *J* = 190.1 Hz), 154.32, 140.40, 138.32, 134.14, 133.92 (d, *J* = 4.2 Hz), 129.82 (d, *J* = 8.2 Hz), 129.60 (d, *J* = 8.3 Hz), 129.21 (d, *J* = 3.2 Hz), 128.86, 127.86 (d, *J* = 3.0 Hz), 127.47, 127.40, 127.00, 126.77, 125.35 (d, *J* = 13.7 Hz), 123.74 (d, *J* = 3.6 Hz), 122.81 (d, *J* = 16.4 Hz), 122.38, 119.25 (d, J = 3.2 Hz), 115.82 (d, *J* = 21.8 Hz), 114.68 (d, *J* = 21.0 Hz), 53.50, 47.04. ^{19}F NMR (376 MHz, $CDCl_3$) δ -115.49, -118.83. $[\alpha]_D^{25}$ = -123.0 (*c* = 1.0 mg/mL in $CHCl_3$). Melting point: 124.0-126.6 °C. ESI-HRMS calcd. for $C_{27}H_{19}F_2NO_2$ $[M+Na]^+$ = 450.1276; found 450.1268. IR (neat): ν (cm^{-1}) 2920, 1713, 1460, 1217, 1277, 1238, 755, 697.

Methyl(*R*)-6-(3,5-dimethylphenyl)-8,10-dimethyl-2-phenylphenanthridine-5(6H)-carboxylate (4y)

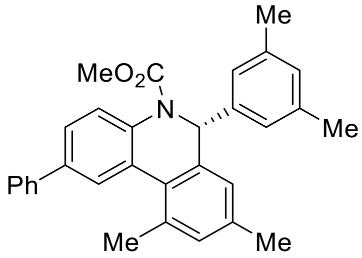

4y was obtained according to the General procedure F as a white solid (91% yield, 97% ee).

^{1}H NMR (400 MHz, $CDCl_3$) δ 7.96 (s, 1H), 7.99-7.35 (m, 7H), 7.18 (s, 1H), 7.10 (s, 1H), 6.78-6.53 (m, 4H), 3.90 (s, 3H), 2.77 (s, 3H), 2.43 (s, 3H), 2.18 (s, 6H). ^{13}C NMR (101 MHz, $CDCl_3$) δ 155.01, 140.94, 139.26, 138.50, 137.52, 137.13, 134.85, 132.68, 129.62, 129.05, 128.82, 128.26, 127.16, 126.95, 126.46, 126.23, 125.71, 125.29, 59.44, 53.27, 23.16, 21.30, 21.06. $[\alpha]_D^{25}$ = -99.0 (*c* = 1.0 mg/mL in $CHCl_3$). Melting point: 171.8-174.0 °C. ESI-HRMS calcd. for $C_{31}H_{29}NO_2$ $[M+Na]^+$ = 470.2091; found 470.2081. IR (neat): ν (cm^{-1}) 2920, 1693, 1474, 1324, 1257, 1023, 771, 758, 747, 692.

Methyl (*R*)-8-phenyl-4-(thiophen-2-yl)thieno[2,3-c]quinoline-5(4H)-carboxylate (4z)

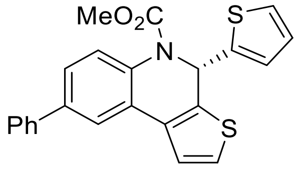

4z was obtained according to the General procedure F as a white solid (97% yield, 93% ee).

^{1}H NMR (400 MHz, $CDCl_3$) δ 7.80 (d, *J* = 2.2 Hz, 1H), 7.66-7.64 (m, 3H), 7.50-7.46 (m, 4H), 7.40-7.36 (m, 2H), 7.22 (s, 1H), 7.15 (dd, *J* = 5.1, 1.2 Hz, 1H), 6.83 (dd, *J* = 5.0, 3.6 Hz, 1H), 6.77-6.76 (m, 1H), 3.93 (s, 3H). ^{13}C NMR (101 MHz, $CDCl_3$) δ 154.71, 143.27, 140.51, 138.09, 133.57, 131.74, 128.80, 127.35, 127.01, 126.57, 125.81, 125.72, 125.62, 125.19, 122.55, 121.90, 53.62, 51.91. $[\alpha]_D^{25}$ = -132.4 (*c* = 1.0 mg/mL in $CHCl_3$). Melting point: 140.1-143.3 °C. ESI-HRMS calcd. for $C_{23}H_{17}NO_2S_2$ $[M+Na]^+$ = 426.0593; found 426.0580. IR (neat): ν (cm^{-1}) 2920, 1710, 1439, 1372, 1287, 1108, 765, 759, 693, 661.

Methyl benzhydryl(4,4''-dimethyl-[1,1':3',1''-terphenyl]-4'-yl)carbamate (4bb)

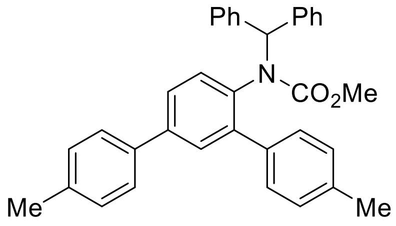

4bb was obtained from rival side reaction in the cascade reaction as a viscous oil.

^{1}H NMR (400 MHz, $CDCl_3$) δ 7.51-7.43 (m, 4H), 7.26-7.14 (m, 11H),7.04-6.95 (m, 6H), 6.00 (s, 1H), 3.64 (s, 3H), 2.44 (s, 3H), 2.41 (s, 3H).^{13}C NMR (101 MHz, $CDCl_3$) δ 159.05, 141.59, 140.30, 138.63, 138.09, 137.35, 137.17, 136.96, 136.79, 129.97, 129.63, 129.49, 128.86, 128.83, 128.40, 128.01, 127.77, 127.42, 126.87, 126.75, 125.93, 68.90, 52.80, 21.17, 21.10. ESI-HRMS calcd. for $C_{35}H_{31}NO_2$ $[M+Na]^+$ = 520.2247; found 520.2249. IR (neat): ν (cm^{-1}) 2925, 1698, 1437, 1219, 1036, 772, 743. IR (neat): ν (cm^{-1}) 2983, 1705, 1440, 1313, 1065, 764, 730, 611.

Methyl benzhydryl(3-bromo-4'-methyl-[1,1'-biphenyl]-4-yl)carbamate (3ac)

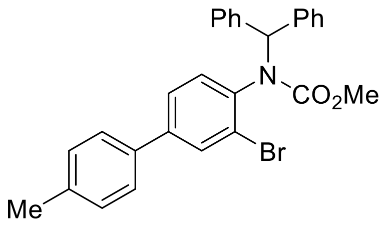

3ac was obtained according to the General procedure H as a viscous oil. (57% yield).

^{1}H NMR (400 MHz, $CDCl_3$) δ 7.63-7.62 (m, 1H), 7.45-7.39 (m, 6H), 7.36-7.32 (m, 2H), 7.25-7.23 (m, 3H), 7.18-7.13 (m, 5H), 6.76 (s, 1H), 3.77 (s, 3H), 2.40 (s, 3H). ^{13}C NMR (101 MHz, $CDCl_3$) δ 156.08, 141.62, 140.84, 137.91, 137.86, 137.28, 135.87, 131.19, 130.92, 130.44, 129.57, 128.84, 128.27, 127.76, 127.08, 126.78, 126.44, 125.73, 66.96, 53.37, 21.09. ESI-HRMS calcd. for $C_{28}H_{24}BrNO_2$ $[M+Na]^+$ = 508.0883; found 508.0881. IR (neat): ν (cm^{-1}) 2925, 1698, 1437, 1219, 1036, 772, 743.

Methyl (*R*)-6-(p-tolyl) phenanthridine-5(6H)-carboxylate and methyl (*R*)-9-methyl-6-phenylphenanthridine-5(6H)-carboxylate (8a/8b)

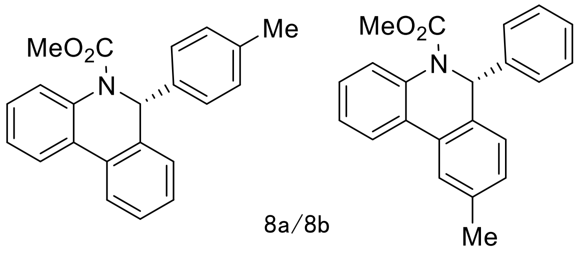

8a/8b

8a/8b mixture was obtained according to the General procedure D, as a viscous oil (91% combined yield with 1:1 ratio determined by ^{1}H NMR). 2.4:97.4 e.r. and 2.4:97.6 e.r.

^{1}H NMR (400 MHz, $CDCl_3$) δ 7.87 (d, *J* = 7.8 Hz, 1H), 7.80-7.75 (m, 2H), 7.69 (s, 1H), 7.49-7.31 (m, 5H), 7.28-7.07 (m, 11H, 6.96 (s, 4H), 6.76 (s, 2H), 3.87 (s, 6H), 2.48 (s, 3H), 2.23 (s, 3H). ^{13}C NMR (101 MHz, $CDCl_3$) δ 155.06, 143.35, 139.94, 138.03, 136.98, 136.77, 135.75, 134.83, 132.72, 131.29, 131.12, 130.52, 128.99, 128.89, 128.57, 128.41, 128.30, 128.20, 128.14, 127.92, 127.76, 127.61, 127.56, 127.25, 127.21, 127.17, 125.99, 125.45, 125.06, 124.36, 123.76, 123.58, 123.51, 58.30, 53.28, 21.55, 20.98. ESI-HRMS calcd. for $C_{22}H_{19}NO_2$ $[M+Na]^+$ = 352.1308; found 352.1301. IR (neat): ν (cm^{-1}) 2920, 1706, 1435, 1271, 1045, 772, 758.

Methyl (*R*)-6-(4-fluorophenyl) phenanthridine-5(6H)-carboxylate and methyl (*R*)-9-fluoro-6-phenylphenanthridine-5(6H)-carboxylate (**9a/9b**)

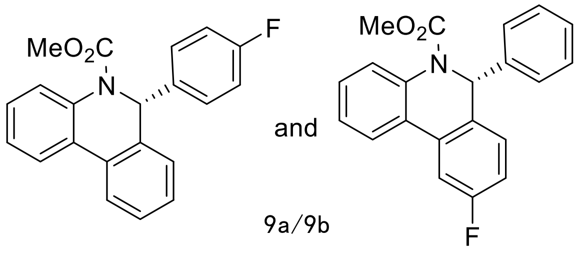

9a/9b

9a/9b mixture was obtained according to the General procedure D, as a viscous oil (93% combined yield with 1:1 ratio determined by ^{19}F NMR). 3.3:96.7 e.r. and 1.2 :98.8 e.r.

^{1}H NMR (400 MHz, $CDCl_3$) δ 7.91-7.69 (m, 3H), 7.59-7.35 (m, 6H), 7.31-7.04 (m, 14H), 6.88-6.80 (m, 3H), 3.90 (d, 6H). ^{13}C NMR (101 MHz, $CDCl_3$) δ 164.13, 163.24, 161.69, 160.80, 155.10, 154.95, 139.53, 135.55, 135.53, 134.80, 134.76, 133.46, 133.38, 131.31, 129.29, 129.21, 129.08, 129.00, 128.68, 128.60, 128.28, 128.11, 127.93, 127.61, 127.51, 127.35, 127.18, 126.09, 125.29, 123.94, 123.78, 123.68, 115.19, 114.98, 114.80, 114.58, 110.77, 110.54, 57.95, 57.87, 53.47, 53.43. ^{19}F NMR (376 MHz, $CDCl_3$) δ -113.41, -115.19. ESI-HRMS calcd. for $C_{21}H_{16}FNO_2$ $[M+Na]^+$ = 356.1057; found 356.1055. IR (neat): ν (cm^{-1}) 2952, 1700, 1438, 1326, 1250, 1020, 773, 762.

Ethyl (*R*)-6-(4-methoxyphenyl)phenanthridine-5(6H)-carboxylate and ethyl (*R*)-9-methoxy-6-phenylphenanthridine-5(6H)-carboxylate (10a/10b)

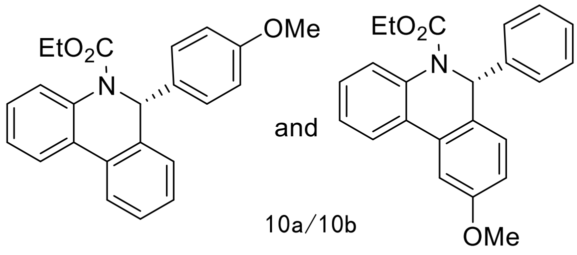

10a/10b

10a/10b mixture was obtained according to the General procedure D, as a viscous oil (90% combined yield with 1:1 ratio determined by ^{1}H NMR). 1.8:98.2 e.r. and 0.7:99.3 e.r.

^{1}H NMR (400 MHz, $CDCl_3$) δ 7.88 (d, J = 7.9 Hz, 1H), 7.80-7.74 (m, 2H), 7.50-7.31 (m, 6H), 7.26-7.09 (m, 10H), 7.02-6.95 (m, 3H), 6.77-6.69 (m, 4H), 4.41-4.32 (m, 4H), 3.94 (s, 3H), 3.71 (s, 3H), 1.38 (t, J = 7.1 Hz, 6H). ^{13}C NMR (101 MHz, $CDCl_3$) δ 159.74, 158.75, 140.29, 135.91, 135.04, 134.93, 132.56, 132.14, 131.37, 128.70, 128.55, 128.29, 128.16, 128.04, 127.93, 127.77, 127.57, 127.24, 126.04, 124.92, 123.76, 123.59, 113.56, 113.42, 109.10, 62.42, 62.38, 57.87, 55.52, 55.15, 14.60. ESI-HRMS calcd. for $C_{23}H_{21}NO_3$ $[M+Na]^+$ = 382.1414; found 382.1415. IR (neat): ν (cm^{-1}) 2954, 1705, 1440, 1223, 1057, 759, 743.

Single crystal of 2s (CCDC Number: 2307693)
Bond precision: C-C = 0.0022 A Wavelength=1.54184
Cell: a=11.43405(6) b=11.43405(6) c=25.89293(13)
alpha=90 beta=90 gamma=120
Temperature: 150 K
Calculated Reported
Volume 2931.65(3) 2931.65(3)
Space group P 31 2 1 P 31 2 1
Hall group P 31 2" P 31 2"
Moiety formula C25 H25 N O2, 0.083(H2 O) C25 H25 N O2, 0.08(H2 O1)
Sum formula C25 H25.17 N O2.08 C25 H25.17 N O2.08
Mr 372.96 372.96
Dx,g cm-3 1.268 1.268
Z 6 6
Mu (mm-1) 0.628 0.628
F000 1193.0 1193.0
F000' 1196.35
h,k,lmax 14,14,32 14,14,32
Nref 4090[2353] 3980
Tmin,Tmax 0.963,0.975 0.735,1.000
Tmin' 0.910
Correction method= # Reported T Limits: Tmin=0.735 Tmax=1.000
AbsCorr = MULTI-SCAN
Data completeness= 1.69/0.97 Theta(max)= 76.125
R(reflections)= 0.0272(3934)
wR2(reflections)=
0.0733(3980)
S = 1.026 Npar= 268

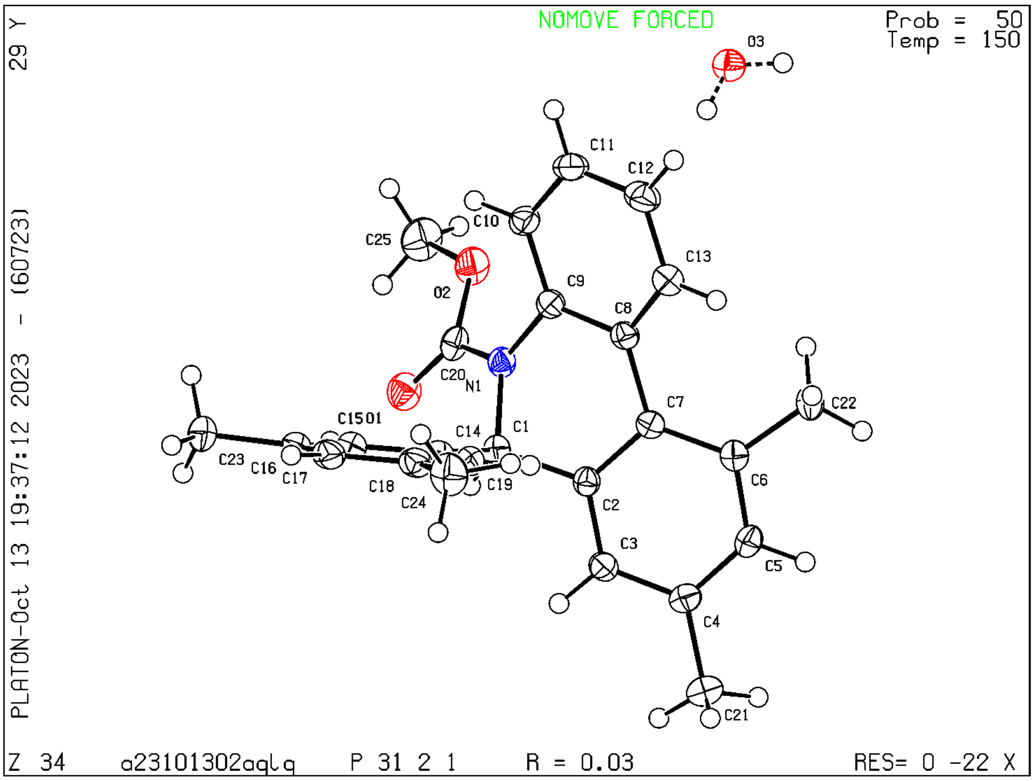

Reference

[1] S. Wang, J. Li, T. Miao, W. Wu, Q. Li, Y. Zhuang, Z. Zhou, L. Qiu, Org. Lett. 2012, 14, 8
[2] L.Yang, M. Neuburger, O. Baudoin, Angew. Chem. Int. Ed. 2018, 57, 1394
[3] A. Tait, A. Luppi, R. Avallone, M. Baraldi, Il Farmaco. 2005, 60, 653.

NMR Spectra

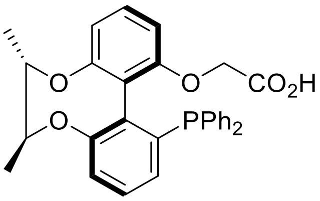

L6

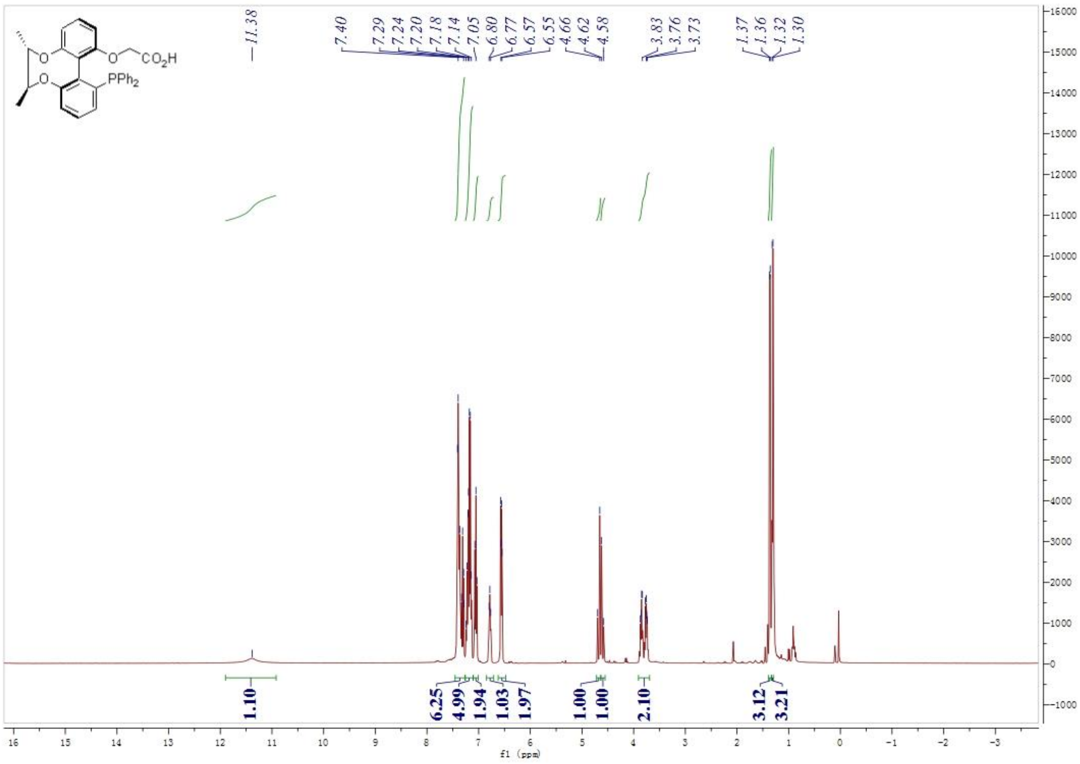
CO2H
PPh2
11.38
7.40
7.29
7.24
7.20
7.18
7.14
7.05
6.80
6.77
6.57
6.55
4.66
4.62
4.58
3.83
3.76
3.73
1.37
1.36
1.32
1.30
1.10
6.25
4.99
1.94
1.03
1.97
1.00
1.00
2.10
3.12
3.21
f1 (ppm)

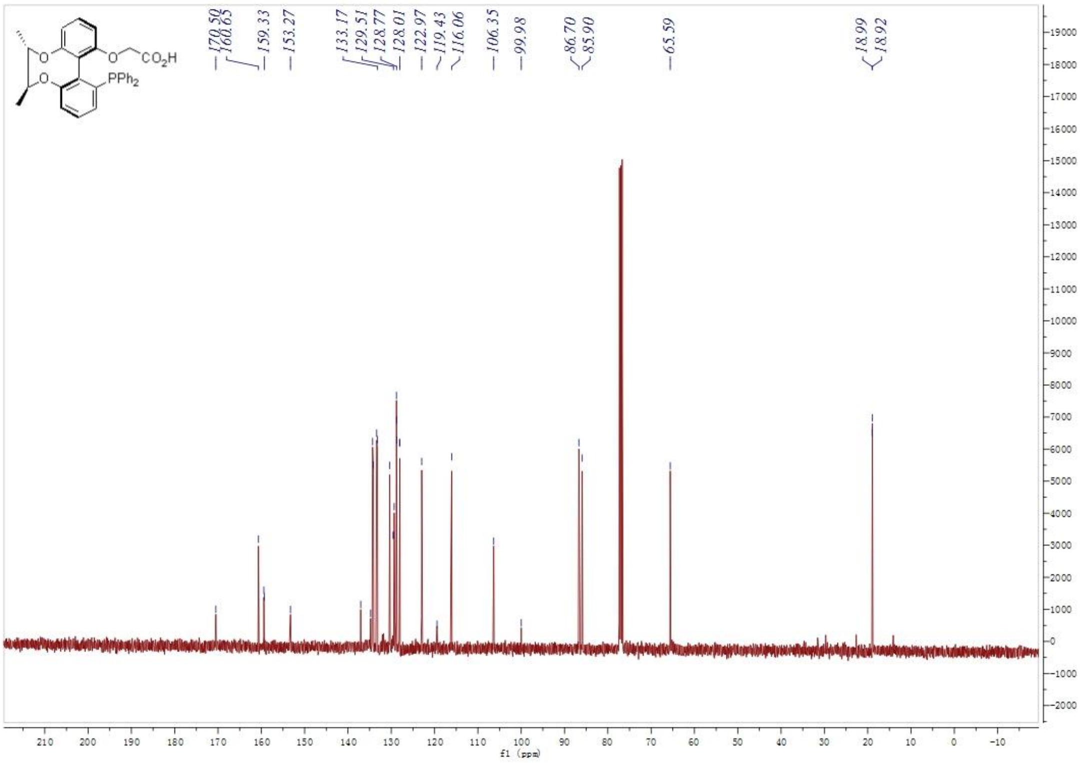
CO2H
PPh2
170.50
160.65
159.33
153.27
133.17
129.51
128.77
128.01
122.97
119.43
116.06
106.35
99.98
86.70
85.90
65.59
18.99
18.92
f1 (ppm)

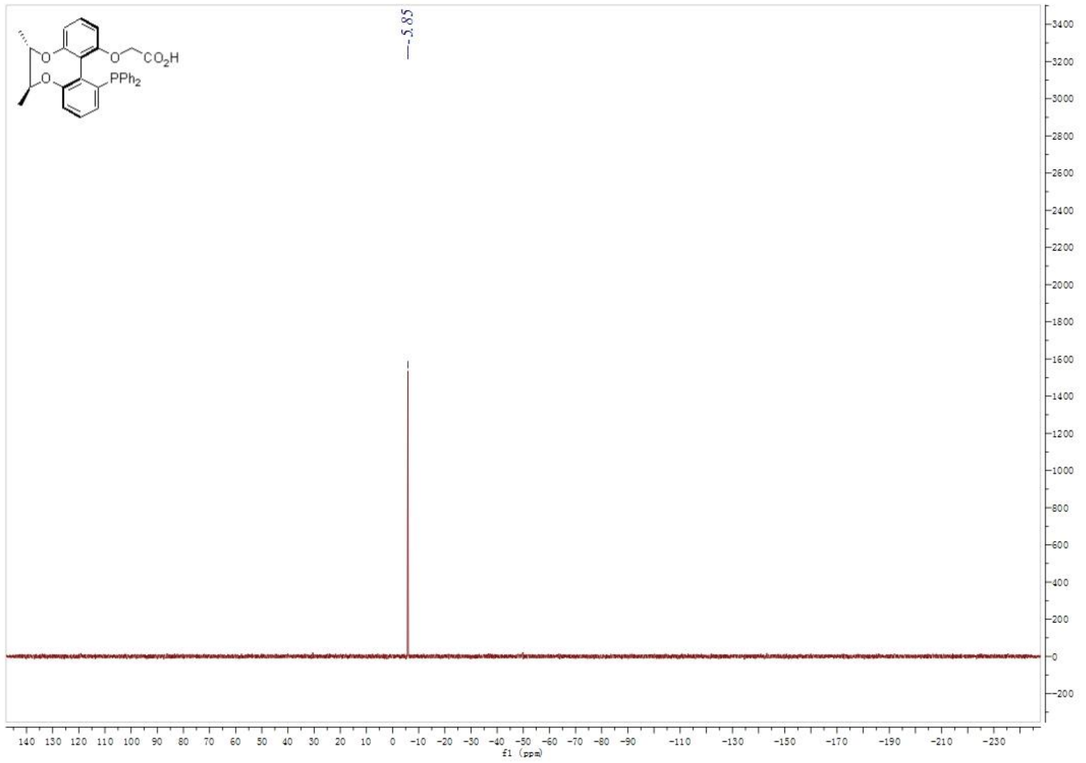

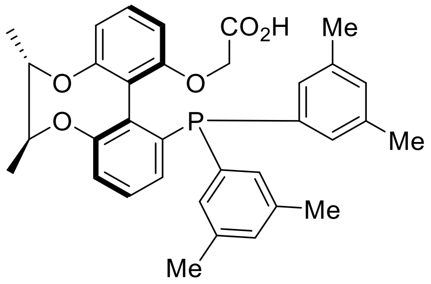

L7

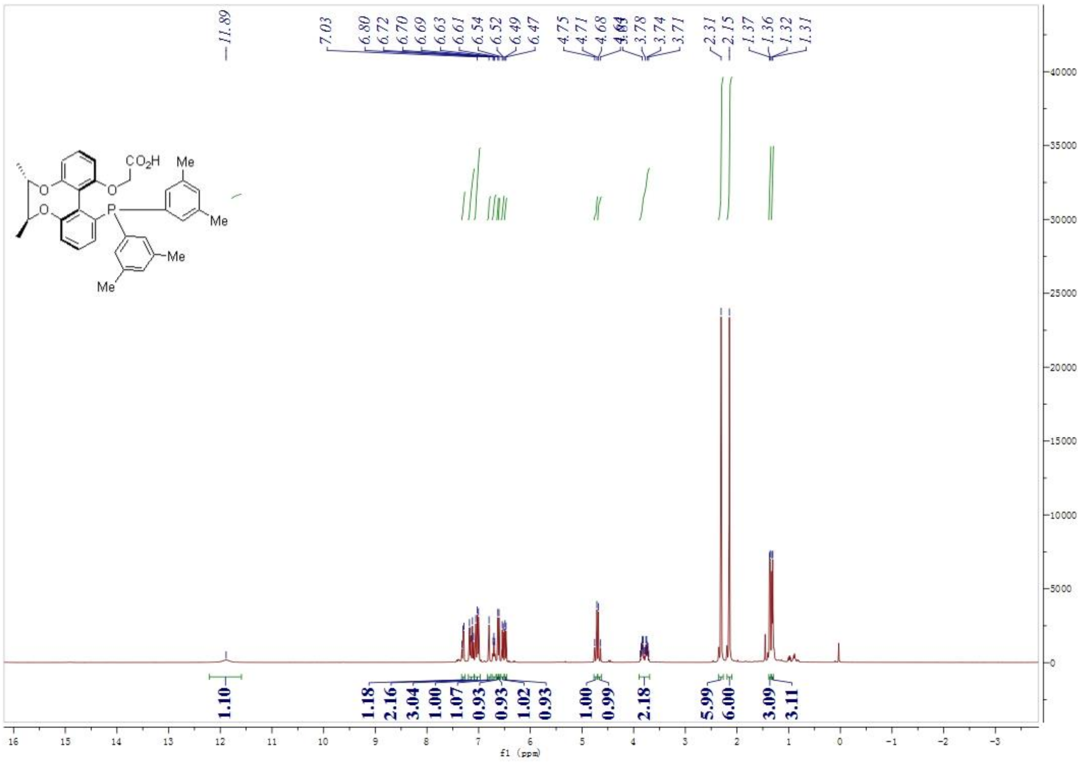

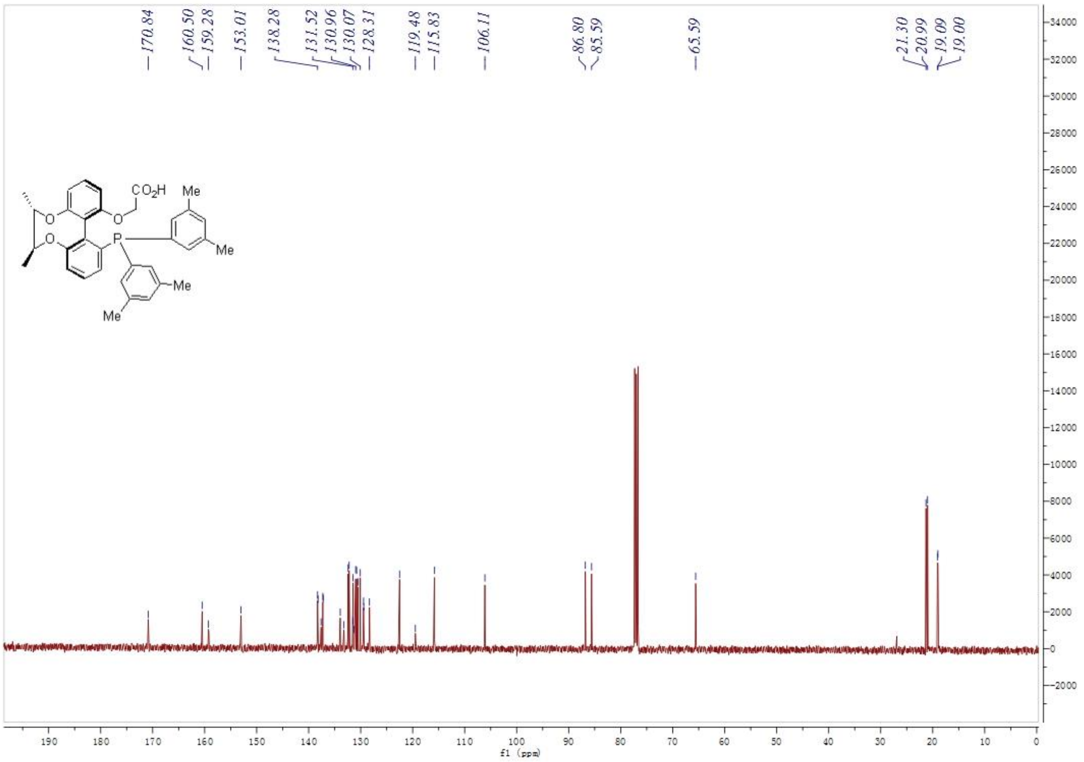

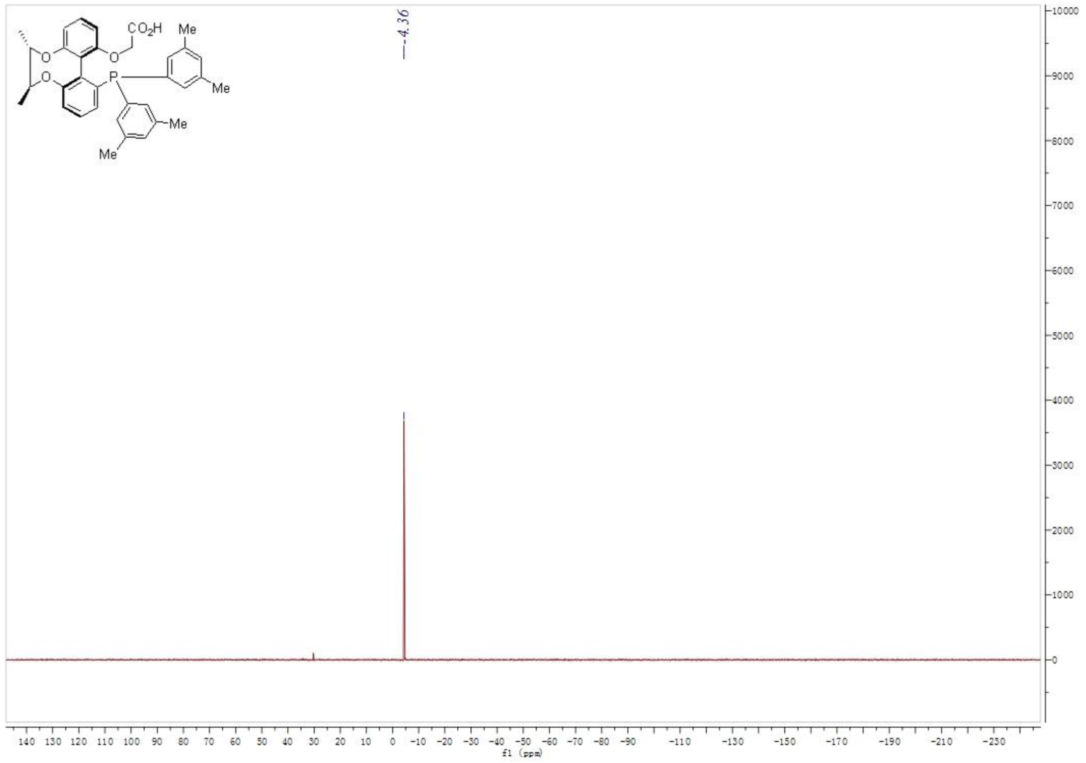

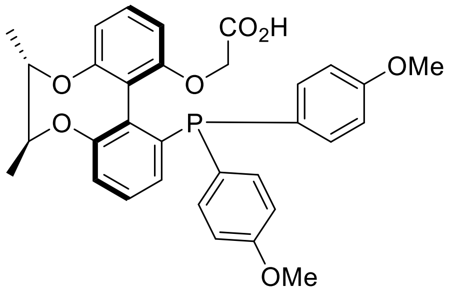

L8

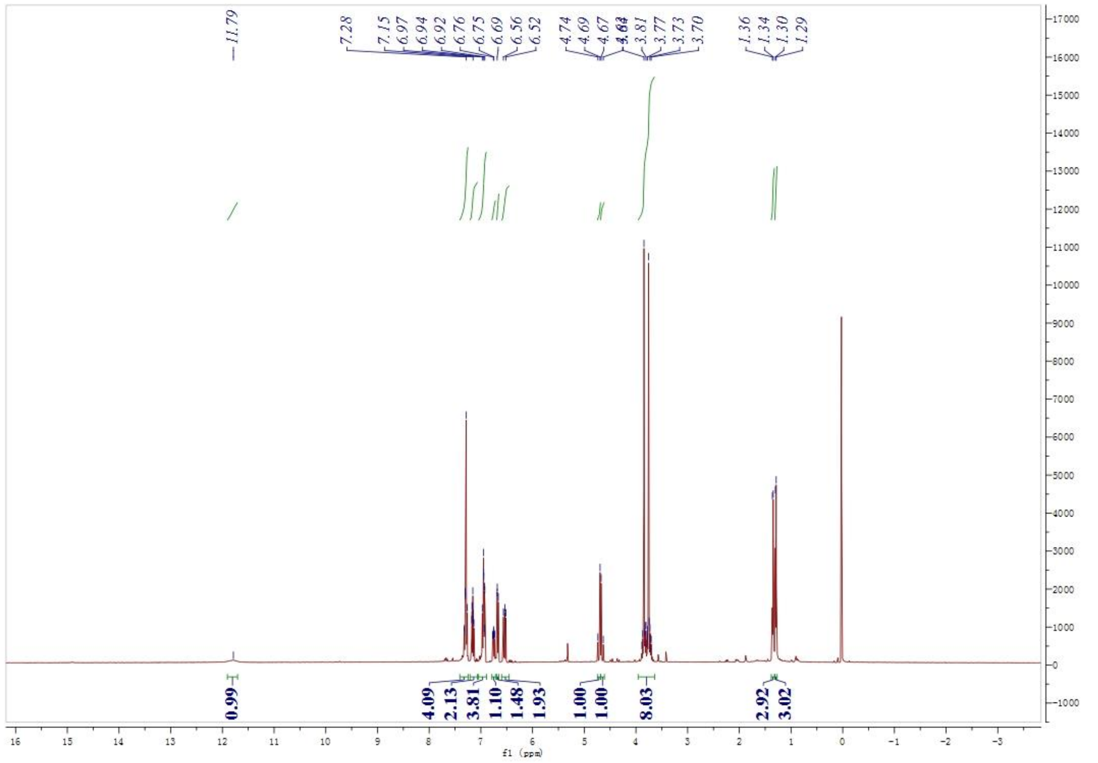

11.79
7.28
7.15
6.97
6.94
6.92
6.76
6.75
6.69
6.56
6.52
4.74
4.69
4.67
3.81
3.77
3.73
3.70
1.36
1.34
1.30
1.29
0.99
4.09
2.13
3.81
1.10
1.48
1.93
1.00
1.00
8.03
2.92
3.02
f1 (ppm)

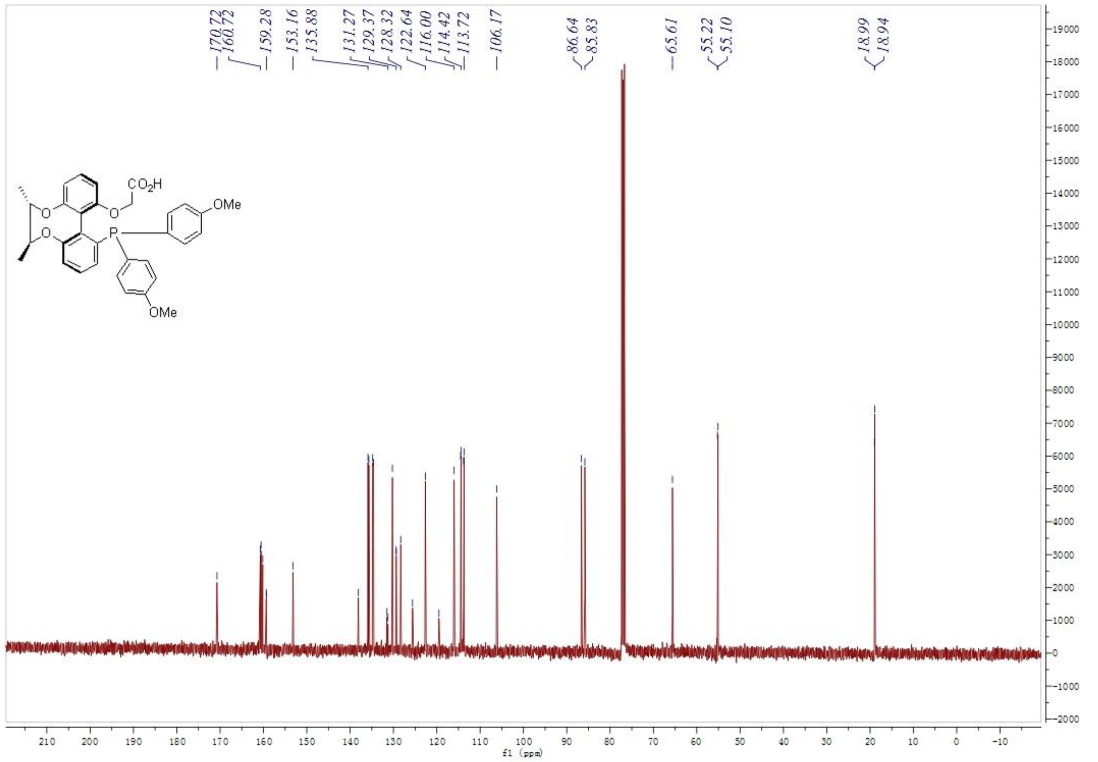

170.72
160.72
159.28
153.16
135.88
131.27
129.37
128.32
122.64
116.00
114.42
113.72
106.17
86.64
85.83
65.61
55.22
55.10
18.99
18.94
CO_2H
OMe
O
O
O
P
OMe
f1 (ppm)

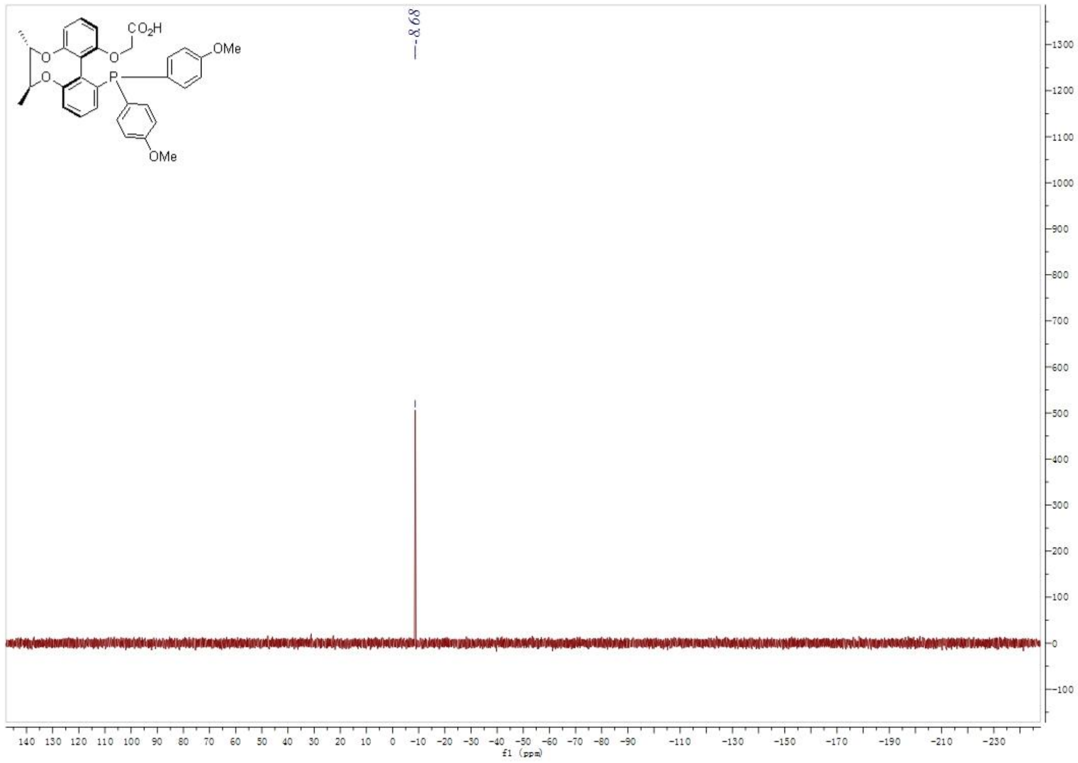

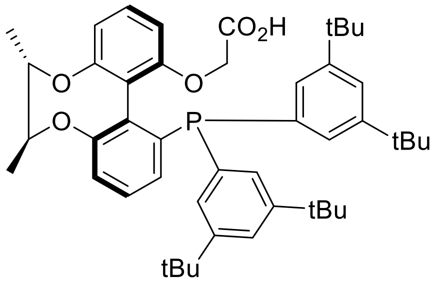

L9

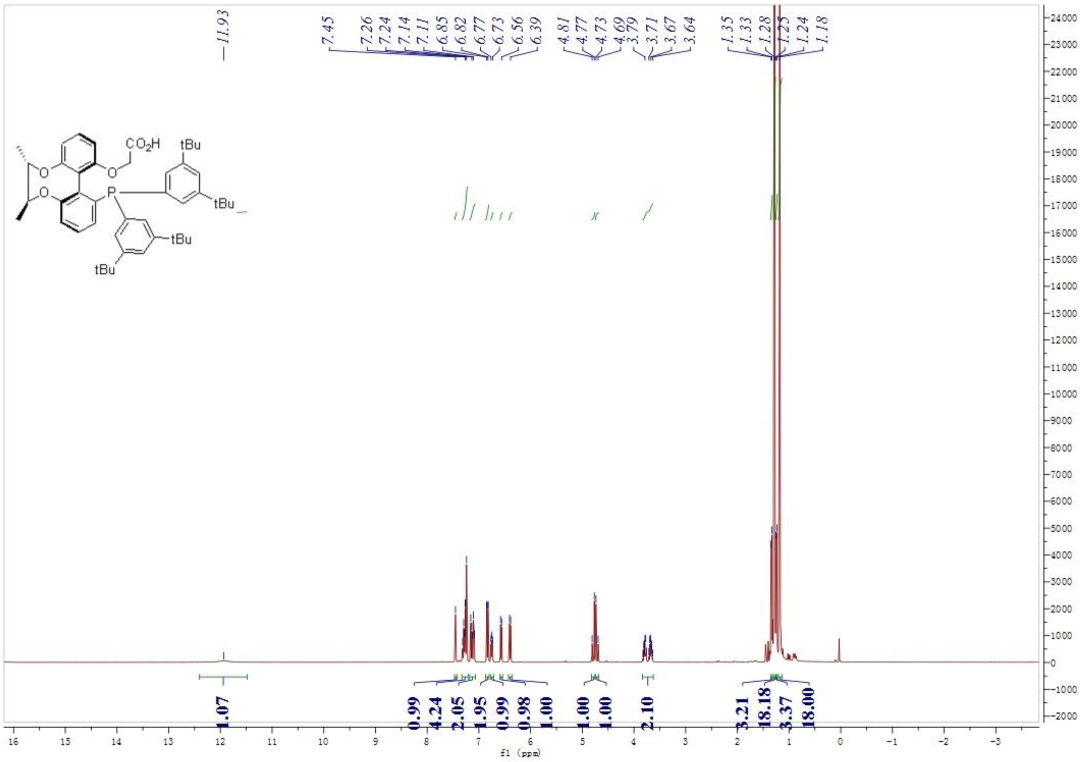
11.93
7.45
7.26
7.24
7.14
7.11
6.85
6.82
6.77
6.73
6.56
6.39
4.81
4.77
4.73
4.69
3.79
3.71
3.67
3.64
1.35
1.33
1.28
1.25
1.24
1.18
CO2H
tBu
tBu
tBu
tBu
P
O
O
O
1.07
0.99
4.24
2.05
1.95
0.99
0.98
1.00
1.00
1.00
2.10
3.21
18.18
3.37
18.00
f1 (ppm)

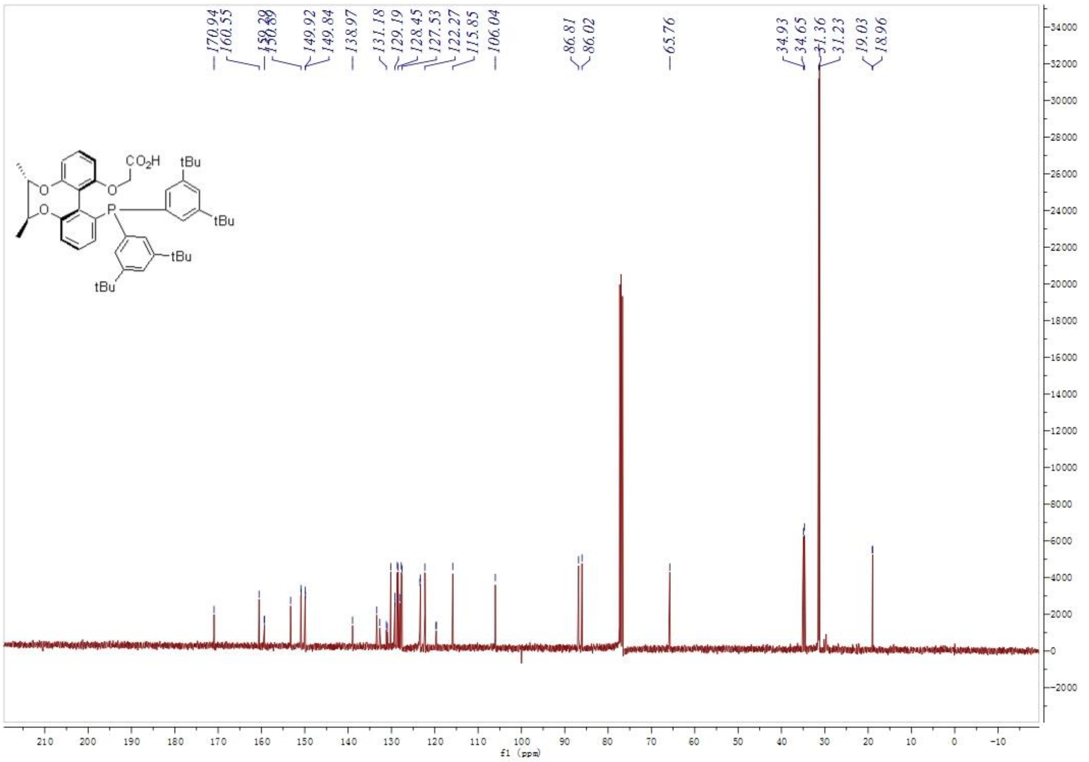
170.94
160.55
149.92
149.84
138.97
131.18
129.19
128.45
127.53
122.27
115.85
106.04
86.81
86.02
65.76
34.93
34.65
31.36
31.23
19.03
18.96
CO2H
tBu
tBu
tBu
tBu
P
O
O
O
f1 (ppm)

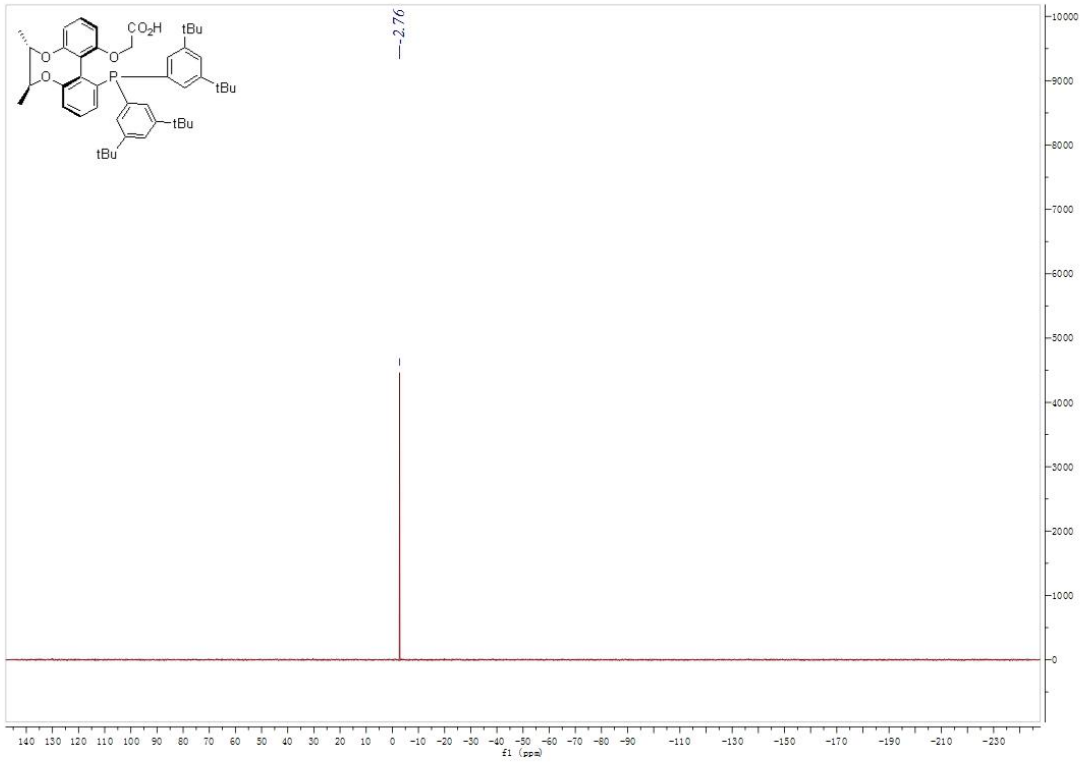

-2.76
CO2H
tBu
tBu
tBu
tBu
O
O
O
P
10000
9000
8000
7000
6000
5000
4000
3000
2000
1000
0
140 130 120 110 100 90 80 70 60 50 40 30 20 10 0 -10 -20 -30 -40 -50 -60 -70 -80 -90 -110 -130 -150 -170 -190 -210 -230
f1 (ppm)

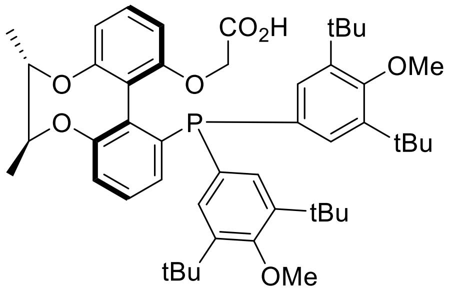

CO2H
tBu
OMe
tBu
O
O
O
P
tBu
tBu
OMe

L10

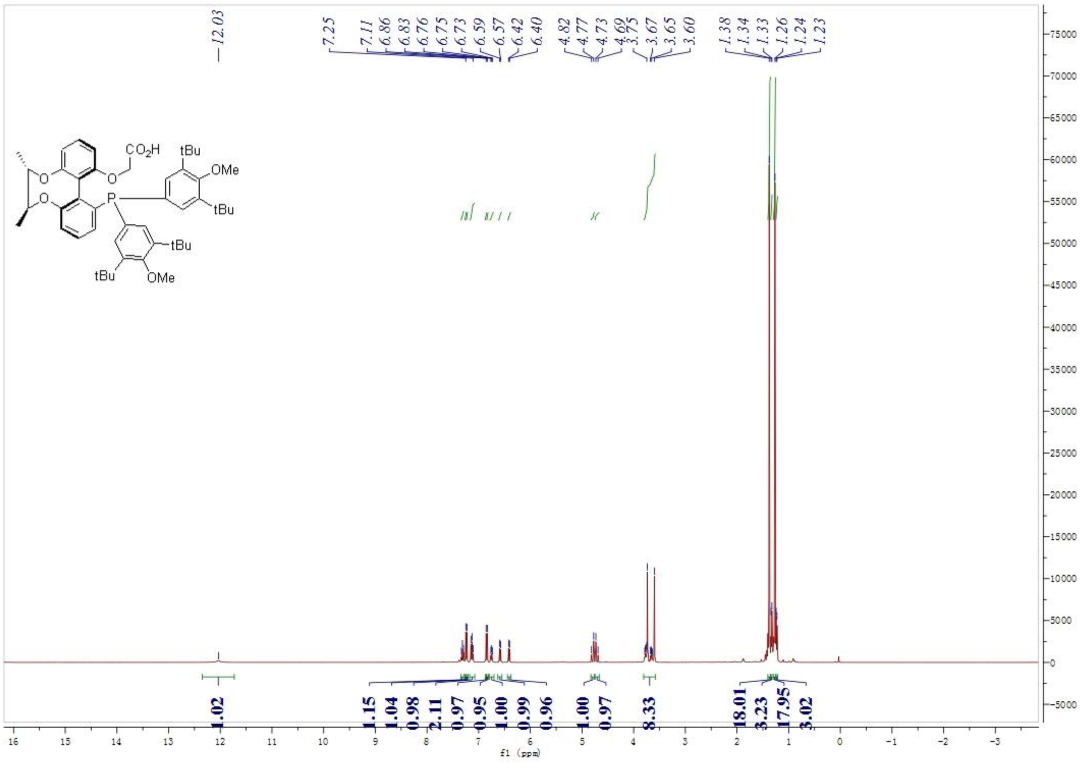
12.03
7.25 7.11 6.86 6.83 6.76 6.75 6.73 6.59 6.57 6.42 6.40
4.82 4.77 4.73 4.69 3.75 3.67 3.65 3.60
1.38 1.34 1.33 1.26 1.24 1.23
CO2H
tBu
OMe
tBu
P
O
O
O
tBu
tBu
OMe
1.02
1.15 1.04 0.98 2.11 0.97 0.95 1.00 0.99 0.96
1.00 0.97
8.33
18.01 3.23 17.95 3.02
f1 (ppm)

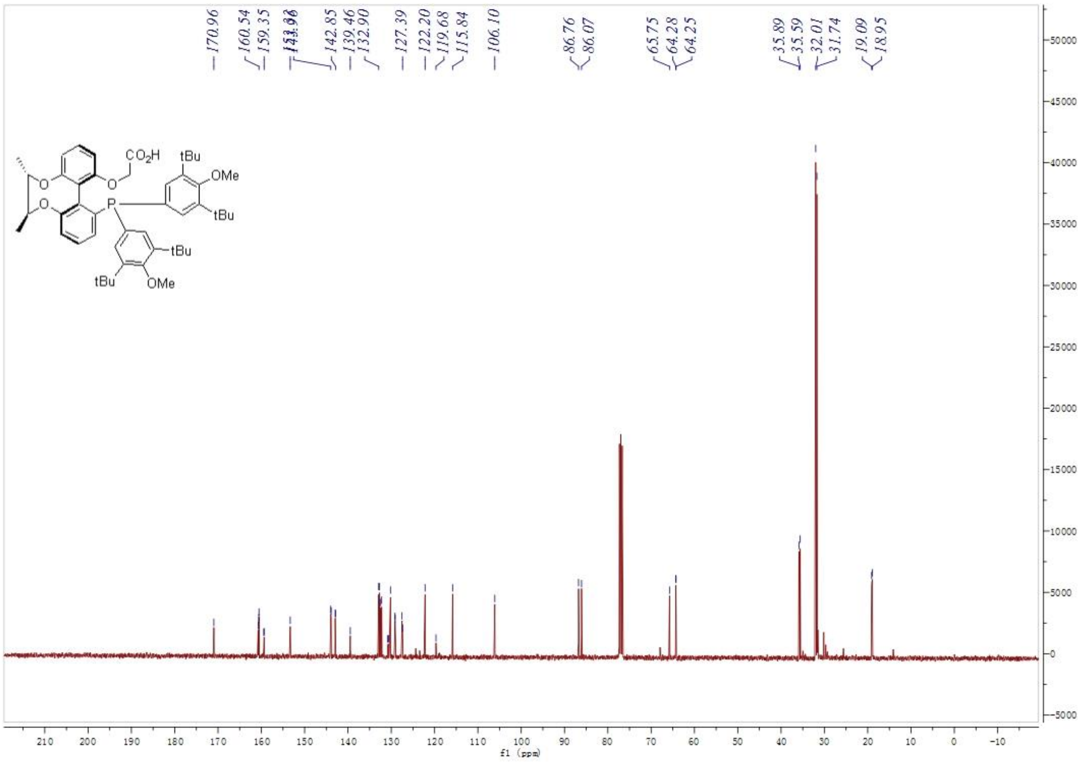
170.96
160.54 159.35
142.85 139.46 132.90
127.39 122.20 119.68 115.84
106.10
86.76 86.07
65.75 64.28 64.25
35.89 35.59 32.01 31.74
19.09 18.95
CO2H
tBu
OMe
tBu
P
O
O
O
tBu
tBu
OMe
f1 (ppm)

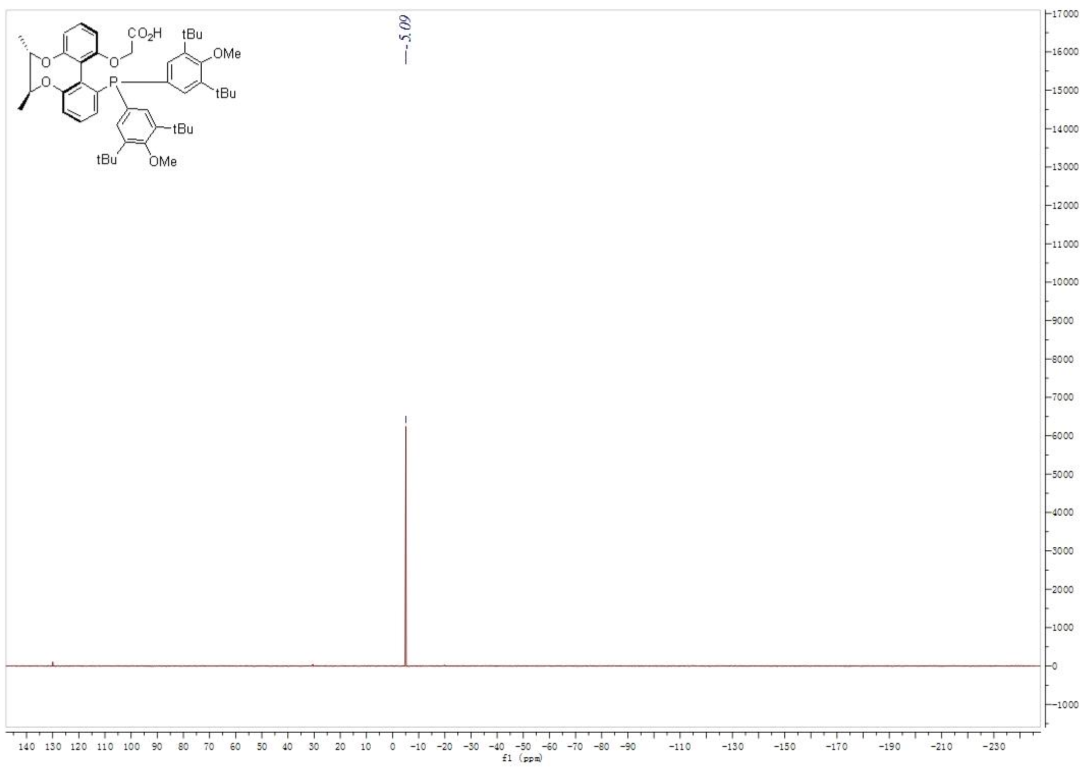

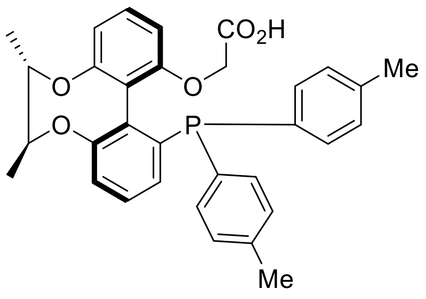

L11

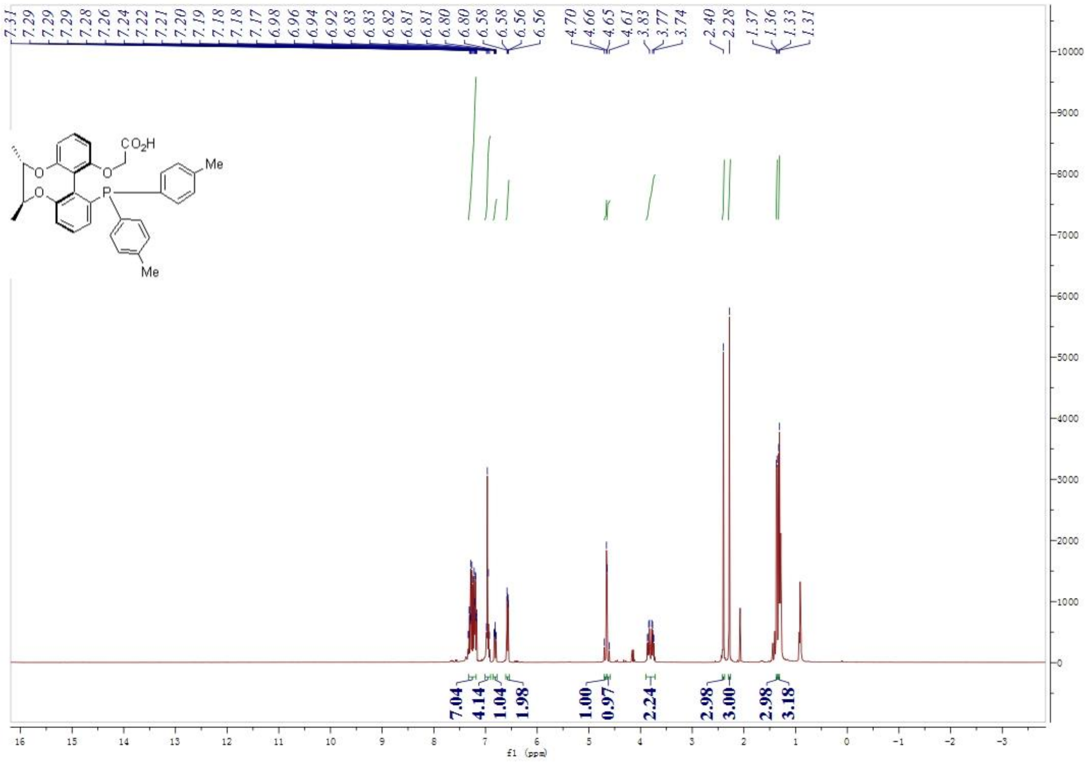

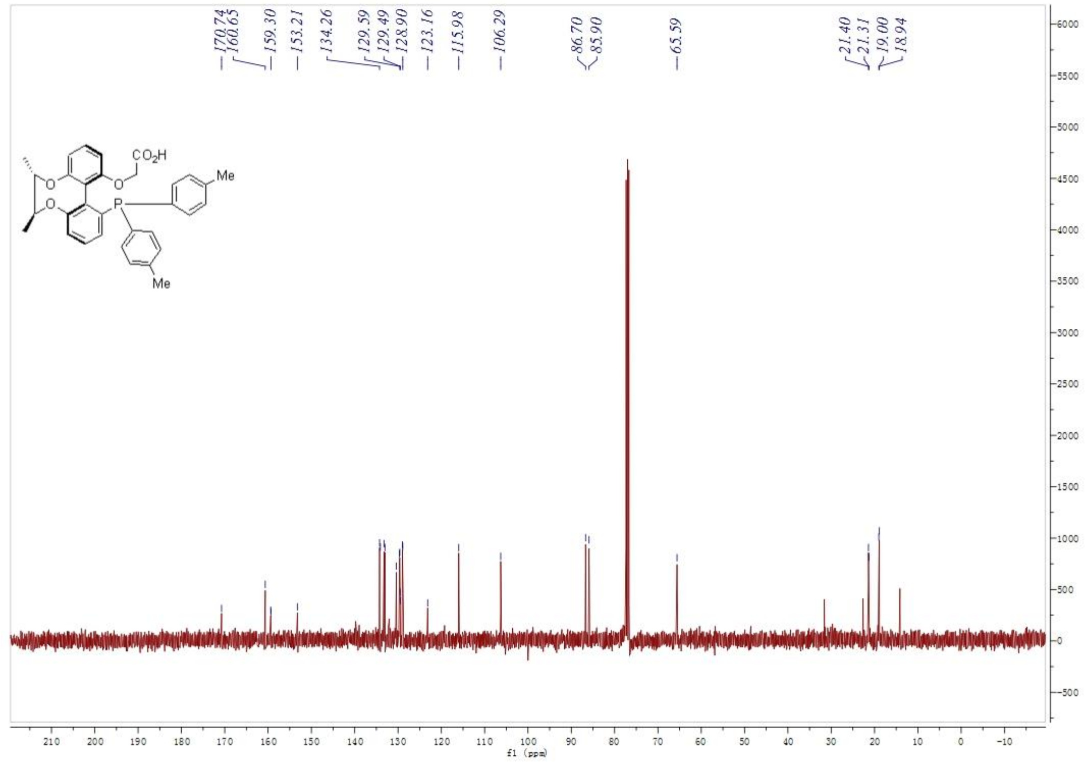

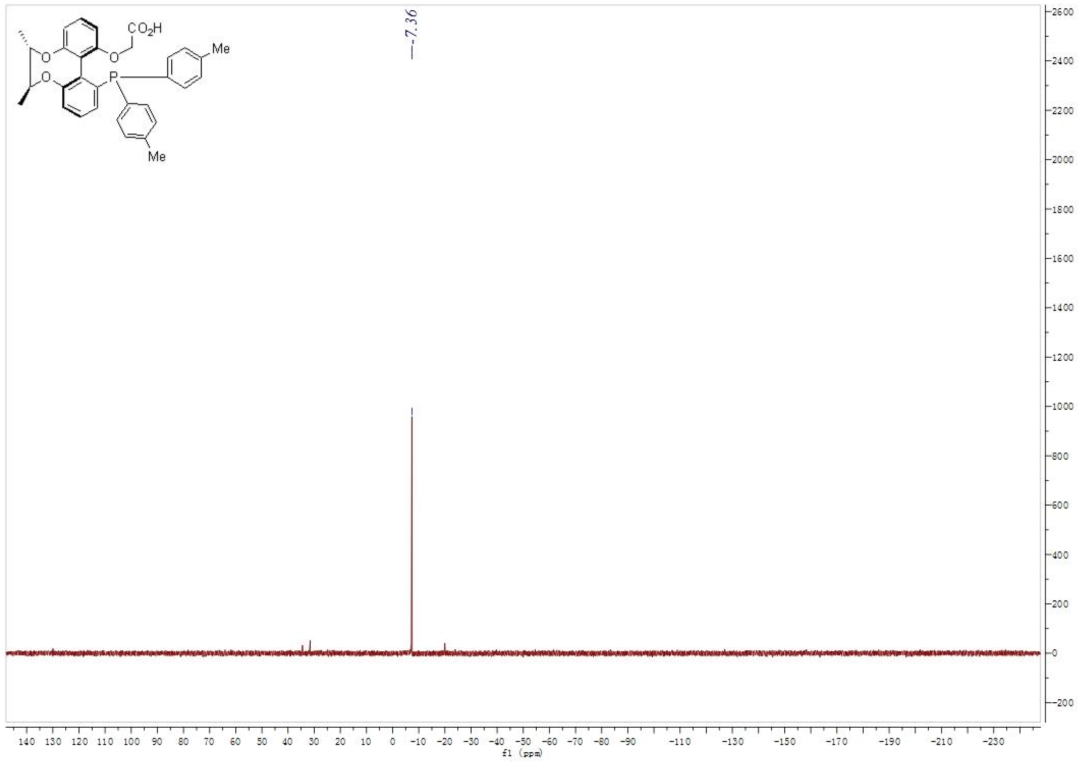

CO2H
O
O
O
P
Me
Me
—7.36
2600
2400
2200
2000
1800
1600
1400
1200
1000
800
600
400
200
0
−200
140 130 120 110 100 90 80 70 60 50 40 30 20 10 0 −10 −20 −30 −40 −50 −60 −70 −80 −90 −110 −130 −150 −170 −190 −210 −230
f1 (ppm)

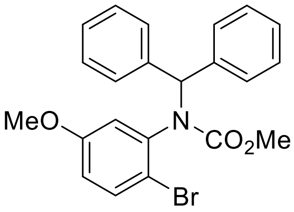

MeO
N
CO2Me
Br

1c

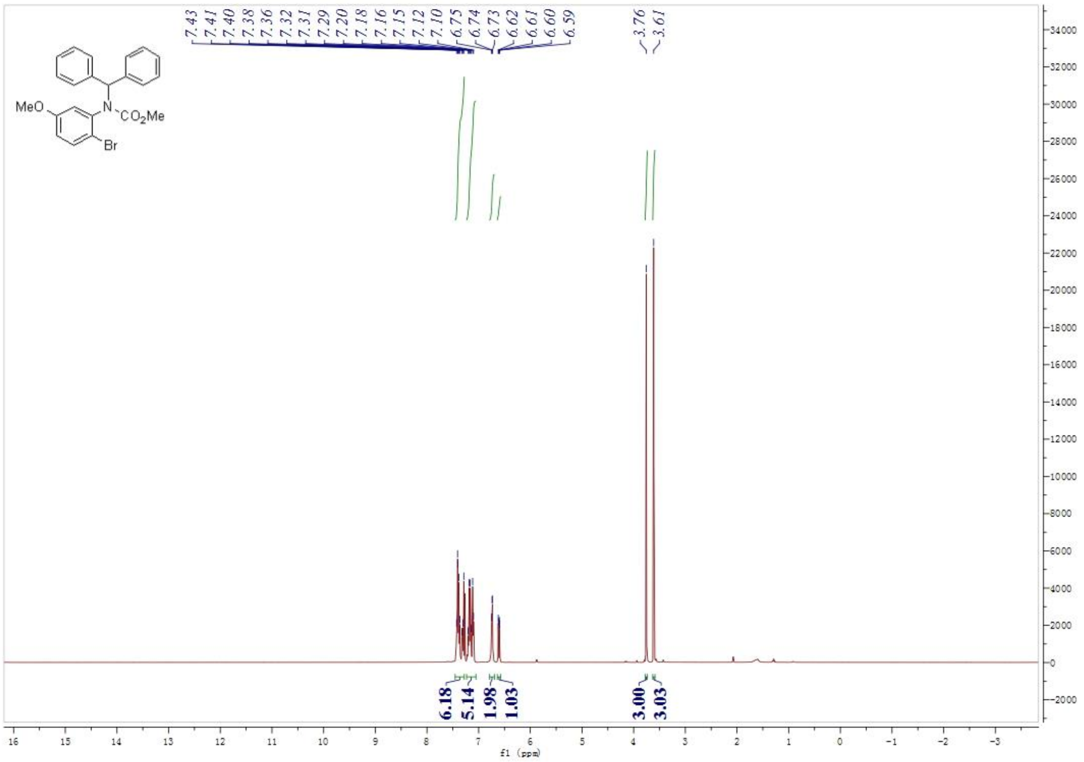
7.43 7.41 7.40 7.38 7.36 7.32 7.31 7.29 7.20 7.18 7.16 7.15 7.12 7.10 6.75 6.74 6.73 6.62 6.61 6.60 6.59
3.76 3.61
MeO
N
CO2Me
Br
6.18 5.14 1.98 1.03
3.00 3.03
f1 (ppm)

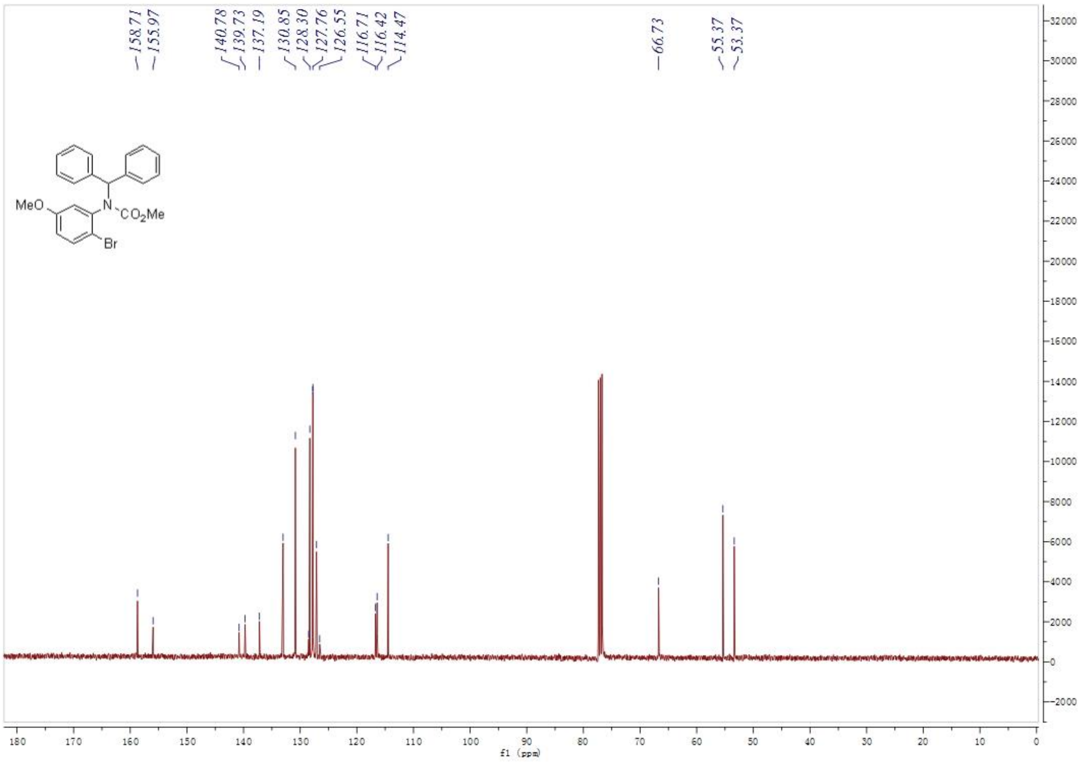
158.71 155.97
140.78 139.73 137.19 130.85 128.30 127.76 126.55 116.71 116.42 114.47
66.73
55.37 53.37
MeO
N
CO2Me
Br
f1 (ppm)

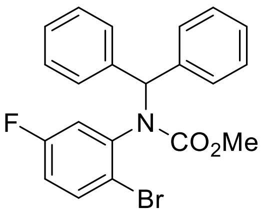
F
N
CO2Me
Br

1d

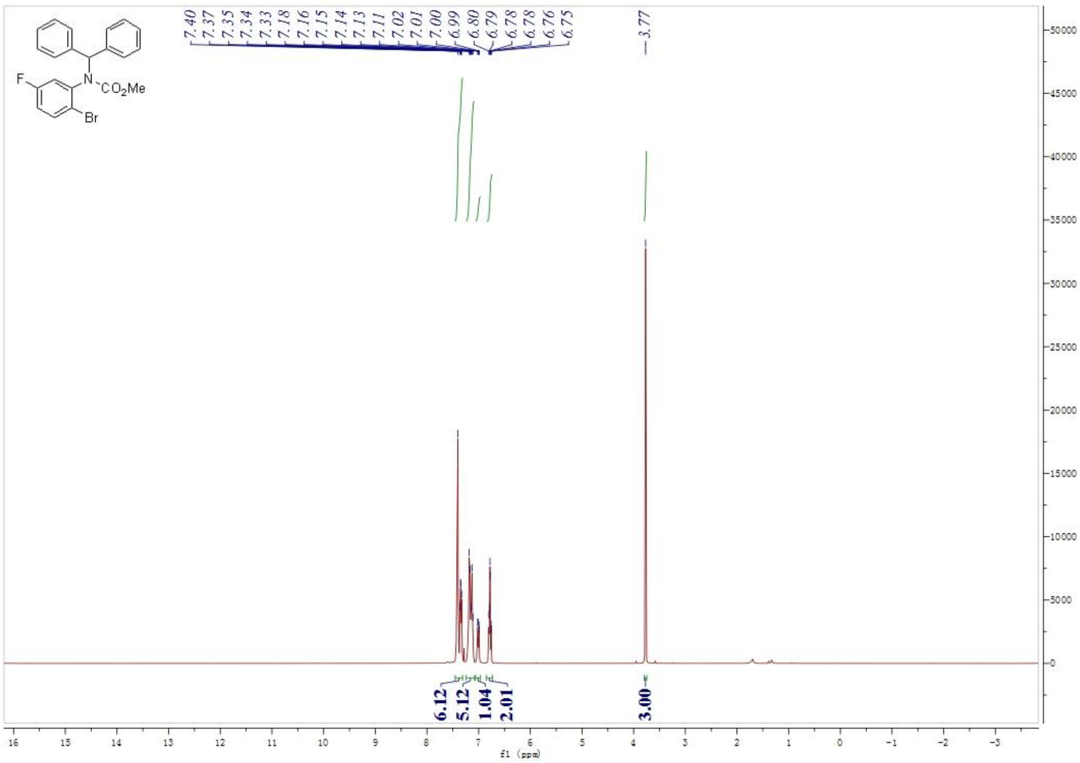
7.40
7.37
7.35
7.34
7.33
7.18
7.16
7.15
7.14
7.13
7.11
7.02
7.01
7.00
6.99
6.80
6.79
6.78
6.76
6.75
3.77
6.12
5.12
1.04
2.01
3.00
f1 (ppm)

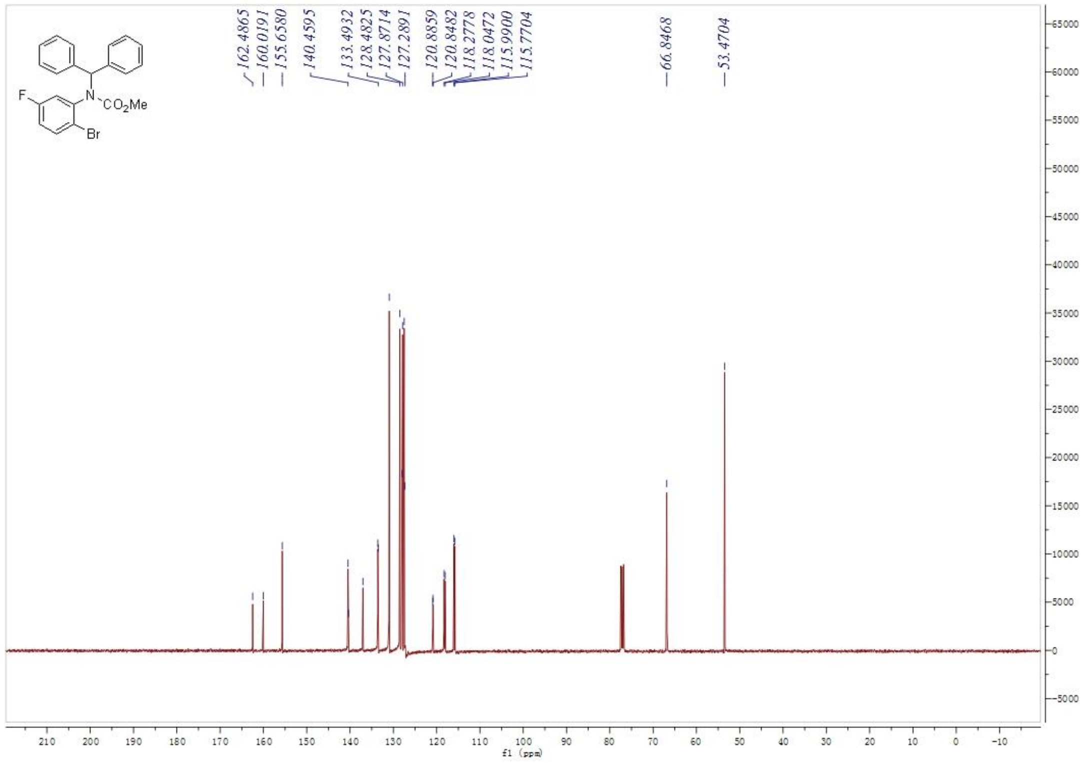

162.4865
160.0191
155.6580
140.4595
133.4932
128.4825
127.8714
127.2891
120.8859
120.8482
118.2778
118.0472
115.9900
115.7704
66.8468
53.4704
CO2Me
F
Br
N
f1 (ppm)

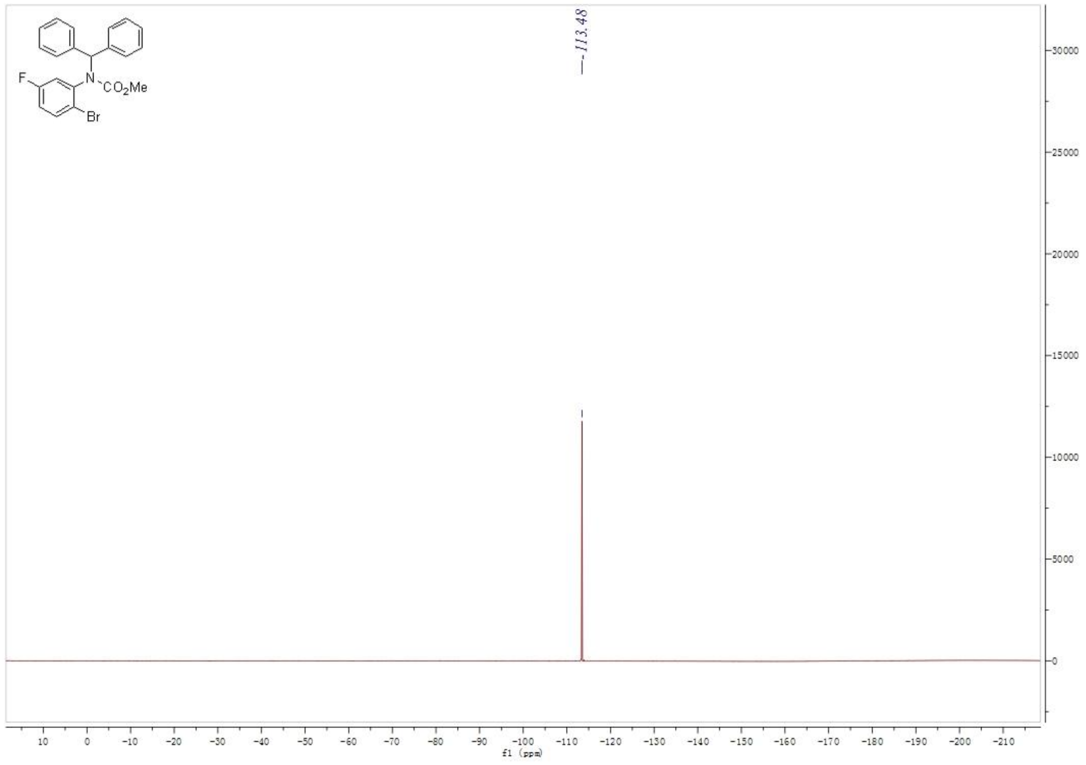

-113.48
CO2Me
F
Br
N
f1 (ppm)

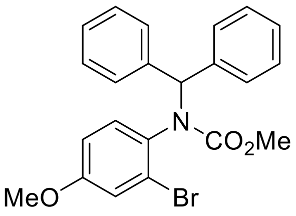

N
CO2Me
MeO
Br

1f

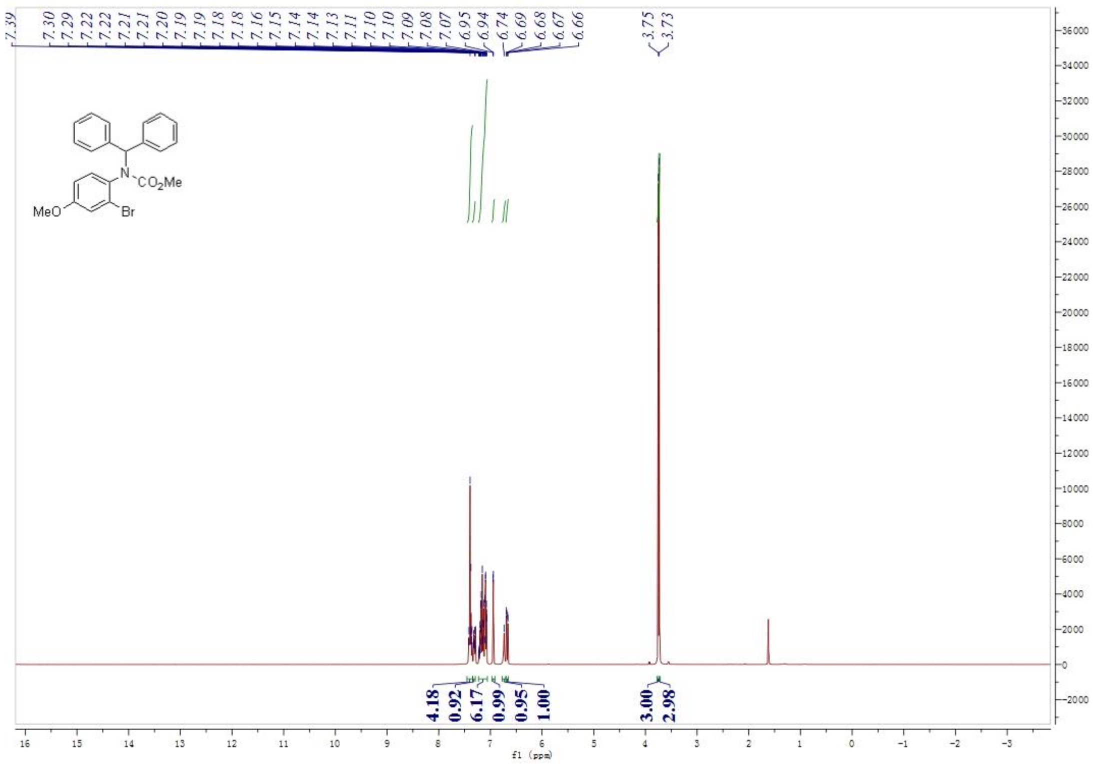

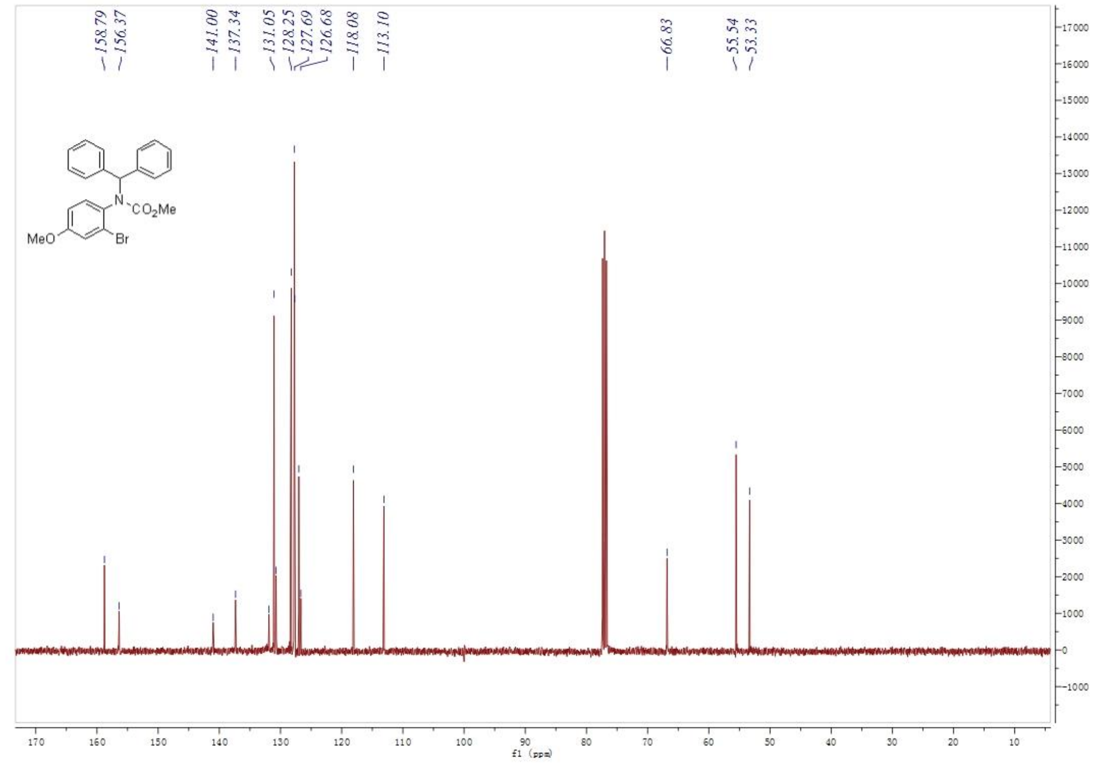

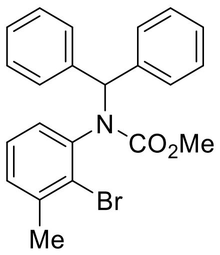

1j

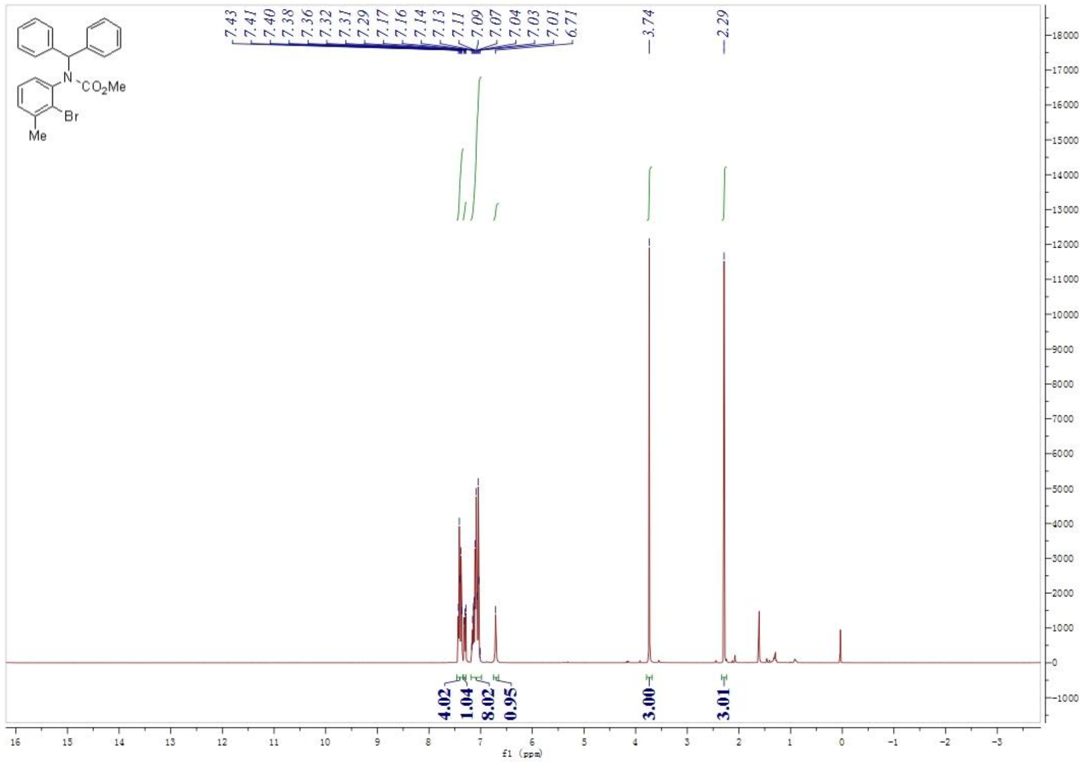

7.43
7.41
7.40
7.38
7.36
7.32
7.31
7.29
7.17
7.16
7.14
7.13
7.11
7.09
7.07
7.04
7.03
7.01
6.71
3.74
2.29
4.02
1.04
8.02
0.95
3.00
3.01
f1 (ppm)

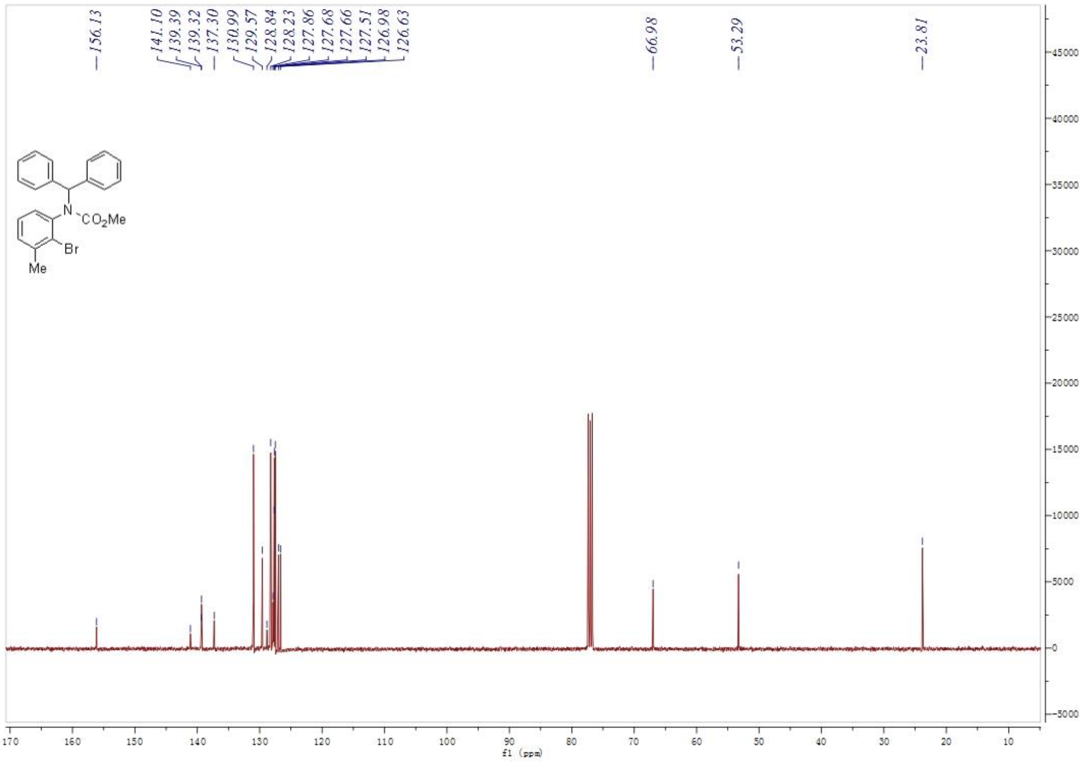
156.13
141.10
139.39
139.32
137.30
130.99
129.57
128.84
128.23
127.86
127.68
127.66
127.51
126.98
126.63
66.98
53.29
23.81
CO2Me
Br
Me
f1 (ppm)

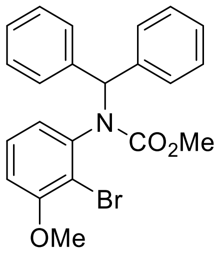
N
CO2Me
Br
OMe

1k

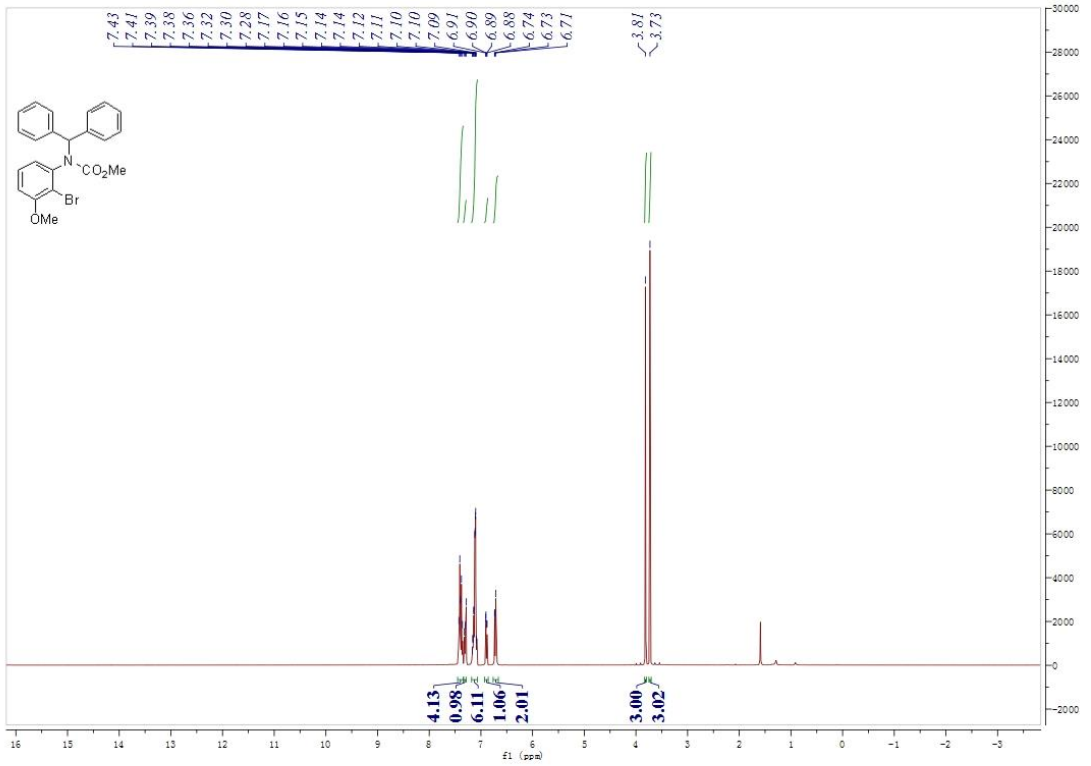
7.43
7.41
7.39
7.38
7.36
7.32
7.30
7.28
7.17
7.16
7.15
7.14
7.14
7.12
7.11
7.10
7.10
7.09
6.91
6.90
6.89
6.88
6.74
6.73
6.71
3.81
3.73
CO2Me
Br
OMe
4.13
0.98
6.11
1.06
2.01
3.00
3.02
f1 (ppm)

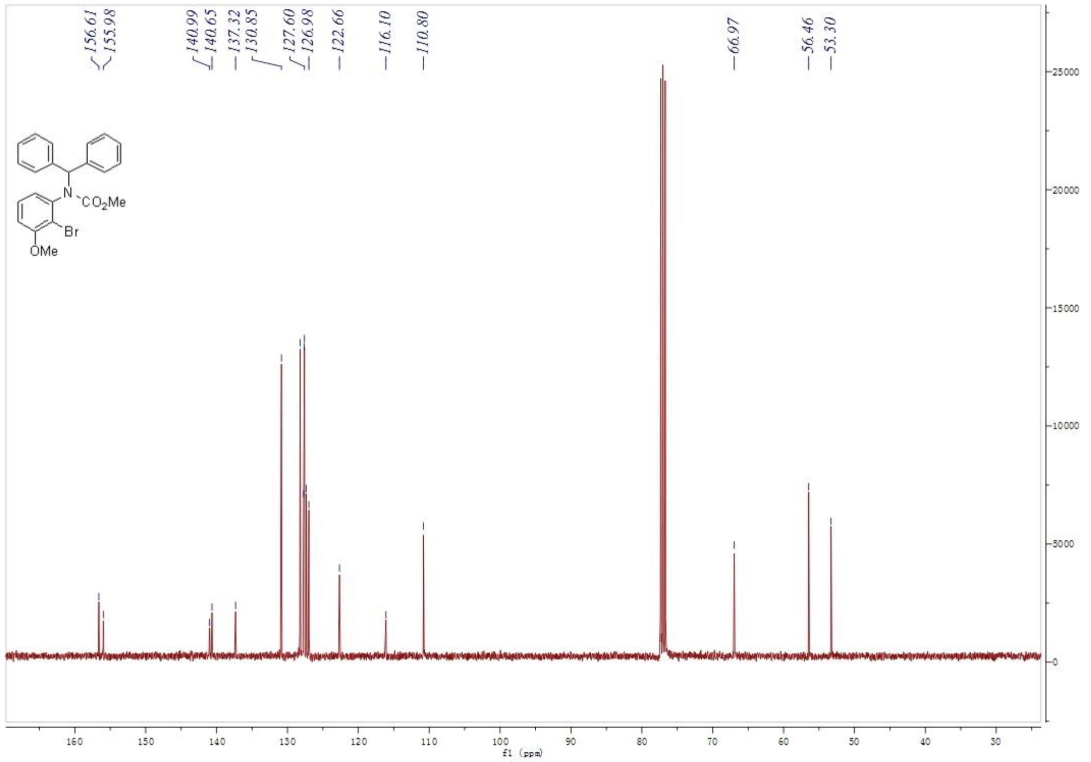
156.61
155.98
140.99
140.65
137.32
130.85
127.60
126.98
122.66
116.10
110.80
66.97
56.46
53.30
CO2Me
Br
OMe
f1 (ppm)

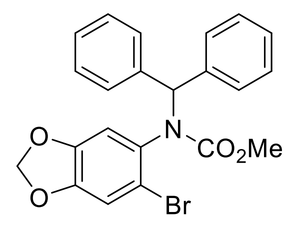
N
CO2Me
O
O
Br

1m

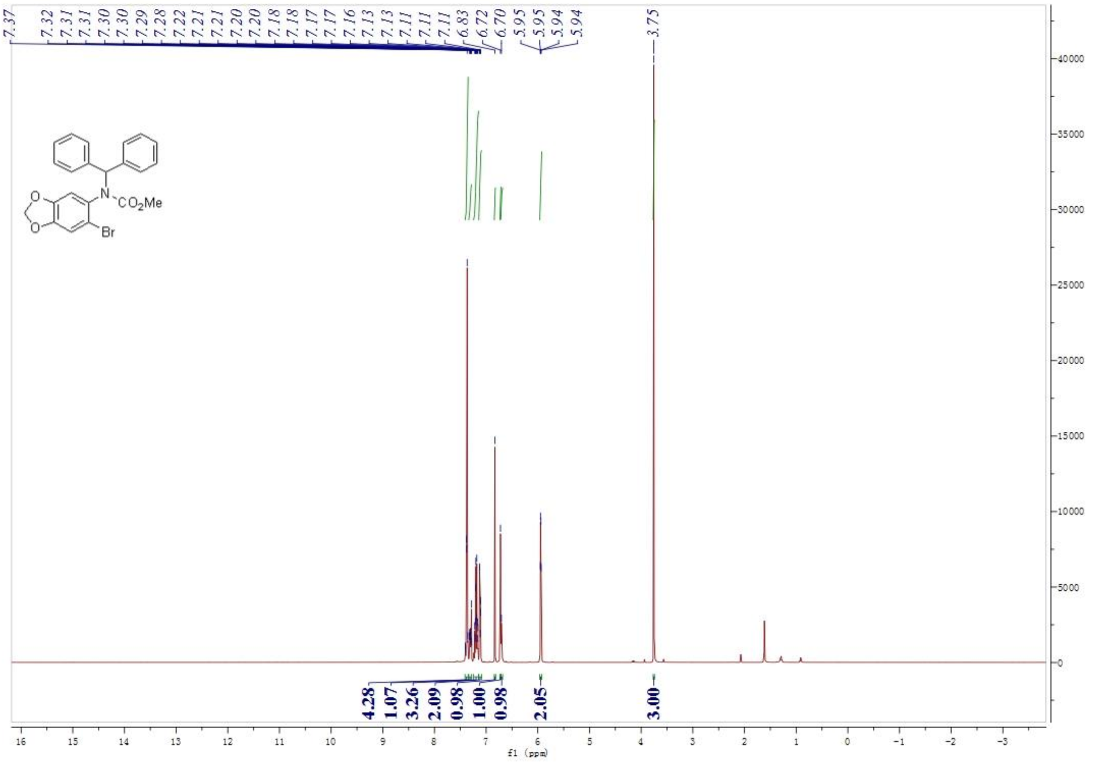

7.37 7.32 7.31 7.31 7.30 7.30 7.29 7.28 7.22 7.21 7.21 7.20 7.20 7.18 7.18 7.17 7.17 7.16 7.13 7.13 7.11 7.11 7.11 7.11 6.83 6.72 6.70 5.95 5.95 5.94 5.94 3.75
4.28 1.07 3.26 2.09 0.98 1.00 0.98 2.05 3.00
CO2Me
Br
f1 (ppm)

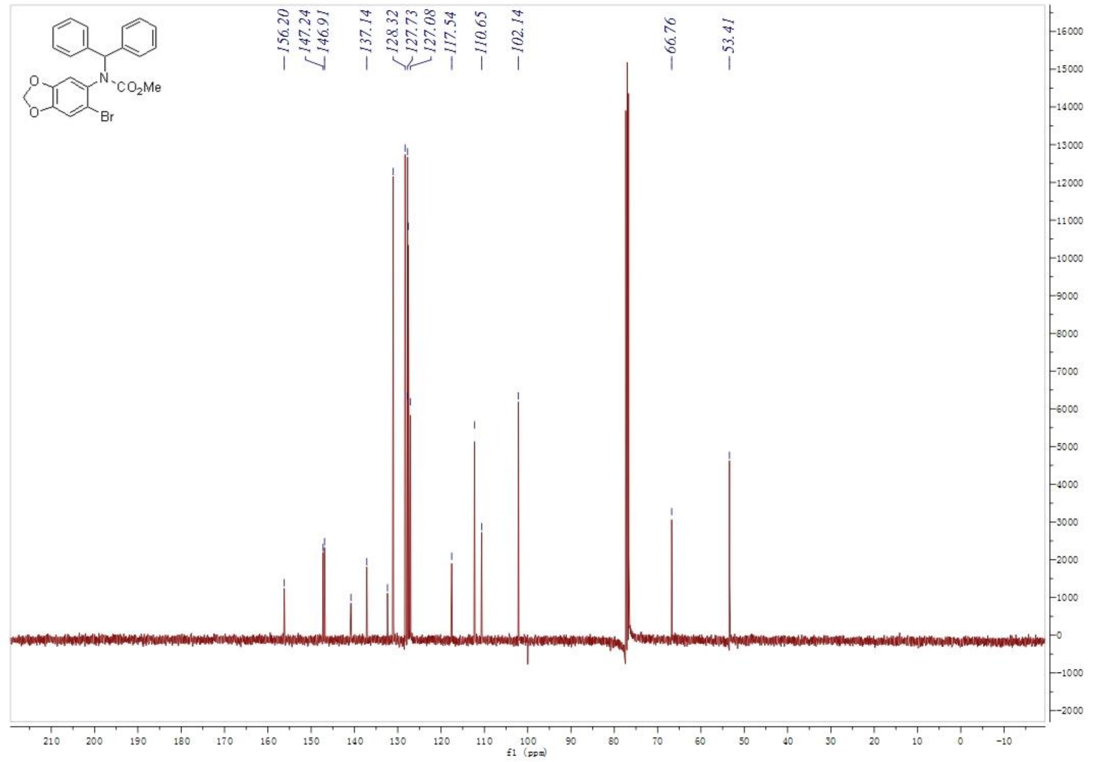

156.20 147.24 146.91 137.14 128.32 127.73 127.08 117.54 110.65 102.14 66.76 53.41
CO2Me
Br
f1 (ppm)

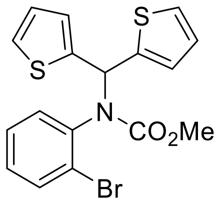

S
S
N
CO2Me
Br

1u

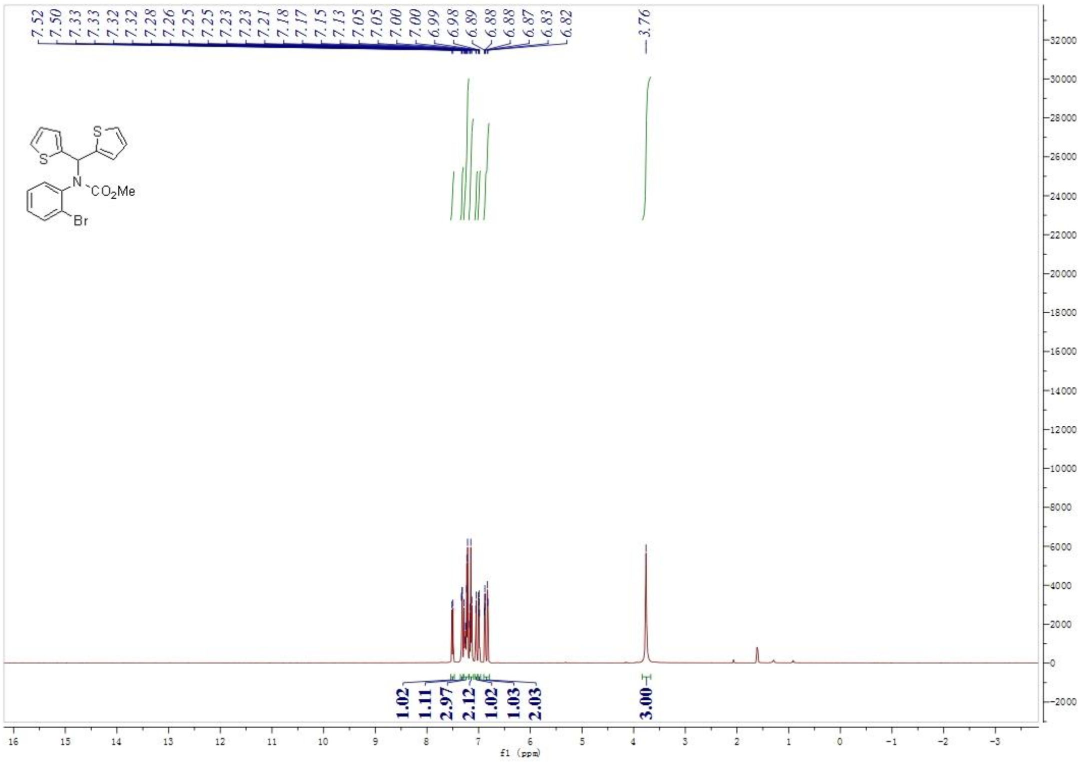

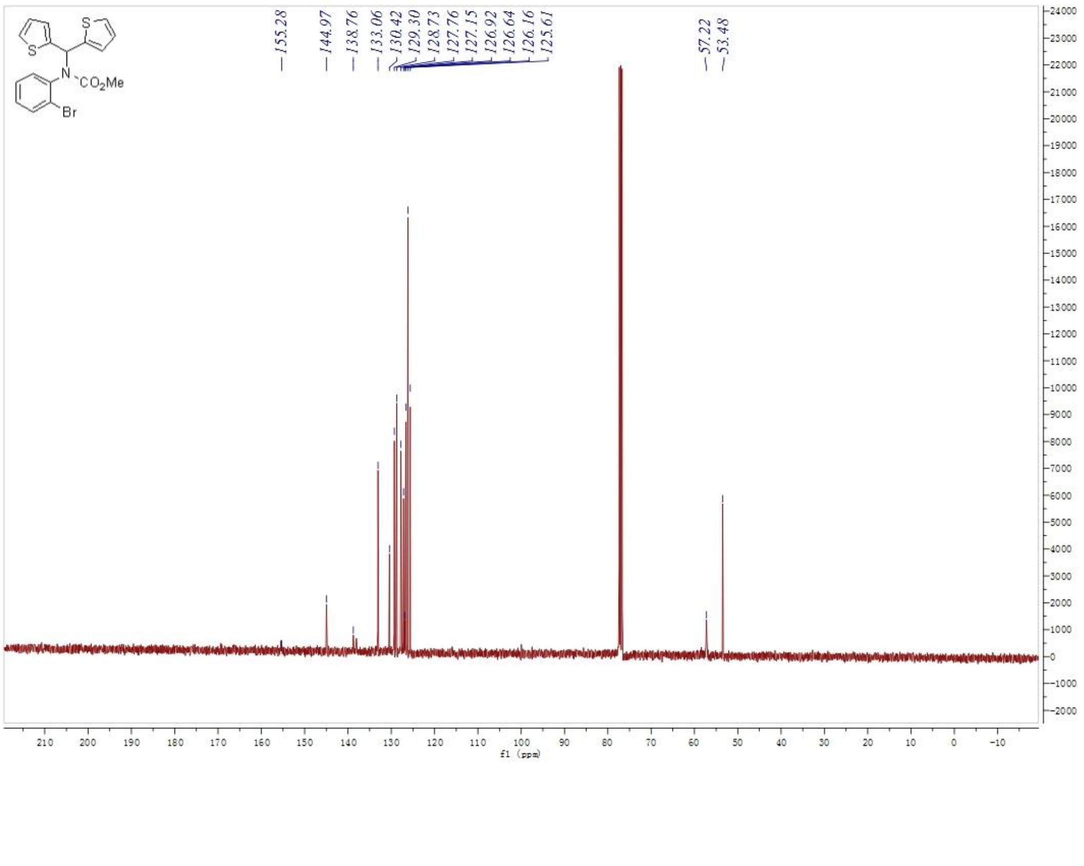

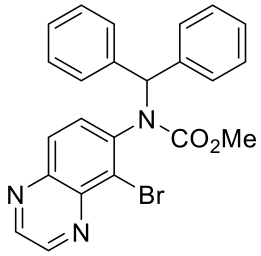
N
CO2Me
N
Br
N

1v

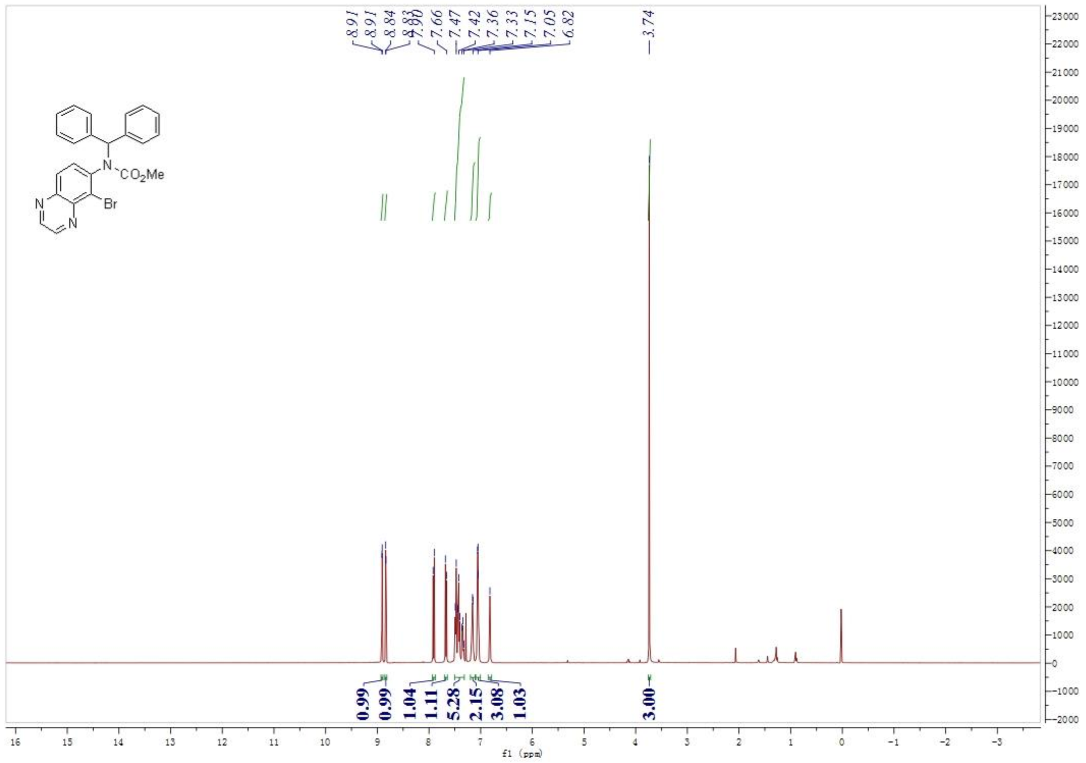
8.91
8.91
8.84
8.83
7.90
7.66
7.47
7.42
7.36
7.33
7.15
7.05
6.82
3.74
CO2Me
Br
0.99
0.99
1.04
1.11
5.28
2.15
3.08
1.03
3.00
f1 (ppm)

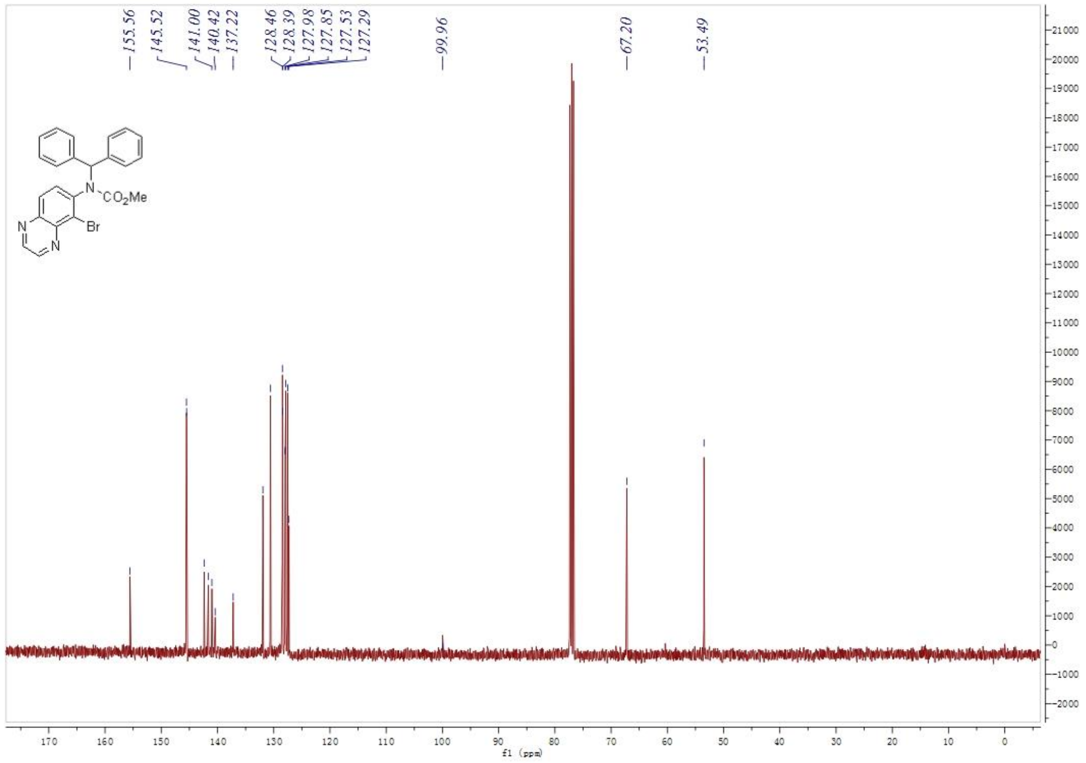
155.56
145.52
141.00
140.42
137.22
128.46
128.39
127.98
127.85
127.53
127.29
99.96
67.20
53.49
CO2Me
Br
N
f1 (ppm)

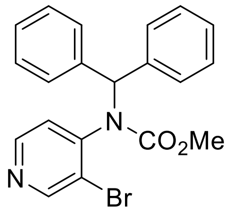
N
CO2Me
N
Br

1x

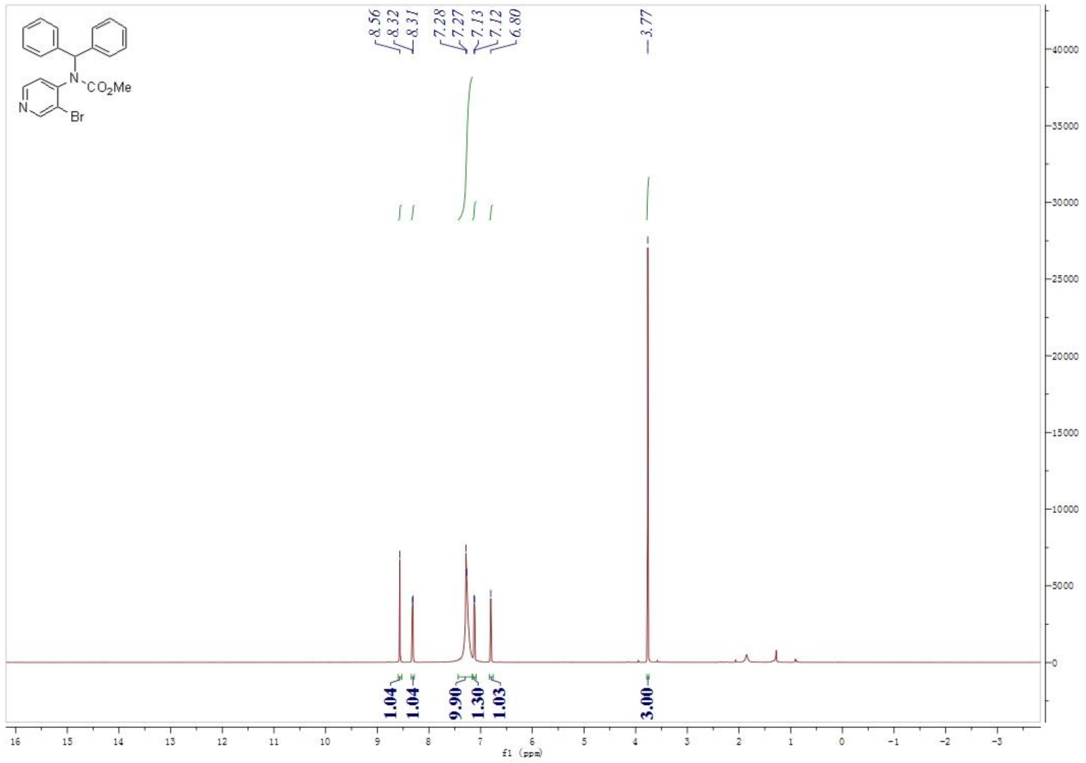
8.56
8.32
8.31
7.28
7.27
7.13
7.12
6.80
3.77
1.04
1.04
9.90
1.30
1.03
3.00
f1 (ppm)

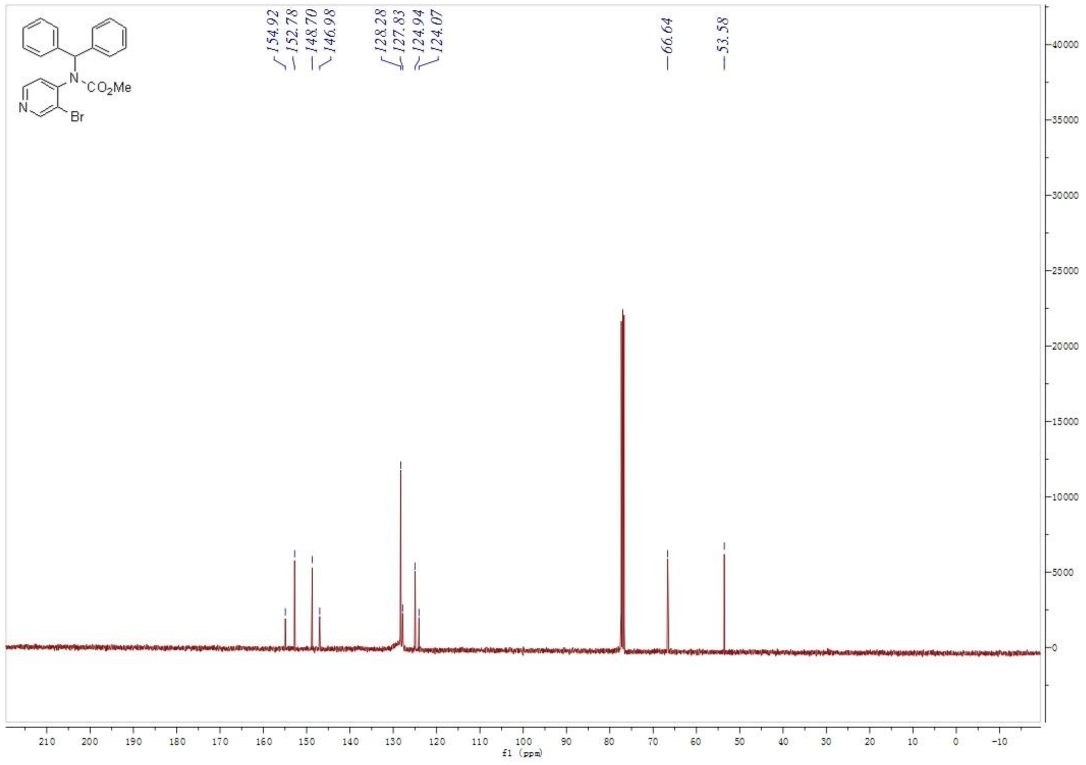
154.92
152.78
148.70
146.98
128.28
127.83
124.94
124.07
66.64
53.58
f1 (ppm)

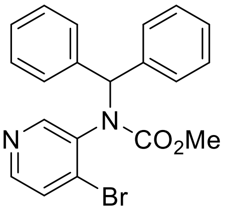
N
N
CO2Me
Br

1y

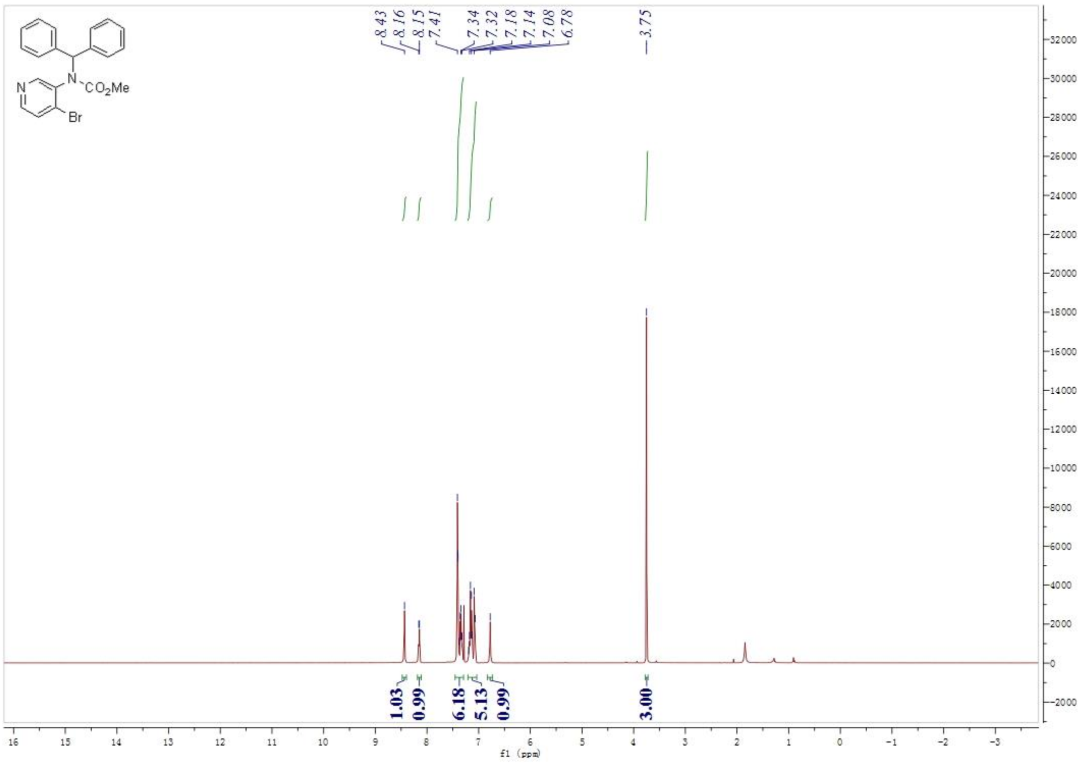
CO2Me
N
N
Br
8.43
8.16
8.15
7.41
7.34
7.32
7.18
7.14
7.08
6.78
3.75
1.03
0.99
6.18
5.13
0.99
3.00
f1 (ppm)

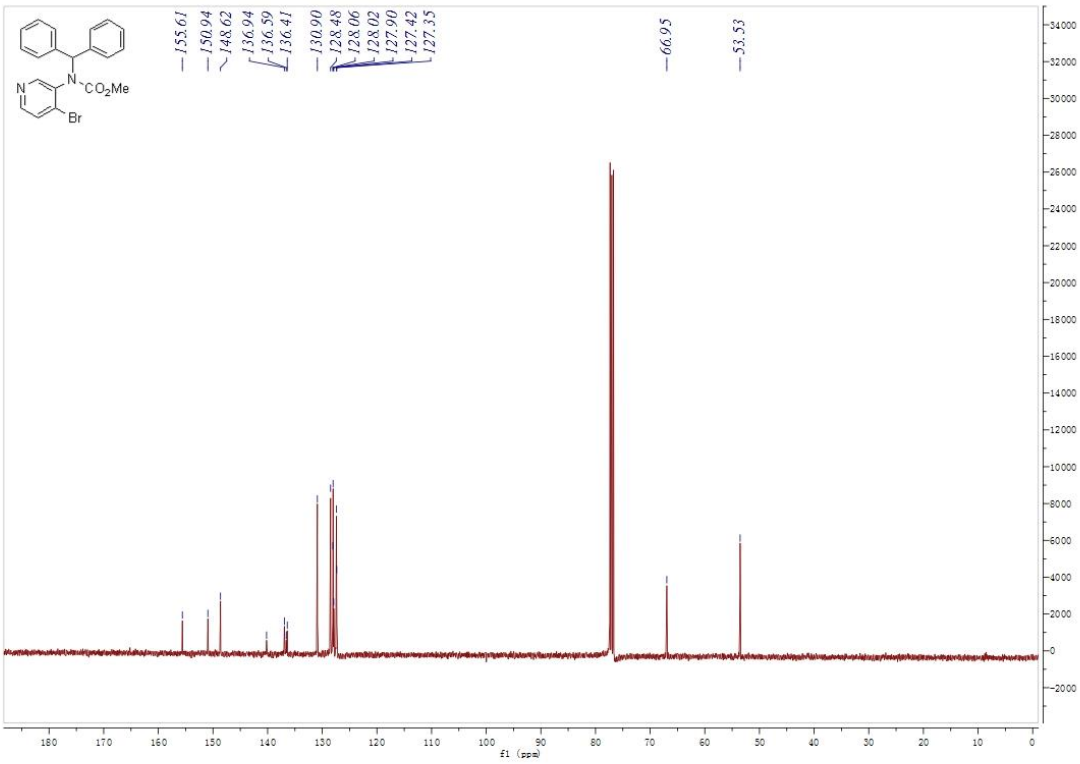
CO2Me
N
N
Br
155.61
150.94
148.62
136.94
136.59
136.41
130.90
128.48
128.06
128.02
127.90
127.42
127.35
66.95
53.53
f1 (ppm)

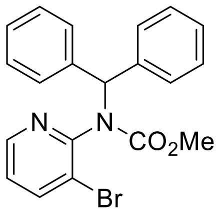
N
N
CO2Me
Br

1z

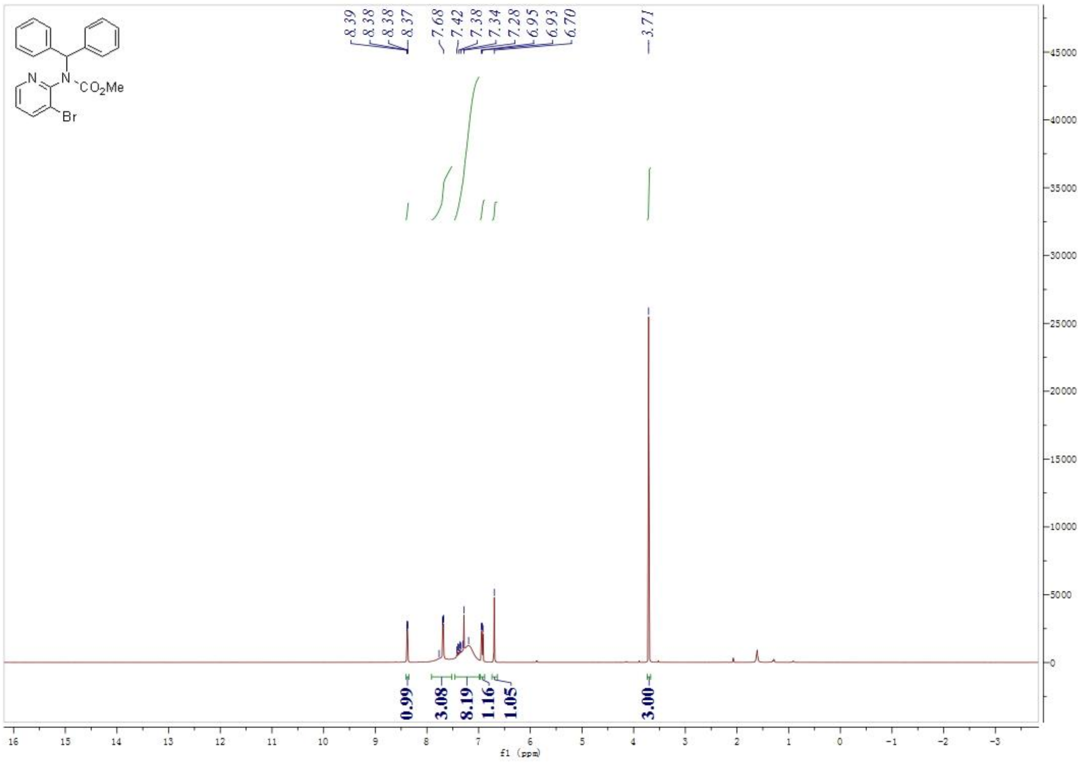

N
CO2Me
Br
8.39 8.38 8.38 8.37 7.68 7.42 7.38 7.34 7.28 6.95 6.93 6.70
3.71
0.99 3.08 8.19 1.16 1.05
3.00
f1 (ppm)

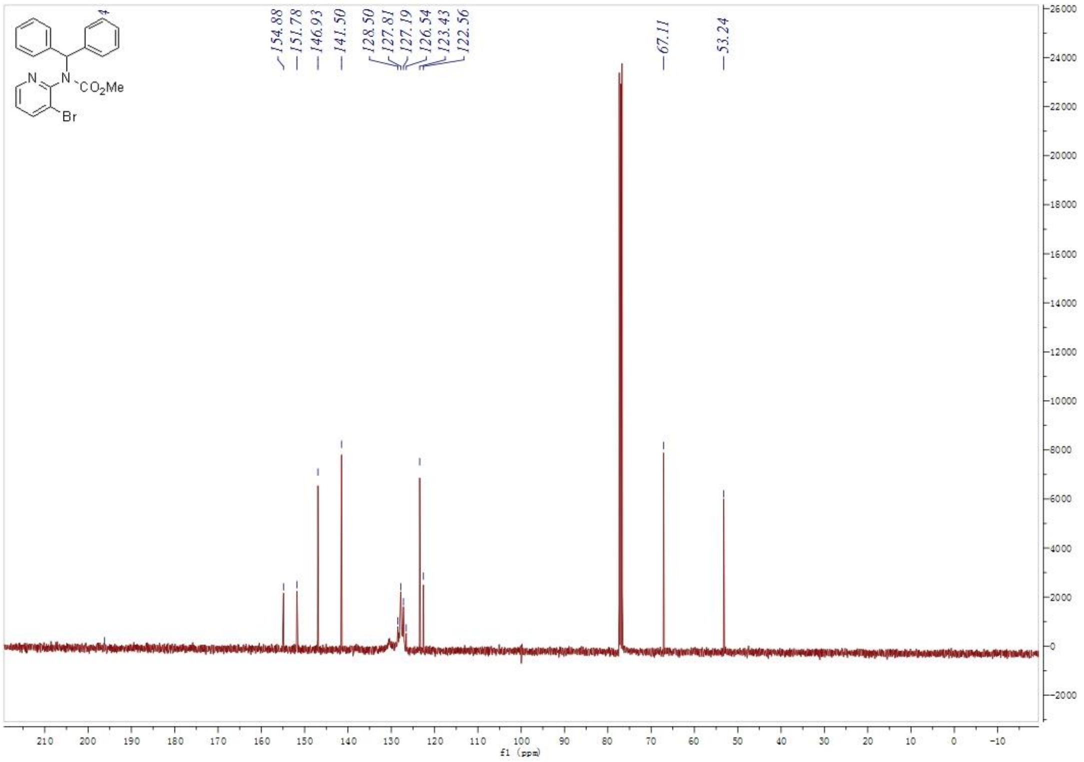

N
CO2Me
Br
154.88 151.78 146.93 141.50
128.50 127.81 127.19 126.54 123.43 122.56
67.11
53.24
f1 (ppm)

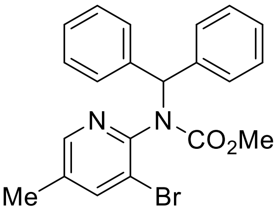

N
N
CO2Me
Me
Br

1aa

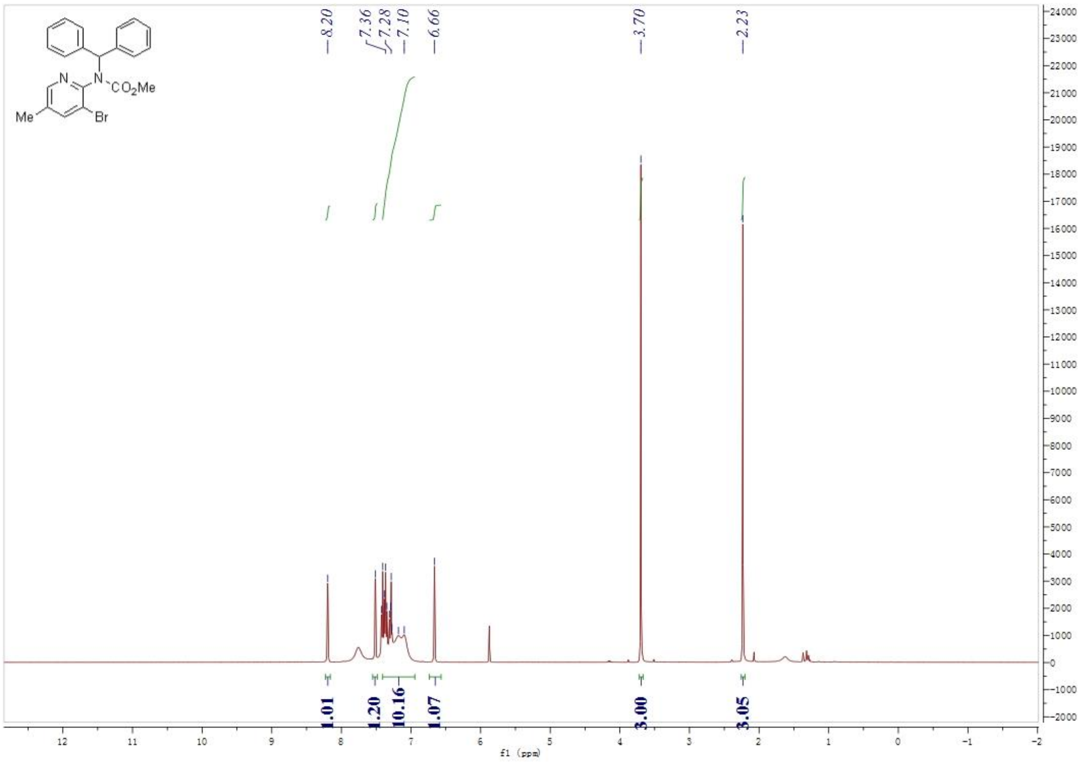

8.20
7.36
7.28
7.10
6.66
3.70
2.23
1.01
1.20
10.16
1.07
3.00
3.05
CO2Me
Me
Br
N
f1 (ppm)

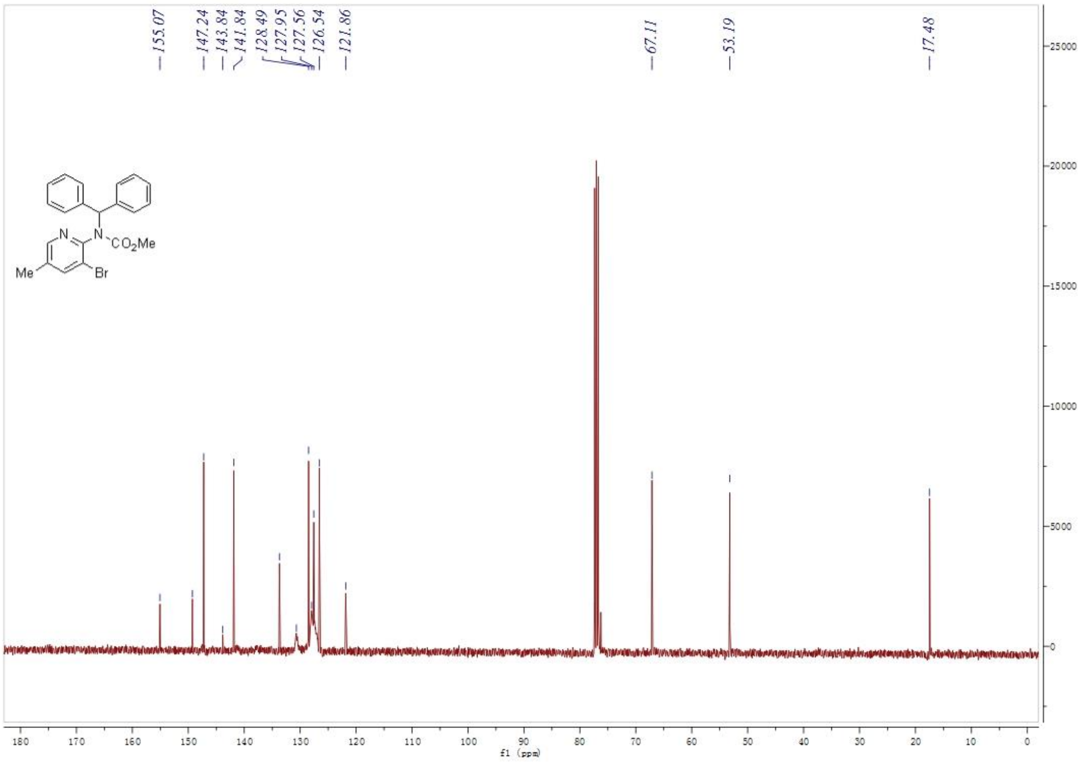

155.07
147.24
143.84
141.84
128.49
127.95
127.56
126.54
121.86
67.11
53.19
17.48
CO2Me
Me
Br
N
f1 (ppm)

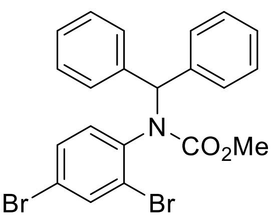

N
CO2Me
Br
Br

3a

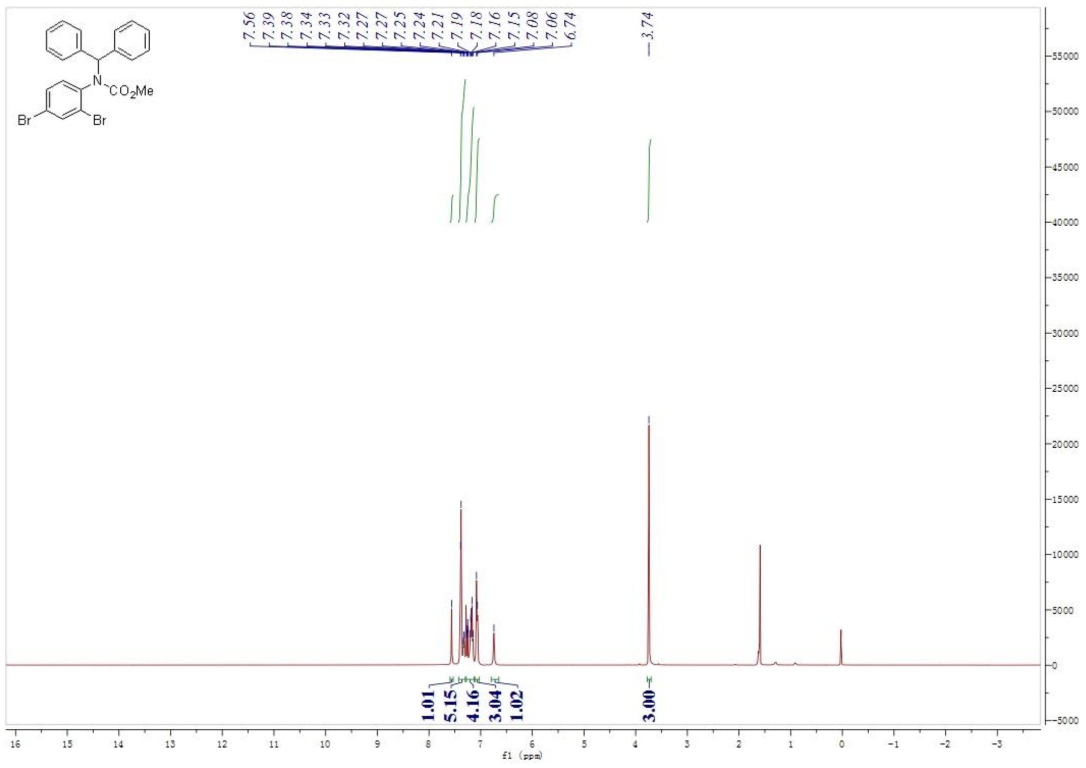

7.56
7.39
7.38
7.34
7.33
7.32
7.27
7.27
7.25
7.24
7.21
7.19
7.18
7.16
7.15
7.08
7.06
6.74
3.74
1.01
5.15
4.16
3.04
1.02
3.00
f1 (ppm)
CO2Me
Br
Br
N

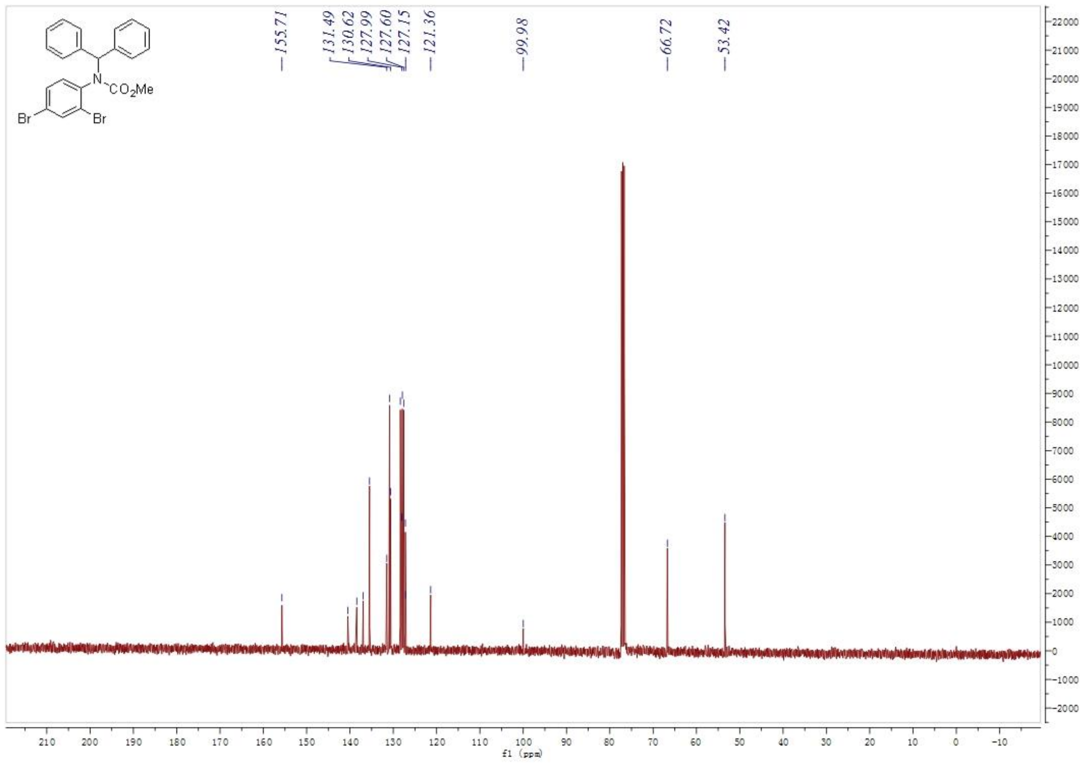

155.71
131.49
130.62
127.99
127.60
127.15
121.36
99.98
66.72
53.42
f1 (ppm)
CO2Me
Br
Br
N

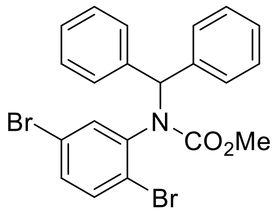

Br
N
CO2Me
Br

3o

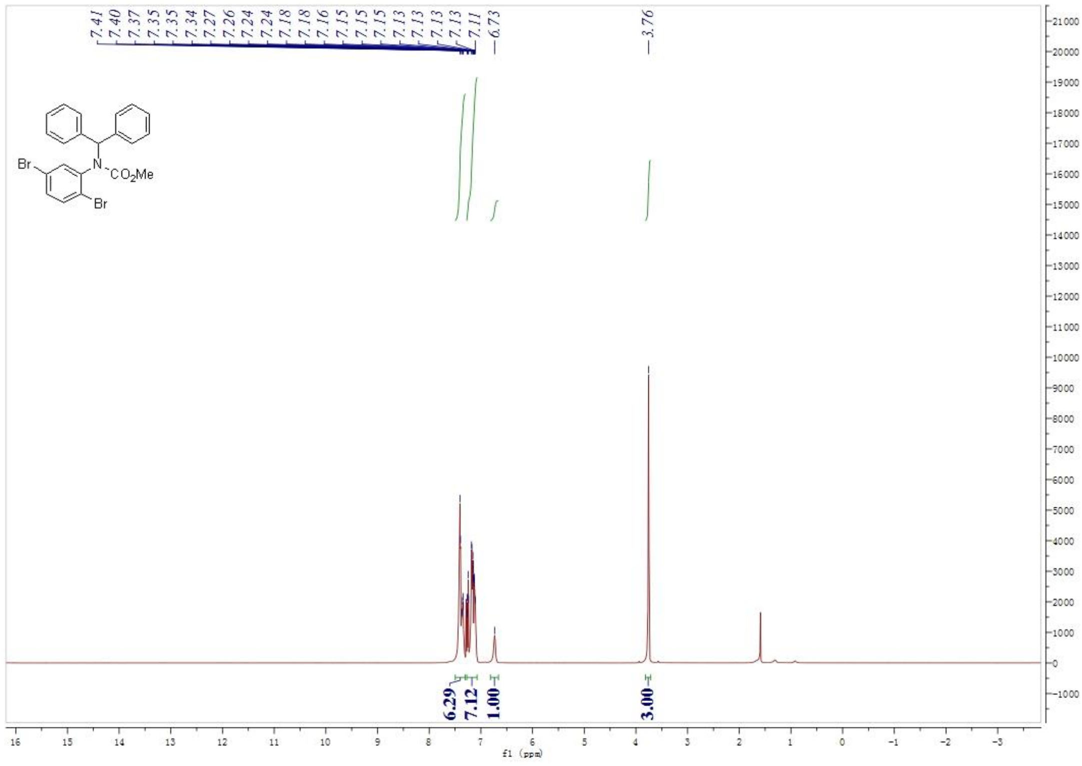

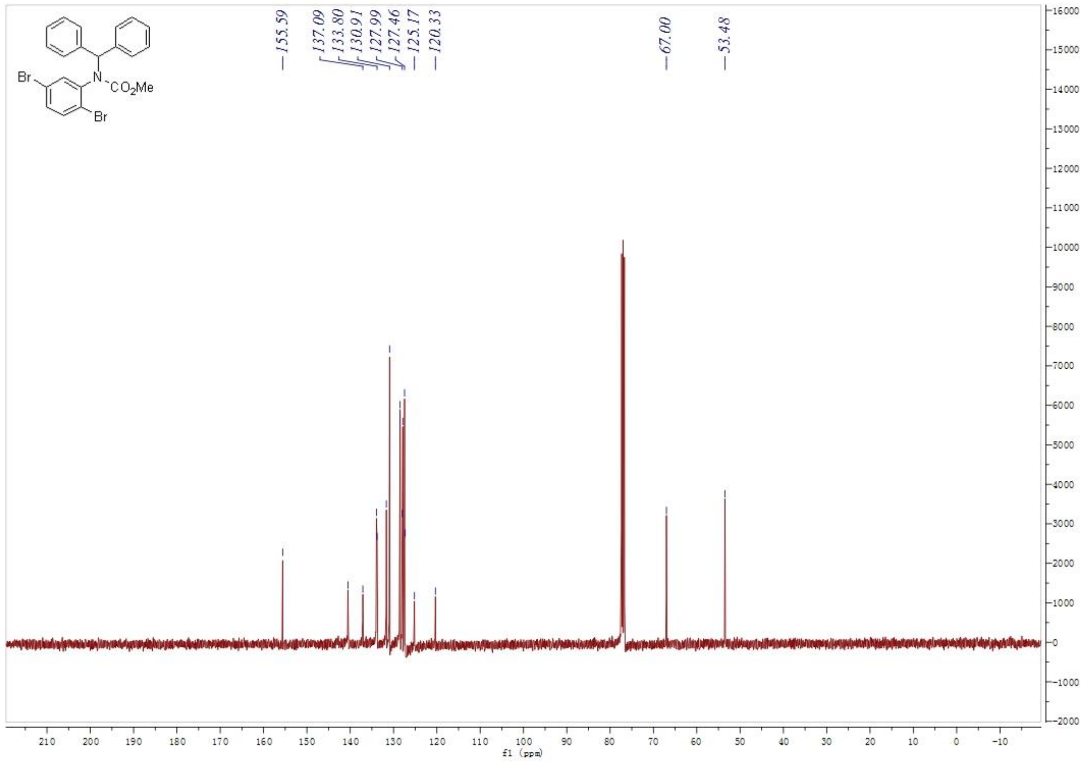

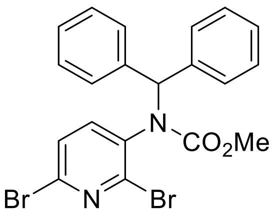

N
CO2Me
Br
N
Br

3p

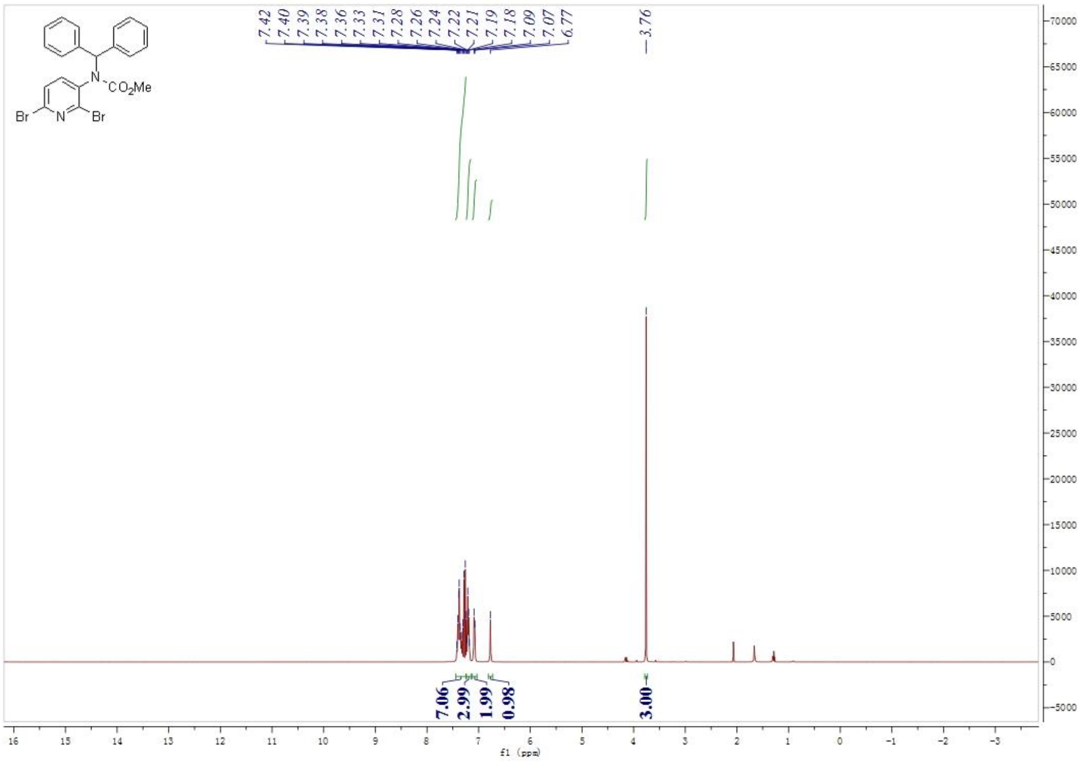

7.42
7.40
7.39
7.38
7.36
7.33
7.31
7.28
7.26
7.24
7.22
7.21
7.19
7.18
7.09
7.07
6.77
3.76
7.06
2.99
1.99
0.98
3.00
f1 (ppm)

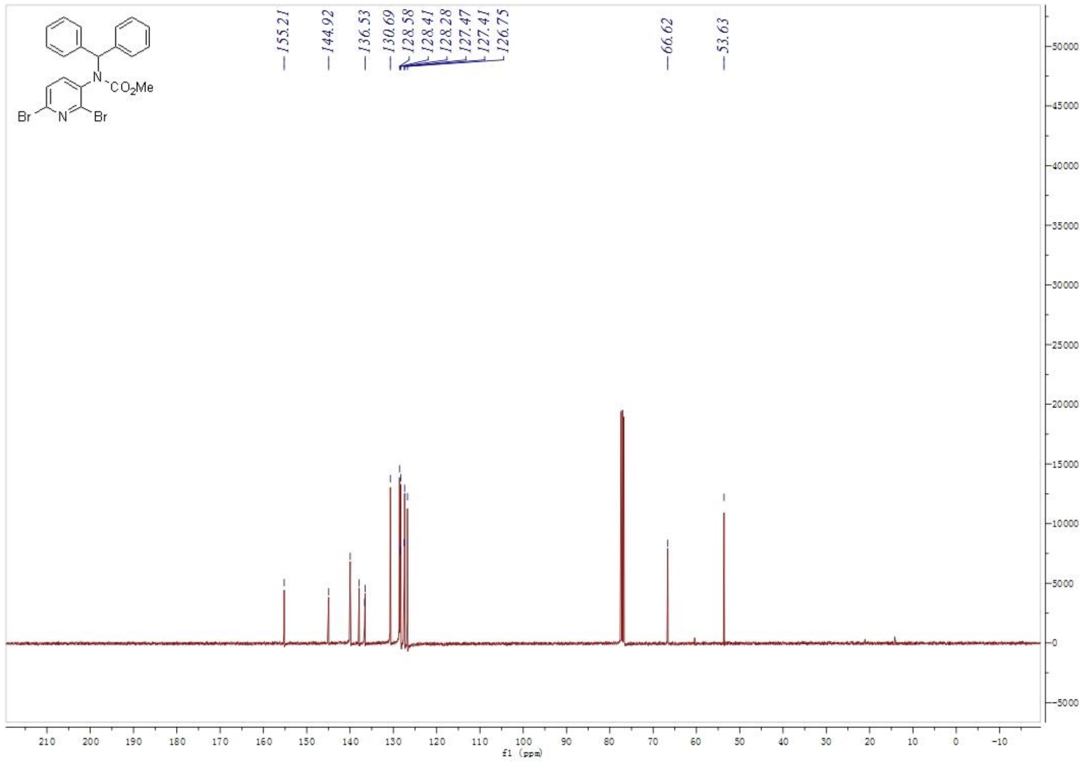
155.21
144.92
136.53
130.69
128.58
128.41
128.28
127.47
127.41
126.75
66.62
53.63
CO2Me
Br
N
Br
f1 (ppm)

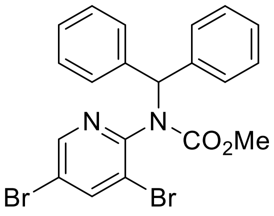
N
N
CO2Me
Br
Br

3q

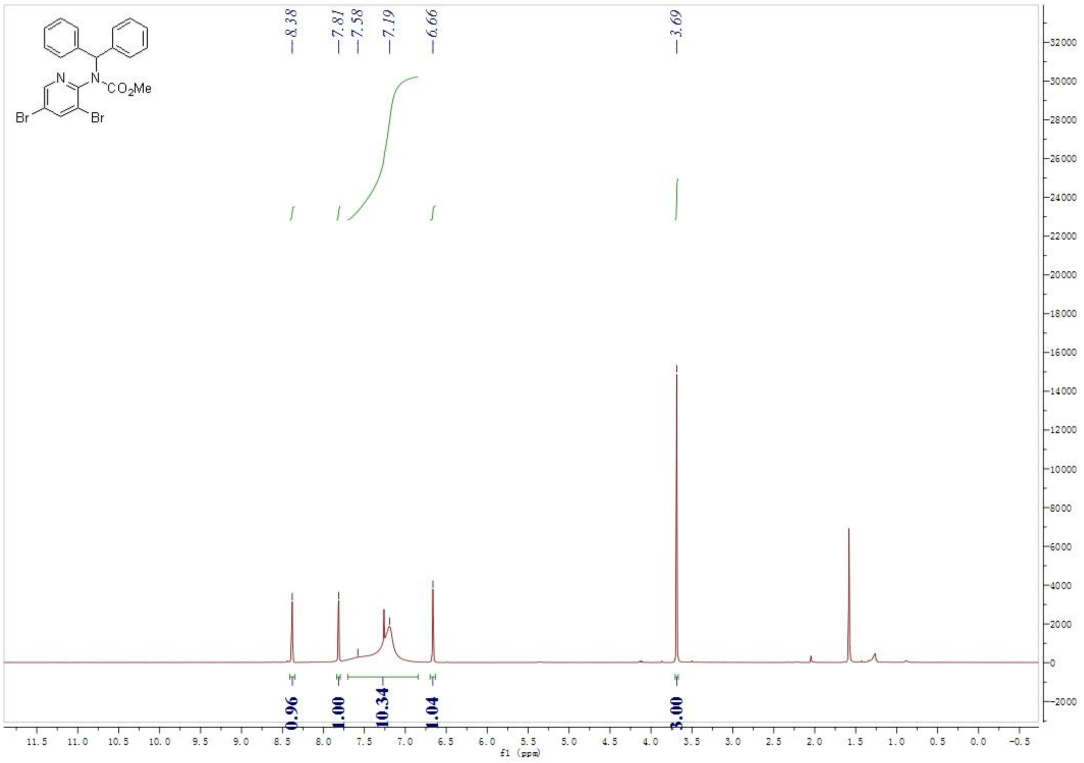
8.38
7.81
7.58
7.19
6.66
3.69
0.96
1.00
10.34
1.04
3.00
f1 (ppm)
CO2Me
Br
Br
N
N

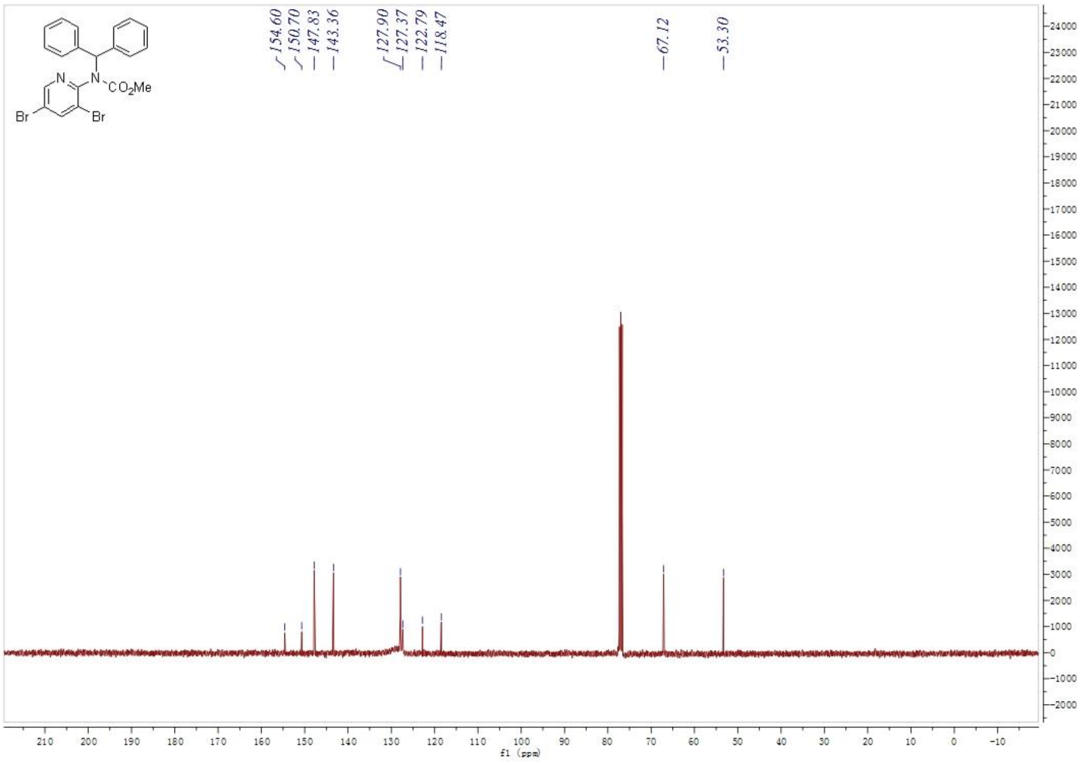
154.60
150.70
147.83
143.36
127.90
127.37
122.79
118.47
67.12
53.30
f1 (ppm)
CO2Me
Br
Br
N
N

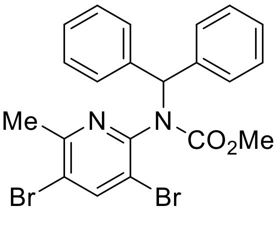
Me
N
N
CO2Me
Br
Br

3r

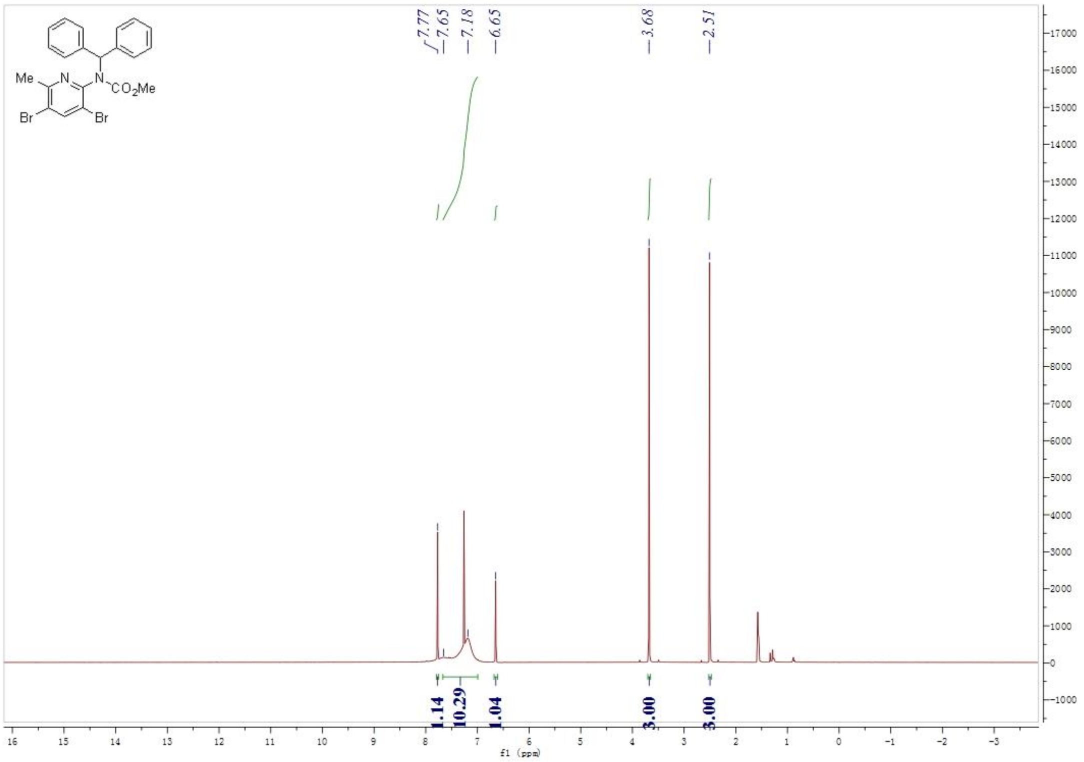
Me
N
N
CO2Me
Br
Br
7.77
7.65
7.18
6.65
3.68
2.51
1.14
10.29
1.04
3.00
3.00
f1 (ppm)

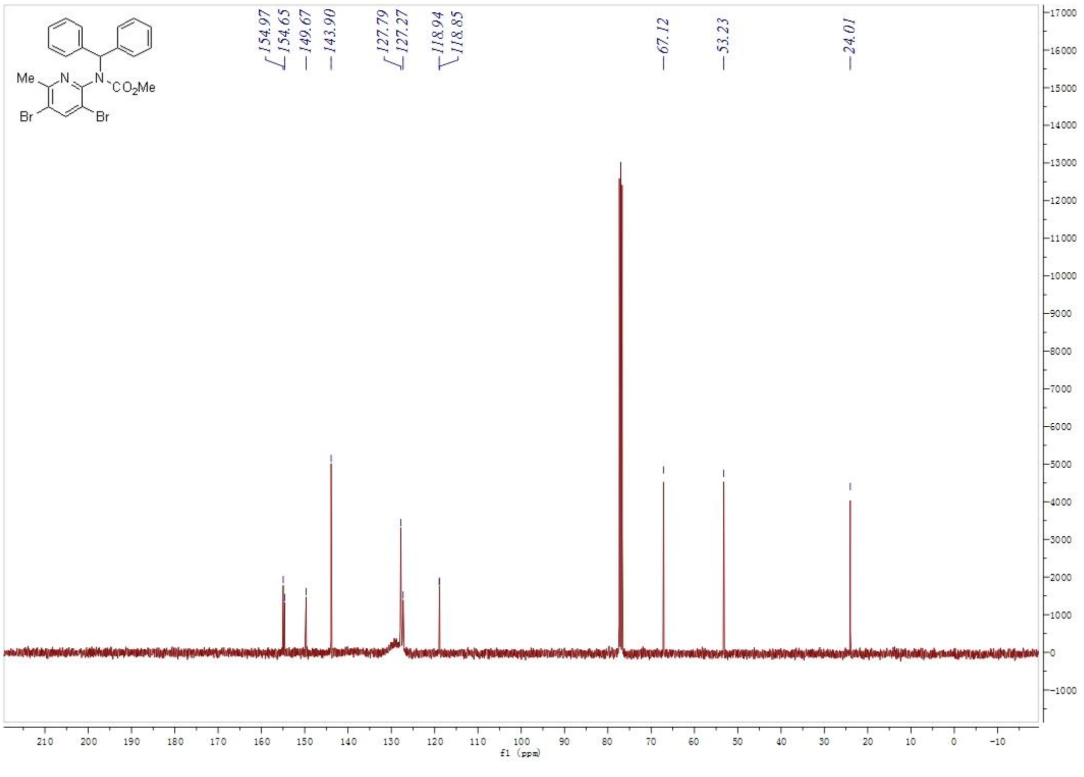
Me
N
N
CO2Me
Br
Br
154.97
154.65
149.67
143.90
127.79
127.27
118.94
118.85
67.12
53.23
24.01
f1 (ppm)

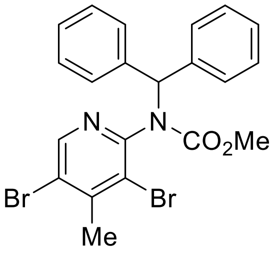
N
N
CO2Me
Br
Br
Me

3s

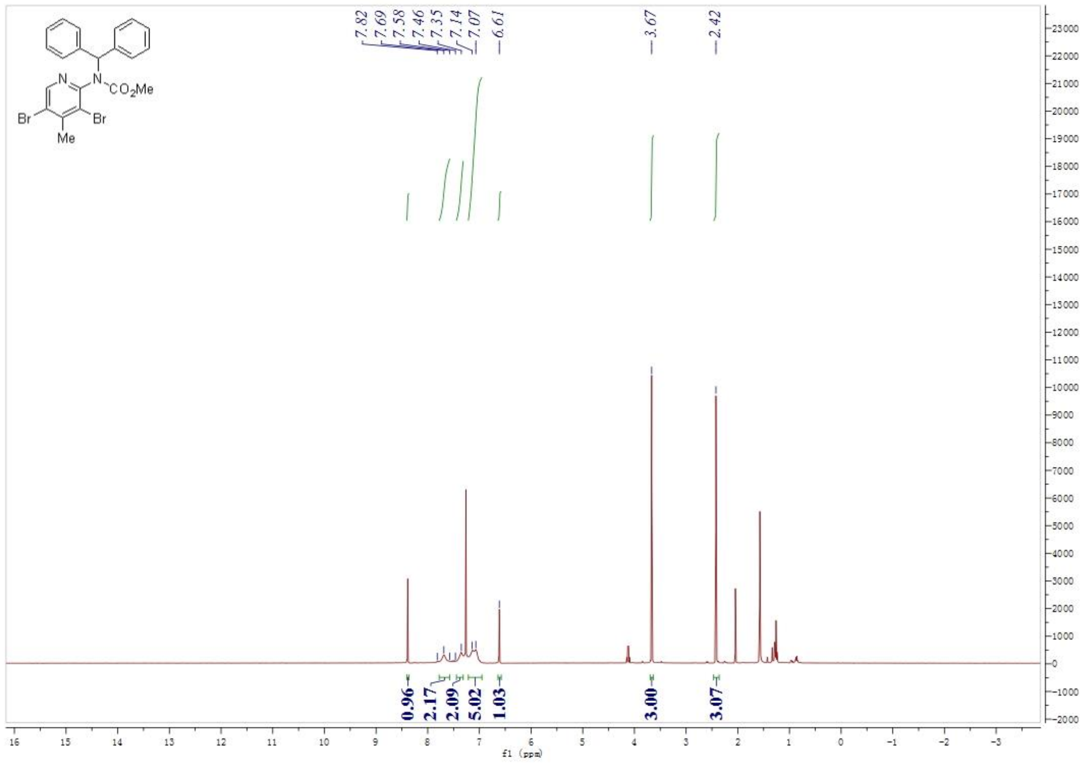
7.82
7.69
7.58
7.46
7.35
7.14
7.07
6.61
3.67
2.42
0.96
2.17
2.09
5.02
1.03
3.00
3.07
f1 (ppm)

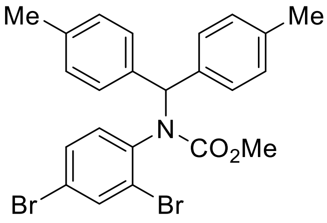
Me
Me
N
CO2Me
Br
Br

3t

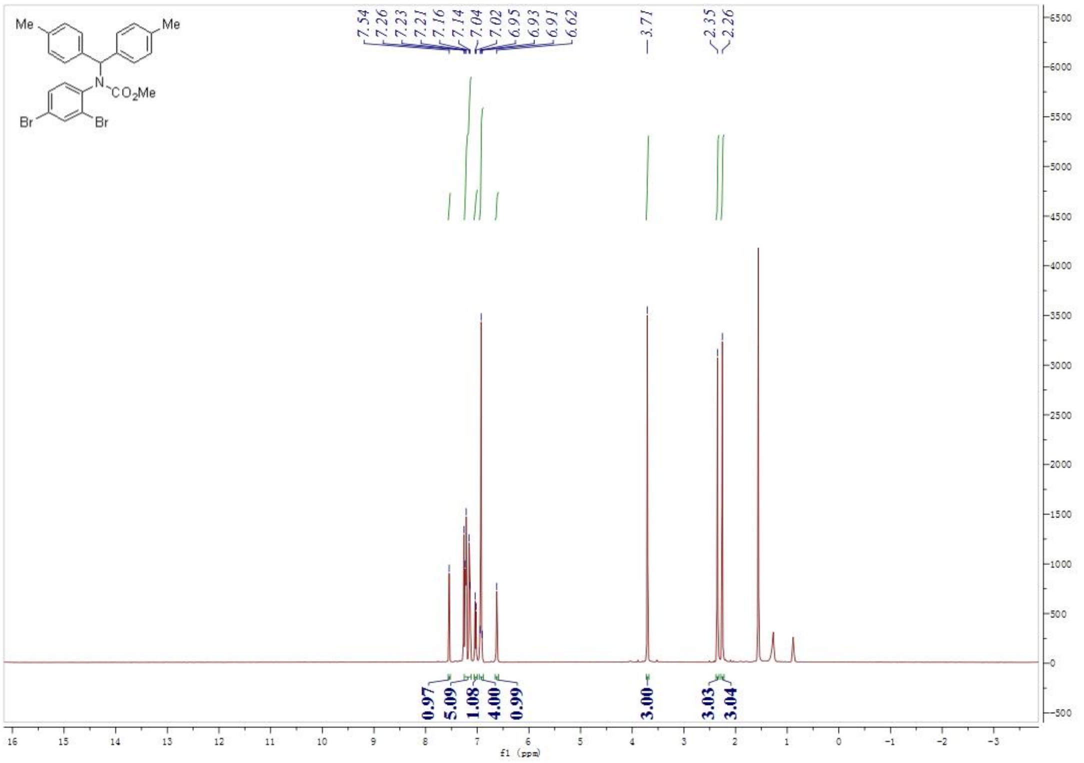
7.54
7.26
7.23
7.21
7.16
7.14
7.04
7.02
6.95
6.93
6.91
6.62
3.71
2.35
2.26
0.97
5.09
1.08
4.00
0.99
3.00
3.03
3.04
f1 (ppm)

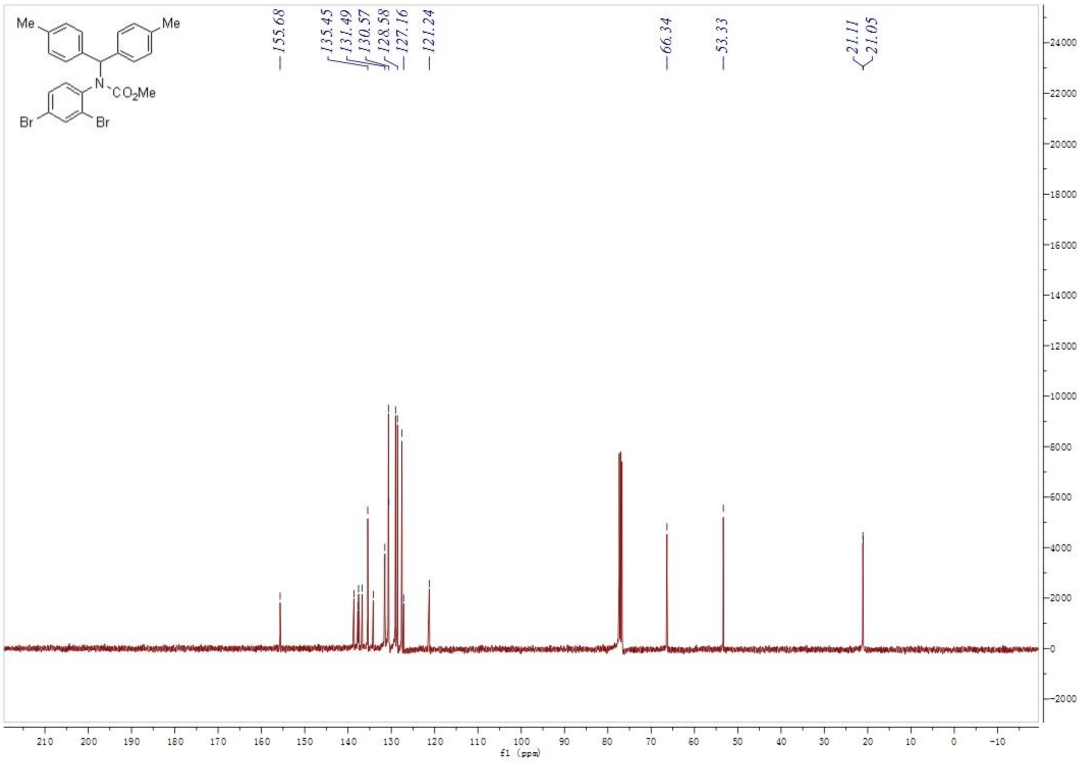
155.68
135.45
131.49
130.57
128.58
127.16
121.24
66.34
53.33
21.11
21.05
f1 (ppm)

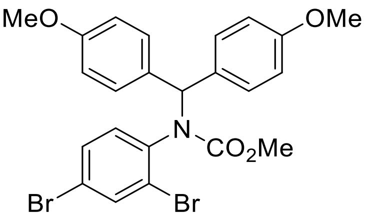
MeO
OMe
N
CO_2Me
Br
Br

3u

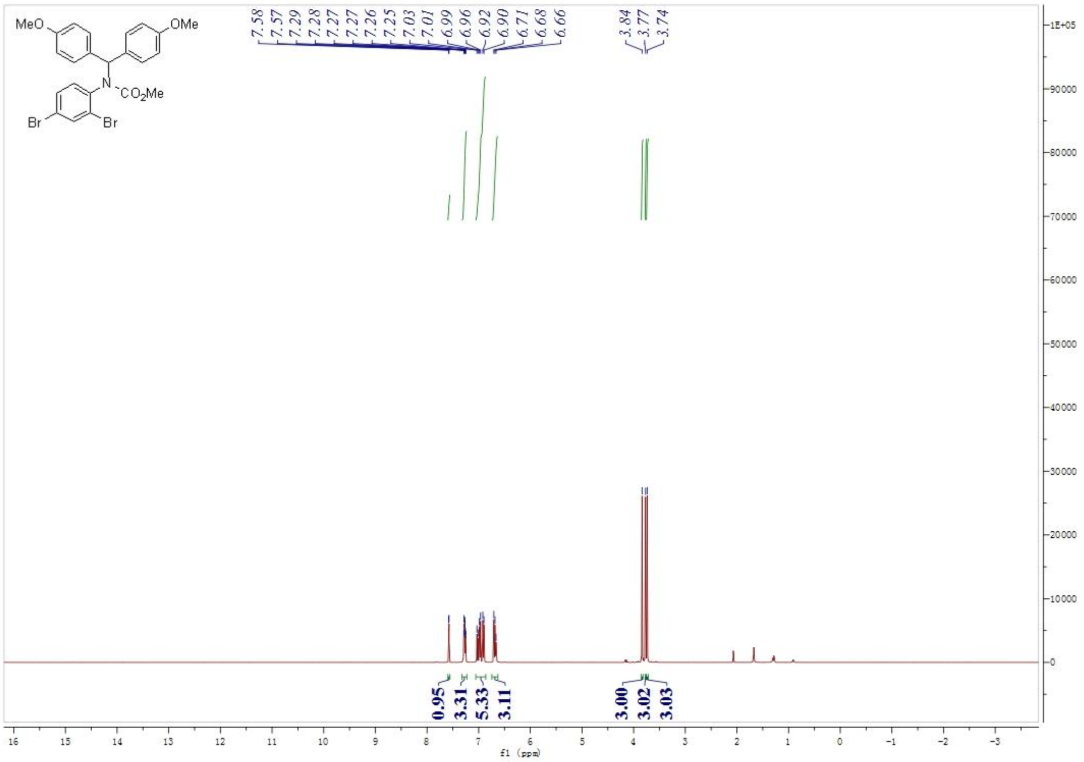
MeO
OMe
N
CO2Me
Br
Br
7.58
7.57
7.29
7.28
7.27
7.27
7.26
7.25
7.03
7.01
6.99
6.96
6.92
6.90
6.71
6.68
6.66
3.84
3.77
3.74
0.95
3.31
5.33
3.11
3.00
3.02
3.03
f1 (ppm)

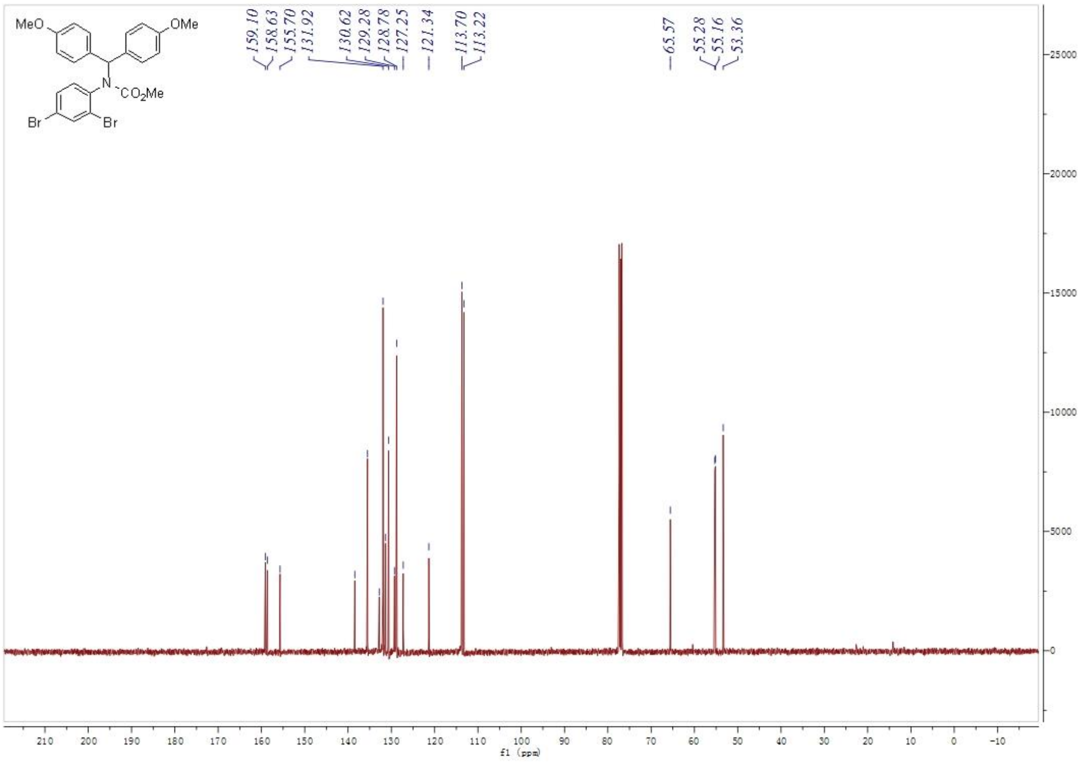
MeO
OMe
N
CO2Me
Br
Br
159.10
158.63
155.70
131.92
130.62
129.28
128.78
127.25
121.34
113.70
113.22
65.57
55.28
55.16
53.36
f1 (ppm)

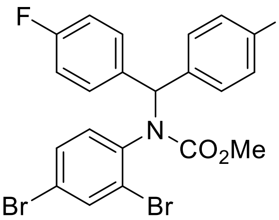
F
F
N
CO2Me
Br
Br

3v

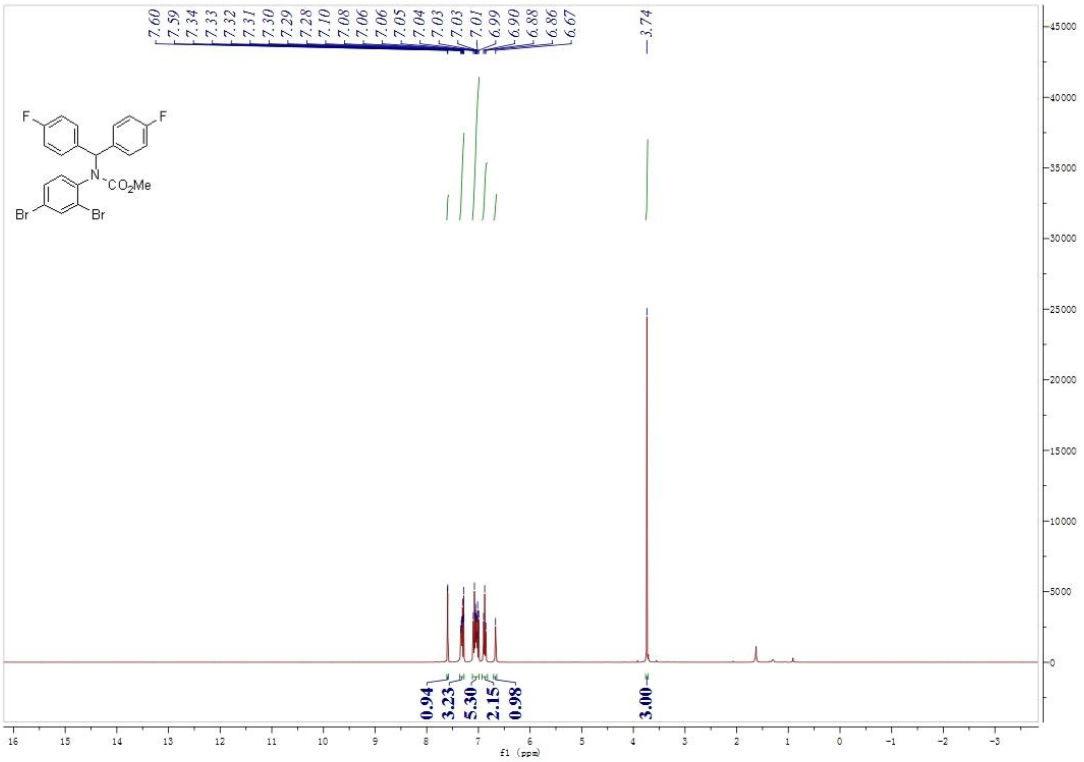
7.60
7.59
7.34
7.33
7.32
7.31
7.30
7.29
7.28
7.10
7.08
7.06
7.06
7.05
7.04
7.03
7.03
7.01
6.99
6.90
6.88
6.86
6.67
3.74
F
F
N
CO2Me
Br
Br
0.94
3.23
5.30
2.15
0.98
3.00
f1 (ppm)

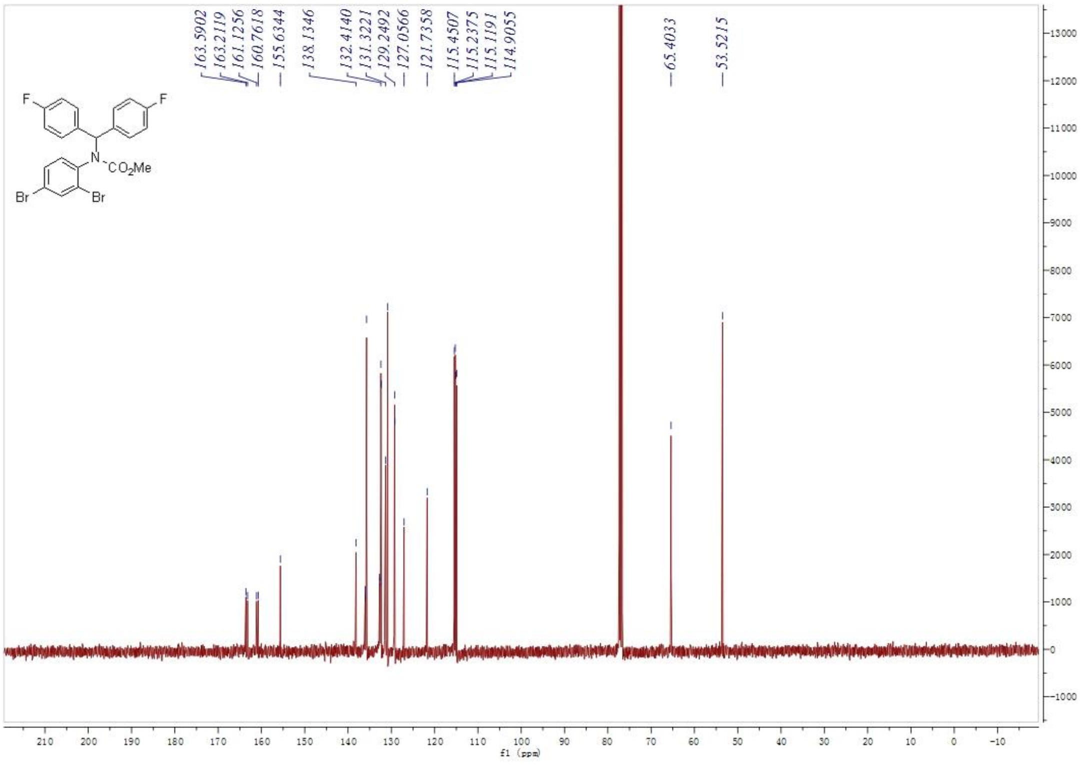
163.5902
163.2119
161.1256
160.7618
155.6344
138.1346
132.4140
131.3221
129.2492
127.0566
121.7358
115.4507
115.2375
115.1191
114.9055
65.4033
53.5215
F
F
N
CO2Me
Br
Br
f1 (ppm)

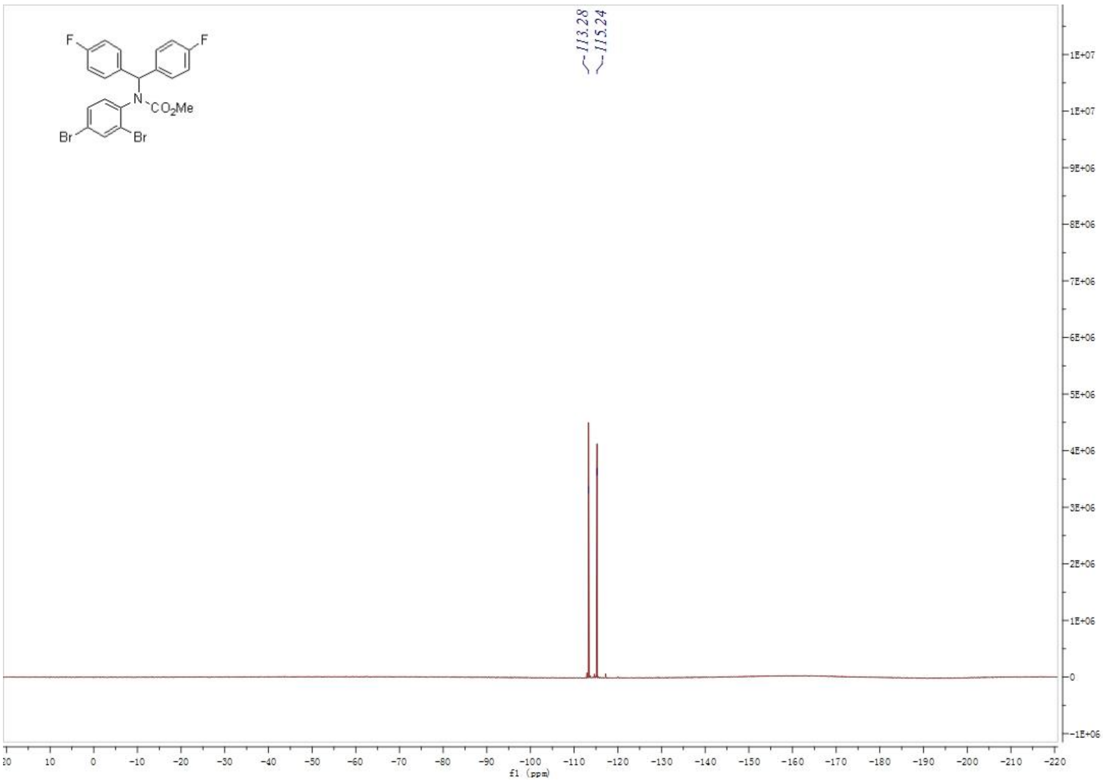

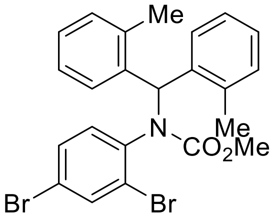

3w

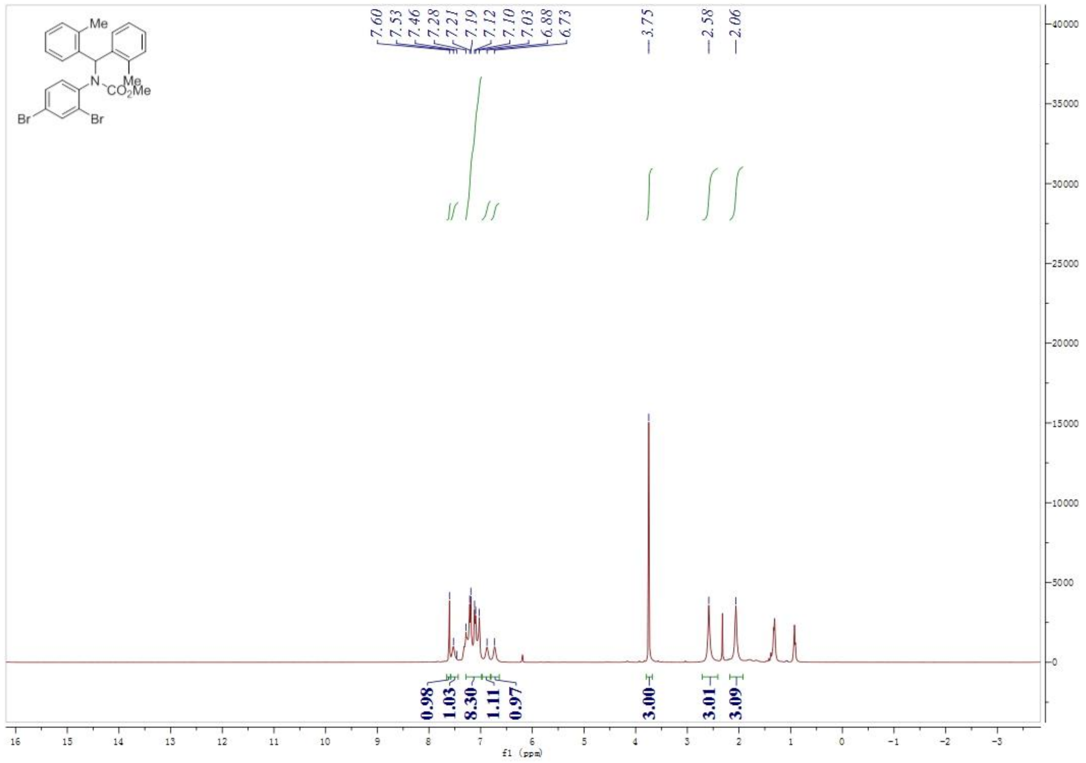

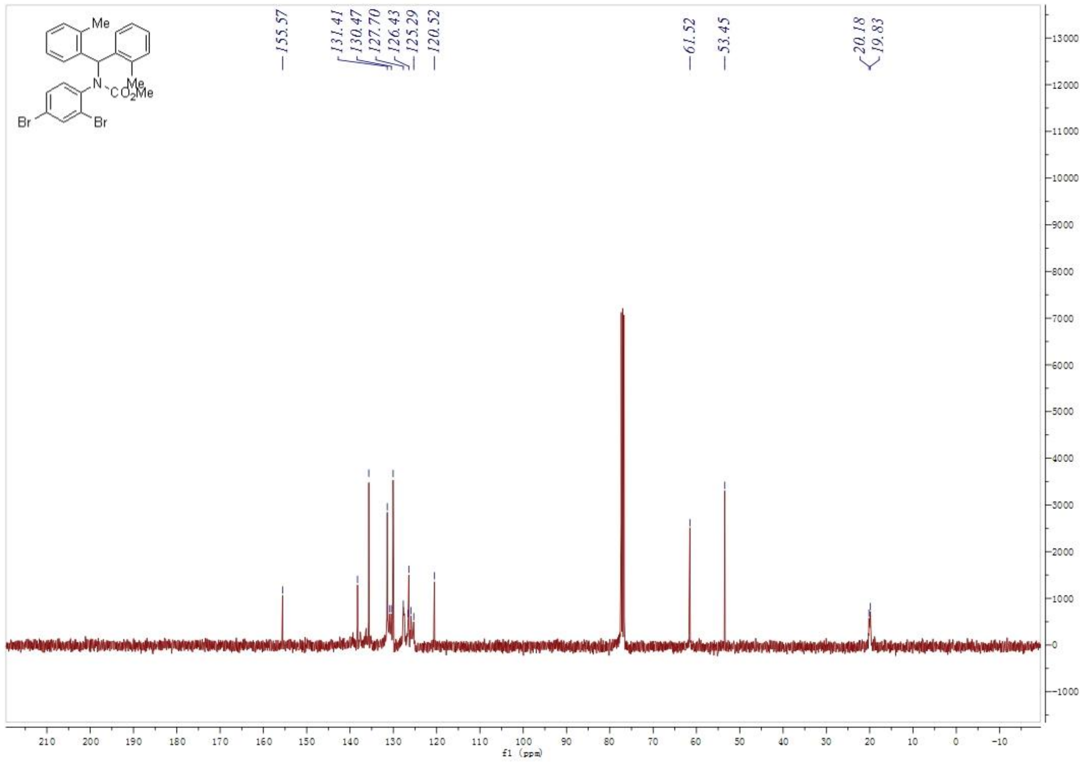

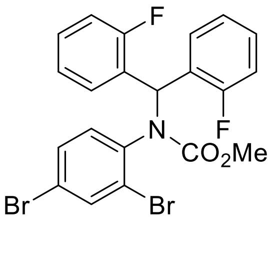

3x

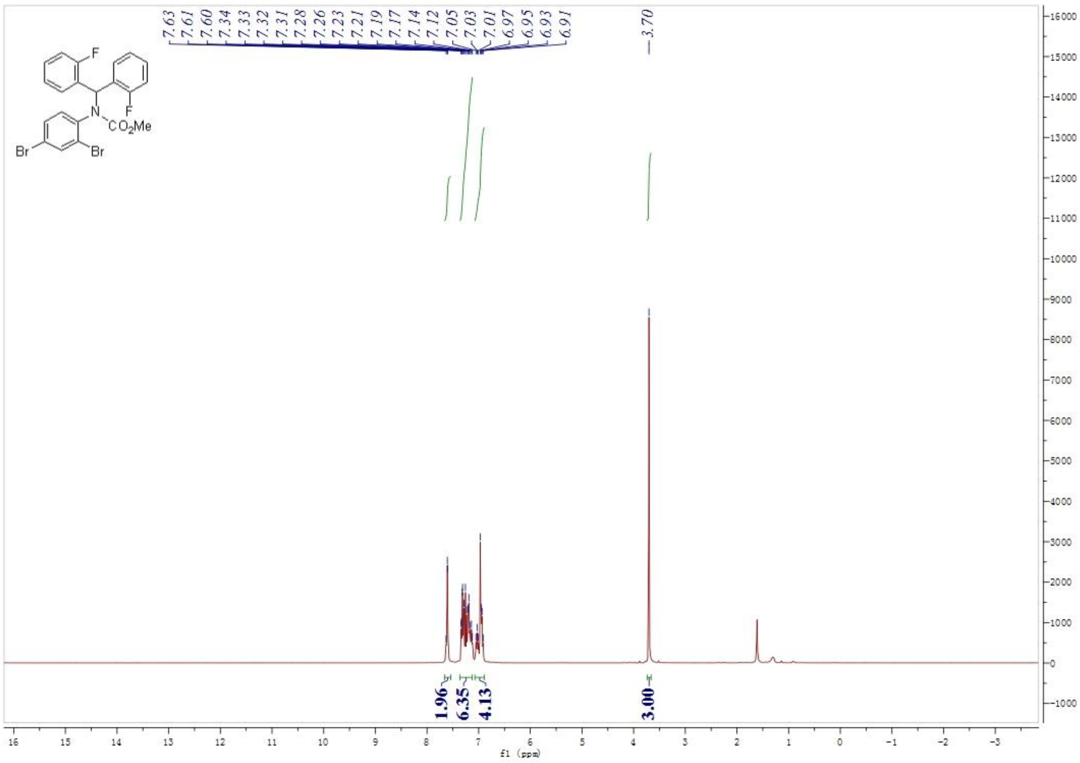
7.63
7.61
7.60
7.34
7.33
7.32
7.31
7.28
7.26
7.23
7.21
7.19
7.17
7.14
7.12
7.05
7.03
7.01
6.97
6.95
6.93
6.91
3.70
1.96
6.35
4.13
3.00
f1 (ppm)
F
F
N
CO2Me
Br
Br

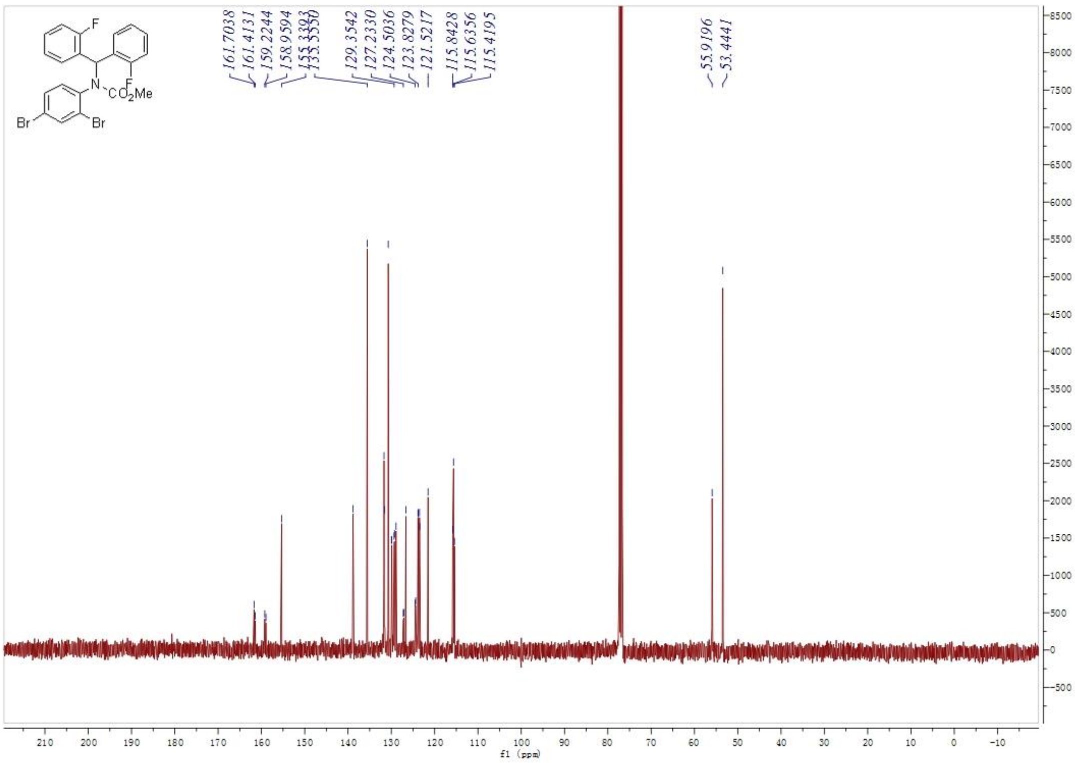
161.7038
161.4131
159.2244
158.9594
f1 (ppm)
129.3542
127.2330
124.5036
123.8279
121.5217
115.8428
115.6356
115.4195
55.9196
53.4441
F
F
N
CO2Me
Br
Br

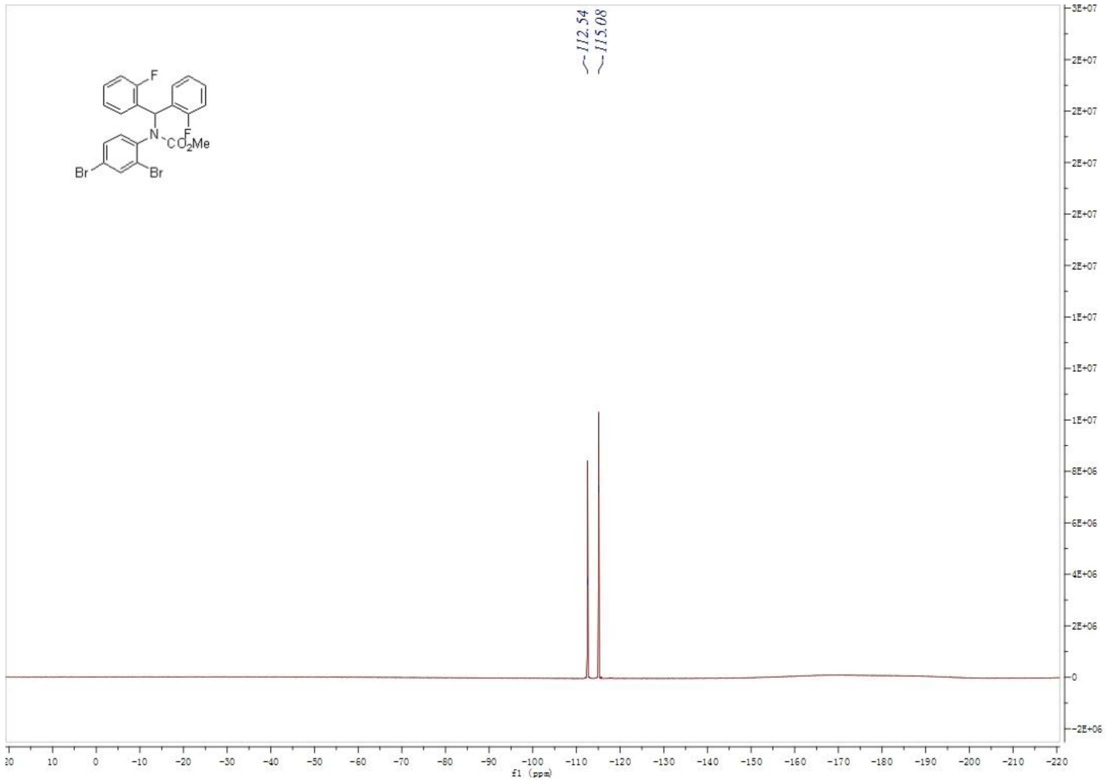

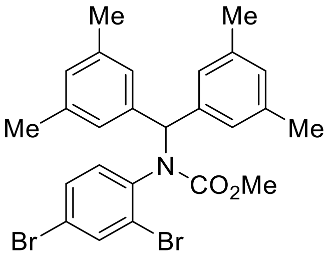

3y

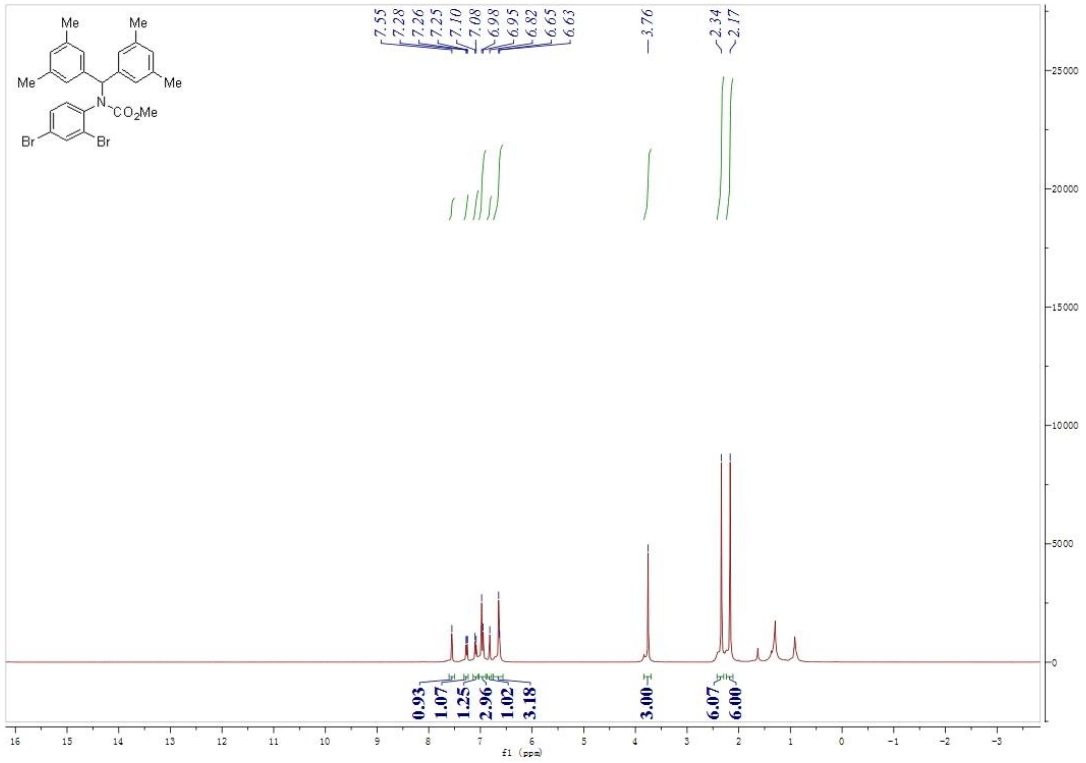
7.55 7.28 7.26 7.25 7.10 7.08 6.98 6.95 6.82 6.65 6.63
3.76
2.34 2.17
Me Me Me Me N CO2Me Br Br
0.93 1.07 1.25 2.96 1.02 3.18
3.00
6.07 6.00
f1 (ppm)

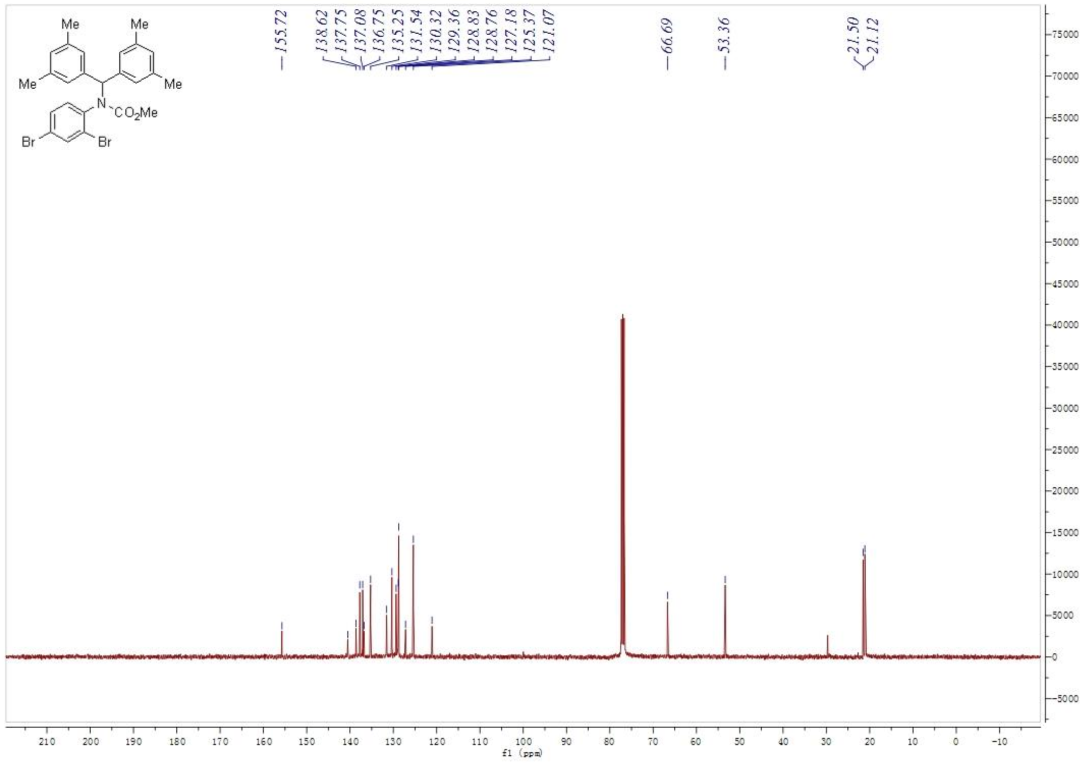
155.72
138.62 137.75 137.08 136.75 135.25 131.54 130.32 129.36 128.83 128.76 127.18 125.37 121.07
66.69
53.36
21.50 21.12
Me Me Me Me N CO2Me Br Br
f1 (ppm)

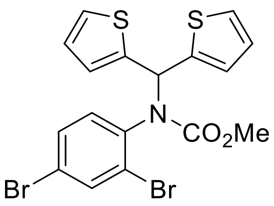
S S N CO2Me Br Br

3z

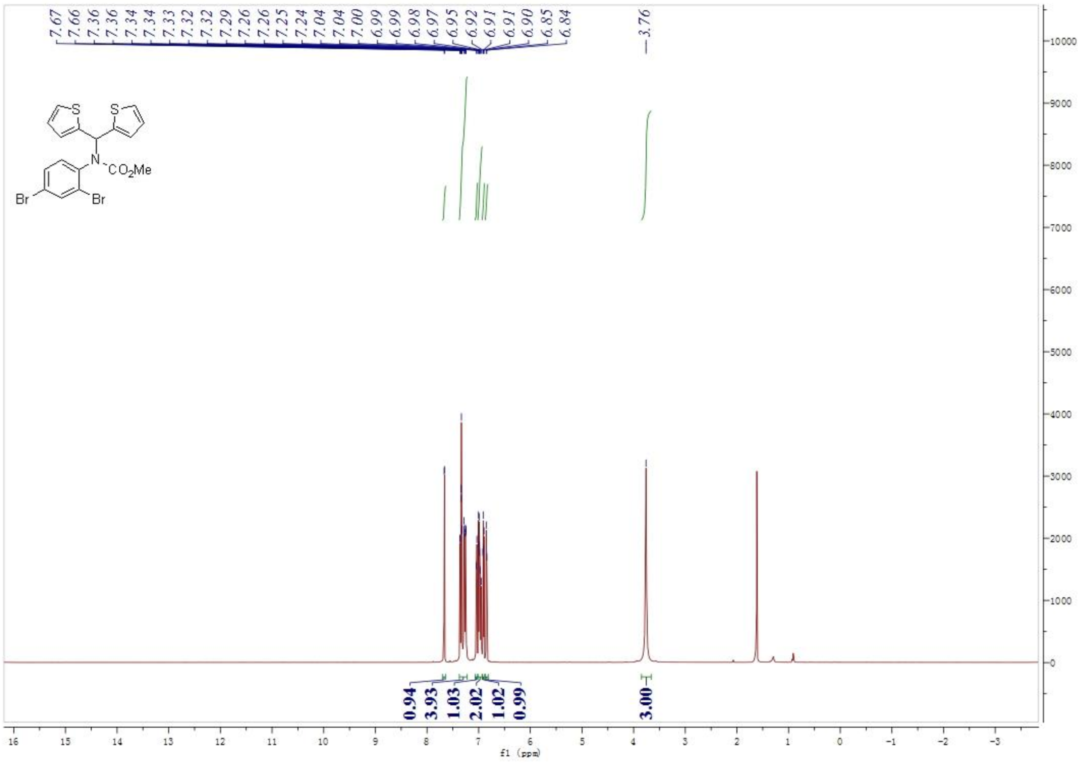
7.67 7.66 7.36 7.36 7.34 7.34 7.33 7.32 7.32 7.29 7.26 7.26 7.25 7.24 7.04 7.04 7.00 6.99 6.99 6.98 6.97 6.95 6.92 6.91 6.91 6.90 6.85 6.84
3.76
S
N
CO2Me
Br
Br
0.94 3.93 1.03 2.02 1.02 0.99
3.00
f1 (ppm)

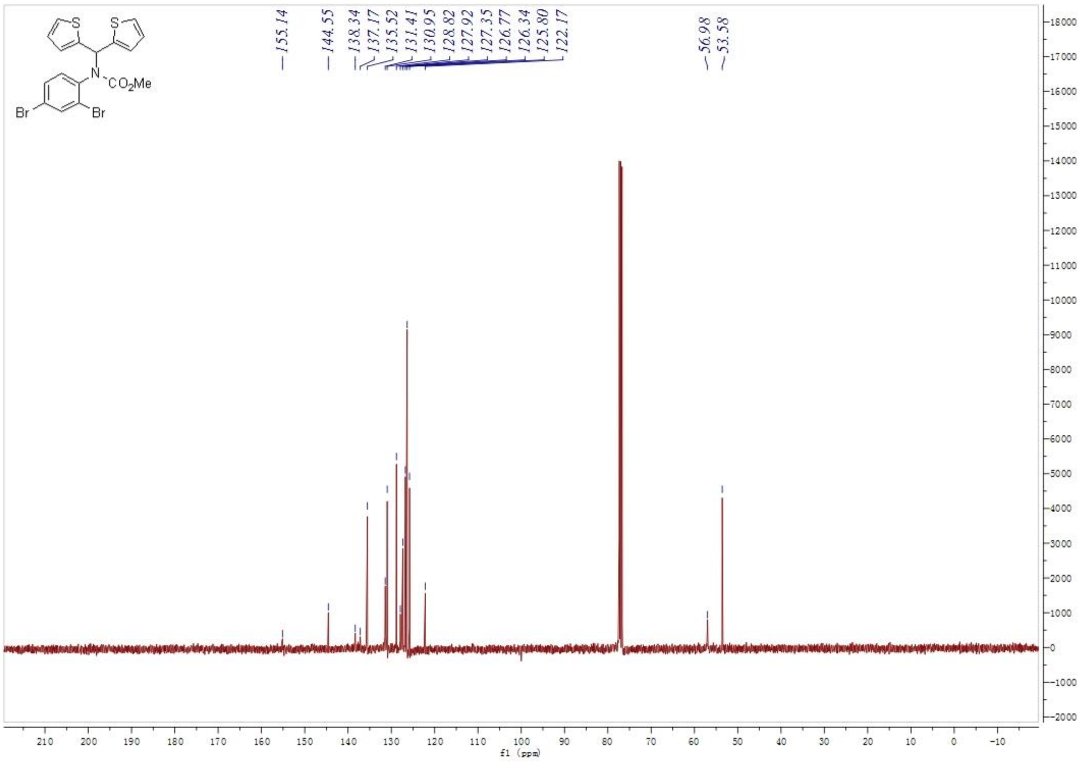
155.14
144.55
138.34 137.17 135.52 131.41 130.95 128.82 127.92 127.35 126.77 126.34 125.80 122.17
56.98 53.58
S
N
CO2Me
Br
Br
f1 (ppm)

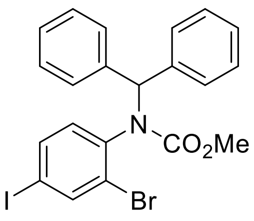
N
CO2Me
I
Br

3aa

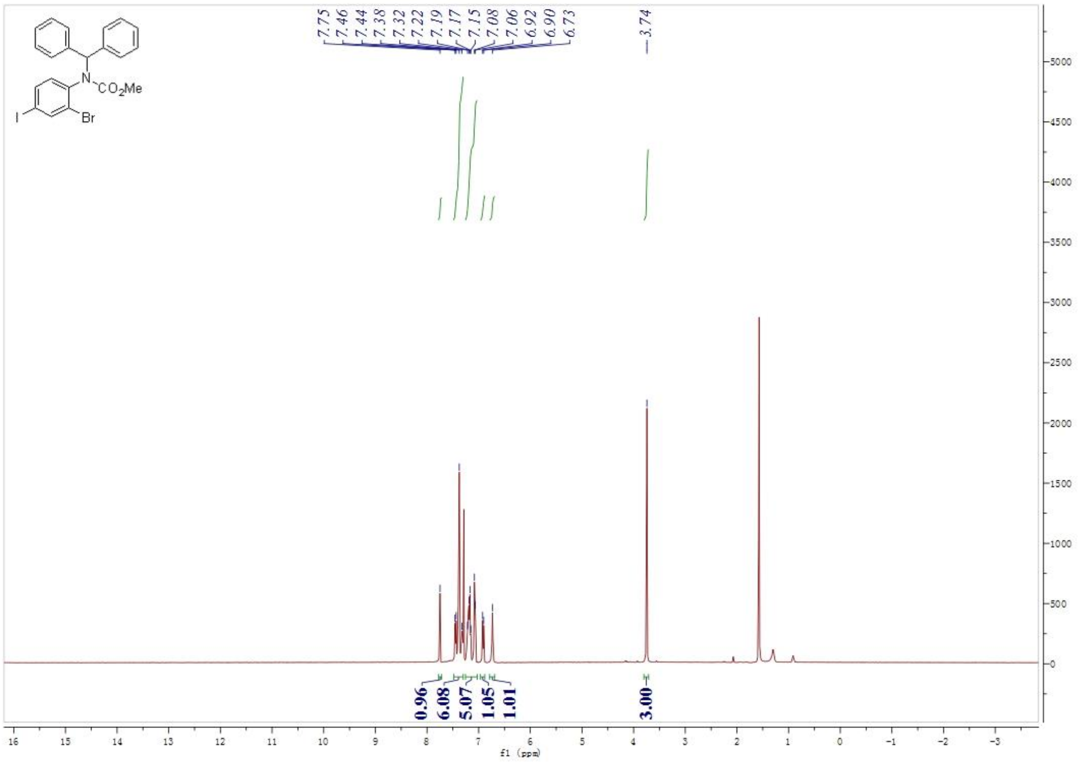
7.75 7.46 7.44 7.38 7.32 7.22 7.19 7.17 7.15 7.08 7.06 6.92 6.90 6.73
3.74
N
CO2Me
I
Br
0.96 6.08 5.07 1.05 1.01
3.00
f1 (ppm)

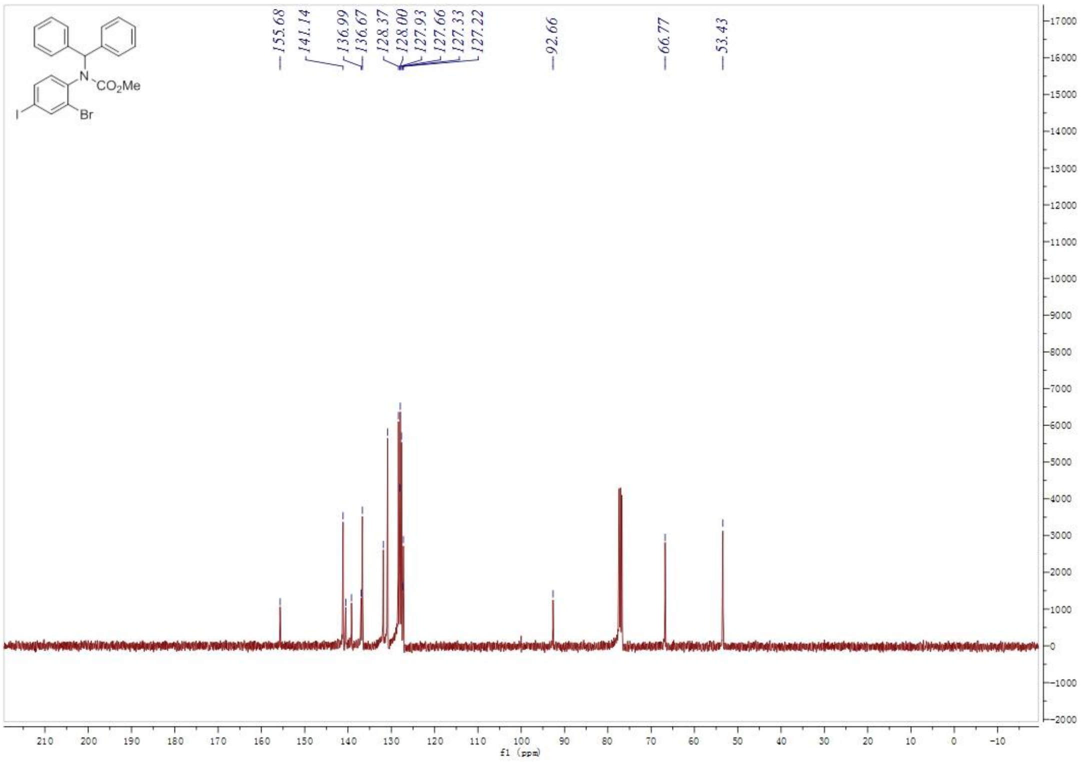
155.68
141.14 136.99 136.67 128.37 128.00 127.93 127.66 127.33 127.22
92.66
66.77
53.43
N
CO2Me
I
Br
f1 (ppm)

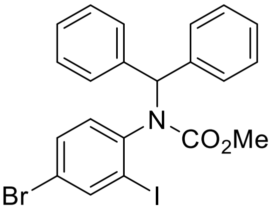
N
CO2Me
Br
I

3ab

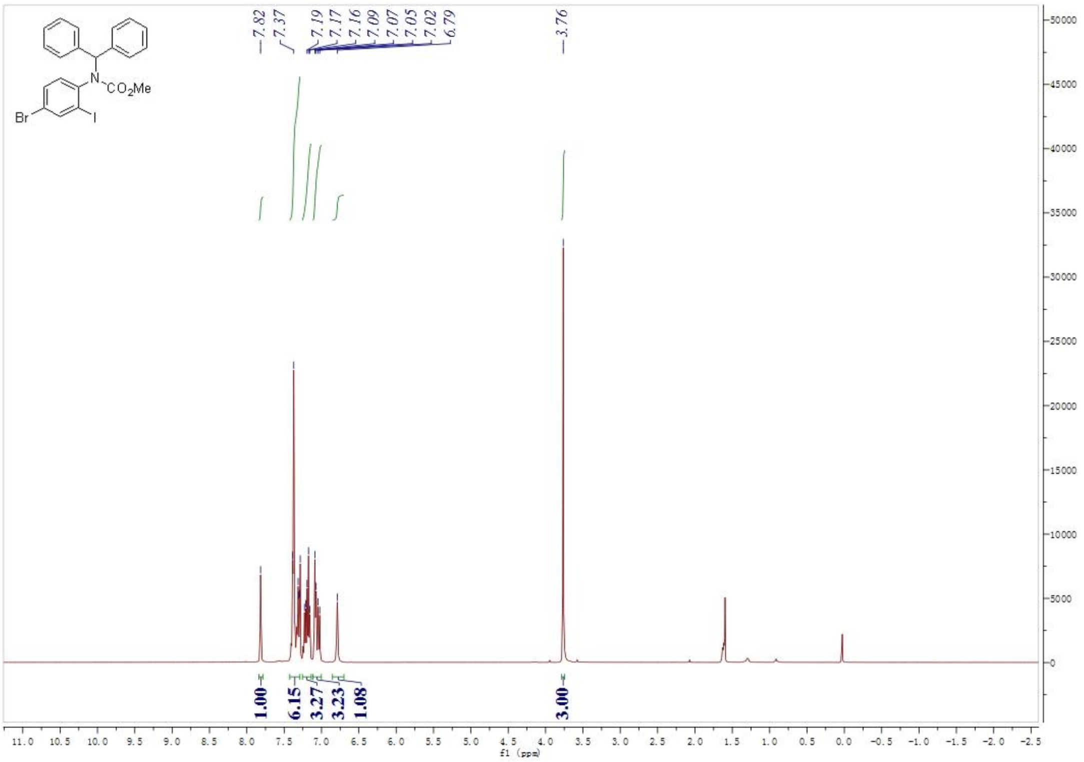
7.82
7.37
7.19
7.17
7.16
7.09
7.07
7.05
7.02
6.79
3.76
CO_2Me
Br
I
N
1.00
6.15
3.27
3.23
1.08
3.00
f1 (ppm)

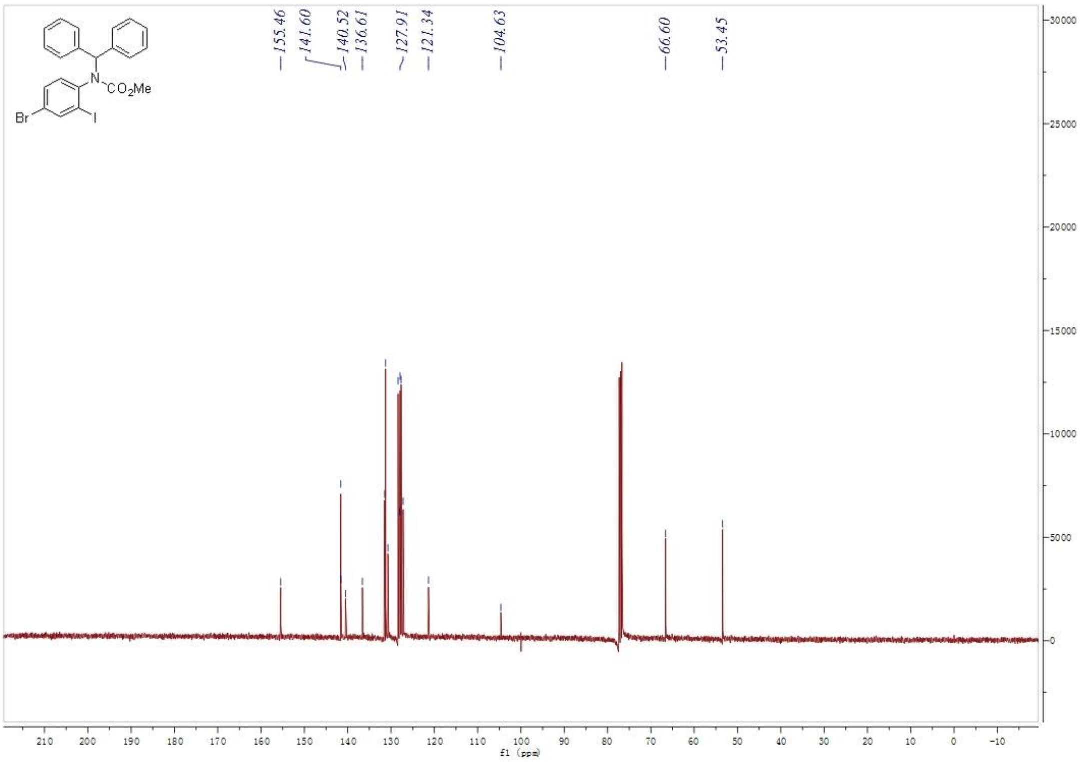
155.46
141.60
140.52
136.61
127.91
121.34
104.63
66.60
53.45
CO_2Me
Br
I
N
f1 (ppm)

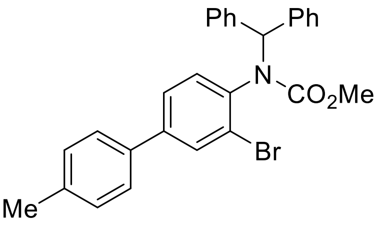
Ph
Ph
N
CO_2Me
Br
Me

3ac

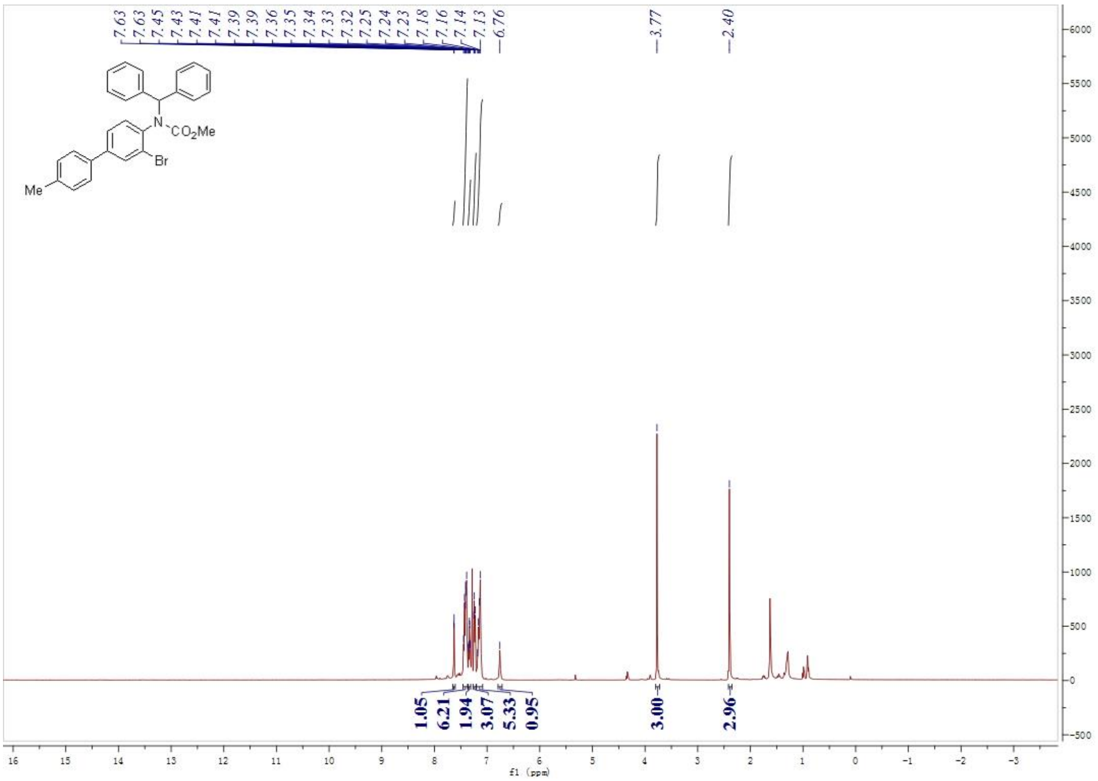
7.63
7.63
7.45
7.43
7.41
7.39
7.39
7.36
7.35
7.34
7.33
7.32
7.25
7.24
7.23
7.18
7.16
7.14
7.13
6.76
3.77
2.40
N
CO2Me
Br
Me
1.05
6.21
1.94
3.07
5.33
0.95
3.00
2.96
f1 (ppm)

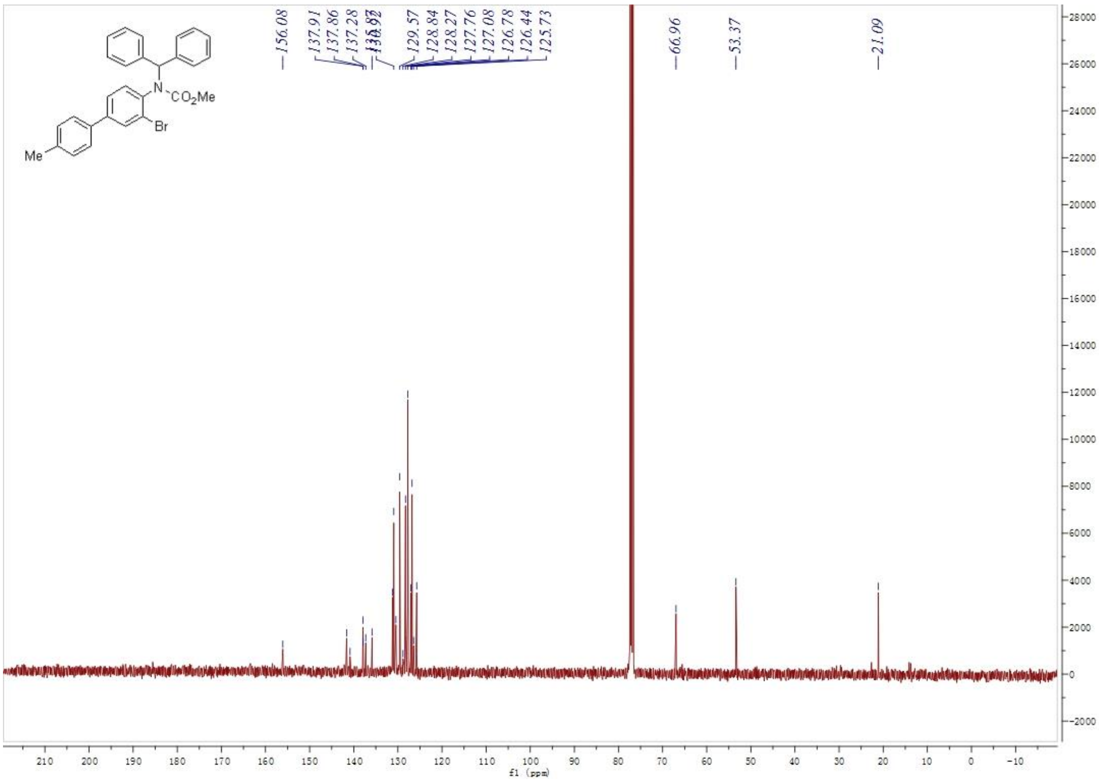
156.08
137.91
137.86
137.28
129.57
128.84
128.27
127.76
127.08
126.78
126.44
125.73
66.96
53.37
21.09
N
CO2Me
Br
Me
f1 (ppm)

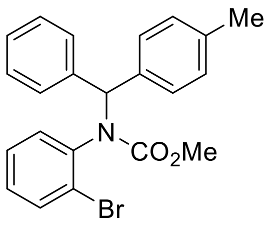
Me
N
CO2Me
Br

5

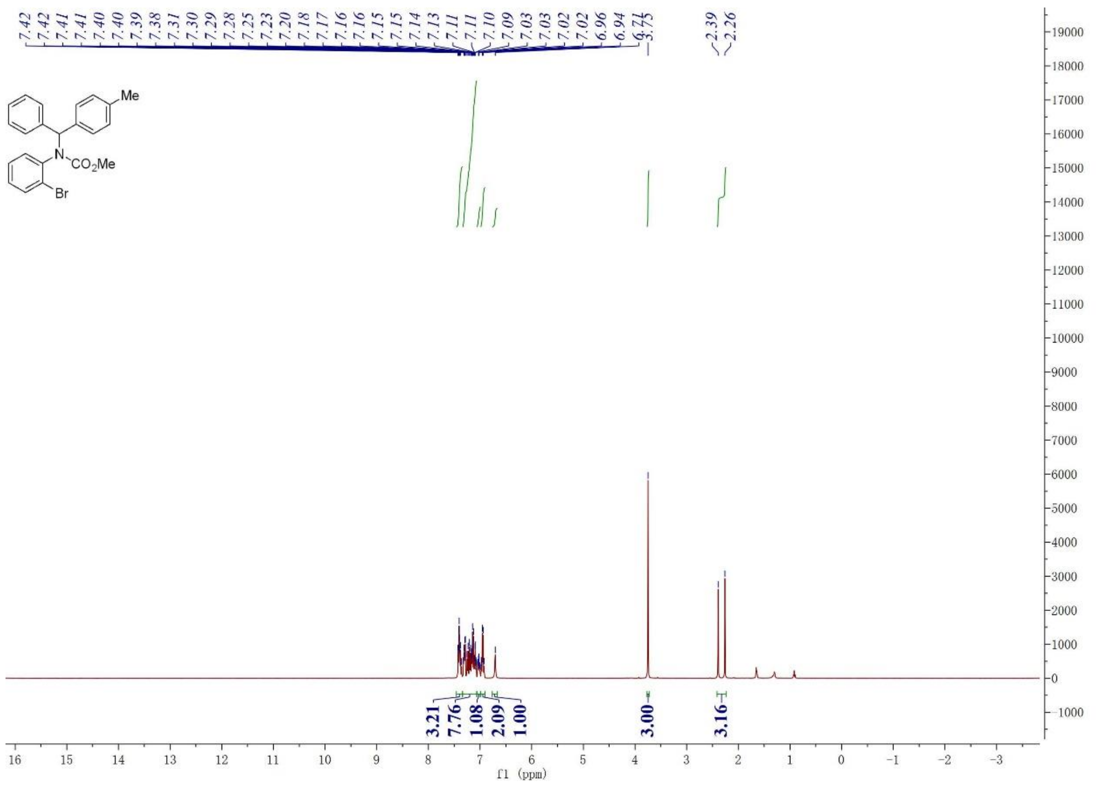

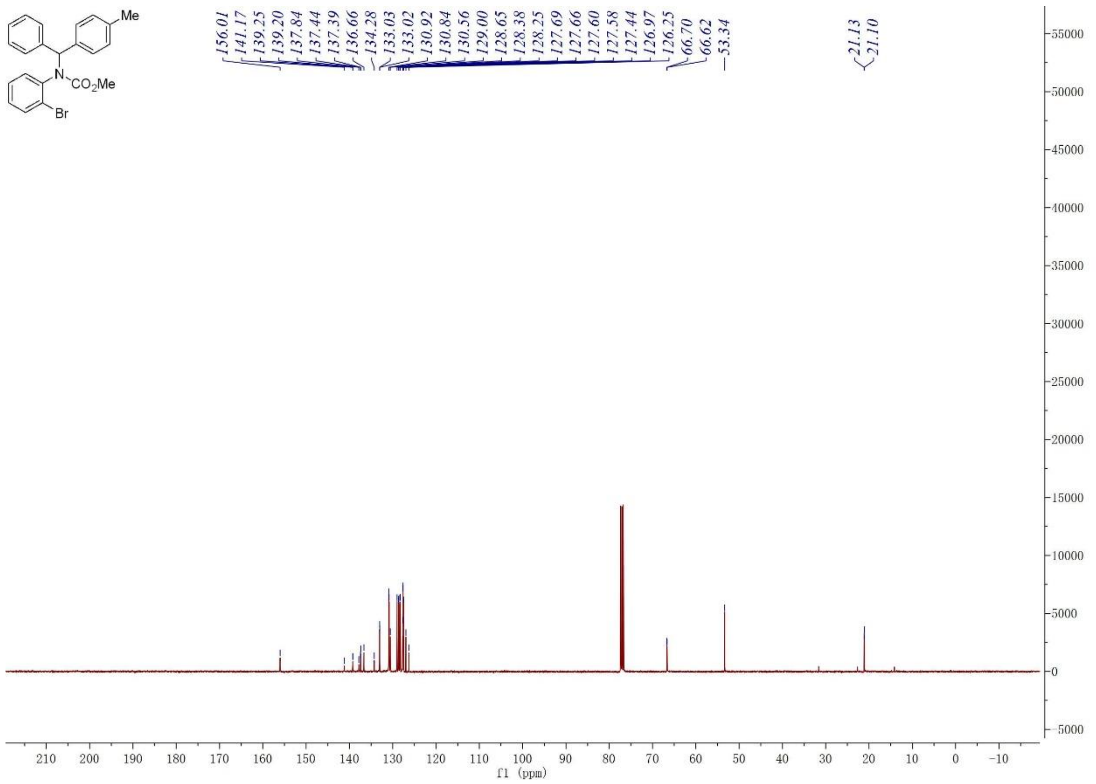

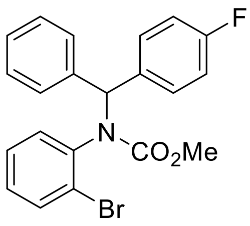

6

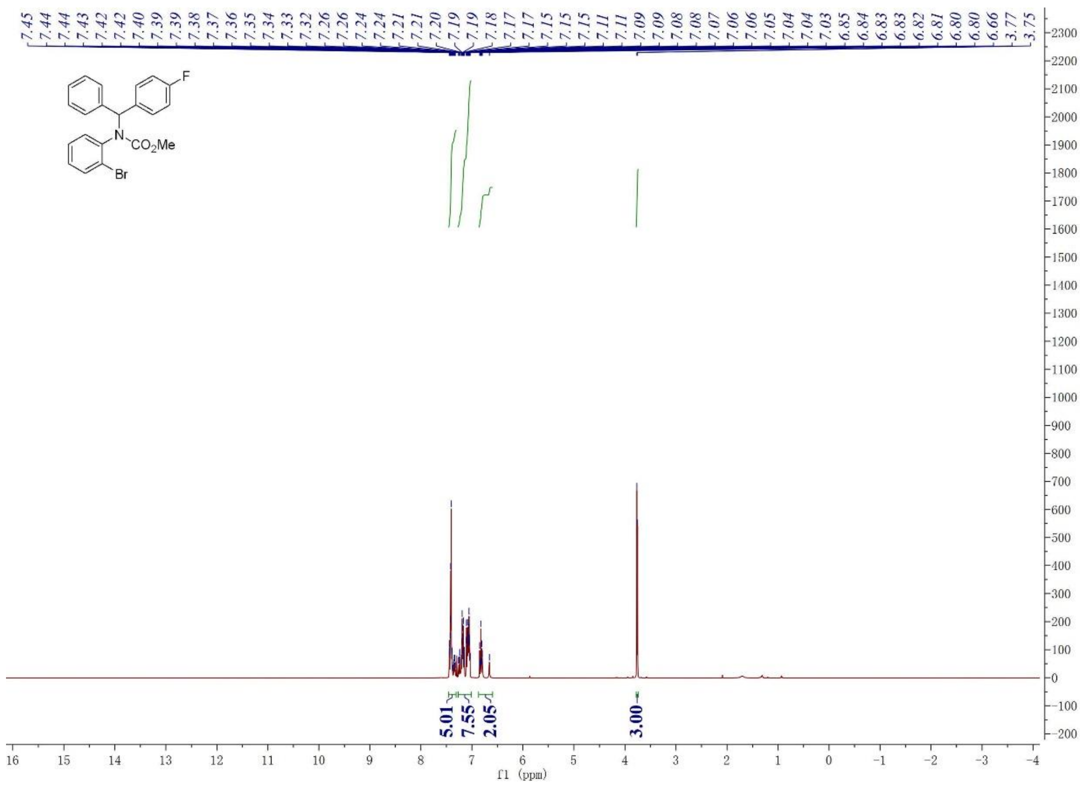

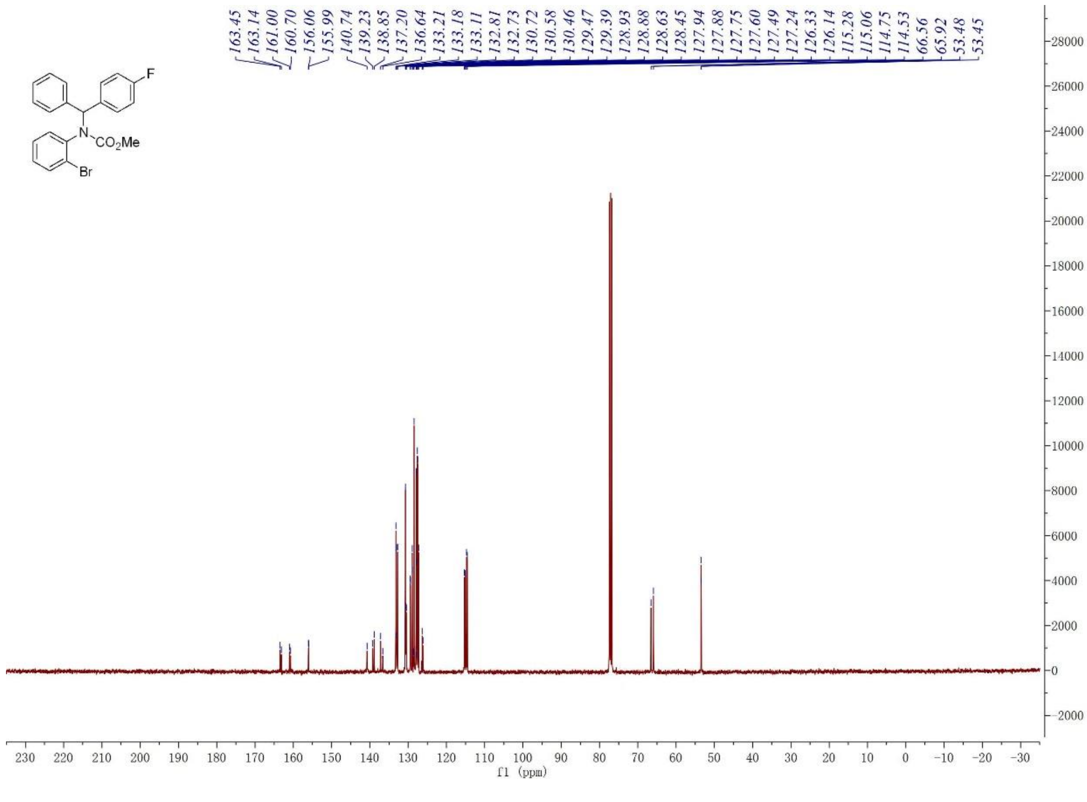

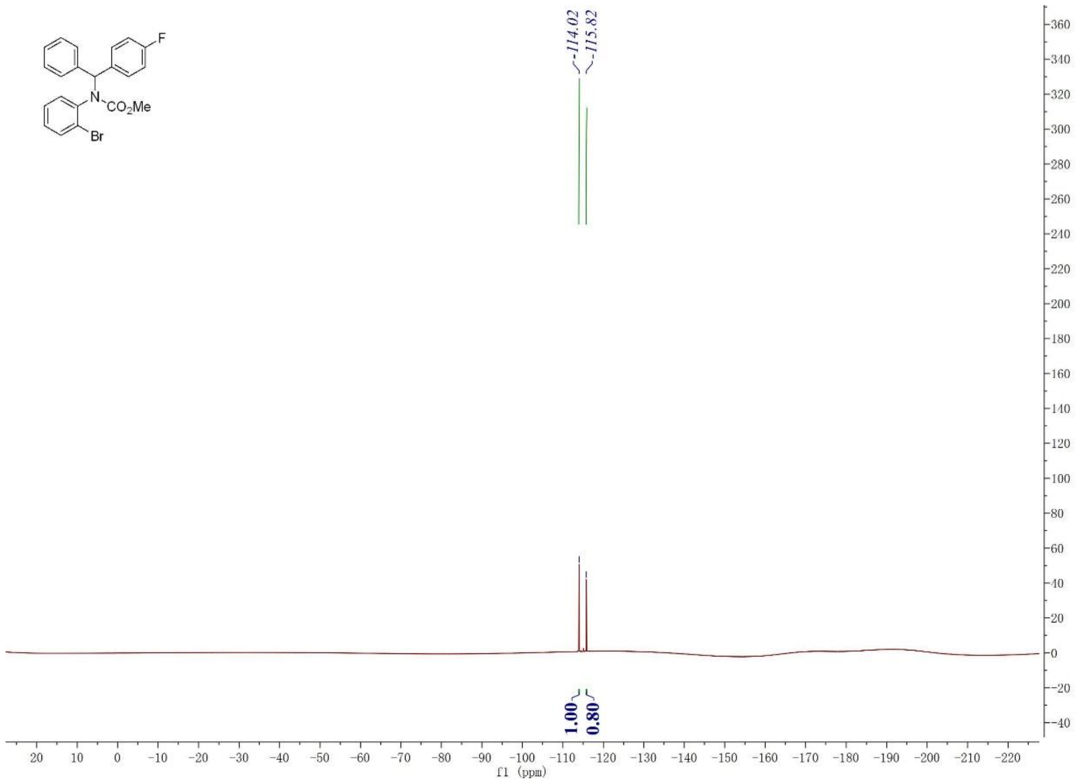

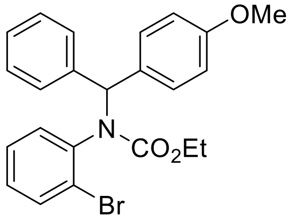

7

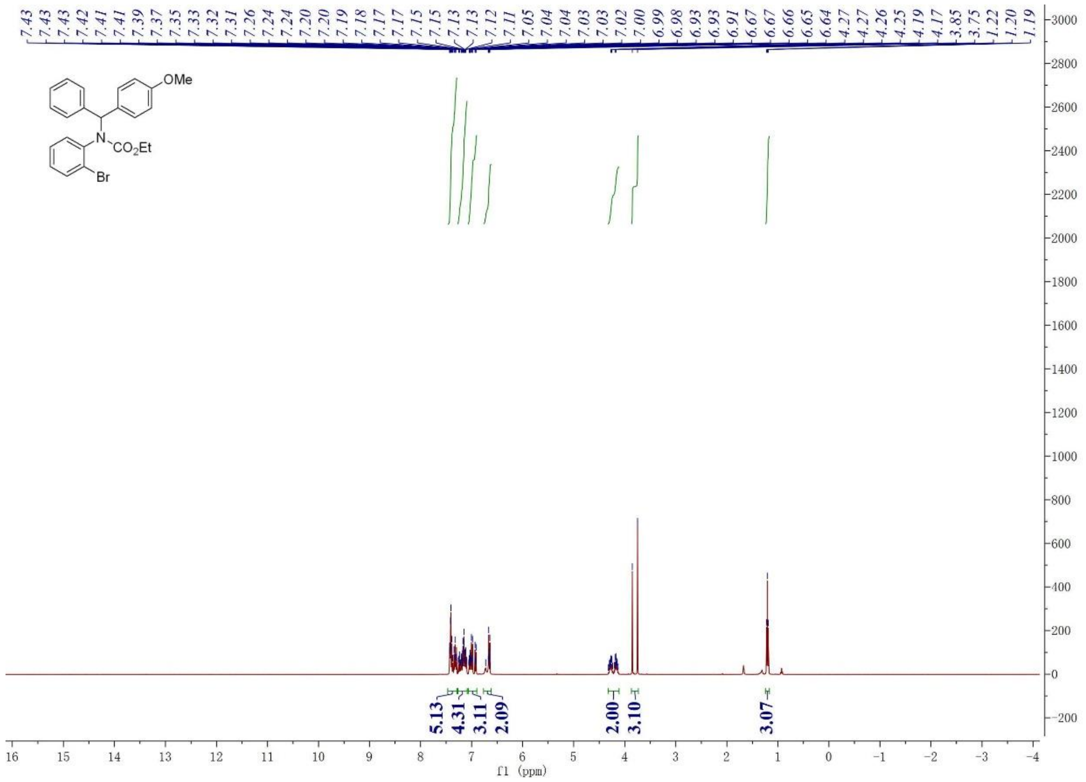
7.43 7.43 7.42 7.41 7.41 7.39 7.37 7.35 7.33 7.32 7.31 7.26 7.24 7.24 7.20 7.20 7.19 7.18 7.17 7.17 7.15 7.15 7.13 7.13 7.13 7.12 7.11 7.05 7.04 7.04 7.03 7.03 7.02 7.00 6.99 6.98 6.93 6.93 6.91 6.67 6.67 6.66 6.65 6.64 4.27 4.27 4.26 4.25 4.19 4.17 3.85 3.75 1.22 1.20 1.19
OMe
N
CO2Et
Br
5.13
4.31
3.11
2.09
2.00
3.10
3.07
f1 (ppm)

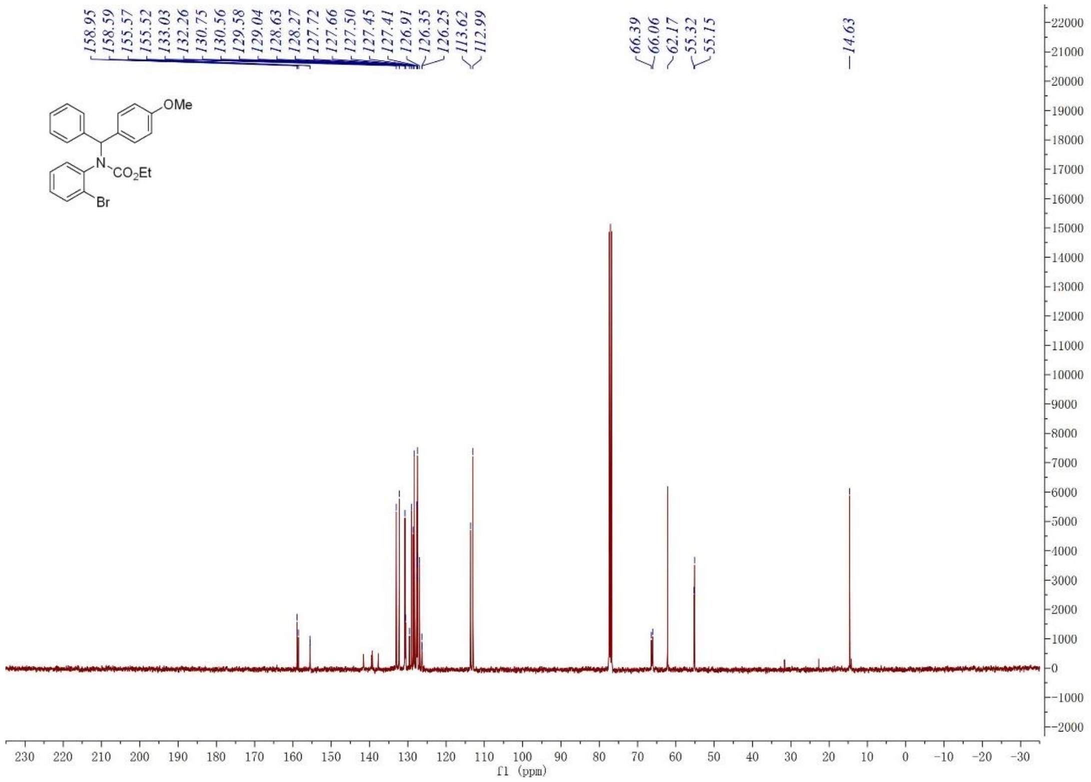
158.95 158.59 155.57 155.52 133.03 132.26 130.75 130.56 129.58 129.04 128.63 128.27 127.72 127.66 127.50 127.45 127.41 126.91 126.35 126.25 113.62 112.99
66.39 66.06 62.17 55.32 55.15
14.63
OMe
N
CO2Et
Br
f1 (ppm)

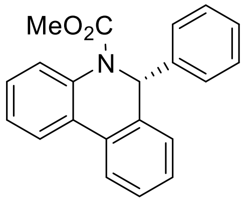
MeO2C
N

2a

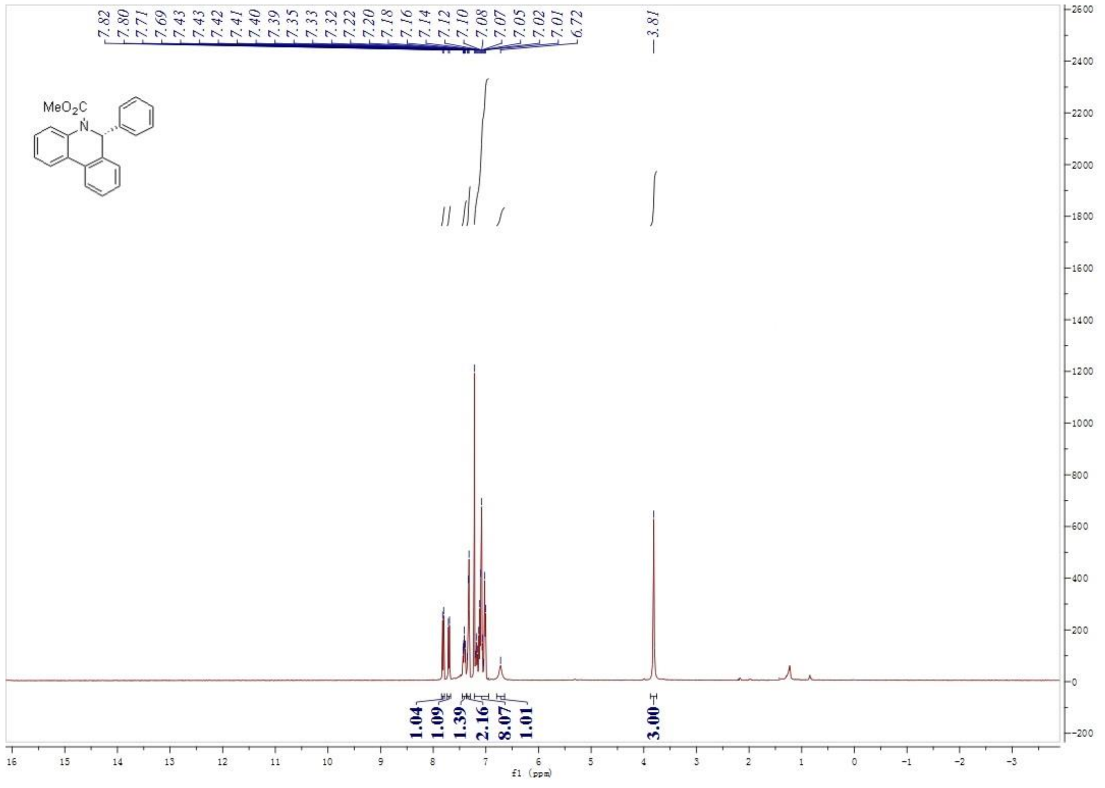
MeO2C
N
7.82 7.80 7.71 7.69 7.43 7.43 7.42 7.41 7.40 7.39 7.35 7.33 7.32 7.22 7.20 7.18 7.16 7.14 7.12 7.10 7.08 7.07 7.05 7.02 7.01 6.72
3.81
1.04 1.09 1.39 2.16 8.07 1.01
3.00
f1 (ppm)

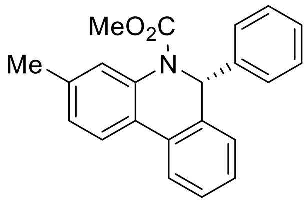
MeO2C
Me
N

2b

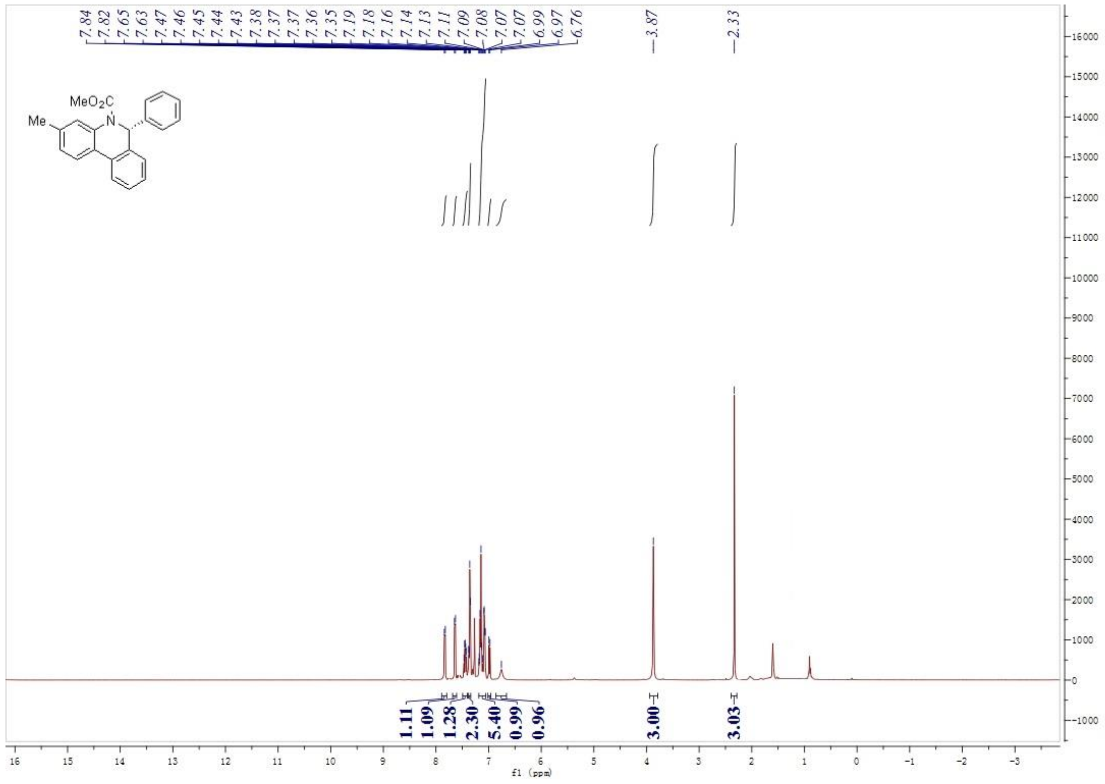
MeO2C
Me
N
7.84 7.82 7.65 7.63 7.47 7.46 7.45 7.44 7.43 7.38 7.37 7.37 7.36 7.35 7.19 7.18 7.16 7.14 7.13 7.11 7.09 7.08 7.07 7.07 6.99 6.97 6.76
3.87
2.33
1.11 1.09 1.28 2.30 5.40 0.99 0.96
3.00
3.03
f1 (ppm)

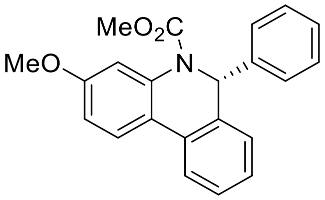

2c

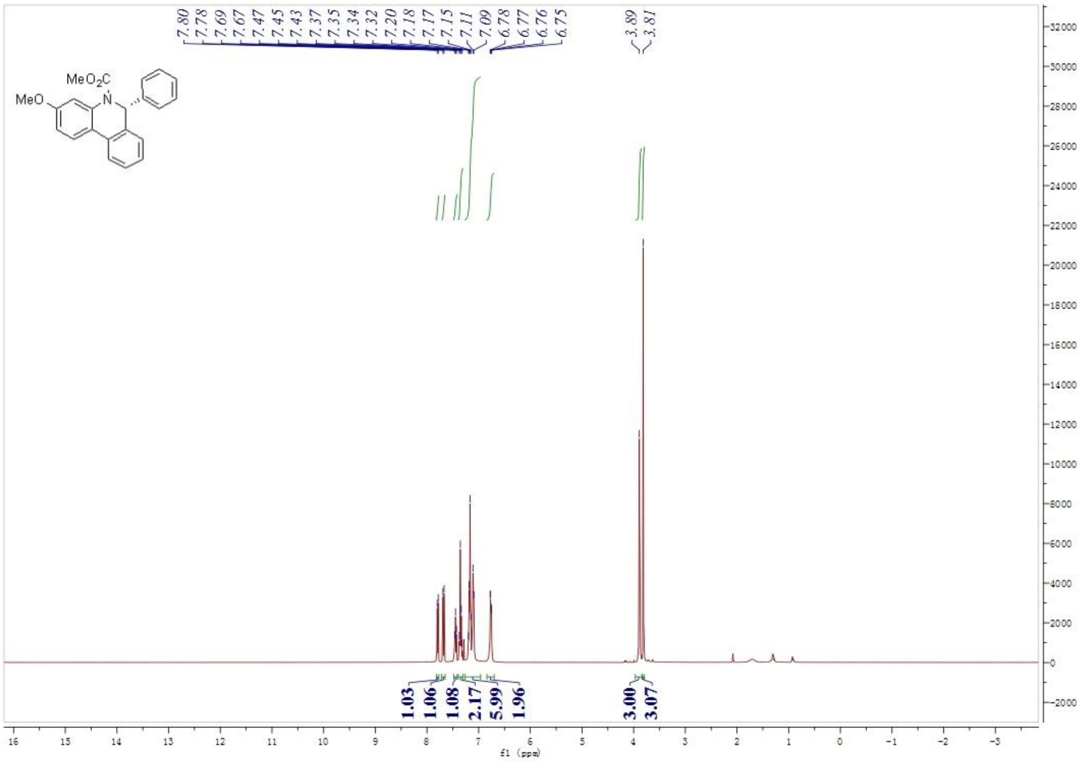

7.80
7.78
7.69
7.67
7.47
7.45
7.43
7.37
7.35
7.34
7.32
7.20
7.18
7.17
7.15
7.11
7.09
6.78
6.77
6.76
6.75
3.89
3.81
1.03
1.06
1.08
2.17
5.99
1.96
3.00
3.07
f1 (ppm)

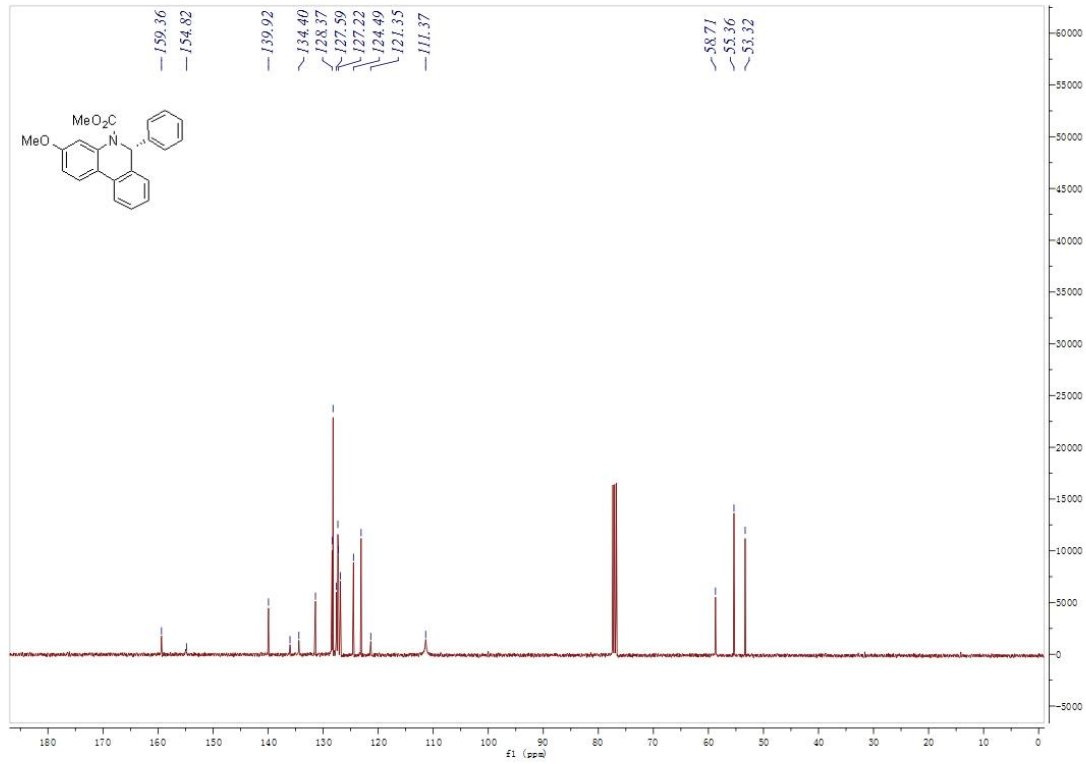
159.36
154.82
139.92
134.40
128.37
127.59
127.22
124.49
121.35
111.37
58.71
55.36
53.32
MeO₂C
MeO
N
f1 (ppm)

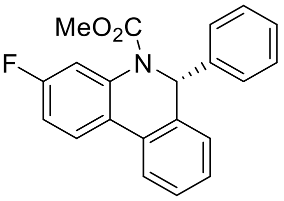

2d

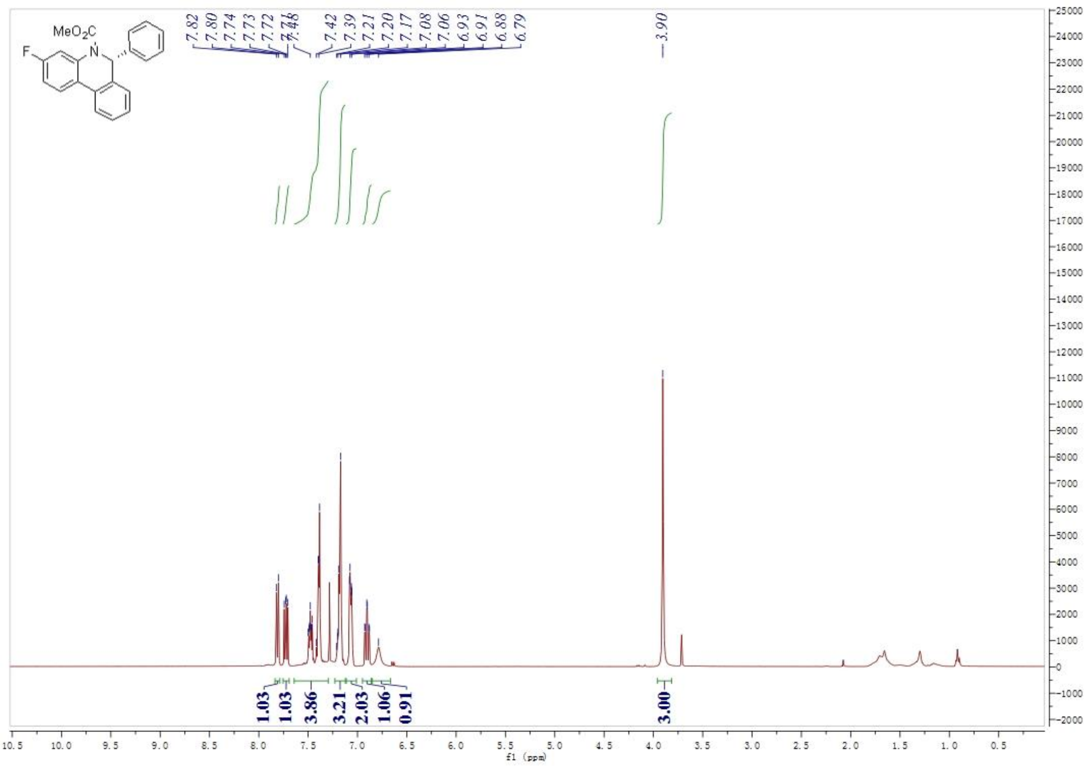
7.82
7.80
7.74
7.73
7.72
7.48
7.42
7.39
7.21
7.20
7.17
7.08
7.06
6.93
6.91
6.88
6.79
3.90
1.03
1.03
3.86
3.21
2.03
1.06
0.91
3.00
f1 (ppm)

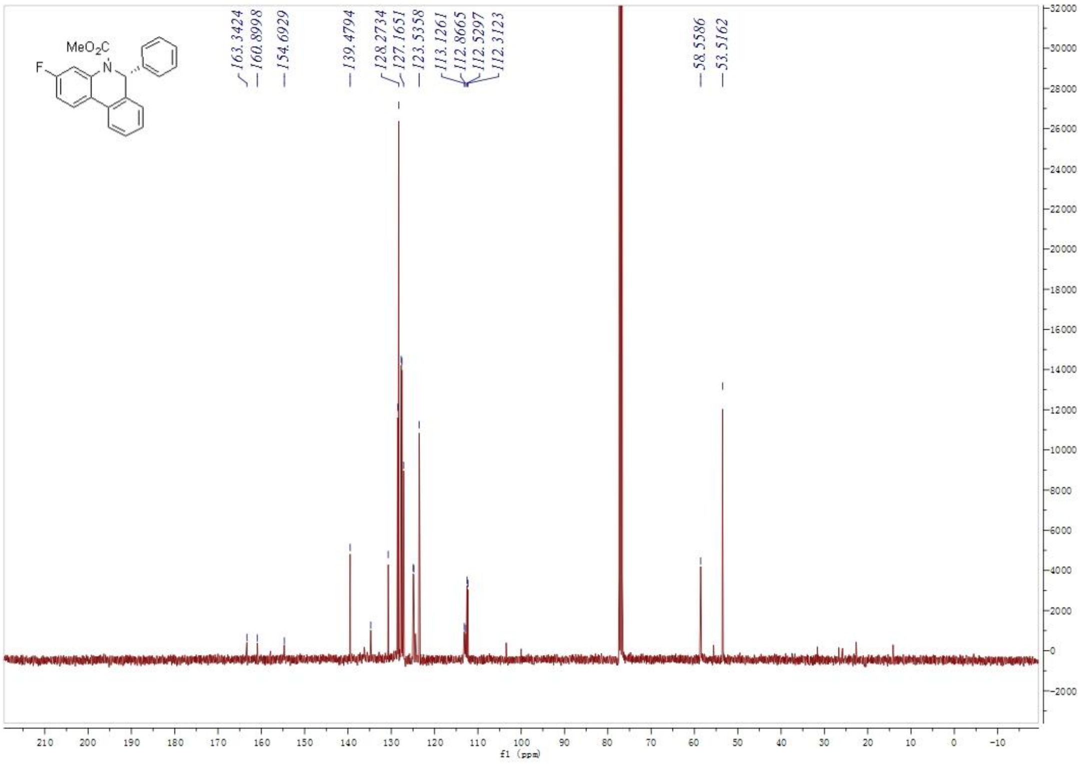
MeO2C
N
F
163.3424
160.8998
154.6929
139.4794
128.2734
127.1651
123.5358
113.1261
112.8665
112.5297
112.3123
58.5586
53.5162
f1 (ppm)

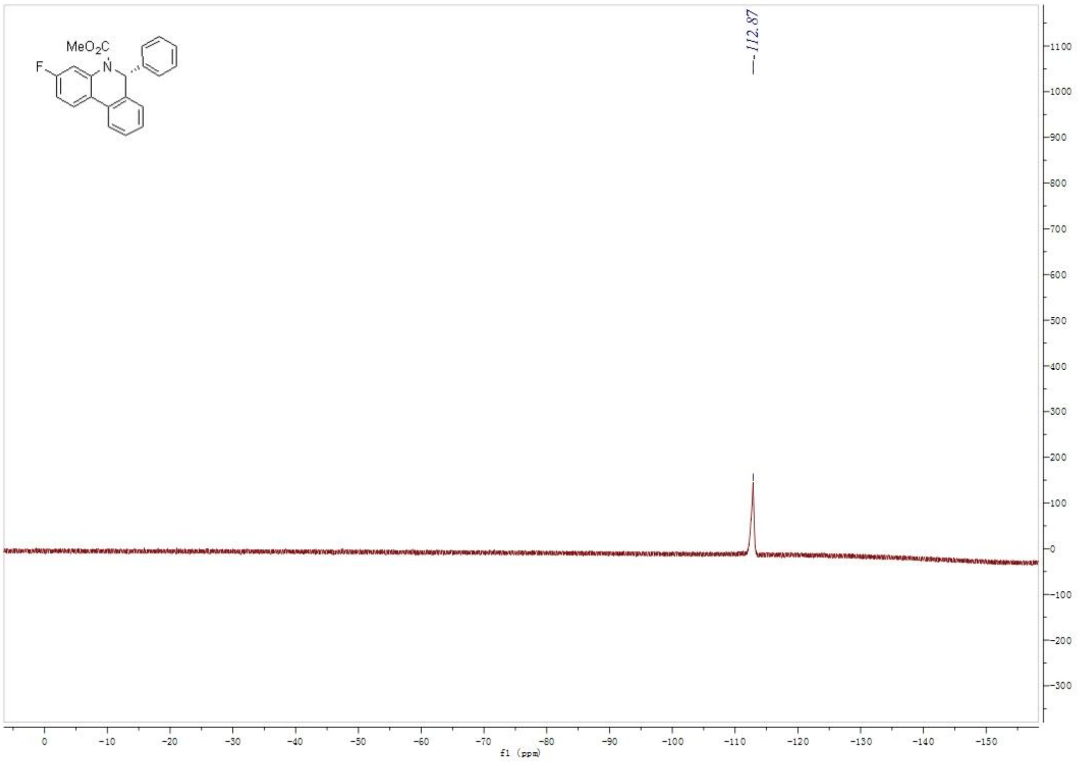
MeO2C
N
F
-112.87
f1 (ppm)

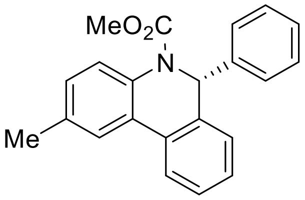
MeO2C
N
Me

2e

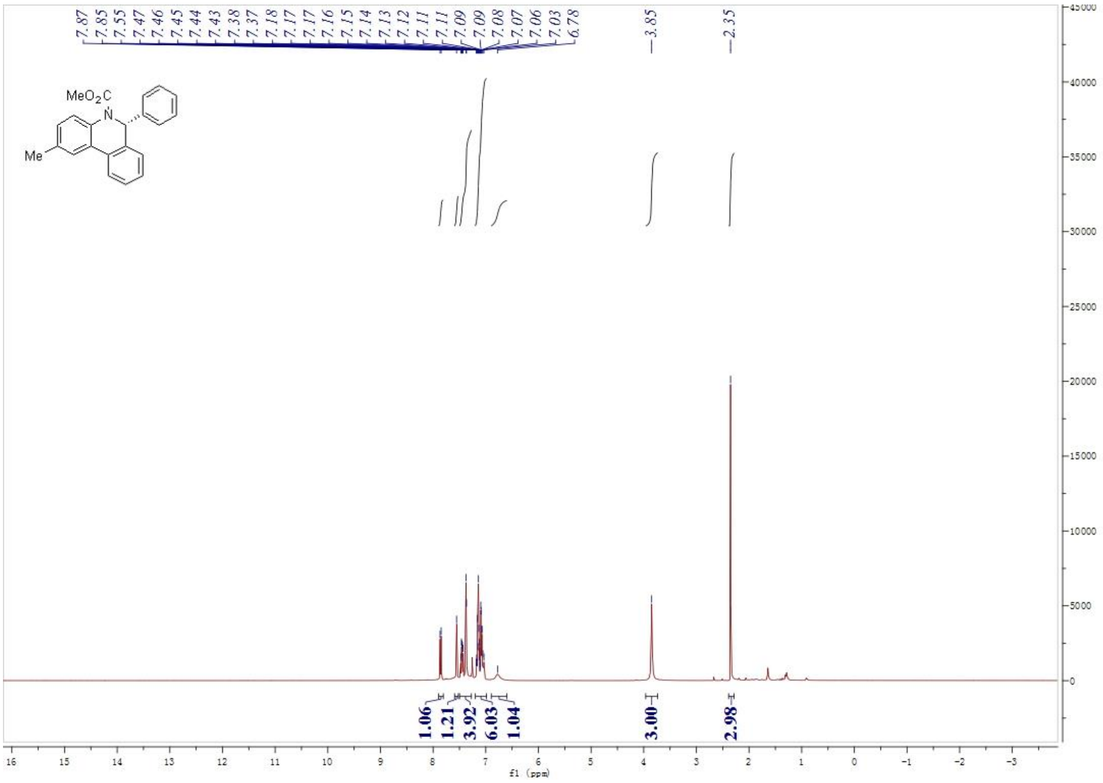
7.87
7.85
7.55
7.47
7.46
7.45
7.44
7.43
7.38
7.37
7.18
7.17
7.17
7.16
7.15
7.14
7.13
7.12
7.11
7.11
7.09
7.09
7.08
7.07
7.06
7.03
6.78
3.85
2.35
MeO2C
N
Me
1.06
1.21
3.92
6.03
1.04
3.00
2.98
f1 (ppm)

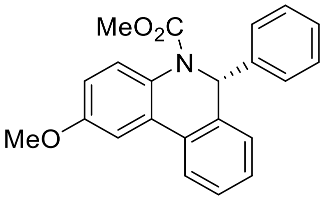
MeO2C
N
MeO

2f

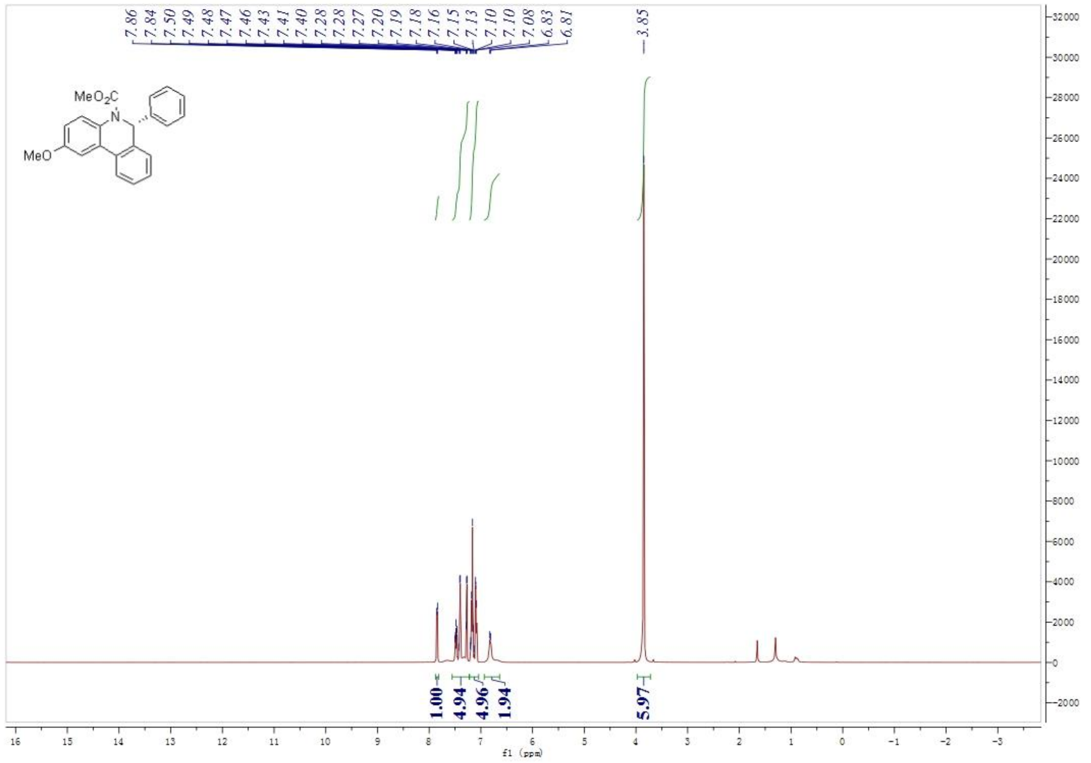

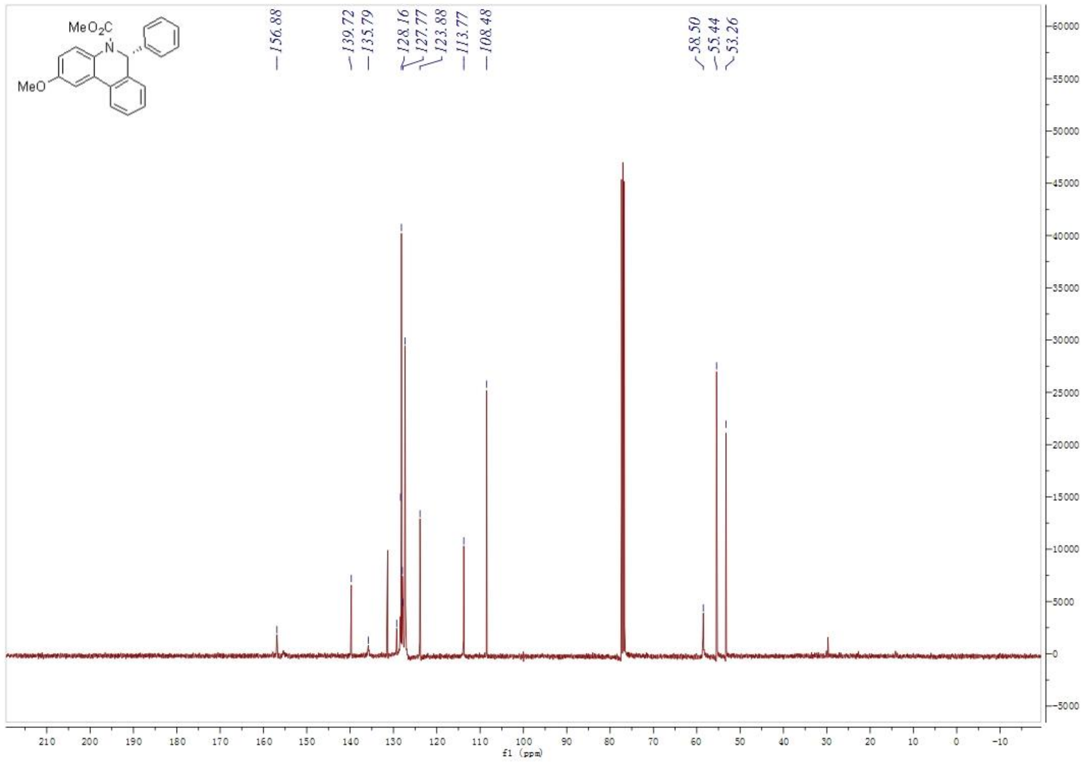

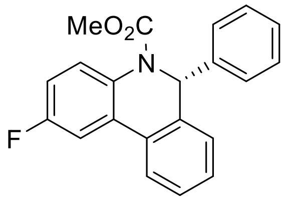

2g

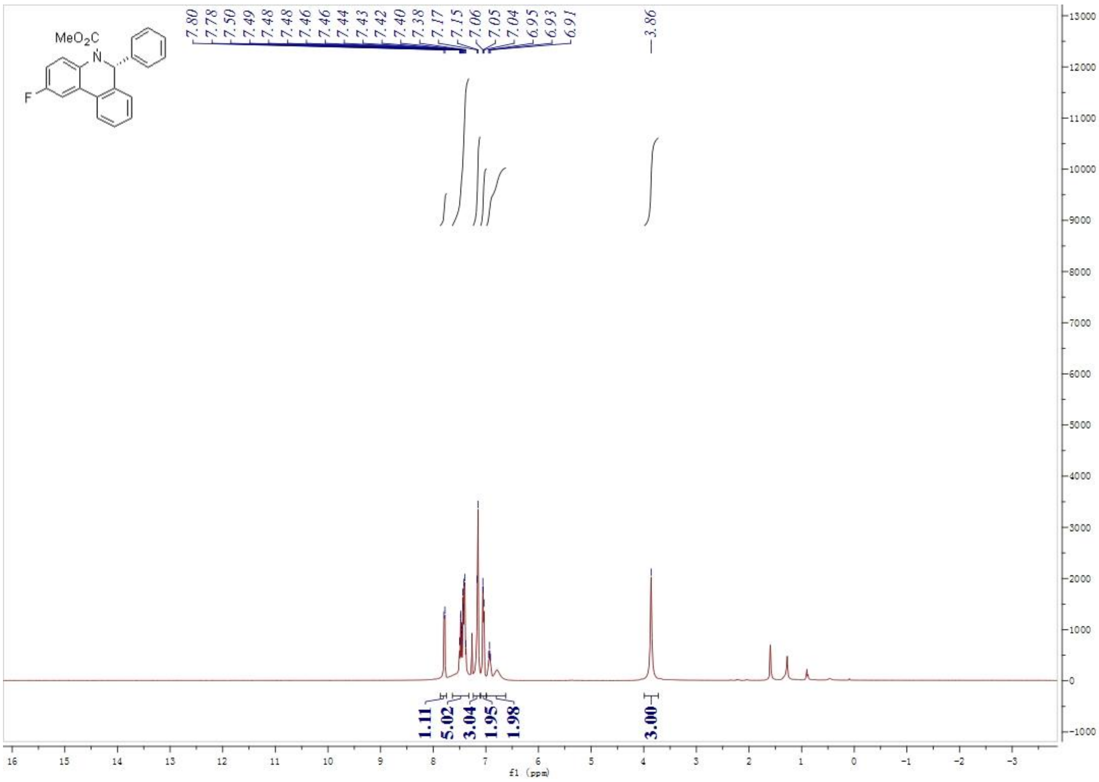
MeO2C
N
F
7.80
7.78
7.50
7.49
7.48
7.48
7.46
7.46
7.44
7.43
7.42
7.40
7.38
7.17
7.15
7.06
7.05
7.04
6.95
6.93
6.91
3.86
1.11
5.02
3.04
1.95
1.98
3.00
f1 (ppm)

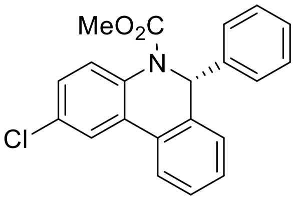
MeO2C
N
Cl

2h

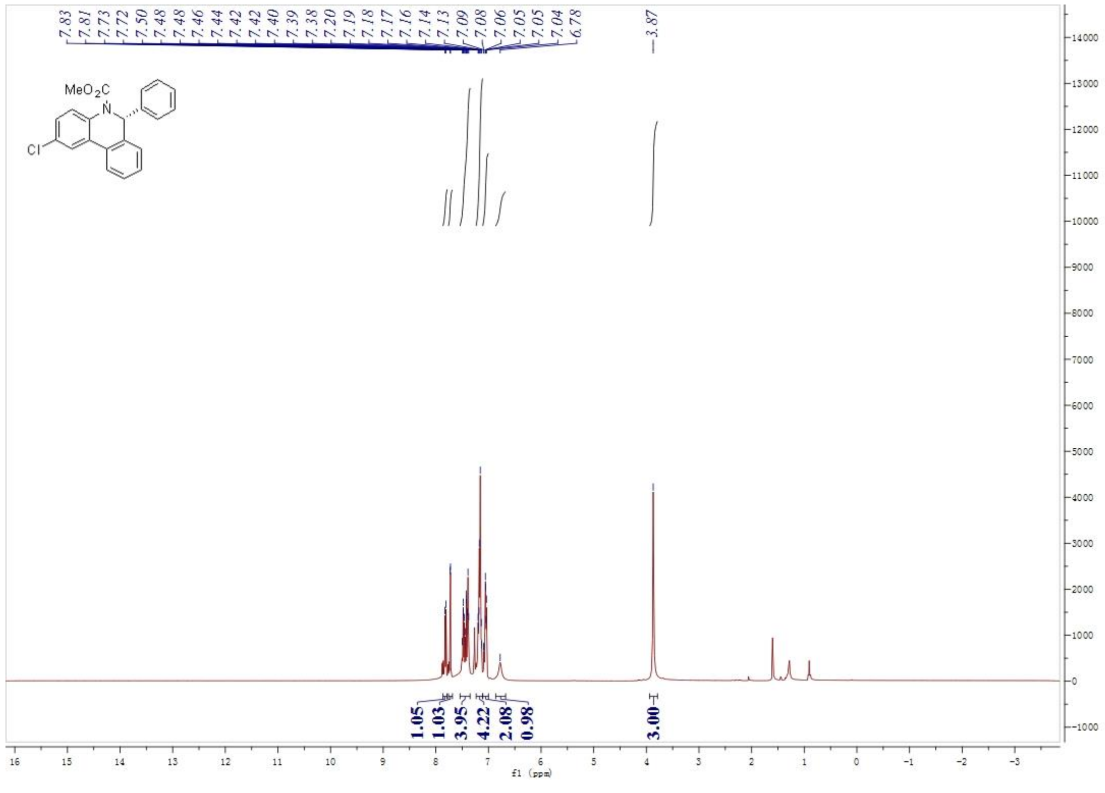
7.83
7.81
7.73
7.72
7.50
7.48
7.48
7.46
7.44
7.42
7.42
7.40
7.39
7.38
7.20
7.19
7.18
7.17
7.16
7.14
7.13
7.09
7.08
7.06
7.05
7.05
7.04
6.78
3.87
MeO2C
N
Cl
1.05
1.03
3.95
4.22
2.08
0.98
3.00
f1 (ppm)

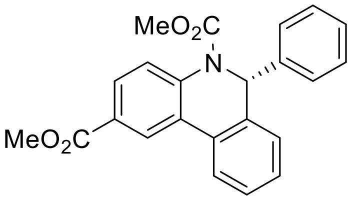

2i

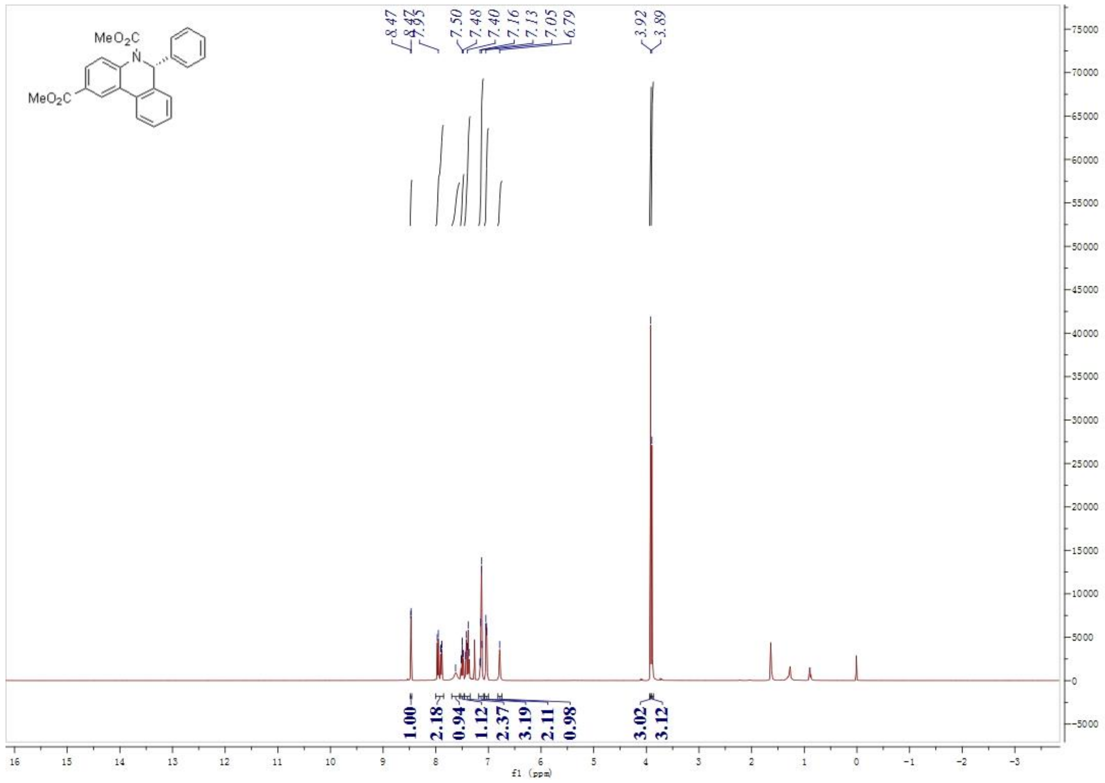

8.47
8.47
7.95
7.50
7.48
7.40
7.16
7.13
7.05
6.79
3.92
3.89
1.00
2.18
0.94
1.12
2.37
3.19
2.11
0.98
3.02
3.12
f1 (ppm)
MeO2C
N
MeO2C

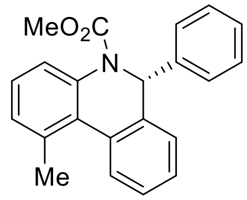

MeO2C
N
Me

2j

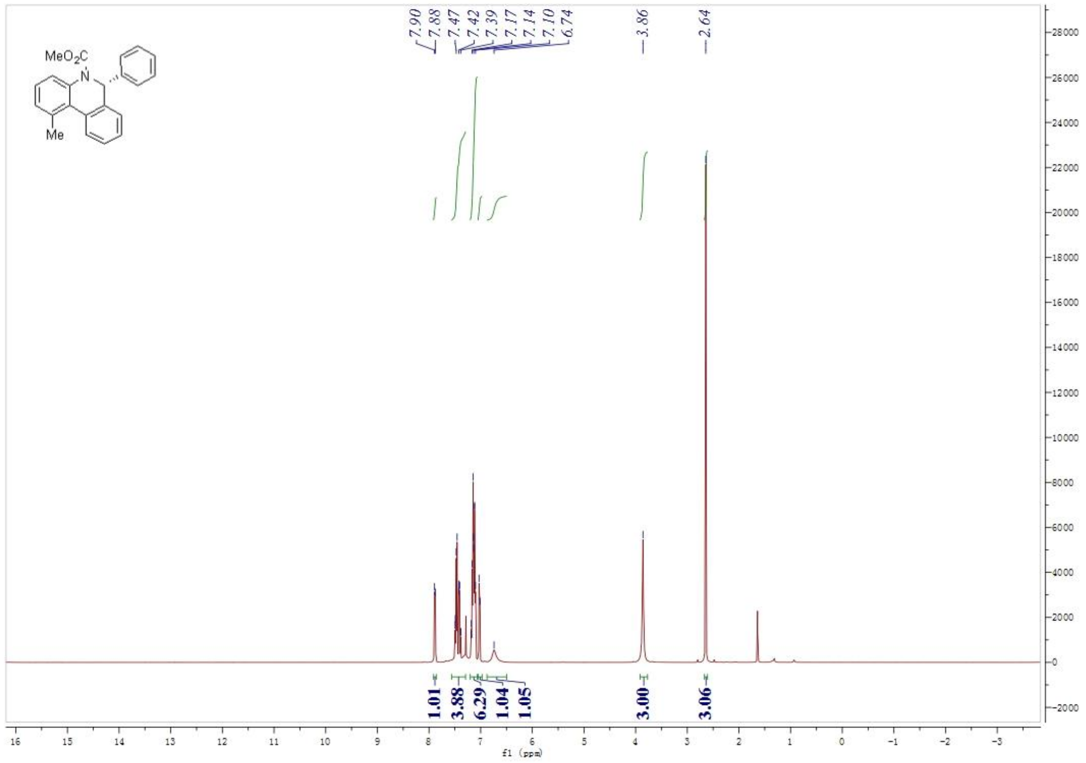

MeO2C
Me
7.90
7.88
7.47
7.42
7.39
7.17
7.14
7.10
6.74
3.86
2.64
1.01
3.88
6.29
1.04
1.05
3.00
3.06
f1 (ppm)

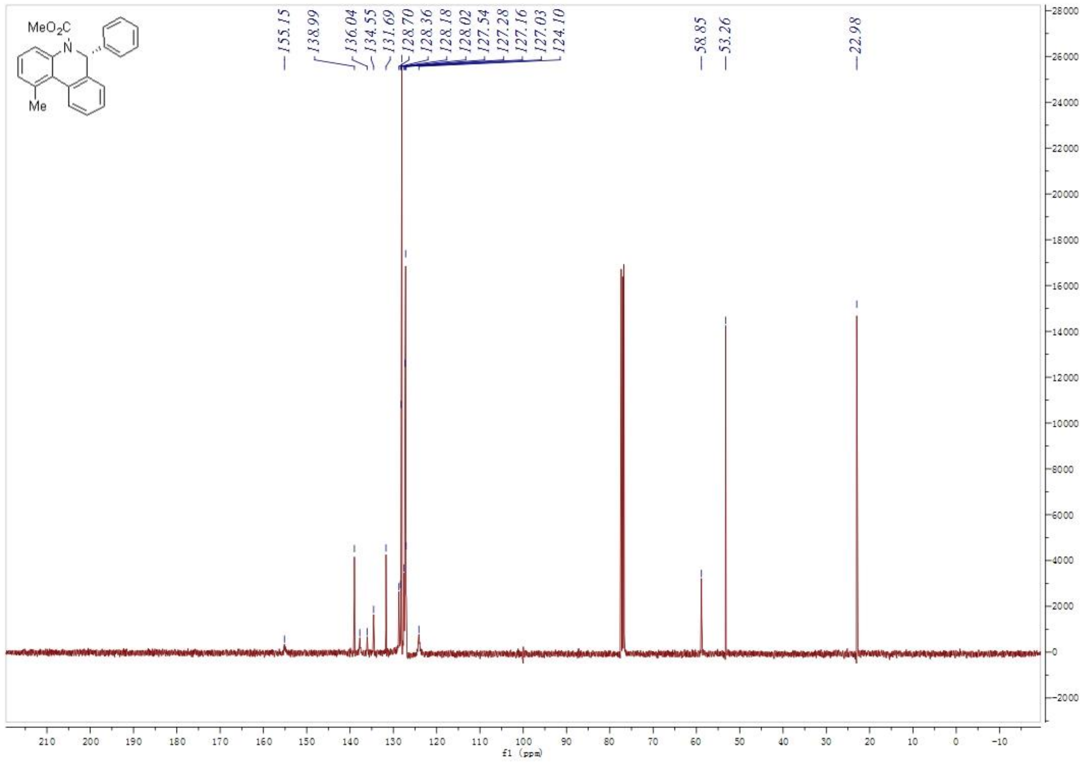

MeO2C
Me
155.15
138.99
136.04
134.55
131.69
128.70
128.36
128.18
128.02
127.54
127.28
127.16
127.03
124.10
58.85
53.26
22.98
f1 (ppm)

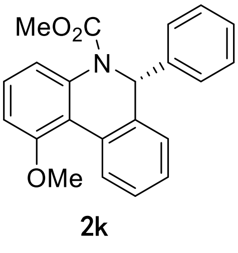

2k

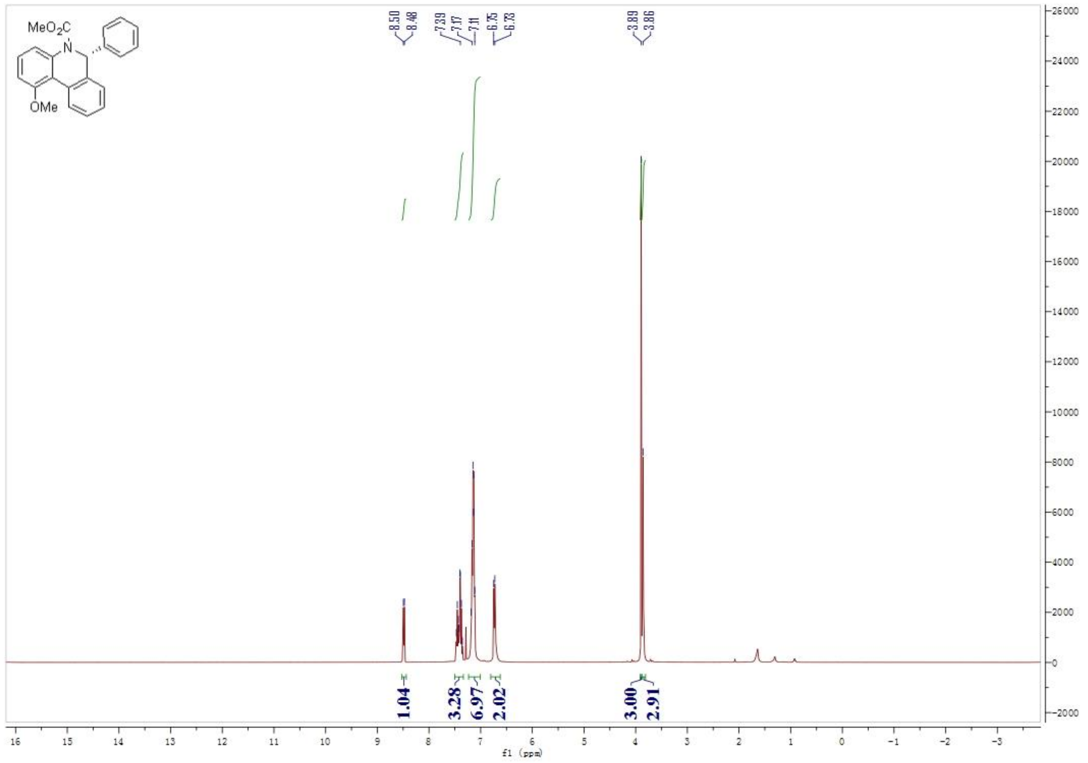
MeO2C
N
OMe
8.50
8.48
7.39
7.17
7.11
6.75
6.73
3.89
3.86
1.04
3.28
6.97
2.02
3.00
2.91
f1 (ppm)

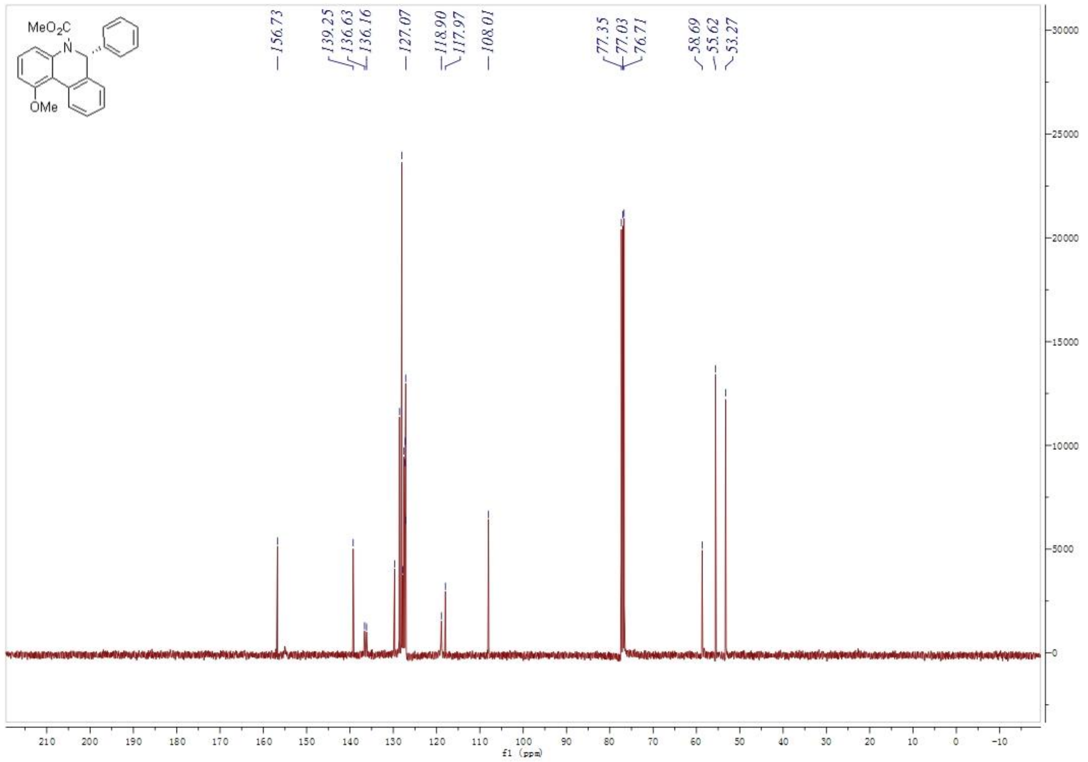
MeO2C
N
OMe
156.73
139.25
136.63
136.16
127.07
118.90
117.97
108.01
77.35
77.03
76.71
58.69
55.62
53.27
f1 (ppm)

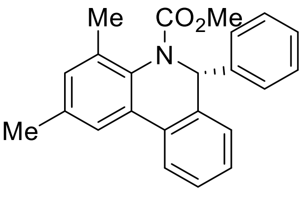
Me
CO2Me
N
Me

2l

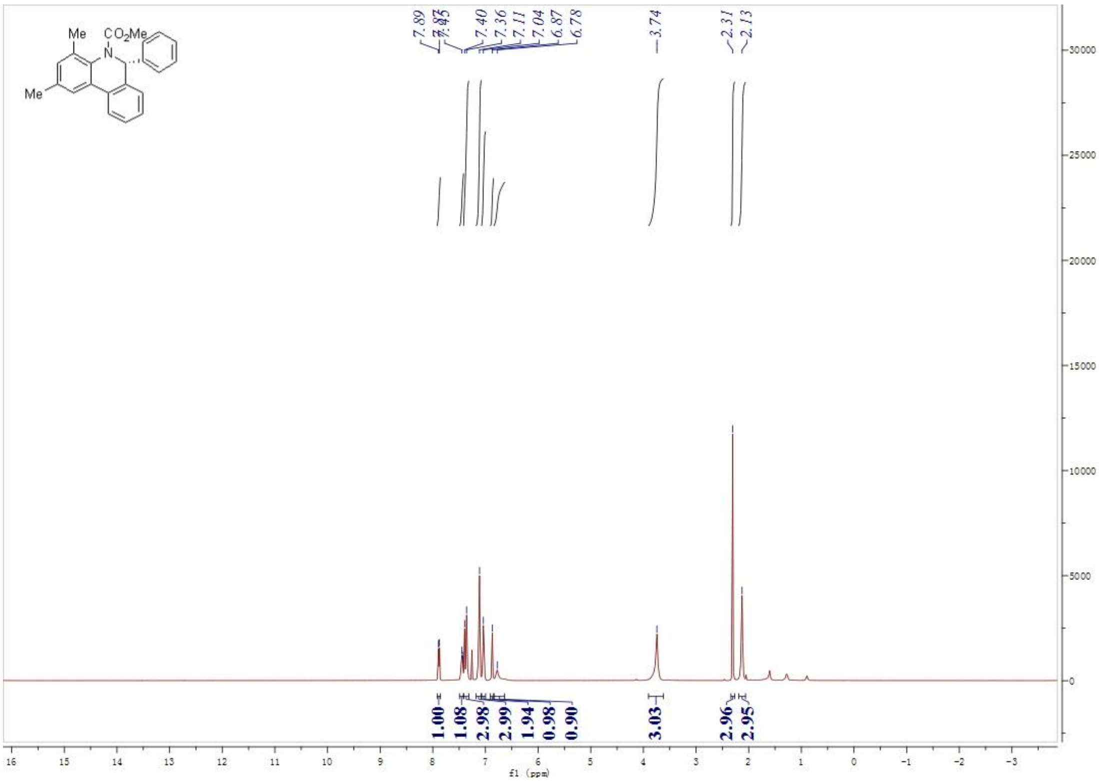
Me
CO2Me
N
Me
7.89
7.40
7.36
7.11
7.04
6.87
6.78
3.74
2.31
2.13
1.00
1.08
2.98
2.99
1.94
0.98
0.90
3.03
2.96
2.95
f1 (ppm)

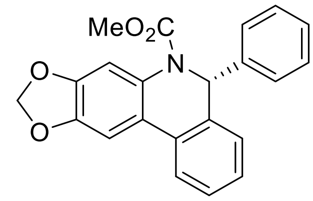
MeO2C
N
O
O

2m

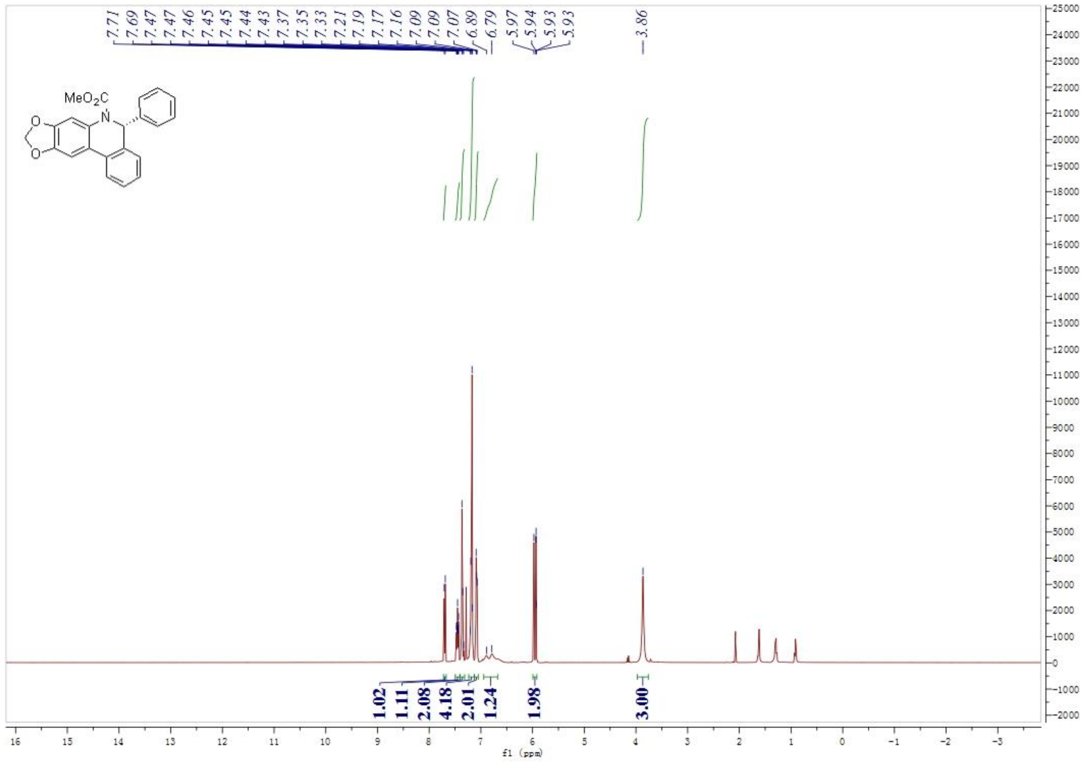
7.71
7.69
7.47
7.47
7.46
7.45
7.45
7.44
7.43
7.37
7.35
7.33
7.21
7.19
7.17
7.16
7.09
7.09
7.07
6.89
6.79
5.97
5.94
5.93
5.93
3.86
MeO2C
N
O
O
1.02
1.11
2.08
4.18
2.01
1.24
1.98
3.00
f1 (ppm)

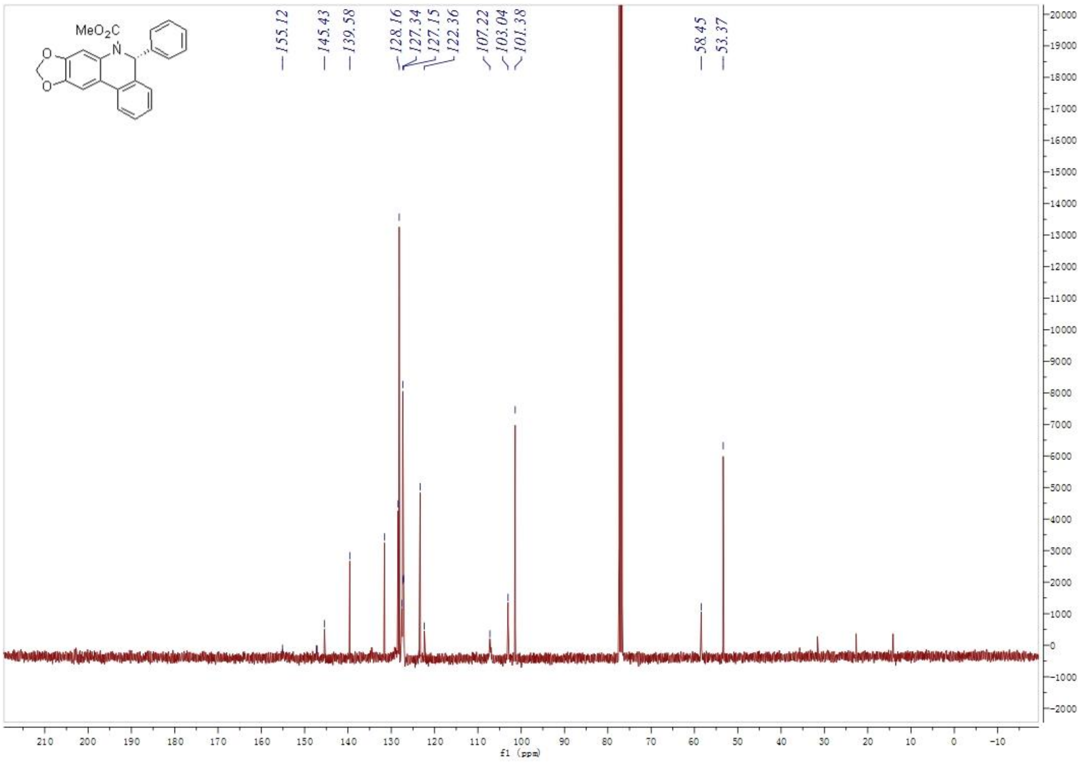
MeO2C
N
O
O
155.12
145.43
139.58
128.16
127.34
127.15
122.36
107.22
103.04
101.38
58.45
53.37
20000
19000
18000
17000
16000
15000
14000
13000
12000
11000
10000
9000
8000
7000
6000
5000
4000
3000
2000
1000
0
-1000
-2000
210
200
190
180
170
160
150
140
130
120
110
100
90
80
70
60
50
40
30
20
10
0
-10
f1 (ppm)

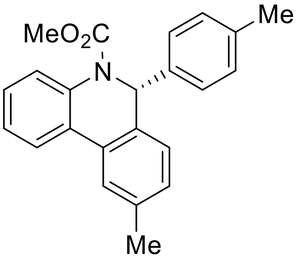
MeO2C
N
Me
Me

2n

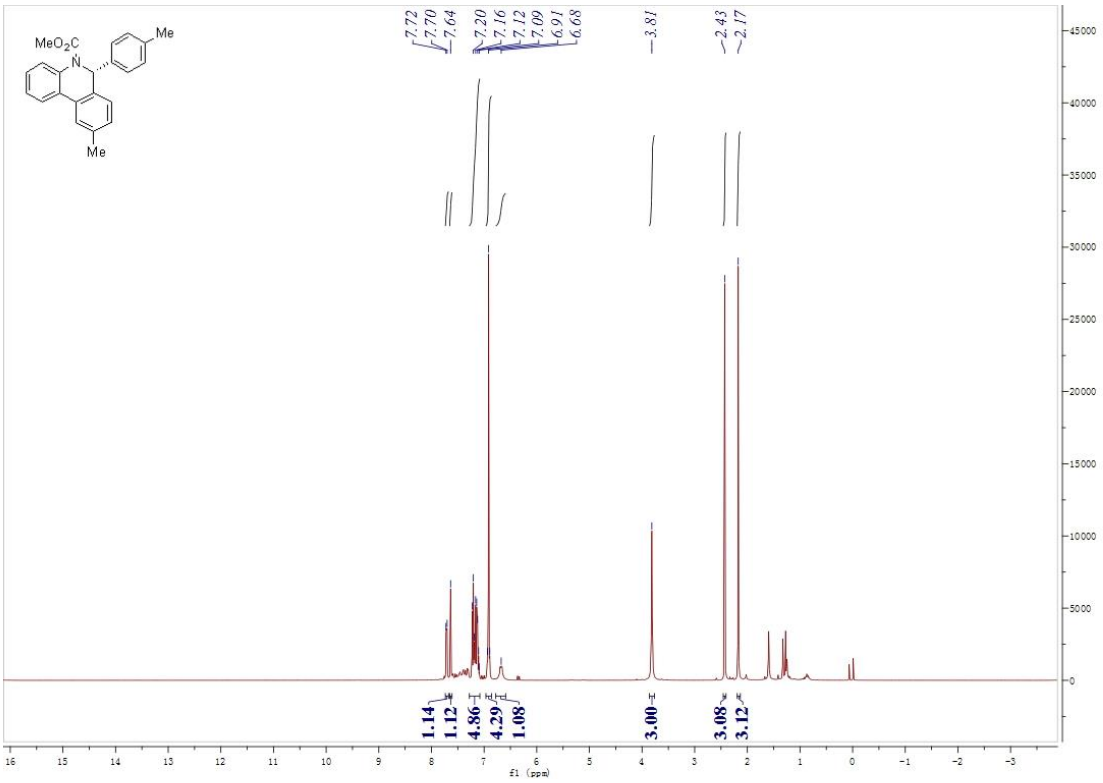
7.72
7.70
7.64
7.20
7.16
7.12
7.09
6.91
6.68
3.81
2.43
2.17
1.14
1.12
4.86
4.29
1.08
3.00
3.08
3.12
f1 (ppm)

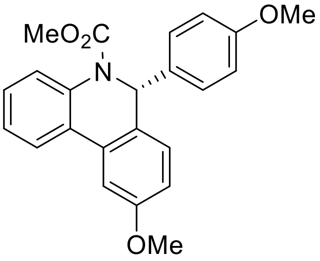

2o

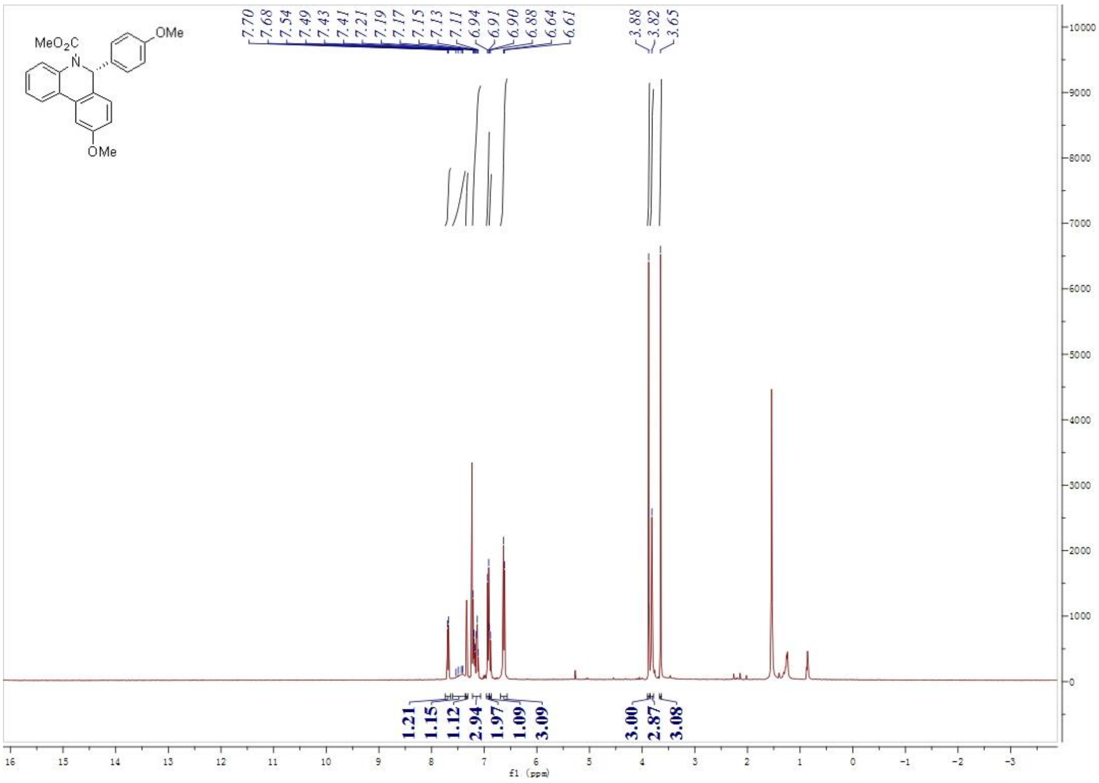
MeO2C
OMe
N
OMe
7.70
7.68
7.54
7.49
7.43
7.41
7.21
7.19
7.17
7.15
7.13
7.11
6.94
6.91
6.90
6.88
6.64
6.61
3.88
3.82
3.65
1.21
1.15
1.12
2.94
1.97
1.09
3.09
3.00
2.87
3.08
f1 (ppm)

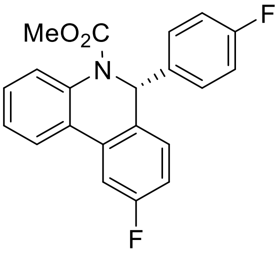
MeO2C
N
F
F

2p

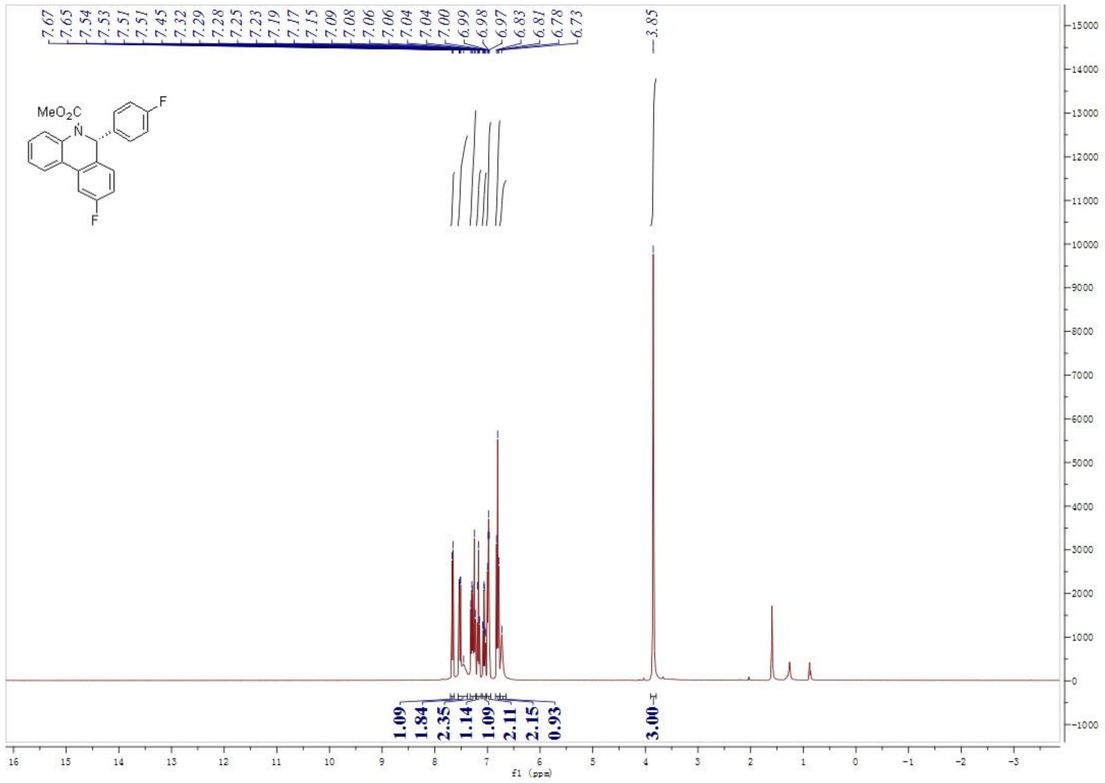
7.67
7.65
7.54
7.53
7.51
7.51
7.45
7.32
7.29
7.28
7.25
7.23
7.19
7.17
7.15
7.09
7.08
7.06
7.06
7.04
7.04
7.00
6.99
6.98
6.97
6.83
6.81
6.78
6.73
3.85
MeO2C
N
F
F
1.09
1.84
2.35
1.14
1.09
2.11
2.15
0.93
3.00
f1 (ppm)

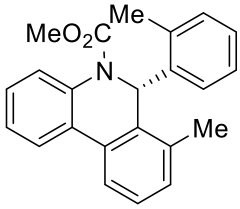

2q

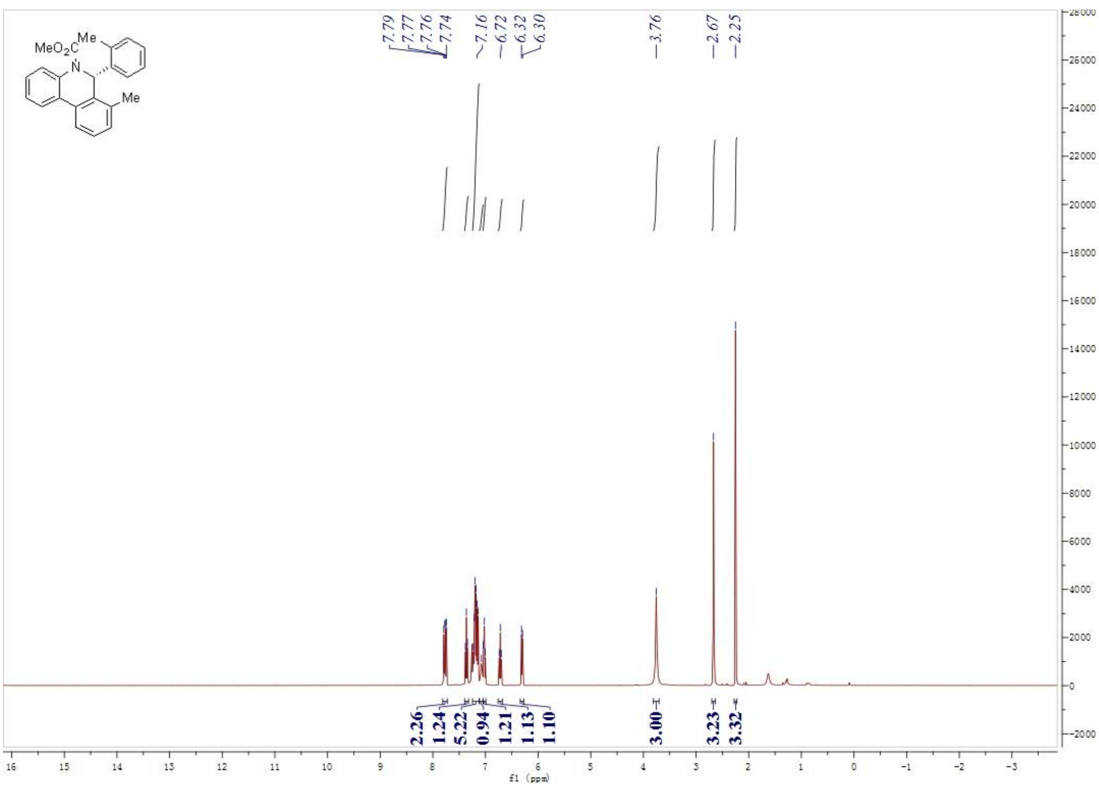
MeO2C
Me
N
Me
7.79
7.77
7.76
7.74
7.16
6.72
6.32
6.30
3.76
2.67
2.25
2.26
1.24
5.22
0.94
1.21
1.13
1.10
3.00
3.23
3.32
f1 (ppm)

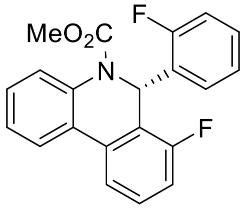
MeO2C
F
N
F

2r

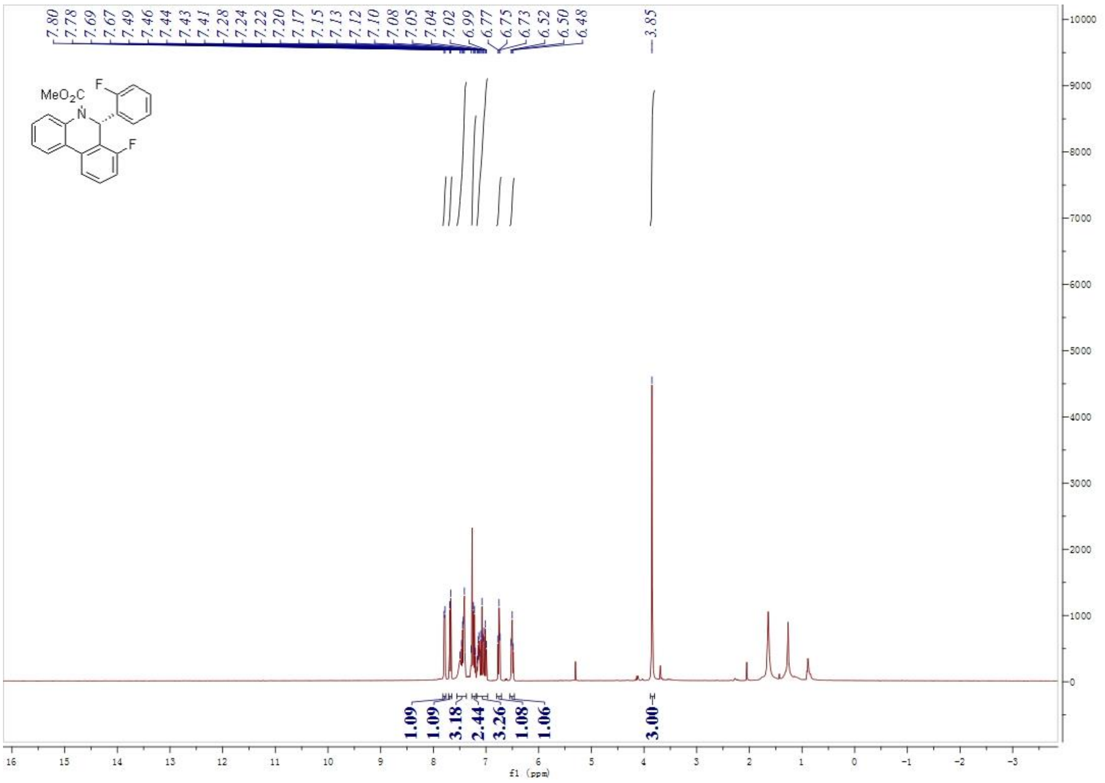

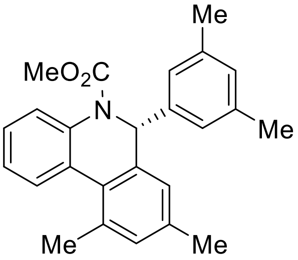

2s

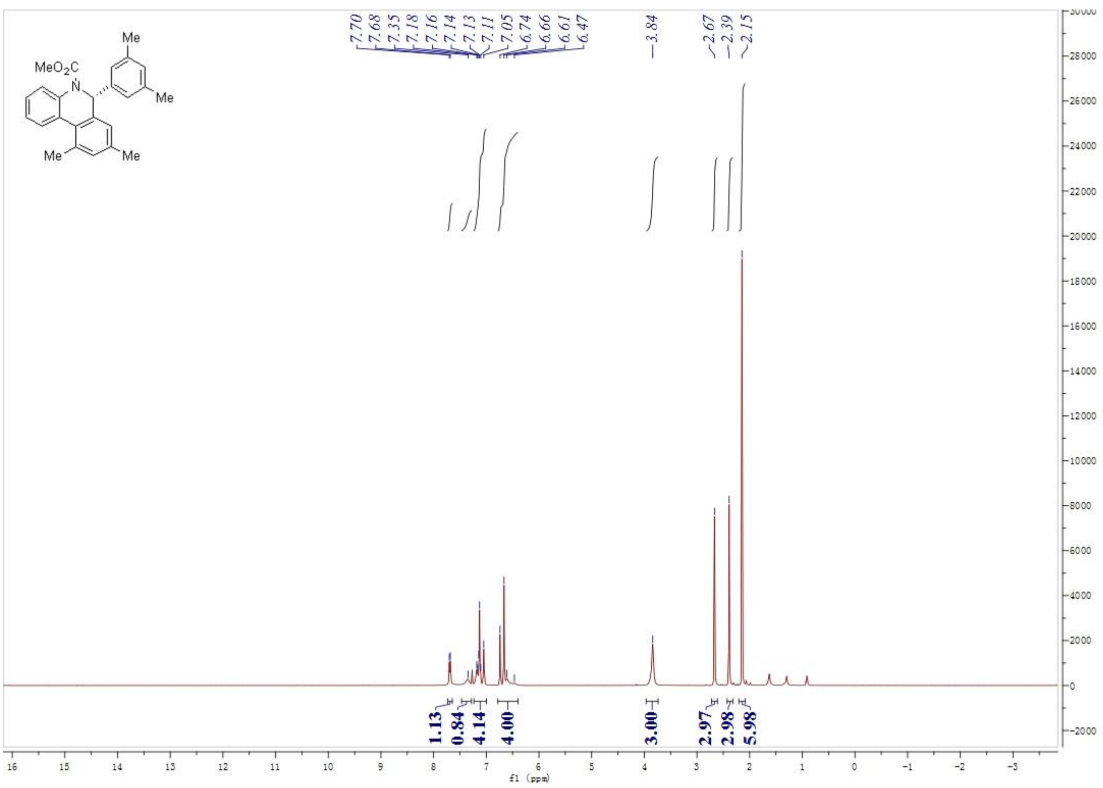

MeO2C
N
Me
Me
Me
Me
7.70
7.68
7.35
7.18
7.16
7.14
7.13
7.11
7.05
6.74
6.66
6.61
6.47
3.84
2.67
2.39
2.15
1.13
0.84
4.14
4.00
3.00
2.97
2.98
5.98
f1 (ppm)

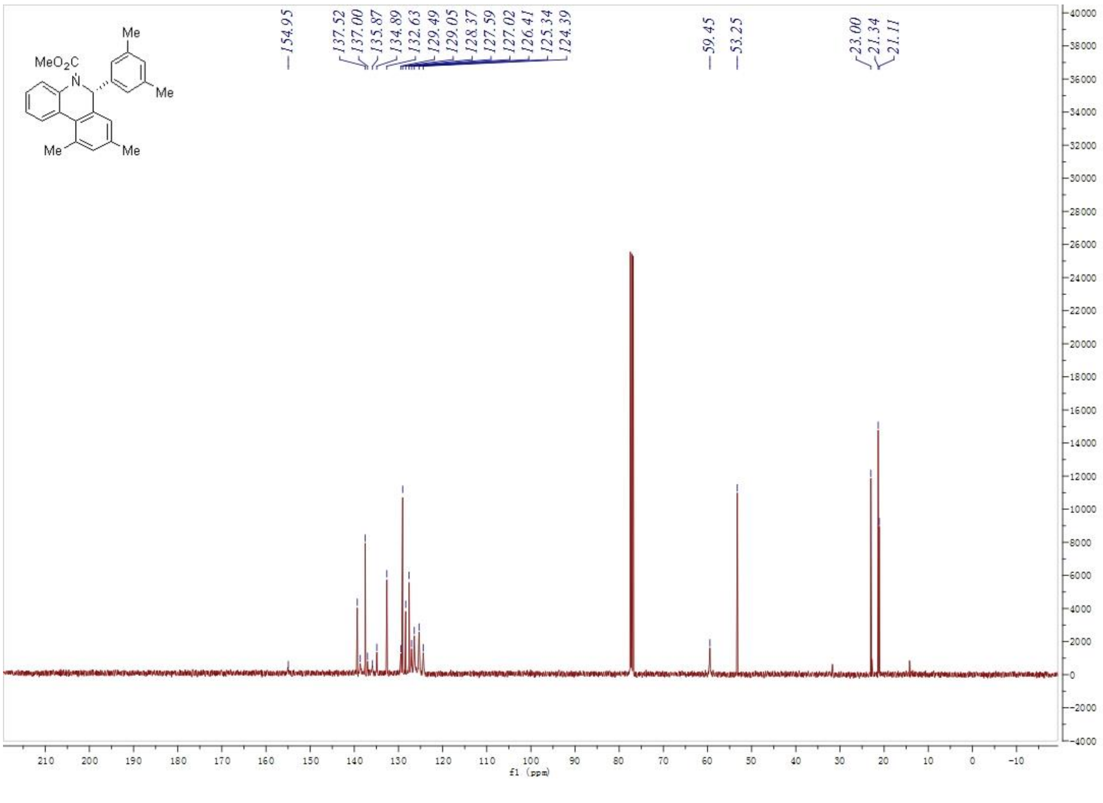

MeO2C
N
Me
Me
Me
Me
154.95
137.52
137.00
135.87
134.89
132.63
129.49
129.05
128.37
127.59
127.02
126.41
125.34
124.39
59.45
53.25
23.00
21.34
21.11
f1 (ppm)

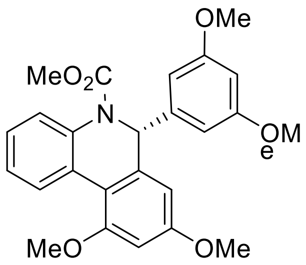
OMe
MeO2C
N
OMe
MeO
OMe

2t

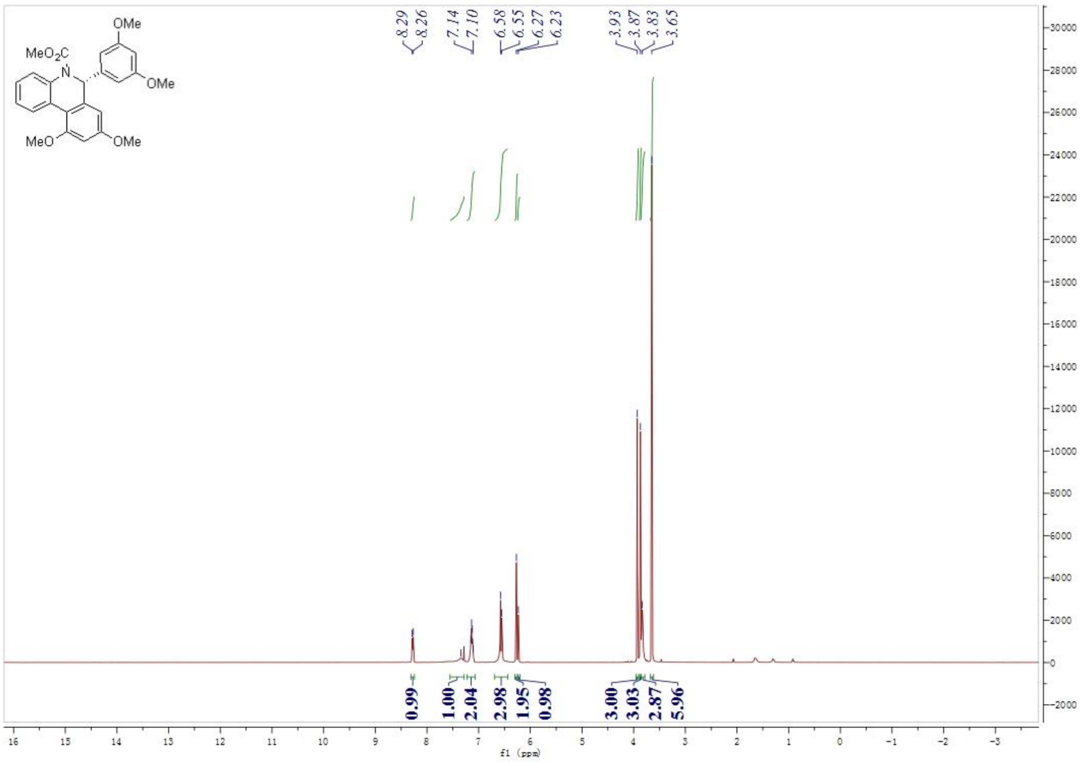
8.29
8.26
7.14
7.10
6.58
6.55
6.27
6.23
3.93
3.87
3.83
3.65
0.99
1.00
2.04
2.98
1.95
0.98
3.00
3.03
2.87
5.96
f1 (ppm)

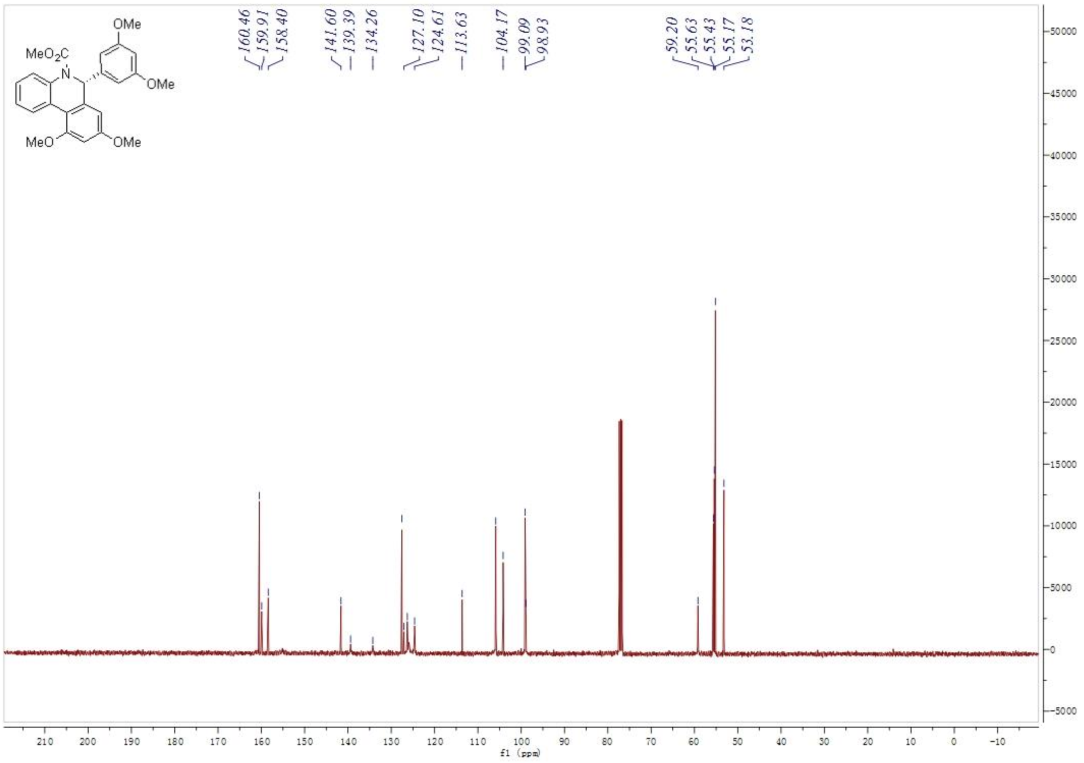
160.46
159.91
158.40
141.60
139.39
134.26
127.10
124.61
113.63
104.17
99.09
98.93
59.20
55.63
55.43
55.17
53.18
MeO2C
N
OMe
OMe
MeO
OMe
f1 (ppm)

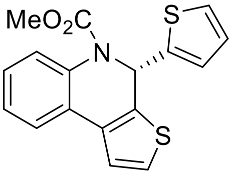

2u

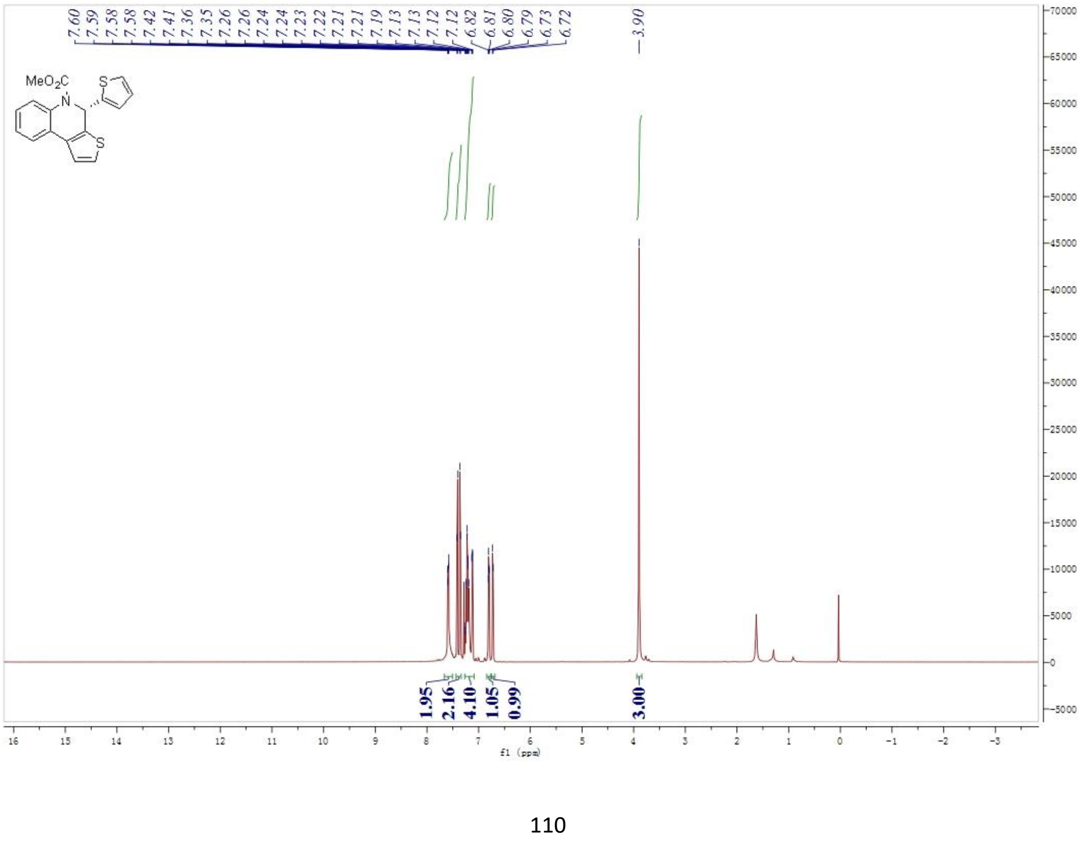
7.60
7.59
7.58
7.58
7.42
7.41
7.36
7.35
7.26
7.26
7.24
7.24
7.23
7.22
7.21
7.21
7.19
7.13
7.13
7.12
7.12
6.82
6.81
6.80
6.79
6.73
6.72
3.90
MeO2C
N
S
S
1.95
2.16
4.10
1.05
0.99
3.00
f1 (ppm)

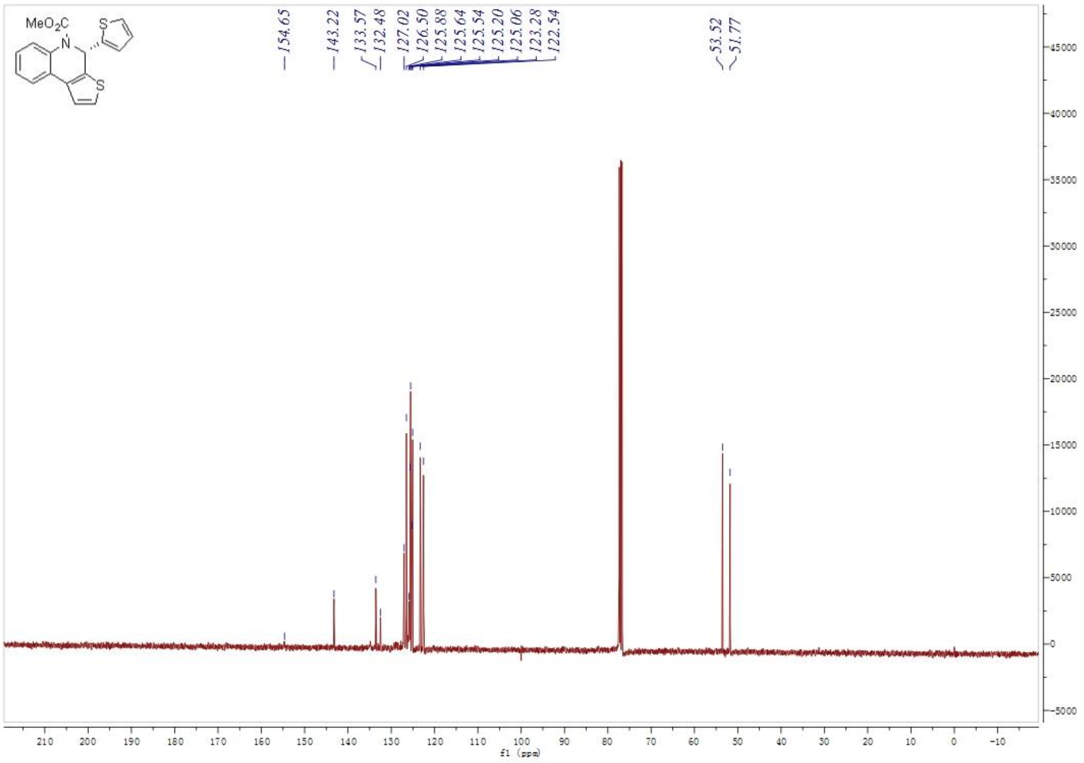
MeO2C
N
S
S
154.65
143.22
133.57
132.48
127.02
126.50
125.88
125.64
125.54
125.20
125.06
123.28
122.54
53.52
51.77
45000
40000
35000
30000
25000
20000
15000
10000
5000
0
-5000
210
200
190
180
170
160
150
140
130
120
110
100
90
80
70
60
50
40
30
20
10
0
-10
f1 (ppm)

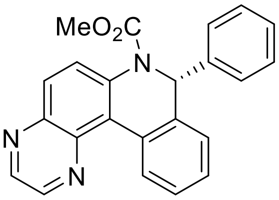
MeO2C
N
N
N

2v

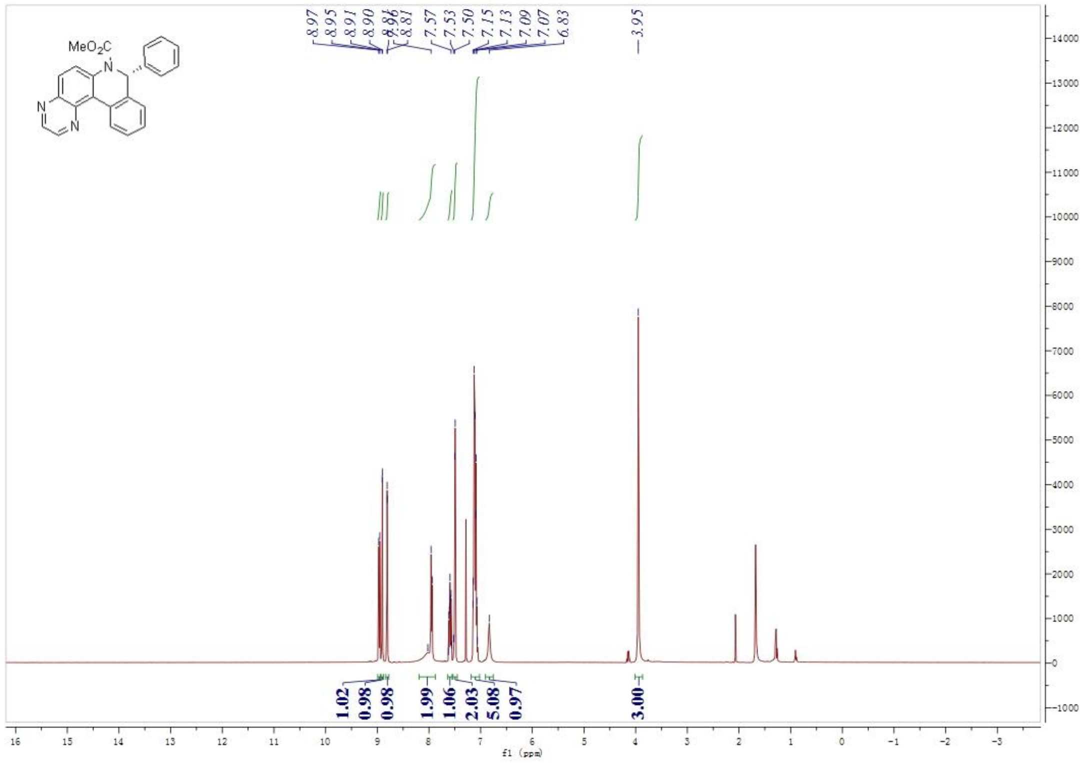

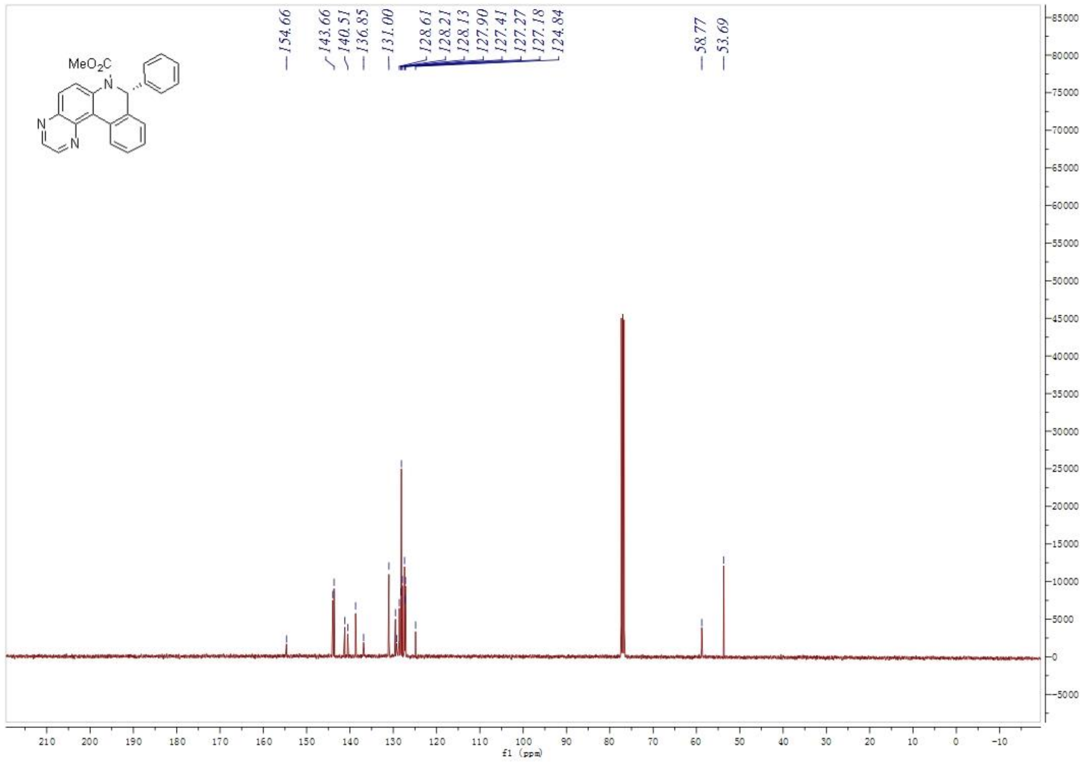

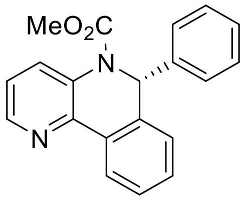

2w

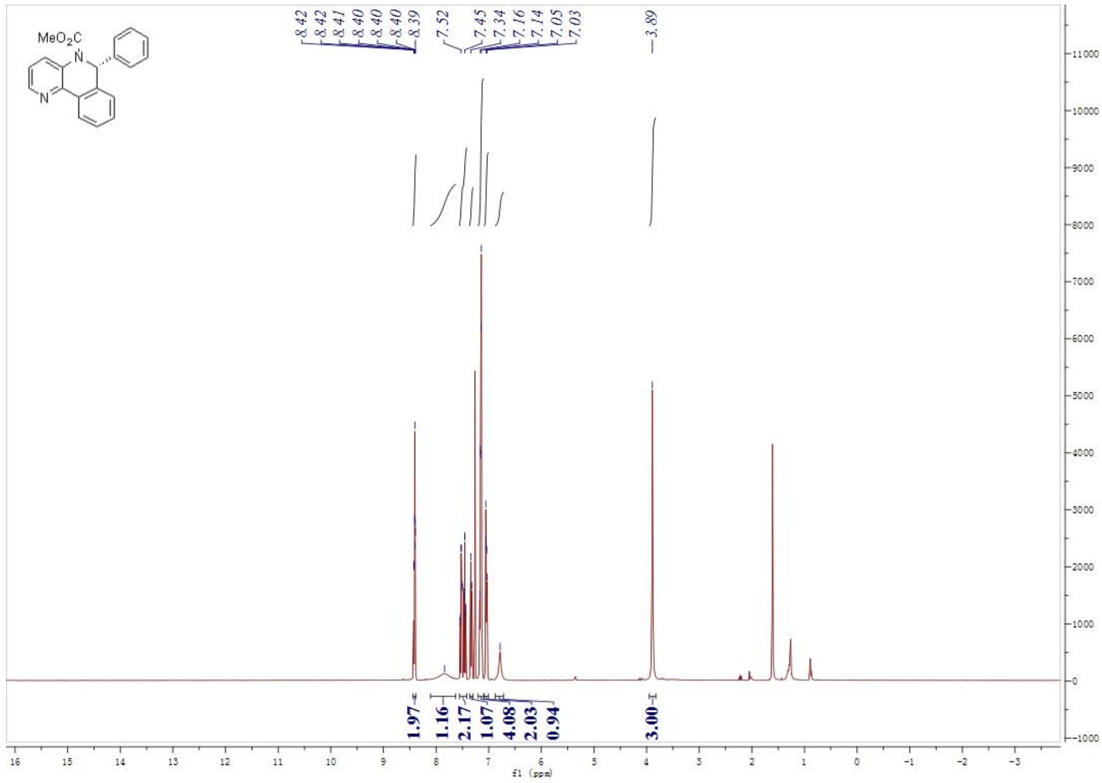

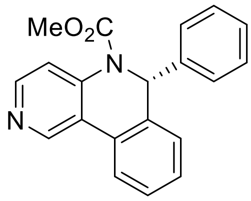

2X

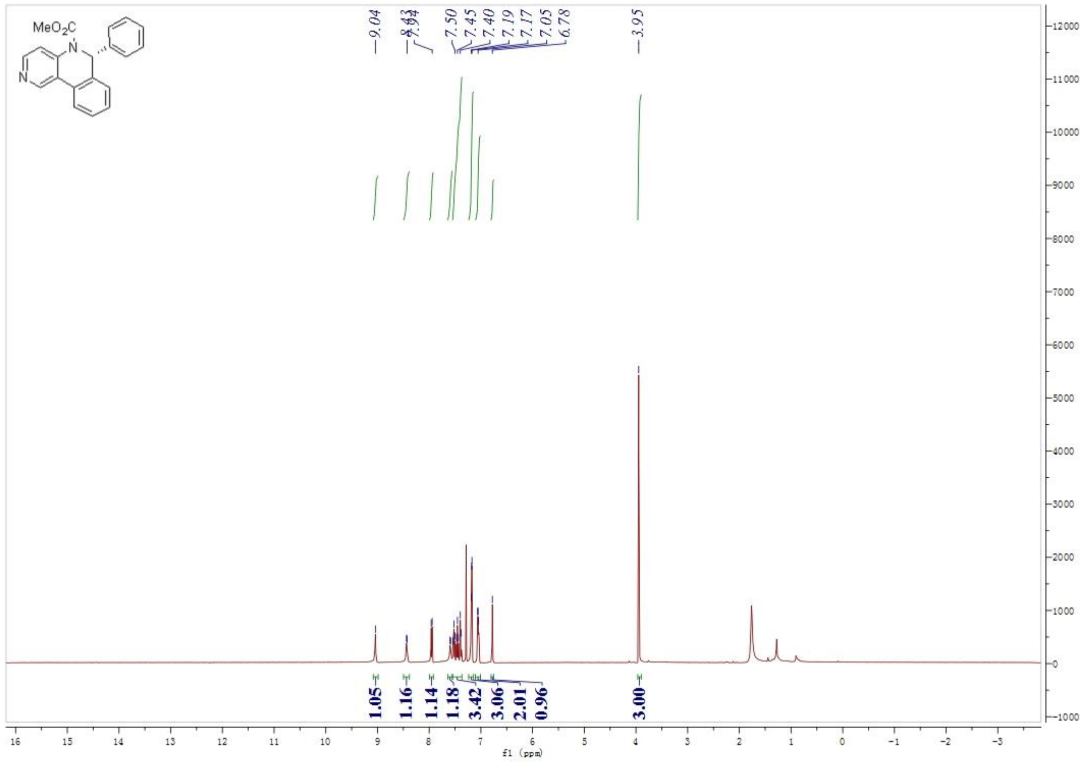

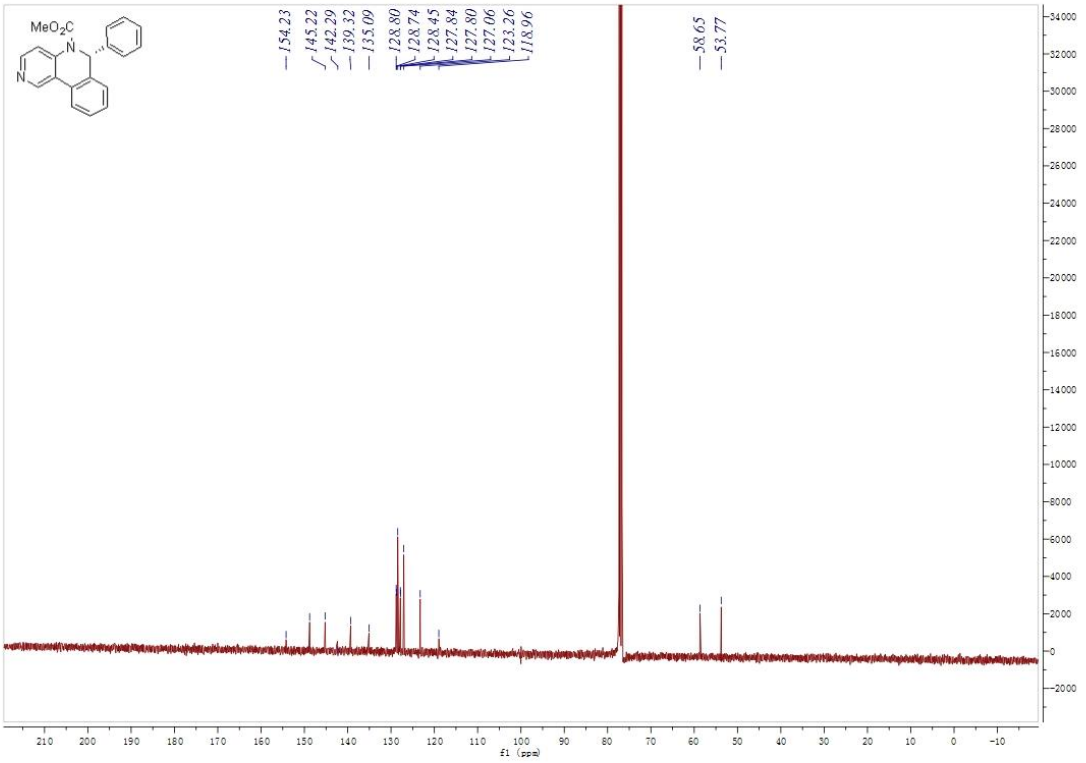
MeO2C
N
154.23
145.22
142.29
139.32
135.09
128.80
128.74
128.45
127.84
127.80
127.06
123.26
118.96
58.65
53.77
f1 (ppm)

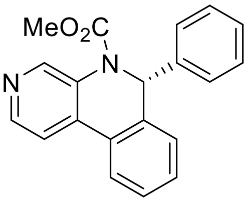
MeO2C
N
N

2y

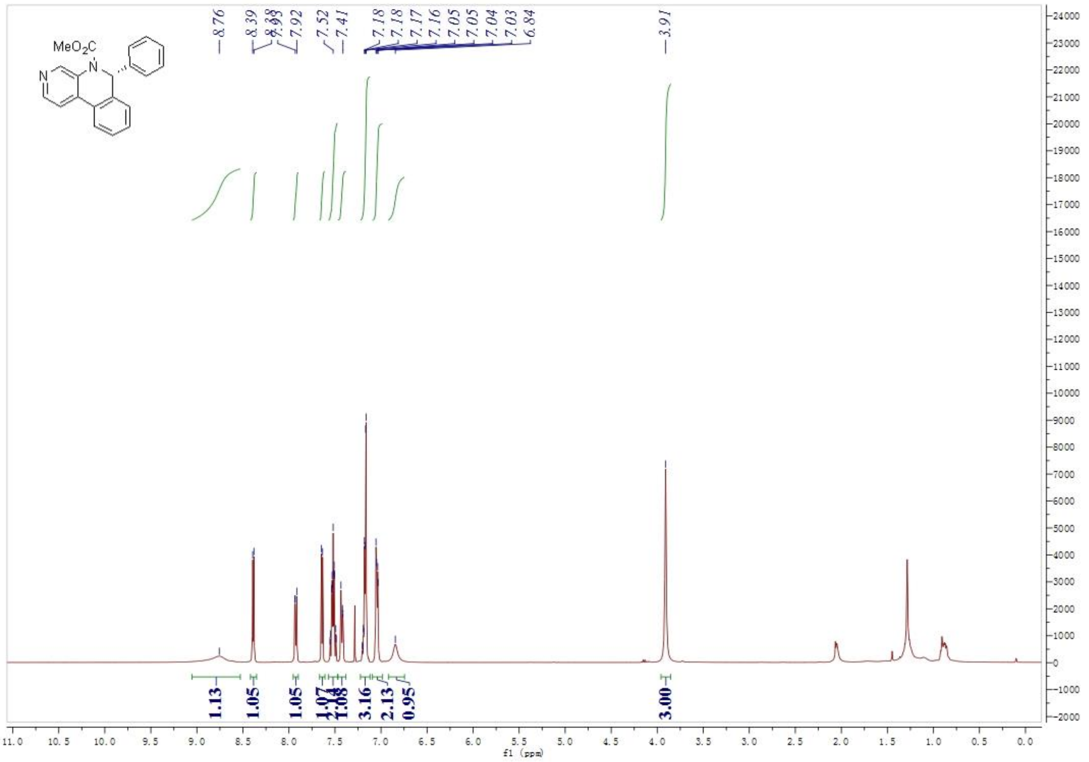
MeO2C
N
8.76
8.39
8.38
7.93
7.92
7.52
7.41
7.18
7.18
7.17
7.16
7.05
7.05
7.04
7.03
6.84
3.91
1.13
1.05
1.05
3.16
2.13
0.95
3.00
f1 (ppm)

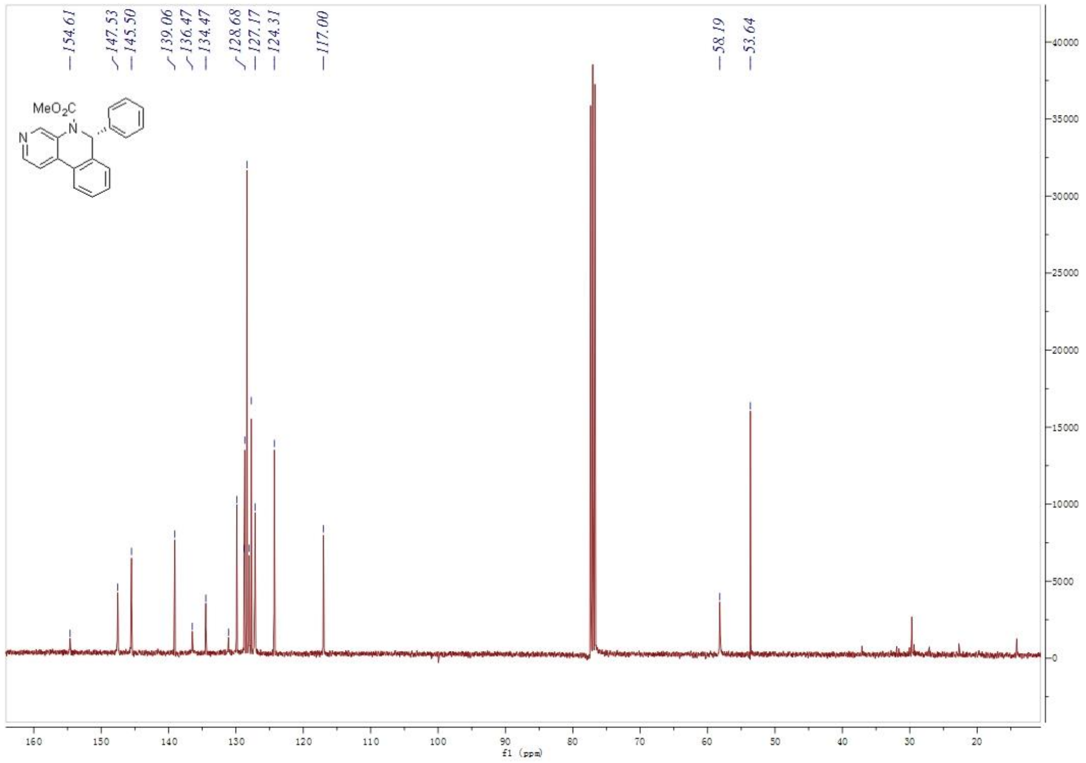

154.61
147.53
145.50
139.06
136.47
134.47
128.68
127.17
124.31
117.00
58.19
53.64
MeO2C
N
f1 (ppm)

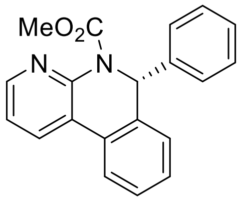

MeO2C
N
N

2z

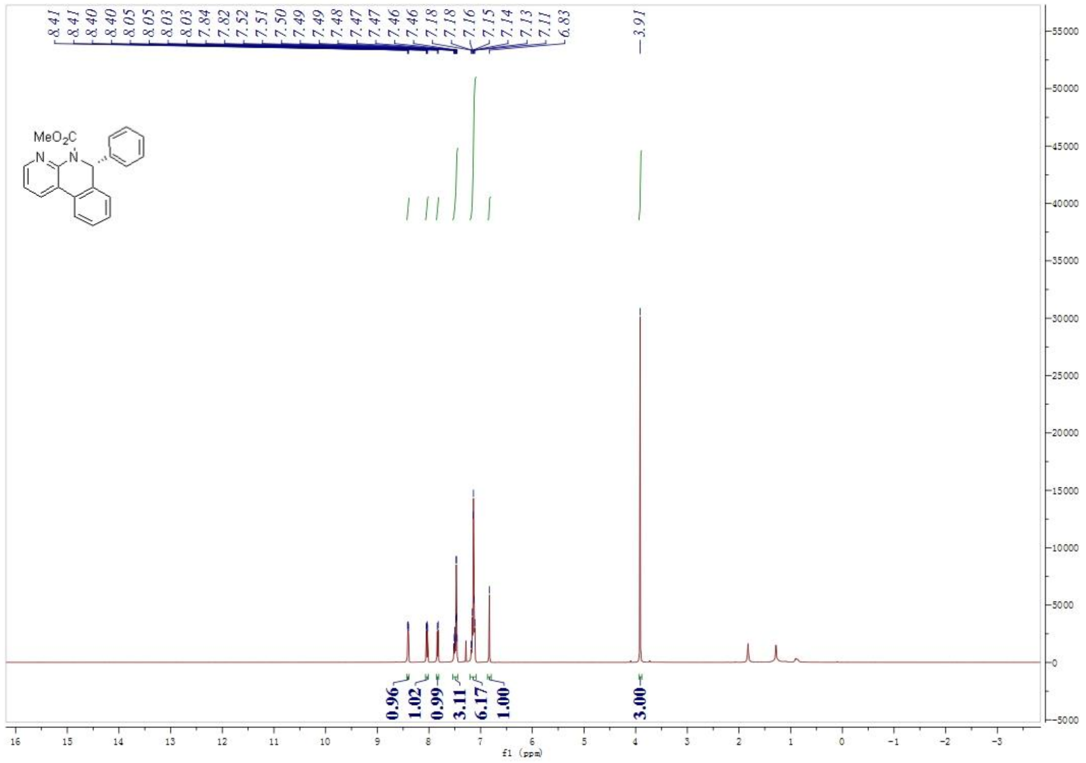

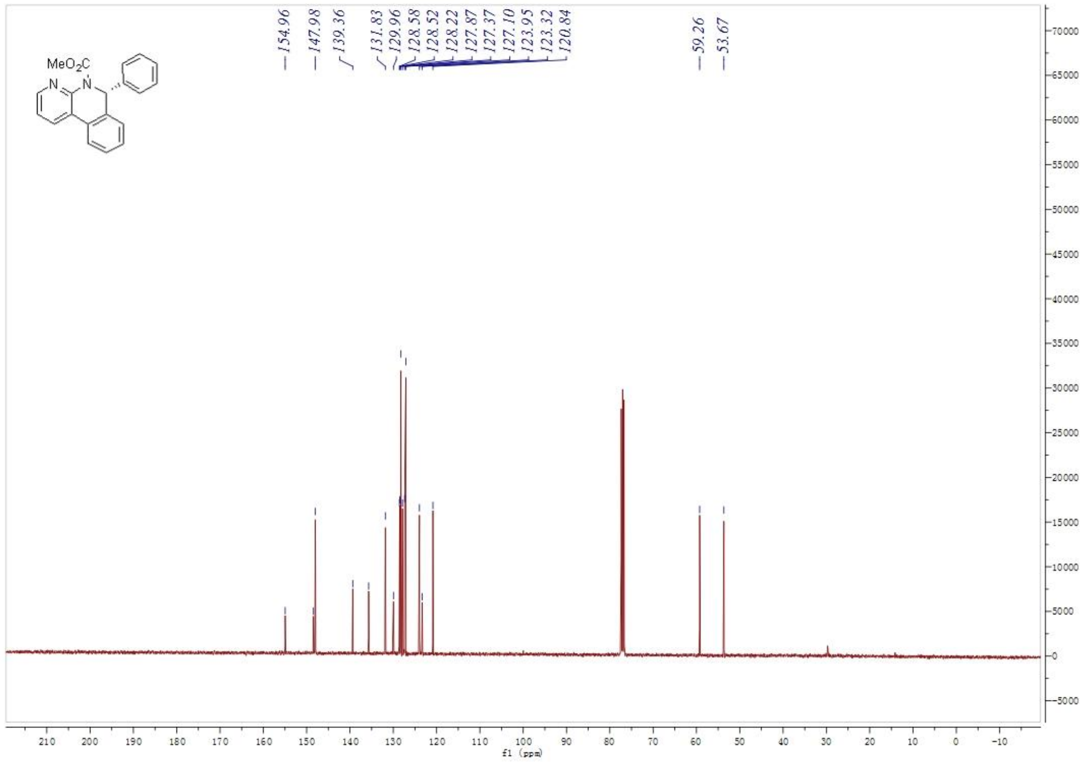

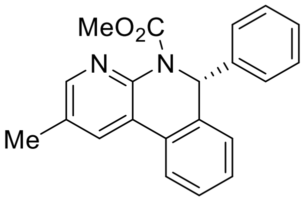

2aa

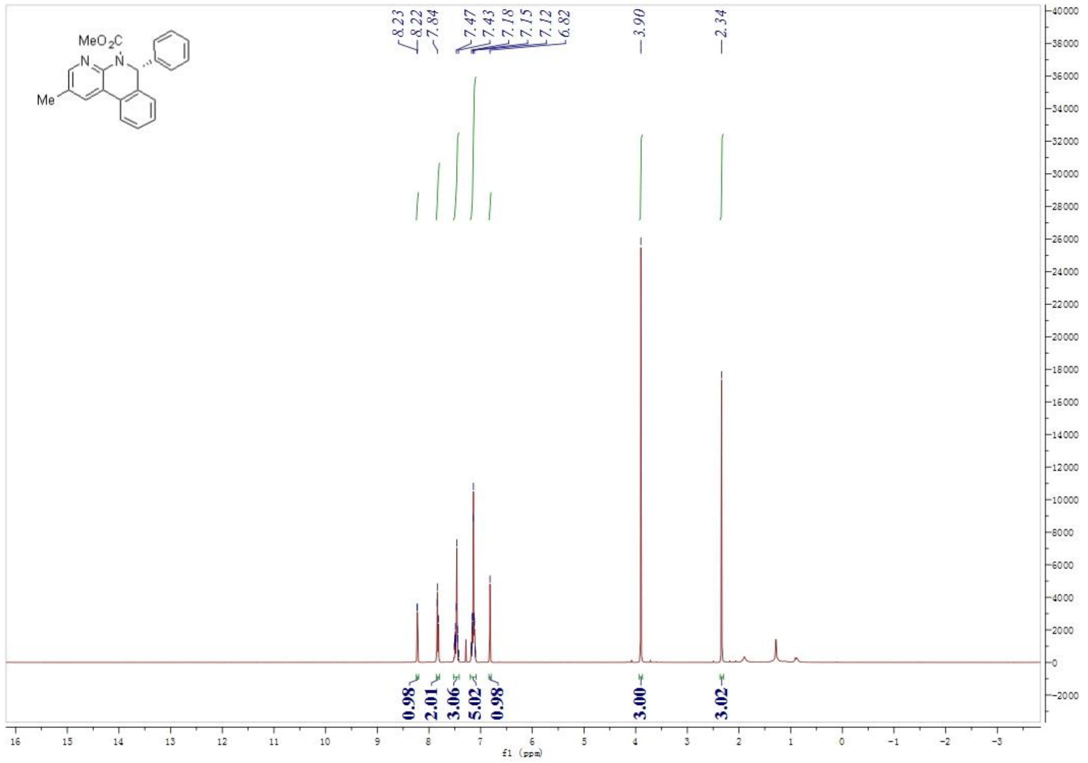
MeO2C
N
N
Me
8.23
8.22
7.84
7.47
7.43
7.18
7.15
7.12
6.82
3.90
2.34
0.98
2.01
3.06
5.02
0.98
3.00
3.02
f1 (ppm)

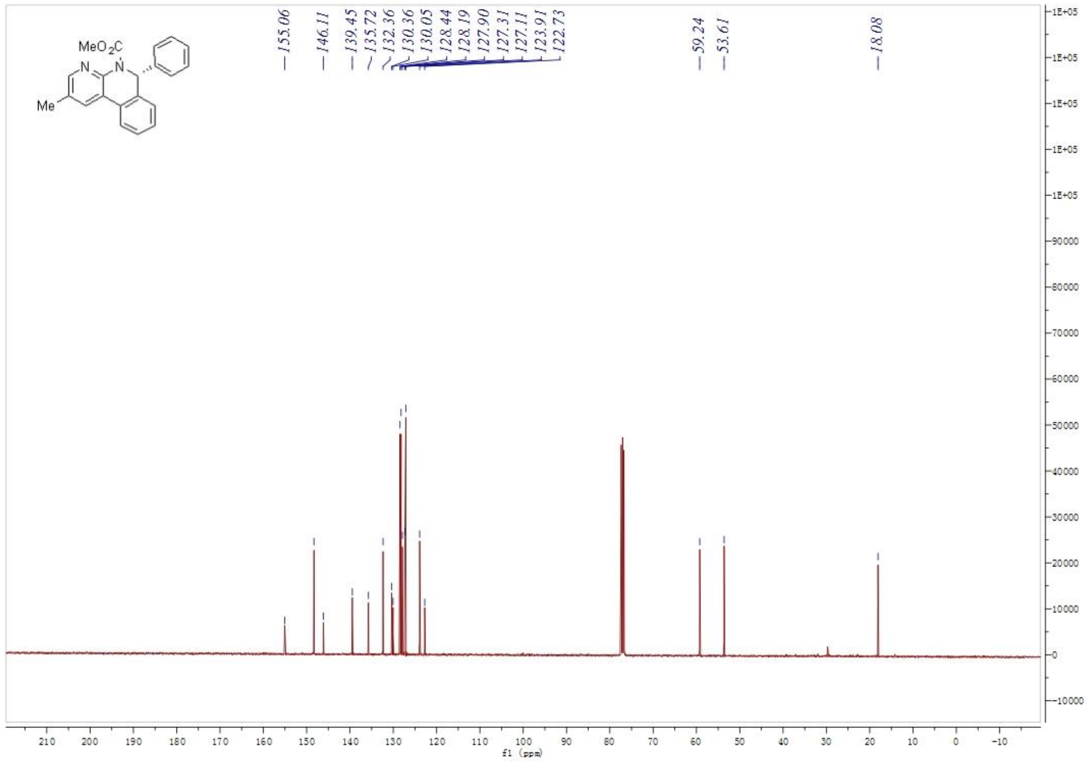
MeO2C
N
N
Me
155.06
146.11
139.45
135.72
132.36
130.36
130.05
128.44
128.19
127.90
127.31
127.11
123.91
122.73
59.24
53.61
18.08
f1 (ppm)

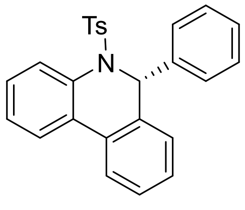
Ts
N

2ab

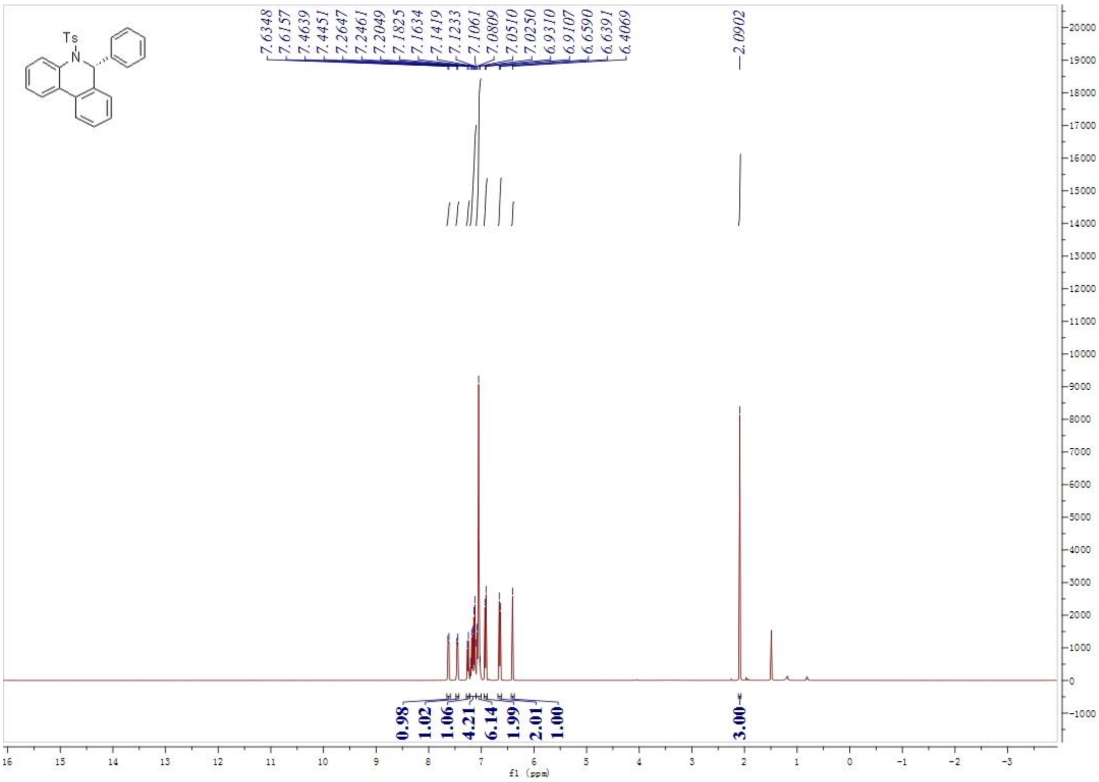
Ts
N
7.6348
7.6157
7.4639
7.4451
7.2647
7.2461
7.2049
7.1825
7.1634
7.1419
7.1233
7.1061
7.0809
7.0510
7.0250
6.9310
6.9107
6.6590
6.6391
6.4069
2.0902
0.98
1.02
1.06
4.21
6.14
1.99
2.01
1.00
3.00
f1 (ppm)

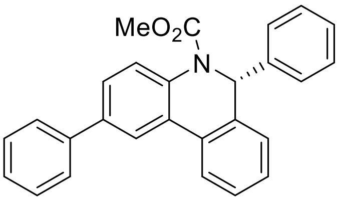
MeO2C
N

4a

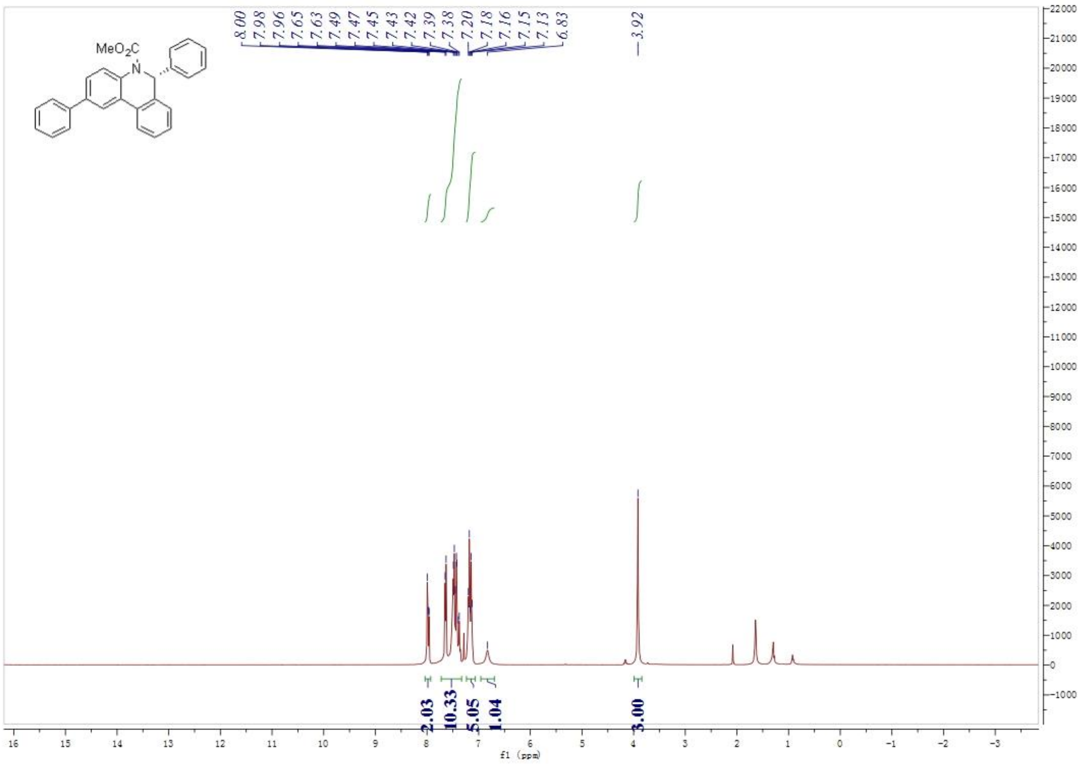
MeO2C
N
8.00
7.98
7.96
7.65
7.63
7.49
7.47
7.45
7.43
7.42
7.39
7.38
7.20
7.18
7.16
7.15
7.13
6.83
3.92
2.03
10.33
5.05
1.04
3.00
f1 (ppm)

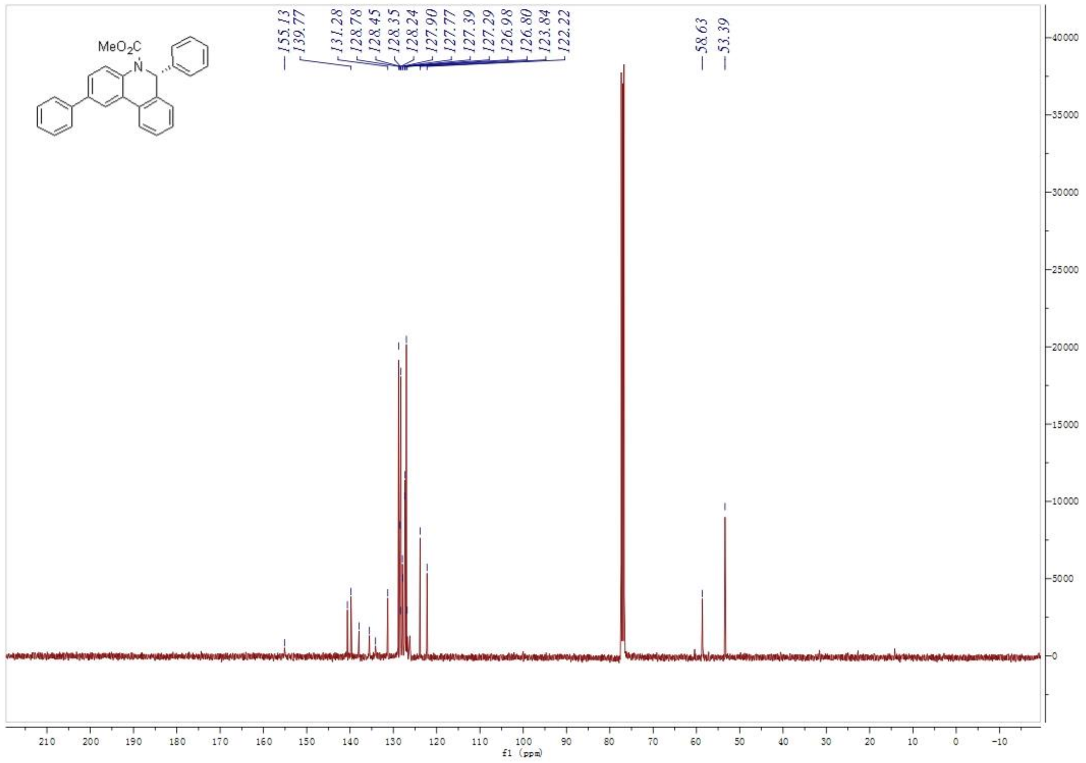
155.13
139.77
131.28
128.78
128.45
128.35
128.24
127.90
127.77
127.39
127.29
126.98
126.80
123.84
122.22
58.63
53.39
MeO2C
N
f1 (ppm)

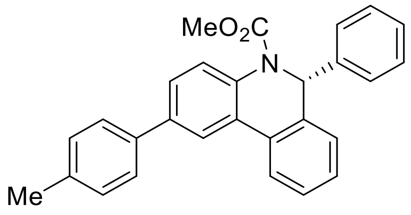
MeO2C
N
Me

4b

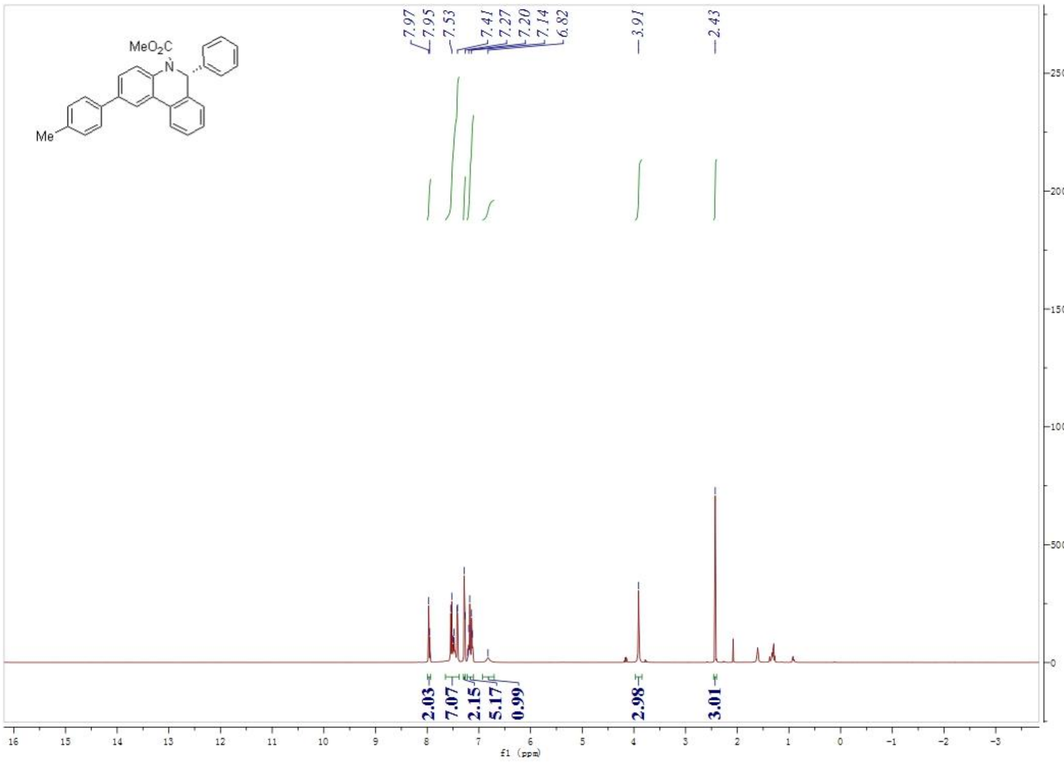

MeO2C
N
Me
7.97
7.95
7.53
7.41
7.27
7.20
7.14
6.82
3.91
2.43
2.03
7.07
2.15
5.17
0.99
2.98
3.01
f1 (ppm)

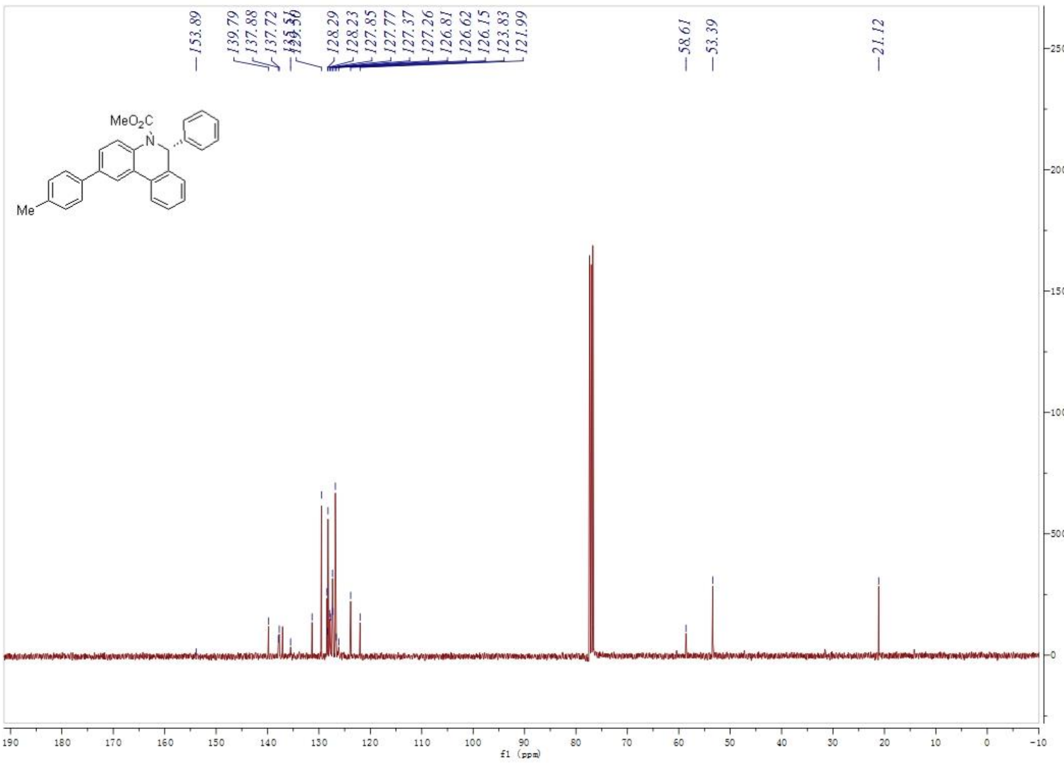

153.89
139.79
137.88
137.72
135.51
135.50
128.29
128.23
127.85
127.77
127.37
127.26
126.81
126.62
126.15
123.83
121.99
58.61
53.39
21.12
MeO2C
N
Me
f1 (ppm)

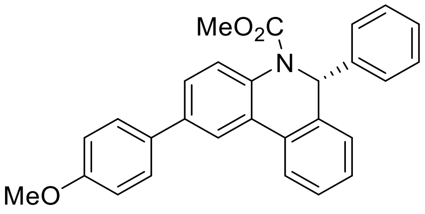

MeO2C
N
MeO

4c

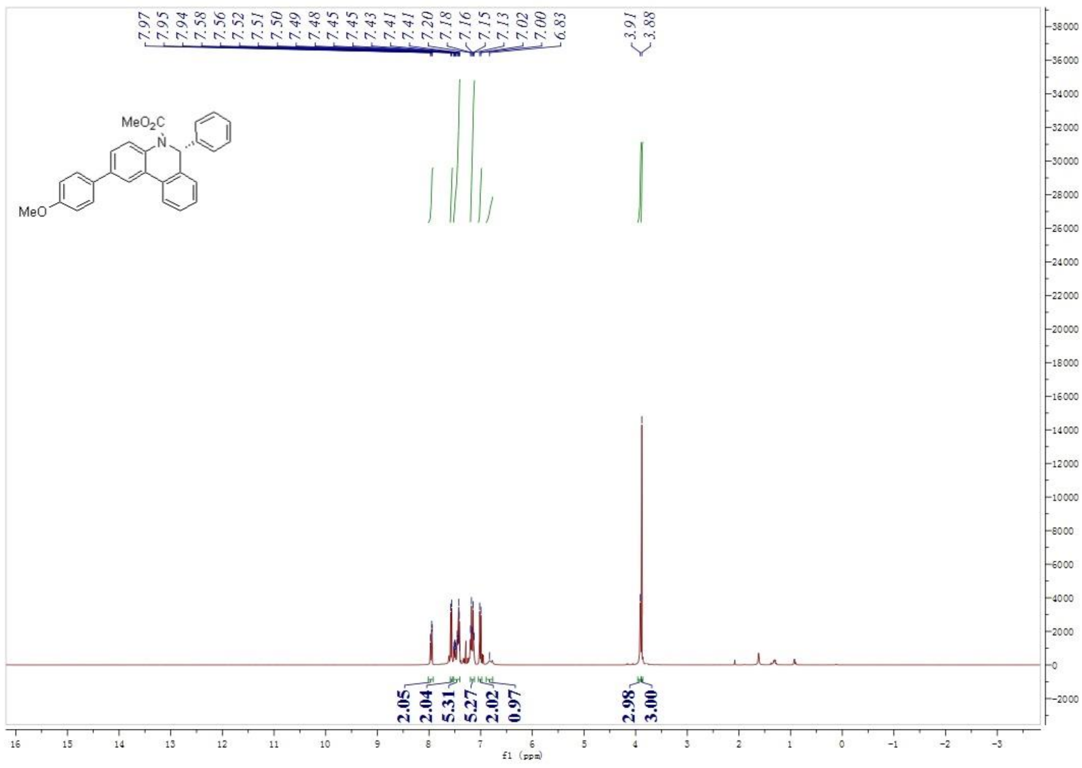

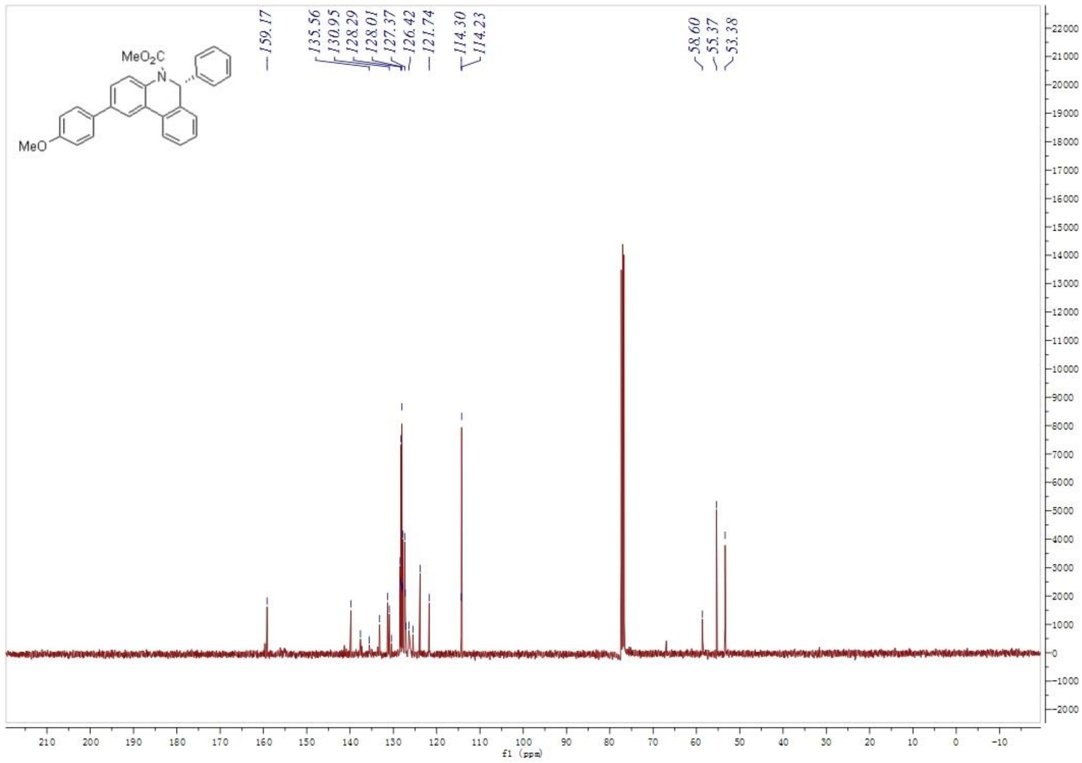

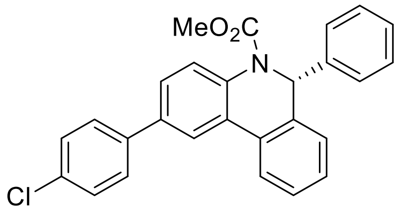
MeO2C
N
Cl

4d

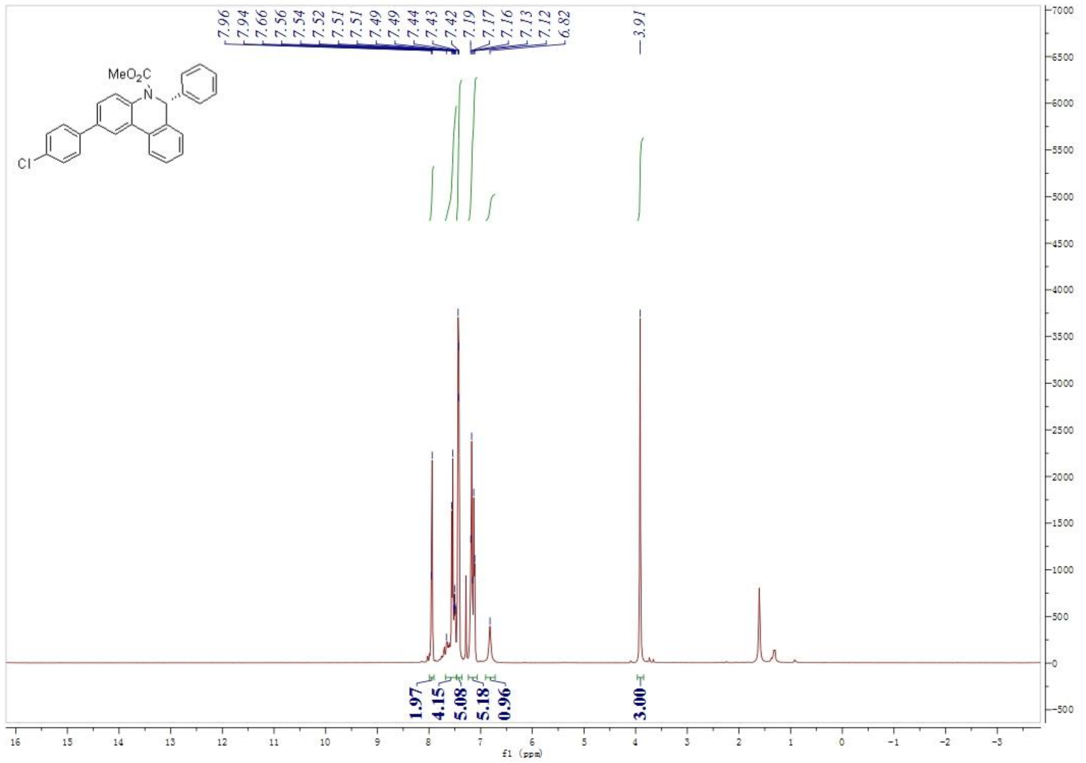
7.96
7.94
7.66
7.56
7.54
7.52
7.51
7.51
7.49
7.49
7.44
7.43
7.42
7.19
7.17
7.16
7.13
7.12
6.82
3.91
1.97
4.15
5.08
5.18
0.96
3.00
f1 (ppm)

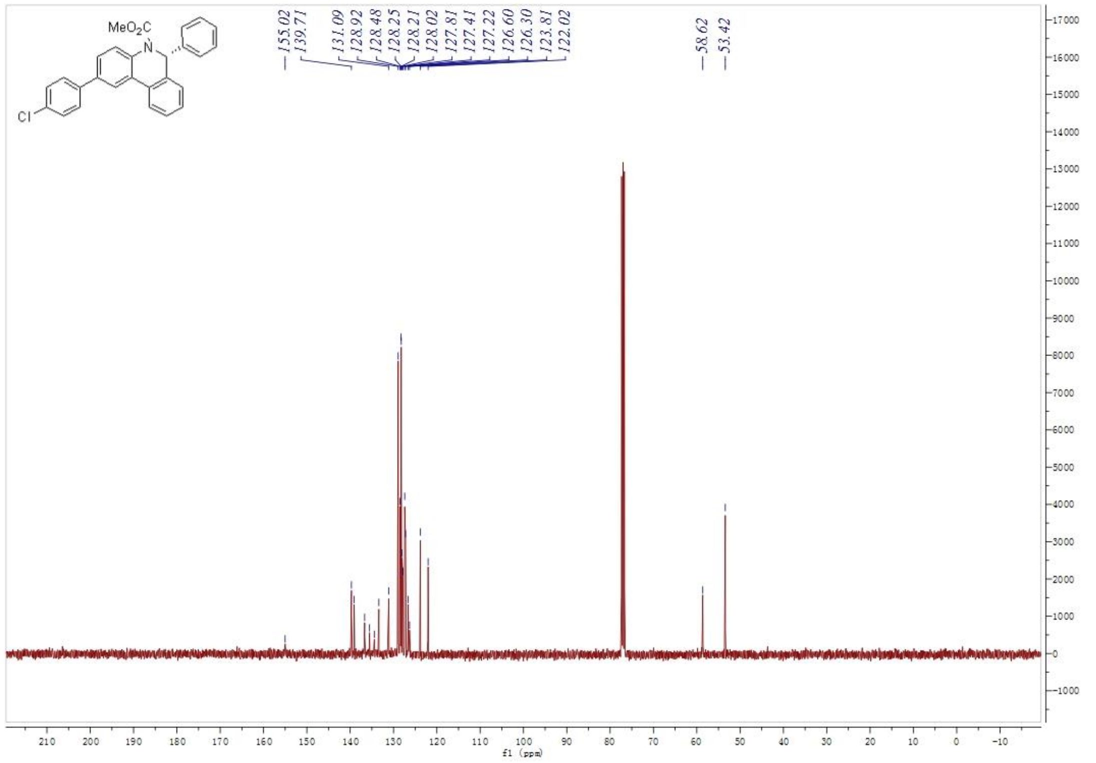

MeO2C
N
Cl
155.02
139.71
131.09
128.92
128.48
128.25
128.21
128.02
127.81
127.41
127.22
126.60
126.30
123.81
122.02
58.62
53.42
f1 (ppm)

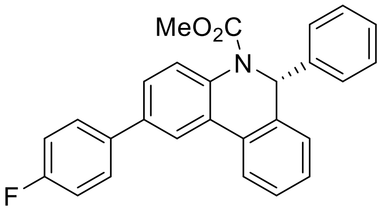

MeO2C
N
F

4e

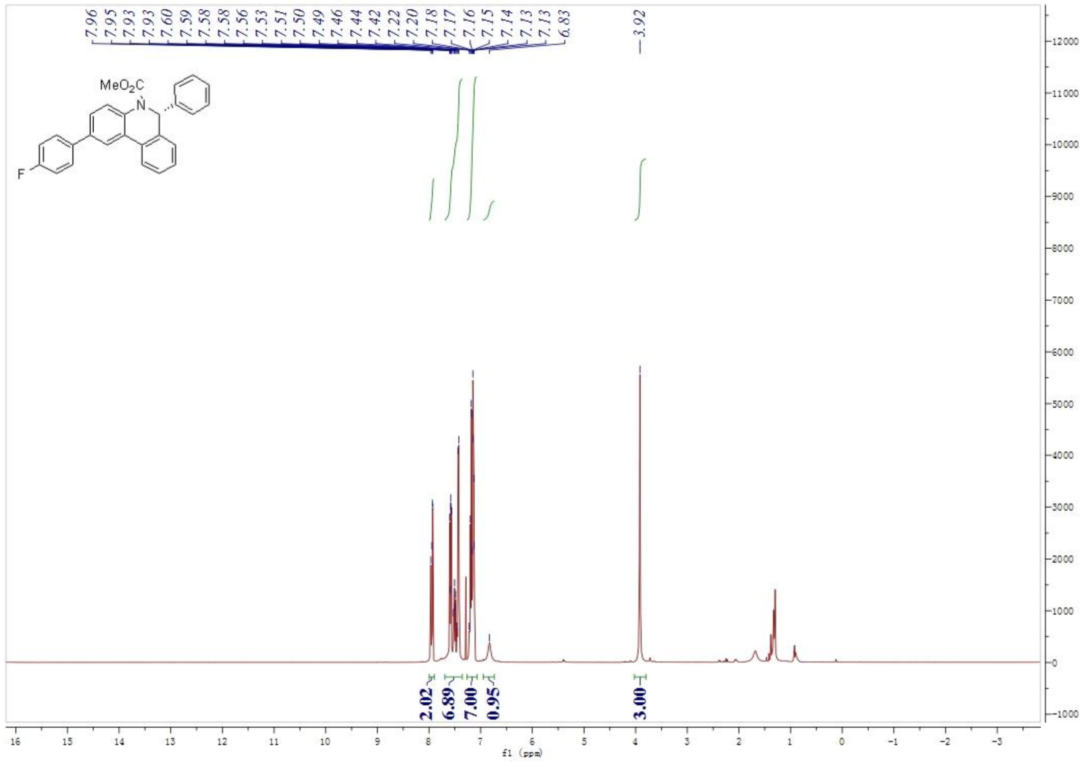
7.96 7.95 7.93 7.93 7.60 7.59 7.58 7.58 7.56 7.53 7.51 7.50 7.49 7.46 7.44 7.42 7.22 7.20 7.18 7.17 7.16 7.15 7.14 7.13 7.13 6.83
3.92
MeO2C
N
F
2.02
6.89
7.00
0.95
3.00
f1 (ppm)

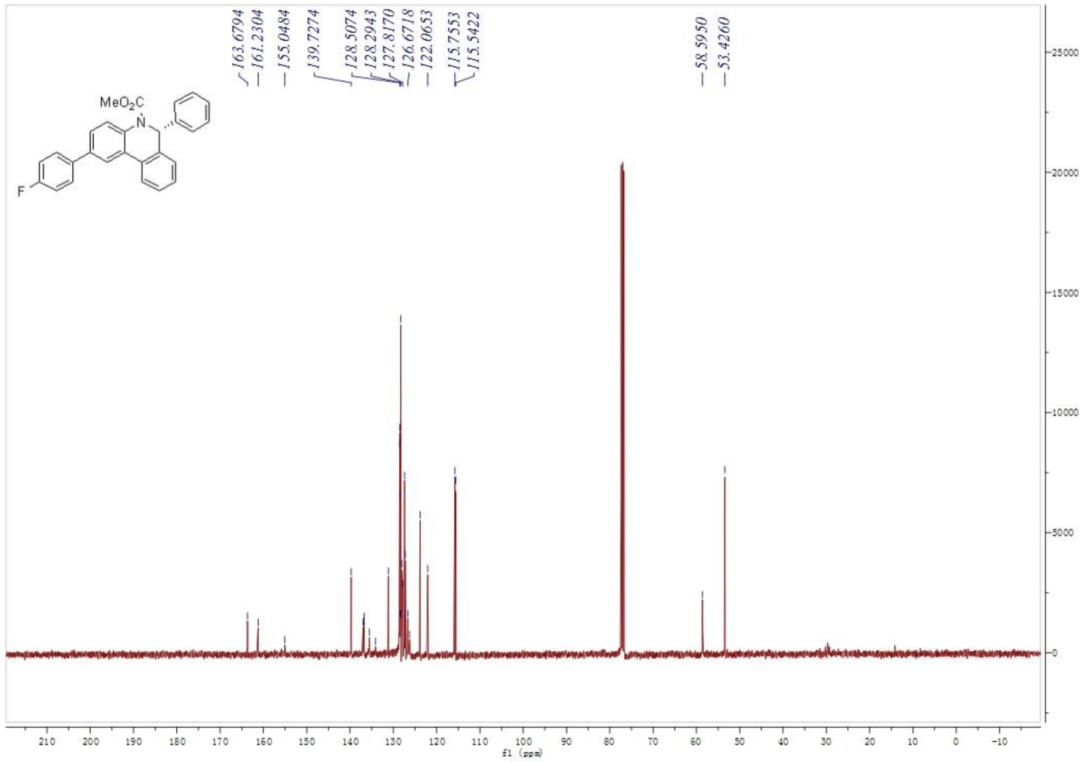
163.6794
161.2304
155.0484
139.7274
128.5074
128.2943
127.8170
126.6718
122.0653
115.7553
115.5422
58.5950
53.4260
MeO2C
N
F
f1 (ppm)

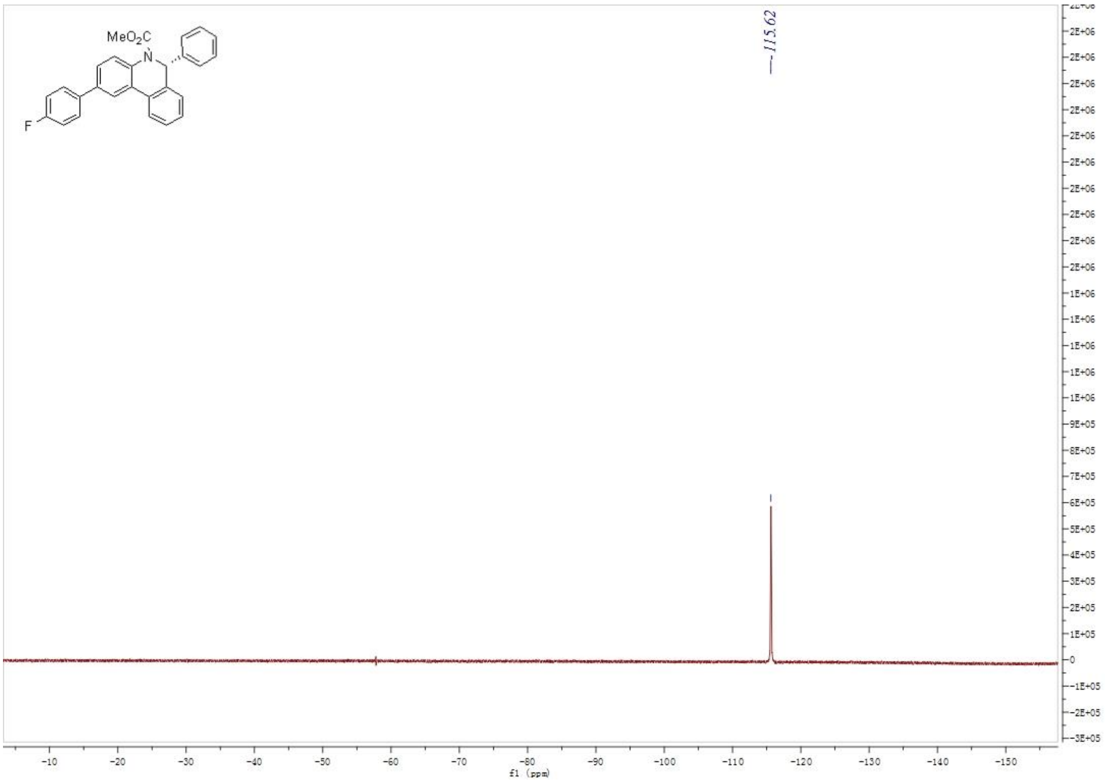

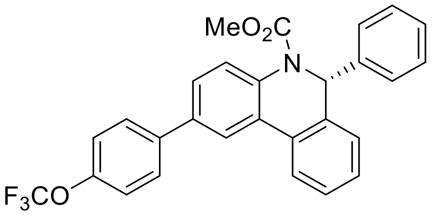

4f

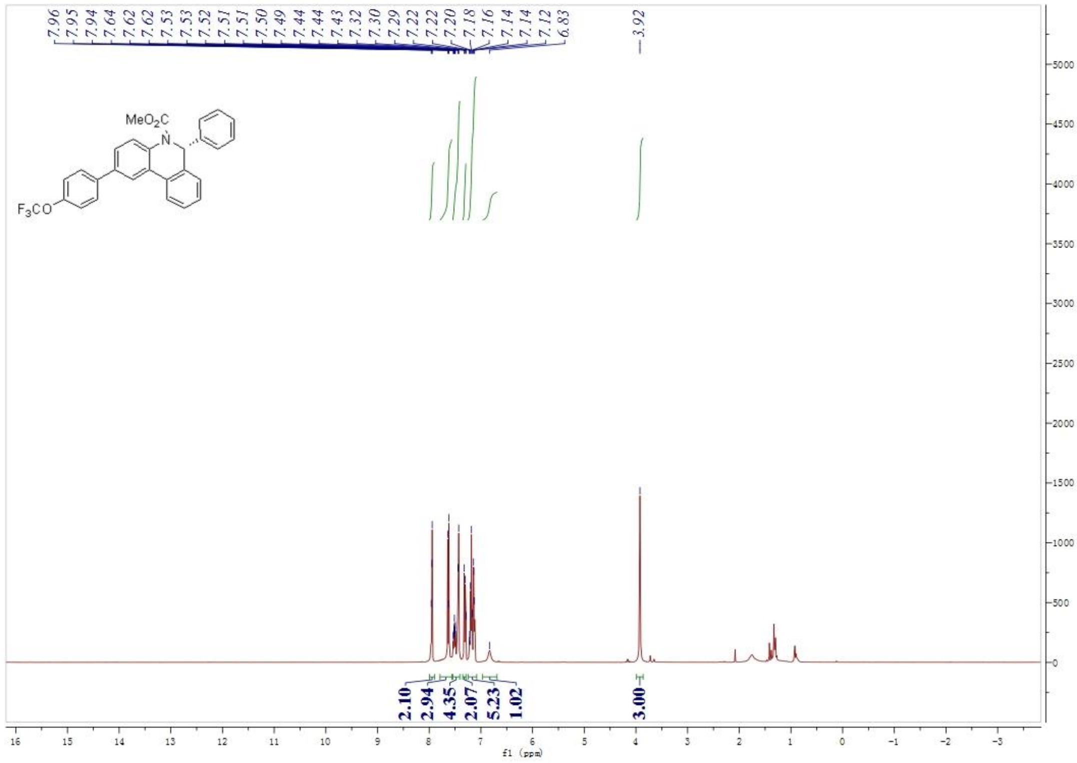
7.96 7.95 7.94 7.64 7.62 7.62 7.53 7.53 7.52 7.51 7.51 7.50 7.49 7.44 7.44 7.43 7.32 7.30 7.29 7.22 7.22 7.20 7.18 7.16 7.14 7.14 7.12 6.83
3.92
MeO2C
N
F3CO
2.10 2.94 4.35 2.07 5.23 1.02
3.00
f1 (ppm)

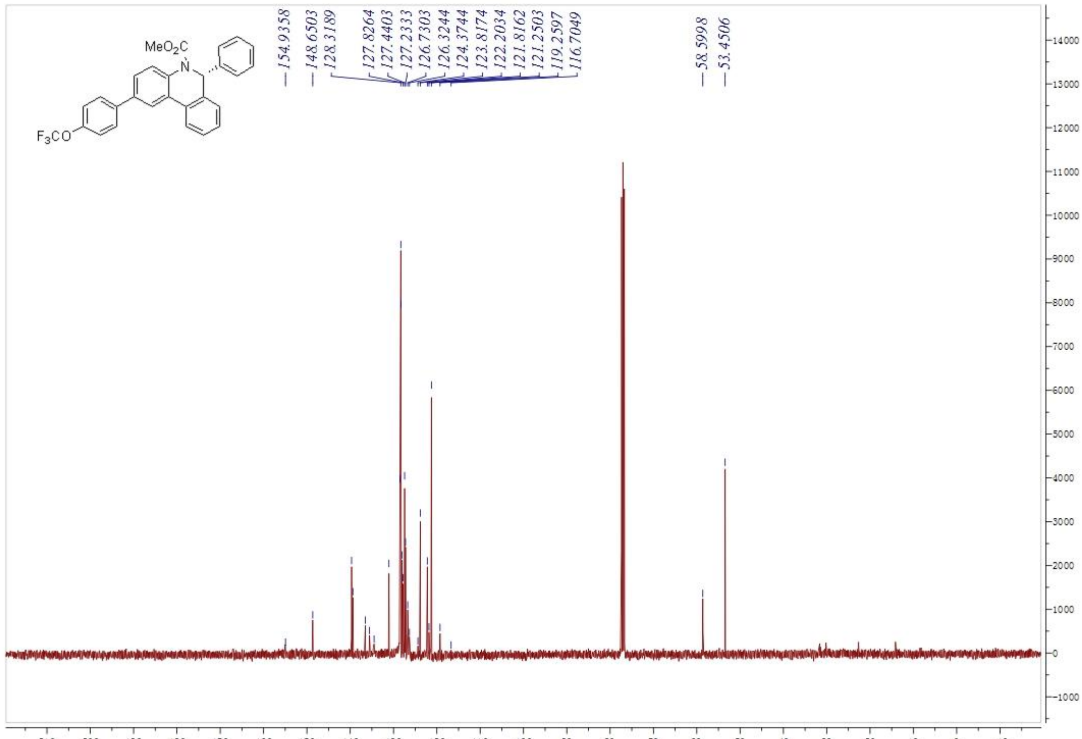
154.9358 148.6503 128.3189 127.8264 127.4403 127.2333 126.7303 126.3244 124.3744 123.8174 122.2034 121.8162 121.2503 119.2597 116.7049
58.5998 53.4506
MeO2C
N
F3CO
f1 (ppm)

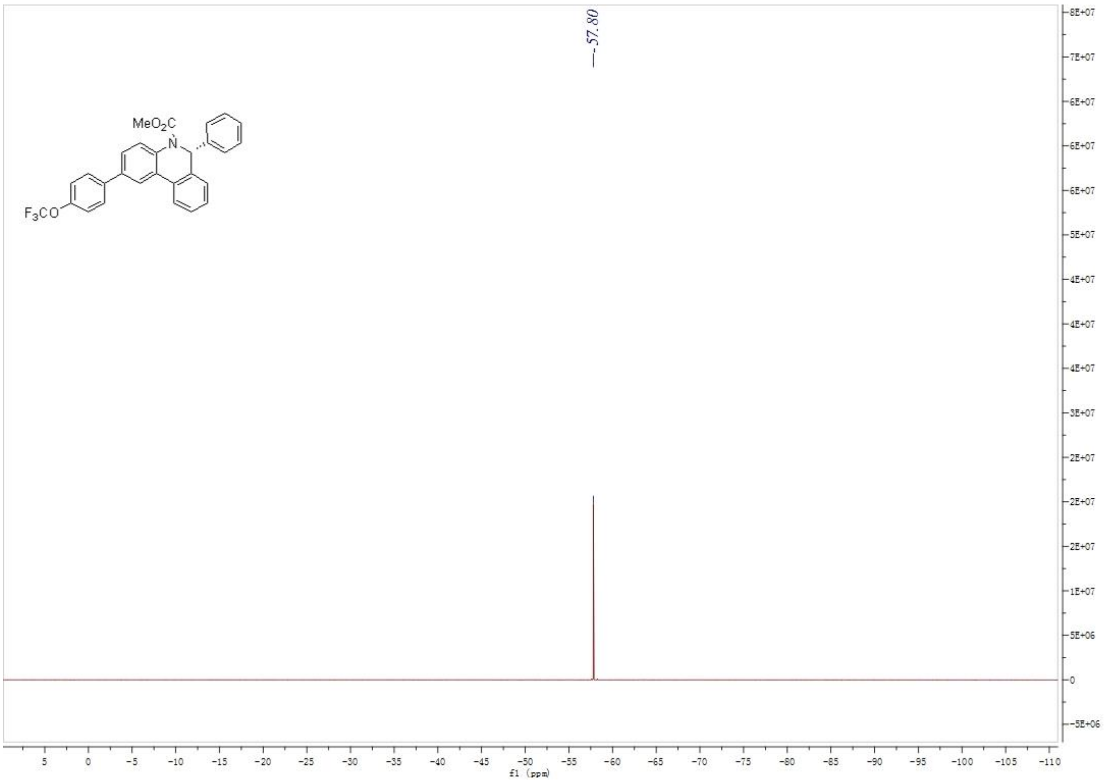

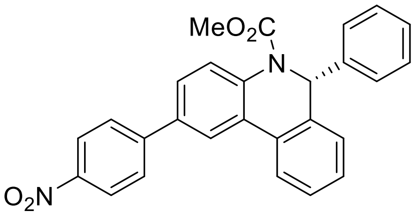

4g

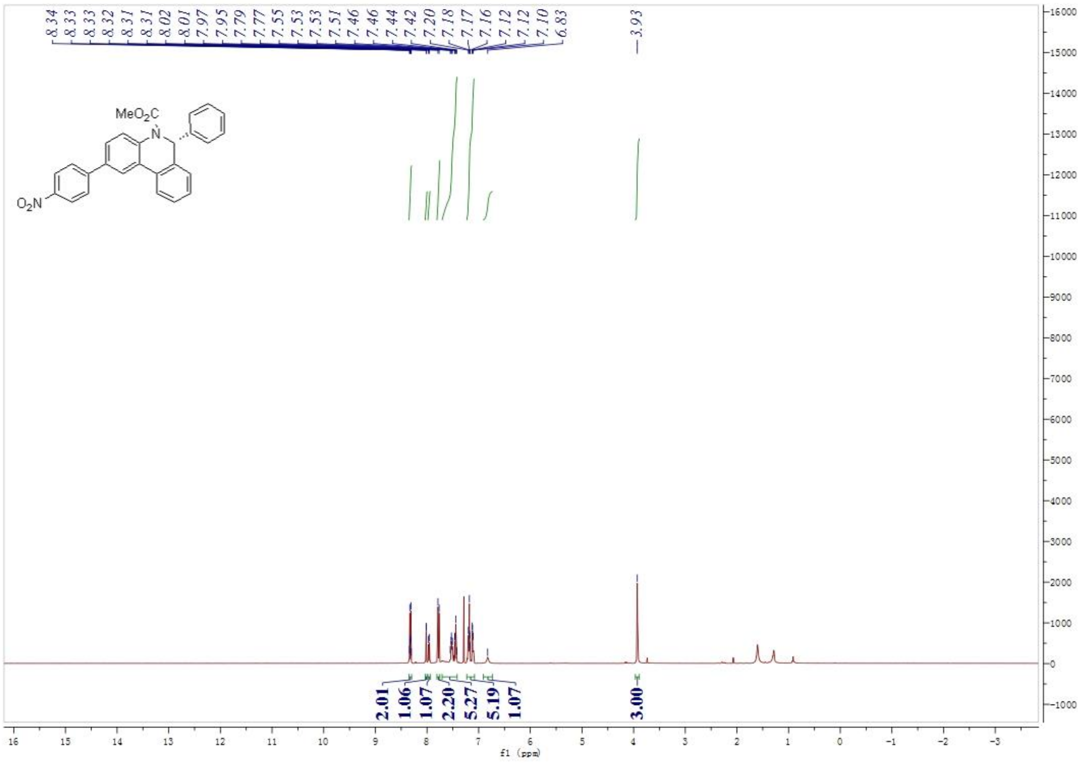
8.34
8.33
8.33
8.32
8.31
8.31
8.02
8.01
7.97
7.95
7.79
7.77
7.55
7.53
7.53
7.51
7.46
7.46
7.44
7.42
7.20
7.18
7.17
7.16
7.12
7.12
7.10
6.83
3.93
MeO2C
N
O2N
2.01
1.06
1.07
2.20
5.27
5.19
1.07
3.00
f1 (ppm)

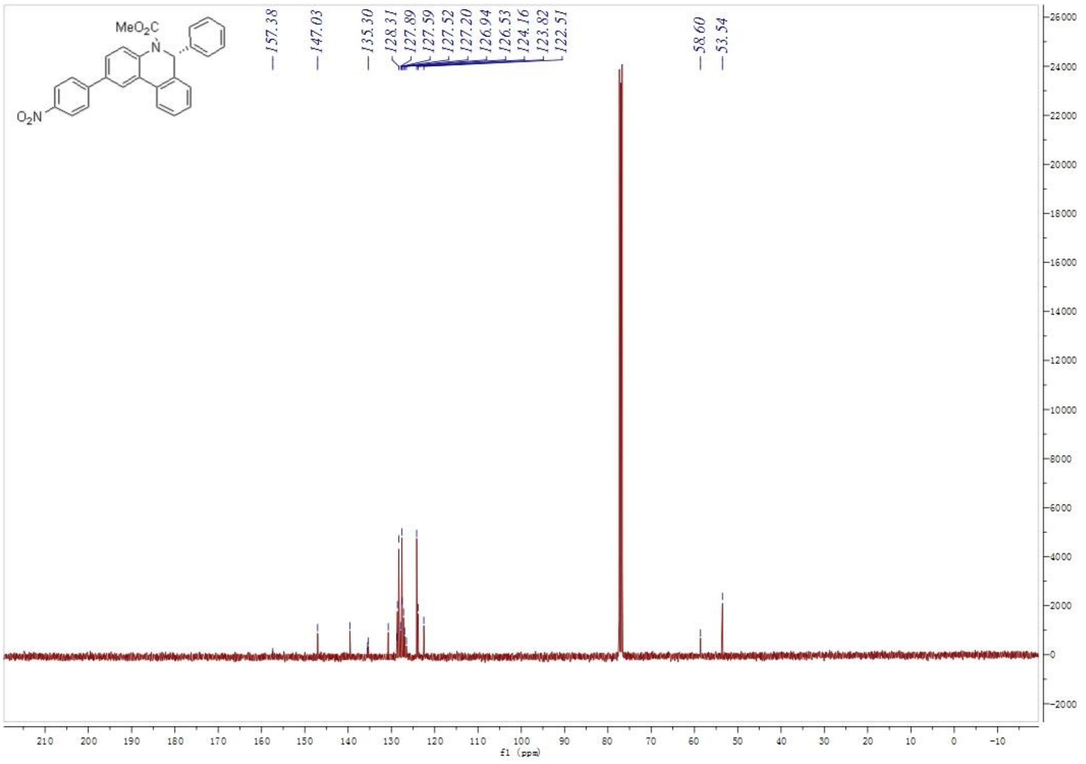
MeO2C
N
O2N
157.38
147.03
135.30
128.31
127.89
127.59
127.52
127.20
126.94
126.53
124.16
123.82
122.51
58.60
53.54
f1 (ppm)

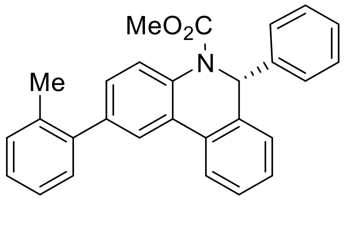
MeO2C
N
Me

4h

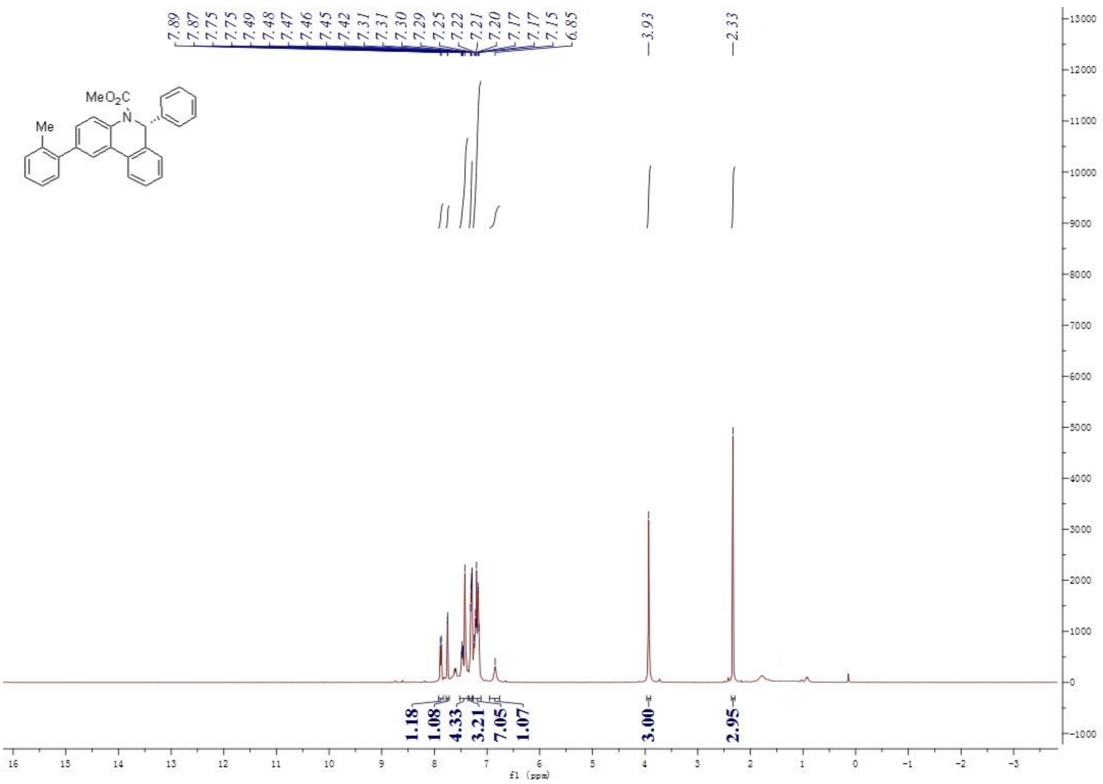
MeO$_2$C
N
Me
7.89 7.87 7.75 7.75 7.49 7.48 7.47 7.46 7.45 7.42 7.31 7.31 7.30 7.29 7.25 7.22 7.21 7.20 7.17 7.17 7.15 6.85
3.93
2.33
1.18 1.08 4.33 3.21 7.05 1.07
3.00
2.95
f1 (ppm)

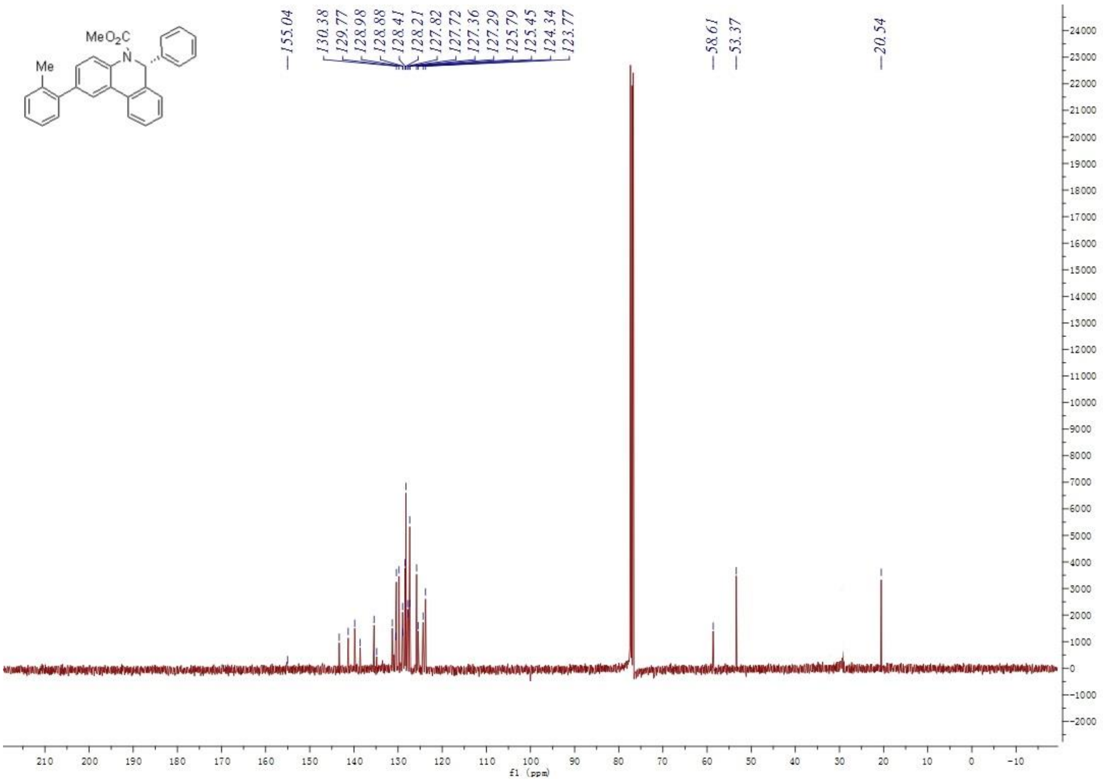
MeO$_2$C
N
Me
155.04
130.38 129.77 128.98 128.88 128.41 128.21 127.82 127.72 127.36 127.29 125.79 125.45 124.34 123.77
58.61
53.37
20.54
f1 (ppm)

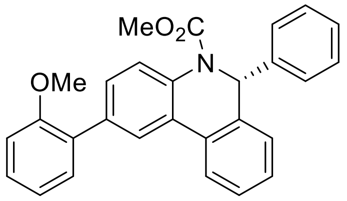
MeO$_2$C
N
OMe

4i

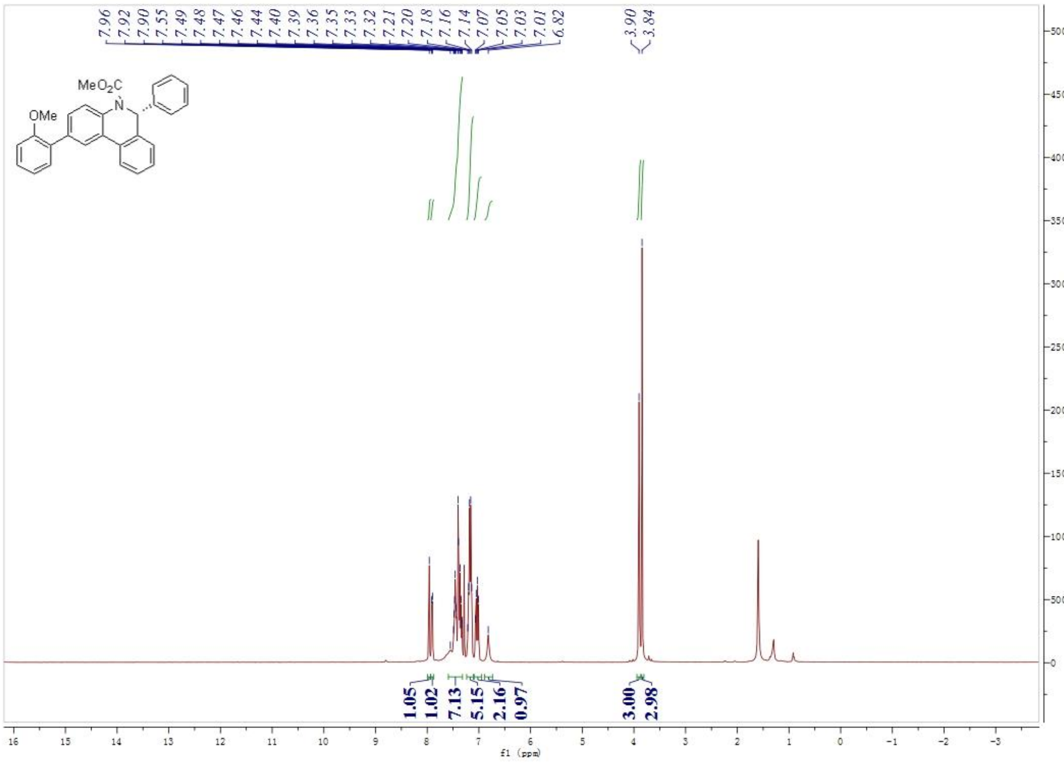

MeO2C
N
OMe
7.96 7.92 7.90 7.55 7.49 7.48 7.47 7.46 7.44 7.40 7.39 7.36 7.35 7.33 7.32 7.21 7.20 7.18 7.16 7.14 7.07 7.05 7.03 7.01 6.82
3.90 3.84
1.05 1.02 7.13 5.15 2.16 0.97
3.00 2.98
f1 (ppm)

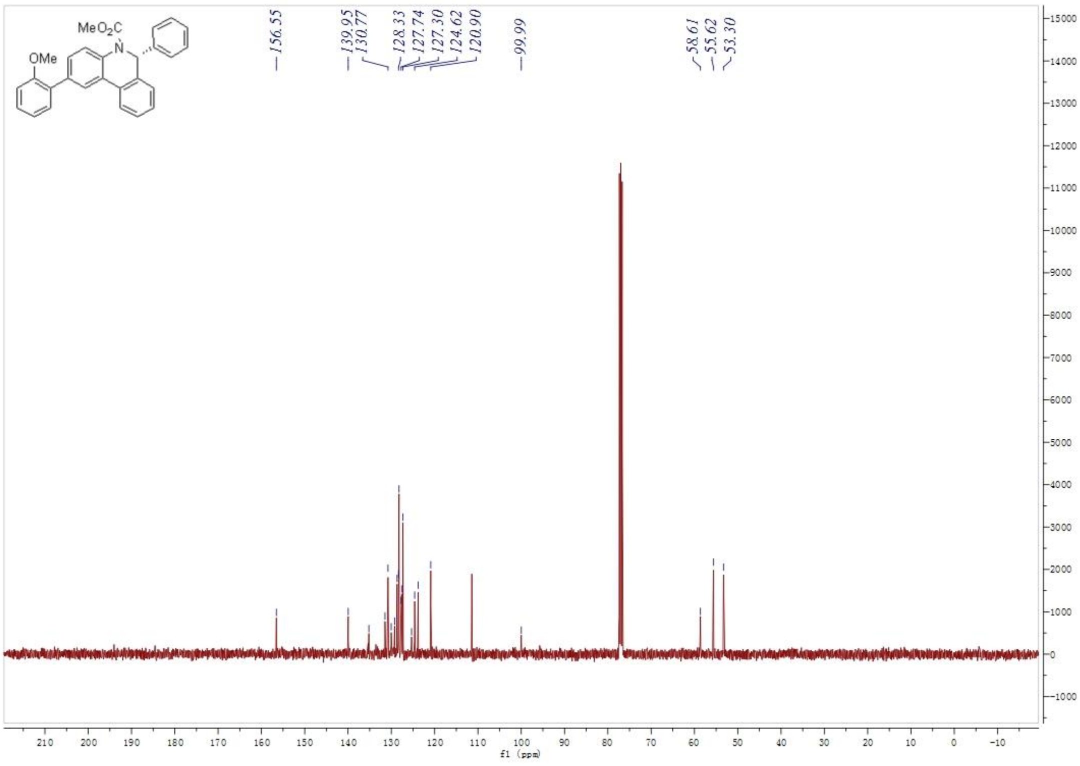

MeO2C
N
OMe
156.55
139.95 130.77
128.33 127.74 127.30 124.62 120.90
99.99
58.61 55.62 53.30
f1 (ppm)

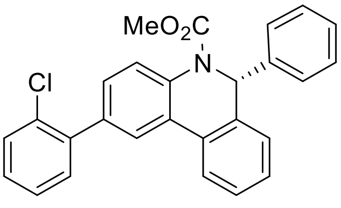

MeO2C
N
Cl

4j

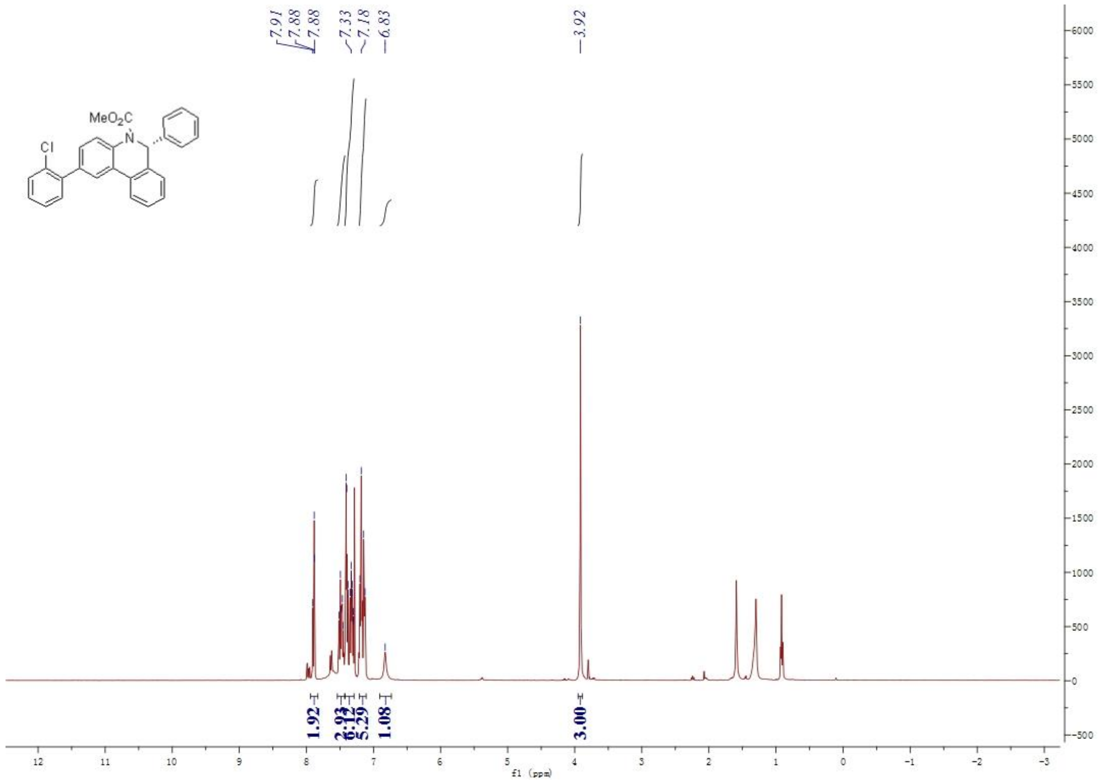
MeO2C
N
Cl
7.91
7.88
7.88
7.33
7.18
6.83
3.92
1.92
2.93
6.12
5.29
1.08
3.00
f1 (ppm)

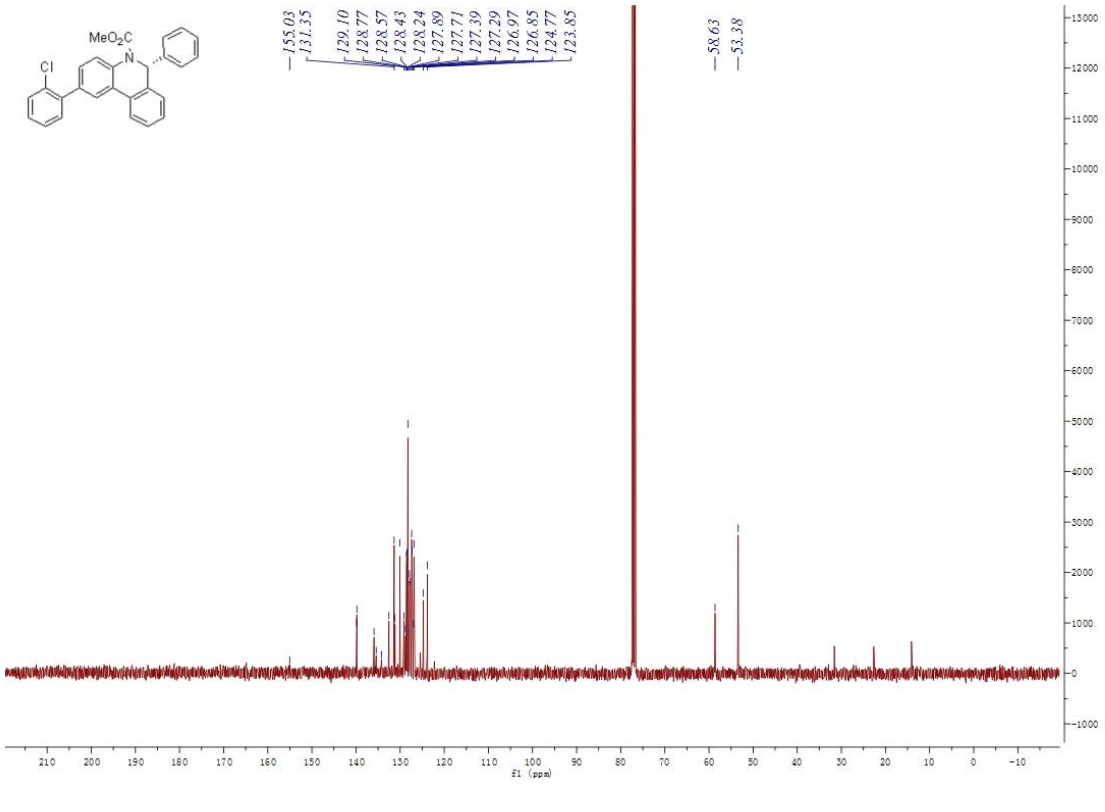
MeO2C
N
Cl
155.03
131.35
129.10
128.77
128.57
128.43
128.24
127.89
127.71
127.39
127.29
126.97
126.85
124.77
123.85
58.63
53.38
f1 (ppm)

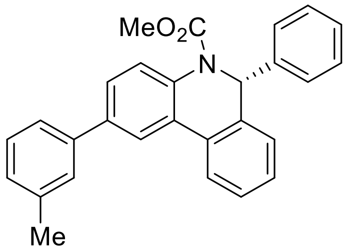

MeO2C
N
Me

4k

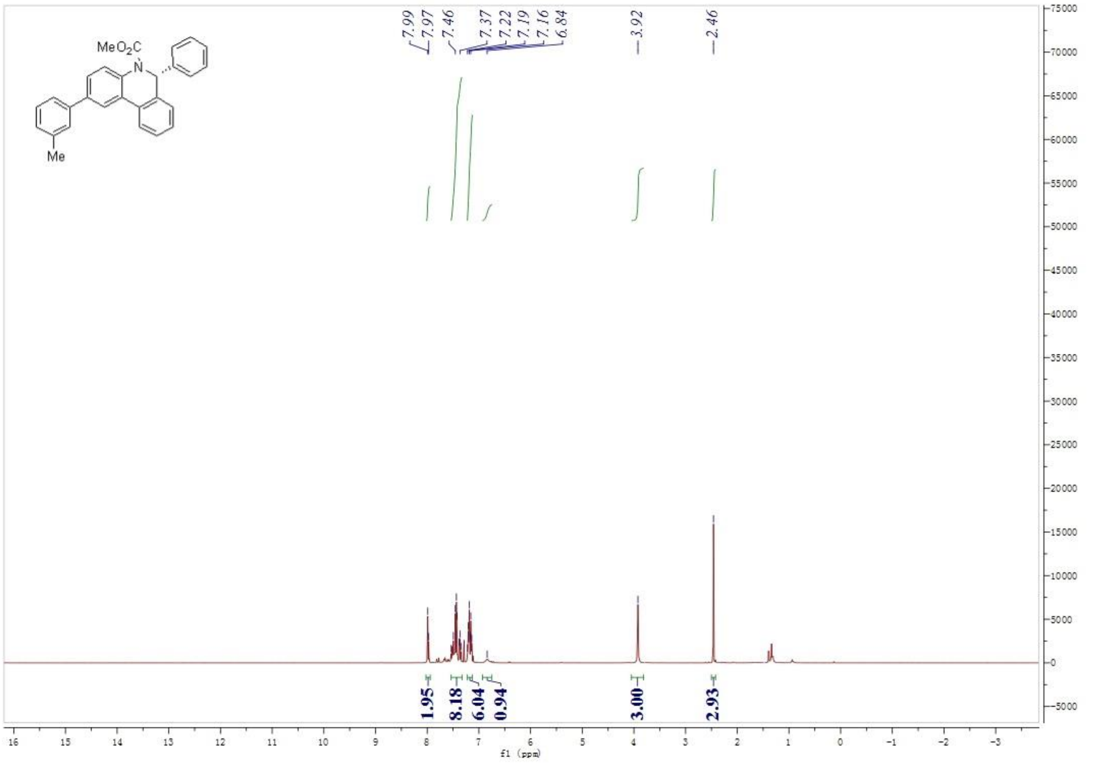

7.99
7.97
7.46
7.37
7.22
7.19
7.16
6.84
3.92
2.46
1.95
8.18
6.04
0.94
3.00
2.93
f1 (ppm)

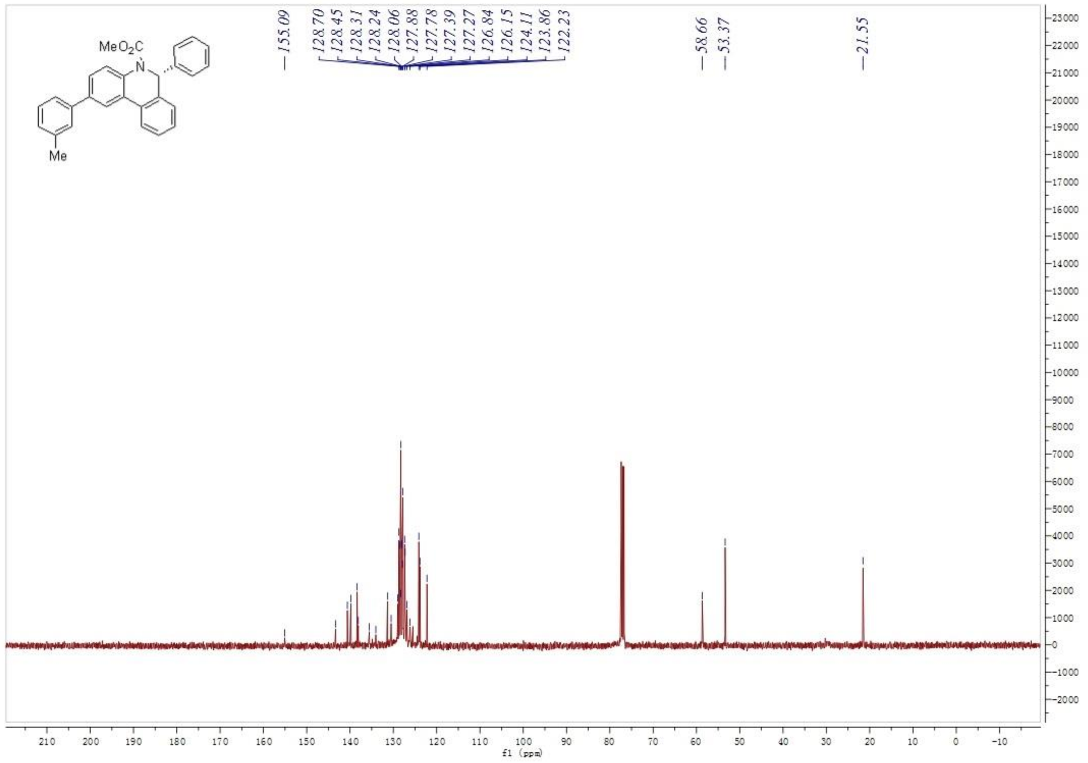
MeO2C
N
Me
155.09
128.70
128.45
128.31
128.24
128.06
127.88
127.78
127.39
127.27
126.84
126.15
124.11
123.86
122.23
58.66
53.37
21.55
f1 (ppm)

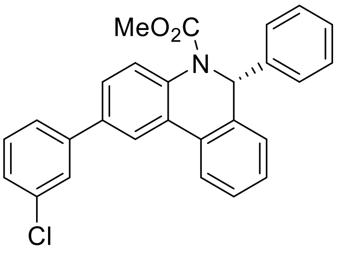
MeO2C
N
Cl

4k

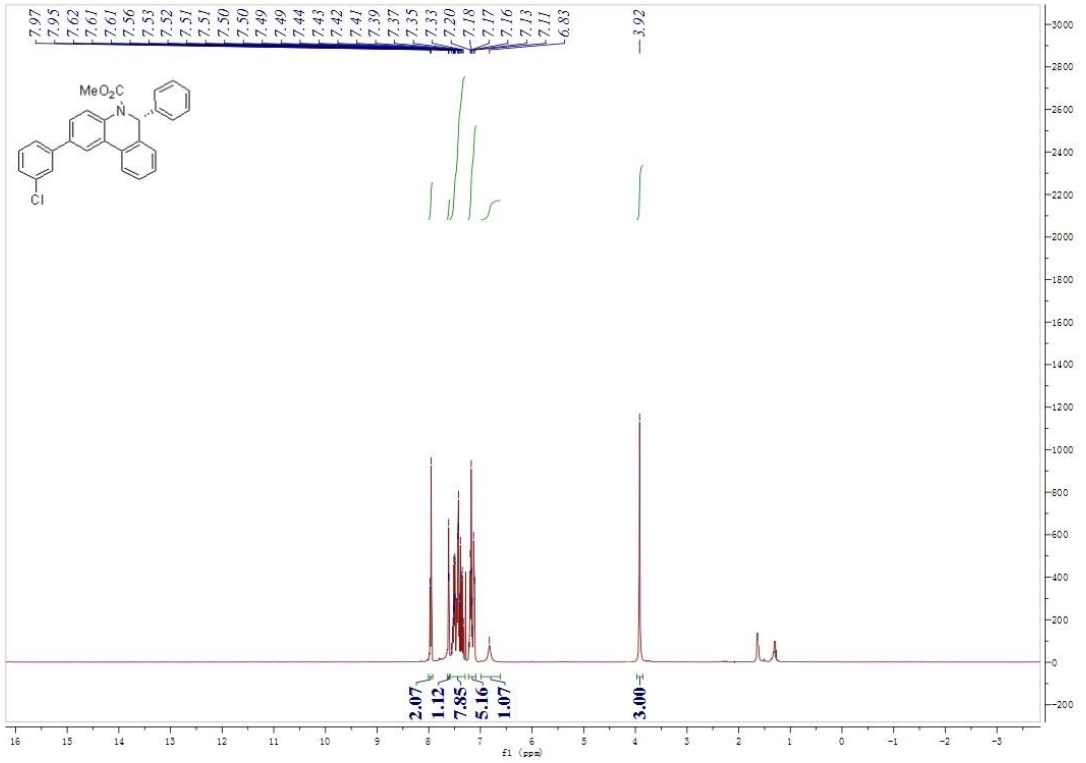
7.97 7.95 7.62 7.61 7.61 7.56 7.53 7.52 7.51 7.51 7.50 7.50 7.49 7.49 7.44 7.43 7.42 7.41 7.39 7.37 7.35 7.33 7.20 7.18 7.17 7.16 7.13 7.11 6.83
3.92
MeO2C
N
Cl
2.07
1.12
7.85
5.16
1.07
3.00
f1 (ppm)

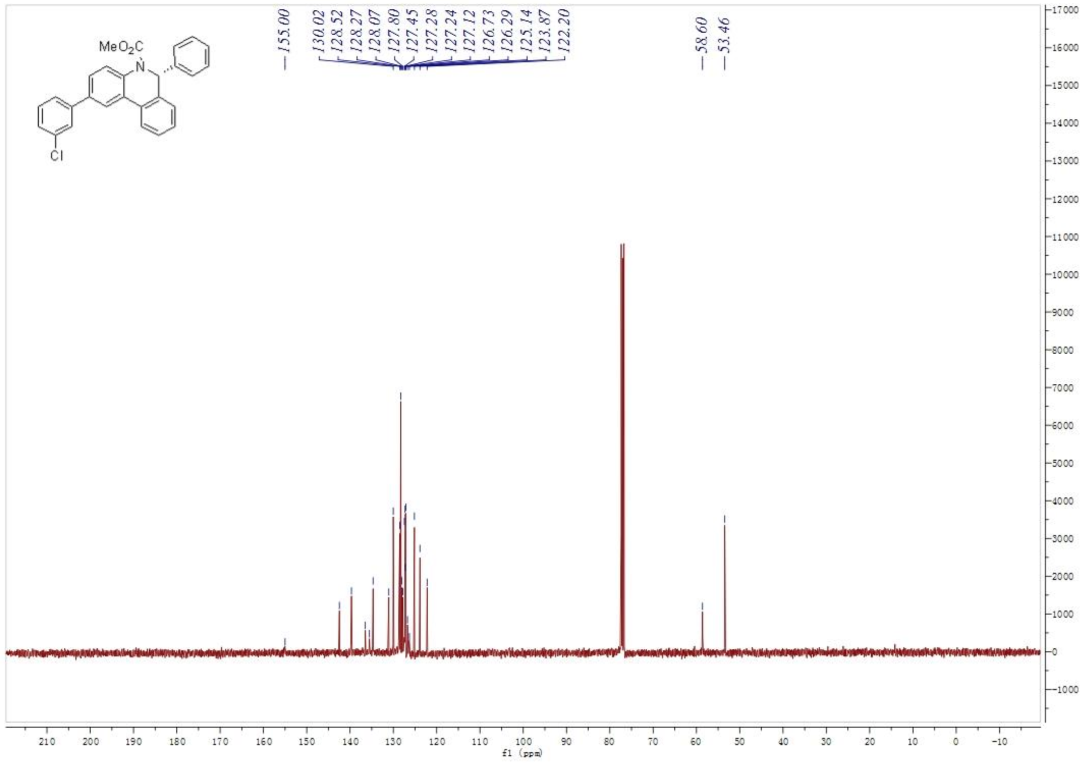
155.00
130.02 128.52 128.27 128.07 127.80 127.45 127.28 127.24 127.12 126.73 126.29 125.14 123.87 122.20
58.60
53.46
MeO2C
N
Cl
f1 (ppm)

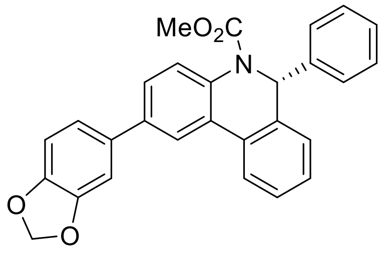
MeO2C
N
O
O

4m

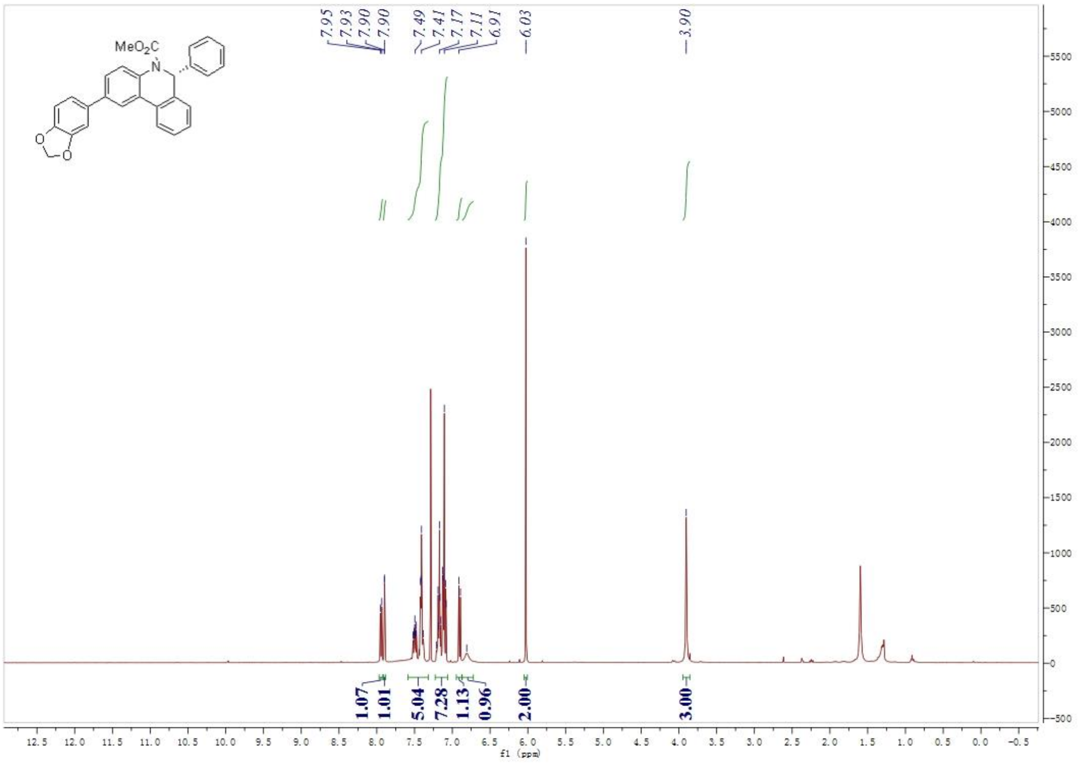

MeO2C
N
O
O
7.95
7.93
7.90
7.90
7.49
7.41
7.17
7.11
6.91
6.03
3.90
1.07
1.01
5.04
7.28
1.13
0.96
2.00
3.00
f1 (ppm)

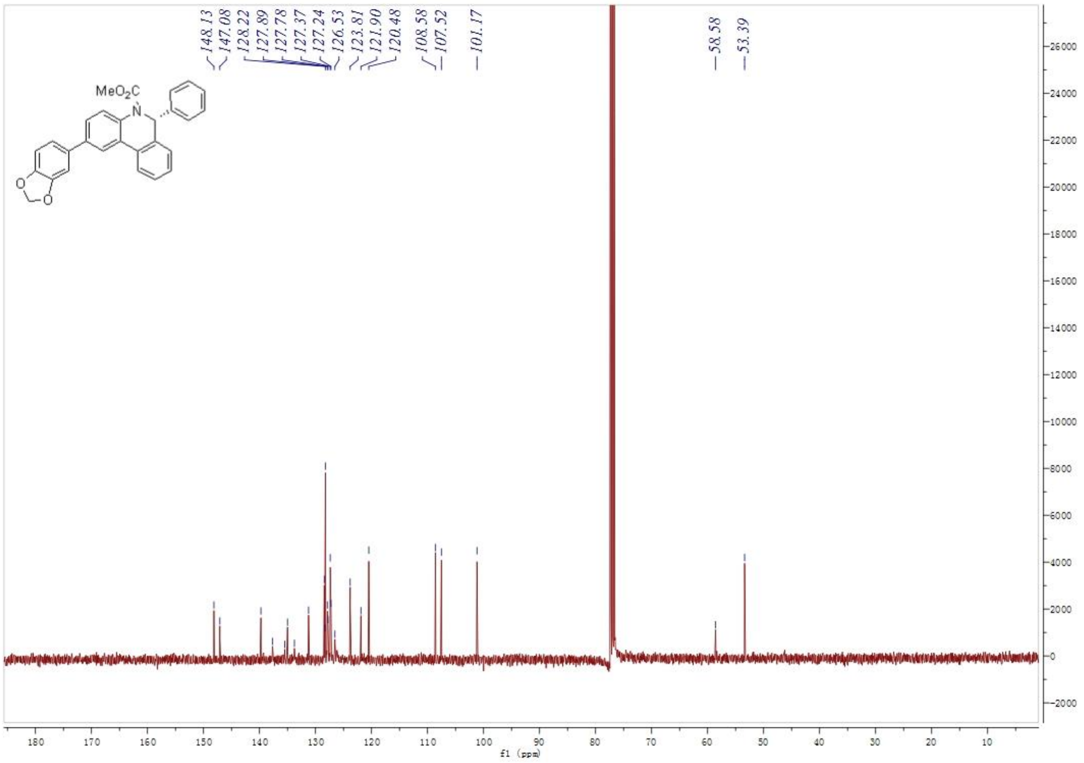

MeO2C
N
O
O
148.13
147.08
128.22
127.89
127.78
127.37
127.24
126.53
123.81
121.90
120.48
108.58
107.52
101.17
58.58
53.39
f1 (ppm)

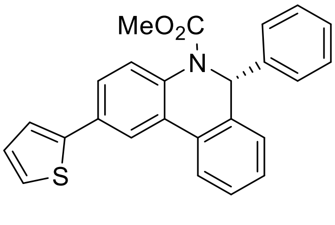

MeO2C
N
S

4o

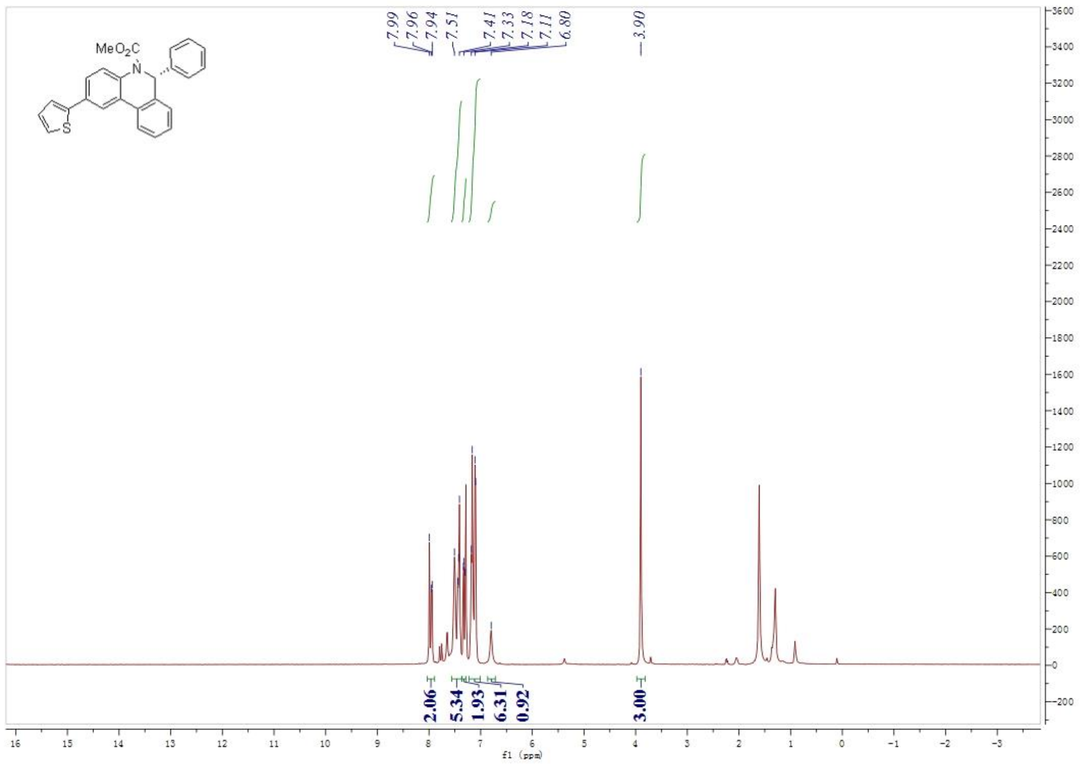

MeO2C
N
S
7.99
7.96
7.94
7.51
7.41
7.33
7.18
7.11
6.80
3.90
2.06
5.34
1.93
6.31
0.92
3.00
f1 (ppm)

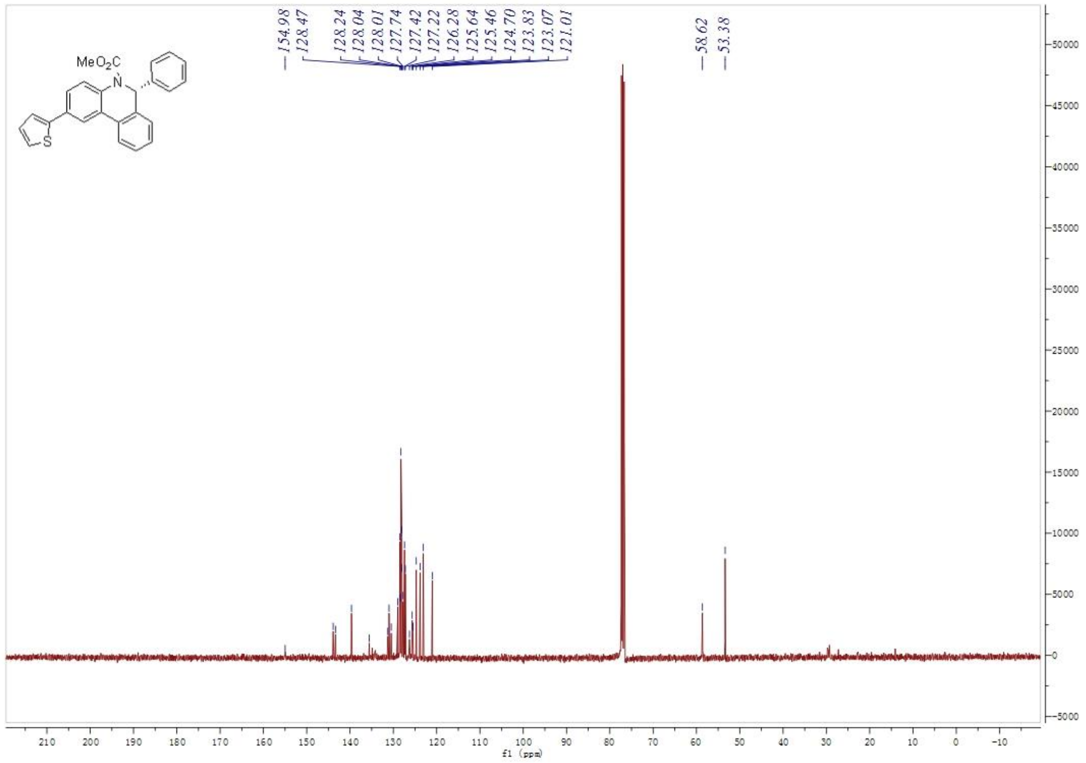

MeO2C
N
S
154.98
128.47
128.24
128.04
128.01
127.74
127.42
127.22
126.28
125.64
125.46
124.70
123.83
123.07
121.01
58.62
53.38
f1 (ppm)

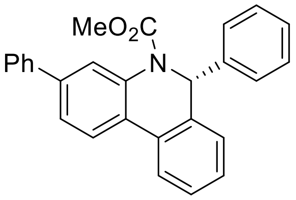

MeO2C
Ph
N

4o

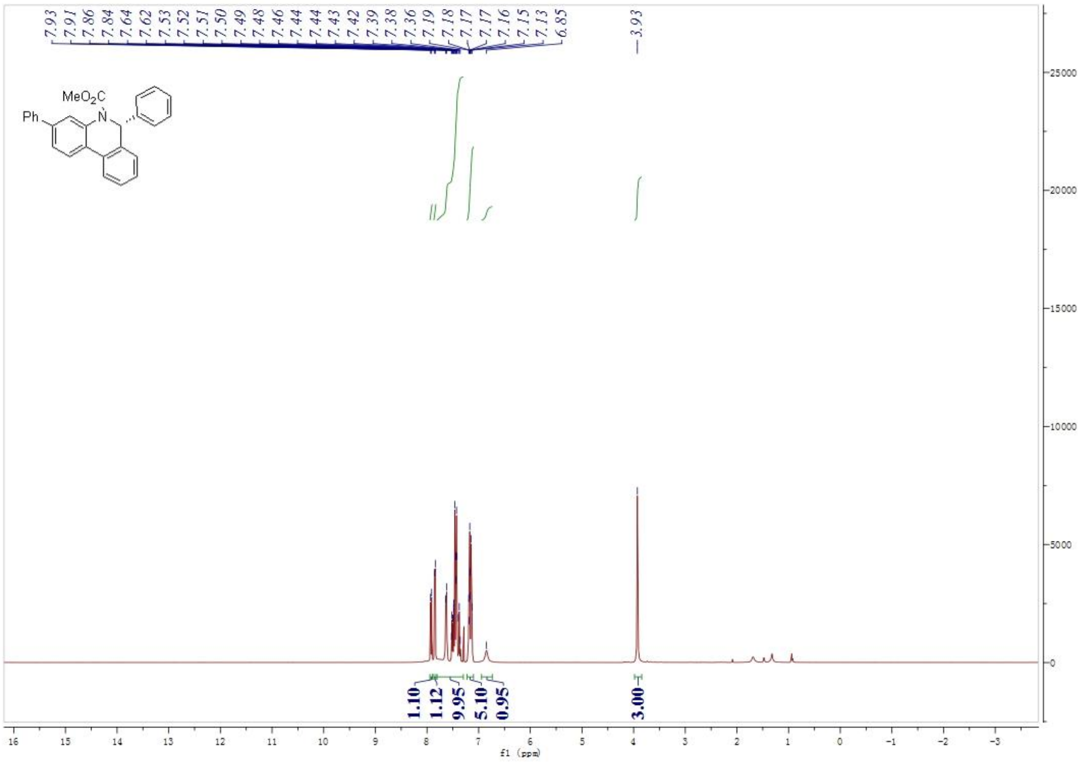
7.93
7.91
7.86
7.84
7.64
7.62
7.53
7.52
7.51
7.50
7.49
7.48
7.46
7.44
7.44
7.43
7.42
7.39
7.38
7.36
7.19
7.18
7.17
7.17
7.16
7.15
7.13
6.85
3.93
MeO2C
N
Ph
1.10
1.12
9.95
5.10
0.95
3.00
f1 (ppm)

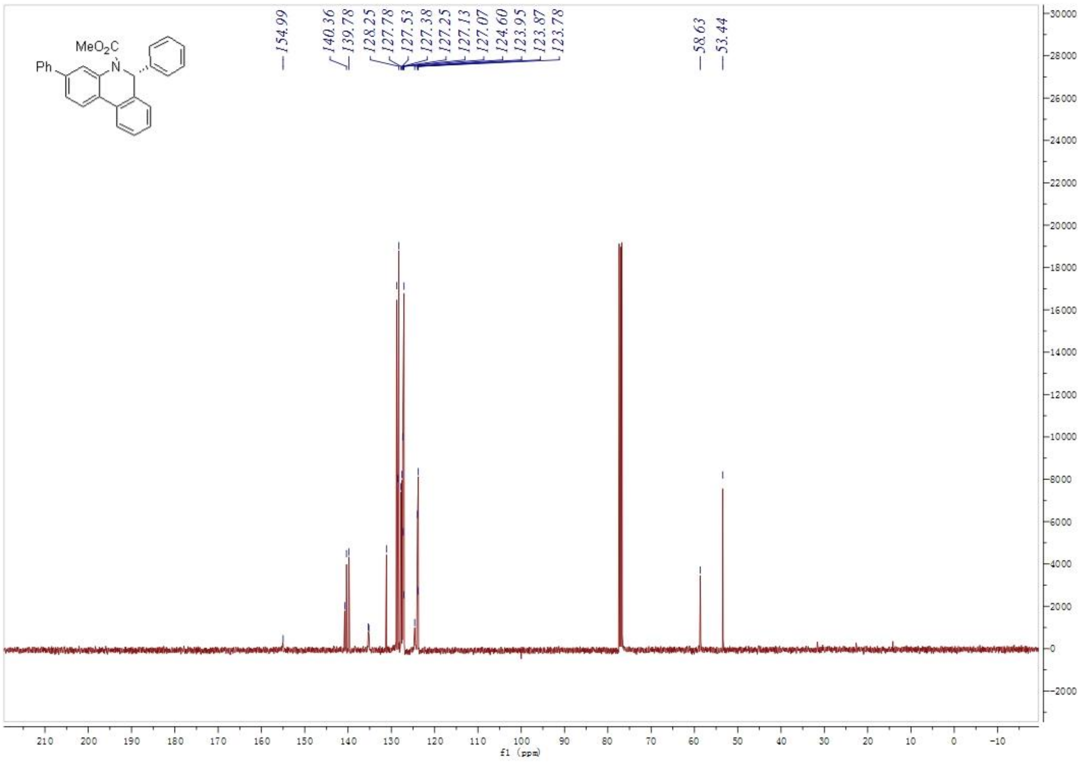
154.99
140.36
139.78
128.25
127.78
127.53
127.38
127.25
127.13
127.07
124.60
123.95
123.87
123.78
58.63
53.44
MeO2C
N
Ph
f1 (ppm)

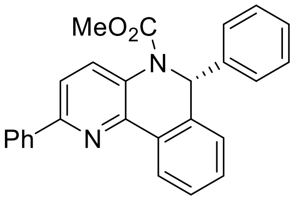
MeO2C
N
Ph
N

4p

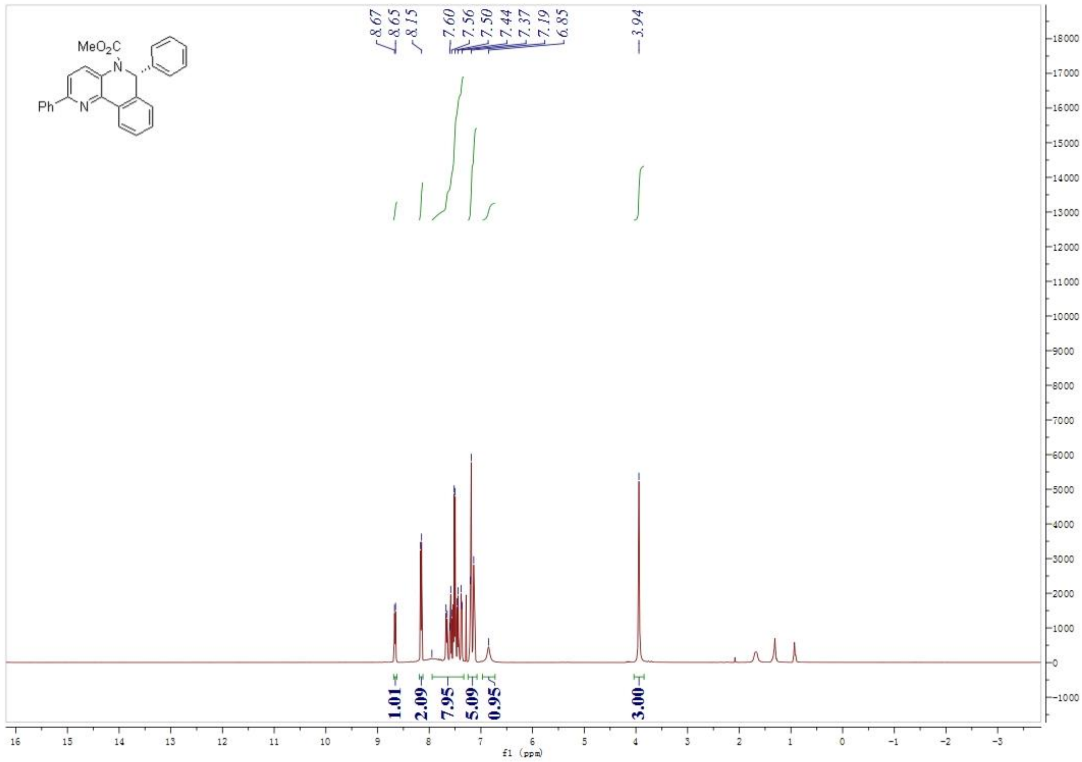
MeO2C
N
Ph
N
8.67
8.65
8.15
7.60
7.56
7.50
7.44
7.37
7.19
6.85
3.94
1.01
2.09
7.95
5.09
0.95
3.00
f1 (ppm)

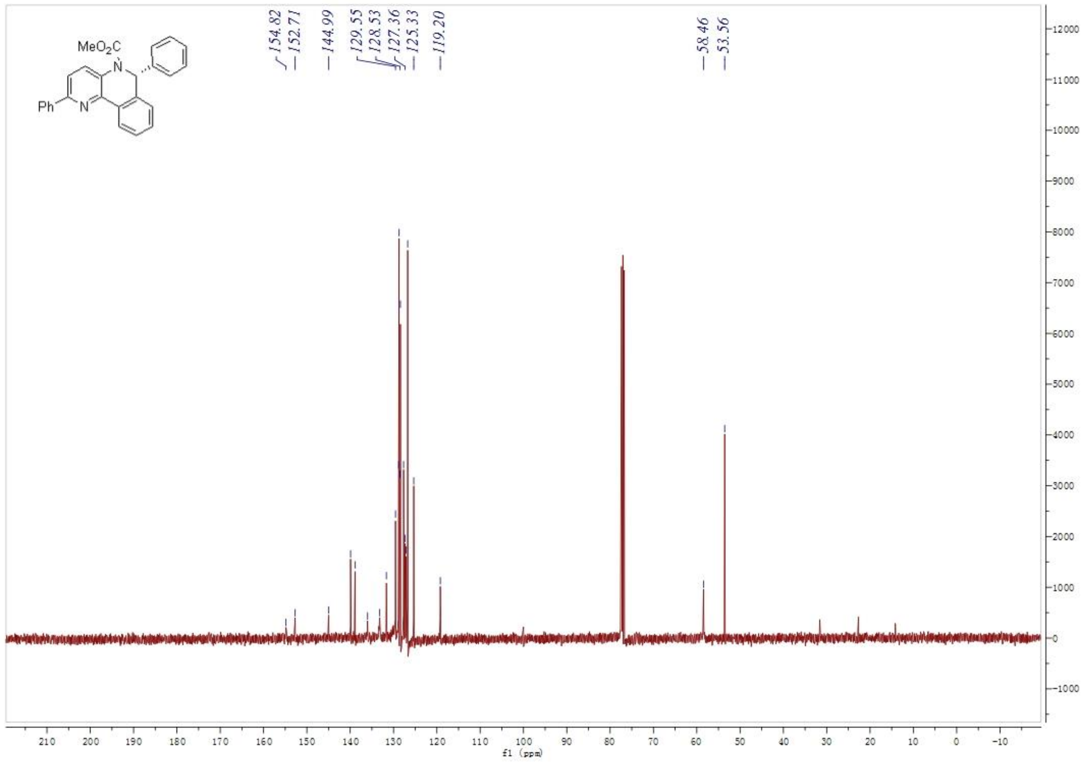
MeO2C
N
Ph
N
154.82
152.71
144.99
129.55
128.53
127.36
125.33
119.20
58.46
53.56
f1 (ppm)

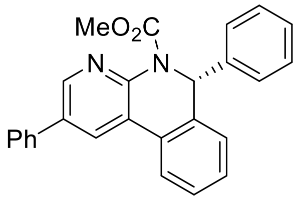
MeO2C
N
N
Ph

4q

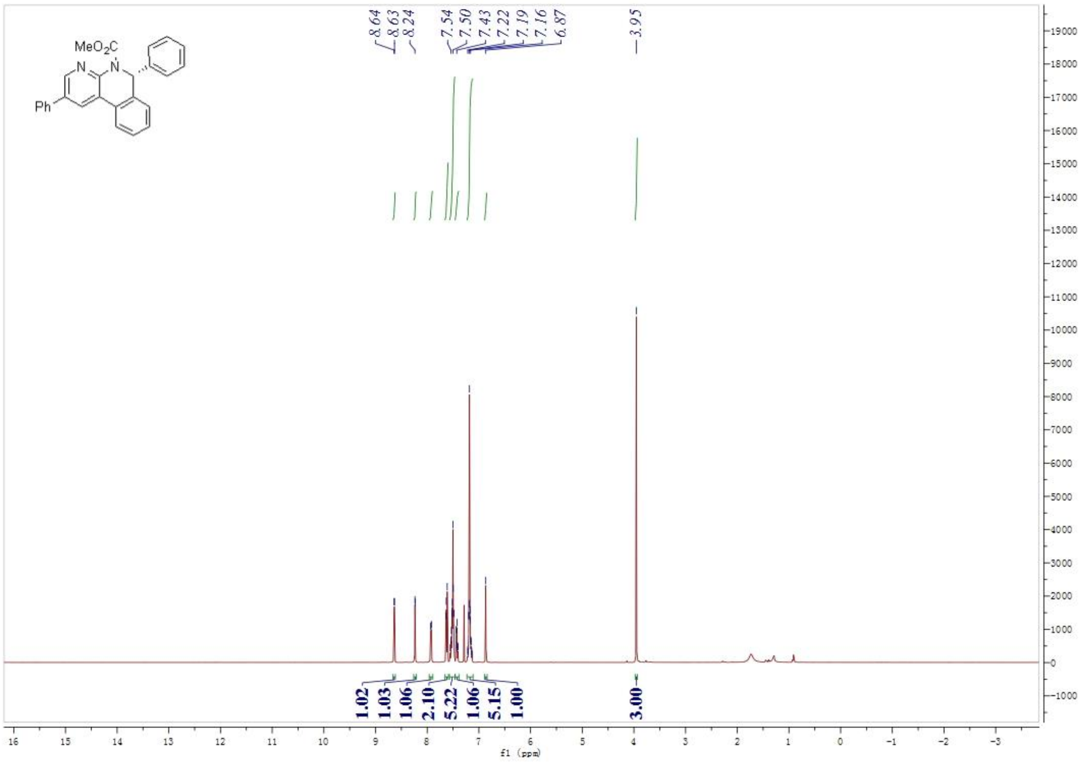

MeO2C
N
N
Ph
8.64
8.63
8.24
7.54
7.50
7.43
7.22
7.19
7.16
6.87
3.95
1.02
1.03
1.06
2.10
5.22
1.06
5.15
1.00
3.00
f1 (ppm)

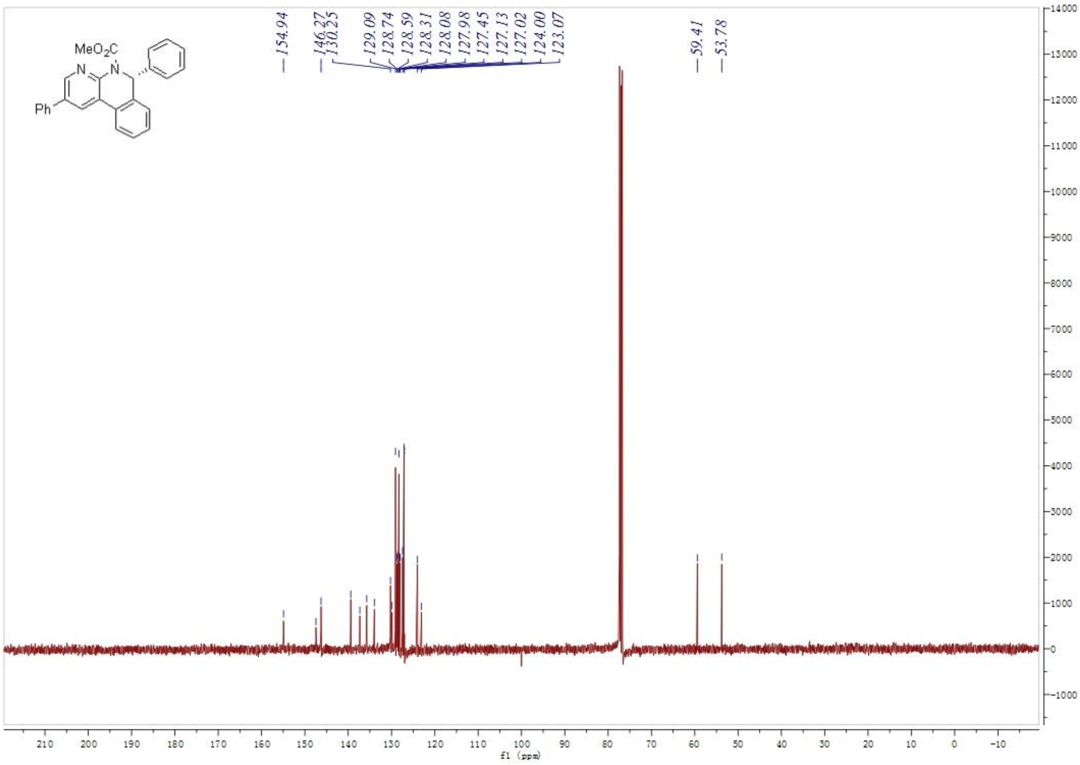

MeO2C
N
N
Ph
154.94
146.27
130.25
129.09
128.74
128.59
128.31
128.08
127.98
127.45
127.13
127.02
124.00
123.07
59.41
53.78
f1 (ppm)

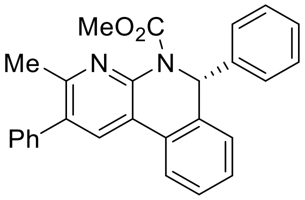

MeO2C
Me
N
N
Ph

4r

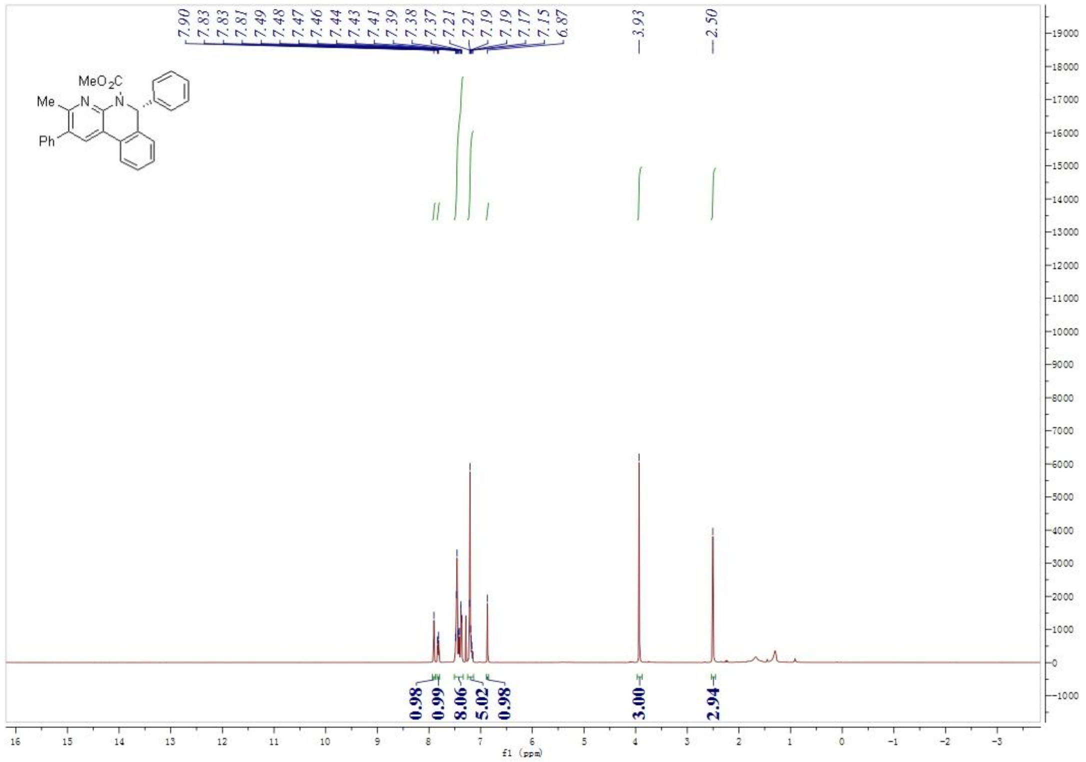

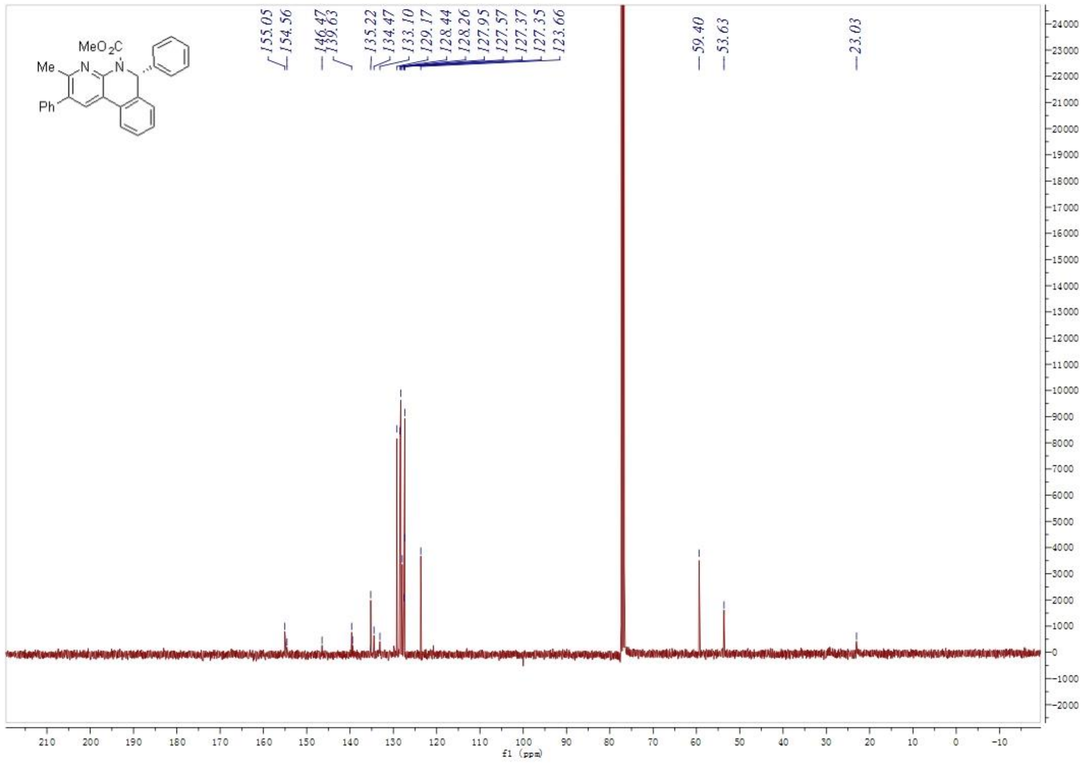

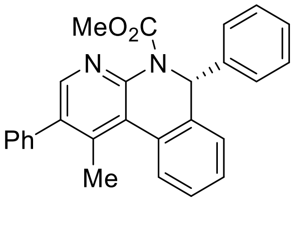

4s

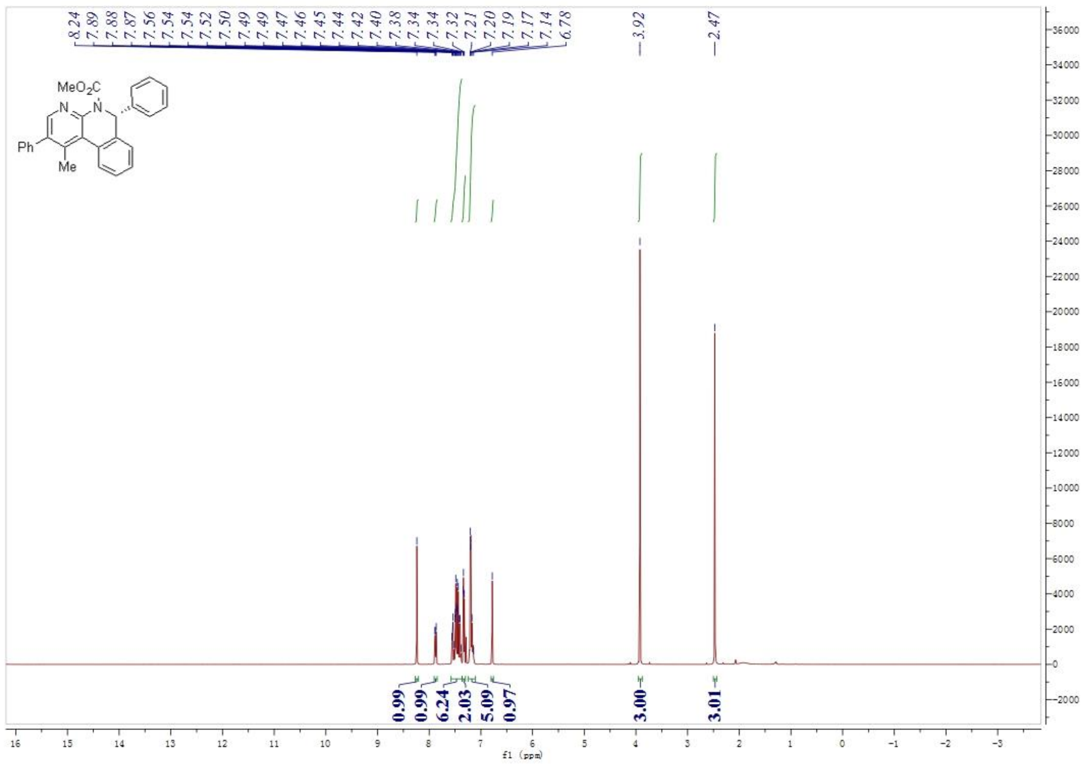

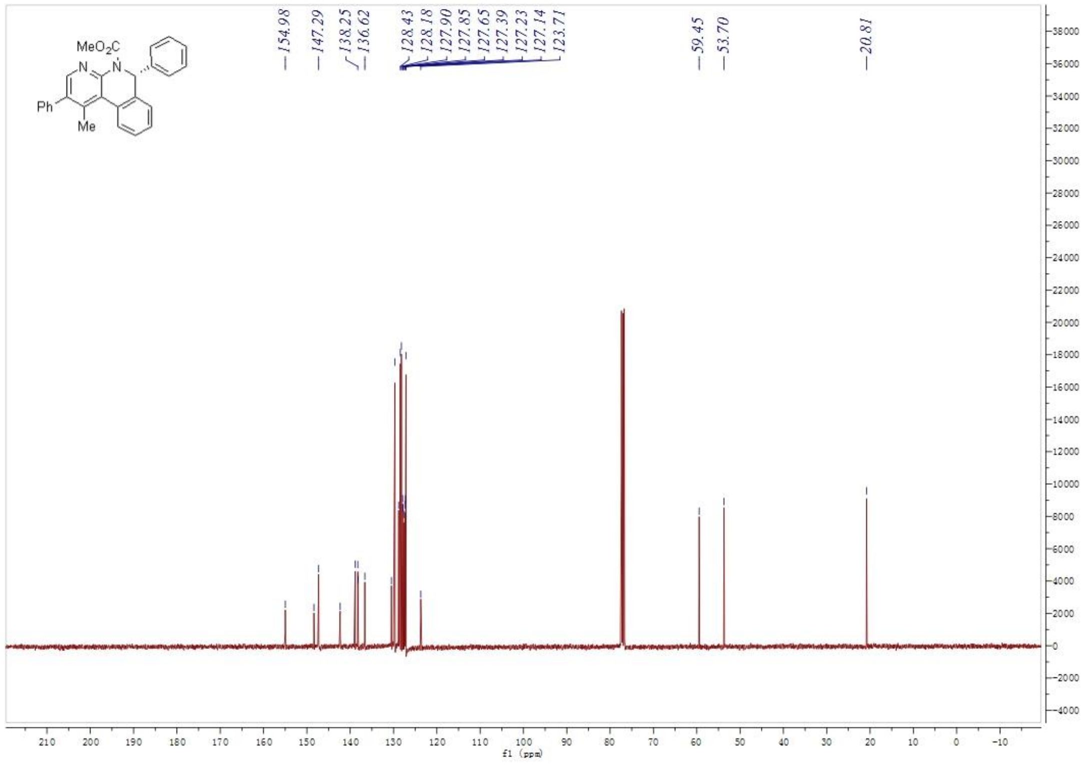

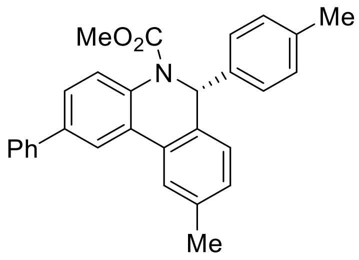

4s

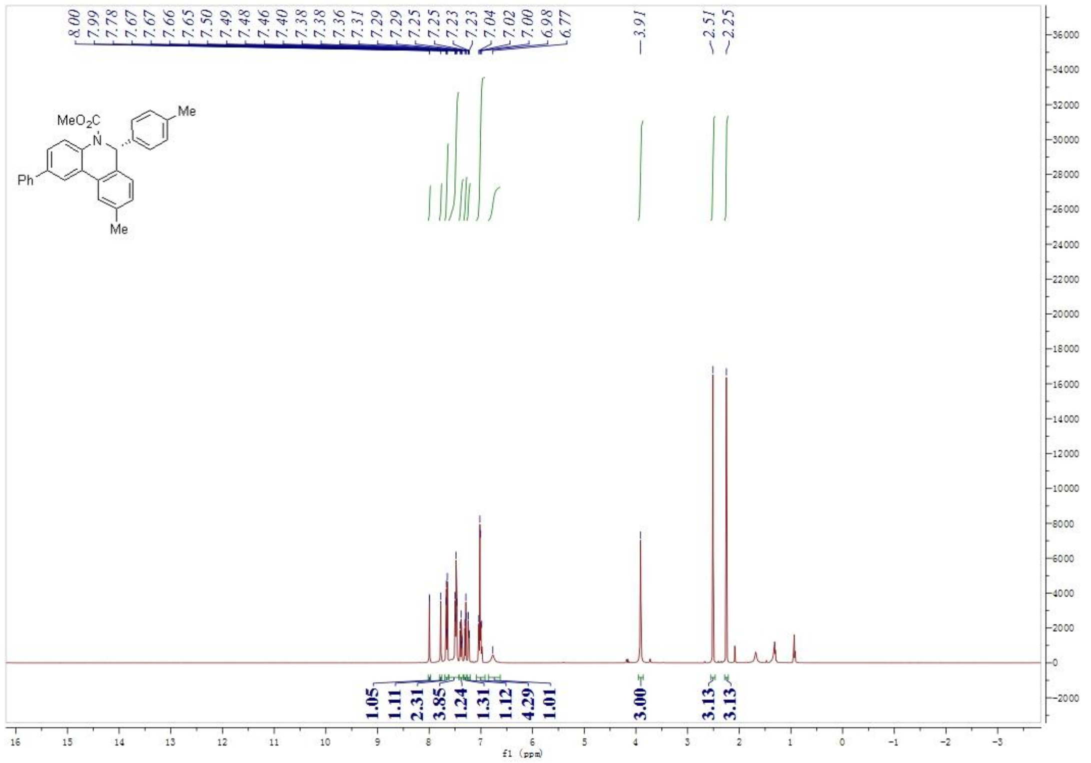

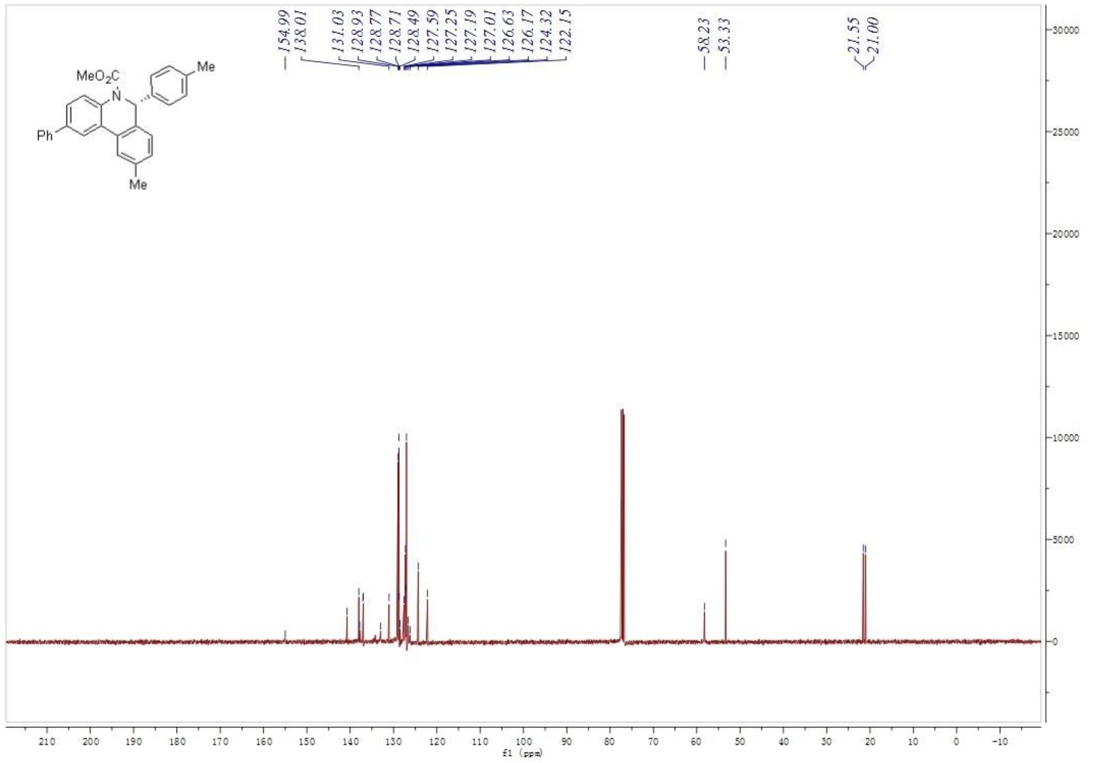

154.99
138.01
131.03
128.93
128.77
128.71
128.49
127.59
127.25
127.19
127.01
126.63
126.17
124.32
122.15
58.23
53.33
21.55
21.00
MeO2C
N
Me
Ph
Me
30000
25000
20000
15000
10000
5000
0
210 200 190 180 170 160 150 140 130 120 110 100 90 80 70 60 50 40 30 20 10 0 -10
f1 (ppm)

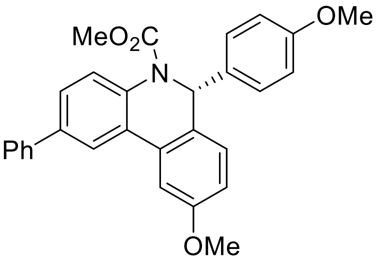

MeO2C
N
OMe
Ph
OMe

4u

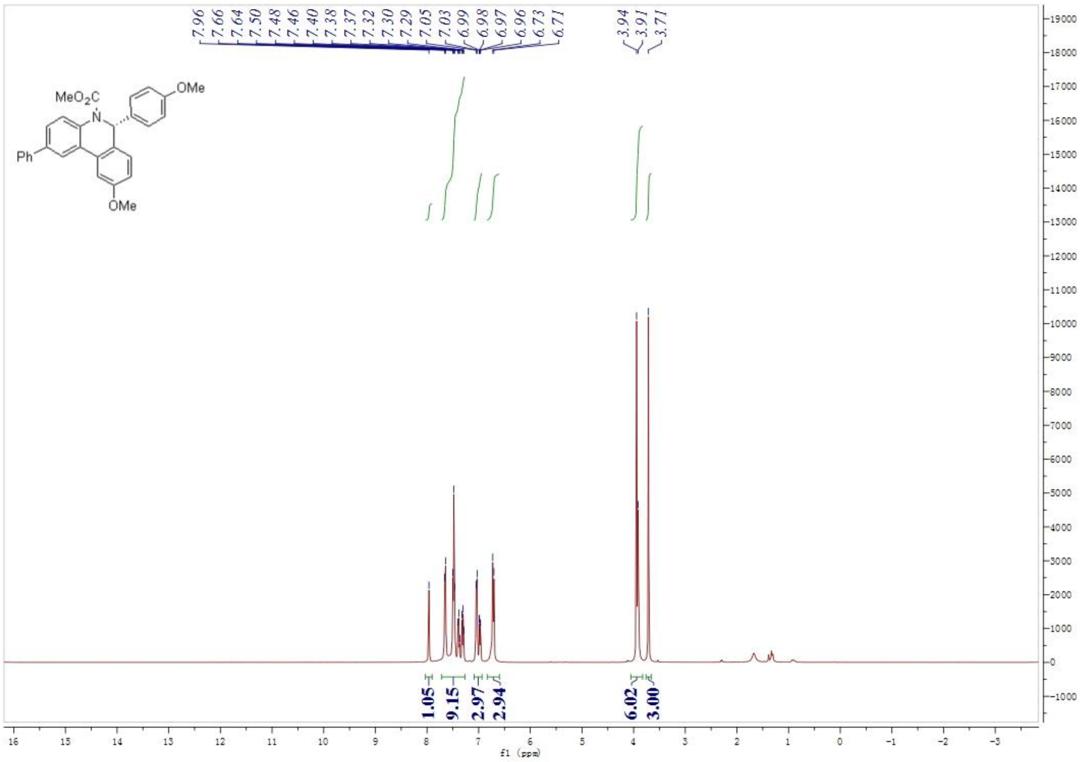
7.96 7.66 7.64 7.50 7.48 7.46 7.40 7.38 7.37 7.32 7.30 7.29 7.05 7.03 6.99 6.98 6.97 6.96 6.73 6.71
3.94 3.91 3.71
MeO2C
N
OMe
Ph
OMe
1.05
9.15
2.97
2.94
6.02
3.00
f1 (ppm)

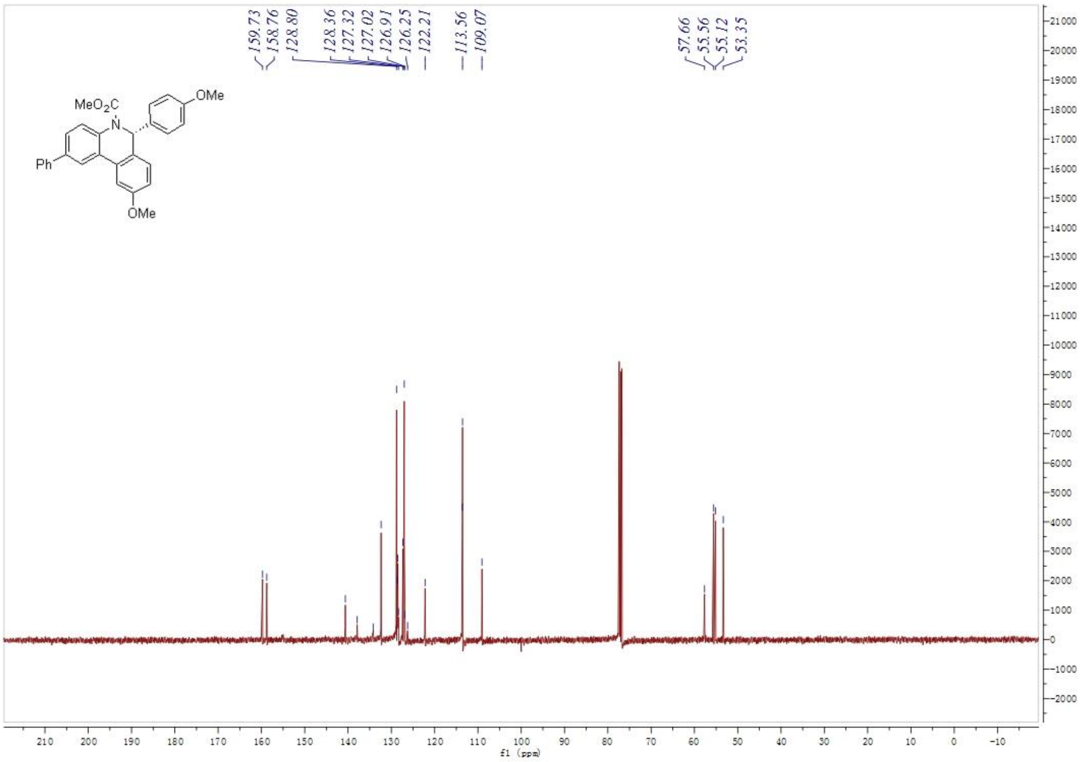
159.73 158.76 128.80 128.36 127.32 127.02 126.91 126.25 122.21 113.56 109.07
57.66 55.56 55.12 53.35
MeO2C
N
OMe
Ph
OMe
f1 (ppm)

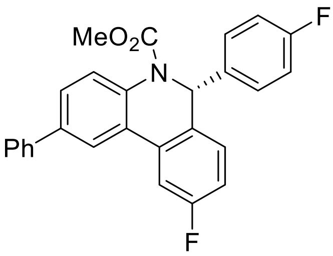
MeO2C
N
F
Ph
F

4v

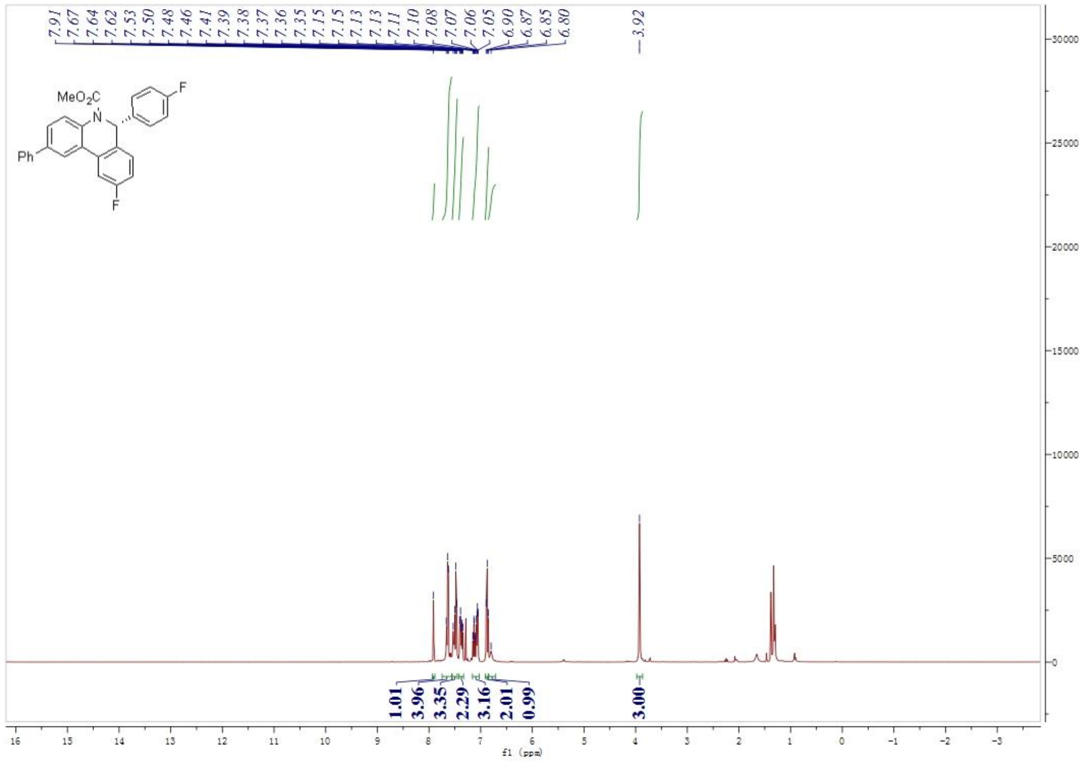

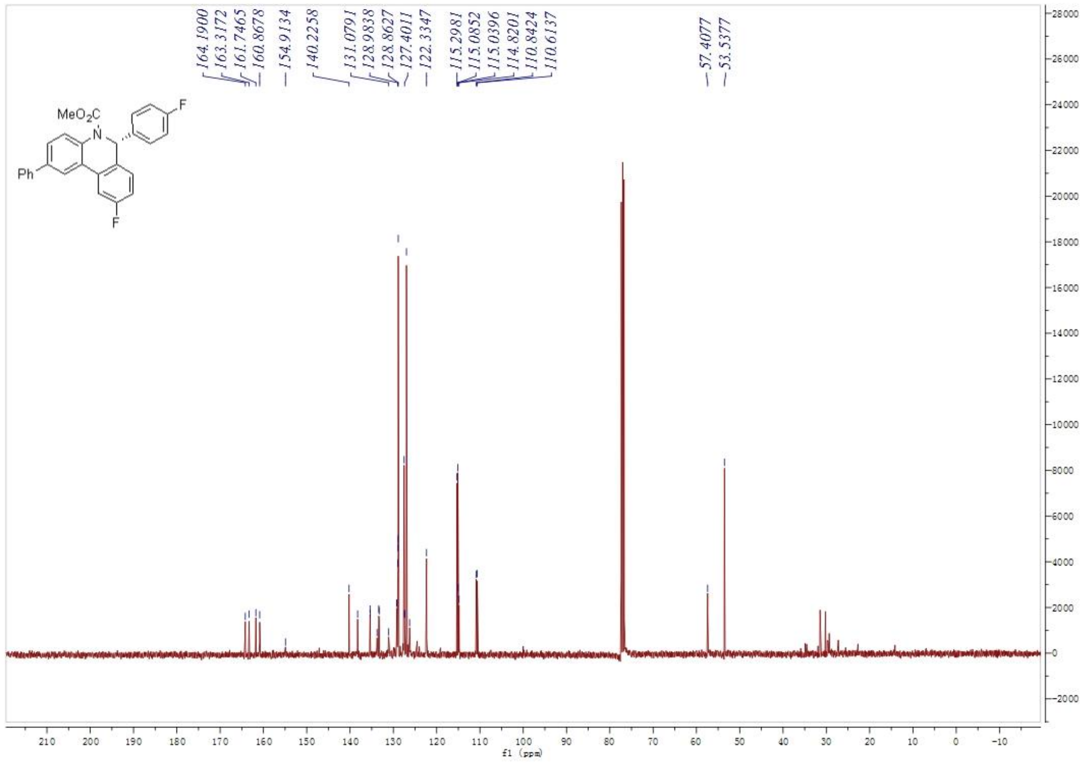

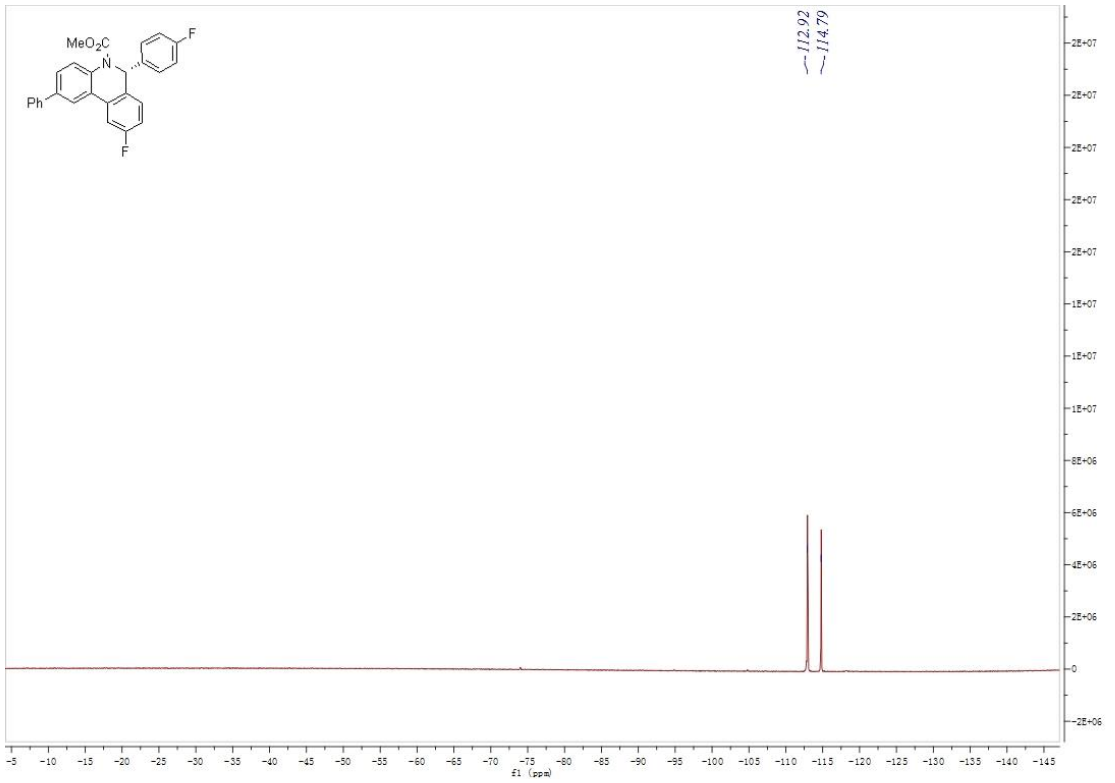

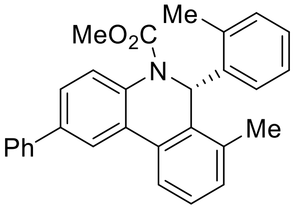

4w

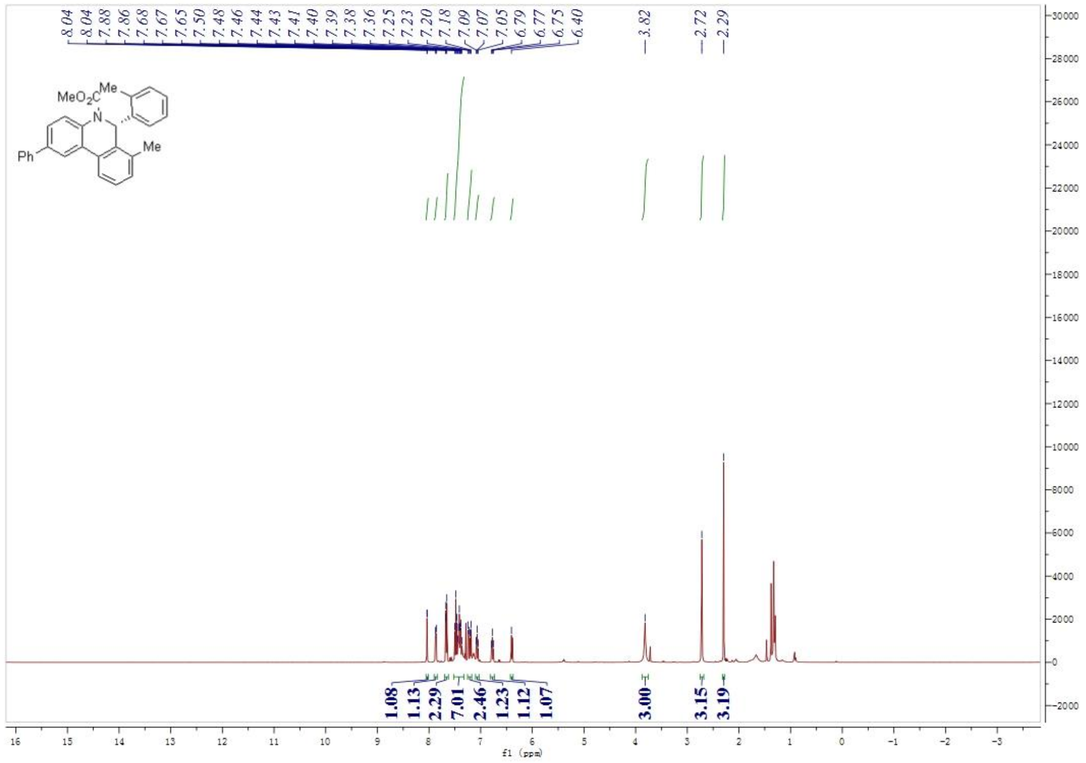

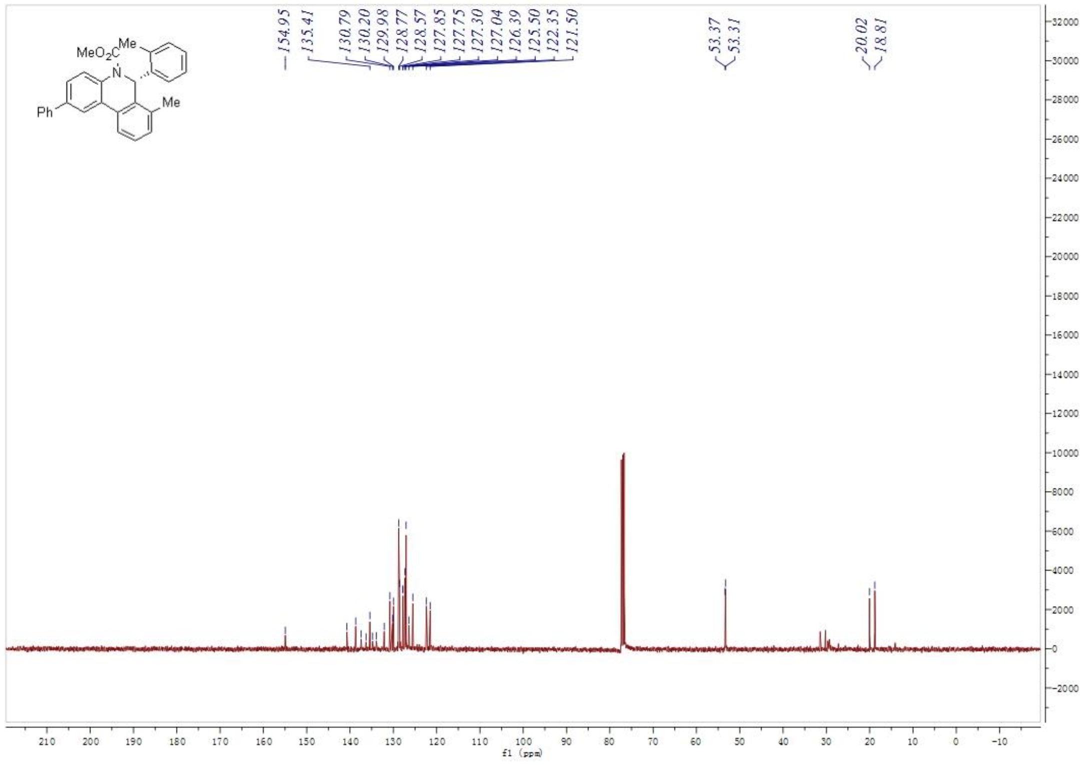

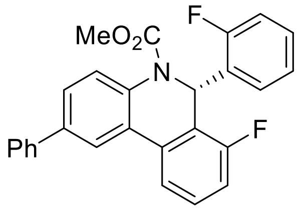

4x

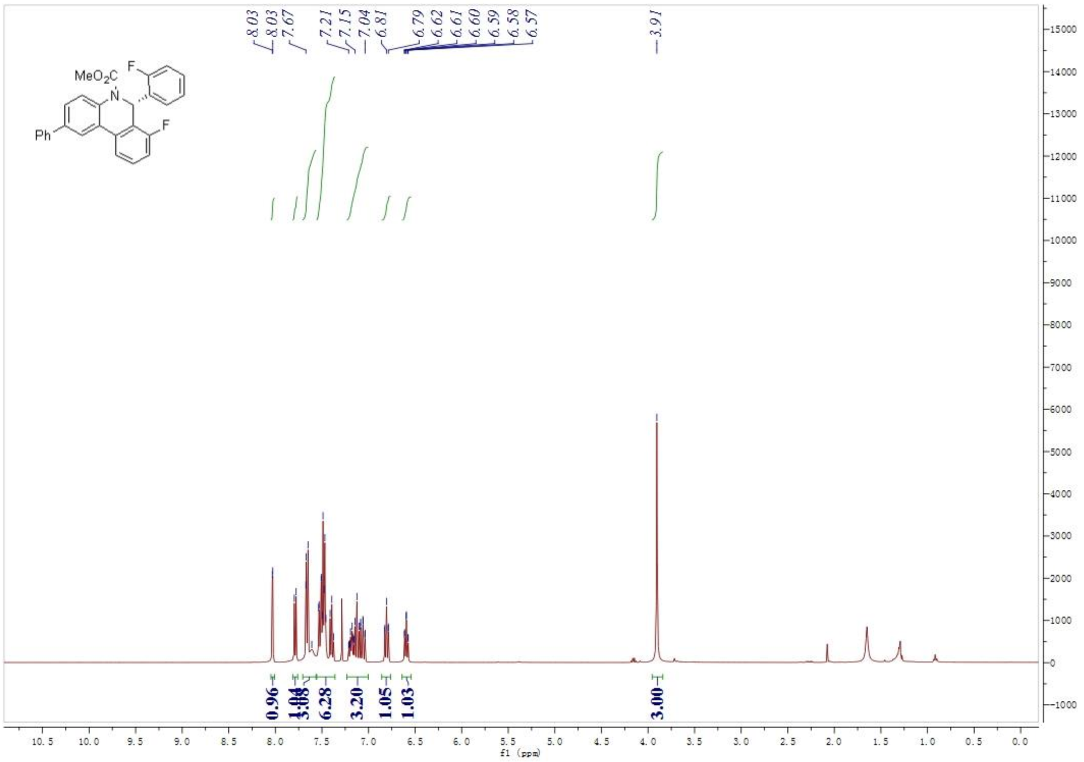
MeO2C
F
N
F
Ph
8.03
8.03
7.67
7.21
7.15
7.04
6.81
6.79
6.62
6.61
6.60
6.59
6.58
6.57
3.91
0.96
1.04
3.08
6.28
3.20
1.05
1.03
3.00
f1 (ppm)

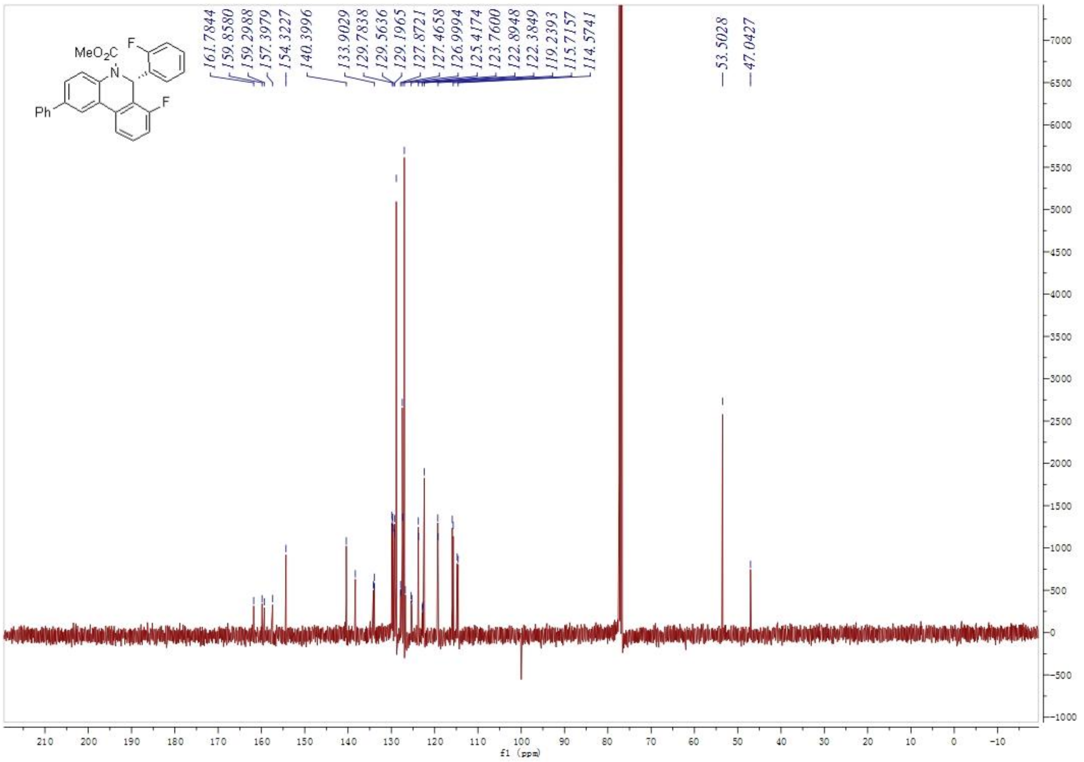
MeO2C
F
N
F
Ph
161.7844
159.8580
159.2988
157.3979
154.3227
140.3996
133.9029
129.7838
129.5636
129.1965
127.8721
127.4658
126.9994
125.4174
123.7600
122.8948
122.3849
119.2393
115.7157
114.5741
53.5028
47.0427
f1 (ppm)

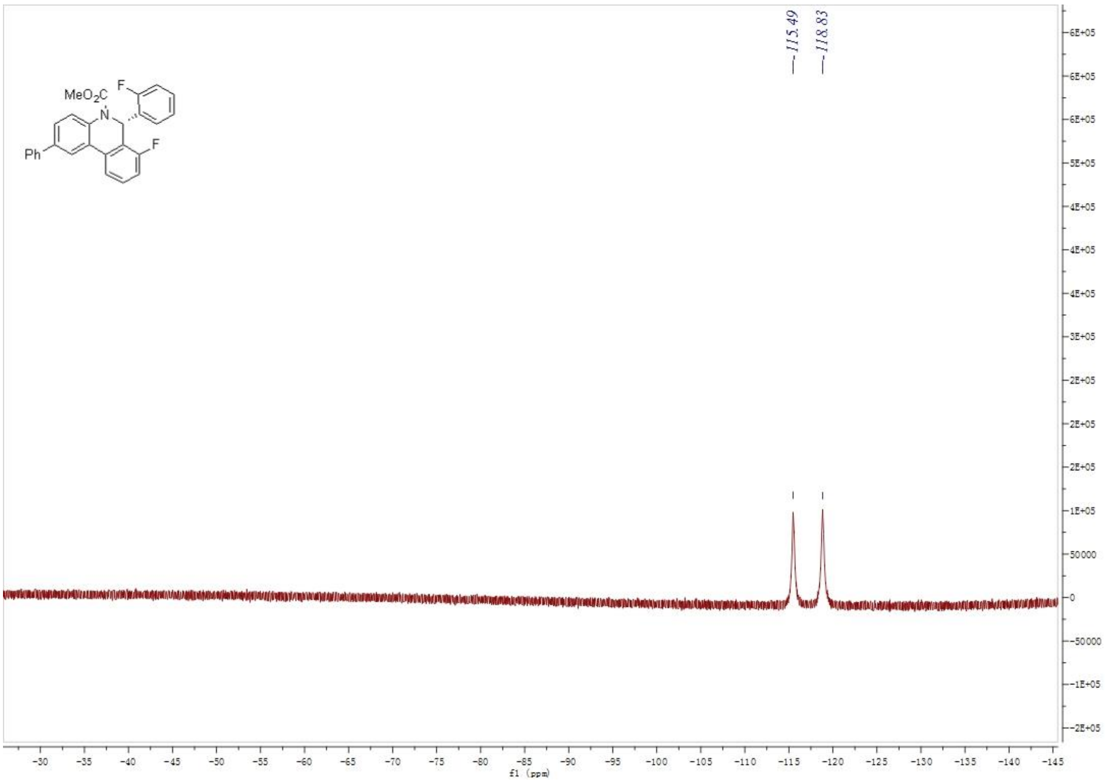
-115.49
-118.83
MeO2C
F
N
F
Ph
6E+05
5E+05
4E+05
3E+05
2E+05
1E+05
50000
0
-50000
-1E+05
-2E+05
-30 -35 -40 -45 -50 -55 -60 -65 -70 -75 -80 -85 -90 -95 -100 -105 -110 -115 -120 -125 -130 -135 -140 -145
f1 (ppm)

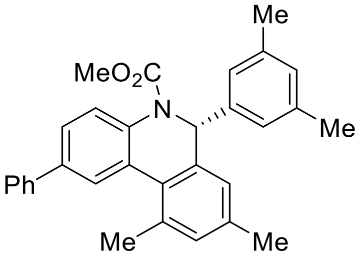
Me
MeO2C
N
Me
Ph
Me
Me

4y

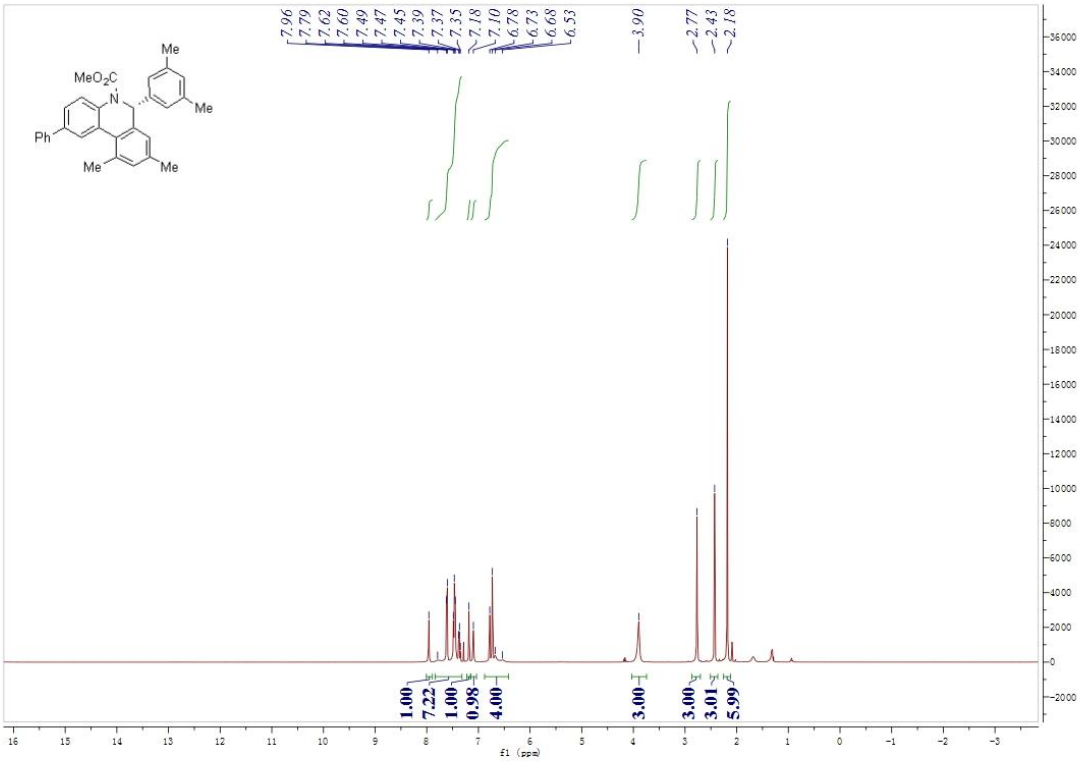
7.96
7.79
7.62
7.60
7.49
7.47
7.45
7.39
7.37
7.35
7.18
7.10
6.78
6.73
6.68
6.53
3.90
2.77
2.43
2.18
MeO2C
N
Me
Me
Ph
Me
Me
1.00
7.22
1.00
0.98
4.00
3.00
3.00
3.01
5.99
f1 (ppm)

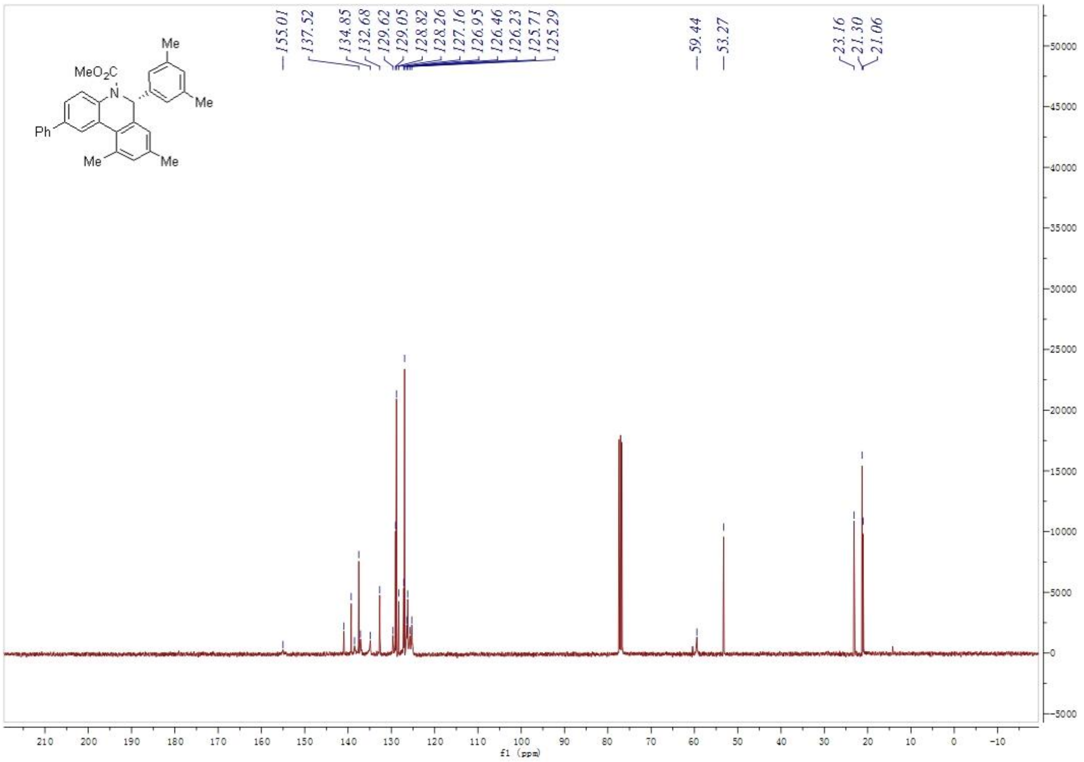
155.01
137.52
134.85
132.68
129.62
129.05
128.82
128.26
127.16
126.95
126.46
126.23
125.71
125.29
59.44
53.27
23.16
21.30
21.06
MeO2C
N
Me
Me
Ph
Me
Me
f1 (ppm)

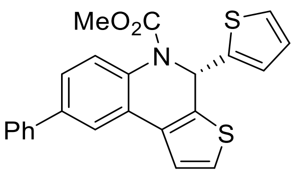
MeO2C
N
S
S
Ph

4z

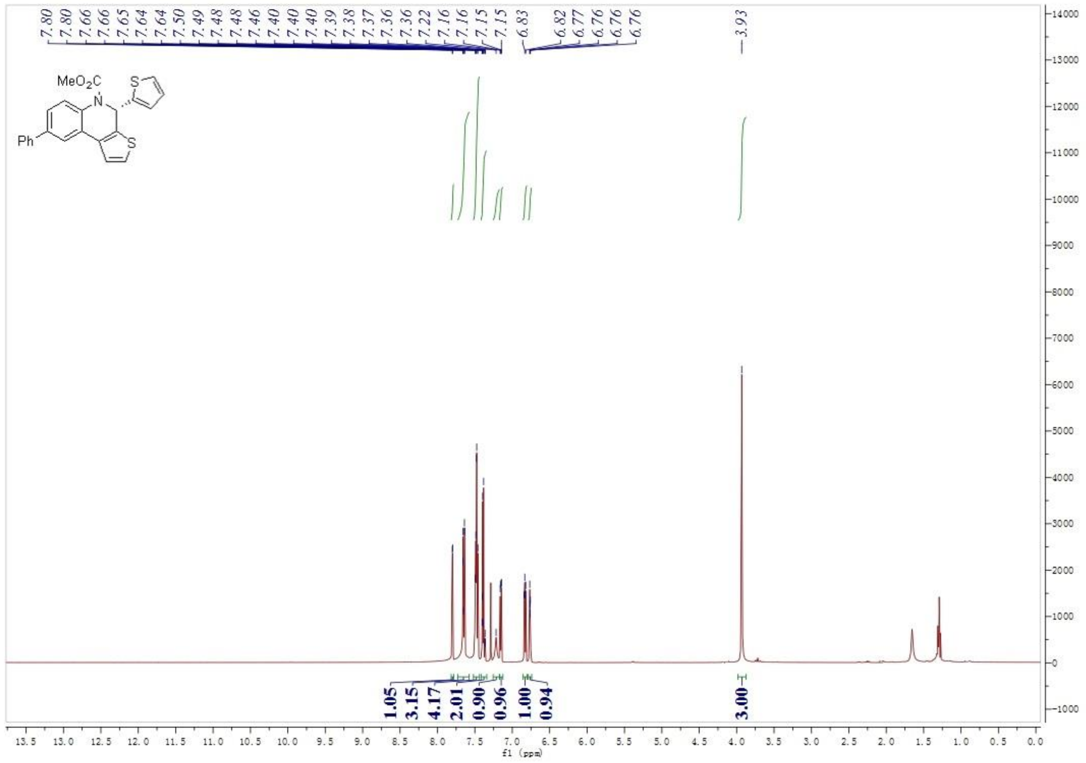

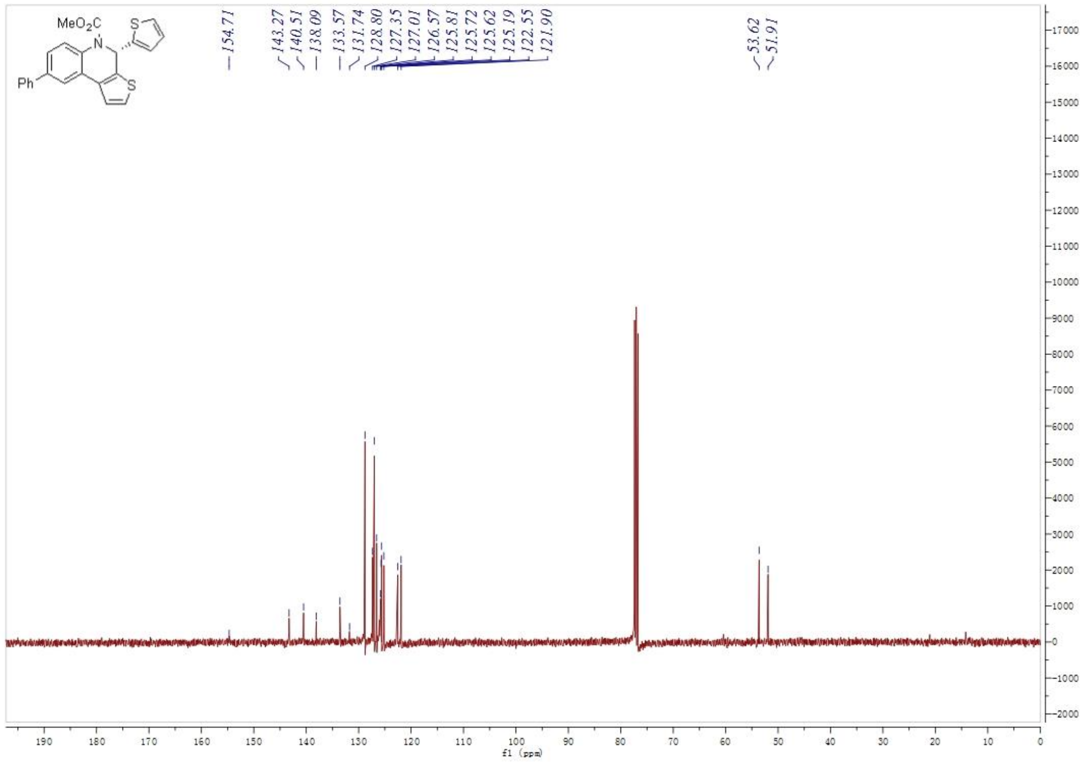

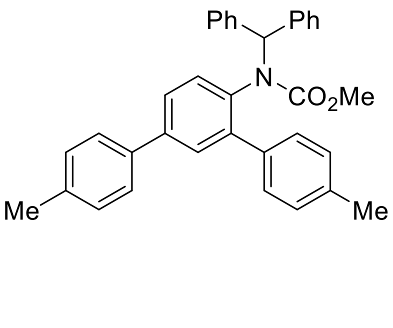

4bb

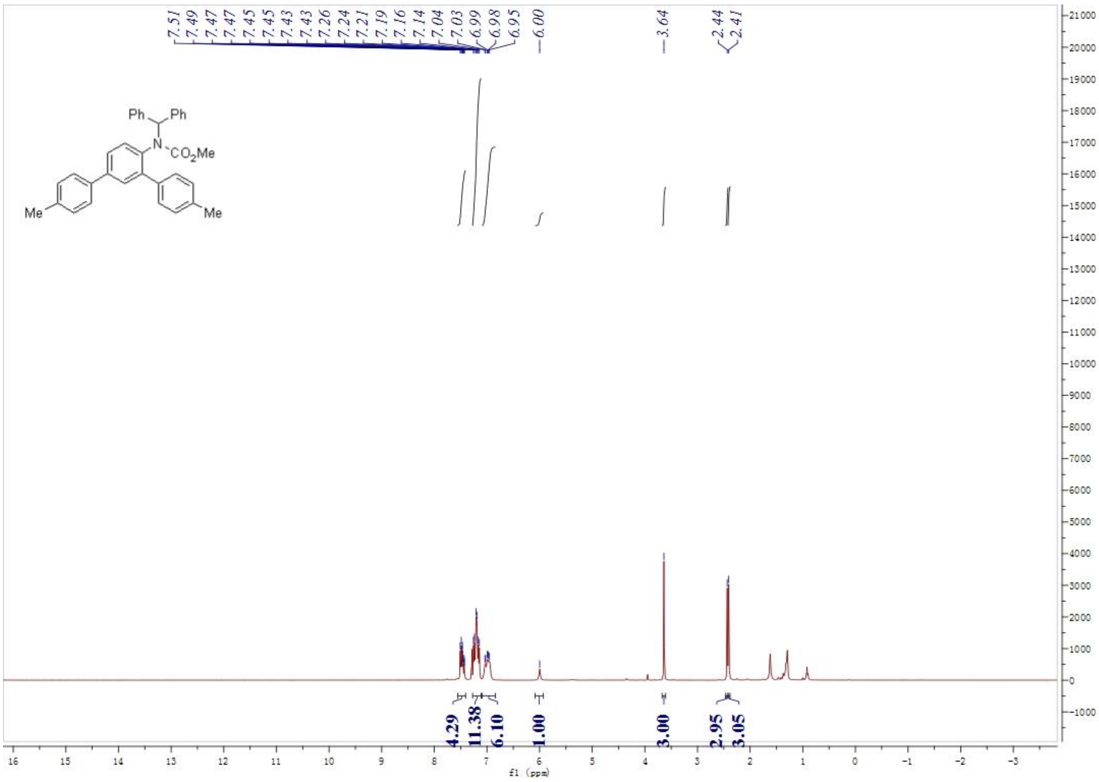
7.51 7.49 7.47 7.47 7.45 7.45 7.43 7.43 7.26 7.24 7.21 7.19 7.16 7.14 7.04 7.03 6.99 6.98 6.95
6.00
3.64
2.44 2.41
Ph Ph N CO2Me Me Me
4.29 11.38 6.10 1.00 3.00 2.95 3.05
f1 (ppm)

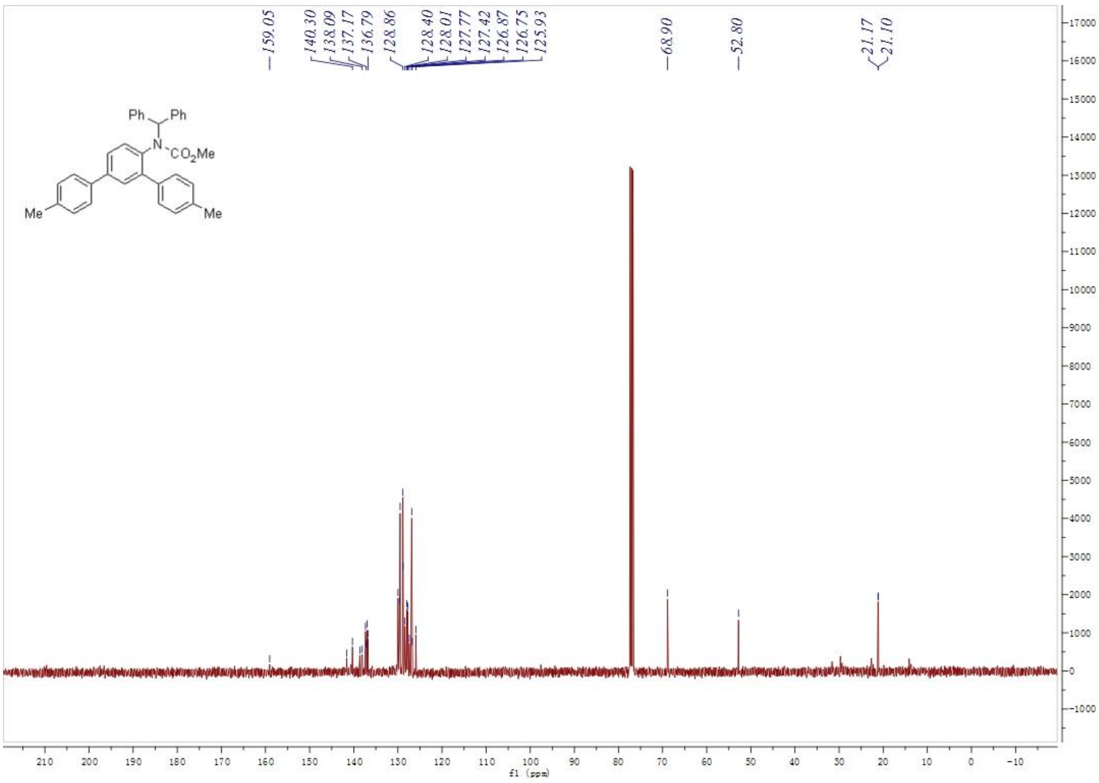
159.05
140.30 138.09 137.17 136.79 128.86 128.40 128.01 127.77 127.42 126.87 126.75 125.93
68.90
52.80
21.17 21.10
Ph Ph N CO2Me Me Me
f1 (ppm)

4b/4bb

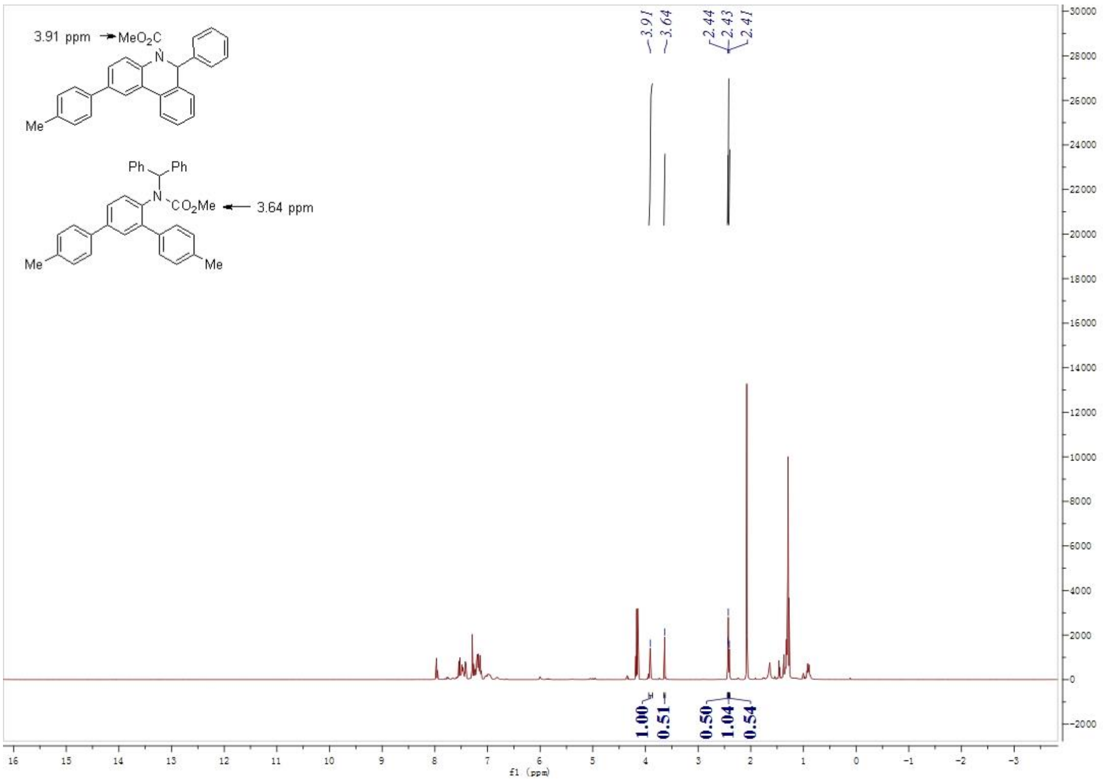
3.91 ppm
MeO2C
N
Me
Ph
Ph
N
CO2Me
3.64 ppm
Me
Me
3.91
3.64
2.44
2.43
2.41
1.00
0.51
0.50
1.04
0.54
f1 (ppm)

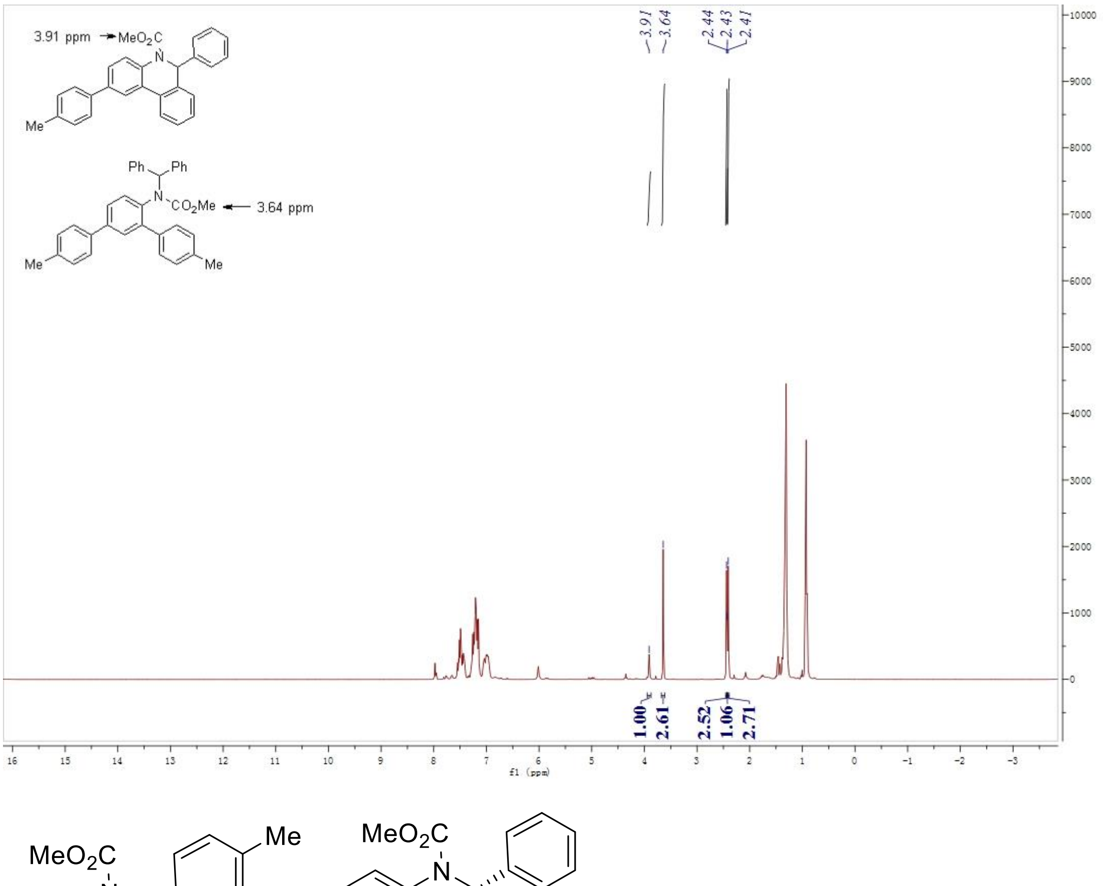
3.91 ppm
MeO2C
N
Me
Ph
Ph
N
CO2Me
3.64 ppm
Me
Me
3.91
3.64
2.44
2.43
2.41
1.00
2.61
2.52
1.06
2.71
f1 (ppm)

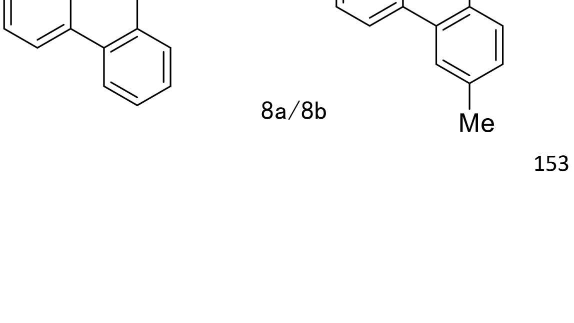
MeO2C
N
Me
MeO2C
N
Me

8a/8b

8a/8b

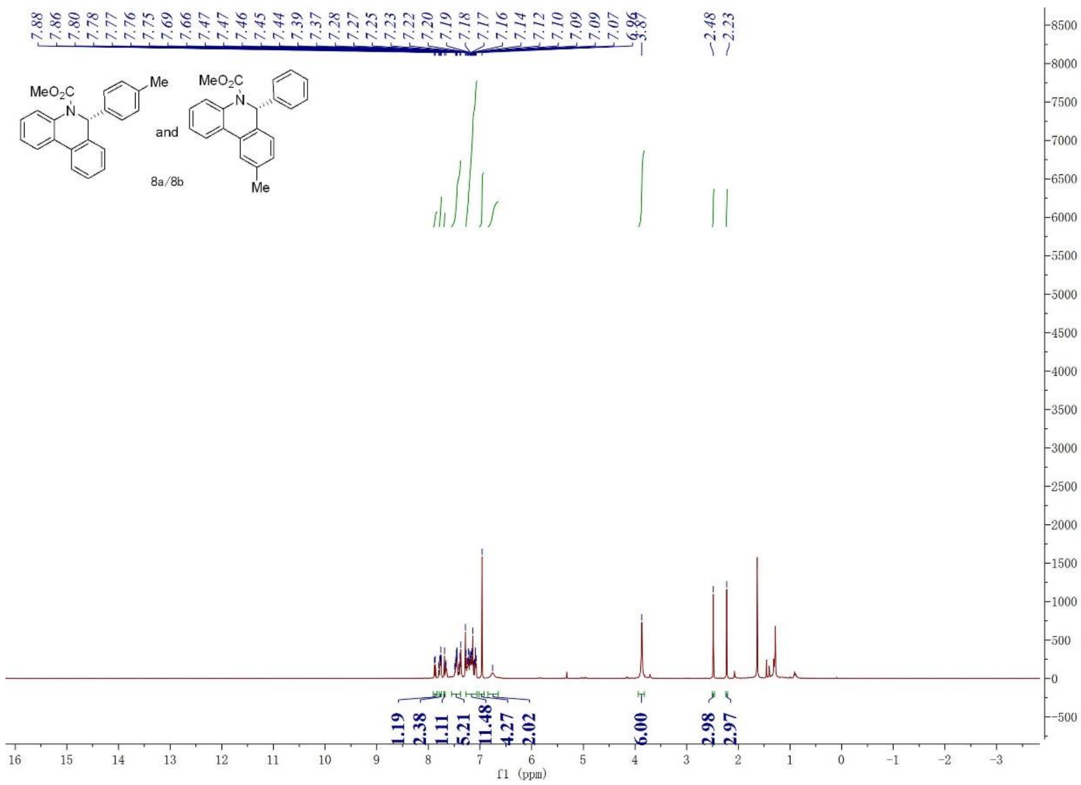

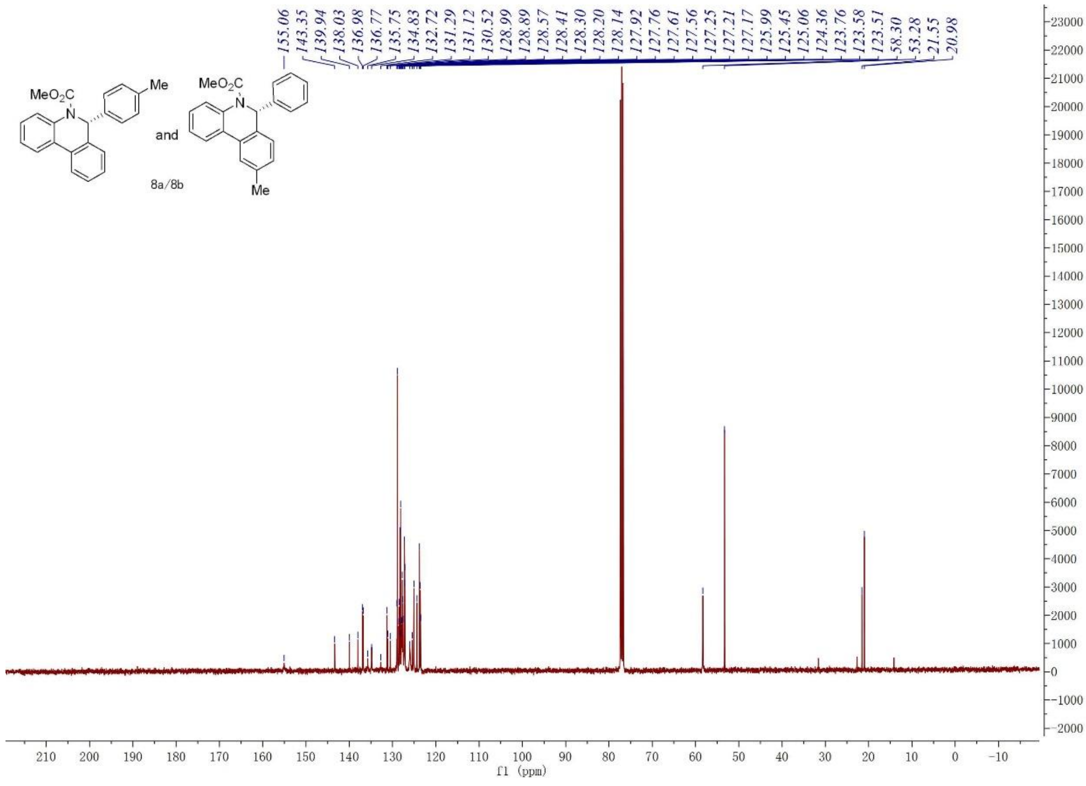

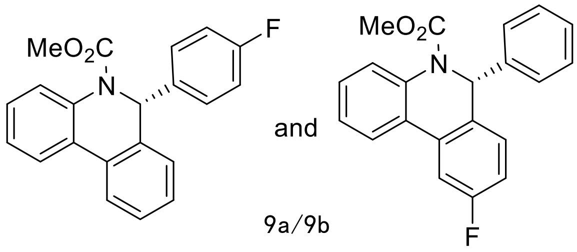

9a/9b

9a/9b

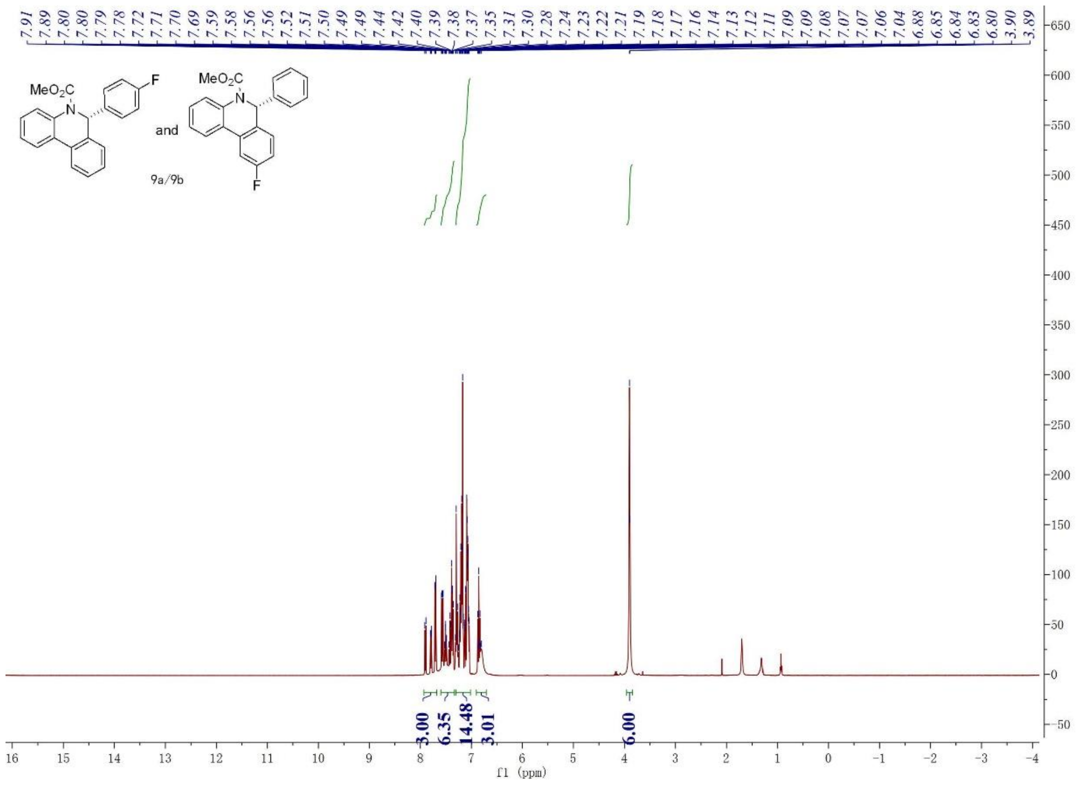

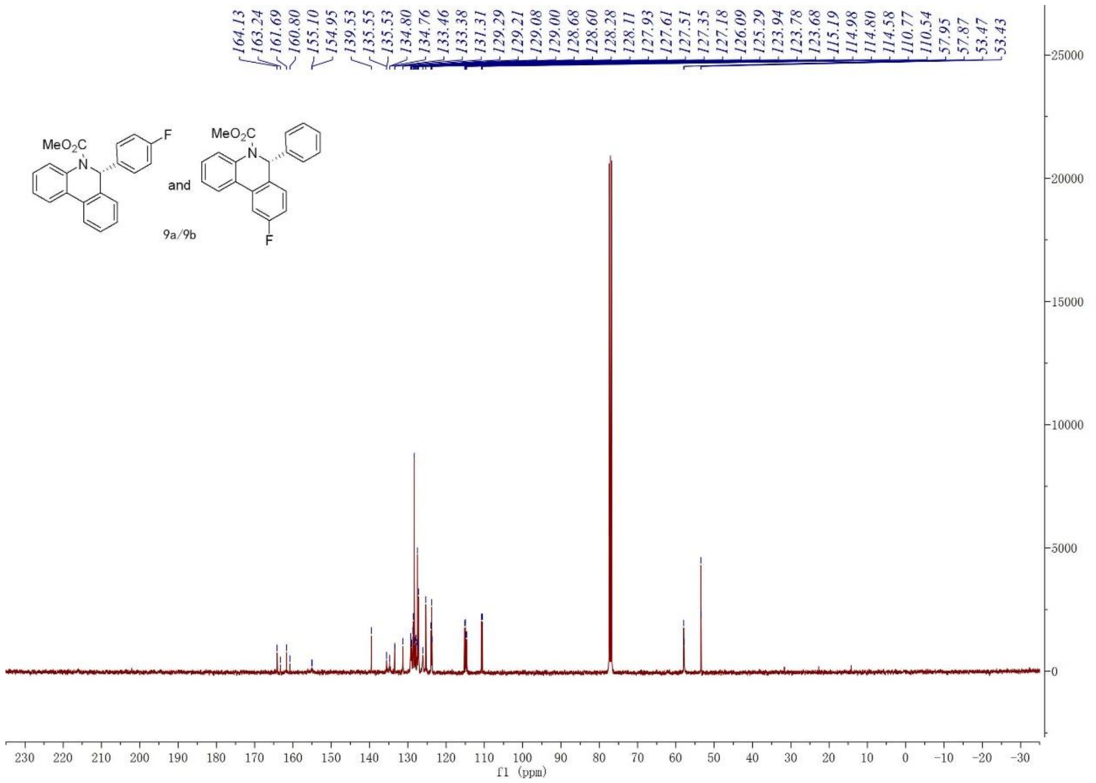
164.13
163.24
161.69
160.80
155.10
154.95
139.53
135.55
135.53
134.80
134.76
133.46
133.38
131.31
129.29
129.21
129.08
129.00
128.68
128.60
128.28
128.11
127.93
127.61
127.51
127.35
127.18
126.09
125.29
123.94
123.78
123.68
115.19
114.98
114.80
114.58
110.77
110.54
57.95
57.87
53.47
53.43
MeO2C
N
F
and
9a/9b
f1 (ppm)

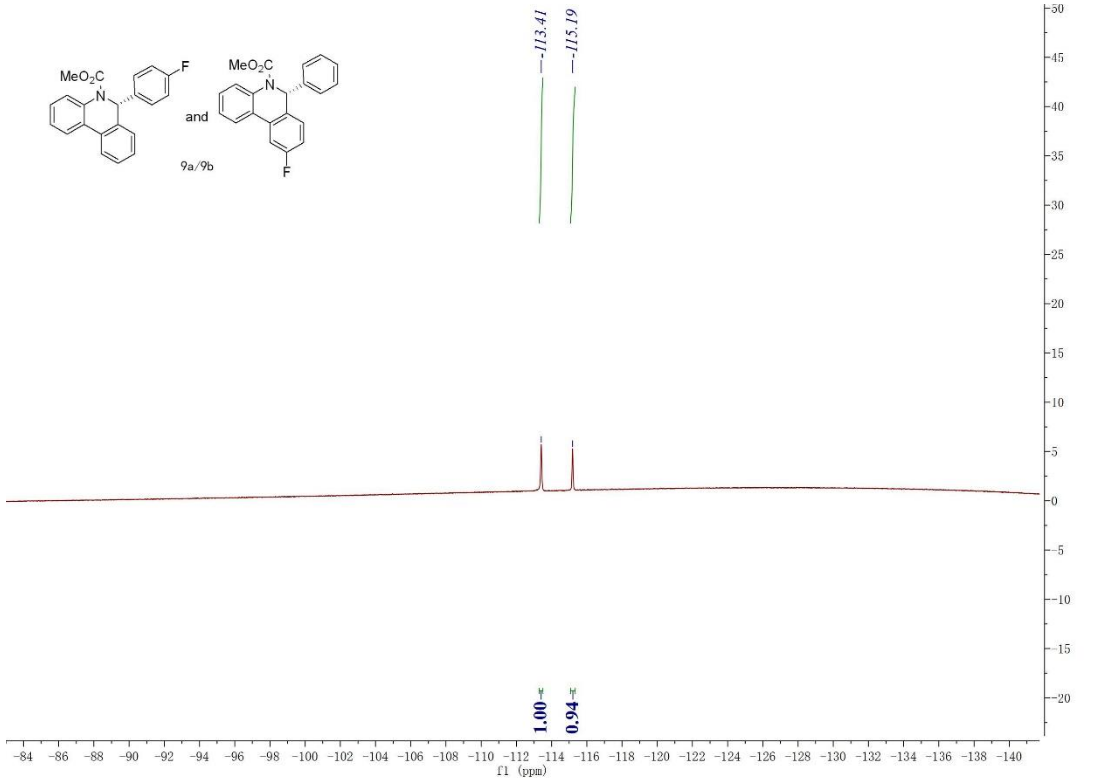
-113.41
-115.19
MeO2C
N
F
and
9a/9b
1.00
0.94
f1 (ppm)

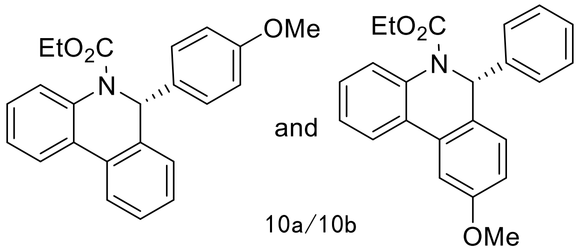
EtO2C
N
OMe
and
10a/10b
EtO2C
N
OMe

10a/10b

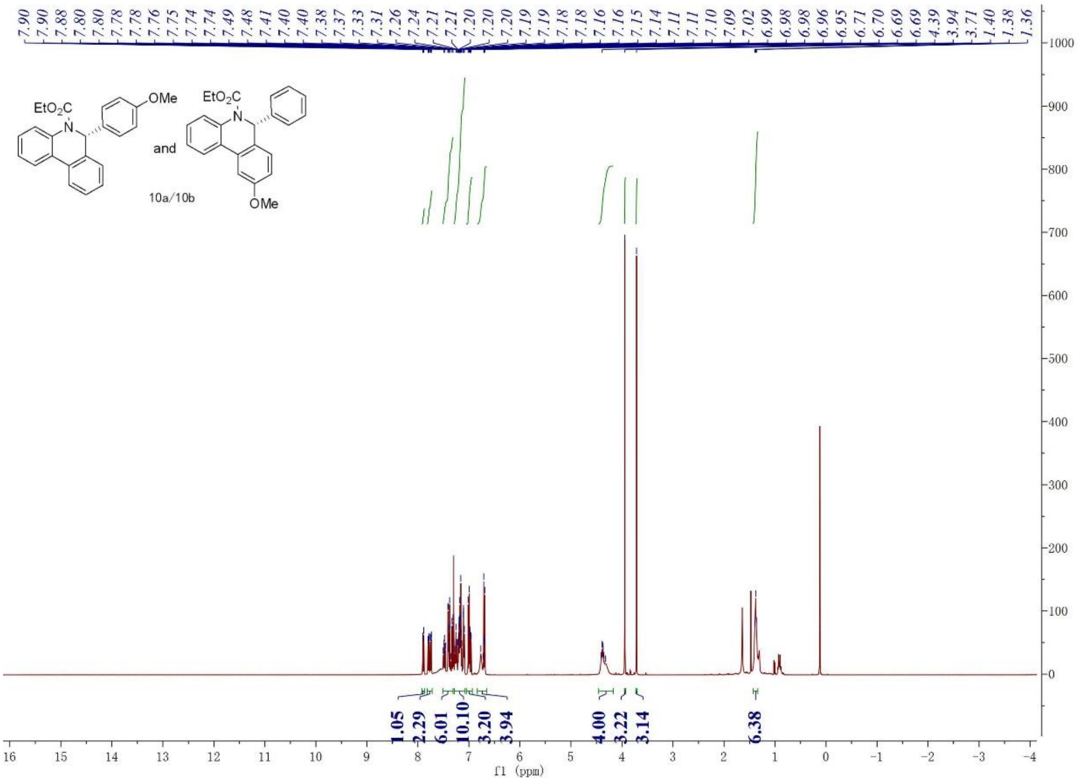
7.90 7.90 7.88 7.80 7.80 7.78 7.78 7.76 7.75 7.74 7.74 7.49 7.48 7.41 7.40 7.40 7.38 7.37 7.33 7.31 7.26 7.24 7.21 7.21 7.21 7.20 7.20 7.20 7.19 7.19 7.18 7.18 7.16 7.16 7.15 7.14 7.11 7.11 7.10 7.09 7.02 6.99 6.98 6.98 6.96 6.95 6.71 6.70 6.69 6.69 4.39 3.94 3.71 1.40 1.38 1.36
EtO2C
N
OMe
and
10a/10b
1.05
2.29
6.01
10.10
3.20
3.94
4.00
3.22
3.14
6.38
f1 (ppm)

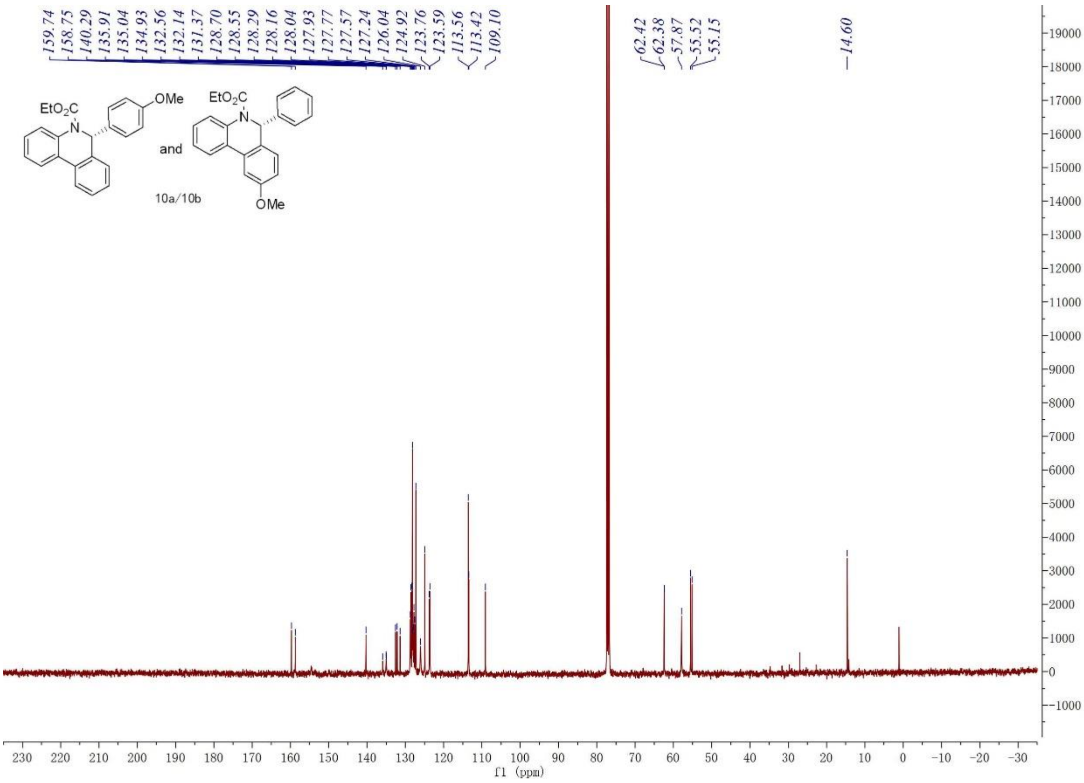

HPLC Data

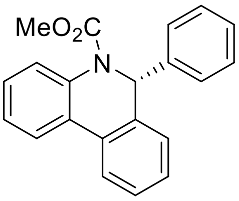

2a (The top one is racemic, the middle one is chiral when leaving group was Br and the bottom one is chiral when leaving group was I)

The enantiomeric excess was determined by HPLC analysis using a chiral stationary phase column [Daicel chiracel® IA-3, 243 nm, n-hexane : i-PrOH = 95 : 5 as the eluent, flow rate: 1 mL/min, temperature 25 °C, retention time: 11.3 min (major isomer)].

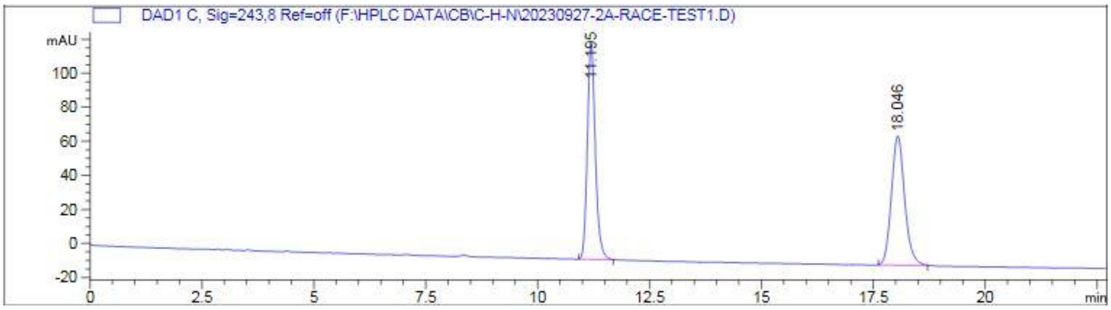

Peak #	RetTime [min]	Type	Width [min]	Area [mAU*s]	Height [mAU]	Area %
1	11.195	BB	0.1907	1575.02148	126.94826	50.0441
2	18.046	BB	0.3173	1572.24512	76.02339	49.9559

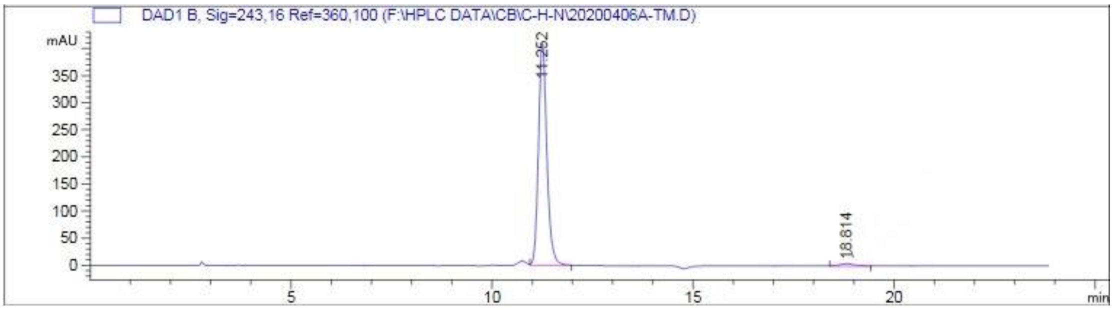

Peak #	RetTime [min]	Type	Width [min]	Area [mAU*s]	Height [mAU]	Area %
1	11.252	VB	0.2218	5967.63867	409.97314	98.0312
2	18.814	MM	0.4700	119.85156	4.25020	1.9688

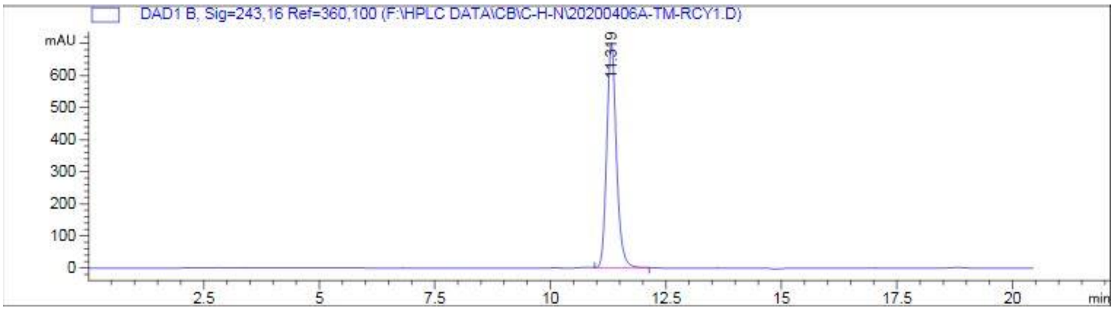

Peak #	RetTime [min]	Type	Width [min]	Area [mAU*s]	Height [mAU]	Area %
1	11.319	VB	0.2225	1.02458e4	700.99786	100.0000

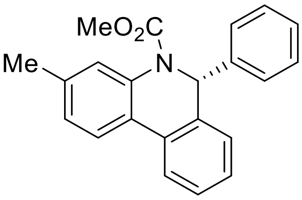

2b (The top one is racemic, and the following part is chiral)

The enantiomeric excess was determined by HPLC analysis using a chiral stationary phase column [Daicel chiracel® IA-3, 245 nm, n-hexane: i-PrOH = 95 : 5 as the eluent, flow rate: 1 mL/min, temperature 25 °C, retention time: 10.8 min (major isomer) and 16.0 min (minor isomer)].

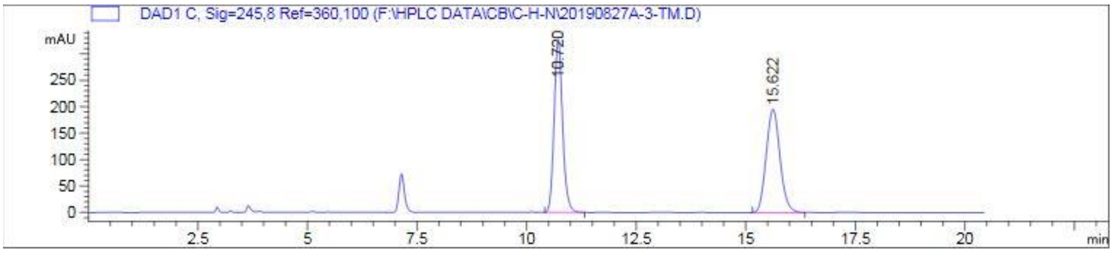

Peak #	RetTime [min]	Type	Width [min]	Area [mAU*s]	Height [mAU]	Area %
1	10.720	BB	0.2038	4372.48926	327.44354	50.4599
2	15.622	BB	0.3422	4292.78027	195.44794	49.5401

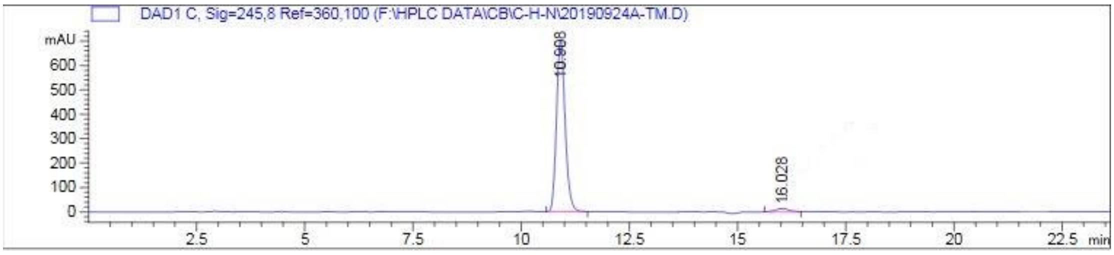

Peak #	RetTime [min]	Type	Width [min]	Area [mAU*s]	Height [mAU]	Area %
1	10.908	BB	0.2141	9801.70605	705.57111	96.9131
2	16.028	MM	0.3770	312.20374	13.80152	3.0869

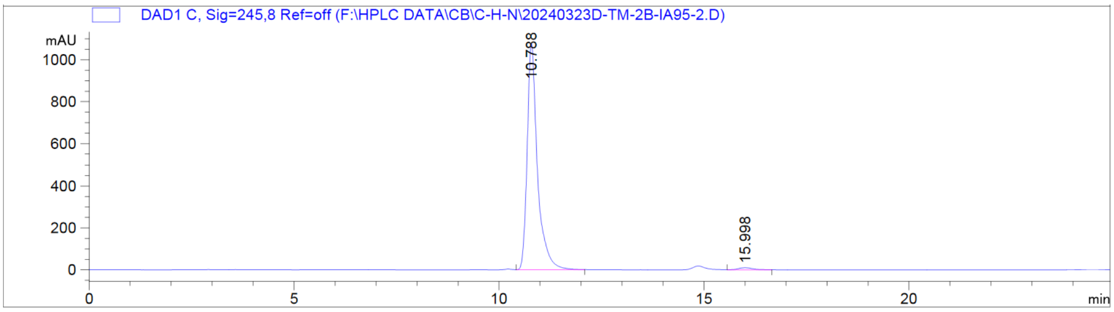

Peak #	RetTime [min]	Type	Width [min]	Area [mAU*s]	Height [mAU]	Area %
1	10.788	VB	0.2555	1.88326e4	1079.47949	98.6198
2	15.998	BB	0.3749	263.57321	10.35561	1.3802

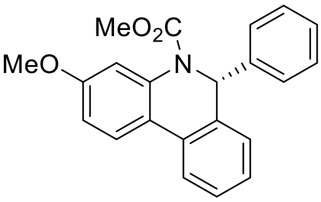

2c (The top one is racemic, and the bottom one is chiral)

The enantiomeric excess was determined by HPLC analysis using a chiral stationary phase column [Daicel chiracel® IC-3, 243 nm, n-hexane : i-PrOH = 80 : 20 as the eluent, flow rate: 1

mL/min, temperature 25 °C, retention time: 7.0 min (major isomer) and 9.8 min (minor isomer)].

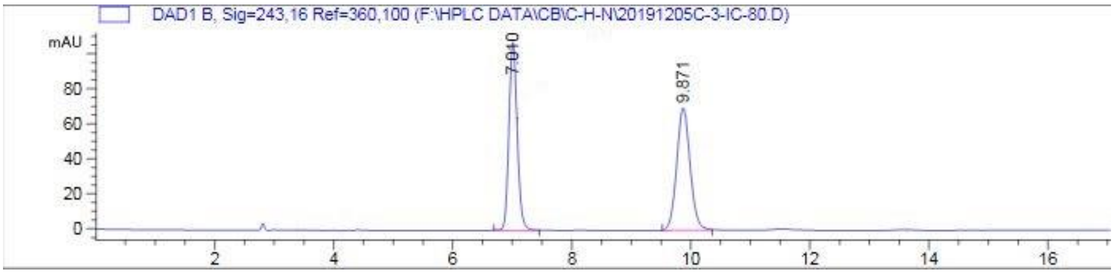

Peak #	RetTime [min]	Type	Width [min]	Area [mAU*s]	Height [mAU]	Area %
1	7.010	MM	0.1619	1047.41943	107.83707	48.8795
2	9.871	BB	0.2408	1095.44189	69.90499	51.1205

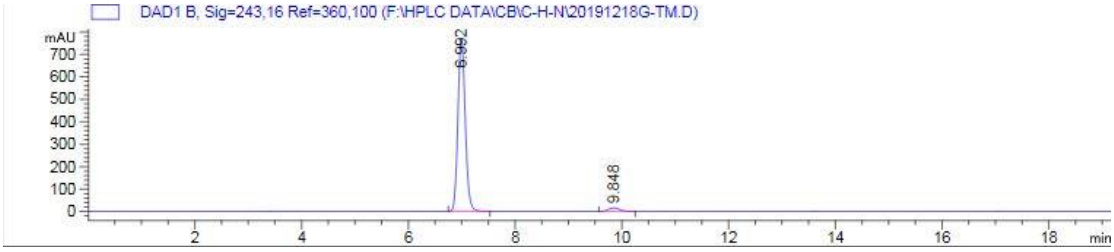

Peak #	RetTime [min]	Type	Width [min]	Area [mAU*s]	Height [mAU]	Area %
1	6.992	BB	0.1474	7388.97021	774.06525	96.8182
2	9.848	BB	0.2303	242.83206	16.25041	3.1818

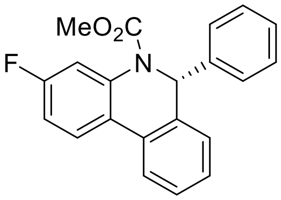

2d (The top one is racemic, and the bottom one is chiral)

The enantiomeric excess was determined by HPLC analysis using a chiral stationary phase column [Daicel chiracel® IA-3, 243 nm, n-hexane : i-PrOH = 95 : 5 as the eluent, flow rate: 1 mL/min, temperature 25 °C, retention time: 11.2 min (major isomer) and 16.2 min (minor isomer)].

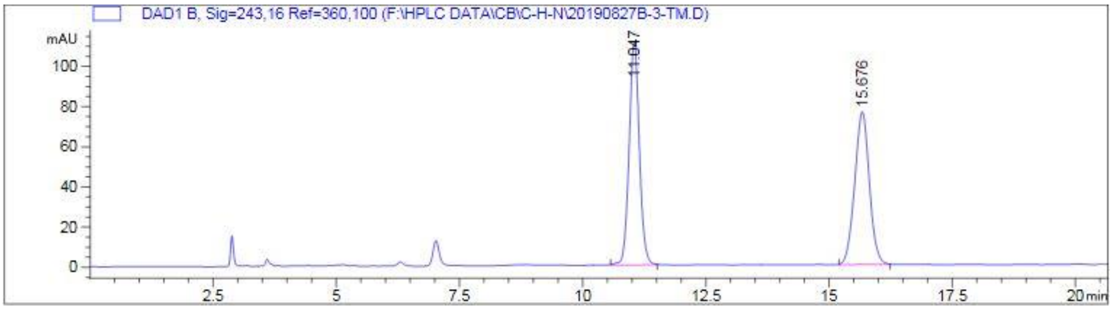

Peak #	RetTime [min]	Type	Width [min]	Area [mAU*s]	Height [mAU]	Area %
1	11.047	BB	0.2202	1602.54285	111.16516	50.4941
2	15.676	BB	0.3172	1571.17932	76.00934	49.5059

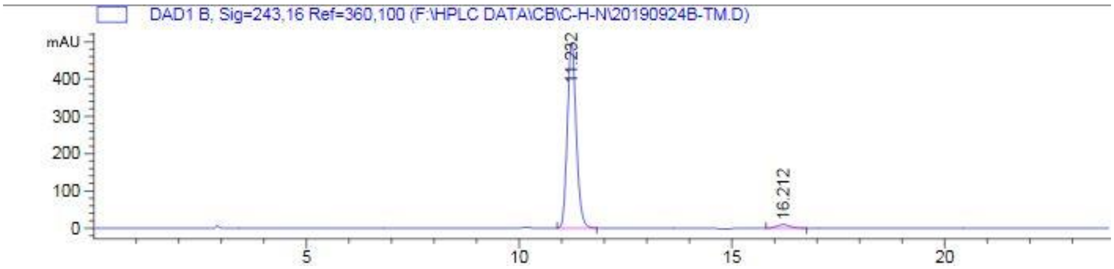

Peak #	RetTime [min]	Type	Width [min]	Area [mAU*s]	Height [mAU]	Area %
1	11.232	BB	0.2204	7192.92773	498.27744	97.2453
2	16.212	BB	0.3230	203.75499	9.62249	2.7547

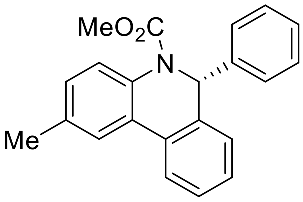

2e (The top one is racemic, and the following part is chiral)

The enantiomeric excess was determined by HPLC analysis using a chiral stationary phase column [Daicel chiracel® IA-3, 244 nm, n-hexane : i-PrOH = 95 : 5 as the eluent, flow rate: 1 mL/min, temperature 25 °C, retention time: 10.0 min (major isomer) and 16.6 min (minor isomer)].

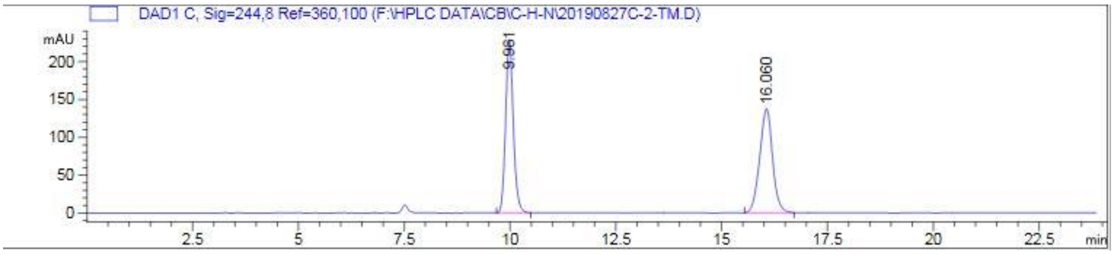

Peak #	RetTime [min]	Type	Width [min]	Area [mAU*s]	Height [mAU]	Area %
1	9.981	BB	0.1917	2898.24219	228.88185	49.9265
2	16.060	BB	0.3228	2906.77588	137.39240	50.0735

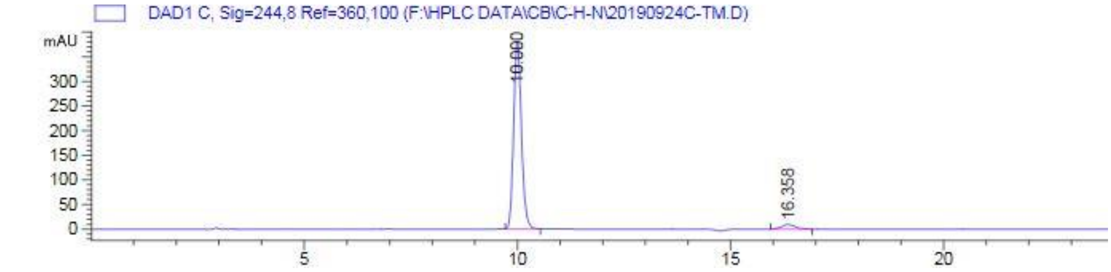

Peak #	RetTime [min]	Type	Width [min]	Area [mAU*s]	Height [mAU]	Area %
1	10.000	BB	0.1959	4832.34717	381.26477	96.0403
2	16.358	BB	0.3347	199.23438	9.19717	3.9597

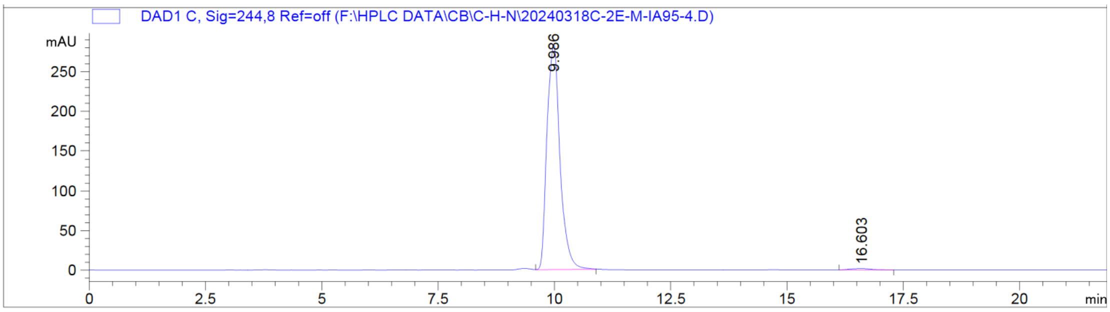

Peak #	RetTime [min]	Type	Width [min]	Area [mAU*s]	Height [mAU]	Area %
1	9.986	VB	0.3091	5519.04541	283.58719	98.9814
2	16.603	BB	0.3947	56.79646	1.80696	1.0186

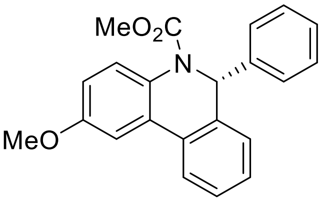

2f (The top one is racemic, and the bottom one is chiral)

The enantiomeric excess was determined by HPLC analysis using a chiral stationary phase column [Daicel chiracel® IA-3, 243 nm, n-hexane : i-PrOH = 95 : 5 as the eluent, flow rate: 1 mL/min, temperature 25 °C, retention time: 19.4 min (major isomer) and 24.3 min (minor isomer)].

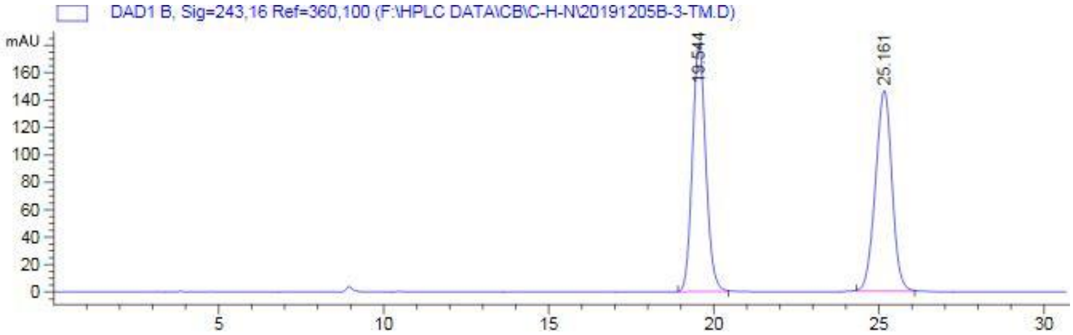

Peak #	RetTime [min]	Type	Width [min]	Area [mAU*s]	Height [mAU]	Area %
1	19.544	BB	0.4373	5097.04980	180.60698	50.0929
2	25.161	BB	0.5389	5078.14111	146.30795	49.9071

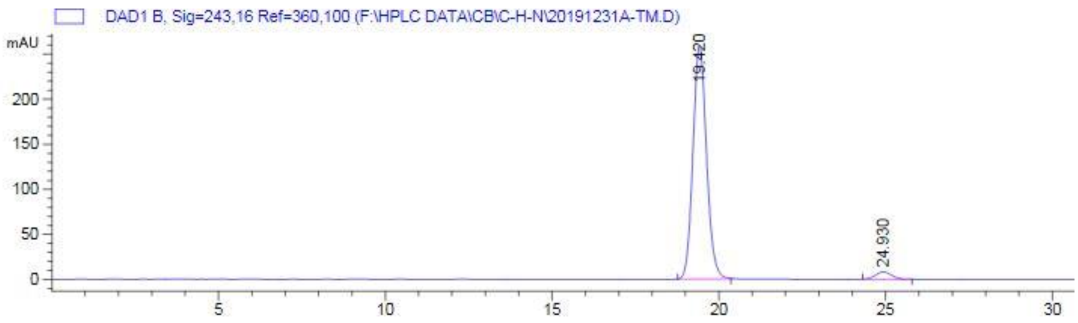

Peak #	RetTime [min]	Type	Width [min]	Area [mAU*s]	Height [mAU]	Area %
1	19.420	BB	0.4357	7326.65625	259.23502	96.3074
2	24.930	BB	0.5219	280.91412	8.19592	3.6926

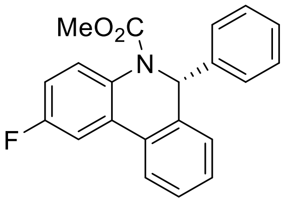

2g (The top one is racemic, and the following part is chiral)

The enantiomeric excess was determined by HPLC analysis using a chiral stationary phase column [Daicel chiracel® IA-3, 243 nm, n-hexane : i-PrOH = 95 : 5 as the eluent, flow rate: 1 mL/min, temperature 25 °C, retention time: 10.2 min (major isomer) and 19.2 min (minor isomer)].

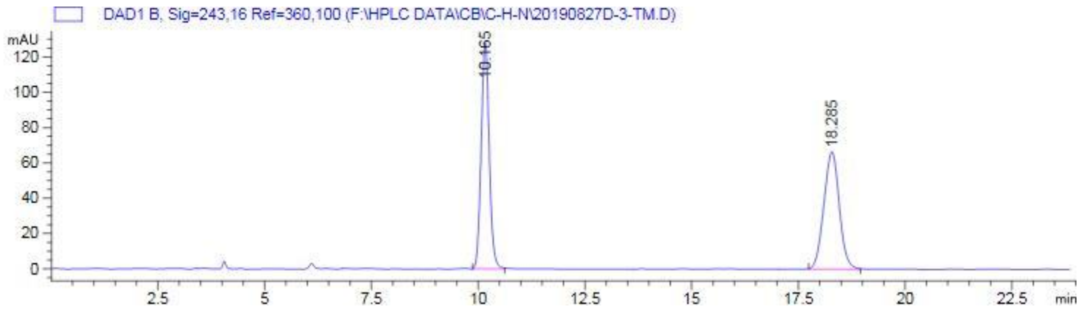

Peak #	RetTime [min]	Type	Width [min]	Area [mAU*s]	Height [mAU]	Area %
1	10.165	BB	0.2003	1672.72375	128.14343	50.0986
2	18.285	BB	0.3886	1666.14185	66.43344	49.9014

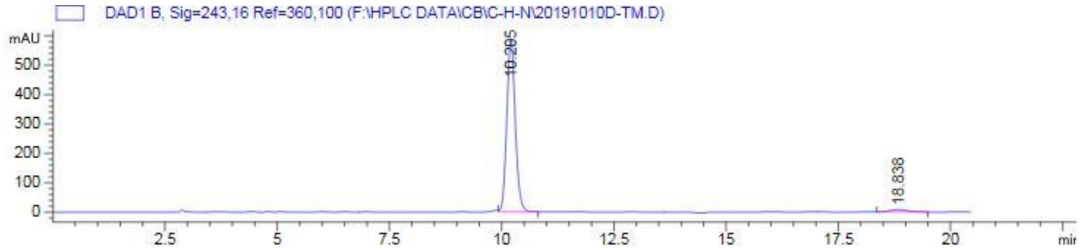

Peak #	RetTime [min]	Type	Width [min]	Area [mAU*s]	Height [mAU]	Area %
1	10.205	VB	0.2011	7738.75049	589.54285	97.2644
2	18.838	BB	0.3859	217.65569	8.69830	2.7356

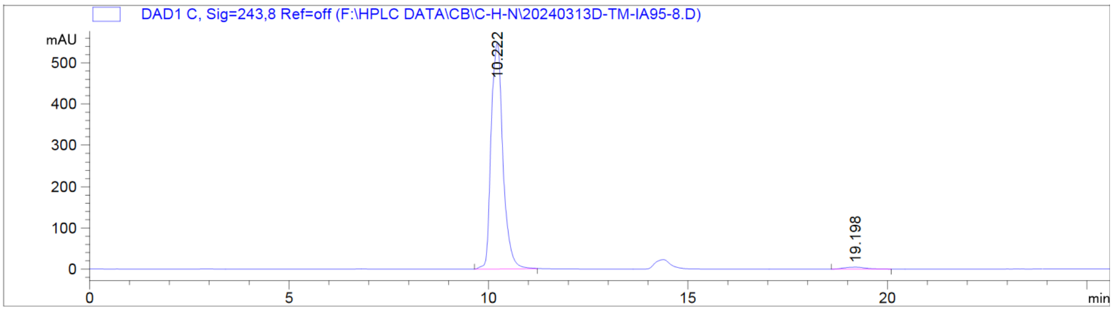

Peak #	RetTime [min]	Type	Width [min]	Area [mAU*s]	Height [mAU]	Area %
1	10.222	BB	0.3235	1.12741e4	549.15228	98.4008
2	19.198	BB	0.5289	183.22429	4.82632	1.5992

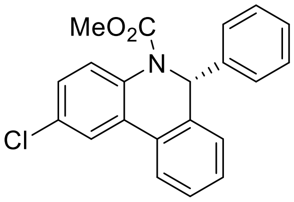

2h (The top one is racemic, and the bottom one is chiral)

The enantiomeric excess was determined by HPLC analysis using a chiral stationary phase column [Daicel chiracel® IA-3, 247 nm, n-hexane : i-PrOH = 95 : 5 as the eluent, flow rate: 1 mL/min, temperature 25 °C, retention time: 9.7 min (major isomer) and 13.8 min (minor isomer)].

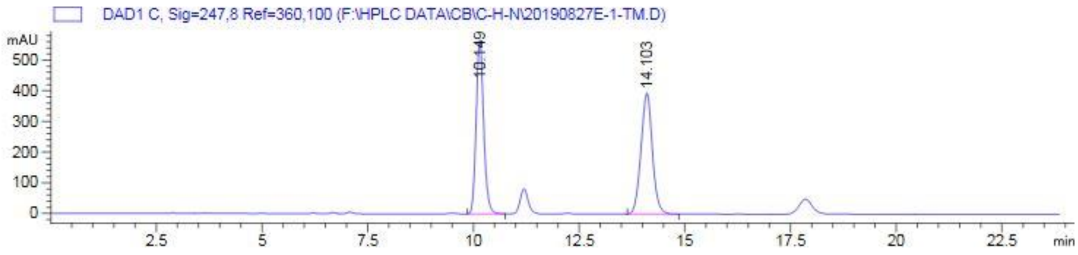

Peak #	RetTime [min]	Type	Width [min]	Area [mAU*s]	Height [mAU]	Area %
1	10.149	BB	0.1882	7011.26074	567.08734	48.6124
2	14.103	BB	0.2892	7411.52344	394.42850	51.3876

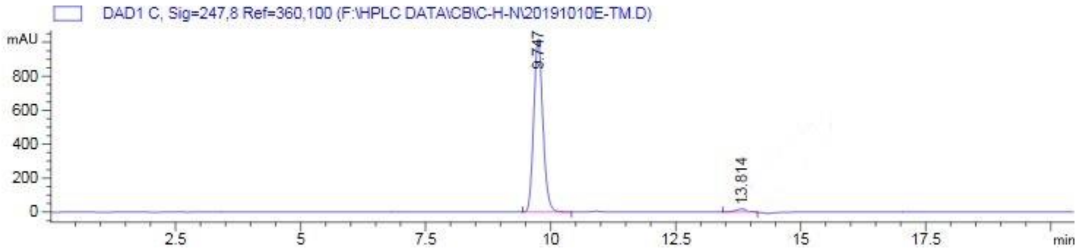

Peak #	RetTime [min]	Type	Width [min]	Area [mAU*s]	Height [mAU]	Area %
1	9.747	BB	0.1968	1.29853e4	1017.97900	97.7383
2	13.814	MM	0.2763	300.48279	18.12452	2.2617

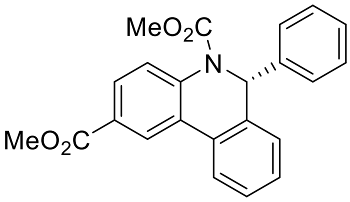

2i (The top one is racemic, and the following part is chiral)

The enantiomeric excess was determined by HPLC analysis using a chiral stationary phase column [Daicel chiracel® IA-3, 243 nm, n-hexane : i-PrOH = 95 : 5 as the eluent, flow rate: 1 mL/min, temperature 25 °C, retention time: 17.5 min (major isomer) and 19.3 min (minor isomer)].

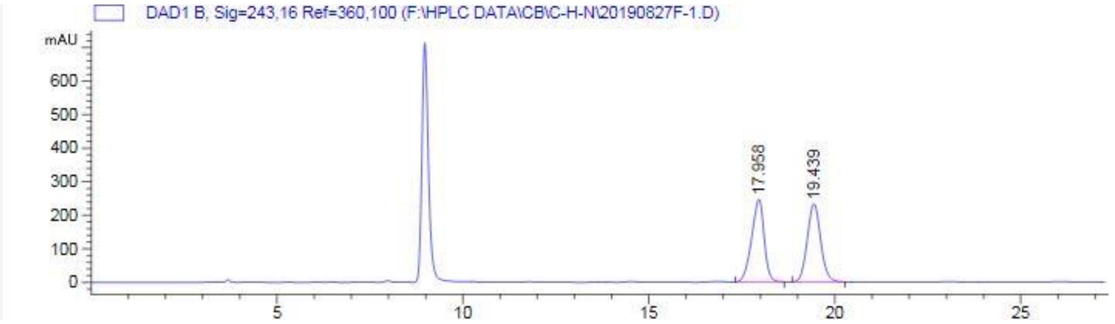

Peak #	RetTime [min]	Type	Width [min]	Area [mAU*s]	Height [mAU]	Area %
1	17.958	BB	0.3609	5806.51367	246.45331	50.0704
2	19.439	BB	0.3823	5790.17725	232.70830	49.9296

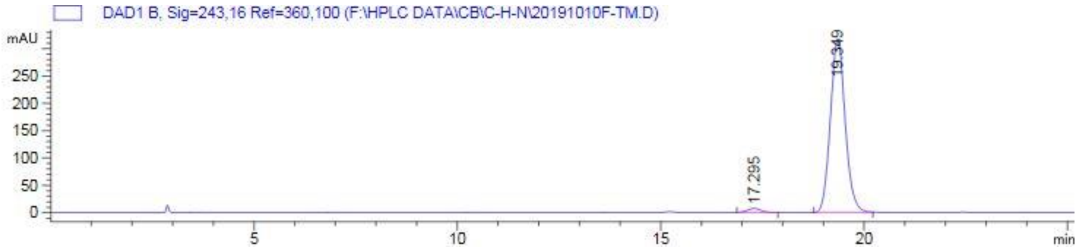

Peak #	RetTime [min]	Type	Width [min]	Area [mAU*s]	Height [mAU]	Area %
1	17.295	BB	0.3530	171.31705	7.48461	2.0898
2	19.349	BB	0.3865	8026.54980	318.00082	97.9102

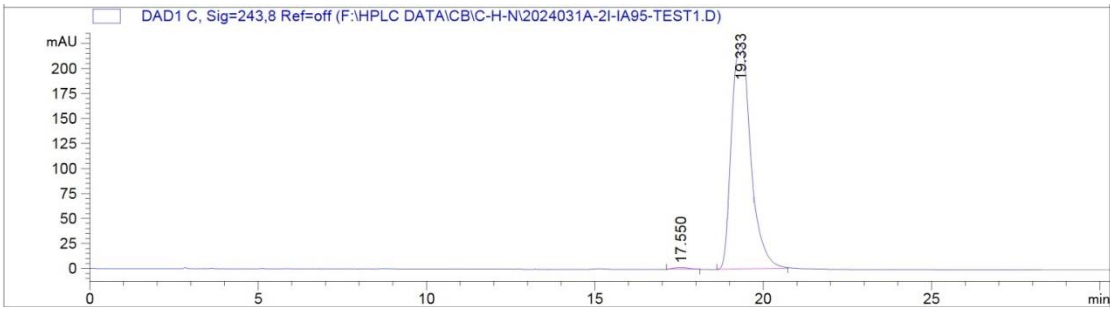

Peak #	RetTime [min]	Type	Width [min]	Area [mAU*s]	Height [mAU]	Area %
1	17.550	MM	0.4979	45.06358	1.50833	0.4991
2	19.333	BB	0.6326	8983.23633	224.59407	99.5009

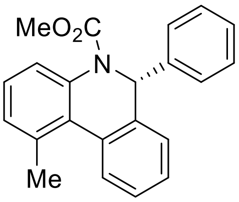

2j (The top one is racemic, and the bottom one is chiral)

The enantiomeric excess was determined by HPLC analysis using a chiral stationary phase column [Daicel chiracel® IA-3, 244 nm, n-hexane : i-PrOH = 95 : 5 as the eluent, flow rate: 1 mL/min, temperature 25 °C, retention time: 10.0 min (major isomer) and 11.6 min (minor isomer)].

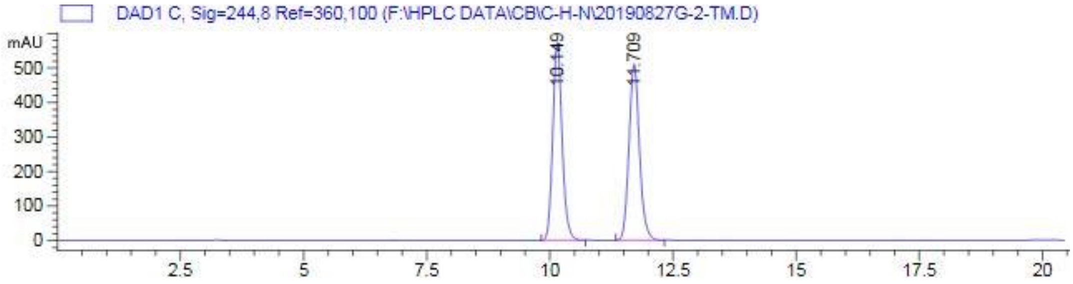

Peak #	RetTime [min]	Type	Width [min]	Area [mAU*s]	Height [mAU]	Area %
1	10.149	BB	0.2001	7572.09082	573.24664	49.9564
2	11.709	BB	0.2272	7585.29932	510.81381	50.0436

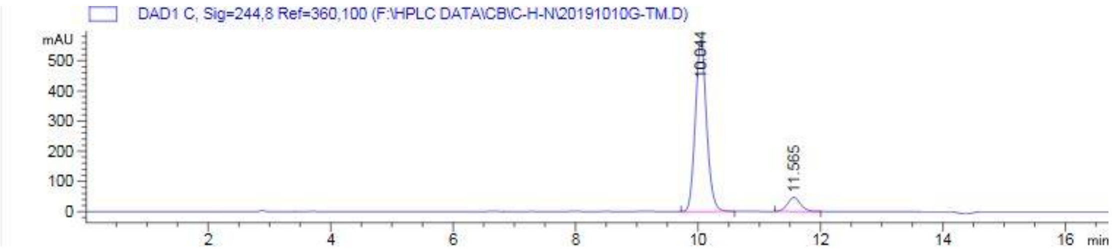

Peak #	RetTime [min]	Type	Width [min]	Area [mAU*s]	Height [mAU]	Area %
1	10.044	BB	0.1975	7295.91748	569.36847	91.2735
2	11.565	BB	0.2303	697.55035	46.68714	8.7265

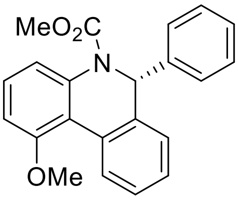

2k (The top one is racemic, and the bottom one is chiral)

The enantiomeric excess was determined by HPLC analysis using a chiral stationary phase column [Daicel chiracel® IA-3, 243 nm, n-hexane : i-PrOH = 98 : 2 as the eluent, flow rate: 1 mL/min, temperature 25 °C, retention time: 22.1 min (major isomer) and 25.5 min (minor isomer)].

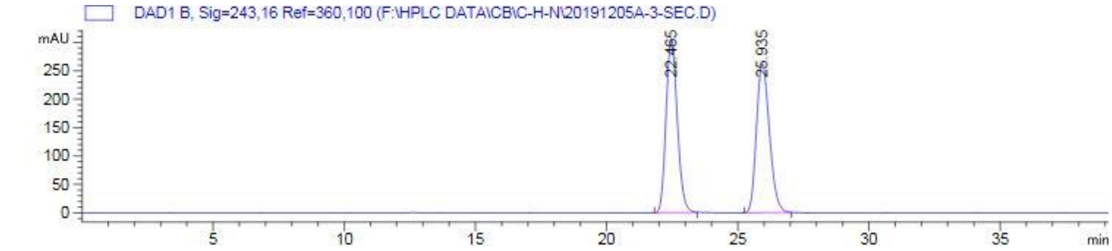

Peak #	RetTime [min]	Type	Width [min]	Area [mAU*s]	Height [mAU]	Area %
1	22.465	BB	0.4632	9117.47266	306.36108	50.1849
2	25.935	BB	0.5385	9050.28516	262.34274	49.8151

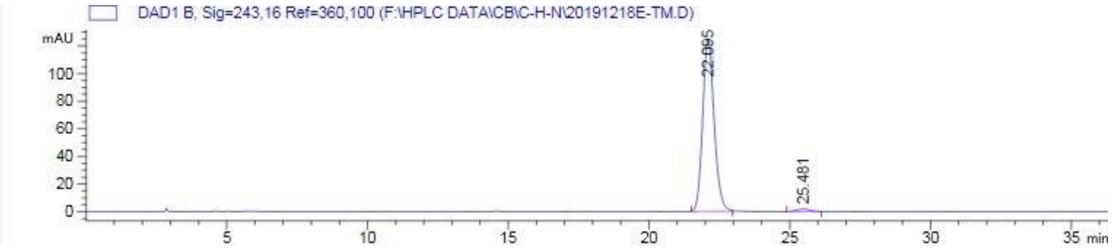

Peak #	RetTime [min]	Type	Width [min]	Area [mAU*s]	Height [mAU]	Area %
1	22.095	BB	0.4325	3525.28711	125.18686	98.2321
2	25.481	MM	0.5542	63.44700	1.90797	1.7679

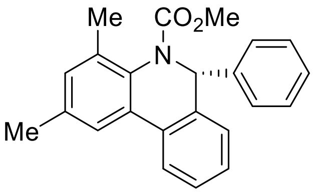

2l (The top one is racemic, and the following part is chiral)

The enantiomeric excess was determined by HPLC analysis using a chiral stationary phase column [Daicel chiracel® IA-3, 271 nm, n-hexane : i-PrOH = 95 : 5 as the eluent, flow rate: 1 mL/min, temperature 25 °C, retention time: 9.5 min (major isomer) and 12.3 min (minor isomer)].

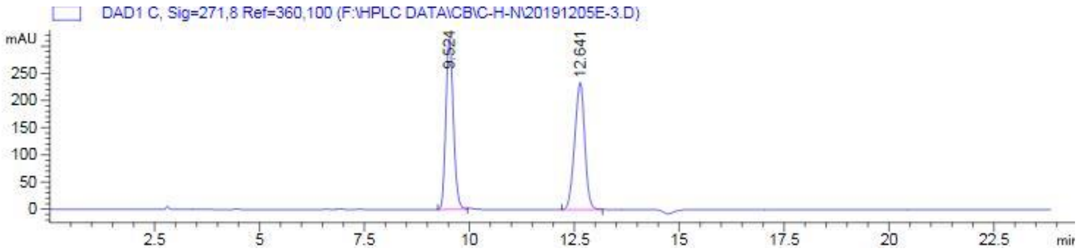

Peak #	RetTime [min]	Type	Width [min]	Area [mAU*s]	Height [mAU]	Area %
1	9.524	BB	0.1958	3920.30249	313.67917	49.9729
2	12.641	BB	0.2595	3924.55884	234.19012	50.0271

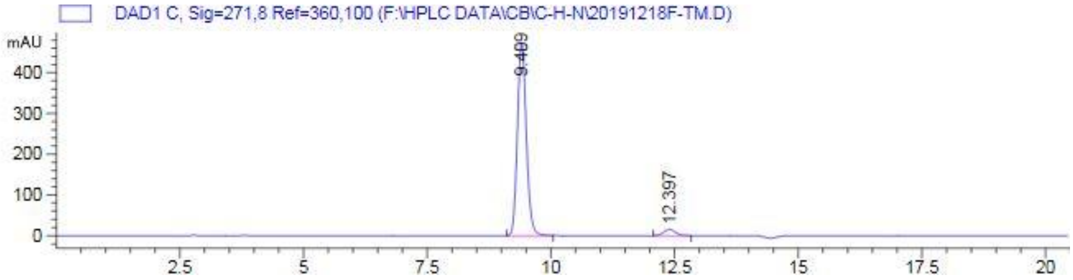

Peak #	RetTime [min]	Type	Width [min]	Area [mAU*s]	Height [mAU]	Area %
1	9.409	BB	0.1921	5940.78809	474.49472	95.8352
2	12.397	BB	0.2574	258.17719	15.57583	4.1648

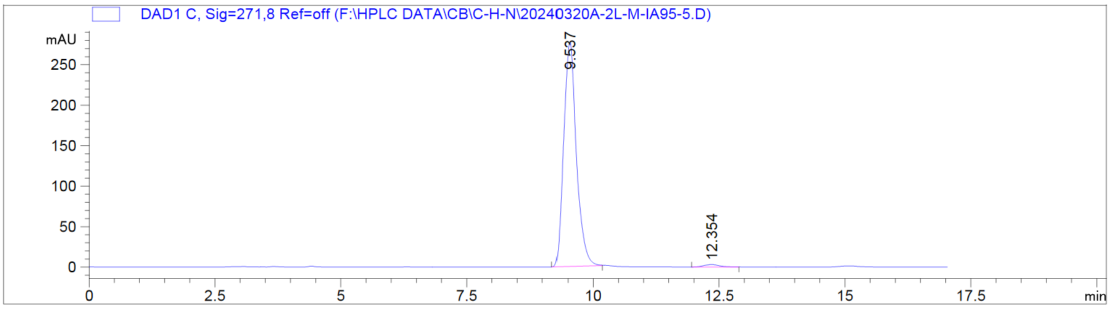

Peak #	RetTime [min]	Type	Width [min]	Area [mAU*s]	Height [mAU]	Area %
1	9.537	BB	0.2726	4809.47607	276.93069	98.6095
2	12.354	BB	0.3186	67.82098	3.08335	1.3905

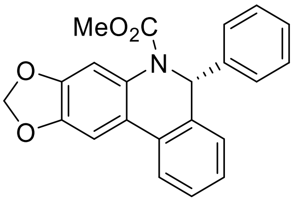

2m (The top one is racemic, and the bottom one is chiral)

The enantiomeric excess was determined by HPLC analysis using a chiral stationary phase column [Daicel chiracel® IA-3, 254 nm, n-hexane : i-PrOH = 80 : 20 as the eluent, flow rate: 1 mL/min, temperature 25 °C, retention time: 9.1 min (major isomer) and 13.8 min (minor isomer)].

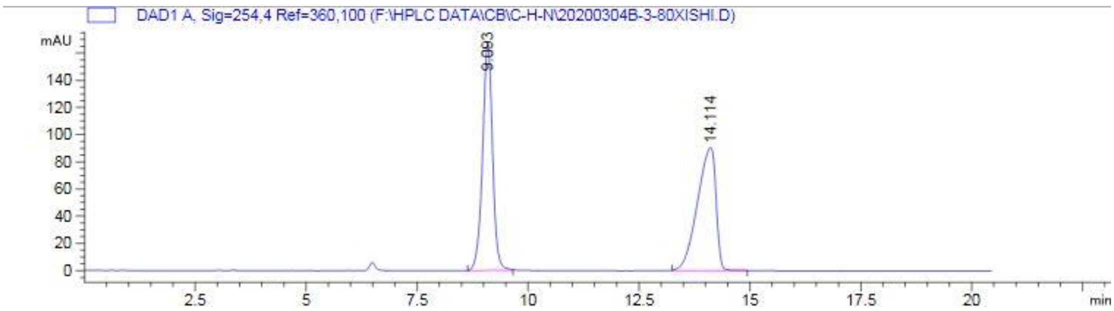

Peak #	RetTime [min]	Type	Width [min]	Area [mAU*s]	Height [mAU]	Area %
1	9.093	BB	0.2383	2610.35425	167.03287	49.7919
2	14.114	BB	0.4632	2632.17749	90.53261	50.2081

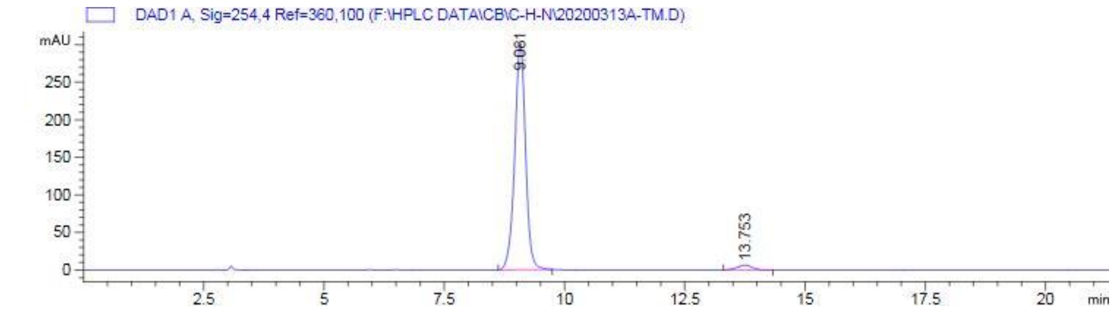

Peak #	RetTime [min]	Type	Width [min]	Area [mAU*s]	Height [mAU]	Area %
1	9.081	BB	0.2369	4730.40967	301.71643	97.0139
2	13.753	BB	0.3487	145.60370	6.46627	2.9861

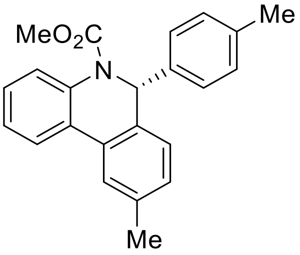

2n (The top one is racemic, and the following part is chiral)

The enantiomeric excess was determined by HPLC analysis using a chiral stationary phase column [Daicel chiracel® IA-3, 271 nm, n-hexane : i-PrOH = 97 : 3 as the eluent, flow rate: 1 mL/min, temperature 25 °C, retention time: 14.4 min (major isomer) and 17.1 min (minor isomer)].

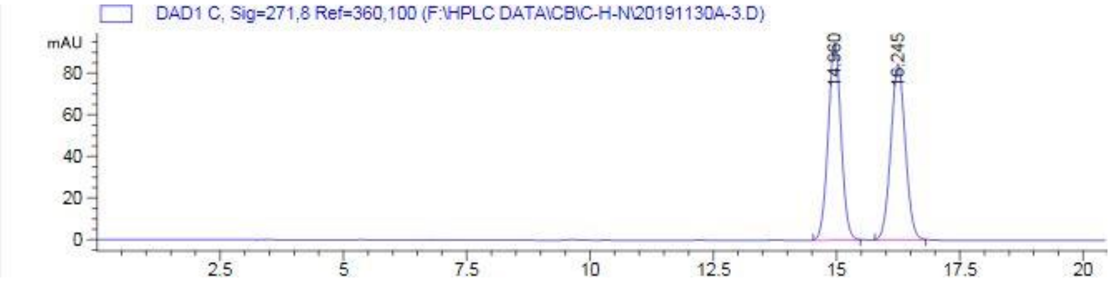

Peak #	RetTime [min]	Type	Width [min]	Area [mAU*s]	Height [mAU]	Area %
1	14.960	BB	0.2811	1733.59045	94.91926	49.9499
2	16.245	BB	0.3151	1737.07031	84.74850	50.0501

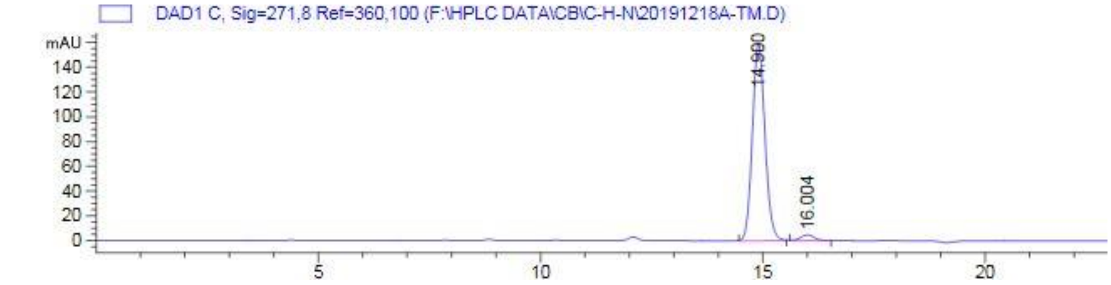

Peak #	RetTime [min]	Type	Width [min]	Area [mAU*s]	Height [mAU]	Area %
1	14.900	BB	0.3030	3134.01855	159.69118	96.9124
2	16.004	BB	0.3318	99.84898	4.62648	3.0876

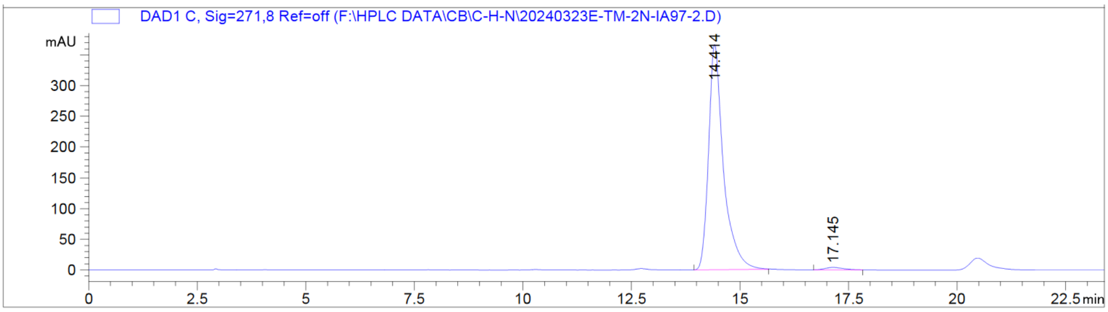

Peak #	RetTime [min]	Type	Width [min]	Area [mAU*s]	Height [mAU]	Area %
1	14.414	BB	0.3491	8776.15625	366.85803	98.6540
2	17.145	BB	0.4320	119.74224	4.06138	1.3460

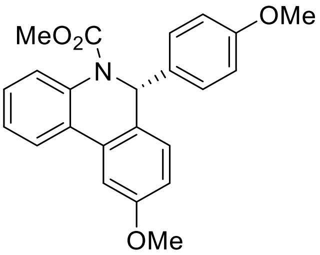

2o (The top one is racemic, and the following part is chiral)

The enantiomeric excess was determined by HPLC analysis using a chiral stationary phase column [Daicel chiracel® IA-3, 272 nm, n-hexane : i-PrOH = 95 : 5 as the eluent, flow rate: 1 mL/min, temperature 25 °C, retention time: 24.4 min (major isomer) and 32.6 min (minor isomer)].

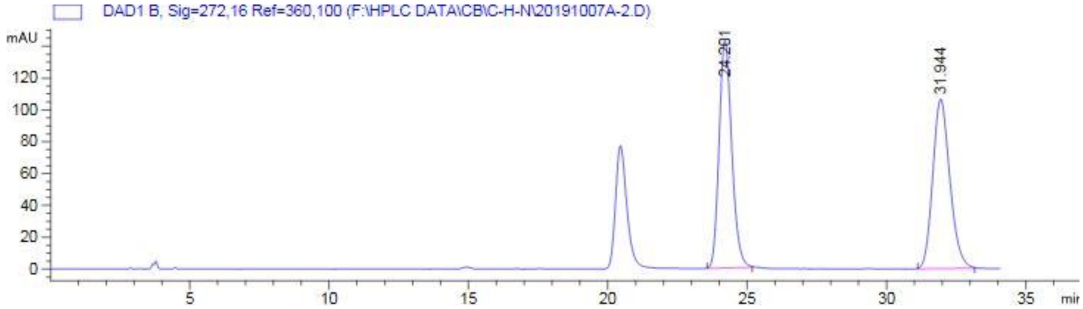

Peak #	RetTime [min]	Type	Width [min]	Area [mAU*s]	Height [mAU]	Area %
1	24.201	BB	0.4863	4509.97461	142.84735	49.8937
2	31.944	BB	0.6526	4529.18750	106.41498	50.1063

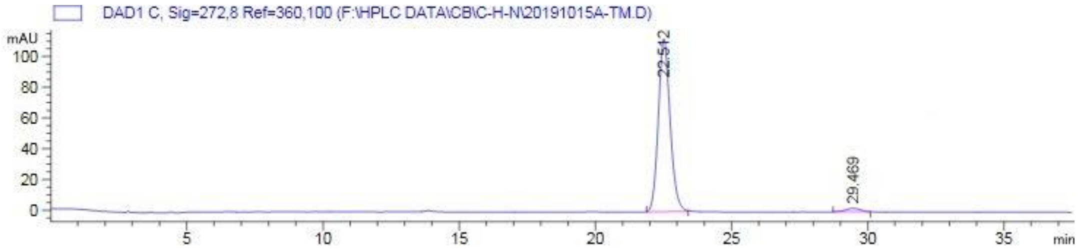

Peak #	RetTime [min]	Type	Width [min]	Area [mAU*s]	Height [mAU]	Area %
1	22.512	BB	0.4604	3374.49463	112.36674	97.5363
2	29.469	MM	0.6371	85.23764	2.22979	2.4637

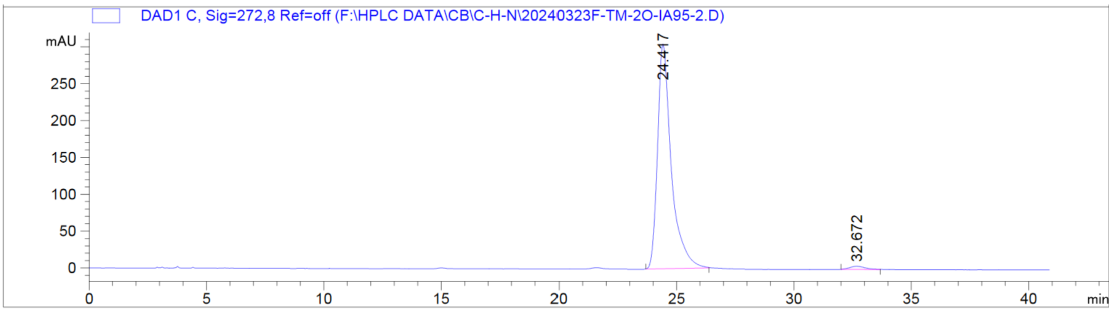

Peak #	RetTime [min]	Type	Width [min]	Area [mAU*s]	Height [mAU]	Area %
1	24.417	BB	0.5956	1.24400e4	305.16516	98.5107
2	32.672	BB	0.5637	188.07396	4.15769	1.4893

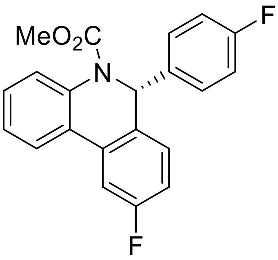

2p (The top one is racemic, and the bottom one is chiral)

The enantiomeric excess was determined by HPLC analysis using a chiral stationary phase column [Daicel chiracel® IA-3, 239 nm, n-hexane : i-PrOH = 95 : 5 as the eluent, flow rate: 1 mL/min, temperature 25 °C, retention time: 10.1 min (major isomer) and 12.9 min (minor isomer)].

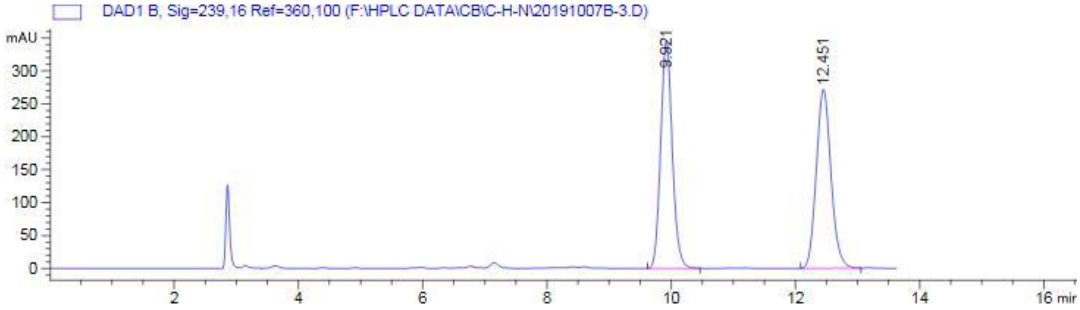

Peak #	RetTime [min]	Type	Width [min]	Area [mAU*s]	Height [mAU]	Area %
1	9.921	BB	0.1990	4482.30029	346.20590	50.1509
2	12.451	BB	0.2514	4455.31787	271.57114	49.8491

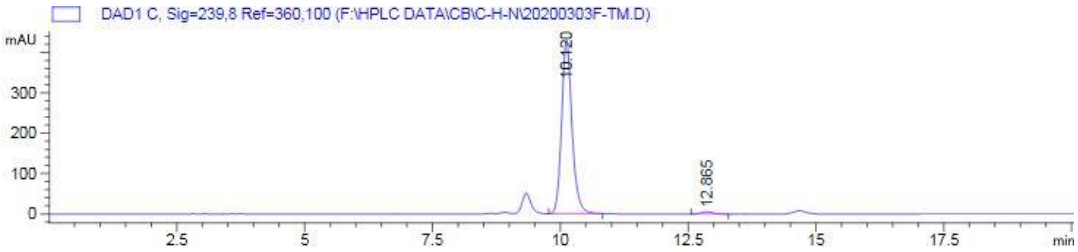

Peak #	RetTime [min]	Type	Width [min]	Area [mAU*s]	Height [mAU]	Area %
1	10.120	BB	0.2131	6098.78809	431.04736	98.5841
2	12.865	BB	0.2521	87.59198	5.21088	1.4159

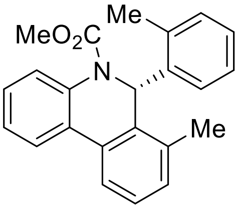

2q (The top one is racemic, and the bottom one is chiral)

The enantiomeric excess was determined by HPLC analysis using a chiral stationary phase column [Daicel chiracel® OD-H, 268 nm, n-hexane : i-PrOH = 99 : 1 as the eluent, flow rate: 1 mL/min, temperature 25 °C, retention time: 20.2 min (major isomer) and 8.4 min (minor isomer)].

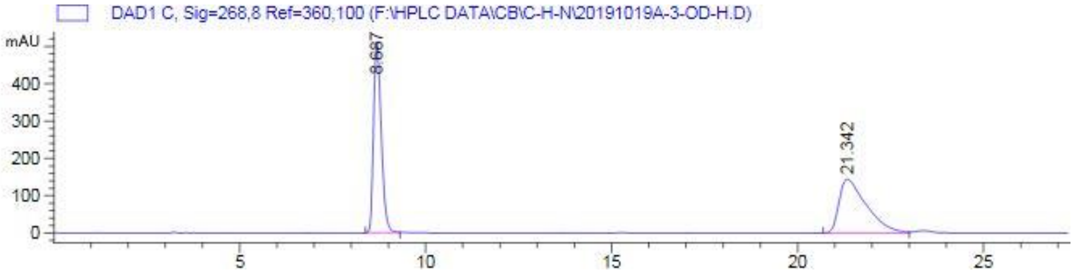

Peak #	RetTime [min]	Type	Width [min]	Area [mAU*s]	Height [mAU]	Area %
1	8.687	BB	0.2187	7329.95313	513.02429	49.7499
2	21.342	BB	0.7447	7403.65771	144.26688	50.2501

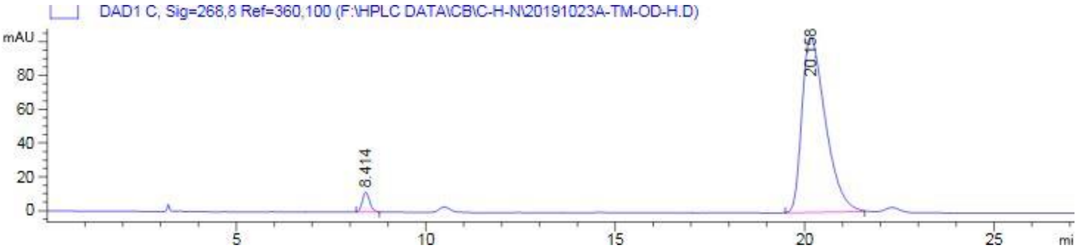

Peak #	RetTime [min]	Type	Width [min]	Area [mAU*s]	Height [mAU]	Area %
1	8.414	BB	0.2114	160.48123	11.74893	3.4664
2	20.158	BB	0.6596	4469.12646	103.53597	96.5336

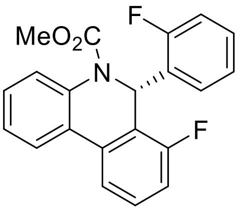

2r (The top one is racemic, and the following part is chiral)

The enantiomeric excess was determined by HPLC analysis using a chiral stationary phase column [Daicel chiracel® IA-3, 271 nm, n-hexane : i-PrOH = 95 : 5 as the eluent, flow rate: 1 mL/min, temperature 25 °C, retention time: 9.8 min (major isomer) and 8.4 min (minor isomer)].

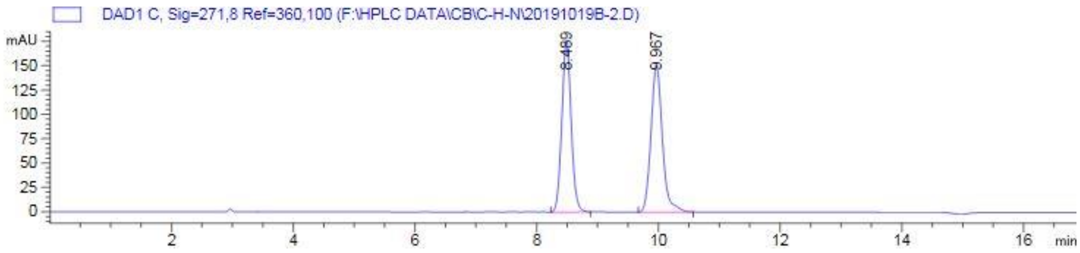

Peak #	RetTime [min]	Type	Width [min]	Area [mAU*s]	Height [mAU]	Area %
1	8.489	BB	0.1621	1876.90454	176.51308	48.6388
2	9.967	BB	0.1990	1981.95691	151.15642	51.3612

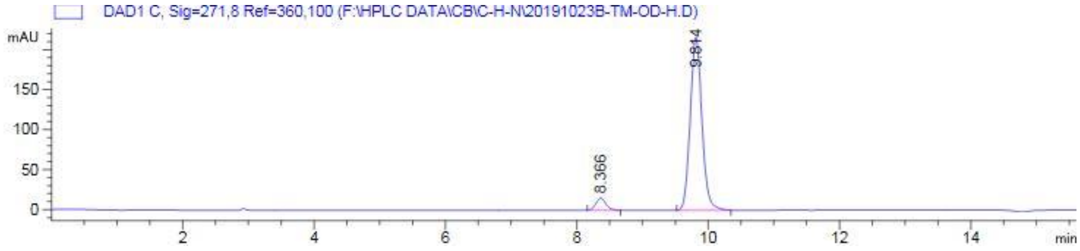

Peak #	RetTime [min]	Type	Width [min]	Area [mAU*s]	Height [mAU]	Area %
1	8.366	BB	0.1631	163.67769	15.26084	5.7489
2	9.814	BB	0.1887	2683.43091	216.34734	94.2511

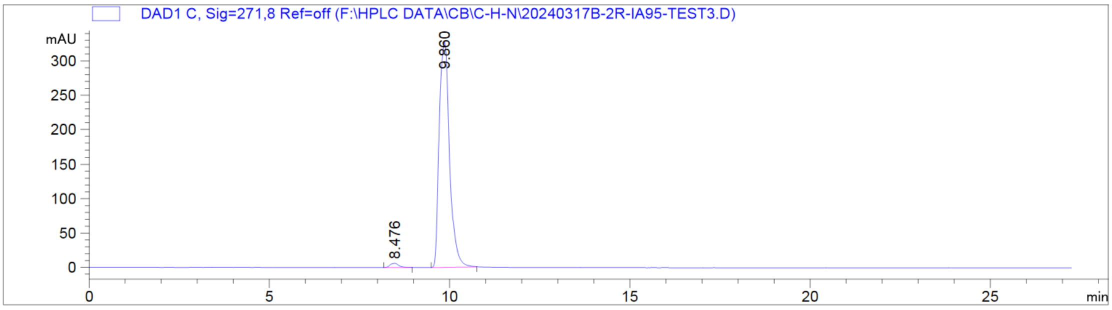

Peak #	RetTime [min]	Type	Width [min]	Area [mAU*s]	Height [mAU]	Area %
1	8.476	BB	0.2703	102.38579	6.02310	1.5488
2	9.860	BB	0.3137	6508.35596	327.73257	98.4512

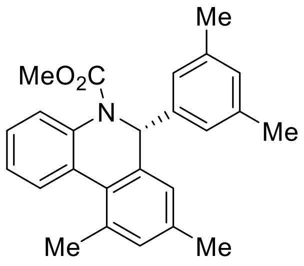

2s (The top one is racemic, and the following part is chiral)

The enantiomeric excess was determined by HPLC analysis using a chiral stationary phase column [Daicel chiracel® IC-3, 269 nm, n-hexane : i-PrOH = 99 : 1 as the eluent, flow rate: 1 mL/min, temperature 25 °C, retention time: 11.8 min (major isomer) and 15.3 min (minor isomer)].

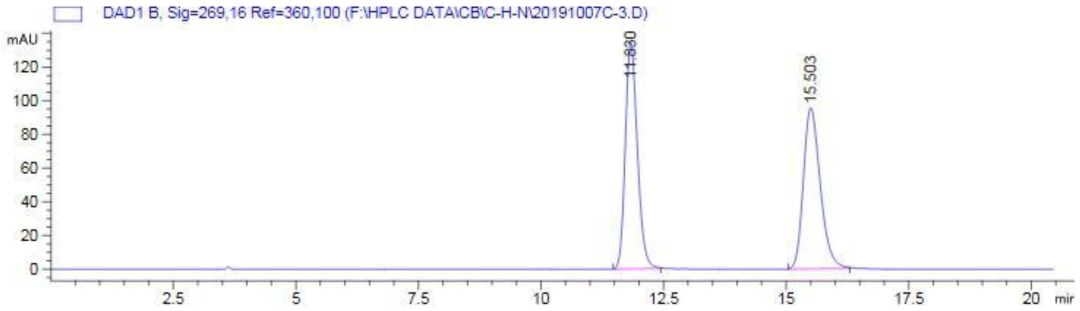

Peak #	RetTime [min]	Type	Width [min]	Area [mAU*s]	Height [mAU]	Area %
1	11.830	BB	0.2631	2297.19751	134.59047	50.0893
2	15.503	BB	0.3693	2289.00830	95.62372	49.9107

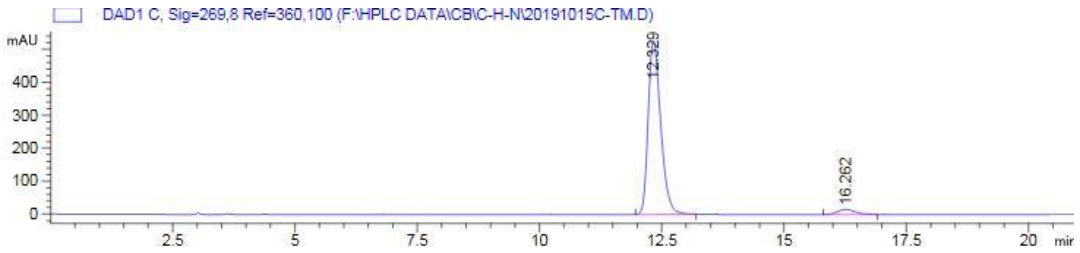

Peak #	RetTime [min]	Type	Width [min]	Area [mAU*s]	Height [mAU]	Area %
1	12.329	BB	0.2737	9381.94336	527.09271	96.1449
2	16.262	BB	0.3904	376.18402	14.90857	3.8551

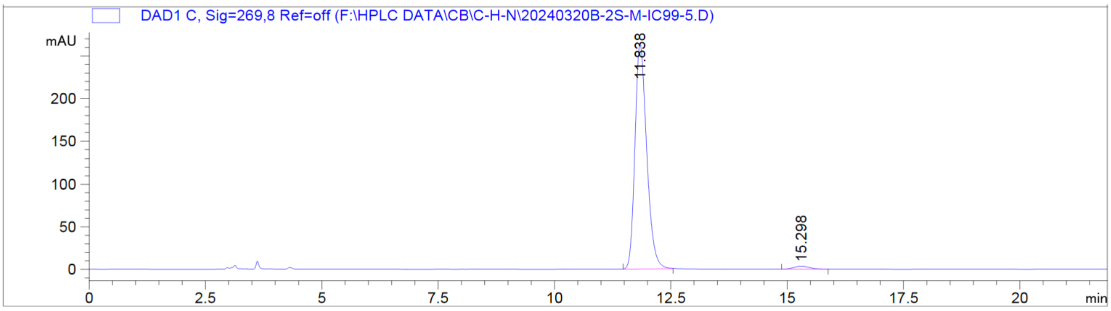

Peak #	RetTime [min]	Type	Width [min]	Area [mAU*s]	Height [mAU]	Area %
1	11.838	BB	0.2708	4630.81494	263.85962	98.2582
2	15.298	BB	0.3489	82.08994	3.45919	1.7418

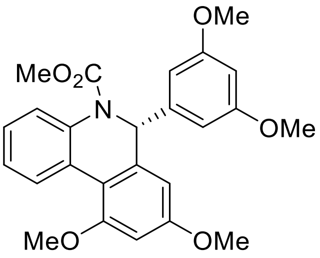

2t (The top one is racemic, and the bottom one is chiral)

The enantiomeric excess was determined by HPLC analysis using a chiral stationary phase column [Daicel chiracel® IA-3, 243 nm, n-hexane : i-PrOH = 80 : 20 as the eluent, flow rate: 1 mL/min, temperature 25 °C, retention time: 10.0 min (major isomer) and 19.5 min (minor isomer)].

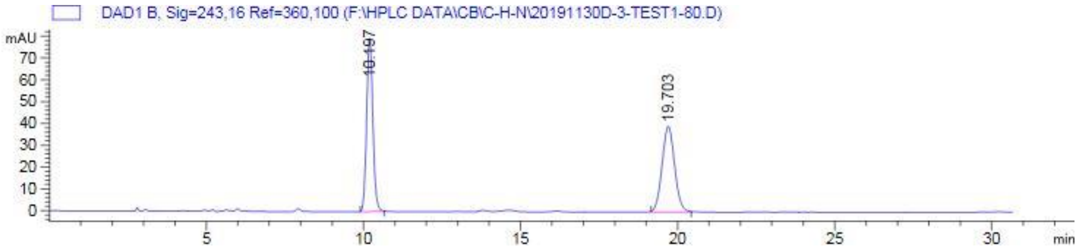

Peak #	RetTime [min]	Type	Width [min]	Area [mAU*s]	Height [mAU]	Area %
1	10.197	BB	0.2098	1087.31384	79.39254	49.9800
2	19.703	BB	0.4272	1088.18250	39.28413	50.0200

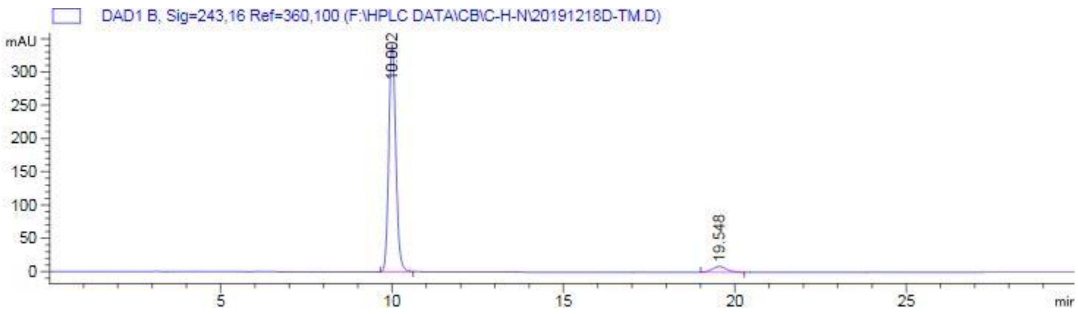

Peak #	RetTime [min]	Type	Width [min]	Area [mAU*s]	Height [mAU]	Area %
1	10.002	BB	0.2206	4881.19678	341.85281	95.3256
2	19.548	BB	0.4388	239.35420	8.39053	4.6744

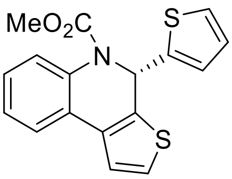

2u (The top one is racemic, and the bottom one is chiral)

The enantiomeric excess was determined by HPLC analysis using a chiral stationary phase column [Daicel chiracel® IA-3, 243 nm, n-hexane : i-PrOH = 95 : 5 as the eluent, flow rate: 1 mL/min, temperature 25 °C, retention time: 2.0 min (major isomer) and 15.2 min (minor isomer)].

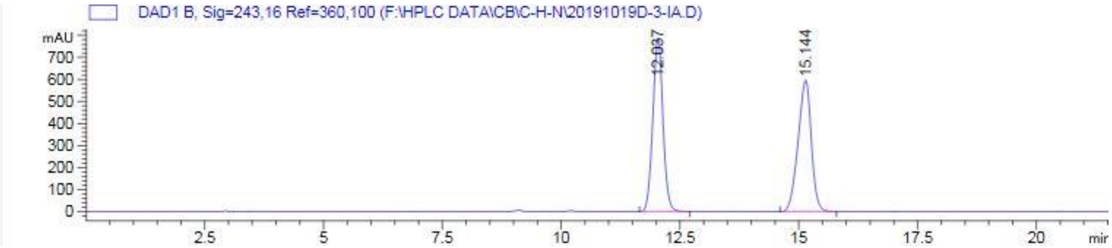

Peak #	RetTime [min]	Type	Width [min]	Area [mAU*s]	Height [mAU]	Area %
1	12.037	BB	0.2271	1.17006e4	788.46613	50.2725
2	15.144	BB	0.2964	1.15737e4	596.36591	49.7275

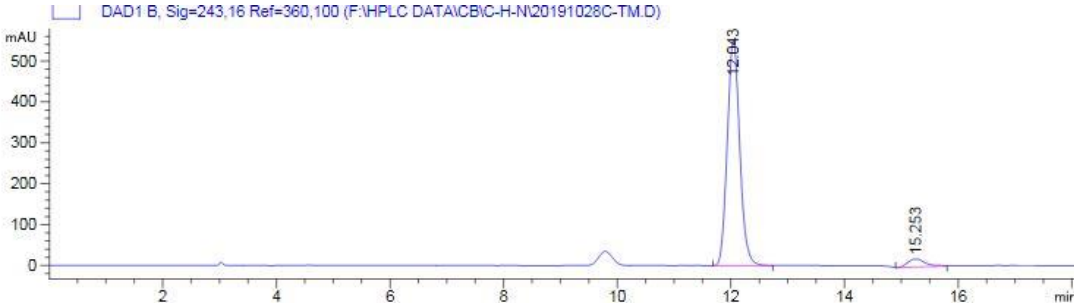

Peak #	RetTime [min]	Type	Width [min]	Area [mAU*s]	Height [mAU]	Area %
1	12.043	BB	0.2358	8405.17969	551.49994	95.3388
2	15.253	BB	0.3274	410.93863	19.53583	4.6612

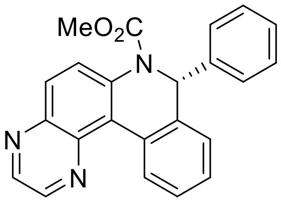

2v (The top one is racemic, and the bottom one is chiral)

The enantiomeric excess was determined by HPLC analysis using a chiral stationary phase column [Daicel chiracel® IC-3, 243 nm, n-hexane : i-PrOH = 90 : 10 as the eluent, flow rate: 1 mL/min, temperature 25 °C, retention time: 15.3 min (major isomer) and 13.0 min (minor isomer)].

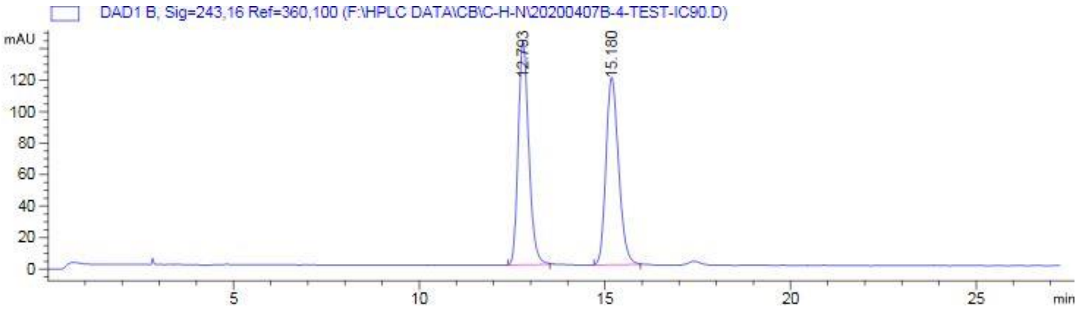

Peak #	RetTime [min]	Type	Width [min]	Area [mAU*s]	Height [mAU]	Area %
1	12.793	BB	0.3034	2808.79590	141.60092	49.9587
2	15.180	BB	0.3659	2813.44409	118.97124	50.0413

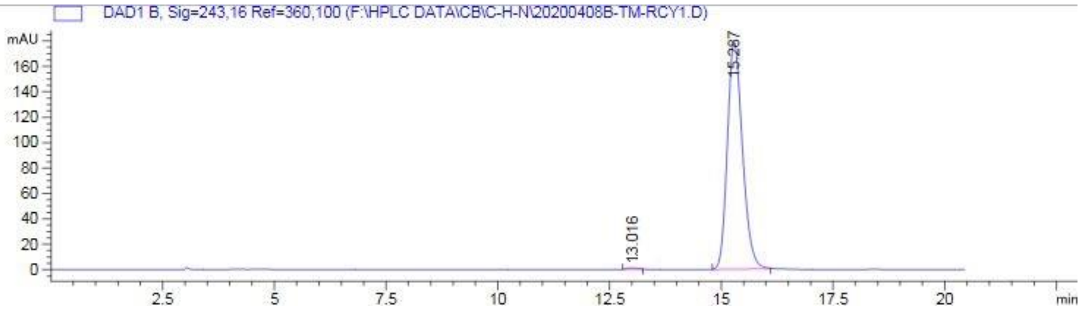

Peak #	RetTime [min]	Type	Width [min]	Area [mAU*s]	Height [mAU]	Area %
1	13.016	MM	0.2706	16.20317	9.98094e-1	0.3840
2	15.287	BB	0.3619	4202.85645	179.01485	99.6160

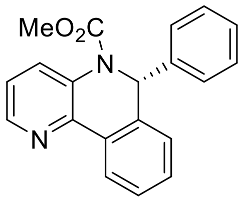

2w (The top one is racemic, and the bottom one is chiral)

The enantiomeric excess was determined by HPLC analysis using a chiral stationary phase column [Daicel chiracel® IA-3, 311 nm, n-hexane : i-PrOH = 95 : 5 as the eluent, flow rate: 1 mL/min, temperature 25 °C, retention time: 19.0 min (major isomer) and 13.6 min (minor isomer)].

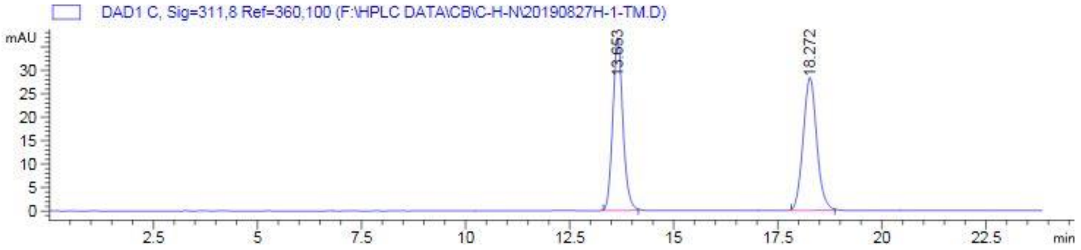

Peak #	RetTime [min]	Type	Width [min]	Area [mAU*s]	Height [mAU]	Area %
1	13.653	BB	0.2570	621.02515	36.78599	49.1417
2	18.272	BB	0.3491	642.71918	28.28987	50.8583

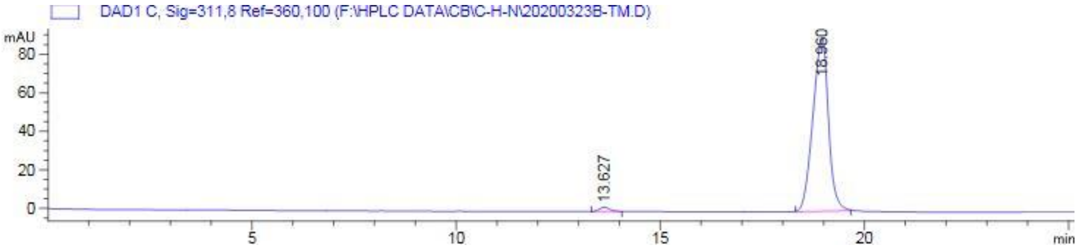

Peak #	RetTime [min]	Type	Width [min]	Area [mAU*s]	Height [mAU]	Area %
1	13.627	BB	0.2735	40.21088	2.23942	1.6553
2	18.960	BB	0.4126	2389.00293	90.30639	98.3447

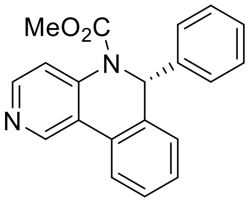

2x (The top one is racemic, and the bottom one is chiral)

The enantiomeric excess was determined by HPLC analysis using a chiral stationary phase column [Daicel chiracel® IA-3, 243 nm, n-hexane : i-PrOH =80 : 20 as the eluent, flow rate: 1 mL/min, temperature 25 °C, retention time: 23.3 min (major isomer) and 21.0 min (minor isomer)].

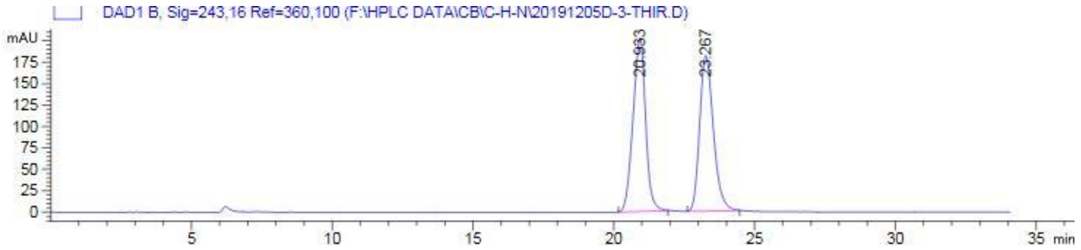

Peak #	RetTime [min]	Type	Width [min]	Area [mAU*s]	Height [mAU]	Area %
1	20.933	BB	0.4798	6278.73926	202.44008	50.4509
2	23.267	BB	0.5247	6166.49951	181.35492	49.5491

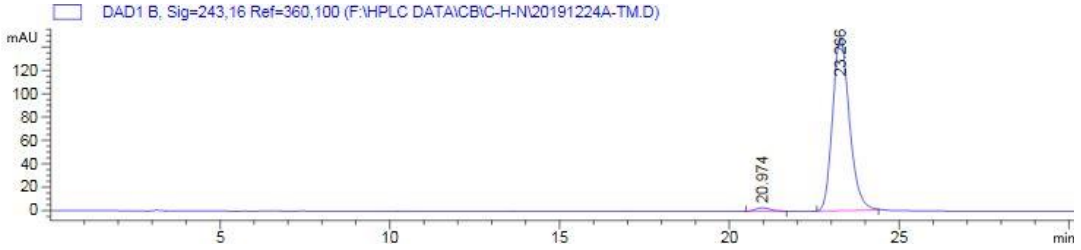

Peak #	RetTime [min]	Type	Width [min]	Area [mAU*s]	Height [mAU]	Area %
1	20.974	BB	0.4548	93.33823	3.01811	1.7879
2	23.266	BB	0.5319	5127.08594	148.07979	98.2121

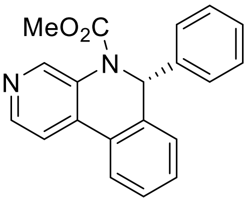

2y (The top one is racemic, and the bottom one is chiral)

The enantiomeric excess was determined by HPLC analysis using a chiral stationary phase column [Daicel chiracel® IC-3, 243 nm, n-hexane : i-PrOH = 50 : 50 as the eluent, flow rate: 1 mL/min, temperature 25 °C, retention time: 22.0 min (major isomer) and 29.9 min (minor isomer)].

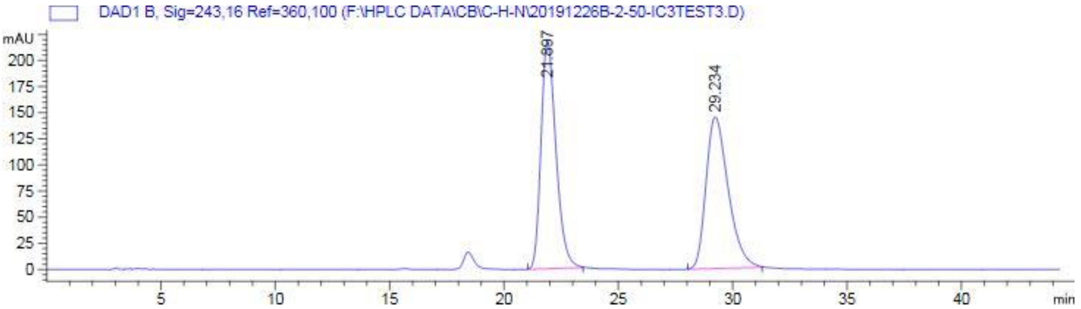

Peak #	RetTime [min]	Type	Width [min]	Area [mAU*s]	Height [mAU]	Area %
1	21.897	BB	0.6999	9877.83887	217.46878	50.2439
2	29.234	BB	1.0285	9781.91992	145.09756	49.7561

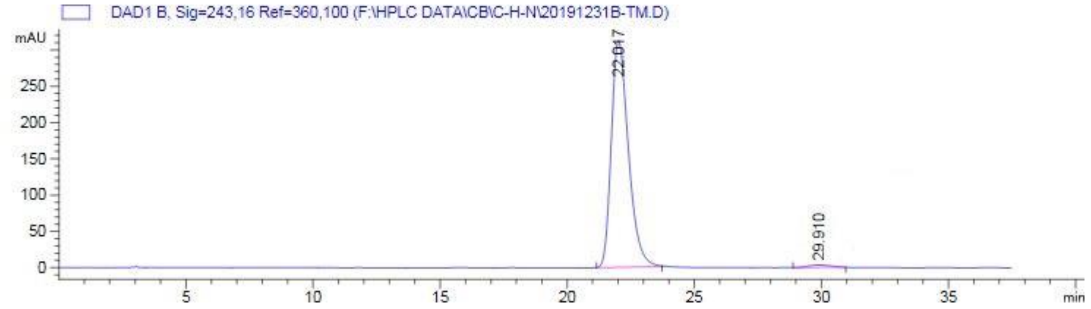

Peak #	RetTime [min]	Type	Width [min]	Area [mAU*s]	Height [mAU]	Area %
1	22.017	BB	0.7153	1.45423e4	312.25345	98.5631
2	29.910	MM	1.0549	212.00418	3.34964	1.4369

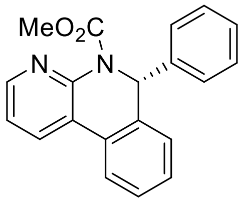

2z (The top one is racemic, and the bottom one is chiral)

The enantiomeric excess was determined by HPLC analysis using a chiral stationary phase column [Daicel chiracel® IA-3, 243 nm, n-hexane : i-PrOH = 80 : 20 as the eluent, flow rate: 1 mL/min, temperature 25 °C, retention time: 10.0 min (major isomer) and 12.6 min (minor isomer)].

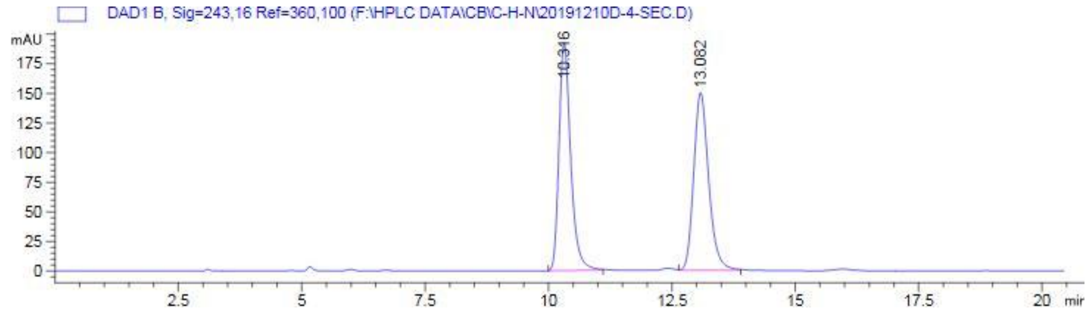

Peak #	RetTime [min]	Type	Width [min]	Area [mAU*s]	Height [mAU]	Area %
1	10.316	BB	0.2493	3157.70337	192.55995	50.0369
2	13.082	VB	0.3213	3153.04834	149.95753	49.9631

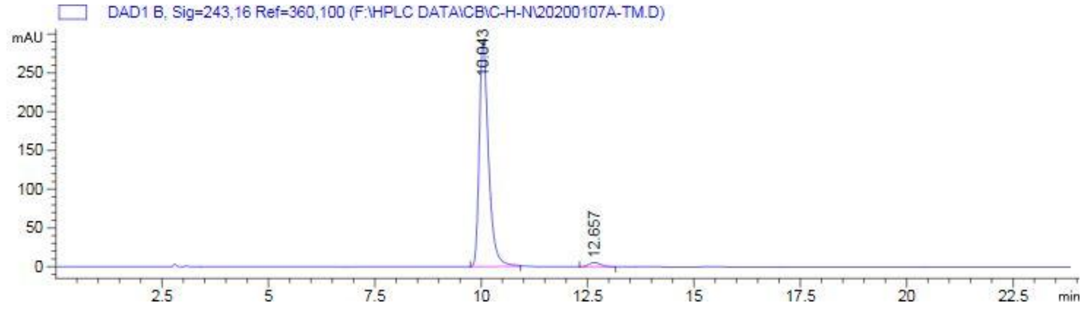

Peak #	RetTime [min]	Type	Width [min]	Area [mAU*s]	Height [mAU]	Area %
1	10.043	BB	0.2262	4403.14697	291.58386	97.5301
2	12.657	BB	0.3034	111.50732	5.57432	2.4699

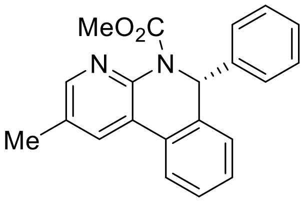

2aa (The top one is racemic, and the bottom one is chiral)

The enantiomeric excess was determined by HPLC analysis using a chiral stationary phase column [Daicel chiracel® IA-3, 243 nm, n-hexane : i-PrOH = 80 : 20 as the eluent, flow rate: 1 mL/min, temperature 25 °C, retention time: 8.0 min (major isomer) and 11.6 min (minor isomer)].

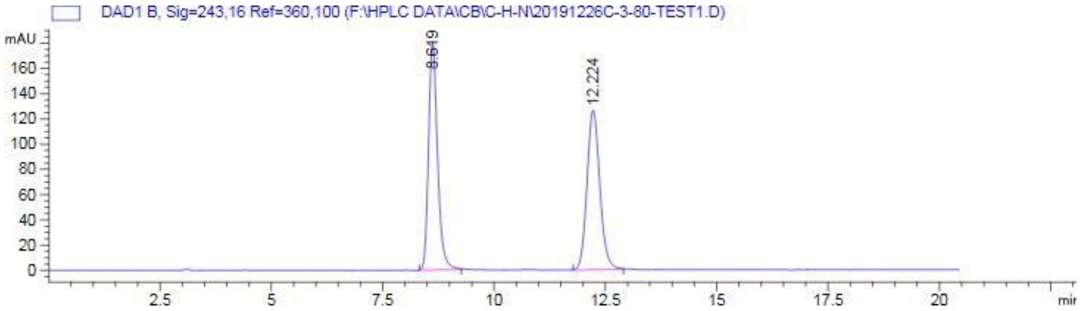

Peak #	RetTime [min]	Type	Width [min]	Area [mAU*s]	Height [mAU]	Area %
1	8.619	BB	0.2130	2526.07715	180.84827	49.9889
2	12.224	BB	0.3094	2527.20386	126.34669	50.0111

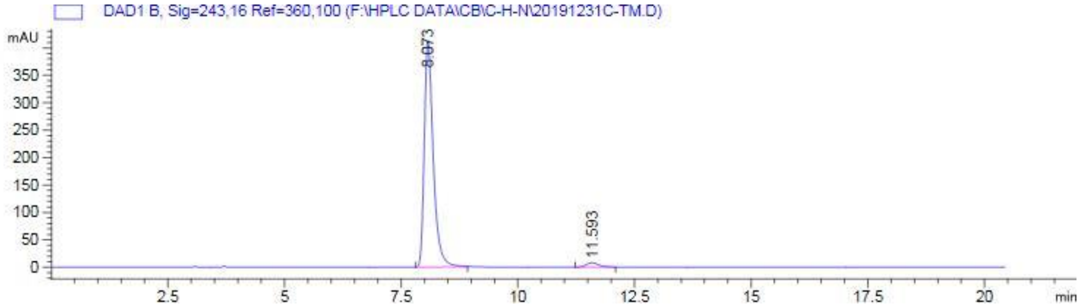

Peak #	RetTime [min]	Type	Width [min]	Area [mAU*s]	Height [mAU]	Area %
1	8.073	BB	0.2021	5535.19336	413.73541	97.3407
2	11.593	BB	0.2949	151.21690	7.84559	2.6593

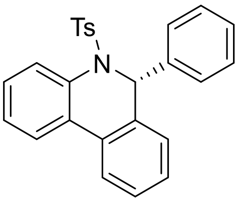

2ab (The top one is racemic, and the bottom one is chiral)

The enantiomeric excess was determined by HPLC analysis using a chiral stationary phase column [Daicel chiracel® OJ-H, 243 nm, n-hexane : i-PrOH = 98 : 2 as the eluent, flow rate: 1 mL/min, temperature 25 °C, retention time: 14.3 min (major isomer) and 23.8 min (minor isomer)].

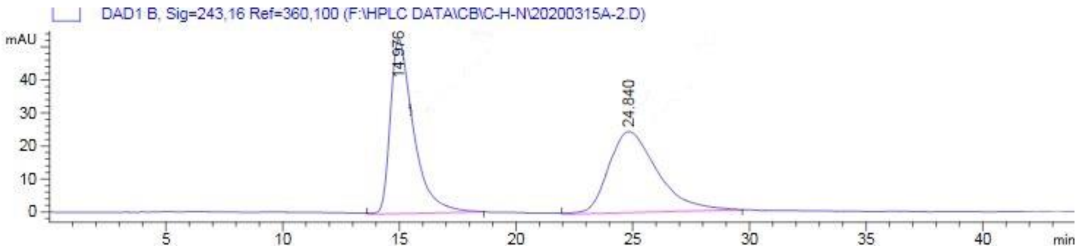

Peak #	RetTime [min]	Type	Width [min]	Area [mAU*s]	Height [mAU]	Area %
1	14.976	MM	1.1552	3654.64575	52.72535	50.5953
2	24.840	MM	2.4234	3568.64429	24.54289	49.4047

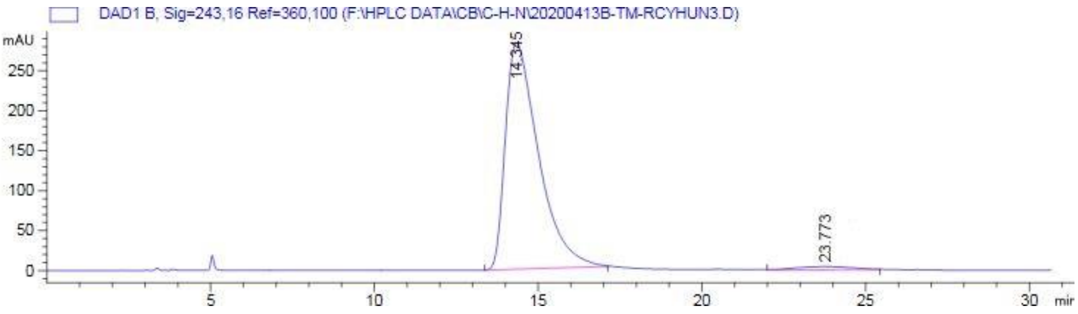

Peak #	RetTime [min]	Type	Width [min]	Area [mAU*s]	Height [mAU]	Area %
1	14.345	BB	1.0418	1.99426e4	283.10660	97.7820
2	23.773	MM	2.0075	452.37018	3.75561	2.2180

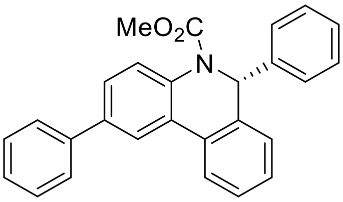

4a (The top one is racemic, and the bottom one is chiral)

The enantiomeric excess was determined by HPLC analysis using a chiral stationary phase column [Daicel chiracel® IA-3, 243 nm, n-hexane : i-PrOH = 95 : 5 as the eluent, flow rate: 1

mL/min, temperature 25 °C, retention time: 14.9 min (major isomer) and 20.1 min (minor isomer)].

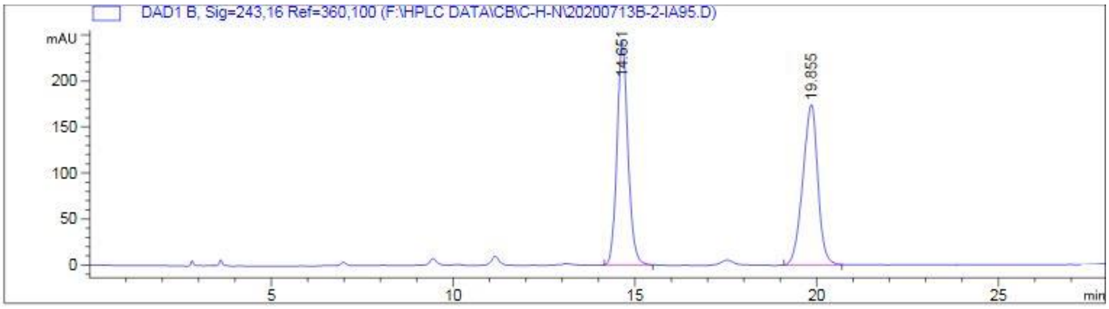

Peak #	RetTime [min]	Type	Width [min]	Area [mAU*s]	Height [mAU]	Area %
1	14.651	BB	0.3170	5100.73486	242.91899	50.3972
2	19.855	BB	0.4423	5020.32520	174.13815	49.6028

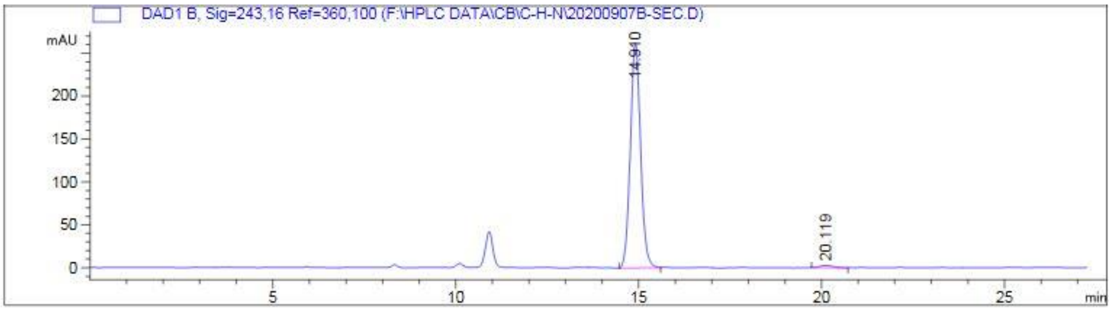

Peak #	RetTime [min]	Type	Width [min]	Area [mAU*s]	Height [mAU]	Area %
1	14.910	BB	0.2989	5099.05225	262.12656	98.7425
2	20.119	BB	0.3658	64.93774	2.70776	1.2575

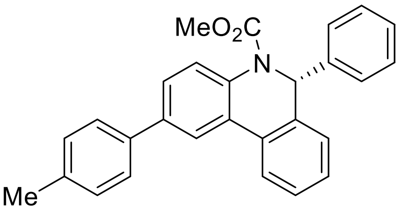

4b (The top one is racemic, and the bottom one is chiral)

The enantiomeric excess was determined by HPLC analysis using a chiral stationary phase column [Daicel chiracel® IA-3, 254 nm, n-hexane : i-PrOH = 95 : 5 as the eluent, flow rate: 1 mL/min, temperature 25 °C, retention time: 18.4 min (major isomer) and 20.1 min (minor isomer)].

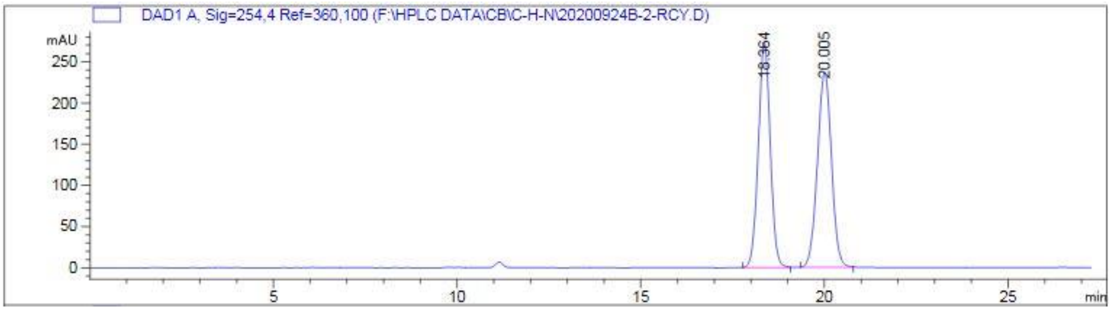

Peak #	RetTime [min]	Type	Width [min]	Area [mAU*s]	Height [mAU]	Area %
1	18.364	BB	0.3472	6149.77783	272.59775	49.4259
2	20.005	BB	0.4138	6292.64893	236.97548	50.5741

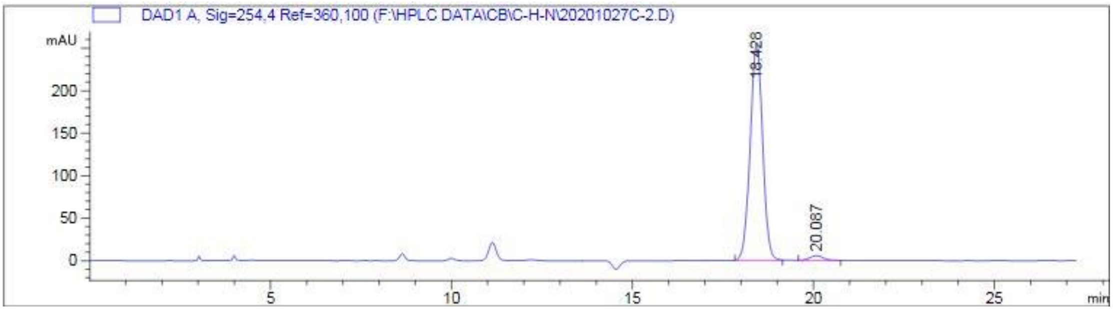

Peak #	RetTime [min]	Type	Width [min]	Area [mAU*s]	Height [mAU]	Area %
1	18.428	BB	0.3660	6096.35547	255.84612	97.6932
2	20.087	BB	0.4008	143.95433	5.51001	2.3068

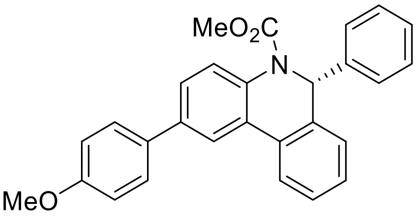

4c (The top one is racemic, and the bottom one is chiral)

The enantiomeric excess was determined by HPLC analysis using a chiral stationary phase column [Daicel chiracel® IA-3, 243 nm, n-hexane : i-PrOH = 97 : 3 as the eluent, flow rate: 1 mL/min, temperature 25 °C, retention time: 28.1 min (major isomer) and 30.1 min (minor isomer)].

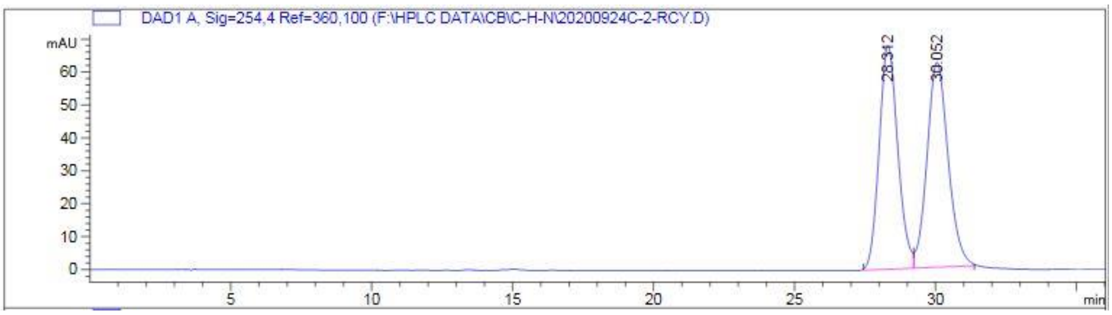

Peak #	RetTime [min]	Type	Width [min]	Area [mAU*s]	Height [mAU]	Area %
1	28.312	BV	0.7022	3112.84912	67.73035	49.0224
2	30.052	VB	0.7953	3237.00293	62.18324	50.9776

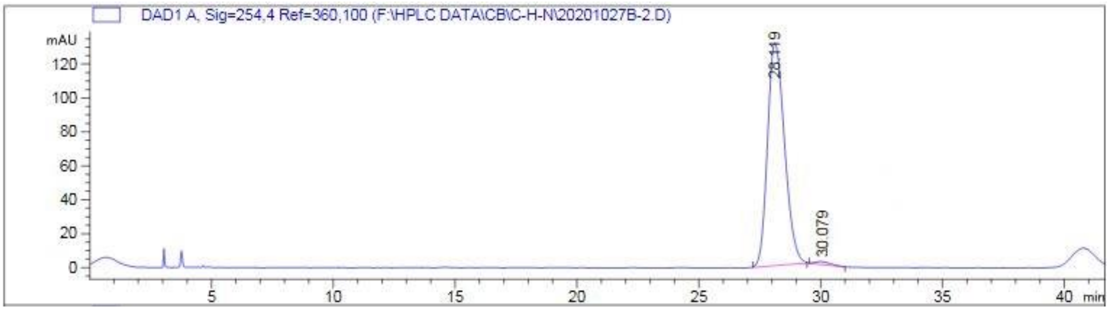

Peak #	RetTime [min]	Type	Width [min]	Area [mAU*s]	Height [mAU]	Area %
1	28.119	BB	0.7549	6359.65137	131.33835	98.8492
2	30.079	MM	0.7518	74.03966	1.64149	1.1508

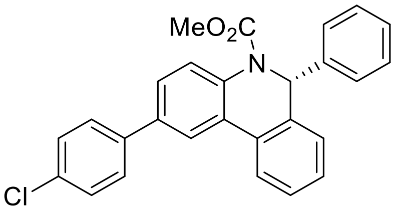

4d (The top one is racemic, and the bottom one is chiral)

The enantiomeric excess was determined by HPLC analysis using a chiral stationary phase column [Daicel chiracel® IA-3, 243 nm, n-hexane : i-PrOH = 95 : 5 as the eluent, flow rate: 1 mL/min, temperature 25 °C, retention time: 26.7 min (major isomer) and 30.0 min (minor isomer)].

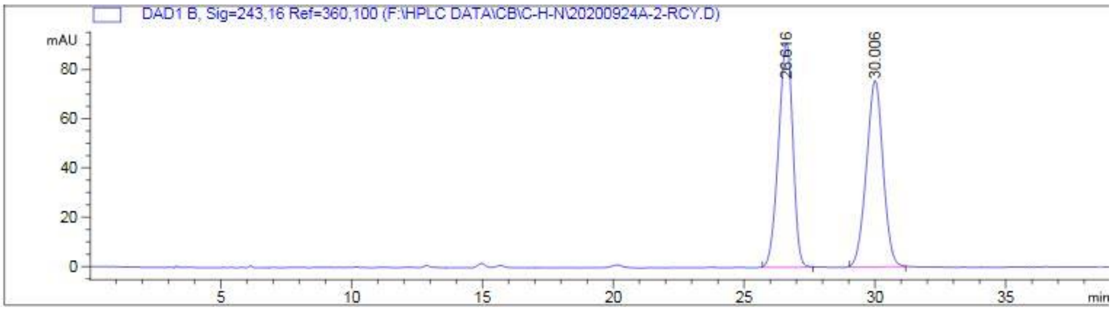

Peak #	RetTime [min]	Type	Width [min]	Area [mAU*s]	Height [mAU]	Area %
1	26.616	BB	0.5963	3466.17188	91.01058	50.8911
2	30.006	BB	0.6738	3344.78906	75.65264	49.1089

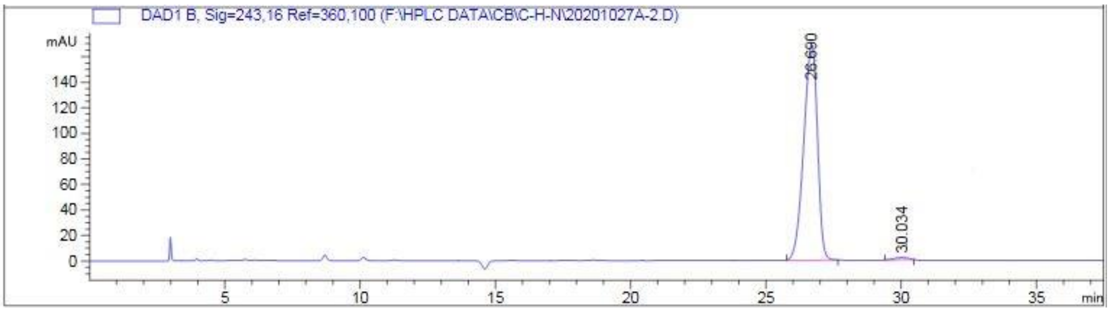

Peak #	RetTime [min]	Type	Width [min]	Area [mAU*s]	Height [mAU]	Area %
1	26.690	BB	0.5570	5956.38721	169.06665	98.9409
2	30.034	MM	0.5776	63.76073	1.83979	1.0591

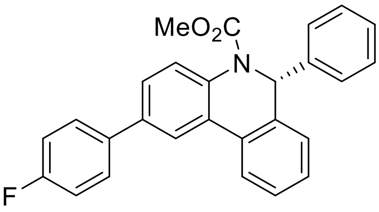

4e (The top one is racemic, and the bottom one is chiral)

The enantiomeric excess was determined by HPLC analysis using a chiral stationary phase column [Daicel chiracel® IA-3, 254 nm, n-hexane : i-PrOH = 95 : 5 as the eluent, flow rate: 1 mL/min, temperature 25 °C, retention time: 20.0 min (major isomer) and 25.4 min (minor isomer)].

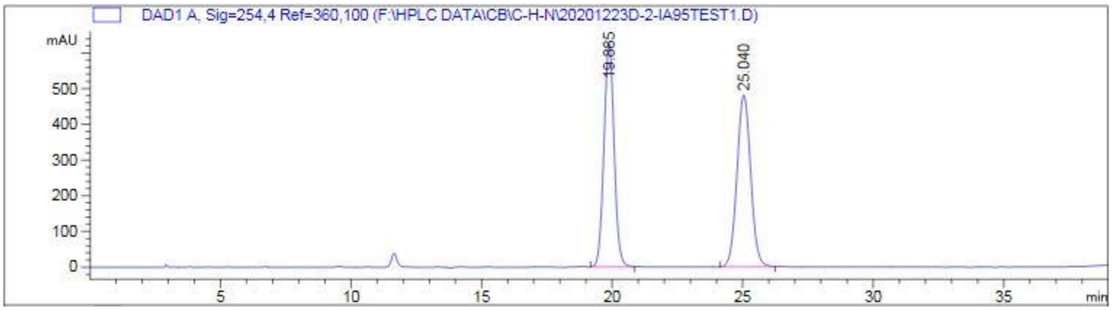

Peak #	RetTime [min]	Type	Width [min]	Area [mAU*s]	Height [mAU]	Area %
1	19.885	BB	0.4192	1.69424e4	627.15485	49.0974
2	25.040	BB	0.5701	1.75653e4	480.99524	50.9026

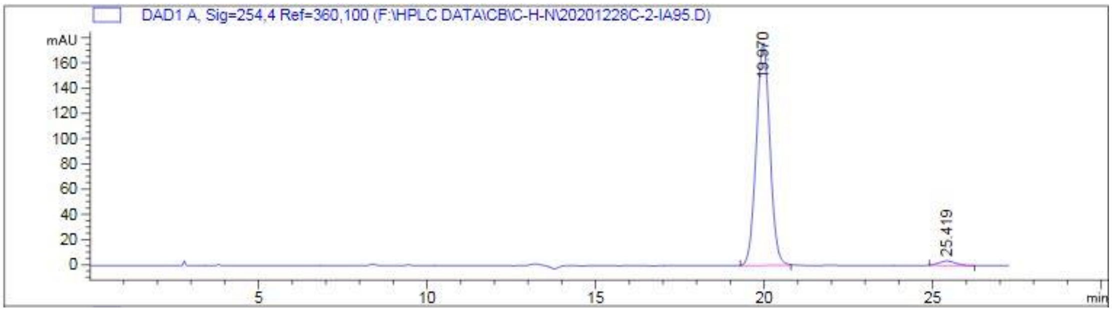

Peak #	RetTime [min]	Type	Width [min]	Area [mAU*s]	Height [mAU]	Area %
1	19.970	BB	0.4362	4959.86572	176.28453	97.6304
2	25.419	BB	0.5067	120.38057	3.47019	2.3696

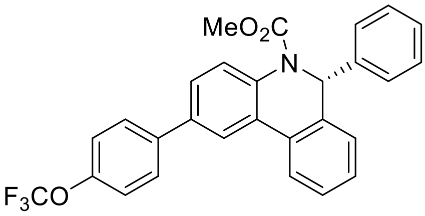

4f (The top one is racemic, and the bottom one is chiral)

The enantiomeric excess was determined by HPLC analysis using a chiral stationary phase column [Daicel chiracel® IA-3, 243 nm, n-hexane : i-PrOH = 95 : 5 as the eluent, flow rate: 1 mL/min, temperature 25 °C, retention time: 17.6 min (major isomer) and 19.7 min (minor isomer)].

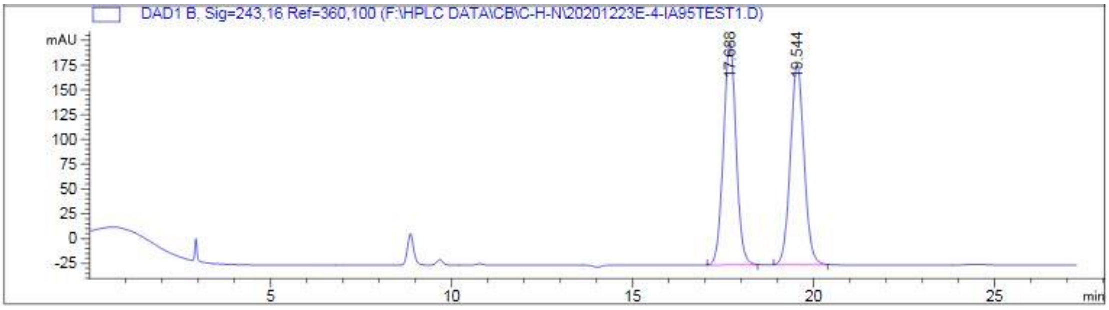

Peak #	RetTime [min]	Type	Width [min]	Area [mAU*s]	Height [mAU]	Area %
1	17.688	BB	0.3759	5458.00928	224.25938	50.6702
2	19.544	BB	0.4082	5313.61719	201.16371	49.3298

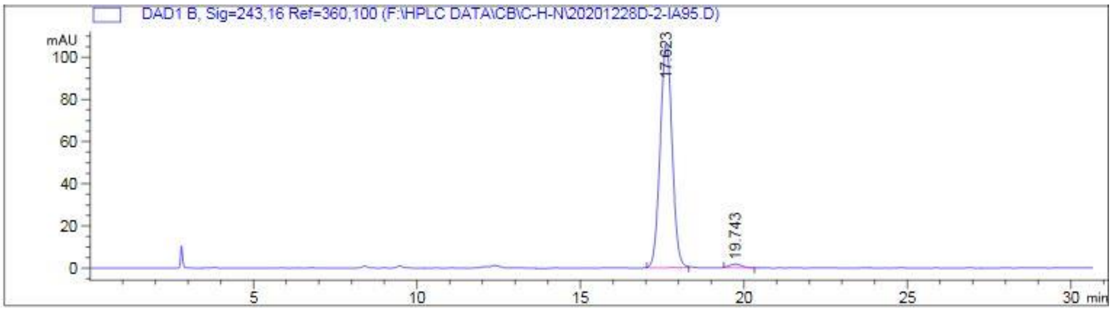

Peak #	RetTime [min]	Type	Width [min]	Area [mAU*s]	Height [mAU]	Area %
1	17.623	BB	0.3955	2698.31885	106.52623	98.4893
2	19.743	BB	0.3691	41.38799	1.60369	1.5107

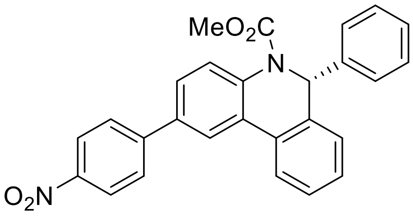

4g (The top one is racemic, and the bottom one is chiral)

The enantiomeric excess was determined by HPLC analysis using a chiral stationary phase column [Daicel chiracel® IA-3, 243 nm, n-hexane : i-PrOH = 80 : 20 as the eluent, flow rate: 1 mL/min, temperature 25 °C, retention time: 29.5 min (major isomer) and 31.8 min (minor isomer)].

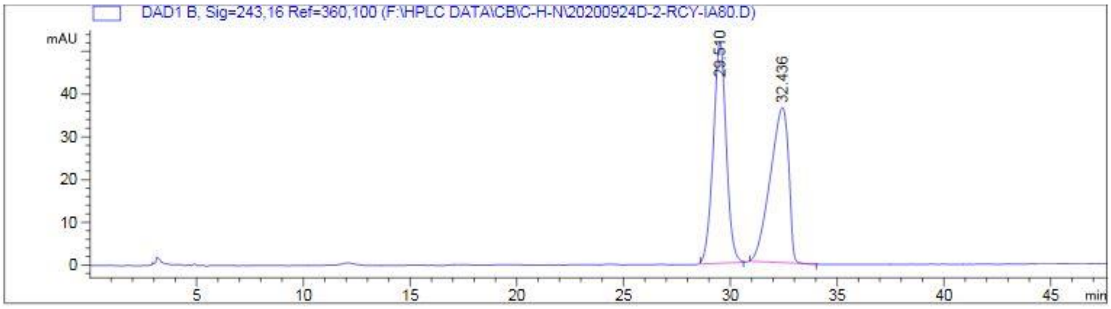

Peak #	RetTime [min]	Type	Width [min]	Area [mAU*s]	Height [mAU]	Area %
1	29.510	BB	0.6399	2187.84473	51.89537	50.1906
2	32.436	BB	0.9197	2171.23022	36.30416	49.8094

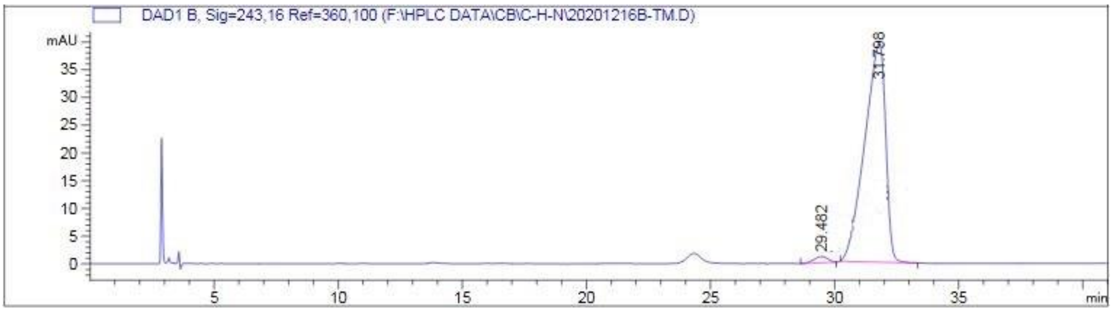

Peak #	RetTime [min]	Type	Width [min]	Area [mAU*s]	Height [mAU]	Area %
1	29.482	MM	0.6620	44.92133	1.13088	1.9039
2	31.798	BB	0.8732	2314.51978	39.41087	98.0961

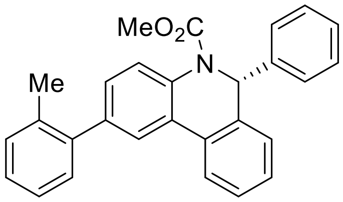

4h (The top one is racemic, and the bottom one is chiral)

The enantiomeric excess was determined by HPLC analysis using a chiral stationary phase

column [Daicel chiracel® IA-3, 254 nm, n-hexane : i-PrOH = 95 : 5 as the eluent, flow rate: 1 mL/min, temperature 25 °C, retention time: 10.0 min (major isomer) and 12.8 min (minor isomer)].

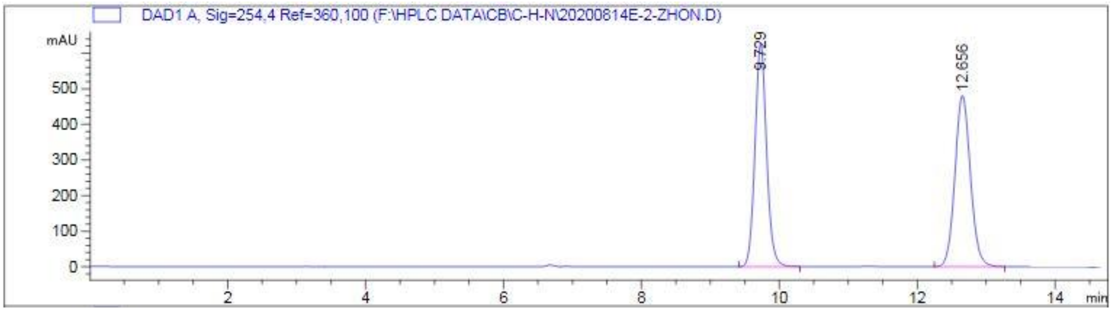

Peak #	RetTime [min]	Type	Width [min]	Area [mAU*s]	Height [mAU]	Area %
1	9.729	BB	0.1811	7386.34424	628.68793	49.5130
2	12.656	BB	0.2429	7531.65381	480.36279	50.4870

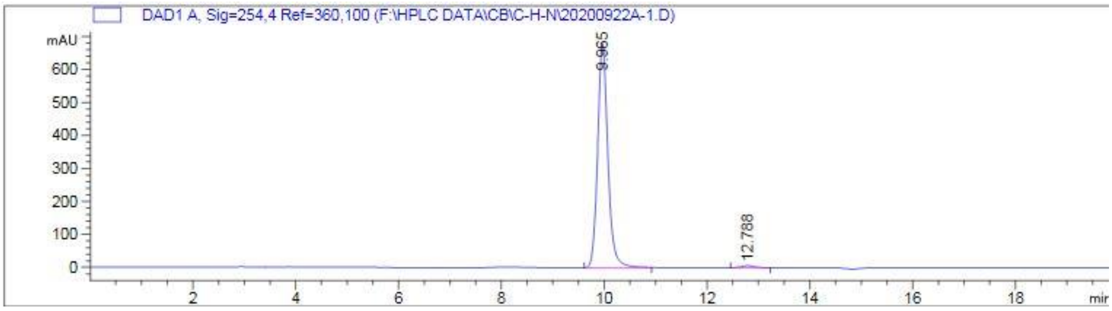

Peak #	RetTime [min]	Type	Width [min]	Area [mAU*s]	Height [mAU]	Area %
1	9.965	BB	0.2098	9445.22461	681.23547	98.8286
2	12.788	BB	0.2631	111.95216	6.49454	1.1714

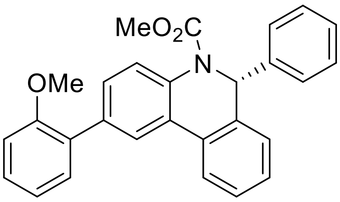

4i (The top one is racemic, and the bottom one is chiral)

The enantiomeric excess was determined by HPLC analysis using a chiral stationary phase column [Daicel chiracel® IA-3, 254 nm, n-hexane : i-PrOH = 95 : 5 as the eluent, flow rate: 1 mL/min, temperature 25 °C, retention time: 16.9 min (major isomer) and 28.6 min (minor isomer)].

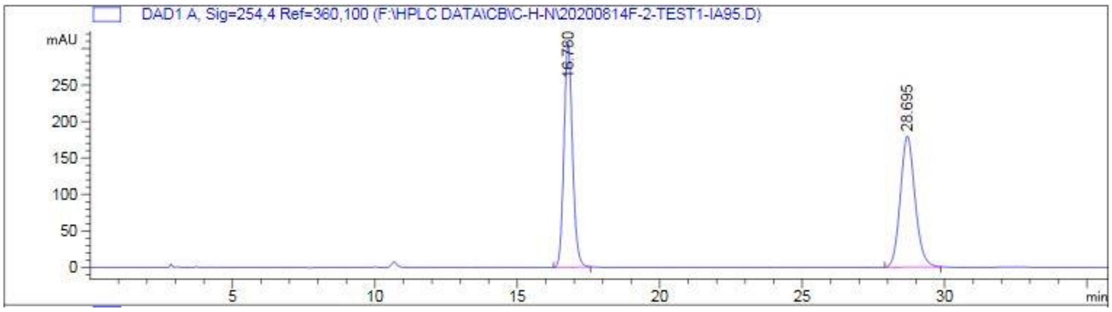

Peak #	RetTime [min]	Type	Width [min]	Area [mAU*s]	Height [mAU]	Area %
1	16.780	BB	0.3197	6435.80859	308.10681	49.8941
2	28.695	BB	0.5495	6463.12158	179.74939	50.1059

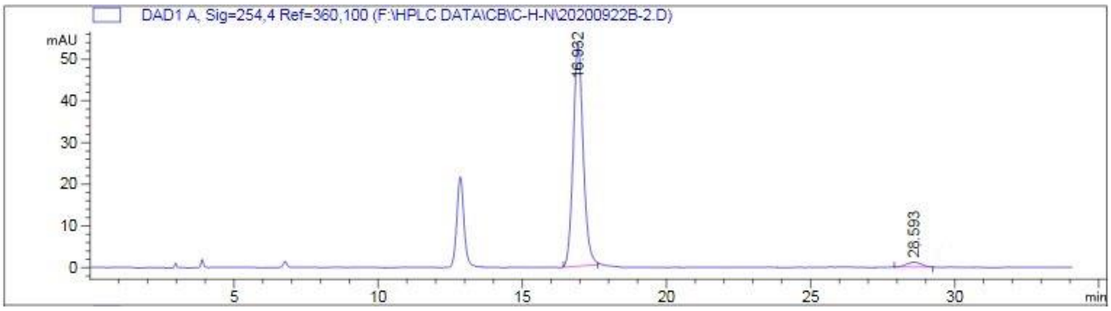

Peak #	RetTime [min]	Type	Width [min]	Area [mAU*s]	Height [mAU]	Area %
1	16.932	BB	0.3639	1284.83301	53.54974	96.9858
2	28.593	MM	0.5887	39.93133	1.13046	3.0142

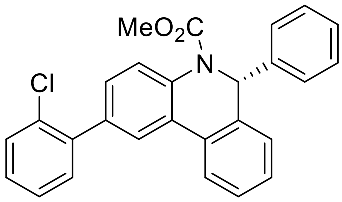

4j (The top one is racemic, and the bottom one is chiral)

The enantiomeric excess was determined by HPLC analysis using a chiral stationary phase column [Daicel chiracel® IA-3, 254 nm, n-hexane : i-PrOH = 95 : 5 as the eluent, flow rate: 1 mL/min, temperature 25 °C, retention time: 11.4 min (major isomer) and 14.2 min (minor isomer)].

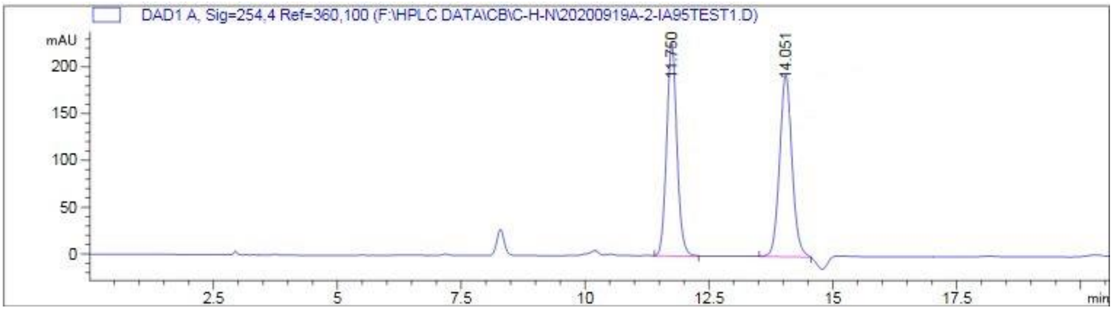

Peak #	RetTime [min]	Type	Width [min]	Area [mAU*s]	Height [mAU]	Area %
1	11.750	BB	0.2204	3284.67261	227.58588	49.1305
2	14.051	MM	0.2915	3400.93457	194.46841	50.8695

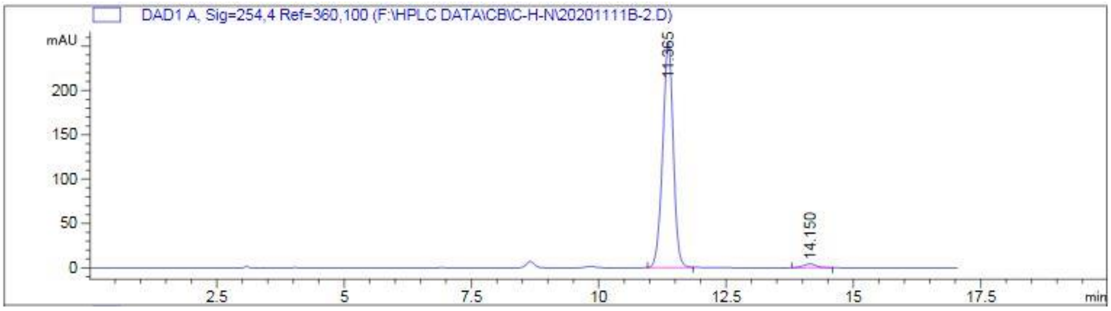

Peak #	RetTime [min]	Type	Width [min]	Area [mAU*s]	Height [mAU]	Area %
1	11.365	BB	0.2206	3662.63574	253.43010	97.9031
2	14.150	BB	0.2722	78.44510	4.35476	2.0969

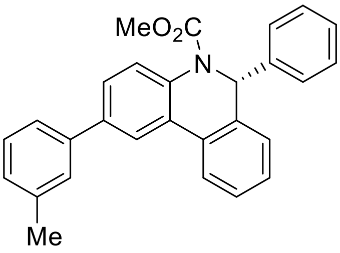

4k (The top one is racemic, and the bottom one is chiral)

The enantiomeric excess was determined by HPLC analysis using a chiral stationary phase column [Daicel chiracel® IA-3, 254 nm, n-hexane : i-PrOH = 95 : 5 as the eluent, flow rate: 1 mL/min, temperature 25 °C, retention time: 11.2 min (major isomer) and 17.7 min (minor isomer)].

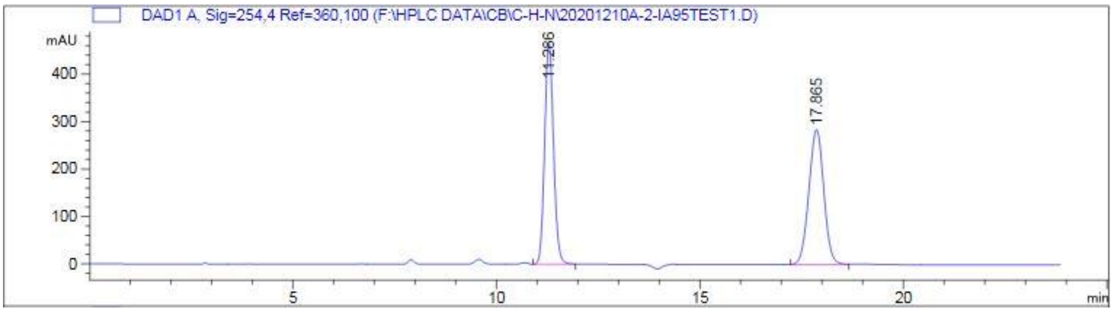

Peak #	RetTime [min]	Type	Width [min]	Area [mAU*s]	Height [mAU]	Area %
1	11.286	VB	0.2376	7106.49609	466.73471	49.5025
2	17.865	BB	0.3974	7249.33838	284.38239	50.4975

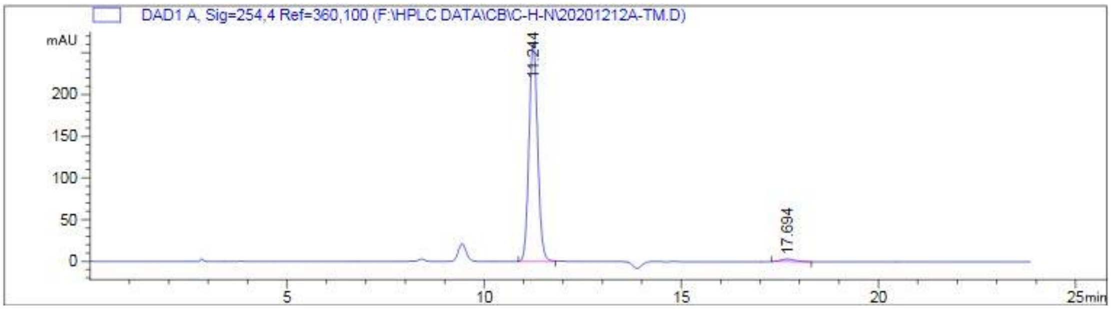

Peak #	RetTime [min]	Type	Width [min]	Area [mAU*s]	Height [mAU]	Area %
1	11.244	BB	0.2364	4003.80078	261.83206	98.1486
2	17.694	BB	0.3688	75.52406	3.11602	1.8514

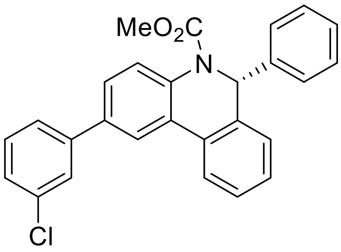

4l (The top one is racemic, and the bottom one is chiral)

The enantiomeric excess was determined by HPLC analysis using a chiral stationary phase column [Daicel chiracel® IA-3, 254 nm, n-hexane : i-PrOH = 95 : 5 as the eluent, flow rate: 1 mL/min, temperature 25 °C, retention time: 13.8 min (major isomer) and 21.5 min (minor isomer)].

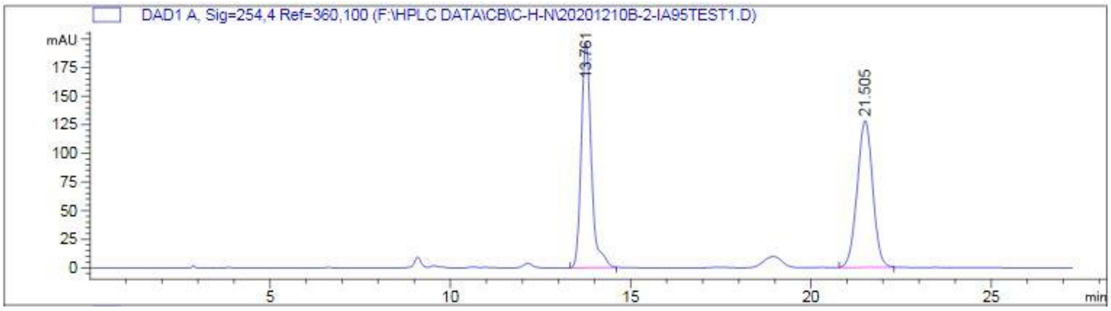

Peak #	RetTime [min]	Type	Width [min]	Area [mAU*s]	Height [mAU]	Area %
1	13.761	BB	0.2989	3824.47241	196.63258	49.8513
2	21.505	BB	0.4679	3847.28833	128.26395	50.1487

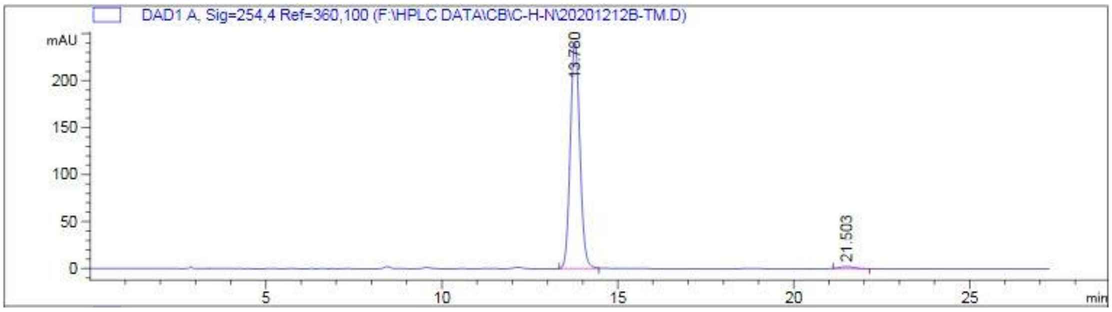

Peak #	RetTime [min]	Type	Width [min]	Area [mAU*s]	Height [mAU]	Area %
1	13.780	BB	0.2913	4508.82129	239.88438	98.8349
2	21.503	BB	0.4118	53.15369	1.96391	1.1651

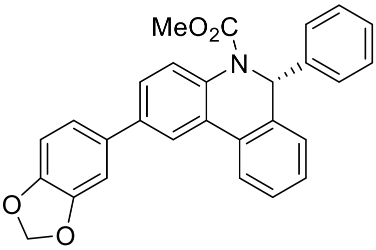

4m (The top one is racemic, and the bottom one is chiral)

The enantiomeric excess was determined by HPLC analysis using a chiral stationary phase column [Daicel chiracel® IA-3, 243 nm, n-hexane : i-PrOH = 85 : 15 as the eluent, flow rate: 1 mL/min, temperature 25 °C, retention time: 18.1 min (major isomer) and 19.3 min (minor isomer)].

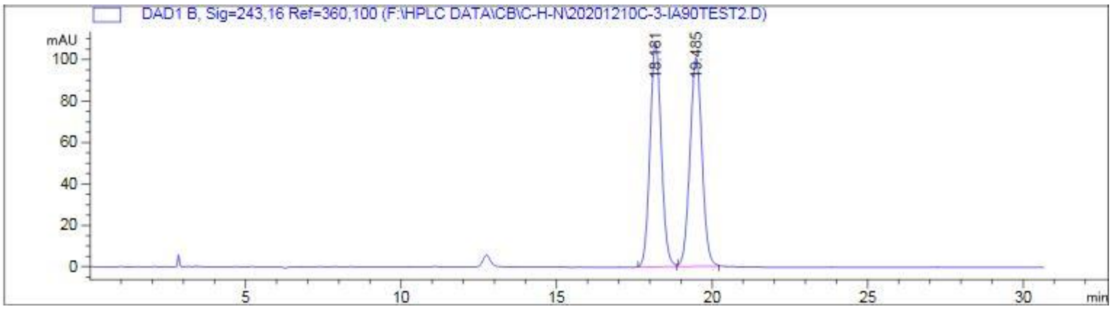

Peak #	RetTime [min]	Type	Width [min]	Area [mAU*s]	Height [mAU]	Area %
1	18.181	BB	0.3886	2708.89307	108.02280	50.0043
2	19.485	BB	0.4178	2708.42896	100.70557	49.9957

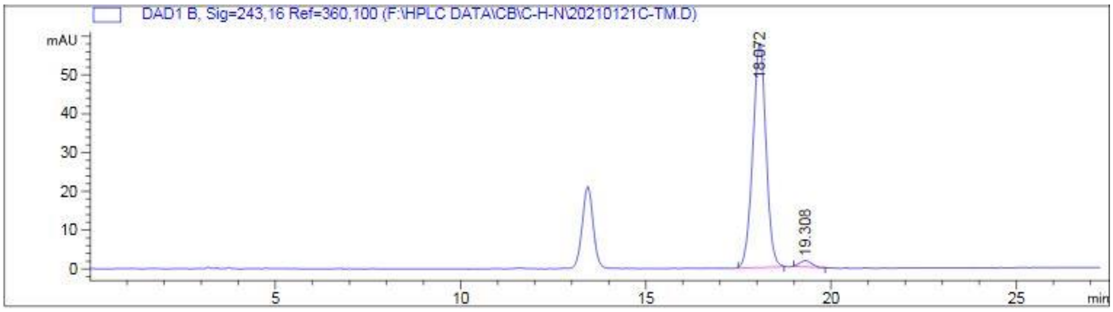

Peak #	RetTime [min]	Type	Width [min]	Area [mAU*s]	Height [mAU]	Area %
1	18.072	BB	0.3795	1437.38806	57.92026	97.5917
2	19.308	BB	0.3351	35.47121	1.57289	2.4083

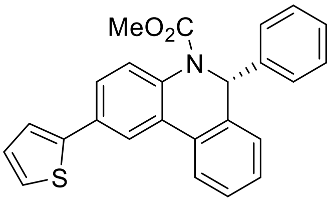

4n (The top one is racemic, and the bottom one is chiral)

The enantiomeric excess was determined by HPLC analysis using a chiral stationary phase column [Daicel chiracel® IA-3, 254 nm, n-hexane : i-PrOH = 95 : 5 as the eluent, flow rate: 1 mL/min, temperature 25 °C, retention time: 18.1 min (major isomer) and 22.5 min (minor isomer)].

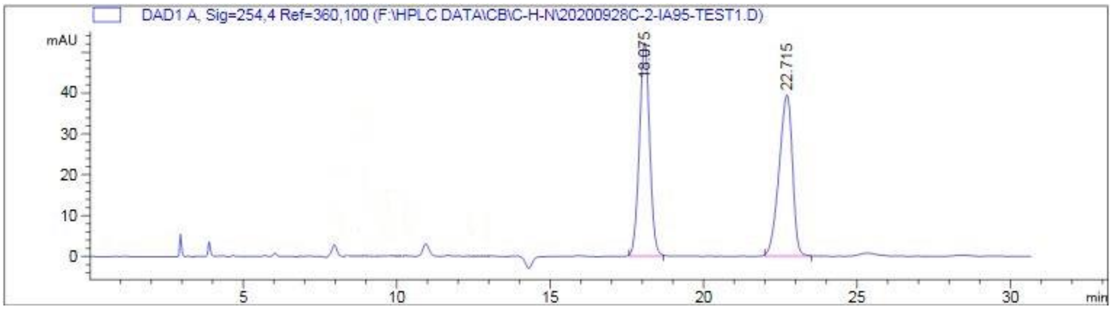

Peak #	RetTime [min]	Type	Width [min]	Area [mAU*s]	Height [mAU]	Area %
1	18.075	BB	0.3594	1213.87585	52.18439	50.3130
2	22.715	BB	0.4706	1198.77283	39.44518	49.6870

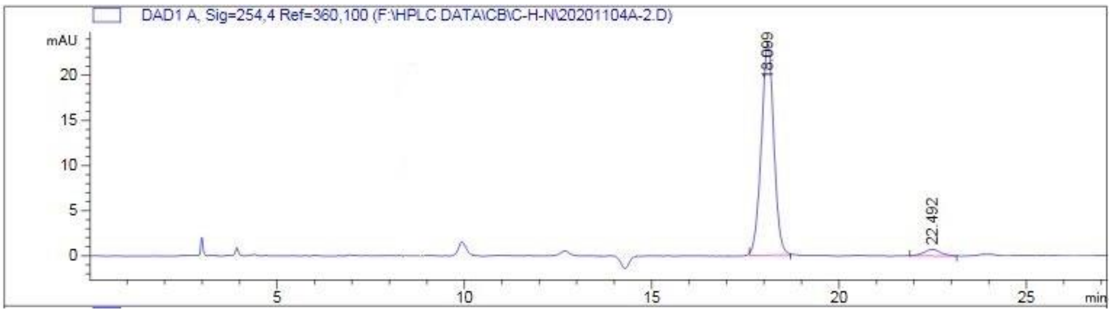

Peak #	RetTime [min]	Type	Width [min]	Area [mAU*s]	Height [mAU]	Area %
1	18.099	BB	0.3522	543.77002	23.47992	96.0578
2	22.492	MM	0.5174	22.31622	7.18836e-1	3.9422

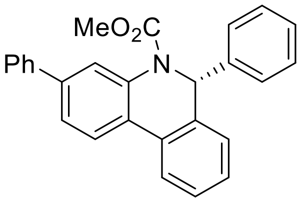

4o (The top one is racemic, and the bottom one is chiral)

The enantiomeric excess was determined by HPLC analysis using a chiral stationary phase column [Daicel chiracel® OD-H, 243 nm, n-hexane : i-PrOH = 98 : 2 as the eluent, flow rate: 1 mL/min, temperature 25 °C, retention time: 12.0 min (major isomer) and 13.8 min (minor isomer)].

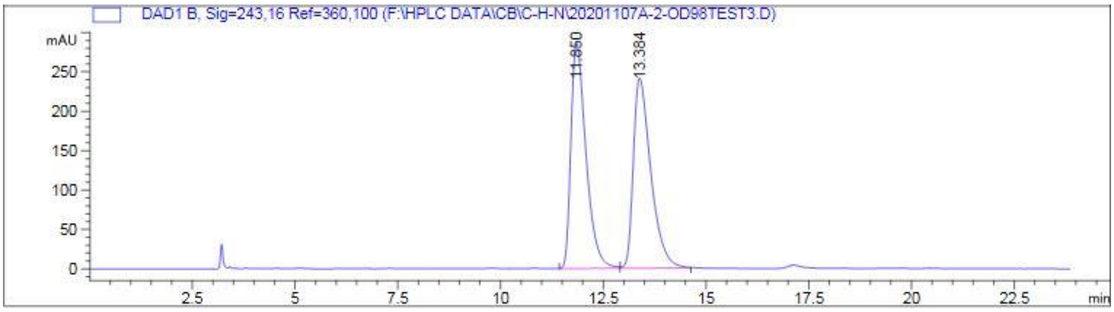

Peak #	RetTime [min]	Type	Width [min]	Area [mAU*s]	Height [mAU]	Area %
1	11.850	BB	0.3873	7296.42627	286.28793	50.5435
2	13.384	BB	0.4472	7139.50537	241.29283	49.4565

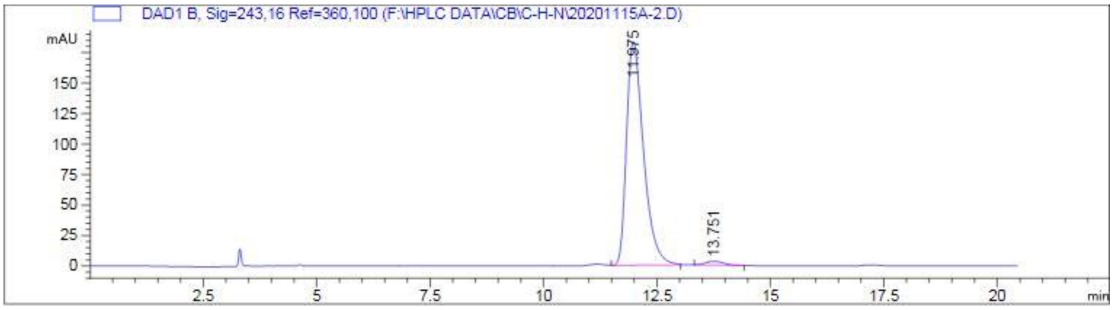

Peak #	RetTime [min]	Type	Width [min]	Area [mAU*s]	Height [mAU]	Area %
1	11.975	VB	0.3975	4767.91797	183.29230	98.1986
2	13.751	BB	0.4065	87.46362	3.16454	1.8014

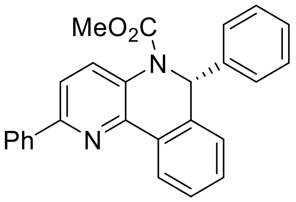

4p (The top one is racemic, and the bottom one is chiral)

The enantiomeric excess was determined by HPLC analysis using a chiral stationary phase column [Daicel chiracel® IC-3, 254 nm, n-hexane : i-PrOH = 97 : 3 as the eluent, flow rate: 1 mL/min, temperature 25 °C, retention time: 10.1 min (major isomer) and 9.0 min (minor isomer)].

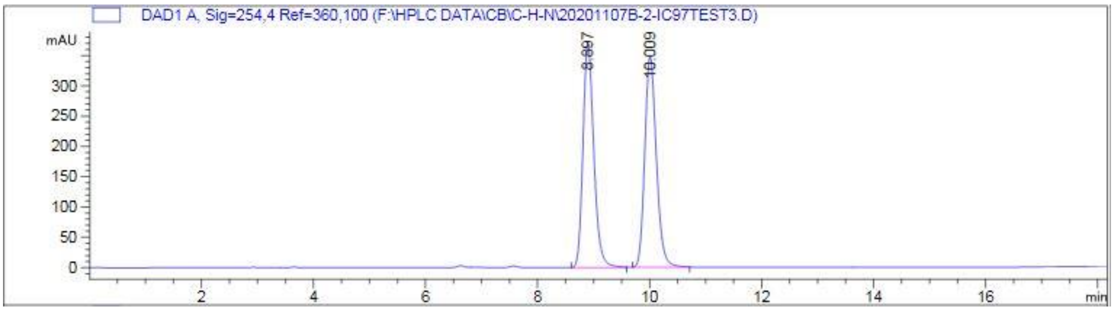

Peak #	RetTime [min]	Type	Width [min]	Area [mAU*s]	Height [mAU]	Area %
1	8.897	BB	0.2070	4988.81445	370.63040	49.9168
2	10.009	BB	0.2214	5005.44824	348.76828	50.0832

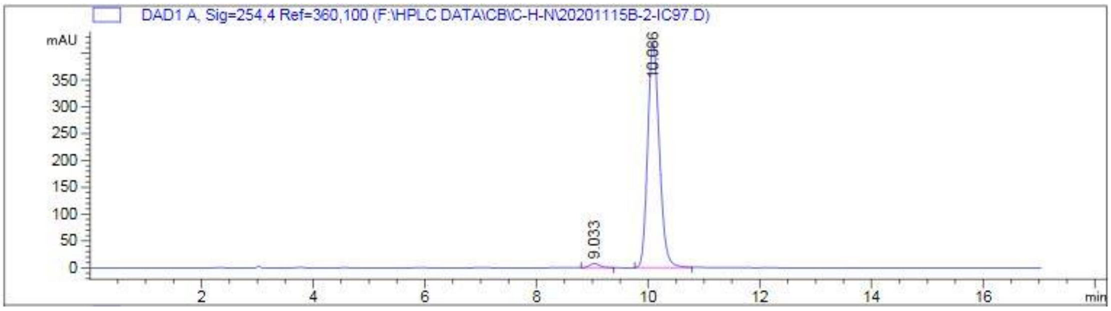

Peak #	RetTime [min]	Type	Width [min]	Area [mAU*s]	Height [mAU]	Area %
1	9.033	BB	0.1954	98.83155	7.82139	1.6004
2	10.086	BB	0.2253	6076.52148	418.79105	98.3996

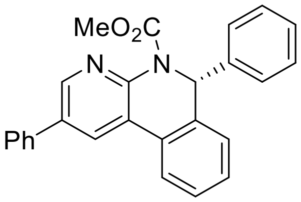

4q (The top one is racemic, and the bottom one is chiral)

The enantiomeric excess was determined by HPLC analysis using a chiral stationary phase column [Daicel chiracel® IA-3, 254 nm, n-hexane : i-PrOH = 85 : 15 as the eluent, flow rate: 1

mL/min, temperature 25 °C, retention time: 11.3 min (major isomer) and 19.8 min (minor isomer)].

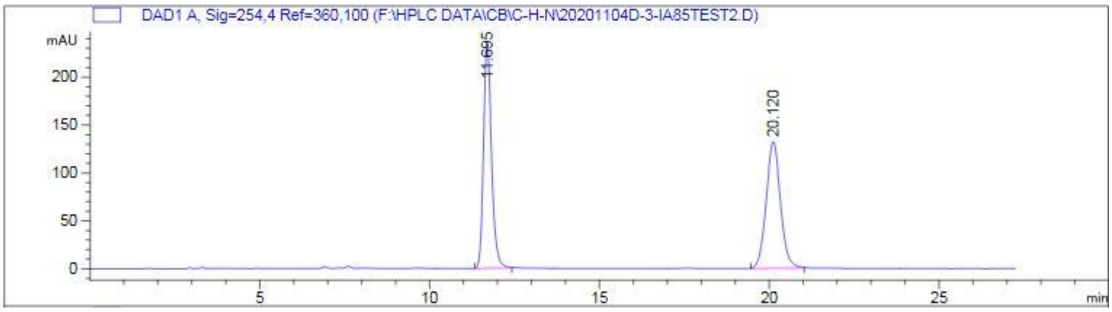

Peak #	RetTime [min]	Type	Width [min]	Area [mAU*s]	Height [mAU]	Area %
1	11.695	BB	0.2481	3827.93140	234.93831	49.9476
2	20.120	BB	0.4447	3835.96899	132.13922	50.0524

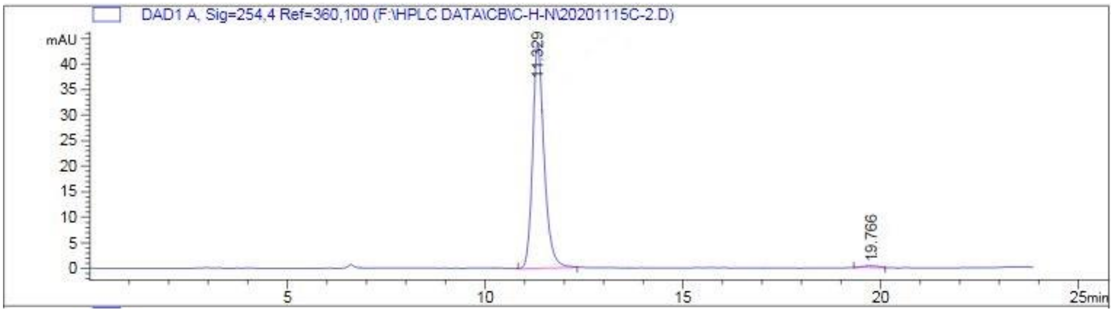

Peak #	RetTime [min]	Type	Width [min]	Area [mAU*s]	Height [mAU]	Area %
1	11.329	MM	0.3340	884.92291	44.15270	99.0880
2	19.766	MM	0.4217	8.14477	3.21884e-1	0.9120

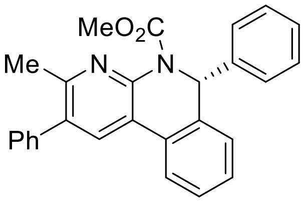

4r (The top one is racemic, and the bottom one is chiral)

The enantiomeric excess was determined by HPLC analysis using a chiral stationary phase column [Daicel chiracel® IC-3, 254 nm, n-hexane : i-PrOH = 80 : 20 as the eluent, flow rate: 1 mL/min, temperature 25 °C, retention time: 10.1 min (major isomer) and 6.9 min (minor isomer)].

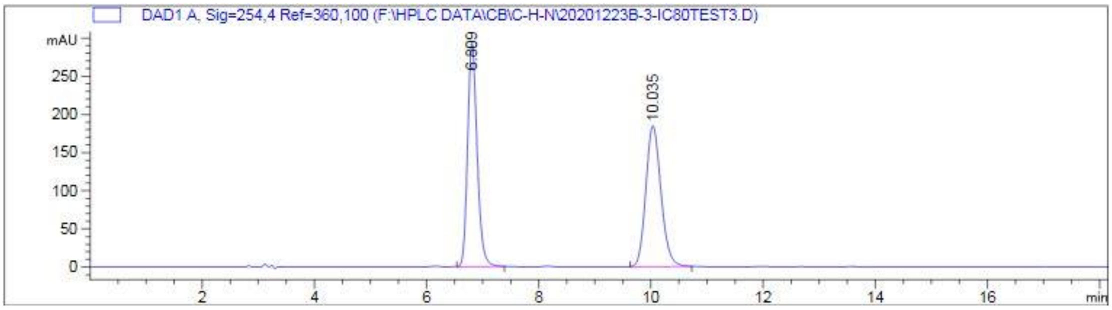

Peak #	RetTime [min]	Type	Width [min]	Area [mAU*s]	Height [mAU]	Area %
1	6.809	BB	0.1804	3476.80981	293.15146	50.0140
2	10.035	BB	0.2899	3474.86890	184.38571	49.9860

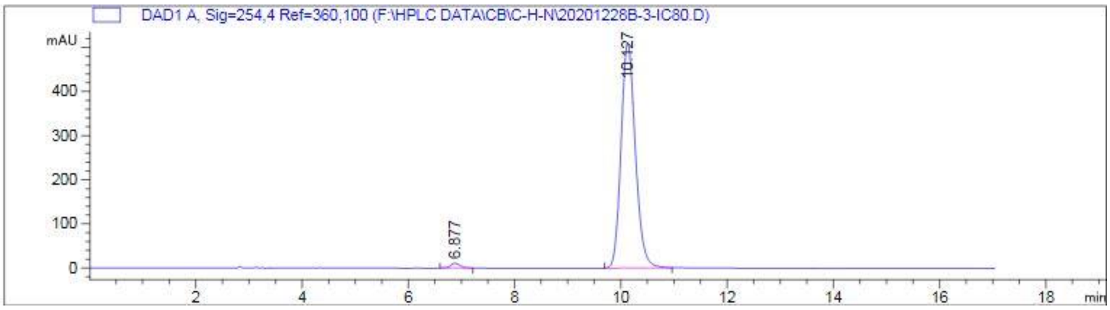

Peak #	RetTime [min]	Type	Width [min]	Area [mAU*s]	Height [mAU]	Area %
1	6.877	BB	0.1911	145.11449	11.51132	1.4982
2	10.127	BB	0.2883	9540.76465	509.94131	98.5018

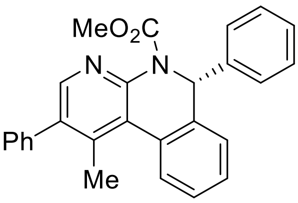

4s (The top one is racemic, and the bottom one is chiral)

The enantiomeric excess was determined by HPLC analysis using a chiral stationary phase column [Daicel chiracel® IE-3, 243 nm, n-hexane : i-PrOH =80 : 20 as the eluent, flow rate: 1 mL/min, temperature 25 °C, retention time: 21.6 min (major isomer) and 24.5 min (minor isomer)].

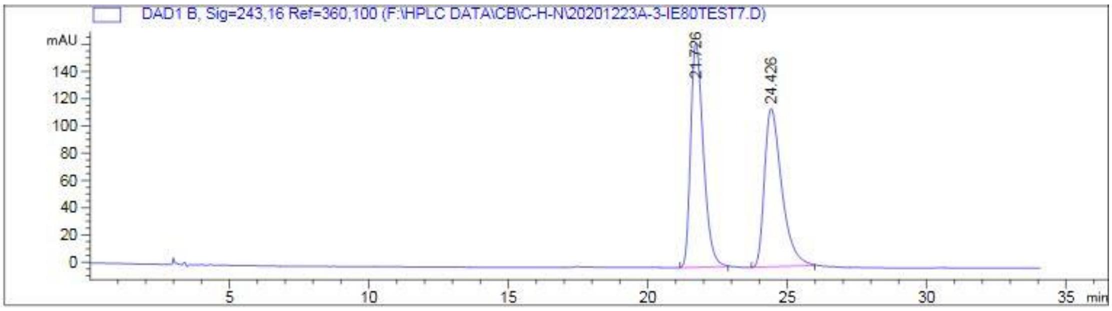

Peak #	RetTime [min]	Type	Width [min]	Area [mAU*s]	Height [mAU]	Area %
1	21.726	BB	0.4898	5220.98828	164.72147	50.9865
2	24.426	BB	0.6540	5018.96143	116.17943	49.0135

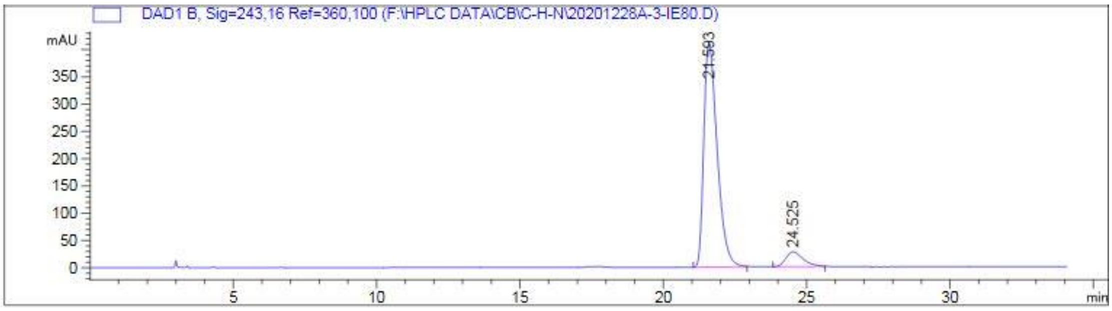

Peak #	RetTime [min]	Type	Width [min]	Area [mAU*s]	Height [mAU]	Area %
1	21.593	BB	0.4968	1.32684e4	410.81720	91.9866
2	24.525	BB	0.6538	1155.87793	27.19721	8.0134

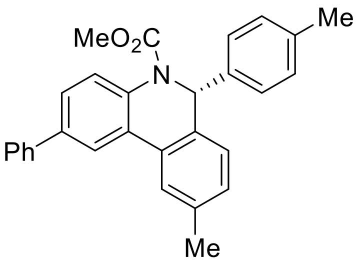

4t (The top one is racemic, and the bottom one is chiral)

The enantiomeric excess was determined by HPLC analysis using a chiral stationary phase column [Daicel chiracel® OD-H, 243 nm, n-hexane : i-PrOH = 98 : 2 as the eluent, flow rate: 1 mL/min, temperature 25 °C, retention time: 12.2 min (major isomer) and 9.2 min (minor isomer)].

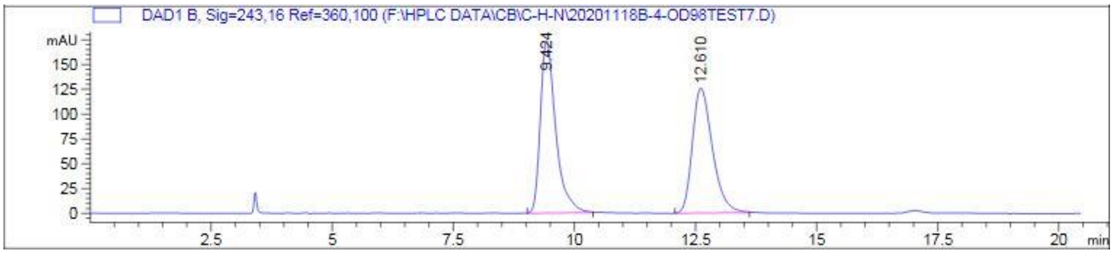

Peak #	RetTime [min]	Type	Width [min]	Area [mAU*s]	Height [mAU]	Area %
1	9.424	BB	0.3337	3837.44531	173.73486	51.4040
2	12.610	BB	0.4421	3627.81445	125.91930	48.5960

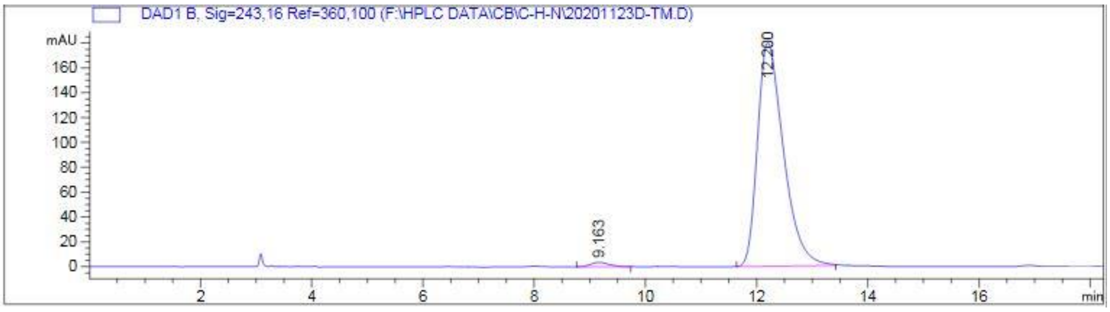

Peak #	RetTime [min]	Type	Width [min]	Area [mAU*s]	Height [mAU]	Area %
1	9.163	BB	0.3592	85.12730	3.50673	1.4376
2	12.200	BB	0.4966	5836.55566	179.85576	98.5624

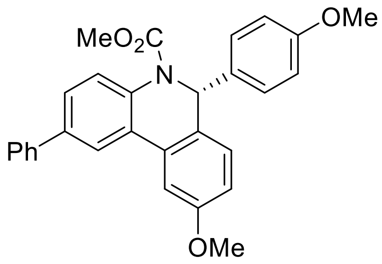

4u (The top one is racemic, and the bottom one is chiral)

The enantiomeric excess was determined by HPLC analysis using a chiral stationary phase column [Daicel chiracel® IA-3, 243 nm, n-hexane : i-PrOH = 85 : 15 as the eluent, flow rate: 1 mL/min, temperature 25 °C, retention time: 12.3 min (major isomer) and 15.4 min (minor isomer)].

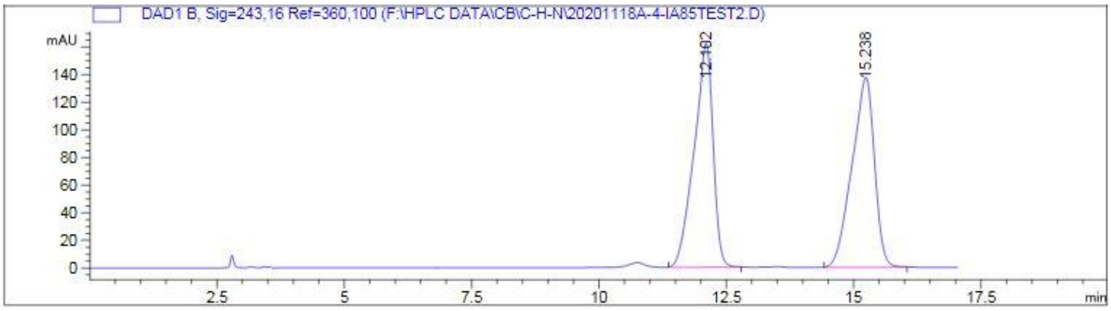

Peak #	RetTime [min]	Type	Width [min]	Area [mAU*s]	Height [mAU]	Area %
1	12.102	BB	0.3702	4210.54199	162.54941	49.9574
2	15.238	BB	0.4412	4217.72803	137.77527	50.0426

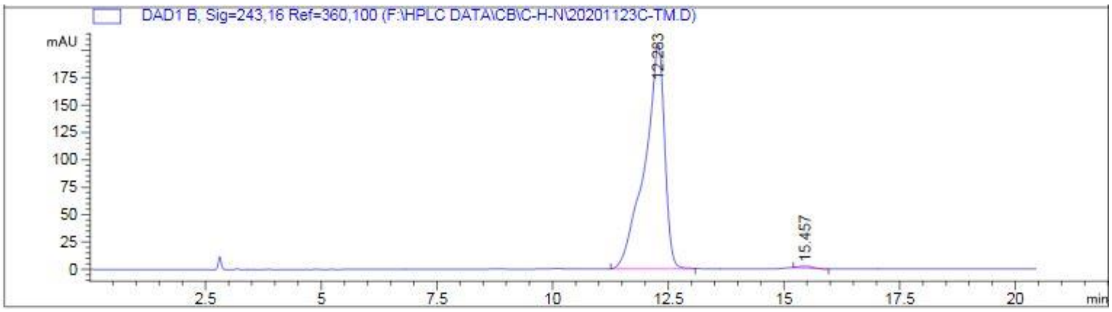

Peak #	RetTime [min]	Type	Width [min]	Area [mAU*s]	Height [mAU]	Area %
1	12.283	BB	0.4190	6132.77393	205.02544	99.4727
2	15.457	BB	0.2920	32.50900	1.69324	0.5273

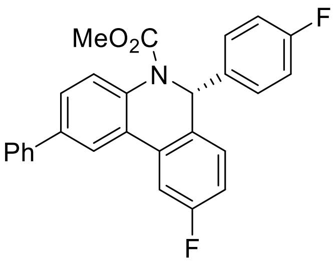

4v (The top one is racemic, and the bottom one is chiral)

The enantiomeric excess was determined by HPLC analysis using a chiral stationary phase column [Daicel chiracel® IA-3, 254 nm, n-hexane : i-PrOH = 95 : 5 as the eluent, flow rate: 1 mL/min, temperature 25 °C, retention time: 11.0 min (major isomer) and 14.8 min (minor isomer)].

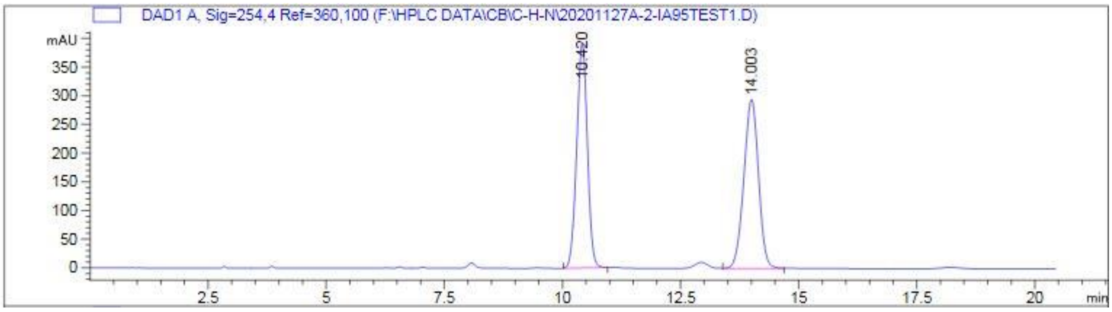

Peak #	RetTime [min]	Type	Width [min]	Area [mAU*s]	Height [mAU]	Area %
1	10.420	BB	0.2427	6146.91895	392.46277	49.2212
2	14.003	VB	0.3332	6341.44238	294.53146	50.7788

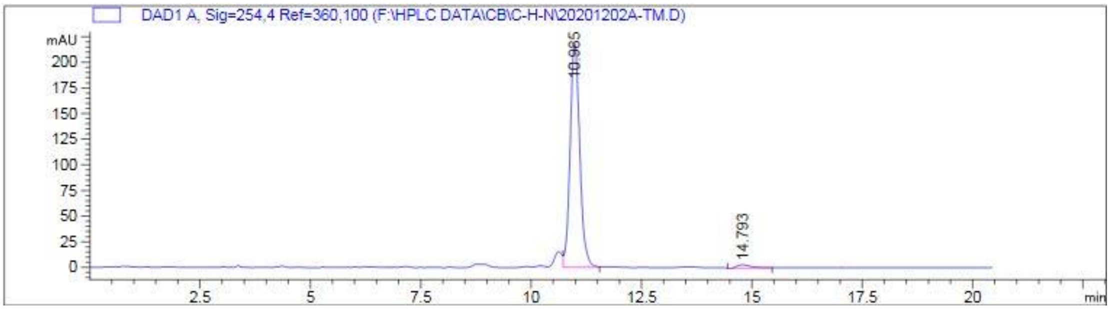

Peak #	RetTime [min]	Type	Width [min]	Area [mAU*s]	Height [mAU]	Area %
1	10.985	VB	0.2347	3313.91138	218.75842	98.1479
2	14.793	BB	0.3102	62.53663	3.08872	1.8521

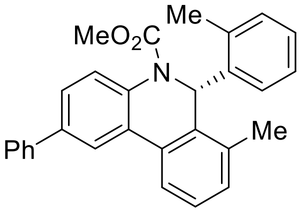

4w (The top one is racemic, and the bottom one is chiral)

The enantiomeric excess was determined by HPLC analysis using a chiral stationary phase column [Daicel chiracel® IA-3, 254 nm, n-hexane : i-PrOH = 95 : 5 as the eluent, flow rate: 1 mL/min, temperature 25 °C, retention time: 5.8 min (major isomer) and 6.4 min (minor isomer)].

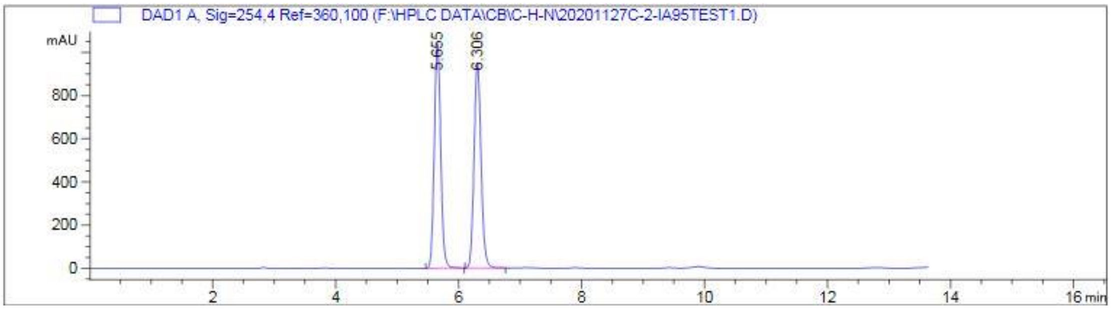

Peak #	RetTime [min]	Type	Width [min]	Area [mAU*s]	Height [mAU]	Area %
1	5.655	BB	0.1125	7566.42041	1044.65710	49.9458
2	6.306	BB	0.1229	7582.82910	951.80859	50.0542

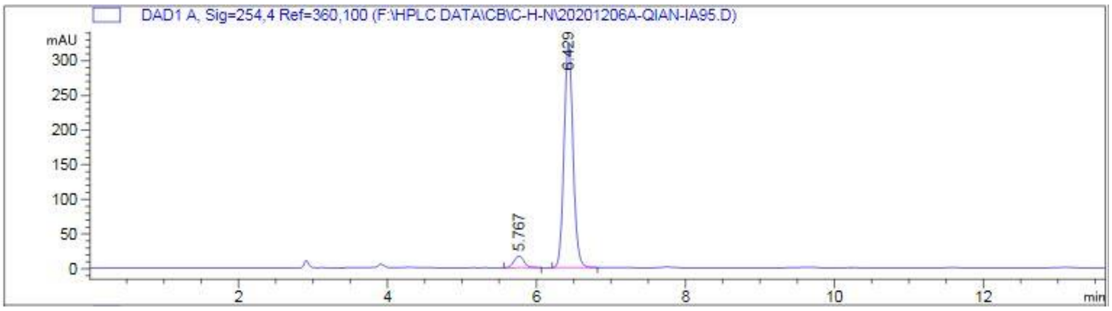

Peak #	RetTime [min]	Type	Width [min]	Area [mAU*s]	Height [mAU]	Area %
1	5.767	BB	0.1513	159.93271	16.77023	5.5666
2	6.429	BB	0.1295	2713.12622	324.67505	94.4334

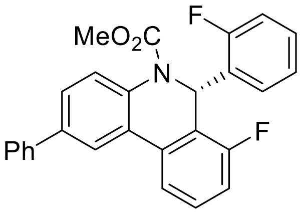

4x (The top one is racemic, and the bottom one is chiral)

The enantiomeric excess was determined by HPLC analysis using a chiral stationary phase column [Daicel chiracel® IA-3, 254 nm, n-hexane : i-PrOH = 95 : 5 as the eluent, flow rate: 1 mL/min, temperature 25 °C, retention time: 15.2 min (major isomer) and 9.4 min (minor isomer)].

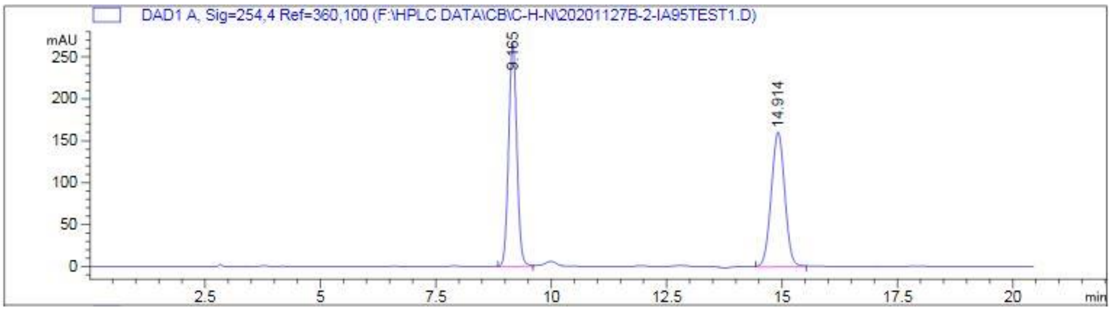

Peak #	RetTime [min]	Type	Width [min]	Area [mAU*s]	Height [mAU]	Area %
1	9.165	BB	0.1931	3315.28271	266.63293	50.3559
2	14.914	BB	0.3196	3268.41553	160.47038	49.6441

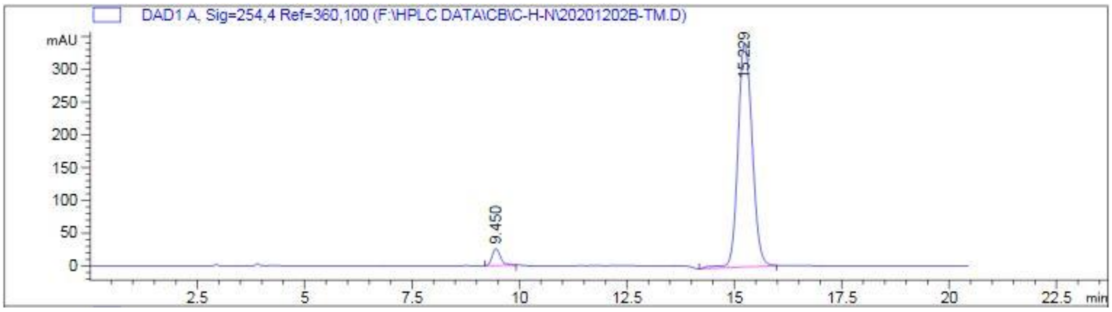

Peak #	RetTime [min]	Type	Width [min]	Area [mAU*s]	Height [mAU]	Area %
1	9.450	BB	0.2112	351.36853	25.75619	4.2766
2	15.229	BB	0.3626	7864.73389	341.61551	95.7234

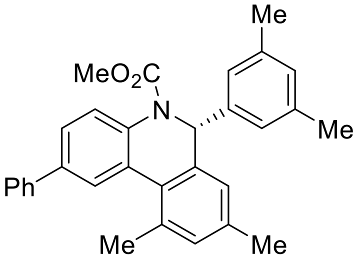

4y (The top one is racemic, and the bottom one is chiral)

The enantiomeric excess was determined by HPLC analysis using a chiral stationary phase column [Daicel chiracel® IC-3, 243 nm, n-hexane : i-PrOH = 99 : 1 as the eluent, flow rate: 1 mL/min, temperature 25 °C, retention time: 17.0 min (major isomer) and 21.9 min (minor isomer)].

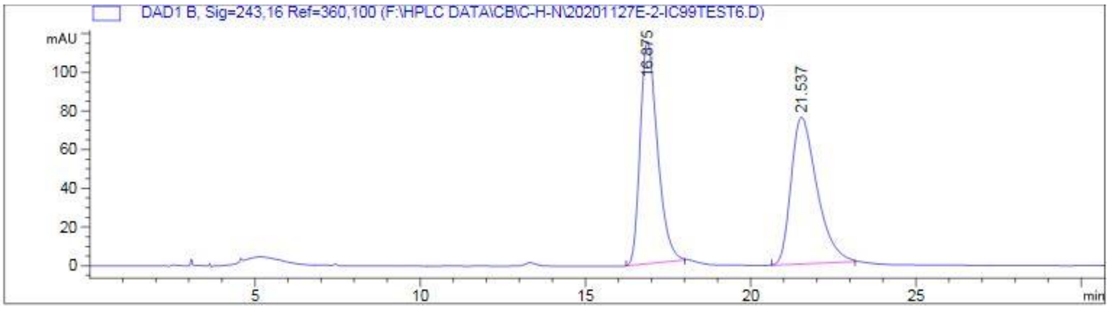

Peak #	RetTime [min]	Type	Width [min]	Area [mAU*s]	Height [mAU]	Area %
1	16.875	BB	0.5541	4178.14160	114.92681	50.2508
2	21.537	BB	0.8203	4136.44092	75.84449	49.7492

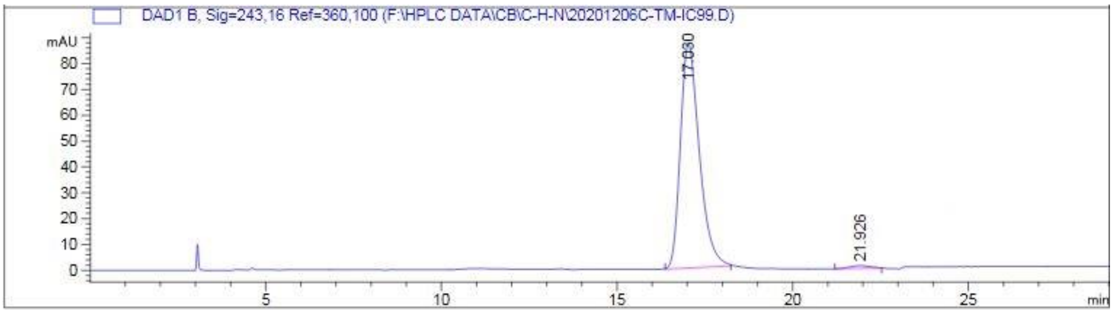

Peak #	RetTime [min]	Type	Width [min]	Area [mAU*s]	Height [mAU]	Area %
1	17.030	BB	0.5723	3232.99951	86.44284	98.7045
2	21.926	MM	0.7167	42.43206	9.86781e-1	1.2955

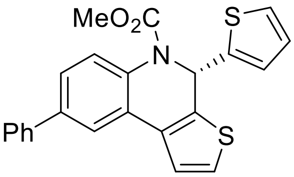

4z (The top one is racemic, and the bottom one is chiral)

The enantiomeric excess was determined by HPLC analysis using a chiral stationary phase column [Daicel chiracel® OD-H, 254 nm, n-hexane : i-PrOH = 80 : 20 as the eluent, flow rate: 1 mL/min, temperature 25 °C, retention time: 20.2 min (major isomer) and 7.2 min (minor isomer)].

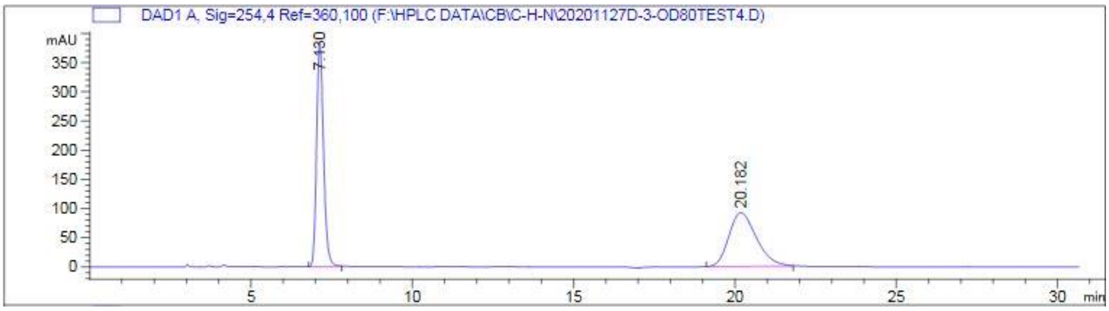

Peak #	RetTime [min]	Type	Width [min]	Area [mAU*s]	Height [mAU]	Area %
1	7.130	BB	0.2280	5715.11865	383.17200	51.0081
2	20.182	BB	0.9124	5489.22021	92.22006	48.9919

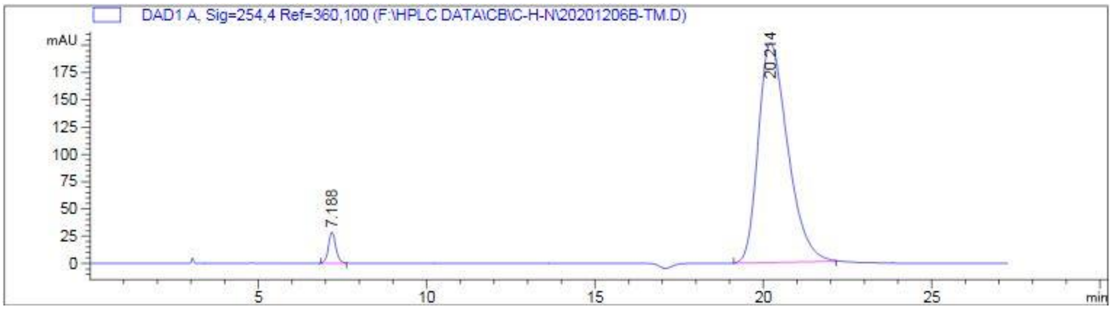

Peak #	RetTime [min]	Type	Width [min]	Area [mAU*s]	Height [mAU]	Area %
1	7.188	BB	0.2351	437.46518	28.50156	3.4538
2	20.214	BB	0.9306	1.22288e4	201.34999	96.5462

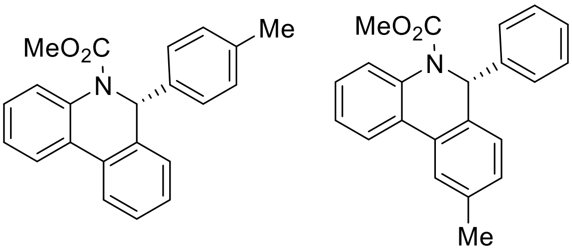

8a/8b (The top one is racemic, and the following part is chiral)

The enantiomeric excess was determined by HPLC analysis using a chiral stationary phase column [Daicel chiracel® IB-3, 237 nm, n-hexane : i-PrOH = 95 : 5 as the eluent, flow rate: 1 mL/min, temperature 25 °C, retention time: 6.5 min (major isomer) and 5.7 min (minor isomer); 11.1 min (major isomer) and 7.2 min (minor isomer)].

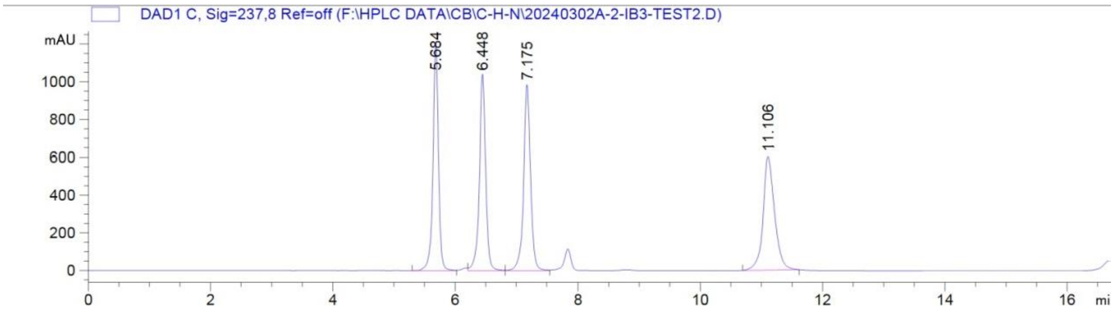

Peak #	RetTime [min]	Type	Width [min]	Area [mAU*s]	Height [mAU]	Area %
1	5.684	BV	0.0927	7545.94189	1210.74438	24.5950
2	6.448	VV	0.1075	7459.19043	1041.89038	24.3123
3	7.175	VV	0.1160	7772.70557	985.71527	25.3341
4	11.106	MM	0.2180	7902.90723	604.29352	25.7585

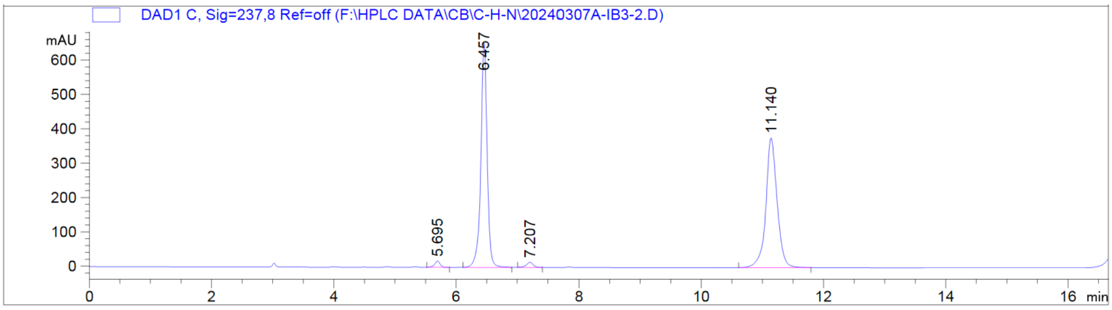

Peak #	RetTime [min]	Type	Width [min]	Area [mAU*s]	Height [mAU]	Area %
1	5.695	BB	0.0924	115.29552	18.58435	1.1814
2	6.457	BB	0.1073	4675.64111	655.24274	47.9086
3	7.207	BB	0.1173	118.18682	15.09967	1.2110
4	11.140	BB	0.1917	4850.38916	377.99420	49.6991

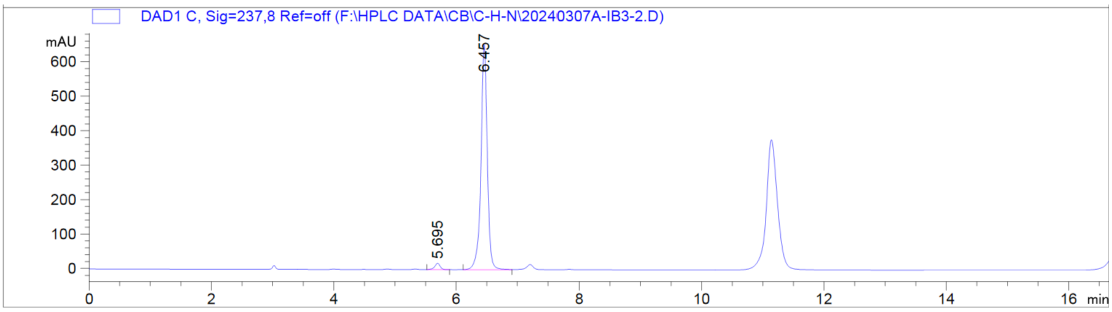

Peak #	RetTime [min]	Type	Width [min]	Area [mAU*s]	Height [mAU]	Area %
1	5.695	BB	0.0924	115.29552	18.58435	2.4065
2	6.457	BB	0.1073	4675.64111	655.24274	97.5935

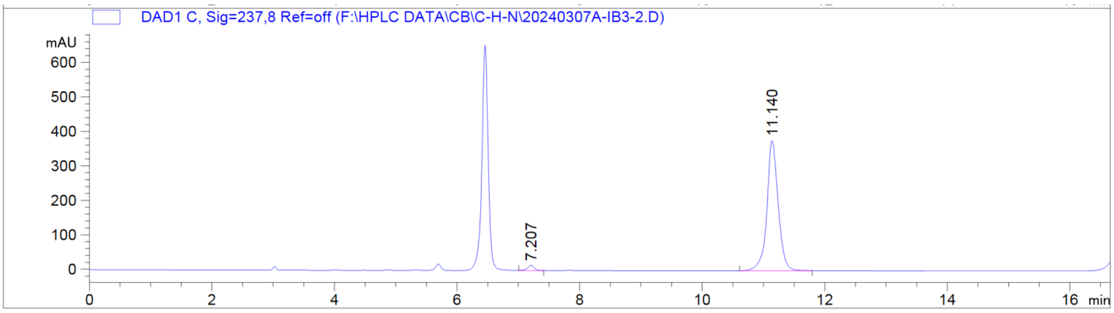

Peak #	RetTime [min]	Type	Width [min]	Area [mAU*s]	Height [mAU]	Area %
1	7.207	BB	0.1173	118.18682	15.09967	2.3787
2	11.140	BB	0.1917	4850.38916	377.99420	97.6213

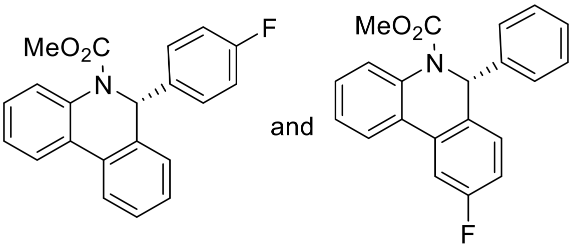

9a/9b (The top one is racemic, and the following part is chiral)

The enantiomeric excess was determined by HPLC analysis using a chiral stationary phase column [Daicel chiracel® IB-3, 254 nm, n-hexane : i-PrOH = 95 : 5 as the eluent, flow rate: 1 mL/min, temperature 25 °C, retention time: 7.0 min (major isomer) and 6.2 min (minor isomer); 10.8 min (major isomer) and 7.5 min (minor isomer)].

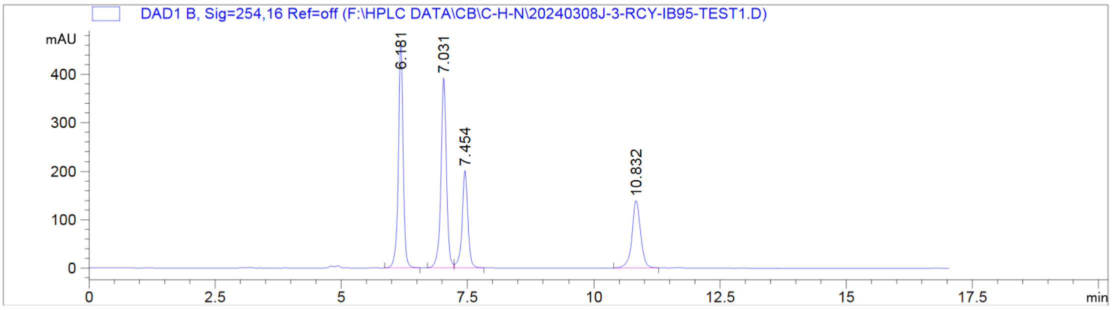

Peak #	RetTime [min]	Type	Width [min]	Area [mAU*s]	Height [mAU]	Area %
1	6.181	BB	0.0988	3074.74121	466.95541	32.8144
2	7.031	BV	0.1148	2990.67017	393.00119	31.9172
3	7.454	VB	0.1198	1624.34790	201.99413	17.3354
4	10.832	BB	0.1804	1680.33740	139.60481	17.9330

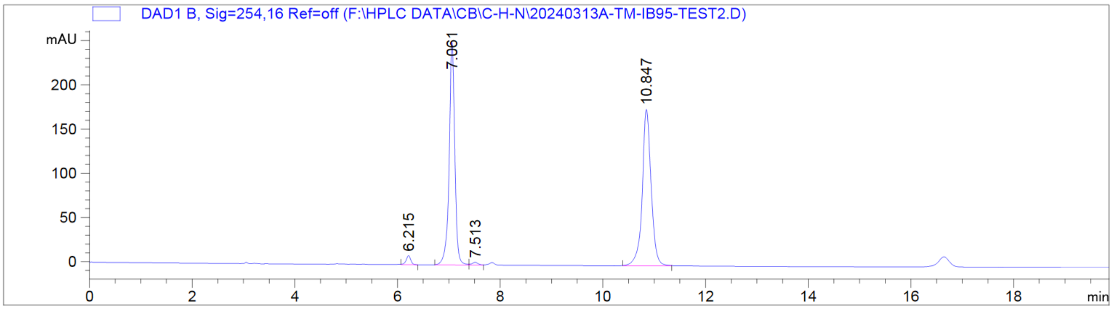

Peak #	RetTime [min]	Type	Width [min]	Area [mAU*s]	Height [mAU]	Area %
1	6.215	BB	0.0959	65.49081	10.33287	1.5757
2	7.061	BB	0.1132	1929.11633	252.32632	46.4153
3	7.513	BV	0.1260	26.50812	3.09367	0.6378
4	10.847	BB	0.1808	2135.09668	176.91974	51.3712

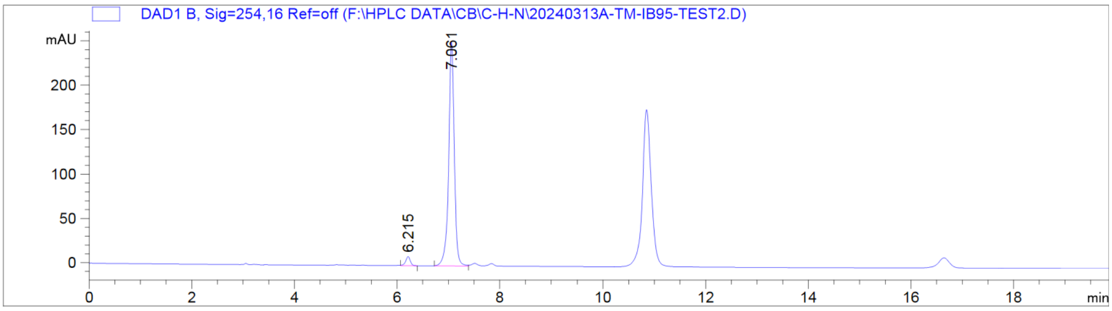

Peak #	RetTime [min]	Type	Width [min]	Area [mAU*s]	Height [mAU]	Area %
1	6.215	BB	0.0959	65.49081	10.33287	3.2834
2	7.061	BB	0.1132	1929.11633	252.32632	96.7166

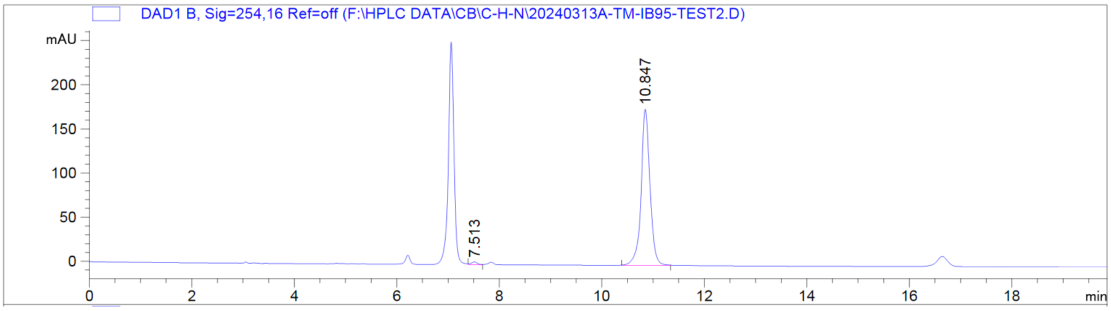

Peak #	RetTime [min]	Type	Width [min]	Area [mAU*s]	Height [mAU]	Area %
1	7.513	BV	0.1260	26.50812	3.09367	1.2263
2	10.847	BB	0.1808	2135.09668	176.91974	98.7737

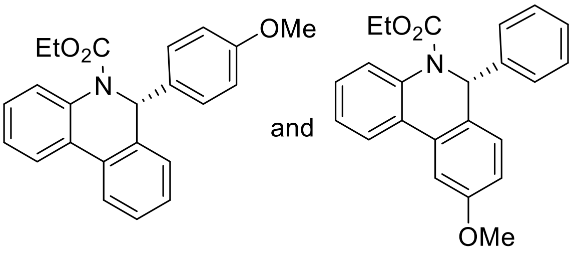

10a/10b (The top one is racemic, and the following part is chiral)

The enantiomeric excess was determined by HPLC analysis using a chiral stationary phase column [Daicel chiracel® IC-3, 254 nm, n-hexane : i-PrOH = 95 : 5 as the eluent, flow rate: 1 mL/min, temperature 25 °C, retention time: 12.9 min (major isomer) and 9.8 min (minor isomer); 11.1 min (major isomer) and 10.4 min (minor isomer)].

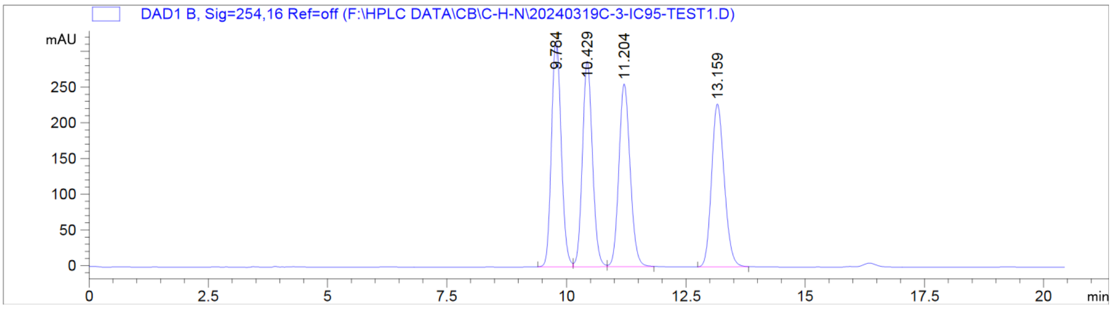

Peak #	RetTime [min]	Type	Width [min]	Area [mAU*s]	Height [mAU]	Area %
1	9.784	BV	0.2094	4246.86426	314.78024	25.0808
2	10.429	VV	0.2269	4216.91064	287.93890	24.9039
3	11.204	VB	0.2546	4222.56104	255.84023	24.9373
4	13.159	BB	0.2871	4246.37451	228.17883	25.0779

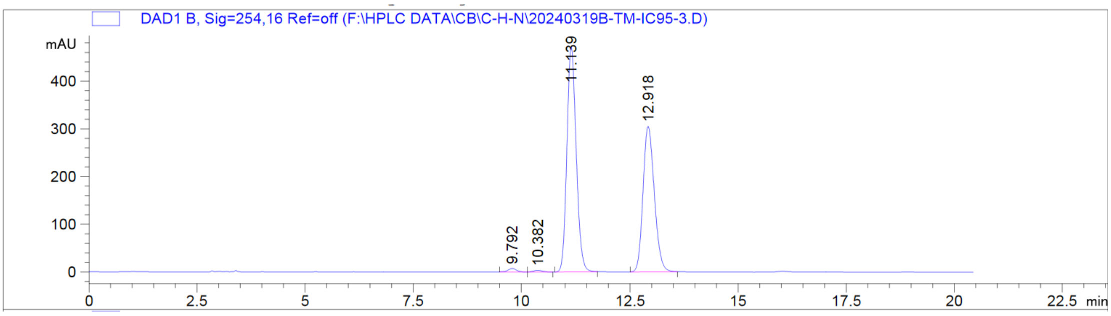

Peak #	RetTime [min]	Type	Width [min]	Area [mAU*s]	Height [mAU]	Area %
1	9.792	BB	0.2082	102.51746	7.56072	0.8027
2	10.382	BB	0.2123	49.58436	3.60820	0.3882
3	11.139	BB	0.2324	7057.34717	471.98584	55.2556
4	12.918	BB	0.2826	5562.74658	305.26059	43.5536

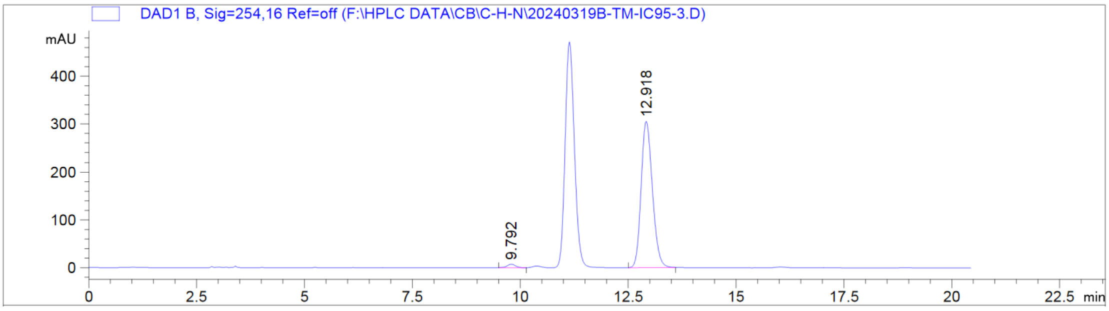

Peak #	RetTime [min]	Type	Width [min]	Area [mAU*s]	Height [mAU]	Area %
1	9.792	BB	0.2082	102.51746	7.56072	1.8096
2	12.918	BB	0.2826	5562.74658	305.26059	98.1904

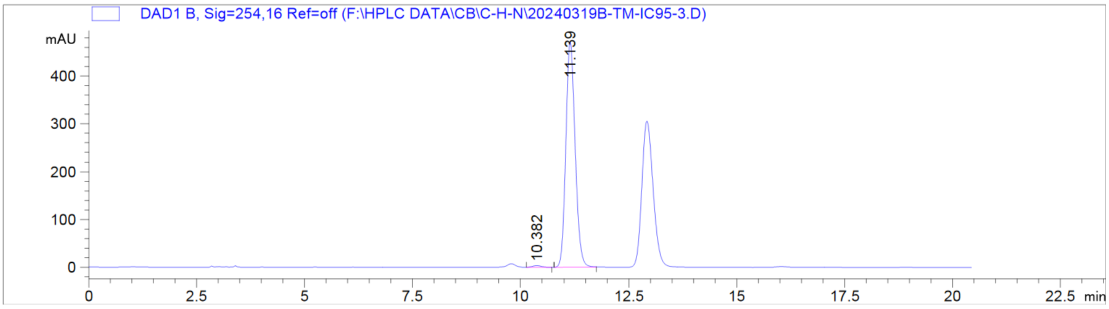

Peak #	RetTime [min]	Type	Width [min]	Area [mAU*s]	Height [mAU]	Area %
1	10.382	BB	0.2123	49.58436	3.60820	0.6977
2	11.139	BB	0.2324	7057.34717	471.98584	99.3023
